# Enhancement of Operational Safety in Marine Cargo Cranes on a Container Ship Through the Application of Authenticated Wi-Fi Based Wireless Data Transmission from Multiple Sensors

**DOI:** 10.3390/s24216799

**Published:** 2024-10-23

**Authors:** Mostafa Abotaleb, Janusz Mindykowski

**Affiliations:** 1 Doctoral School, Gdynia Maritime University, Morska 81-87, 81-225 Gdynia, Poland; m.abotaleb@sd.umg.edu.pl; 2 Department of Ship Electrical Power Engineering, Faculty of Marine Electrical Engineering, Gdynia Maritime University, Morska 81-87, 81-225 Gdynia, Poland

**Keywords:** marine cargo cranes, wireless, Wi-Fi, ESP32, ESP-NOW, WebSerial, predictive maintenance, functional safety, economic efficiency

## Abstract

The use of wireless technology in common marine engineering applications as a medium for data transaction in measurement and control systems, is not as popular as it should be. This article aims to demonstrate the advantages of using wireless technology in maritime engineering applications through a proposed Wi-Fi based wireless system dedicated to performance and safety monitoring in marine cargo cranes. The system is based on some concepts that were suggested in the earlier literature to perform authenticated data transmission from multiple sensors through using both the ESP-NOW protocol and the WebSerial remote serial monitor. The introduced system will be integrated with an already installed system in order to render the means for implementing effective principles in automation and control engineering, such as functional safety and predictive maintenance. Additionally, this article will highlight the economic efficiency of adopting wireless technology instead of cabling as a medium for data transaction in measurement and control systems in marine engineering applications such as cargo cranes.

## 1. Introduction

In maritime engineering facilities, heavy lifting equipment is considered one of the most important systems. Its major vital role in cargo loading/discharging operations is a clear manifestation of such an importance. On commercial ships such as bulk carriers, general cargo and container ships, cargo cranes undertake the task of handling the loaded/discharged cargo through an agreement with the port authorities at which the ship will exist. At some ports, this agreement stipulates that the port staff will take on the loading/discharging jobs using port cranes which are usually equipped with higher rated power for hydraulic and electro-hydraulic machinery as well as more sophisticated safety and control systems that increase the Safe Working Load (SWL) of such cranes so that cargo handling operations can be executed smoothly, efficiently and most importantly, in shorter periods of time. In other conditions, the ship’s cargo cranes might carry out the loading/discharging operations in case of the unavailability of cranes at the port, which naturally will cost an additional price paid by the cargo supplier to the shipping company owning the vessel. This price naturally will be higher than the price charged for the same cargo if it would have been loaded/discharged by the port cranes. Accordingly, the cargo cranes can be regarded as an additional resource of financial profit for the shipping company owning the vessel. Therefore, periodic maintenance for the cargo cranes should be carried out regularly and strictly by the ship’s staff in order to keep such a piece of equipment in its best possible functional condition. Commitment to the schedule of a periodic regular maintenance plan with the utmost degrees of discipline is a critical factor to reduce the downtime of any possible failure during cargo handling operations. In some cases, and due to the natural drawback of aging on some ships, periodic maintenance of the cargo crane electro-hydraulic system might not render the adequate required proper outcome, preventing possible future troubles. Periodic routine maintenance is naturally carried out when the equipment is not in operational condition, which means that some variables which are measured only during cargo operation are not taken into account. On-load hydraulic pressure, increasing temperatures of hydraulic oil during handling heavier loads and other parameters are examples of such variables that might not be taken into account during periodic maintenance. Therefore, monitoring such variants during cargo operation can result in an improved performance in conjunction with a modifiable maintenance schedule based on the data collected during the monitoring process, which can be considered an application for the principle of predictive maintenance (PdM). On the other hand, the application of other principles, such as economic efficiency and functional safety, are highly recommended to be taken into consideration while upgrading a conventional classic marine measurement/control system, or during the planning for the installation of a new one. Wireless technology is a reliable as well as a cost effective alternative through which such essential and vital principles can be applied. Therefore, this article will introduce a Wi-Fi wireless performance and safety monitoring system dedicated to the cargo cranes on a container ship, based on which the previously mentioned essential principles (economic efficiency, functional safety and PdM) will be successfully implemented. The article will be divided into seven major divisions. The introduction, the marine cargo cranes’ technical description, explanation for the possible causes of fire incidents in marine cargo cranes, illustration of the construction of the developed wireless system as well as the measured parameters during the monitoring process, discussion about the system efficiency consisting of four sections, demonstration for the conclusions and future work.

Initially, Section 1 will render a brief summary for some of the major contributions achieved by other researchers who investigated various aspects related to the possibility of adopting wireless technology in marine engineering applications. Section 5.1, Section 5.2 and Section 5.3 of the discussion will emphasize the role played by the developed Wi-Fi based wireless performance and safety monitoring system dedicated to the cargo cranes in the implementation of economic efficiency, functional safety and PdM. Section 5.4 of the discussion will elaborately illustrate the most important features of the proposed system in relation to the contributions of other authors in the selected recent literature.

In [1], the authors identified some of the necessary requirements for successful implementation of the internet of things (IoT) in applications dedicated to marine environment monitoring. The main purpose of these requirements is to avoid the negative influence of harsh conditions in maritime environments on the stability, the reliability and the robustness of the wireless measurement process. Naturally, such requirements can also be applied, to a specific degree, on wireless measurement/control systems on commercial ships. Based on the available information in [2], the discussion will recommend using housing of high ingress protection (IP) rating for the ESP32 modules in order to improve the robustness of the developed wireless system. The discussion will also shed light on the advantage of low power consumption by the developed wireless system due to the utilization of ESP32 modules [3]. In [4], the authors rendered an organizational structure for the industry of maritime transport. The discussion will emphasize the significance of such a structure by manifesting the vital role played by governmental and educational local organizations, in cooperation with the International Maritime Organization (IMO) and the shipping companies, through considering the developed wireless performance and safety monitoring system discussed in this article as an example of the coordination between such organizations. In [5], the authors enlisted some of the examples of marine measurement and control systems atwhich wireless technology was applied for the purpose of measurement data transaction. The discussion will include additional specific applications such as the developed wireless system dedicated to cargo cranes and others as parts that can be included in such a list. From a point of view related to the general classification of marine measurement/control systems in [5], the discussion will include an additional categorization for these systems according to the criteria of their activity periods of time whether it was during sailing or berthing. As vibration is an intrinsic element characterizing the harsh marine environment among other elements, its probable negative effect on wireless measurement data transaction should be taken into account. Researchers in [6] have experimentally depicted the negative influence of high vibration levels on the path loss of RF waves propagation. The researchers have also recommended some preventive measures to avoid such an effect. The discussion will highlight such suggestions from a perspective linked to the classification of the marine measurement/control systems based on their periods of activity.

The system is based on a laboratory stand that was thoroughly described in [7]. This laboratory stand was constructed to perform authenticated wireless data transmission from multiple sensors using simultaneous serial and Wi-Fi based wireless communication tasks. The serial communication takes place between the ATMEGA625 microcontroller (to which the sensors are connected) and the ESP32 controller, which sends the received data from the sensors through wireless Wi-Fi communication to the host controller. As described in [8], there are two adopted techniques to send the collected measured data from multiple sensors wirelessly. The first technique is based on using only the WebSerial remote serial monitor, which is recommended for wireless communication in case of a clear line of sight (LOS) between the sensors’ location and the host controller’s location with minor metallic or infrastructural objects of relatively low heights. In the second technique, additional ESP32 modules play the role of wireless switches at positions where low RSSI (Received Signal Strength Indicator) levels are expected because of higher metallic or infrastructural objects. The ESP-NOW protocol is adopted by these switches in order to exchange messages between the ESP32 controller located at the sensors’ location and the ESP32 controller located at the host controller. On a container ship equipped with cranes, the second technique might be the most proper technique to implement the proposed wireless safety and performance monitoring system dedicated to the cargo cranes, as the extremely high density of metallic obstructions (crane structure and container units) will definitely lead to generally decreased RSSI levels at many locations [9]. The selected parameters to be monitored by the suggested system are critical key elements that will ensure an enhanced level of safety during operation as well as an efficient mechanism to predict possible future failures. The enhanced level of operational safety will be rendered through fire detection/alarming components that will notify the ship’s staff to respond in case of any possible fire incidents caused by the high temperature levels of hydraulic oil splashed on hot surfaces [10,11] or by electrical sparks. Other than the fire detection/alarm signal, the rest of the measured parameters will help the engineering personnel responsible for the crane maintenance to create a performance pattern in order to construct or modify the periodic maintenance schedules so that future failures can be avoided, which is the main purpose of applying the principle of predictive maintenance (PdM).

PdM has basically evolved after the emergence of Industry 4.0 (I4.0) [12,13]. Machine Learning (ML) and Deep Learning (DL) are considered approaches to guarantee the successful implementation of PdM.IoT can be considered a tool facilitating the functionality of these approaches with lower costs and better efficiency. An example of PdM is the creation of algorithms or mathematical models comparing historical and recent data collected by a specific sensor. This technique is called Supervised lLearning (ML method). Another example of PdM is to carry out analysis for data sequences collected over specific, consistent periods of time. This technique is called Time Series Analysis (DL method). Alarm IDs and their timestamps included in event data logs are very important tools to apply both machine learning and deep learning. These data logs are created by means of a software tool collecting data from multiple sensors or repeaters and sending it from the sensors’ location to the database analysis center (host controller). These data transactions can take place through cabling or wirelessly (IoT).

The crucial disadvantage of adopting only scheduled maintenance is the increased cost required for the replacement of some elements in the system before its due service lifetime [14]. On the other hand, scheduled maintenance can be preventive only to a specific extent as it would not have prevented the occurrence of some failures which might take place in the future due to the absence of monitoring of the relations between some important variables or parameters in the system. These parameters or variables are only monitored separately, indicating alarming states in each of them without further calculations for the relations between them. In order to provide more understanding for such a notion in conjunction with the discussed system in this article (marine cargo crane), the relation between hydraulic oil feed pressure and temperature should be taken into account (Vogel equation), as the dynamic viscosity of the hydraulic oil [15,16] is dependent on such a relation between pressure and temperature.

Since this article is proposing the use of RF waves as a medium for data transaction in measurement and control systems, additional light will be shed on possible partial implementation of the functional safety principle if both cabling and RF waves were simultaneously as well as redundantly adopted as data transaction mediums. An improved Safety Integrity Level (SIL) is a key factor in implementing the principle of functional safety. It can be achieved by the application of redundant decomposition for the various elements of measurement/control systems [17,18]. Multichannel architecture is an evident demonstration for such a concept of redundancy [19].

Each of the measured parameters at the developed wireless performance and safety monitoring system will be described thoroughly in the discussion in order to provide an adequate comprehensive understanding for readers who are not strongly affiliated with such an engineering application. Afterwards, the proposed wireless system will be described from a perspective linked to its cost effectiveness as well as its vital role in the implementation of functional safety and predictive maintenance principles. The discussion will include a comparative cost analysis (Section 5.1) for the system to manifest the achieved economic efficiency by using a wireless medium for data transaction ratherthan cabling. The analysis will be based on calculating the cost of two types of instrumentation cables, if the same system was assumed to be implemented using cables.

Section 5.2 of the discussion will demonstrate the partial implementation of functional safety if it is supposed that the developed system would have adopted a technique of redundant pairing for the measurement data transaction medium.

Section 5.3 of the discussion will be dedicated to the derivation of a mathematical model depicting the implementation of PdM through processing the stored measured values in the performance log of the two most critical measured quantities (hydraulic oil feed pressure and temperature) during each working hour in order to create a pattern for the calculated dynamic viscosity of hydraulic oil, and comparing it with dynamic viscosity reference values found in charts provided by the crane manufacturer or hydraulic oil supplier. The model will also provide a technique for creating a smaller-size database for the working hours during which the difference between the calculated and reference dynamic viscosity values was greater than a specific preset critical value.

Section 5.4 of the discussion will illustrate the most important features of the developed wireless performance and safety monitoring system in relation to the contributions of other authors in the selected recent literature. Such an integrating analysis will highlight the elements of novelty in this article as a demonstration of the progress of the scientific research related to the different possibilities of utilizing wireless technology in measurement and control systems on commercial ships, supported by the classifications, the conclusions and the recommendations rendered by other researchers.

## 2. Marine Cargo Cranes

As previously mentioned, cargo cranes are one of the most important pieces of equipment on any commercial ship. The simplified basic principle of its operation is the conversion of high hydraulic oil pressures into three types of movements. These movements are luffing, hoisting and slewing. There are three hydraulic pumps as well as three hydraulic motors dedicated to each of these movements. The main hydraulic oil feed pump driven by a three-phase electrical motor produces the required hydraulic feed pressure to drive the hydraulic pumps dedicated to each type of movement. Each of these hydraulic pumps provide the required pressure to rotate each of the hydraulic motors dedicated to each type of movement through converting the applied output pressure by the pumps into rotational movement with suitable torque for each movement. Control is provided for the electro-hydraulic circuits (dedicated to each of these movements) by means of electro-hydraulic valves. In order to provide more understanding here for the non-specialized readers, further explanation should be provided for the word (electro-hydraulic). The electro-hydraulic circuit can be defined as a circuit based on two types of control lines. The first line is the hydraulic line, while the second line is the electrical line. The electrical circuit activates/deactivates electro-hydraulic valves, the role of which is to deliver/cut the hydraulic control pressure between two points. Each hydraulic pump produces the required hydraulic oil pressure required to rotate each of the hydraulic motors dedicated to each type of the previously mentioned movements. The flow rate produced by each of these pumps is controlled by the position as well as the voltage detected by the operator lever (joystick). The flow rate produced by the pump affects the speed of rotation of the hydraulic motor, while the pressure produced by the hydraulic pump affects the torque of the hydraulic motor. The more flow rate applied by the hydraulic pump, the higher the rotational speed of the hydraulic motor. On the other hand, the higher the pressure applied to the motor by the hydraulic pump, the higher the torque of the hydraulic motor. High torque levels reflect the capability of the hydraulic motor to handle heavier loads. Consequently, in most marine cargo cranes, it can be noticed that the hydraulic pumps and motors dedicated to luffing and hoisting movements are usually larger than the hydraulic pumps and motors dedicated to slewing movement. This can be explained, as the highest portion of the handled weight by the crane is lifted by both the luffing and hoisting winches.

Control levers (joysticks) can be considered potentiometers. The voltage applied to the non-varying terminals of these potentiometers is usually in the range of 12 VDC. At the neutral position of the control lever, the detected voltage at the potentiometer middle-varying terminal is almost 6 VDC. This voltage increases or decreases with respect to the other two fixed points of the potentiometer, when the control lever is moved by the operator in one of two directions. In one of the most popular marine cargo cranes, there is usually only one control lever dedicated to hoisting movement and another control lever dedicated to both luffing and slewing movements.

Each of the three previously described winches (hoisting, luffing and slewing), has its own electro-hydraulic valve for the brakes. The brake valve is engaged when the control lever is at the neutral position. When the crane operator starts moving the lever from the neutral position, the brake valve will be energized, electrically disengaging the brakes in order to allow for the movement of the hydraulic motor.

**Figure 1:**
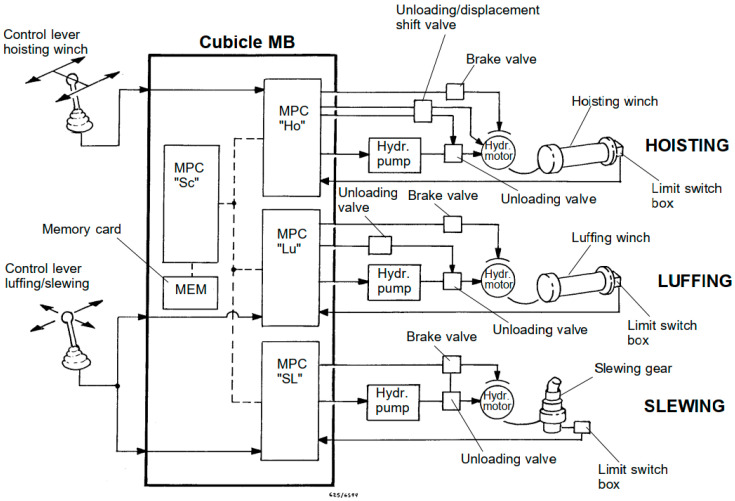
Marine cargo crane control system (Appendix A).

Automation and control tasks of marine cargo cranes can be carried out by means of classical regular control equipment such as various types of relays, Programmable Logic Controllers, PLCs, or by Micro-Processor Cards (MPCs), particularly designed for a specific model of cranes by a specific manufacturer. The first two choices of adopting classical control equipment or PLCs are usually not adopted by most of the marine crane manufacturers, as each company tends to preserve some kind of uniqueness for their own control systems in order to guarantee that the client or the buyer of the marine cargo crane will always return to the company if spare parts will be needed in the future, which can be considered an additional resource of economical profit for the marine crane manufacturer. In this article, the proposed wireless system was integrated with a conventional control system (already installed) for cargo cranes in which automation and control tasks are executed by 4 MPCs (Figure 1). Three MPCs are dedicated to control the hoisting, luffing and slewing circuits, while the fourth MPC plays the role of supervising the whole control system.

## 3. Fire Detection in Marine Cargo Cranes

The fire hazard in marine cargo cranes can be briefly summarized in two important points:

1. The high pressure levels of hydraulic oil can increase the possibility of fire outbreak in cases of possible interaction between the hydraulic oil sprays [10,11] (due to cut hoses or any kind of hydraulic failure) and other materials such as power cables or other hot surfaces.
2. The marine cargo cranes are usually supplied with electrical power by means of brushes and slip rings because of the slewing movement, which can twist the cables and eventually cut it if cables were supposed to be connected directly to the cranes. In cases of negligence or absence of periodic maintenance of the slip rings, this might lead to electrical sparks that might cause the outbreak of fire.

Accordingly, the marine cargo cranes are recommended to be equipped with fire detection equipment in order to guarantee rapid notification for the ship’s crew in case of fire incidents. The sooner the response for the detected fire notification signal (fire alarm), the shorter the required period of time to extinguish the fire.

## 4. The Proposed Wi-Fi Based Wireless System

The system was suggested to be installed on a container ship equipped with two cargo cranes. The full length of the ship is almost 140 m. As previously described in the Section 1, the system is based on the collaboration between the WebSerial remote serial monitor and ESP-NOW protocol. ESP-NOW protocol played the key role of exchanging messages between the transmission and reception points. The WebSerial remote serial monitor collected the transmitted data from the sensors located on the crane side through ESP-NOW switches. This technique is based on the second configuration adopted at [8] to enhance the range of Wi-Fi wireless based communication using the ESP32 controller. The container ship on which the system was applied is equipped with two cargo cranes. The first crane is located closer to the ship’s front (forward station), while the second crane is located closer to the ship’s accommodation and the bridge. The most proper location for the host controller receiving the wirelessly collected data isthe bridge (wheelhouse) as it is the highest point at the accommodation side and allows for better RF waves propagation with less obstacles such as infrastructural metallic objects or the loaded container units. In order to guarantee an increased reliability of Wi-Fi wireless communication, the ESP32 switches (exchanging messages using ESP-NOW protocol) were mounted at the following locations:

1. On the top of the second crane (ESP32 No.2);
2. On the bridge starboard STBD side wing (ESP32 No.3);
3. On the bridge port side wing (ESP32 No.4);
4. At the top of the navigation bridge deck (ESP32 No.5).

**Figure 2:**
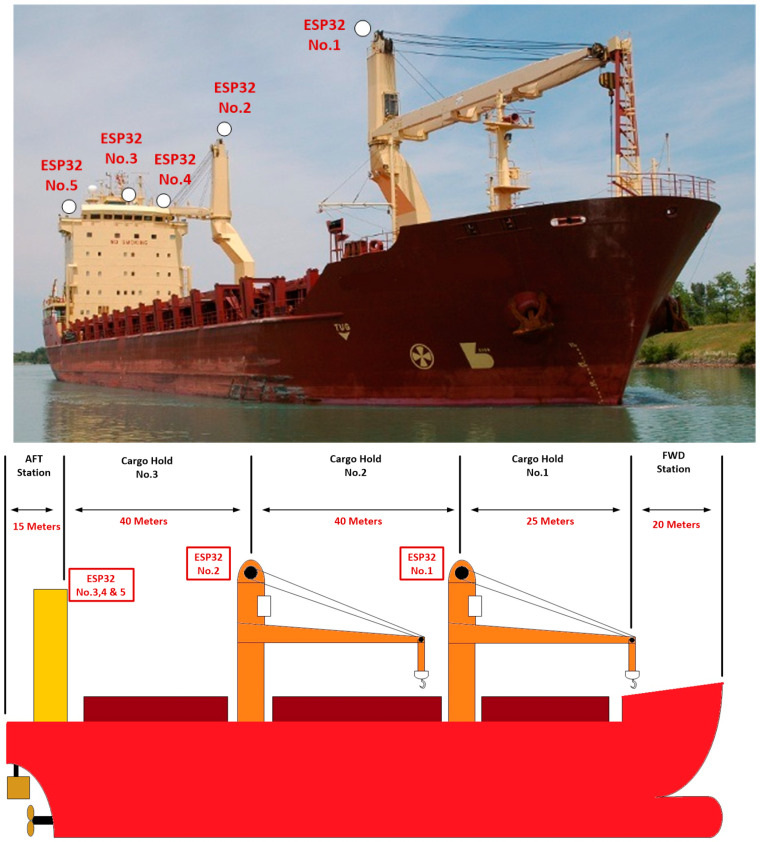
Illustration of the locations of the required ESP32 modules for the developed wireless system, in addition to an approximate dimensional drawing of the ship.

The first switch collected the measured data from the first crane and transmitted it to switches number 2, 3 and 4 through ESP-NOW protocol. This wireless switch performed a bit differently than switches 2, 3 and 4 as it additionally collected the measured data from the second crane (serial communication with Arduino controller where measured signals from the first crane were collected) and forwarded it to switches 2, 3 and 4. It also received authentication messages from the host controller to the sensors’ station at the crane. The purpose of using multiple switches in the previously mentioned locations is to ensure redundant wireless communication with low levels of distortion or attenuation caused by the movement of the heavy steel jib of the crane during cargo operation. Accordingly, the system depended mainly on five ESP32 modules (Figure 2) to perform the following wireless data transaction tasks:

1. ESP32 No.1: It collected the measured data from crane No.1 and forwarded it to the 1st switch at crane No.2 using ESP-NOW protocol.
2. ESP32 No.2 (1st Switch): It collected the measured data from crane No.2 and forwarded it to switches number 2, 3 and 4 using ESP-NOW protocol. It also forwarded the authentication messages from the host controller to crane No.1 using ESP-NOW protocol.
3. ESP32 No.3 (2nd Switch), ESP32 No.4 (3rd Switch) and ESP32 No.5 (4th Switch): They collected the data from modules No.1 and No.2 using ESP-NOW protocol and forwarded it to the host controller using the WebSerial remote serial monitor. They also forwarded the authentication data from the host controller to both modules No.1 and No.2.

The developed Wi-Fi based wireless system was dedicated to monitor some critical parameters at the crane to ensure improved performance during operation, shorter downtime in case of failure and a more specific planned maintenance schedule based on the observations collected from the performance monitoring log during operation. Table 1 illustrates these parameters with indication to its correspondent values during the crane idle state and also during the crane operational state.

In order to detect any probable fire incidents, it was proposed to install four fire detection/alarming devices at the following locations:

1. Manual call point at the crane operator cabin, which would help the crane operator to activate this point in case of noticing any fire.
2. Optical smoke detector at the location of the crane hoisting and luffing drums, to detect any potential fire incidents due to any hydraulic oil leak.
3. Optical smoke detector at the location of the electrical power supply cable entrance and the slip rings to detect any potential fire incidents caused by electrical sparks.
4. Manual call point at the crane entrance which could be activated by the crane observer during cargo operation.

**Figure 3:**
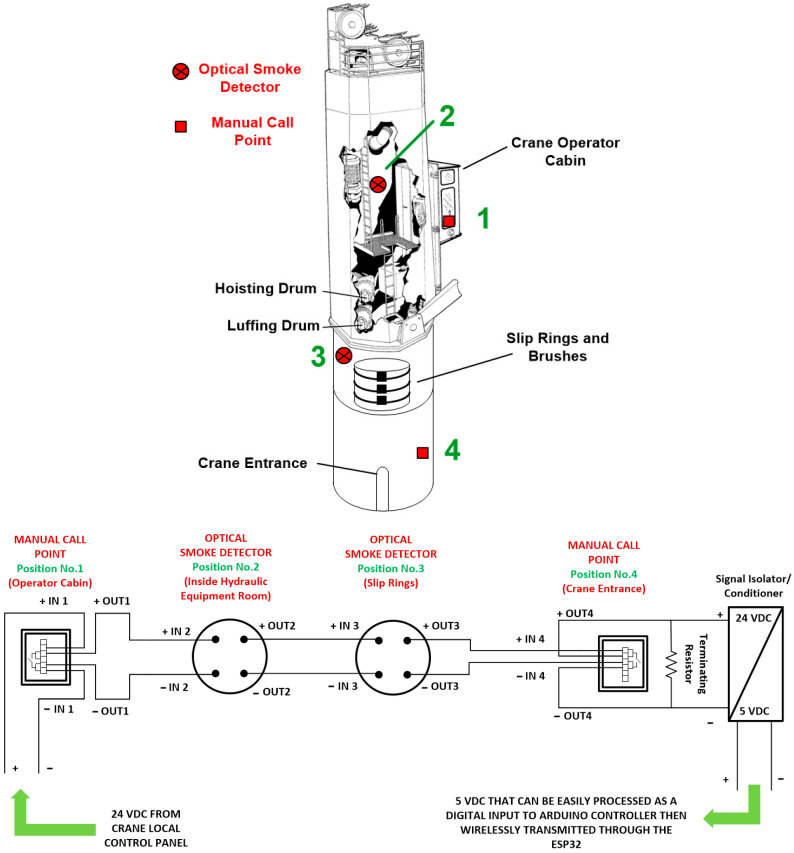
Illustration of the locations as well as the connection diagram of the optical smoke detectors (2 and 3) and manual call points (1 and 4) dedicated to fire detection (Appendix A).

The previously mentioned fire detection/alarming devices formed a single fire detection conventional loop and were fed as an input to the Arduino controller to detect any possible fire incidents. Figure 3 demonstrates the locations as well as the connection diagram of both optical smoke detectors No.2 and No.3, in addition to both manual call points No.1 and No.4.

In each cargo crane, there is a storage tank for the hydraulic oil fluid used in the electro-hydraulic circuit. Usually, there is a level transmitter or a level switch to activate an alarm signal in case of low fluid level at the tank. Moreover, in some cases, there is an additional level switch for the Low-Low fluid level of the tank. In case the Low-Low level switch is activated, it will not be possible for the crane to start due to the start–inhibit signal from the Low-Low level switch. In the developed wireless system, the hydraulic oil level signal will be only an alarming signal in order to notify the crane engineer to add some hydraulic oil to the storage tank.

The pressure and the temperature of the hydraulic oil used in the electro-hydraulic circuit are another two important parameters monitored by the developed wireless system. The hydraulic oil normal feed pressure is assumed to be in the range from 20 to 40 bars, so the system will generate an alarm if the detected feed pressure is lower than the lower range limit or higher than the upper range limit. For the hydraulic oil temperature, the system will generate an alarm in case the temperature exceeds the value of 60 Celsius degrees.

Without investigating deeply into the technical principles of hydraulic engineering, attention should be paid to distinguish between two terms, the hydraulic oil feed pressure and the hydraulic load pressure. The feed pressure is the output pressure to all hydraulic motors by the feed pump; however, the load pressure is the hydraulic oil pressure proportional to the weight being handled by the crane. In the crane model discussed in this article, the load pressure can reach levels up to 300 bars. The developed system will also monitor the hydraulic load pressure during hoisting of the handled weight by the crane. If the load pressure is higher than a specific value, the hoisting hydraulic motor will shift from operating with high speed to operating with lower speed as more torque will be required to handle loads of heavier weights.

**Figure 4:**
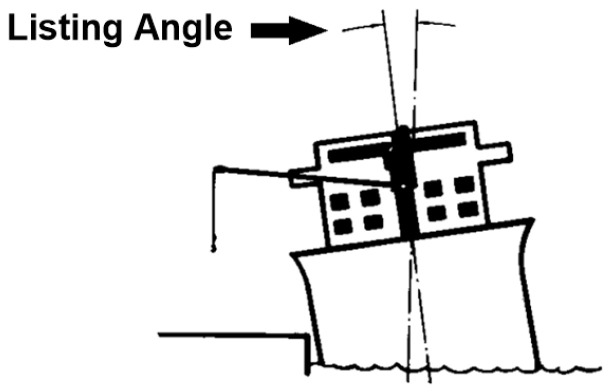
Listing angle (Appendix A).

The listing angle is the angle that the ship swerves from its rolling axis either to the starboard side or to the port side (Figure 4). In marine cargo cranes, it is highly recommended not to operate the crane if the listing angle is more than four degrees. The system will monitor the listing angle and generates an alarm as an indication for unsafe operation of the crane. The last two parameters monitored by the system will be the indication of brakes engagement/disengagement and the output of the crane operator lever (briefly described in the introduction).

**Figure 5:**
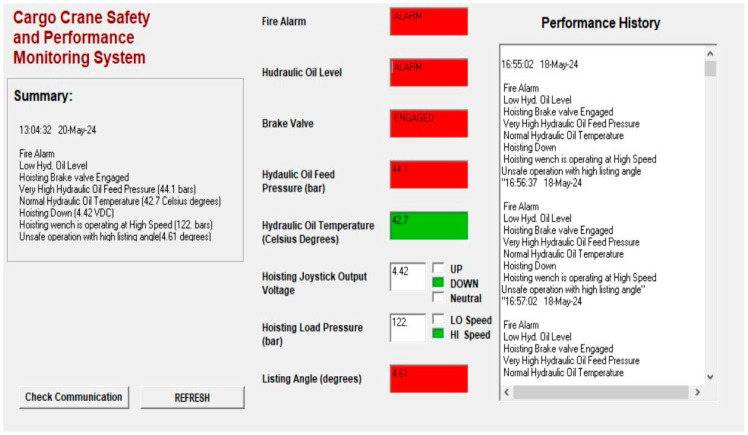
Demonstration of the system’s GUI (Graphical User Interface) during operation.

The system will keep a performance monitoring log for all the measured parameters. This log will provide the capability of creating charts for the measured values with respect to time. These charts will help the ship’s electro-technical officer to build or modify the maintenance schedules of the crane in order to search for remedies for any abnormalities observed in the charts created based on the available data in the performance monitoring log. The GUI (Graphical User Interface) of the system in Figure 5 demonstrates the successful test of the system indicating normal as well as alarm states of the previously explained parameters and measured variables, in addition to the stored data of the performance log.

## 5. Discussion

This article has provided a detailed analysis for a Wi-Fi based wireless system dedicated to the process of monitoring specific major parameters in a marine cargo crane as an example of the application of using wireless technology in maritime engineering. This wireless system was developed by the first author to be integrated with an already installed conventional measurement and control system in which control circuits are constructed using classical control devices such as different types of relays and supervised by Micro-Processor Cards (MPCs). Due to safety reasons related to the uniqueness of the environment where maritime engineering applications are installed, the transmission of measurement/control data using wireless technology is not very popular among maritime engineering companies as a sole medium for data transaction. In spite of this unpopularity, wireless technology can still find its own footing in maritime engineering as a cheap as well as an easy alternative for cabling rendering higher levels of operational safety for various types of critical marine equipment through the integration with conventional measurement/control systems.

### 5.1. Cost Analysis

The importance of the system lies in its cost effectiveness, implementing the principles of functional safety and predictive maintenance. The credit for such an economical efficiency goes to the reliance on wireless technology (Wi-Fi) instead of cabling. The developed wireless performance and safety monitoring system undertakes the task of scanning eight parameters. These parameters are the fire alarm signal, hydraulic oil tank level, hydraulic oil feed pressure, hydraulic oil temperature, hydraulic brake engaged/disengaged signal, hoisting joystick output voltage, hoisting load pressure and listing angle. Supposing that these signals were transmitted to the host controller at the engine room or at the bridge using cables and not using wireless technology, there would have been two options for cabling:

1. Using a single cable of two twisted pairs for each of the 8 variables, which means that the total number of cables will be 8 cables. The spare capacity in such a case will be 100%, as each signal will be transmitted through a twisted pair of wires, while the remaining twisted pair in the same cable will be considered as a spare pair in case of any future failure for the used twisted pair.
2. Using a single cable with 12 twisted pairs, 8 twisted pairs will be used for the whole 8 scanned variables and 4 twisted pairs used as spare wires in case of any failure for the already used pairs. The spare capacity in such a case will be 50%.

The uniqueness of the maritime environment naturally imposes additional preventive measures that should be taken into consideration to eliminate any possible negative influences on cables due to high levels of corrosion, temperature and vibration in marine engineering applications. Formation of ground loops or coupled noise to the wires of the cables are examples of such negative effects that will increase the error in the measured quantity. Accordingly, it is always recommended to preserve some spare wires at the cables used in marine engineering applications.

If the first cabling option was chosen during installation, it will be inevitable that the spare capacity will be less than 100% because if the wires of a single cable (dedicated to only one parameter) of only a single pair suffered any failure, the only solution will be the replacement of the whole cable. However, if the single cable (dedicated to only one parameter) had two pairs of wires, the spare pair of wires can be used instead of replacing the whole cable.

In the case of adopting the second cabling option of using a single instrumentation cable that will include the eight measured quantities, there will be more flexibility to control the capacity of the spare added pairs. The selected 50% spare capacity was assumed here as a moderate alternative to calculate the cabling cost, taking a moderate risk of a maximum pair failure probability of 50% of the used pairs (4 pairs out of 8 used pairs). Therefore, a 12-pairs cable can be used according to such an option.

The overall cost of cabling in any measurement/control system can be divided into five subsidiary costs:

1. Cost of the cables.
2. Cost of the cable containers or trays.
3. Cost of the cable bonds.
4. Cost of installation process or manpower.

As the first cost of cables is the higher cost among the previously mentioned four subsidiary costs, this article will concentrate only on the cables’ cost in order to demonstrate the economic efficiency of the developed wireless system through a comparison between the cabling cost and Wi-Fi modules cost as mediums of data transaction for the same task. In Table 2 and Table 3, there is an illustration of some randomly picked up cables that could have been used to construct the same system without using wireless technology based on thefirst cabling option (using a single cable for each parameter) and second cabling option (using a single cable for the eight measured parameters), respectively.

In order to implement the same system by using cabling for data transaction, the required cables’ lengths for cranes No.1 and No.2 will be assumed to be almost 120 m and 60 m, respectively, from the locations of the cranes to the host controller at the bridge. Therefore, the overall required cable length will be 180 m. After adding 20 m more as an approximate 10% of the overall calculated length for compensating any possible shortage during installation, the final overall cable length will be almost 200 m.

**Figure 6:**
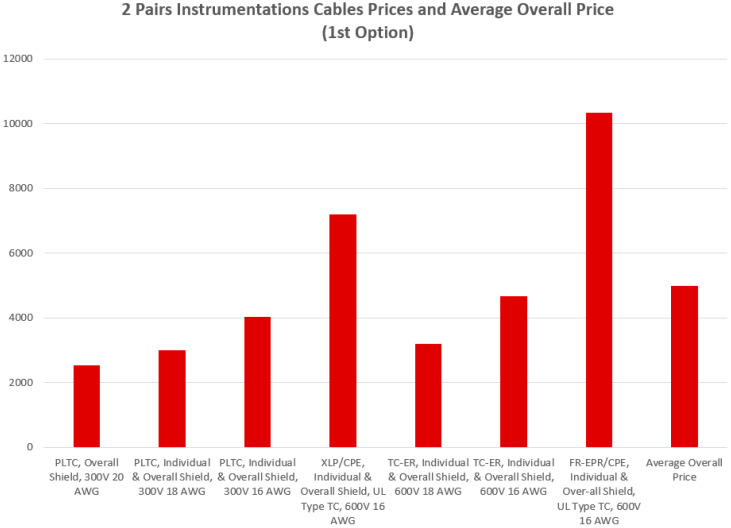
Illustration of the prices of the 7 types of the two pairs instrumentation cables indicated in Table 2 with an average overall price of almost USD 4933.

**Figure 7:**
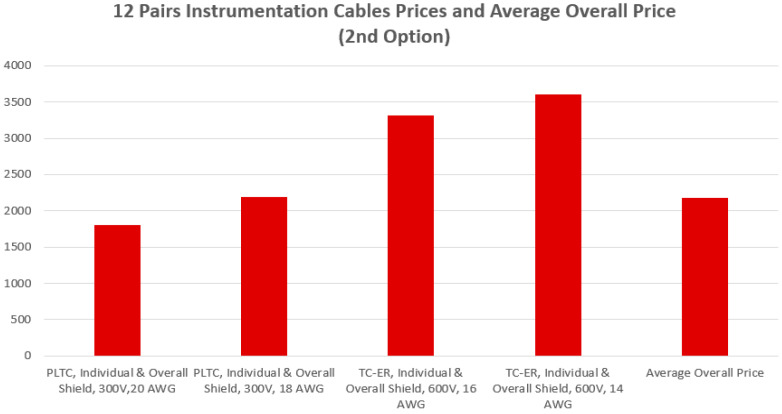
Illustration of the prices of the 4 types of the twelve pairs instrumentation cables indicated in Table 3 with an average overall price of almost USD 2181.86.

Figure 6 and Figure 7 demonstrate a comparative statistical analysis for the prices of the instrumentation cables required to implement both of the first and second cabling options, respectively. The average overall cost required to implement the first cabling option is almost USD 4933. This average cost was calculated for seven types of 2 pairs of wires instrumentation cables fabricated from different materials with AWG (American Wire Gauge) values of 16, 18 and 20. The average overall cost required to implement the second cabling option is almost USD 2181.86. This average cost was calculated for four types of 12 pairs of wires instrumentation cables fabricated from different materials with AWG values of 14, 16, 18 and 20.

It can be easily noticed that the average required price to implement the second cabling option with 50% spare capacity is almost half the price required to implement the first cabling option with 100% spare capacity; that is why the second cabling option is the most popular option on commercial ships for ships’ owners and ships’ builders in order to achieve maximum possible financial gain for the ships’ builders as well as the maximum possible cost saving for the ships’ owners. The critical disadvantage of the second cabling option is that it is sometimes rather difficult to implement, as some maritime engineering measurement/control systems are based on using sensors which are remotely separated by considerably long distances, which impedes the possibility of transmitting the collected signals from multiple sensors through only one cable (second cabling option), which means that the only remaining choice is to designate a cable for each sensor (first cabling option). That is why shipyards tend to adopt the first cabling option in such a case, however, they usually adopt it without the choice of the 100% spare capacity feature, they simply adopt it with 0% spare capacity (Only 1 pair, two wires for each signal). In systems subjected to high levels of humidity and corrosion, this leads to shortened lifetimes for the cables and consequently, totally inaccurate readings.

In order to demonstrate the cost efficiency of the developed wireless system from a perspective related to the comparative analysis of the selected cabling options (first and second cabling options), the total cost required to implement the Wi-Fi wireless data transaction medium should be calculated. The developed wireless system is based on using five ESP32 modules. The cost of each of these modules is almost USD 10 at the local market, which means that the overall cost to implement the Wi-Fi wireless data transaction medium is USD 50. Accordingly, the cost saving efficiency can be calculated as follows for both the first and second cabling options.

Cost saving efficiency = [1 − (Wi-Fi implementation cost/Average Cabling Cost)] × 100

Cost saving efficiency (1st cabling option) = [1 − (50/4933)] × 100 = 98.986%

Cost saving efficiency (2nd cabling option) = [1 − (50/2181.86)] × 100 = 97.7%

### 5.2. Implementation of the Principle of Functional Safety

The principle of functional safety is basically based on pairing the components included in any measurement system so that any component can be taken over by its functional pair in case of failure. For example, in a simple 4–20 mA measurement current loop, in order to implement the principle of functional safety partially from a perspective linked to the multichannel concept [19], the developed wireless proposed system will adopt an additional wireless channel for measurement data transaction, which will function as a backup channel for the cabling channel at the original system based on classical control/measurement techniques. This redundant decomposition [17,18] for the data transaction medium is a basic requirement for the application of the functional safety principle. According to the previous cost analysis, if such a redundant decomposition for the data transaction medium was carried out using two paired channels of cabling, the cabling average cost would have been (2 × USD 4933 = USD 9866) for the first cabling option and (2 × USD 2181.86 = USD 4363.72) for the second cabling option. However, if the redundant decomposition concept was applied through one cabling channel and one Wi-Fi wireless channel, the cost would have been reduced to (USD 50 + USD 4933 = USD 4983) for the first cabling option and (USD 50 + USD 2181.86 = USD 2231.86) for the second cabling option.

**Figure 8:**
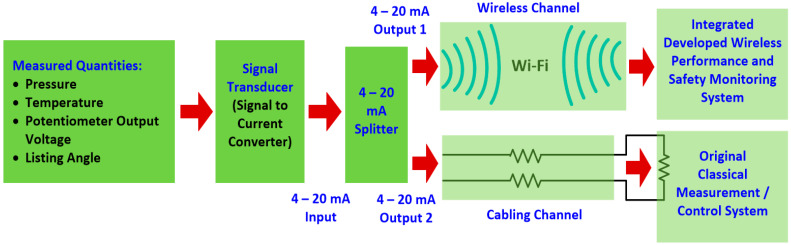
Illustration of the partial implementation of the functional safety principle through the redundant decomposition of the channel through which measurement/control data are exchanged into two channels. The first channel is Wi-Fi wireless based, while the second is based on conventional cabling (multichannel architecture).

In order to render further reduction for the cost of the developed proposed system, it was assumed that some signal conditioning units (splitters) would be used to obtain two measured outputs signals from the same sensor dedicated to a specific measured parameter. One signal is dedicated to the crane conventional control/measurement system and the other signal is dedicated to the developed wireless performance and safety monitoring system (Figure 8).

### 5.3. Implementation of the Principle of Predictive Maintenance (PdM)

The predictive maintenance principle was implemented by the developed Wi-Fi based wireless performance and safety monitoring system through providing a means of early detection for future failures through a performance monitoring log illustrating the changes in some critically important parameters over an unlimited period of time during cargo crane operation. Using additional software tools, charts can be built for the collected stored data in the performance monitoring log. Based on these charts, maintenance plans can be modified to eliminate minor uncritical failures before they become major and critical. This will consequently result in less downtime during crane failures. With such an effective maintenance plan based on reliable measurement data stored in the performance monitoring log, the lifetime of the marine cargo crane critical equipment will be extended which will lead to less need for repetitive replacement of spare parts. The lower the demand for spare parts, the greater the economic efficiency of the ship’s cargo crane, and also the higher the economical profit achieved by the ship owner.

**Figure 9:**
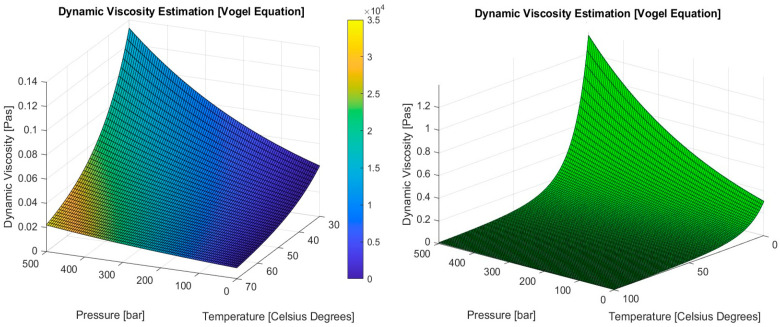
Example for hydraulic oil dynamic viscosity estimation charts that can be provided by the hydraulic oil supplier or the cargo crane manufacturer. Dynamic viscosity (μ) is measured in (Pas). Pressure is measured (p) in (bar). Temperature (T) is measured in °C based on [15].

Among the monitored parameters by the developed system, two parameters are considered the most critical. These two parameters are the hydraulic oil feed pressure and temperature. The importance of both parameters emerges from the fact that the efficiency of the overall hydraulic system is entirely dependent on proper in-range values of hydraulic oil feed pressure and temperature. The Vogel equation was used to calculate the dynamic viscosity of the hydraulic oil for specific values of pressure and temperature. As a detailed application for PdM, the following mathematical model was developed to analyze the changes in dynamic viscosity of the hydraulic oil during cargo crane operation based on the differences between calculated dynamic viscosity values at specific working hours and the dynamic viscosity values obtained by the hydraulic oil original characteristics charts constructed by the oil supplier or the cargo crane manufacturer (Figure 9). These differences might be a reflection of possible hydraulic equipment failure or an indication of a serious deterioration of the hydraulic oil condition and a change in its properties.

For specific pressure and temperature values, the dynamic viscosity of the hydraulic oil can be calculated using the Vogel equation (1), where (a1), (a2), (a), (b) and (c) are constants. The values of these constants are dependent on the nature of the analyzed hydraulic oil. The developed wireless system will monitor the temperature as well as the feed pressure values for the hydraulic oil during cargo crane operation. The monitored values will be stored in the performance log. Based on the preset scanning interval of the system, the average values for both feed pressure and temperature of the hydraulic oil will be calculated for each working hour. Each maintenance schedule is basically constructed to conduct some failure-preventive actions after a specific number of elapsed working hours (n). Therefore, the average values of temperature and feed pressure during each working hour will be used to calculate the dynamic viscosity for the hydraulic oil for each working hour. The obtained dynamic viscosity calculated using the averaged feed pressure and temperature values for each working hour will be compared with the dynamic viscosity value obtained from the charts provided by the crane manufacturer or the hydraulic oil supplier for the same averaged values of hydraulic oil feed pressure and temperature. The difference between both values of dynamic viscosity will be calculated for each working hour. The collected averaged values of hydraulic oil feed pressure for (n) working hours will be stored at the vector p(n). The collected averaged values of hydraulic oil temperature for (n) working hours will be stored at the vector T(n). Calculated values of hydraulic oil dynamic viscosity using corresponding pressure and temperature values at p(n) and T(n) for (n) working hours will be stored at the ${µ}_{c}(n)$. Obtained values for hydraulic oil dynamic viscosity through using the charts rendered by the hydraulic oil supplier or the crane manufacturer for corresponding feed pressure and temperature values at p(n) and T(n) for (n) working hours will be stored at the vector ${µ}_{r}(n)$. $∆\mu \left(n\right)$ represents the deviation values between corresponding values of calculated hydraulic oil dynamic viscosity values at ${µ}_{c}(n)$ and reference chart hydraulic oil dynamic viscosity values at ${µ}_{r}(n)$. $∆\mu_{CRITICAL}$ represents the critical deviation value between the dynamic viscosity value obtained through calculation and the dynamic viscosity value obtained from the supplier or manufacturer charts. $A\left(n_{r}\right)$ is a reduced vector for working hours at which $∆\mu \left(n\right)$ is greater than $∆\mu_{CRITICAL}$ where ${(n}_{r})$ is less than (n). In other words, $A\left(n_{r}\right)$ will indicate the working hours at which $∆\mu \left(n\right)$ has exceeded the critical deviation value $∆\mu_{CRITICAL}$, which will provide a timestamp for any alarming change in hydraulic oil dynamic viscosity. Matrix B will be constructed to provide a pattern for the repetition of $∆\mu \left(n\right)$ values which exceed $∆\mu_{CRITICAL}$, as each element of the matrix $A_{w,y}$ will represent the difference between $A_{nr}(w)$ and $A_{nr}(y)$ where the deviation between the calculated dynamic viscosity value and reference value from the supplier charts is greater than $∆\mu_{CRITICAL}$ for working hours w and y. In order to reduce the size of the data processed by the mathematical model, square matrix B will be reduced to the C matrix where main diagonal elements as well as elements located above the main diagonal will be eliminated. Main diagonal elements of B will be eliminated as they will be equal to zero. Since elements located at the upper half of matrix B will have the same absolute values as those located at the lower half, they will also be eliminated. The size of matrix C will be $C_{nr/2\times nr-1}$ for $n_{r}$ even values, while it will be $C_{nr-1/2\times nr}$ for $n_{r}$ odd values. Each element of the C matrix $C_{u\times v}$ will be equal to a corresponding element $A_{w\times y}$ located at the lower half of the B matrix. The correspondence between both elements can be derived using Equations (22)–(28). The first diagonal lower than the main diagonal of the B matrix will be relocated to the first row of the C matrix and similarly, will be relocated the rest of the diagonals at the B matrix lower part.

Example 1 illustrates the case for the obtained 6 ($n_{r}$ is even) working hours when ∆μ was greater than $∆\mu_{CRITICAL}$, while Example 2 illustrates the case for the obtained 7 ($n_{r}$ is odd) working hours when ∆μ was greater than $∆\mu_{CRITICAL}$. The first row of the C matrix is the most important as it reflects the repetition pattern for the working hours at which ∆μ was greater than $∆\mu_{CRITICAL}$. In the first example, the first row of the C matrix was (1,1,1,2,3), which indicates that ∆μ was greater than $∆\mu_{CRITICAL}$ for 3 successive hours (1,1,1), then it returned to normality where ∆μ was less than $∆\mu_{CRITICAL}$ only for 1 working hour, then it jumped back again to be greater than $∆\mu_{CRITICAL}$ after 2 h (from the 5th to 7th working hours) since the last deviation (1,2), then it came back to normality for 2 h, then it jumped back to a level greater than $∆\mu_{CRITICAL}$ after 3 h (from the 7th to 10th working hours) from the last deviation (2,3).

In the second example, the first row of the C matrix contained (1,1,1,1,2,3), which indicates that ∆μ was greater than $∆\mu_{CRITICAL}$ for 4 successive hours (1,1,1,1), then it returned to normality where ∆μ was less than $∆\mu_{CRITICAL}$ only for 1 working hour, then it jumped back again to be greater than $∆\mu_{CRITICAL}$ after 2 h (from the 5th to 7th working hours) since the last deviation (1,2), then it came back to normality for 2 h, then it jumped back to a level greater than $∆\mu_{CRITICAL}$ after 3 (from the 7th to 10th working hours) hours from the last deviation (2,3).

In the first example, the number of elements included in matrices B and C are 36 and 15, respectively. Similarly, the number of elements included in matrices B and C are 49 and 21, respectively, in the second example. Accordingly, it can be easily noticed that adopting such a technique of forming reduced size matrix C to store the time span between the working hours at which ∆μ was greater than $∆\mu_{CRITICAL}$ will lead to constructing databases of small sizes than it would been if only the B matrix was used.

The maintenance engineer or electro-technical officer can make use of such a mathematical model by setting a maximum permissible value for the successive working hours at which ∆μ was greater than $∆\mu_{CRITICAL}$. If such a value was exceeded, it means that urgent inspection should be conducted on the crane hydraulic system at the first possible chance in order to avoid further problems.

(1) $$\mu \left(p,T\right)=ae^{\left[\frac{b}{\left(T+273.15\right)-c}\right]}e^{\left[\frac{p}{{a_{1}+a}_{2}T}\right]}$$

(2) $$p\left(n\right)=[p_{1},\;p_{2},p_{3},\;p_{4},\;p_{5},\;\ldots \ldots \ldots \ldots ,\;p_{n}]\;$$

(3) $$T\left(n\right)=[T_{1},\;T_{2},T_{3},\;T_{4},\;T_{5},\;\ldots \ldots \ldots \ldots ,\;T_{n}]$$

(4) $$\mu_{c}\left(n\right)=[{\mu_{c}}_{1},\;{\mu_{c}}_{2},{\mu_{c}}_{3},\;{\mu_{c}}_{4},\;{\mu_{c}}_{5},\;\ldots \ldots \ldots \ldots ,\;{\mu_{c}}_{n}]$$

(5) $$\mu_{r}\left(n\right)=[{\mu_{r}}_{1},\;{\mu_{r}}_{2},{\mu_{r}}_{3},\;{\mu_{r}}_{4},\;{\mu_{r}}_{5},\;\ldots \ldots \ldots \ldots ,\;{\mu_{r}}_{n}]$$

(6) $$∆\mu \left(j\right)=\left|\;\mu_{c}\left(j\right)-\mu_{r}\left(j\right)\;\right|\;where\;j=1:n$$

(7) $$A\left(n\right)=[∆\mu \left(1\right),\;∆\mu \left(2\right),∆\mu \left(3\right),\;∆\mu \left(4\right),\;∆\mu \left(5\right),\;\ldots \ldots \ldots \ldots ,\;∆\mu \left(n\right)]$$

(8) $$A\left(j\right)=\left\{\begin{array}{c} j\;if\;∆\mu \left(j\right)>∆\mu_{CRITICAL}\;where\;j=1:n\\ 0\;if\;∆\mu \left(j\right)<∆\mu_{CRITICAL}\;where\;j=1:n \end{array}\right.$$

(9) $$A\left(n_{r}\right)=[A_{1},\;A_{2},A_{3},\;A_{4},\;A_{5},\;\ldots \ldots \ldots \ldots ,\;A_{n_{r}}]\;B_{nr\times nr}=\left[\begin{array}{cccccccc} A_{1,1} & A_{1,2} & A_{1,3} & A_{1,4} & A_{1,5} & \ldots \ldots \ldots & \ldots \ldots \ldots & A_{1,nr}\\ A_{2,1} & A_{2,2} & A_{2,3} & A_{2,4} & A_{2,5} & \ldots \ldots \ldots & \ldots \ldots \ldots & A_{2,nr}\\ A_{3,1} & A_{3,2} & A_{3,3} & A_{3,4} & A_{3,5} & \ldots \ldots \ldots & \ldots \ldots \ldots & A_{3,nr}\\ A_{4,1} & A_{4,2} & A_{4,3} & A_{4,4} & A_{4,5} & \ldots \ldots \ldots & \ldots \ldots \ldots & A_{4,nr}\\ A_{5,1} & A_{5,2} & A_{5,3} & A_{5,4} & A_{5,5} & \ldots \ldots \ldots & \ldots \ldots \ldots & A_{5,nr}\\ \vdots & \vdots & \vdots & \vdots & \vdots & \vdots & \vdots & \vdots \\ \vdots & \vdots & \vdots & \vdots & \vdots & \vdots & \vdots & \vdots \\ A_{nr,1} & A_{nr,2} & A_{nr,3} & A_{nr,4} & A_{nr,5} & \ldots & A_{nr,nr-1} & A_{nr,nr} \end{array}\right]$$

(10) $$B_{nr\times nr}=\left[\begin{array}{cccccccc} 0 & 0 & 0 & 0 & 0 & \ldots \ldots \ldots & \ldots \ldots \ldots & 0\\ A_{2,1} & 0 & 0 & 0 & 0 & \ldots \ldots \ldots & \ldots \ldots \ldots & 0\\ A_{3,1} & A_{3,2} & 0 & 0 & 0 & \ldots \ldots \ldots & \ldots \ldots \ldots & 0\\ A_{4,1} & A_{4,2} & A_{4,3} & 0 & 0 & \ldots \ldots \ldots & \ldots \ldots \ldots & 0\\ A_{5,1} & A_{5,2} & A_{5,3} & A_{5,4} & 0 & \ldots \ldots \ldots & \ldots \ldots \ldots & 0\\ \vdots & \vdots & \vdots & \vdots & \vdots & \ddots & \vdots & \vdots \\ \vdots & \vdots & \vdots & \vdots & \vdots & \vdots & \ddots & \vdots \\ A_{nr,1} & A_{nr,2} & A_{nr,3} & A_{nr,4} & A_{nr,5} & \ldots & A_{nr,nr-1} & 0 \end{array}\right]$$

(11) $$d_{1}=[A_{2,1},\;A_{3,2},A_{4,3},\;A_{5,4},\;A_{6,5},\;\ldots \ldots \ldots \ldots ,\;A_{nr,nr-1}],\;h_{1}=nr-1$$

(12) $$d_{2}=[A_{3,1},\;A_{4,2},A_{5,3},\;A_{6,4},\;A_{7,5},\;\ldots \ldots \ldots \ldots ,\;A_{nr,nr-2}],h_{2}=nr-2$$

(13) $$d_{3}=\left\{A_{4,1},\;A_{5,2},A_{6,3},\;A_{7,4},\;A_{8,5},\;\ldots \ldots \ldots \ldots ,\;A_{nr,nr-3}\right\},h_{3}=nr-3$$

(14) $$d_{4}=[A_{5,1},\;A_{6,2},A_{7,3},\;A_{8,4},\;A_{9,5},\;\ldots \ldots \ldots \ldots ,\;A_{nr,nr-4}],h_{3}=nr-4$$

(15) $$d_{5}=[A_{6,1},\;A_{7,2},A_{8,3},\;A_{9,4},\;A_{10,5},\;\ldots \ldots \ldots \ldots ,\;A_{nr,nr-5}],h_{3}=nr-5$$

(16) $$d_{nr-2}=\left\{A_{nr-1,1},\;A_{nr,2}\right\},h_{nr-2}=2$$

(17) $$d_{x}=\left\{A_{nr,1}\right\},h_{x}=1\;where\;x=nr-1$$

(18) $$\frac{C_{nr/2\times nr-1}}{nr\;even}=\left[\begin{array}{cccccccc} C_{1,1} & C_{1,2} & C_{1,3} & C_{1,4} & C_{1,5} & \ldots \ldots \ldots & \ldots \ldots \ldots & C_{1,nr-1}\\ C_{2,1} & C_{2,2} & C_{2,3} & C_{2,4} & C_{2,5} & \ldots \ldots \ldots & \ldots \ldots \ldots & C_{2,nr-1}\\ C_{3,1} & C_{3,2} & C_{3,3} & C_{3,4} & C_{3,5} & \ldots \ldots \ldots & \ldots \ldots \ldots & C_{3,nr-1}\\ \vdots & \vdots & \vdots & \vdots & \vdots & \vdots & \vdots & \vdots \\ C_{\frac{nr}{2},1} & C_{\frac{nr}{2},2} & C_{\frac{nr}{2},3} & C_{\frac{nr}{2},4} & C_{\frac{nr}{2},5} & \ldots & C_{\frac{nr}{2},nr-2} & C_{\frac{nr}{2},nr-1} \end{array}\right]$$

(19) $$\frac{C_{nr/2\times nr-1}}{nr\;even}=\left[\begin{array}{cccccccccc} A_{2,1} & A_{3,2} & A_{4,3} & A_{5,4} & A_{6,5} & \ldots \ldots \ldots & \ldots \ldots \ldots & \ldots \ldots \ldots & \ldots \ldots \ldots & A_{nr,nr-1}\\ A_{3,1} & A_{4,2} & A_{5,3} & A_{6,4} & A_{7,5} & \ldots \ldots \ldots & \ldots \ldots \ldots & \ldots \ldots \ldots & A_{nr,nr-2} & A_{4,1}\\ A_{5,2} & A_{6,3} & A_{7,4} & A_{8,5} & A_{9,6} & \ldots \ldots \ldots & A_{nr,nr-3} & A_{5,1} & A_{6,2} & A_{7,3}\\ \vdots & \vdots & \vdots & \vdots & \vdots & \vdots & \vdots & \vdots & \vdots & \vdots \\ \ldots \ldots \ldots & \ldots \ldots \ldots & \ldots \ldots \ldots & \ldots \ldots \ldots & \ldots \ldots \ldots & A_{nr-1,2} & A_{nr,3} & A_{nr-1,1} & A_{nr,2} & A_{nr,1} \end{array}\right]$$

(20) $$\frac{C_{nr-1/2\times nr}}{nr\;odd}=\left[\begin{array}{cccccccc} C_{1,1} & C_{1,2} & C_{1,3} & C_{1,4} & C_{1,5} & \ldots \ldots \ldots & \ldots \ldots \ldots & C_{1,nr}\\ C_{2,1} & C_{2,2} & C_{2,3} & C_{2,4} & C_{2,5} & \ldots \ldots \ldots & \ldots \ldots \ldots & C_{2,nr}\\ C_{3,1} & C_{3,2} & C_{3,3} & C_{3,4} & C_{3,5} & \ldots \ldots \ldots & \ldots \ldots \ldots & C_{3,nr}\\ \vdots & \vdots & \vdots & \vdots & \vdots & \vdots & \vdots & \vdots \\ C_{\frac{nr-1}{2},1} & C_{\frac{nr-1}{2},2} & C_{\frac{nr-1}{2},3} & C_{\frac{nr-1}{2},4} & C_{\frac{nr-1}{2},5} & \ldots & C_{\frac{nr-1}{2},nr-1} & C_{\frac{nr-1}{2},nr} \end{array}\right]$$

(21) $$\frac{C_{nr-1/2\times nr}}{nr\;odd}=\left[\begin{array}{cccccccccc} A_{2,1} & A_{3,2} & A_{4,3} & A_{5,4} & A_{6,5} & \ldots \ldots \ldots & \ldots \ldots \ldots & \ldots \ldots \ldots & A_{nr,nr-1} & A_{3,1}\\ A_{4,2} & A_{5,3} & A_{6,4} & A_{7,5} & A_{8,6} & \ldots \ldots \ldots & A_{nr,nr-2} & A_{4,1} & A_{5,2} & A_{6,3}\\ A_{7,4} & A_{6,3} & \ldots \ldots \ldots & A_{nr,nr-3} & A_{5,1} & A_{6,2} & A_{7,3} & A_{8,4} & A_{9,5} & A_{10,6}\\ \vdots & \vdots & \vdots & \vdots & \vdots & \vdots & \vdots & \vdots & \vdots & \vdots \\ \ldots \ldots \ldots & \ldots \ldots \ldots & \ldots \ldots \ldots & \ldots \ldots \ldots & \ldots \ldots \ldots & A_{nr-1,2} & A_{nr,3} & A_{nr-1,1} & A_{nr,2} & A_{nr,1} \end{array}\right]$$

$$C_{u,v}=A_{w,y}\;where\;u=1:k,\;v=1:l$$

(22) $$S=\left(u-1\right)l+v$$

(23) ∑x=1 x=g−1hx<S ≤ ∑x=1 x=ghxwhere g ϵ dg

(24) $$h_{g}=nr-g$$

(25) $$h_{x}=1\;where\;x=nr-1$$

(26) f=∑x=1 x=ghx−S

(27) $$y=h_{g}-f$$

(28) w=y+g

Example 1:

$$A\left(6\right)=\left\{2,3,4,5,7,10\right\}\;where\;nr=6$$

$$B_{6\times 6}=\left[\begin{array}{cccccc} 0 & 0 & 0 & 0 & 0 & 0\\ 1 & 0 & 0 & 0 & 0 & 0\\ 2 & 1 & 0 & 0 & 0 & 0\\ 3 & 2 & 1 & 0 & 0 & 0\\ 5 & 4 & 3 & 2 & 0 & 0\\ 8 & 7 & 6 & 5 & 3 & 0 \end{array}\right]$$

$$C_{3\times 5}=\left[\begin{array}{ccccc} {C_{1,1}=A}_{2,1}=1 & {C_{1,2}=A}_{3,2}=1 & {C_{1,3}=A}_{4,3}=1 & {C_{1,4}=A}_{5,4}=2 & {C_{1,5}=A}_{6,5}=3\\ {C_{2,1}=A}_{3,1}=2 & {C_{2,2}=A}_{4,2}=2 & {C_{2,3}=A}_{5,3}=3 & {C_{2,4}=A}_{6,4}=5 & {C_{2,5}=A}_{4,1}=3\\ {C_{3,1}=A}_{5,2}=4 & {C_{3,2}=A}_{6,3}=6 & {C_{3,3}=A}_{5,1}=5 & {C_{3,4}=A}_{6,2}=7 & {C_{3,5}=A}_{6,1}=8 \end{array}\right]$$

$$C_{3\times 5}=\left[\begin{array}{ccccc} 1 & 1 & 1 & 2 & 3\\ 2 & 2 & 3 & 5 & 3\\ 4 & 6 & 5 & 7 & 8 \end{array}\right]$$

Example 2:

$$A\left(7\right)=\left\{1,2,\;3,4,\;5,\;7,10\right\}\;where\;nr=7$$

$$B_{7\times 7}=\left[\begin{array}{ccccccc} 0 & 0 & 0 & 0 & 0 & 0 & 0\\ 1 & 0 & 0 & 0 & 0 & 0 & 0\\ 2 & 1 & 0 & 0 & 0 & 0 & 0\\ 3 & 2 & 1 & 0 & 0 & 0 & 0\\ 4 & 3 & 2 & 1 & 0 & 0 & 0\\ 6 & 5 & 4 & 3 & 2 & 0 & 0\\ 9 & 8 & 7 & 6 & 5 & 3 & 0 \end{array}\right]$$

$$C_{3\times 7}=\left[\begin{array}{ccccccc} {C_{1,1}=A}_{2,1}=1 & {C_{1,2}=A}_{3,2}=1 & {C_{1,3}=A}_{4,3}=1 & {C_{1,4}=A}_{5,4}=1 & {C_{1,5}=A}_{6,5}=2 & {C_{1,6}=A}_{6,5}=3 & {C_{1,7}=A}_{3,1}=2\\ {C_{2,1}=A}_{4,2}=2 & {C_{2,2}=A}_{5,3}=2 & {C_{2,3}=A}_{6,4}=3 & {C_{2,4}=A}_{7,5}=5 & {C_{2,5}=A}_{4,1}=3 & {C_{2,6}=A}_{5,2}=3 & {C_{2,7}=A}_{6,3}=4\\ {C_{3,1}=A}_{7,4}=6 & {C_{3,2}=A}_{5,1}=4 & {C_{3,3}=A}_{6,2}=5 & {C_{3,4}=A}_{7,3}=7 & {C_{3,5}=A}_{6,1}=6 & {C_{3,6}=A}_{7,2}=8 & {C_{3,7}=A}_{7,1}=9 \end{array}\right]$$

$$C_{3\times 7}=\left[\begin{array}{ccccccc} 1 & 1 & 1 & 1 & 2 & 3 & 2\\ 2 & 2 & 3 & 5 & 3 & 3 & 4\\ 6 & 4 & 5 & 7 & 6 & 8 & 9 \end{array}\right]$$

### 5.4. Developed System Analysis in Conjunction with Selected Previous Literature

The purpose of this section is to provide a brief description of the conclusions obtained by other researchers in the previous literature from a perspective related to the use of various wireless technologies in maritime engineering applications, particularly those based on Wi-Fi and IoT. This description will be presented through a comparative analysis differentiating between the contributions of those authors and the elements of novelty depicted in this article.

In [1], the authors shed light on some of the requirements that should be taken into account while considering the use of IoT in marine environment monitoring and protection systems. Naturally, these requirements will be applicable (to some extent) for measurement and control systems on commercial ships as well. Such requirements, as well as the importance of their implementation at the proposed developed wireless safety and performance system for the cargo cranes, will be briefly discussed as follows:

#### 5.4.1. High Water Resistance and Strong Robustness

**Figure 10:**
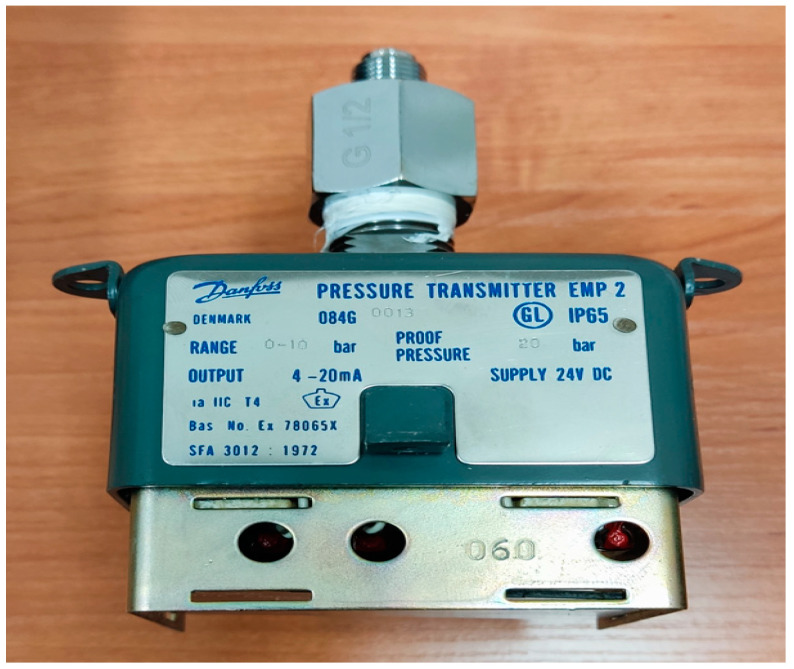
Example of pressure switch with ingress protection rating (IP65). Integer “6” refers to solid ingress level. (No ingress of dust; complete protection against contact dust-tight. A vacuum must be applied. Test duration of up to 8 h based on airflow.) Integer “5” refers to liquid ingress level. (Protection against water jets, water projected by a nozzle (6.3 mm (0.25 in)) against enclosure from any direction shall have no harmful effects).

The output signals from the used sensors in the crane measurement and control system are either 4–20 mA transmitters (Pressure, Temperature and Listing angle) or ON–OFF switches with NO/NC contacts (hydraulic oil level switch, hydraulic brake engaged/disengaged and fire alarm). Both of these types are usually designed and produced to be water-resistant sensors to inhibit the possibility of water penetration to the electronic circuits inside the sensor. Ingress protection (IP) rating [2] on any sensor defines the degree to which the housing of the sensor will be capable of preventing any probable ingression by either solids or liquids. Additionally, the IP rating can optionally provide the maximum pressure level that the sensor housing can withstand. Figure 10 demonstrates an example of the indicated IP65 rating on a pressure transmitter. The integer 6 refers to the level of solid ingress protection rating, while the integer 5 refers to the liquid ingress protection rating. In addition to the water resistivity of the used sensors by the developed wireless system, the ESP32 modules included in the system should be fitted in housings with high degrees of IP rating, especially as all the ESP32 modules at the developed system will be mounted outside the crane funnel in the open air (unlike the sensors that will be mounted inside the crane funnel), which means that the wireless modules will be more vulnerable to the negative effects that might be induced by harsh weather conditions such as strong wind and heavy rain.

#### 5.4.2. Low Energy Consumption

The backbone of the developed cargo crane wireless safety and performance monitoring system is the utilization of ESP32 modules as wireless data transponders interfacing with multiple sensors and also as wireless switches compensating possible reduced RSSI levels at specific locations during cargo operation. The average power consumption levels of an ESP32-S3-WROOM1 module in deep sleep mode and active mode are 25.85 µW and 78.32 mW, respectively, while the current consumption levels in both modes are 81.4 µA and 23.88 mA, respectively [3]. Therefore, the reliance of the developed wireless system in this article on the use of ESP32 modules has fulfilled the requirement of maintaining low energy consumption levels in a Wi-Fi based measurement process.

#### 5.4.3. Stability of Radio Signal

In the developed wireless performance and safety monitoring system discussed in this article, stability of radio signal was achieved through the ESP32 modules No. 3, No.4 and No.5, as they functioned as switches receiving the transmitted data by the two modules located at the top of the cranes. The three modules located on the bridge side carried out their communication tasks simultaneously as well as alternatively. The simultaneous functionality was performed by all modules on the bridge side in case of the absence of any possible obstacles that could disrupt the RF waves propagation between those modules located at the top of the cranes and those located on the bridge side. The alternative functionality of the ESP32 modules on the bridge side took place in case of an operational obstacle attenuating or blocking the propagation of the RF waves between the ESP32 modules on the tops of the cranes and one or two modules located on the bridge side. If the crane jib was moving with a very sharp angle, this might be considered as an operational obstacle if the jib would have attenuated or blocked the RF waves propagation between the ESP32 modules on the top of the crane and one of the modules located on one of the bridge wings, for instance. In such a case, the ESP32 module at the bridge other wing would compensate for the absence of the blocked module by carrying out its communication tasks with the ESP32 modules on the top of the crane. Similarly, the container units loaded at very high elevation level might be considered an operational obstacle as they can attenuate or block the propagation of the RF waves during crane operation, particularly during two specific periods of time. The first period is at the beginning of the discharging operation, while the second period is at the end of the loading operation. At these two periods of time, the handled containers by the crane are probably located at a high elevation level; that is why the ESP32 modules on the bridge side function alternatively to compensate for any possible communication failure with any of them.

#### 5.4.4. Reliability

The developed wireless performance and safety monitoring system was mainly based on a laboratory stand that was elaborately described in [7]. Communication tasks at this laboratory stand are carried out authentically, as the ESP32 modules on the bridge side are continuously sending authenticating messages confirming reception of the measurement data to the ESP32 modules located on the top of the cranes. Such an authentication technique increases the reliability level of the system from a perspective linked to the aspect of confirming the delivery of the measurement data from the sensors’ station to the host controller.

**Figure 11:**
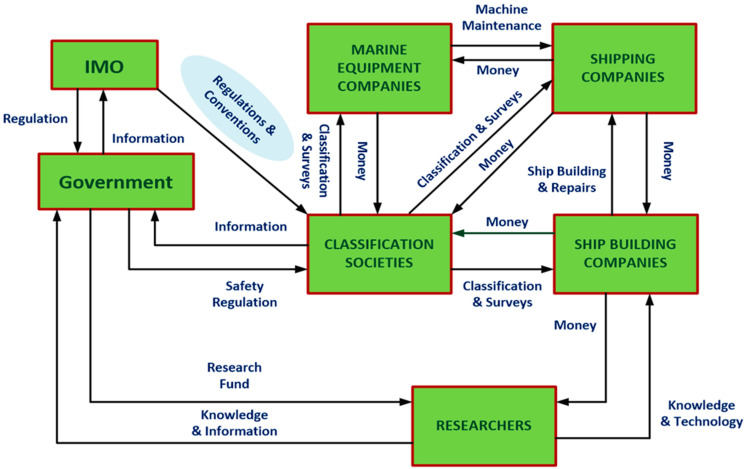
Illustration for the decomposition of shipping services to several types of governmental organizations based on [4].

In [4], the article rendered a logical decomposition for the shipping service from a point of view related to the possibilities of deployment of IoT in maritime engineering (Figure 11). This decomposition has highlighted the relation between three important types of organizations.

1. Educational organizations (maritime universities and institutes);
2. Legal organizations (the International Maritime Organization, IMO, and local governmental maritime authorities);
3. Marine equipment production organizations (classification societies, marine equipment companies and shipbuilding companies).

The importance of these three types of organizations lies in the necessity of their consent or their support in order for any new scientific/technical proposed solution or system to be approved and legally utilized. If the developed wireless performance and safety monitoring system discussed in this article was considered an example of the application of such a concept, it suffices to mention that the developed system is a part of a doctoral study in a maritime university funding the process of conducting as well as advertising the research through a publication discussing the main features of the system in a scientific journal. On the other hand, the developed wireless system was implemented on a ship owned by an international shipping company through its existence in dry dock. According to such a concept, it would also be valuable to raise attention to the significant role played by classification societies, as their approval is an important requirement for the developed system to be in service.

Moreover, the authors in [4] have categorized the most important marine engineering applications at which IoT can be adopted in the following:

1. Marine equipment monitoring.
2. Marine equipment remote maintenance.
3. Hull load control systems.
4. Advanced weather routing.
5. Cargo handling equipment monitoring and automation.
6. Streamlining port operation.
7. IoT utilizing for ship design.

The developed wireless safety and performance monitoring system can be considered an example for the first as well as the second items among the previously enumerated applications as it carries out the function of monitoring the major critical parameters of the cargo crane control system and also renders the required database to conduct remote maintenance through the collected information in the performance log.

In [5], the article enlisted some of the used wireless shipboard machinery monitoring systems. The enlisted systems can be considered general use monitoring systems of a relatively wide (not specific) perspective. Examples of the wirelessly monitored parameters in these systems are as follows:

1. Vibration in the ship’s hull.
2. Fire detection system.
3. Main engine bearing temperature.
4. Generator rotor temperature.
5. Generator excitation voltage/current.
6. Machinery vibration in engine room.
7. Fluid levels in holding tanks.

More specific and critical parameters can be added to the enlisted monitored variables in each of the shipboard equipment. For the main engine and diesel generators, exhaust temperature, jacket cooling freshwater temperature and cylinder lubrication oil temperature at each of the engine units are very critical parameters that can be monitored wirelessly. For holding tanks, not only fluid level (through pressure transmitters) can be wirelessly monitored, but also fluid temperatures, particularly in fuel oil or diesel oil tanks such as storage tanks, settling tanks and service tanks. Additionally, more attention should be paid to cargo handling machinery such as cargo cranes. (The developed wireless cargo crane performance and safety monitoring system in this article has achieved such a goal.) Similarly, maneuvering propelling equipment, such as bow thrusters, can also be wirelessly monitored.

An important aspect should be taken into account during planning for specific shipboard equipment to be monitored wirelessly. This aspect is the specific period of time at which such a system will be functional. Shipboard measurement and control systems can be classified into three categories according to such a point of view:

1. Systems which are active only during sailing (main engine and bow thruster during maneuvering).
2. Systems which are active only during ship’s existence in port (cargo cranes and hatch covers hydraulic units).
3. Systems which are continuously active regardless of ship’s location (diesel generators, firefighting and detection systems, ballast water treatment system, tank level measurement system and others).

Generally, overall sailing periods are longer than periods of a ship’s existence in ports, except for some rare cases, particularly for smaller size ships. Therefore, shipboard systems which are only active during ship’s existence in ports have less working hours than shipboard systems which are only active during sailing. Accordingly, it is much more easier to implement a wireless monitoring process for such systems from a perspective related to the expected lifetime of the used equipment, power consumption and longer periods of inactivity that can be dedicated to periodic maintenance or upgrading. On the other hand, it should be taken into account that systems which are continuously active during sailing and in port are the most critical shipboard systems, especially diesel generators and electrical power generation units. Consequently, implementation of wireless monitoring process for such systems mandates more careful selective requirements during the planning phase of implementing such a process. Robustness, rigid structure and being less affected by vibration are examples of such selective requirements, especially when choosing the wireless modules based on whichthe wireless monitoring process will be built.

Through experimental research conducted in [6], the authors reached a certain conclusion that path loss of RF wave propagation increases at higher vibration levels. The research was carried out on different types of antennas at RF frequencies of 2.7 GHz and 4.8 GHz. These RF frequencies were located in a very close proximity to the ISM band Wi-Fi frequencies of 2.4 GHz and 5 GHz; that is why wireless measurement systems based on using Wi-Fi modules might suffer a similar effect at higher vibration levels. Due to such a drawback, the authors had the following recommendations to avoid the negative influence on RF waves propagation:

1. Avoiding antennas installations at positions with accelerated vibration.
2. Utilizing directional or beamforming antennas and avoiding omnidirectional antennas.
3. At high vibration levels, it would be favorable to adopt the 5 GHz ISM Wi-Fi frequency band.

Accordingly, it would be valuable to consider embracing such recommendations in order to avoid reduced path loss levels if Wi-Fi technology is utilized for measurement data transaction in maritime engineering applications, particularly in those systems which are continuously active regardless of the ship’s location and those which are active only during sailing, as both categories are subjected to higher vibration levels than systems which are only active during ship’s existence in port.

## 6. Conclusions

- The popularity of adopting wireless technology as a medium for measurement and control data transaction can be increased among ships’ owners and marine engineering companies if the wireless technology was used as an integrated element to upgrade conventional measurement and control systems in marine engineering applications.
- Wireless technology is a more cost effective option that can be adopted by marine engineers, rendering enhanced levels of operational safety for classical maritime measurement and control systems so that the negative influence of equipment aging (such as longer downtime and continuous need for the replacement of relatively expensive spare parts) can be reduced, eliminated or early-detected.
- The cost analysis conducted in this article has evidently verified the concept of wireless technology economic efficiency through a comparison between the required cost for two possible cabling options and the required cost for the ESP32 Wi-Fi modules as data transaction mediums in order to implement the proposed wireless performance and safety monitoring system. These two cabling options are:
  1- Eight instrumentation cables of 4 pairs and 100% spare capacity for each parameter measured by the system.
  2- A single instrumentation cable of 12 pairs and 50% spare capacity for all eight parameters measured by the system.
  The calculated cost efficiencies of both the first and second cabling options in comparison with the cost required for the ESP32 Wi-Fi modules are 98.986% and 97.7%, respectively.
- Wireless technology can be considered an economically efficient alternative to implement the principle of functional safety in marine engineering applications. If both cabling and RF waves propagation were used simultaneously as data transaction mediums, this can be considered a cheaper option to partially implement the principle of functional safety, rather than using redundantly paired cabling channels.
- The main goal of the predictive maintenance, or PdM, principle can be achieved with the least affordable cost through developing wireless integral systems dedicated to monitor specific critical parameters in conventional marine measurement/control systems. Performance logs and charts will be constructed for the collected stored data in order to create or modify periodic maintenance schedules for the monitored equipment.
- Based on the collected measurement data by the developed wireless performance and safety system for the cargo cranes, this article demonstrated the derivation of a mathematical model processing the stored hydraulic oil feed pressure and temperature readings stored in the system’s performance log for each working hour during operation. Using the measured feed pressure and temperature values, the mathematical model calculated the dynamic viscosity of the hydraulic oil at specific working hours and compared it with reference values for the dynamic viscosity of hydraulic oil obtained from the charts provided by the crane manufacturer or the oil supplier. According to the detected critical deviation between the calculated and referenced dynamic viscosity values at specific working hours, the maintenance engineer will have the ability to detect possible changes in hydraulic oil properties or predict a critical failure in hydraulic oil system before it takes place, which is the main purpose of applying PdM. The main advantage of such a model is the reduced size of the database required to identify the periods of time elapsing between the working hours at which the measured hydraulic oil dynamic viscosity critically deviates from its reference value for the same feed pressure and temperature readings.
- In light of the contributions achieved by other researchers in the recent literature related to the topic of wireless technology implementation in maritime engineering applications, this article has highlighted the importance of the following concepts from a point of view related to the evaluation of the efficiently developed wireless performance and safety monitoring system dedicated to cargo cranes:
  1- Reliability, robustness, RF waves stability of propagation, low power consumption and high resistivity to water/weather conditions are all critical requirements for any wireless measurement system. These requirements can be fulfilled at the developed wireless system if recommendations mentioned in discussion are taken into account.
  2- The developed wireless system in this article is a clear demonstration for different types of organizations (educational, governmental and international) in the shipping industry.
  3- The application of the developed wireless system in this article has reflected the less complicated implementation of marine measurement/control systems which are only active during ship’s existence in port, according to the upgraded classification of the maritime engineering applications based on the period of activity whether it was sailing or berthing.
  4- For maritime engineering applications which are mostly active during sailing, high vibration levels might lead to increased path loss values of RF waves propagation during wireless data transaction. This negative influence can be relatively reduced by recommendations suggested by other researchers [6] and mentioned in t Section 5.4 of the discussion.

## 7. Future Work

The developed wireless performance and safety monitoring system discussed in this article was successfully tested on a container ship during its quinquennial routine planned inspection and maintenance in the dry-dock. Consequently, some operational factors were unfortunately not taken into consideration while conducting such a test. Latency, packet loss and estimation of the effect of moving containers during cargo handling operations are examples of such factors. Therefore, there is a plan to conduct a detailed analysis evaluating the quality of the performance of the developed Wi-Fi based wireless monitoring system, where these factors will be taken into account.

## Figures and Tables

**Table 1:** Critical parameters of the cargo crane monitored by the developed Wi-Fi based wireless system. Default state refers to the parameter state when the crane is not in operational state. Operational state refers to the parameter state when the crane is handling cargo (loading/discharging) (Appendix A).

No.	Parameter	Default State	Operational State
1	Fire Alarm	Normal	Alarm
2	Brake Valve Status	De-energized Engaged Idle	Energized Disengaged In Operation
3	Hydraulic Oil Tank Level	Normal	Low (Alarm)
4	Operator Joystick Output Voltage	6 VDC (Neutral)	(0–6 VDC) (Lowering) (6–12 VDC) (Hoisting)
5	Hydraulic Oil Feed Pressure	0 bar (Idle)	(20–40 bar) (Normal) (<20 bar) (Alarm) (>40 bar) (Alarm)
6	Hydraulic Oil Temperature	Ambient Temp. (Idle)	(<60 °C) (Normal) (>60 °C) (Alarm)
7	Hoisting Load Pressure	0 bar (Idle)	(<200 bar) (High Speed) (>200 bar) (Low Speed)
8	Listing Angle	Ideal State (Should be 0°)	Operational State (<4° Normal) (>4° Alarm)

**Table 2:** Instrumentation cables that might be used in cabling process in case the first option was taken into account. More specific and technical information about the cables are available in Appendix B (from 1 to 7). These links refer to the website www.wireandcableyourway.com, where various types of cables by various manufacturers (Prysmian Draka, Lapp, West Penn Wire, Helukabel, Alpha Wire, Corning, Thomas & Betts, Hubbell, Leviton, Burndy, Pnduit and Commscope) can be purchased.

No.	Material, No. of Pairs, Shielding, Vmax	AWG/Wire Diameter mm	Price for 200 m
1	PLTC, Overall Shield, 300 V 2 Pairs	20/0.812	USD 2519.04
2	PLTC, Individual & Overall Shield, 300 V 2 Pairs	18/1.024	USD 2991.36
3	PLTC, Individual & Overall Shield, 300 V 2 Pairs	16/1.291	USD 4040.96
4	XLP/CPE, Individual & Overall Shield, UL Type TC, 600 V 2 Pairs	16/1.291	USD 7189.76
5	TC-ER, Individual & Overall Shield, 600 V 2 Pairs	18/1.024	USD 3201.28
6	TC-ER, Individual & Overall Shield, 600 V 2 Pairs	16/1.291	USD 4670.72
7	FR-EPR/CPE, Individual & Overall Shield, UL Type TC, 600 V 2 Pairs	16/1.291	USD 10,338.56

**Table 3:** Instrumentation cables that might be used in cabling process in case the second option was taken into account. More specific and technical information about the cables are available in Appendix B (from 8 to 11). These links refer to the website www.wireandcableyourway.com, where various types of cables by various manufacturers (Prysmian Draka, Lapp, West Penn Wire, Helukabel, Alpha Wire, Corning, Thomas & Betts, Hubbell, Leviton, Burndy, Pnduit and Commscope) can be purchased.

No.	Material, No. of Pairs, Shielding, Vmax	AWG/Wire Diameter mm	Price for 200 m
1	PLTC, Individual & Overall Shield, 300 V 12 Pairs	20/0.812	USD 1804
2	PLTC, Individual & Overall Shield, 300 V 12 Pairs	18/1.024	USD 2184.48
3	TC-ER, Individual & Overall Shield, 600 V 12 Pairs	16/1.291	USD 3312.8
4	TC-ER, Individual & Overall Shield, 600 V 12 Pairs	14/1.628	USD 3608

## Data Availability

The data presented in this study are available on request from the first author. The data are not publicly available due to concerns regarding possible exploitation of the codes (developed by the first author) without permission preserving the authors’ copyrights.

## Supplementary Materials

The following supporting information can be downloaded at: https://www.mdpi.com/article/10.3390/s24216799/s1, instruction manual of the marine cargo crane.

## Appendix A

All the information related to the technical details of the marine cargo crane, which is the point of discussion in this article, is available in the instruction manual of the marine cargo crane. This manual can be requested from the first author of this article (Supplementary Material).

## Appendix B

1. https://www.wireandcableyourway.com/20-awg-2-pair-instrumentation-cable-pltc-overall-shield-300v (accessed on 22 August 2024).
2. https://www.wireandcableyourway.com/18-awg-2-pair-instrumentation-cable-pltc-individual-overall-shield-300v (accessed on 22 August 2024).
3. https://www.wireandcableyourway.com/16-awg-2-pair-instrumentation-cable-pltc-individual-overall-shield-300v (accessed on 22 August 2024).
4. https://www.wireandcableyourway.com/catalog/product/view/id/11485/s/16-awg-2-pair-xlp-cpe-instrumentation-cable-individual-overall-shield-ul-type-tc-600v/category/487/ (accessed on 22 August 2024).
5. https://www.wireandcableyourway.com/18-awg-2-pair-type-tc-er-instrumentation-tray-cable-individual-overall-shield-600v (accessed on 22 August 2024).
6. https://www.wireandcableyourway.com/16-awg-2-pair-type-tc-er-instrumentation-tray-cable-overall-shield-600v (accessed on 22 August 2024).
7. https://www.wireandcableyourway.com/16-awg-2-pair-fr-epr-cpe-instrumentation-cable-individual-overall-shield-ul-type-tc-600v (accessed on 22 August 2024).
8. https://www.wireandcableyourway.com/20-awg-12-pair-instrumentation-cable-pltc-individual-overall-shield-300v (accessed on 22 August 2024).
9. https://www.wireandcableyourway.com/18-awg-12-pair-instrumentation-cable-pltc-individual-overall-shield-300v (accessed on 22 August 2024).
10. https://www.wireandcableyourway.com/16-awg-12-pair-type-tc-er-instrumentation-tray-cable-overall-shield-600v (accessed on 22 August 2024).
11. https://www.wireandcableyourway.com/catalog/product/view/id/32315/s/14-awg-12-pair-type-tc-er-instrumentation-tray-cable-individual-amp-overall-shield-600v/category/487/ (accessed on 22 August 2024).
