# Supplementary material for: Enhancement of Operational Safety in Marine Cargo Cranes on a Container Ship Through the Application of Authenticated Wi-Fi Based Wireless Data Transmission from Multiple Sensors
Source: Sensors (Basel). 2024 Oct 23;24(21):6799. doi: 10.3390/s24216799 (PMC11548059; doi:10.3390/s24216799)
Supplement: Supplementary file 1 [file sensors-24-06799-s001.zip › sensors-3137936-supplementary.pdf]

TABLE OF CONTENTS

Manual No.	10179 (4 pcs); CD-ROM
Certificate for crane issued by:	GL
Certificate for foundation issued by:	GL
**IMO No.**	9144158
Name of ship	HELENE RICKMERS
Newbuilding No.	NB 170/III/9
Shipyard	Stettin Yard
Owners	m.s. "Helene Rickmers" Schiffs
**Project No.**	KR97038
Mfg. No.	62504309 - Crane 1; 62504310 - Crane 2; 62504311 - Crane 3
**Type of crane**	GL 4028-2
Collecting list	-
Crane specification	-
Order specification	489 5734 /G

SAFETY DOCUMENTS
Document names written with bold letters are documents with safety aspects.

		INSTRUCTION NO.	REV	NAME OF INSTRUCTION
GENERAL	1	1.000 E	e	SAFETY INSTRUCTIONS
		1.005 E	d	PREFACE
		1.009 E	a	GENERAL INSTRUCTIONS, CLEANLINESS IN HYDRAULIC SYSTEMS
		1.102 E	c	CONVERSION FACTORS
		1.518.1 SvED	e	HYDRAULIC AND ELECTRIC SYMBOLS
TECHN. DESCRIP.	2	KR97038		TECHNICAL DATA
		2.007.54 E	b	TECHNICAL DESCRIPTION
FUNCTION	3	3.110.62 E	-	HYDRAULIC FUNCTION
		3.131.8 E	b	STARTING AND STOPPING, ELECTRIC SAFETY FUNCTIONS, SINGLE CRANES
		3.142.13 E	-	CRANE CONTROL SYSTEM
		3.155 E	a	THERMOSTATIC CONTROLLED VENTILATION FAN
OPERATION	4	**PM40 1836-E00**	**a**	**CREW CHECKLIST**
		**PM40 5500-E00**	**b**	**OPERATING INSTRUCTION**
		**PM40 6062-E00**	**c**	**OPERATING INSTRUCTION, Crane 1, 3**
		**PM40 6063-E00**	**b**	**OPERATING INSTRUCTION, Crane 2**
		**PM40 6171-E00**	**-**	**CONTROLS AND INDICATORS**
		**PM41 0012-E00**	**-**	**EMERGENCY ESCAPE INSTRUCTIONS**
		**4.620.1 E**	**m**	**PARKING THE CRANE, SEA STOWING, GENERAL**
		**PM46 2505-E00**	**a**	**PARKING OF CRANE IN WIRES, Crane 2**
		4.630 E	-	HANDLING HATCHES BY DECK CRANE
MAINTENANCE	5	5.005.31 E	d	MAINTENANCE CHART
		5.018 E	a	INSPECTION OF STRESSED COMPONENTS AND STRUCTURES
		PM51 1700-E00	c	LUBRICATING CHART

TABLE OF CONTENTS

		INSTRUCTION NO.	REV	NAME OF INSTRUCTION
MAINTENANCE	5	5.150.1 E	b	LUBRICATING AND MAINTENANCE
		5.301.8 E	-	FILTERS
		5.302.16 E	d	HYDRAULIC OIL TANK
		5.315 E	a	SAMPLING OF OIL TO VERIFY THE CLEANLINESS IN HYDRAULIC SYSTEM
		5.421.22 E	d	LUBRICANTS FOR DECK MACHINERY
SERVICE	6			
	6.0	6.000 E	b	HOW TO ORDER SPARE PARTS
		6.000.1 E	a	STATEMENT REGARDING DELIVERY OF SPARE PARTS TO SWIVELS, LIFTING B
		6.000.2 E	-	RETURN FORM
		6.003.1 E	k	GLOBAL SERVICE
		6.004.1 E	as	MacGREGOR GLOBAL SERVICES
		6.005.1 E	c	INTRODUCTION
	6.1	6.100 E	b	MEASURES BEFORE STARTING, DURING AND AFTER COMPLETED SERVICE/M
		6.106.1 E	b	TROUBLE-SHOOTING CHART, OVERHEATING
		6.117 E	b	TROUBLE SHOOTING, PUMPS
		6.120.15 E	c	SLEWING GEARSET (PINION MODULE 14 AND 16)
		6.120.51 E	b	HYDRAULIC MOTOR
		6.120.78 E	-	COMPACT WINCH
		6.121.2 E	-	TROUBLE-SHOOTING CHART, HYDRAULIC MOTORS
		6.122.3 E	b	HYDRAULIC MOTOR, COMPACT
		6.125.14 E	-	FEED PUMP UNIT
		6.127.22 E	a	HIGH PRESSURE PUMPS
		6.189 E	e	HYDRAULIC PISTON ACCUMULATOR 388 0362 -801, -802, -803
	6.2	6.203.14 E	b	HYDRAULIC BRAKE MDA
		6.214 E	h	TIGHTENING TORQUES, SCREWS AND BOLTS
		6.216.61 E	e	ROPE REEVING DIAGRAM CRANES TYPE GL-2 (new version)
		6.222.1 E	a	HANDLING, INSTALLATION AND MAINTENANCE OF STEEL WIRE ROPES
		6.223.4 E	f	REPLACEMENT OF WIRES, ONE OR TWO FALL RIGGING
		625/1560 E	b	LIFTING BLOCK
	6.3	6.300.1 E	-	EMERGENCY OPERATION CARD CC2000
		6.303.22 E	c	CRANE CONTROL SYSTEM CC2000, SINGLE CRANES TYPE GL AND LC
		414 6848	f	SIGNAL OVERVIEW, CRANE CONTROL SYSTEM CC2000
		414 7012	f	ERROR MESSAGES, CRANE CONTROL SYSTEM CC2000
		6.305.1 E	b	ELECTRICAL EQUIPMENT, GENERAL MAINTENANCE
		6.307.11 E	b	SLIP-RING UNIT
		6.311.3 E	a	OIL LEVEL FLOAT SWITCH AND OVERHEATING PROTECTOR
		6.320.147 E	a	LIMIT SWITCHES IN HOISTING AND LUFFING WINCH SYSTEMS
		6.321.12 E	b	SLACK WIRE SAFETY SWITCH HOISTING AND LUFFING WINCHES
		**6.990.27 E**	**-**	**EMERGENCY RELEASE OF PARKING LOCK IN CASE OF POWER FAILURE BY U**
		**6.990.28 E**	**f**	**EMERGENCY BRAKE RELEASE IN CASE OF COMPLETE POWER FAILURE OPE**
EXTRA EQUIPMENT	7			
APPENDICES	8			
SPARE PARTS	9			SEE SEPARATE TABLE OF CONTENTS

TABLE OF CONTENTS

GROUP	DESCRIPTION	PART NUMBER		FIG
9				E 625-0014
9.0	HOW TO ORDER SPARE PARTS		b	6.000 E
9.1	HOISTING WINCH	188 0533-801	A/	625-1410.002B
	HYDRAULIC KIT, HOISTING WINCH	478 3728-801	B/B	625-1411.001A
	HYDRAULIC MOTOR	178 2573-801	B/B	625-1420.003B
	SPEED ENCODER, MOUNTING	378 2264-801	-/-	625-1421.002
	MULTI DISC BRAKE	178 2581-702	A/A	625-1430.003A
	LUFFING WINCH	188 0538-801	A/	625-1410.003B
	HYDRAULIC KIT, LUFFING WINCH	478 3719-801	A/A	625-1411.002A
	HYDRAULIC MOTOR	178 1934-701	C/C	625-1420.004B
	MULTI DISC BRAKE	178 2268-722	B/	625-1430.001B
	SLEWING GEAR	288 0867-801	E/	625-3255.002C
	HYDRAULIC MOTOR	388 3127-801	H/	625-1203B
9.2	PUMP UNIT	188 0298-801	B/B	625-4935.035A
	PUMP A4VG 125	287 9493-801	A/	625-4945.002B
	PUMP A4VG 125	287 9494-801	A/	625-4945.003A
	TANDEM ASSEMBLY KIT	388 5167-801	C/	625-4963A
	OIL COOLER ASSEMBLY	388 9899-801	D/	625-5740.001A
	OIL COOLER	287 6954-801	D/D	625-5735G
9.3	CRANE HOUSE MAIN COMPONENTS, Crane 1, 3	188 0718-801	-/-	625-6102.083
	CRANE HOUSE MAIN COMPONENTS, Crane 2	188 0719-801	-/	625-6102.084
	PARKING LOCKING MOUNTING, Crane 2	288 1444-801	B/	625-6160.001B
	CRANE TOP MOUNTING	288 1878-801	C/	625-6459.007B
	OIL TANK	188 0343-801	H/H	625-5865.007D
	FEED PUMP UNIT	388 0364-802	H/H	625-5181.001A
	SLACK WIRE DEVICE	288 1616-801	B/	625-6250.007
	VENTILATION FAN, ASSEMBLY	288 1118-801	C/	625-6625.001B
	CAB	188 0375-801	-/-	625-6545.011
	JIB TOP MOUNTING	288 1527-801	C/	625-6244.007A
	JIB BEARING MOUNTING	389 1451-801	-/	625-6170.018
	JIB BEARING HOUSING	389 1454-801	B/	625-6170.019A
	CRANE JIB, YARD MOUNTING	489 5409-801	A/	625-6275.004B
	LIFTING BLOCK	288 1424-803	C/	625-7013.043B
	LIMIT SWITCHES BOX, HOISTING	288 1103-803	D/	625-6245.172
	LIMIT SWITCHES BOX, LUFFING	288 1104-804	J/	625-6245.183
	INCLINOMETER, C	388 0176-801	B/B	625-6509A
	SLEWING BEARING MOUNTING	389 1628-801	B/	389 1628
	SLEWING BEARING	288 1508-801	C/	288 1508
	SLEWING RIM YARD MOUNTING	388 7330-801	E/	388 7330
	JIB WELDING, Crane 1	188 0720-801	B/	188 0720
	JIB WELDING, Crane 2	188 0750-801	A/	188 0750
	LOAD WIRE ROPE, SEE TECHNICAL DATA	388 9794-808	J/	-
	LUFFING WIRE ROPE; SEE TECHNICAL DATA	388 9795-807	H/	-
9.4	TWO SPEED VALVE	178 2568-801	A/A	625-7239.002D
	FLUSH - UNLOADING UNIT	178 2569-801	-/-	625-7802.003B

TABLE OF CONTENTS

GROUP	DESCRIPTION	PART NUMBER		FIG
9.4	VALVE UNIT	278 2101-801	A/A	625-7207.001A
	UNLOADING UNIT	278 2088-801	A/A	625-7802.002A
	DIRECTION VALVE	287 5950-801	L/	625-7508C
	FLUSH AND UNLOADING UNIT	388 3580-801	C/C	625-7449B
	FLUSHING VALVE	388 3579-801	F/F	625-7287C
	UNLOADING UNIT	388 3576-801	B/B	625-7291C
	HYDRAULIC CYLINDER, C	388 5046-801	C/	625-4493
	FILTER UNIT, INLET	189 0418-801	-/	625-7314.003
	FILTER UNIT, OUTLET	188 0118-801	B/	625-7314.002A
	ACCUMULATOR	388 0362-801	F/	625-7951E
	HYDRAULIC HOSES			625-7970G
	FILTER ELEMENT	489 3104-801	C/	-
	HYDRAULIC CIRCUIT DIAGRAM	188 0689	C/C	188 0689
9.5	CONTROLLER PANEL	414 6792-801	A/A	625-8596
	CONTROLLER, HOISTING	314 2005-802	D/	625-8591C
	CONTROLLER, LUFFING/SLEWING	314 2006-802	D/	625-8592C
	POWER SUPPLY CE, C	314 3437-801	A/A	625-8562.001A
	SLIPRING UNIT	314 3481-801	A/	625-8750.004C
	BLOCK DIAGRAM	314 3904	-/	314 3904
	CIRCUIT DIAGRAM	314 3905	A/	314 3905
	COMPONENT LIST	414 6983	-/	414 6983
	EL. ASSY CRANE	114 1324-801	-/	114 1324
	EL. INST. CRANE HOUSE	114 1224-801	F/F	114 1224
	DRIVER'S DESK	114 1210-801	C/C	114 1210
	CONTROL PANEL	214 1734-801	C/	625-8597.001
	ELECTRIC PANEL	214 1803-801	-/	625-8550.001
	CUBICLE A	214 1802-801	-/-	214 1802
	ELECTRONIC BOX MB	314 3500-801	F/F	625-8607.001A
	EL. INST. JIB LIGHT	114 1314-801	B/	625-8565.001
	EL. INST. JIB LIGHT	114 1315-801	-/	625-8567.001
9.7	RESCUE EQUIPMENT	388 9561-801	A/	624-2500B
	ANTI-DAZZLING SCREEN MOUNT	287 7010-801	C/C	624-2510
	FIRE EXTINGUISHER, MOUNT.	488 8556-801	E/E	624-2520A
	ASH TRAY	6931 4692-001		-
	EL. INST. CRANE PARKING LOCK, Crane 2	114 1286-801	A/	114 1286
	EL. INST. CABIN PARKING LOCK, Crane 2	114 1285-801	B/B	114 1285
	EL. INST. CUBICLE A PARK. LOCK, Crane 2	214 1797-801	A/	214 1797
	MOUNTING IMPULS UNIT, Crane 2	288 1845-801	-/	288 1845
	BLOCK DIAGRAM, PARKING LOCK, Crane 2	314 3787	-/	314 3787
	CIRCUIT DIAGRAM; PARKING LOCK, Crane 2	314 3788	A/	314 3788
	EL. INST WARNING LIGHT	114 1234-801	B/	114 1234
	EL. INST. WARNING LIGHT	114 1235-801	A/	114 1235
	EL. INST. CABIN WARN LIGHT JIB	314 3908-801	-/	314 3908
	EL. INST. DECK LIGHT	214 1801-801	A/A	214 1801
	EL. INST. CABIN DECK LIGHT	214 1800-801	A/A	214 1800
	EL. INST. EMERGENCY LIGHT	114 1325-801	A/	114 1325
	EL. INST. POSITION LIGHT	114 1326-801	-/-	114 1326

Safety Instructions

Cargo Handling Equipment

The MacGREGOR crane is a cargo handling device. It is designed and manufactured to meet international cargo handling standards for quality, safety and performance within capacity specifications and outreach as per certificate.

The crane is designed for operation in harbour or sheltered water environments where there is no significant movement of the ship due to wave action.

Testing and examination

The crane shall be tested and thoroughly examined and certified by a competent person after any substantial repair.

Safe Operating Conditions, Proper Instructions and Precautions

The responsible officer on board shall ensure that the crane is operated under safe conditions, by personnel familiar with its functions and operating instructions. All safety and precautionary issues contained in this manual must be observed. Proper instructions and precautions are prerequisites for proper use.

Preparation for Use and Operation According to Manual

The crane operator, authorized by the responsible officer on board, must be familiar with all safety issues contained in this Chapter and with preparation for use and operating instructions before work is started.

Maintenance and Service

Prerequisite for proper use is regular maintenance (see Chapter 5) and service (see Chapter 6).

MacGREGOR are available for full service of this cargo crane. MacGREGOR will take responsibility only for its own professional services. Our product guarantee is not valid unless MacGREGOR authorized service is used.

The use of proper Spares and Exchange details

MacGREGOR shall be contacted for ordering of exchange equipment and spares for this crane. Articles supplied by MacGREGOR are designed and quality tested for this application and are supplied with our warranty for correct function and safe working of the crane.

MacGREGOR accepts no liability for the functioning of the crane systems, nor for the safe use of the crane, unless original spare parts are used.

Supervision, Instructions, Signs and Warnings

Proper use of this crane and its equipment is possible as long as the owners, users and operators maintain the crane to meet full operational standards. This includes the replacement of signs, instructions and warnings as per delivery. MacGREGOR does not accept responsibility for any party who fails to ensure that this manual, signs, instructions, and warnings are legible and easily accessible to personnel.

Improper Use

1. Elevation of persons.
2. Exposing persons to the risks of falling cargo.
3. Overloading.
4. Unlocking the crane or crane jib or operating the equipment at sea without full acceptance and control of personnel and equipment safety.
5. Unprofessional, intended or unintended, adjustment of safety locks, hydraulic or electric settings.
6. Disregard of occupational and safety standards of work.

Functions, Installations and Equipment

General

The MacGREGOR crane has built-in safety functions and installations to allow for safe normal use, operation, care and control. This is applicable to instructed personnel without specific professional education or certification, but working according to this manual.
Specific service and repair of the electrical, hydraulic and the mechanical functions, controls and settings require professional service personnel knowledgeable of risks involved.

Regular Care and Control (Maintenance)

This manual contains checklists for regular care and control of crane equipment, wire ropes and hydraulic filters. These checklists routine contain inspections and maintenance regularly prescribed for safe function, e.g.:

Chapter 4 contains:
Safety Precautions Checklist for Single and Twin Cranes.

Chapter 5 contains, if applicable:
Inspection of Stressed Components and Structures.
Lubrication chart; Crane.
Lubrication chart; Twin Platform.
Lubrication chart; Cable Winch and Stabilizing Equipment.
Lubrication chart; Power Swivel.
Filters.
Handling and Checking Steel Wire Ropes.
Checking brake settings.

Before maintenance and service work is started, certain measures and procedures must be followed.

Chapter 6 contains:
Measures before Starting Service/Maintenance Work.
Electrical Equipment, General Maintenance.

Safety Functions and Equipment

Specific safety equipment is installed and delivered with this crane and must be available for its intended use. The owner/operator of the ship and its equipment is responsible to ensure that safety functions, installations and equipment are maintained to operational standards and are not being removed, bypassed or worn to non-working condition.
This manual features important presentations of safety functions, installations and equipment. A summary is presented below.

1. Safety Functions

a. Maneuvring functions:
   1. All the crane's working functions are stopped and the brakes will automatically be activated when the emergency stop button is pressed. The button is loacated in cabin. This action activates safety stops and automatic lock positions.
   2. At twin crane operation the crane movements automatically stop and Synchronizing failure indicator lamp lights if the hooks or jibs get out of syncronizing position.
   3. The crane has several fail-safe functions for over-ruling or stopping the operator's maneuvring in the event that normal hydraulic or electrical functions fail, a sudden uncontrolled movement occurs or during overspeed operation (see below).
   4. There are limit switches for reducing speed of or stopping all dangerous motions.

b. Hydraulic functions:
   1. Hydraulic valve functions are set for exact pressure. Any change in the settings adversely affects crane operation and must exclusively be set by professional service personnel (see instructions Hydraulic function and High pressure pumps).
   2. Hydraulic oil pressure sensor automatically indicate an overload. For safe hoisting or lowering, the system automatically switch from a highspeed movement to a lowspeed movement or may stop the hoisting movement.

3. In case of a ruptured high-pressure oil pipe/ hose or other feed pressure drops, the oil feed pressure will collapse. A feed pressure switch stops the crane and the mechanical brakes are automatically applied.
4. Each winch has a hydraulic motor brake valve flanged to the motor. Mounted directly to the motor it also provides hydraulic braking in case of a pipe rupture. (Valid for winches equipped with low speed motors.)
5. If there is brake band failure, e.g. caused by worn-out lining, hydraulic braking capacities by the pump and motor brake systems are still a-vailable. (Valid for winches equipped with low speed motors.)
6. If there is a multiple-disc failure caused by worn out brake discs, sticking cylinder, brake pilot valve or valve spool, hydraulic braking by pumps and motor is still avaiable. (Valid for winches equipped with high-speed motors).
7. In case of overheating oil, a temperature sensor stops the main electric motor, locking crane operation.
8. Efficient oil filters with exclusive bypass to the hydraulic oil reservoir protects the system from harmful oil conditions. A warning light on the operator's panel indicates if the filter has to be changed. Dirty filter might cause mal function to the system.
9. If the hydraulic oil tank oil level drops below the minimum permissible level.
   A warning light on the operator's panel is activated.
   An indicator stops the main electric motor and crane functions are closed while all brake systems are automatically activated.

c. Electrical functions:
   1. During a complete power failure all solenoid valves are de-energized, causing brakes to be activated.
   2. The electronic cabinets have power failure indicators. This function closes the electronic signals down which automatically activates all mechanical brakes.
   3. Heat sensors located in the main electric motors and feed pump motor stops all crane operations and activates all brakes.
   4. The overcurrent protections on all electric motors prevent the electric motors from overloading.
      The main electric motor and feed pump electric motor overcurrent protections stop all crane operations and activates all brakes.
   5. A separate electrical feed for the cranehouse lights operator's cab and on jib, maintains function of lights even during feed disruption to the crane operating functions. As there is not a separate emergency illuminating system for the crane (this is dependent on the ship's systems) the owner of the crane must provide a battery type flash-light for the operator's cab.

d. Mechanical functions:
   1. Automatic sensors recognize dangerous wire slacks and stop hoisting and luffing movements that threaten safe control and the equipment itself.
   2. Each working circuit has at least two independent brake systems.
      Dynamic braking, deceleration, is achived by the regenerative function of motor and pump. (hydraulic braking).
      True static braking (parking brakes) is provided by multiple disc-brakes or band brakes. There are band brakes for low speed winches and an extra mechanical pawl-brake for luffing. The mechanical brakes are automatically activated in the event of dangerous oil pressure. High speed winches are provided with multiple disc brakes. One for each gear unit.
      Each slewing gear unit is equipped with one multiple disc brake.

2. Safety Installations

a. Warning lights, measuring instruments and meters:
   1. The operator's panel is provided with warning lights which indicate clogged filters or low oil level in the oil tank.
   2. During crane start-up a warning light on the operator's panel indicates that starting procedures are still running and that working conditions are not acceptable before the lamp goes out. Maneuvres are not to be ordered before the warning light goes out.

3. A pushbutton siren is located in the crane operator's cabinet, allowing the operator to alert persons in the working area (e.g. against prohibited riding on the load or against load movements).
4. Measuring instruments and meters (e.g. for checking the crane functions, adjusting valve positions, and setting functional positions) are supplied as per agreement with the crane buyer/owner. Tools and equipment which are eliminated from the original order to MacGREGOR are delivered at the discretion of the owner.

b. Covers and houses:
   1. Unauthorized visitors in the cranehouse, especially during crane operation, is prohibited. The crane is a dangerous working area, and requires special permission to enter. Instructions given by the superintendent or responsible officer must be followed.
   2. Covers for electric boxes must be opened only by authorized personnel.

c. Alert and warning signs:
   The crane is equipped with a system of danger, warning, caution and notice signs. These signs are presented on pages 6 - 15.

3. Safety Equipment

a. Locks, seals and keys:
   1. Functions and equipment that are essential for oil pressure settings, coordination of controls and crane movements or expose dangerous high-voltage electricity are locked. All keys to locked positions and switches must be kept and controlled by the responsible officer onboard and are only to be used by qualified personnel.
   2. Safety valves for hydraulic pressure settings and switches for electric safety circuits are sealed to secure correct position and trim. Sealed equipment may be opened and positions changed only after consulting the MacGREGOR service organization for proper handling.

b. Safety belt, rescue line, safety chains:
   1. For work or entry to the open-air crane top, authorized personnel must wear a safety belt. The connector hook should be anchored to rails and fixed position while staying or working on crane top.
   2. The rescue line and equipment for emergency evacuation of the crane operator must always be easily accessible on the interior wall-side of the crane cabin. The equipment status and completeness must be inspected regularly by the responsible officer onboard.
   3. The safety chains on the open-air crane top and work platforms inside the crane, must be used for attendant's safety during stay and work.

c. Firefighting equipment:
   A fire extinguisher shall be made available by owner in the operator's cabin.

   The fire extinguisher is for emergency use inside the crane.

   The equipment must be regularly inspected for proper pressure reading at the meter and must be serviced annually. This is to be supervised by the responsible officer on board.

d. Test device:
   At delivery, the MacGREGOR crane is equipped with two pressure gauges.

Danger, Warning, Caution and Notice Signs

Principles of Risk Classification

1. **DANGER SIGNS** are attached to equipment or are related to operations that will expose personnel to a life-threatening danger (e.g. high voltage electricity).

   Safety installations, covers or houses prevent exposure during normal working conditions, but cannot eliminate the imminent risk for any unprofessional/non-certificated person, should the danger sign not be followed.

   Those risks classified as DANGER sign are presented and explained below with this presentation.

2. **WARNING SIGNS** are attached to equipment or are related to operations that will expose personnel to severe risk of physical injury. The injury is not expected to be life-threatening. A warning sign is also used to warn against misuse that risks severe equipment and property damage.

   The danger does not exist unless protection installations or devices are removed, providing procedures are followed.

3. **CAUTION SIGNS** are attached to equipment or related to operations that may produce a physical risk to any person who is not cautious or aware of the risk.

   Any person should be able to avoid the danger.

4. **NOTICE SIGNS** are attached to equipment and provide references to procedures, and instructions or policies to follow to avoid danger to the function and operation of the equipment or damage to the equipment itself.

   Responsible officers onboard must ensure that the signs are in good condition and readable to any person admitted to the crane area.

   Replacement signs must follow international and national standards. Signs of non-MacGREGOR design are not allowed. MacGREGOR does not accept responsibility for unauthorized signs.

Specific Presentation of Signs and Their Message

All signs attached to the crane are illustrated on the following pages. Each sign is given a specific number to which it is referred.

Alert / Warning signs

Presentation

PM40 7101-E00<br>NOTICE SIGN	**REPORT TO SUPERINTENDENT OR OPERATOR'S ROOM FOR PERMIT TO ENTER**<br>Any visitor to the crane must obtain a special permit from responsible officer on board. For authorized entry during crane operation, the operator must be fully knowledgeable of safety precautions. The foundation, platform and crane are a dangerous working environment and requiers that the authorized visitor understands relevant safety.<br>This sign is at the bottom entrance to the crane foundation.
PM40 7102-E00<br>WARNING SIGN	**DO NOT ENTER DURING CRANE OPERATION - MOVING CRANE HOUSE AND MACHINE PARTS ...**<br>Authorized entry requires that visitor is aware of the character of general risk and that visitor has adequate Manual information about how to avoid the exposure.<br>This sign is at the bottom entrance to the crane foundation.
PM40 7103-E00<br>CAUTION SIGN	**HIGH NOISE LEVEL - USE EAR PROTECTION!**<br>Motors and winches produce a noise level that is harmful to personnel after lengthy exposure. To eliminate the risk, adequate ear protection is required.<br>This sign is at the bottom entrance to the crane foundation.
PM40 7104-E00<br>WARNING SIGN	**LADDER WILL ROTATE WITH CRANE - WATCH OUT!**<br>This is specifically warn of exposure of the ladder leading up to the cranehouse. Upon crane rotation, this ladder rotates and moves suddenly without warning.<br>This sign is at the bottom entrance to the crane (twin) platform.
PM40 7105-E00<br>WARNING SIGN	**RISK OF GETTING CAUGHT BETWEEN WIRE AND DRUM - WATCH OUT!**<br>At start of and during winch operation there is a risk of getting caught and jammed by moving wire. A safe distance from wire and drums must be maintained and adequate caution must be regarded.<br>Attached to the crane wall surfaces close to winches.
PM40 7106-E00<br>WARNING SIGN	**RISK OF GETTING CAUGHT BETWEEN DRUM AND BRACKET - WATCH OUT!**<br>This sign warns the visitor about the risk of getting jammed during drum rotation.<br>Adequate caution must be regarded.<br>Attached to brackets and walls of work platforms inside crane

PM40 7107-E00<br>WARNING SIGN	**RISK OF GETTING CAUGHT BETWEEN BRAKE CYLINDER AND WALL - WATCH OUT!**<br>During service or replacement work, the serviceman may be exposed to the power of released brake cylinders.	Attached at brake cylinders, narrow spaces near them, at work platforms
PM40 7108-E00<br>WARNING SIGN	**RISK OF GETTING CAUGHT BETWEEN WIRES DURING CRANE OPERATION - WATCH OUT!**<br>This sign warns about the risk of moving wires that may catch and jam personnel.<br>The authorized visitor must be aware of the risk and be cautious to avoid being seriously hurt.	This sign is attached to the steel bracket side at the open-air crane top entry.
PM40 7109-E00<br>WARNING SIGN	**RISK OF FALLING DOWN - USE SAFETY BELT!**<br>During any visit to the crane top (e.g. for service jobs), the authorizing officer and the person entering to the area must fully understand the risks involved.<br><br>The safety belt must be worn during any visit to the crane top.	This sign is attached to the steel bracket side at the open-air crane top entry.
PM40 7110-E00<br>CAUTION SIGN	**UNIT UNDER CONSTANT SPRING TENSION. USE PRESCRIBED TOOL FOR SERVICE**<br>The label warns of the release of tension power of brake cylinders.<br>The exposed force of cylinders might expose personnel to specific risk.<br>The risk is exposed only during service or replacement work.	Attached at brake cylinders.
PM40 7111-E00<br>CAUTION SIGN	**ROTATING FAN - KEEP HANDS OFF!**<br>The rotating fans are protected by grids and the risk is for anyone who sticks fingers or hand inside. This can be avoided by sign information.	Attached to fan houses on the open-air crane top and to wall surfaces near the top roof of the crane.
PM40 7112-E00<br>DANGER SIGN	**RIDING ON CARGO FORBIDDEN**<br>For the attention of the crane operator, to observe that the crane is a Cargo Handling device.<br><br>The crane operator is obliged to stop operation and to alert any person riding to keep off. The operator’s alarm bell, in the crane operator’s cabin, may be used, preferably.	Attached on the wall side of the crane operator’s cabin.
PM40 7113-E00<br>NOTICE SIGN	**HOT OIL AND SURFACES - WATCH OUT!**<br>During crane operation, surfaces of oil heated equipment are extemely hot.<br>Service personnel dismounting such equipment (e.g. checking and setting valves) must be aware of this exposure.	Attached to surfaces inside the cranehouse, close to pumps, valves, pipes and manifold blocks. Also in the foundation close to oil tank and pipes.

PM40 7114-E00<br>WARNING SIGN	**DANGEROUS OIL PRESSURE - BE CAREFUL!**<br>The oil pressure inside oil pressure pipes is 25 - 350 bar.<br>Under normal working conditions, this exposes no risk for personnel because of rigorous pipe quality control and dimensions and specifications applied to the equipment. For specific service work, exchange of parts or equipment, the exposure may be dangerous for unqualified or uninformed personnel.<br>Instructions for service must be followed and must be restricted to qualified personnel only.<br>Attached to surfaces close to pipes inside the cranehouse.
PM40 7115-E00<br>WARNING SIGN	**SLIPPERY SURFACES - WATCH OUT!**<br>Also under normal working conditions, all walking surfaces are slippery from dirt, oil spill, etc. The responsible officers onboard must have the crane ladders and work platform surfaces cleaned regularly. Any person who is admitted to the area must be aware of the risk of slippery surfaces.<br>This sign is attached generally to the walls of work platforms.
PM40 7116-E00<br>NOTICE SIGN	**FAN STARTS AUTOMATICALLY WITHOUT NOTICE, AT +10°C (+50°F)**<br>Under normal working conditions, of risk is not exposed to any person near of fans. During service or upon removal of covers, the risk must be recognized. Such operation is restricted to qualified personnel only.<br>Attached to cooling fans, at the open-air crane top, and at the inside top roof.
PM40 7117-E00<br>DANGER SIGN	**HIGH VOLTAGE INSIDE - DO NOT OPEN! AUTHORIZED PERSONNEL ONLY**<br>High voltage equipment, couplings and boxes are covered and do not expose risk unless covers or hoses are removed. Access is restricted to qualified and authorized personnel only.<br>Attached to electrical equipment and boxes where 110 V or 440 V is exposed after covers are removed.
PM40 7118-E00<br>WARNING SIGN	**RISK OF FALLING DOWN - APPLY SAFETY CHAIN!**<br>During any visit to the work platforms, the authorizing officer and the person entering the area must fully understand the risks involved.<br>The safety chains (at work platforms) must be used to prevent personnel from falling.<br>Attached to the steel bracket side at the open-air crane top platform, and at the crane work platforms inside the cranehouse.
PM40 7122-E00<br>DANGER SIGN	**RISK OF BODILY INJURY - SUSPENDED LOAD - WATCH OUT!**<br>The crane operator must ensure that the suspended load does not expose personnel below to risks of falling material.<br>The crane operator must stop operation and alert personnel.<br>The operator’s alarm bell in the crane operator’s cabin is preferably used.<br>Applied on the wall side of the crane operator’s cabin.

PM40 7123-E00<br>WARNING SIGN	**RISK OF GETTING CAUGHT BETWEEN WIRE AND WIRE SHEAVES - WATCH OUT!**<br>This sign warns of the risk of moving wires that may catch and jam personnel.<br>The authorized visitor must be aware of the risk and be cautious to avoid being seriously hurt.<br>This sign is attached to the steel bracket side of the open-air crane top entry.
PM40 7124-E00<br>DANGER SIGN	**RISK OF BODILY INJURY. EMERGENCY OPERATION, AUTHORIZED PERSONNEL ONLY OPERATE WITH UTMOST CARE, SEE INSTRUCTION**<br>Emergency operation of luffing and slewing, in the event of complete power failure or main pump unit failure, is extremely difficult and dangerous.<br>This operation must be undertaken with great care and only by skilled personnel. Instructions must be observed at all times.<br>Attached to the wall side of the crane operator’s cabin.
PM40 7125-E00<br>DANGER SIGN	**DANGER - KEEP OUT OF WORKING AREA!**<br>The working area of the crane, over deck as well as over the deck-sides, is dangerous for personnel, equipment or property. Items lifted or articles dropped may cause death, injury or severe damage.<br>The crane operator must stop operation and alert personnel.<br>The operator’s alarm bell must also be used for attention.<br>Attached on both sides of the crane jib.
PM40 7126-E00<br>DANGER SIGN	**220V INSIDE, SEPARATE FEED, EVEN WHEN MAIN SWITCH (HA) IS SWITCHED OFF. DO NOT OPEN! AUTHORIZED PERSONNEL ONLY.**<br>High voltage equipment, couplings and boxes are covered and do not expose risk unless covers or hoses are removed.<br>Boxes with this sign are powered even if main switch has shut off other electrical power. Access is restricted to qualified and authorized personnel only.<br>Attached to electric boxes at work platforms and in the operator's cabin.
PM40 7127-E00<br>DANGER SIGN	**220V INSIDE. DO NOT OPEN! AUTHORIZED PERSONNEL ONLY**<br>This high voltage equipment is covered and does not expose risk unless cover is removed.<br>The electrical equipment inside cover is powered even if the main switch has shut off other electrical supply.<br>Access is restricted to qualified and authorized personnel only.<br>Attached to the cover of slipring device in the foundation.
PM40 7129-E00<br>NOTICE SIGN	**STARTS AUTOMATICALLY WITHOUT NOTICE AT +25° (+80°F)**<br>Under normal working conditions, of risk is not exposed to any person near of fans. During service or upon removal of covers, the risk must be recognized. Such operation is restricted to qualified personnel only.<br>Attached to cooling fans, at the open-air crane top, and at the inside top roof.

PM40 7130-E00<br>WARNING SIGN	**DANGEROUS OIL PRESSURE. BE CAREFUL!**<br>The oil pressure inside oil pressure pipes is 25 - 350 bar.<br>Under normal working conditions, this exposes no risk for personnel because of rigorous pipe quality control and dimensions and specifications applied to the equipment. For specific service work, exchange of parts or equipment, the exposure may be dangerous for unqualified or uninformed personnel.<br>Instructions for service must be followed and must be restricted to qualified personnel only.<br>Attached to surfaces close to pipes inside the cranehouse.
PM40 7131-E00<br>WARNING SIGN	**RISK OF GETTING CAUGHT BETWEEN DRUM AND BRACKET - WATCH OUT!**<br>This sign warns the visitor about the risk of getting jammed when the drum is rotating.<br>Adequate caution must be exercised.<br>Attached to brackets and walls of work platforms inside the crane.
PM40 7132-E00<br>NOTICE SIGN	**HOT OIL AND SURFACES - WATCH OUT!**<br>Surfaces of equipment in which there is hot oil can be extremely hot when the crane is in operation. When service personnel are working on such equipment (e.g. checking and setting valves) they must be aware of the danger.<br>Attached to surfaces inside the crane housing, close to pumps, valves, pipes, and manifold blocks. Also in the foundation, close to oil tank and pipes.
PM40 7134-E00<br>WARNING SIGN	**RISK OF GETTING CAUGHT BETWEEN WIRE AND DRUM. WATCH OUT!**<br>When the winch is started and during operation, there is a risk of getting caught and jammed by moving wire. A safe distance from wires and drums must be maintained and adequate caution must be exercised.<br>Attached to the crane wall surfaces close to winches.
PM40 7135-E00<br>WARNING SIGN	**RISK OF GETTING CAUGHT BETWEEN DRUM AND BRACKET - WATCH OUT!**<br>This sign warns the visitor about the risk of getting jammed when the drum is rotating.<br>Adequate caution must be exercised.<br>Attached to brackets and walls of work platforms inside the crane.
PM40 7136-E00<br>WARNING SIGN	**RISK OF GETTING CAUGHT BETWEEN WIRES DURING CRANE OPERATION - WATCH OUT!**<br>This sign warns about the risk of moving wires, that may catch and jam personnel.<br>Authorized visitors must be aware of the risk and be very cautious to avoid serious injury.<br>This sign is attached to the steel bracket at the open-air crane top entry.

PM40 7137-E00<br><br>WARNING SIGN	**BEFORE USING GRAB OR CARGO SPOTTING EQUIPMENT MAKE SURE THAT THE CORRECT FUSES ARE INSTALLED FOR THE EQUIPMENT TO BE USED.**<br>**TO SELECT THE CORRECT FUSES, SEE FUSE LIST ON THE INSIDE OF THE FUSE BOX DOOR.**<br>The grab equipment has a larger (electric) motor than the cargo spotting equipment. This must be taken into consideration when using the same cable winch to operate the cargo spotting and grab equipment.<br>Before switching over from grab to cargo spotting equipment, or vice versa, the fuses must be changed in the fuse box.<br>If the wrong fuses are used, the following problems will occur:<br>1. When the cargo spotting equipment is used with the fuses intended for the grab equipment. The cargo spotting motor will be overloaded and can be seriously damaged by fire. Overloading the motor can also cause short-circuiting in the motor cables and they can also be seriously damaged.<br>2. When the grab equipment is used with the fuses intended for the cargo spotting equipment. The fuses will blow immediately and the equipment will not be damaged.
	This sign is attached to the fuse box at the main electric cabinet (HC).

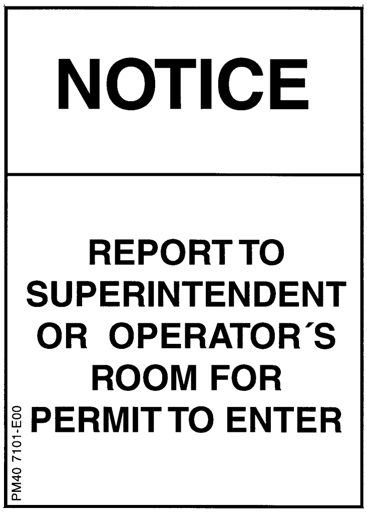

Fig. 1. PM40 7101-E00

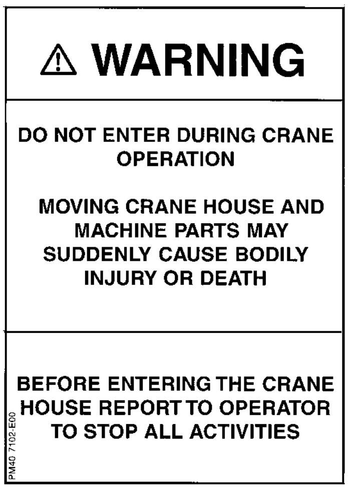

Fig. 2. PM40 7102-E00

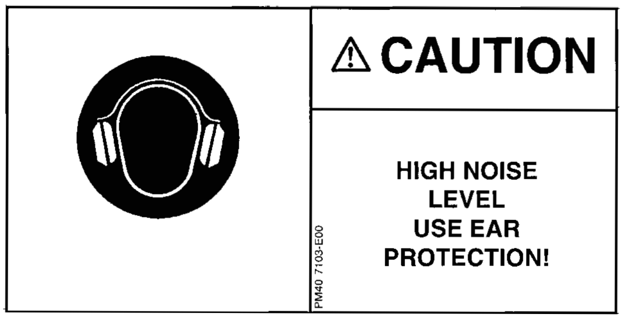

Fig. 3. PM40 7103-E00

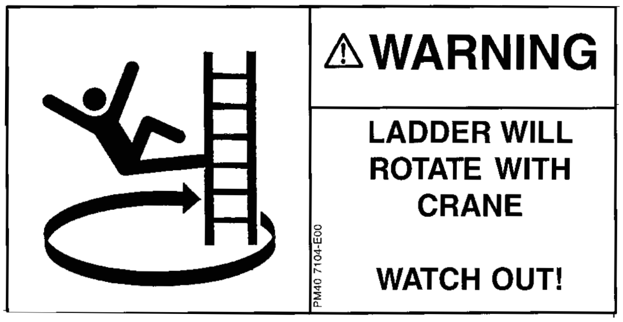

Fig. 4. PM40 7104-E00

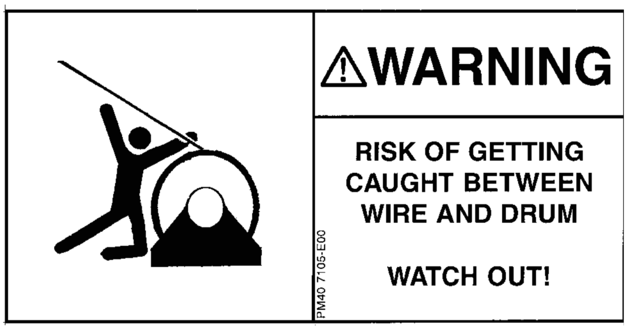

Fig. 5. PM40 7105-E00

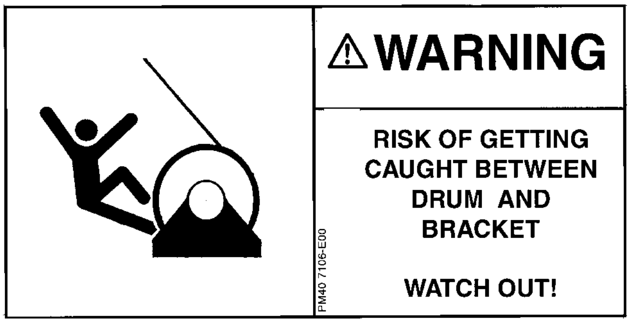

Fig. 6. PM40 7106-E00

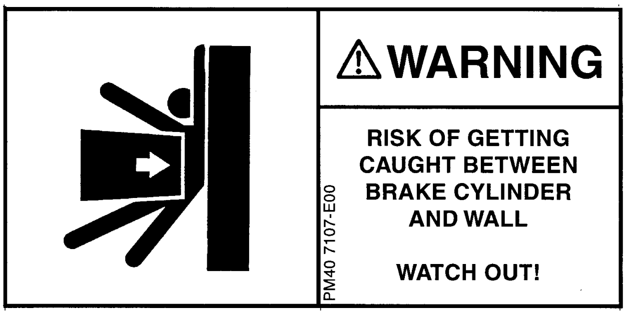

Fig. 7. PM40 7107-E00

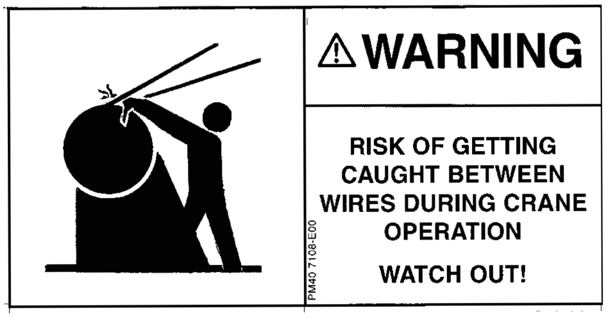

Fig. 8. PM40 7108-E00

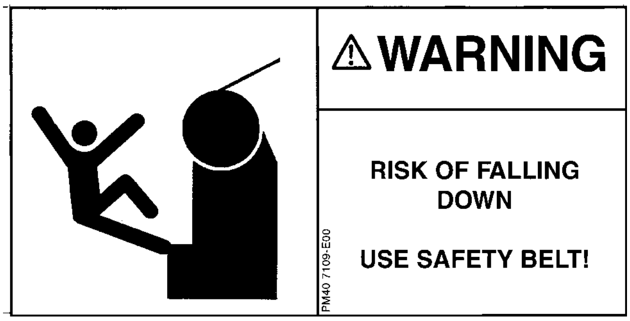

Fig. 9. PM40 7109-E00

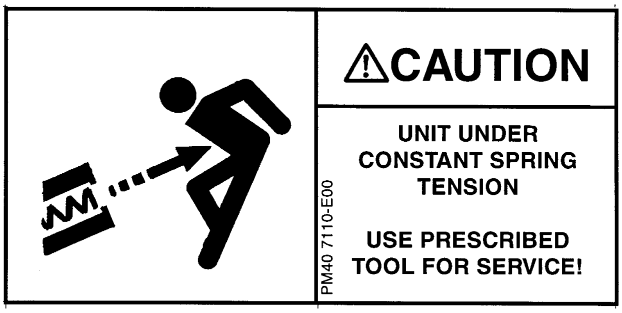

Fig. 10. PM40 7110-E00

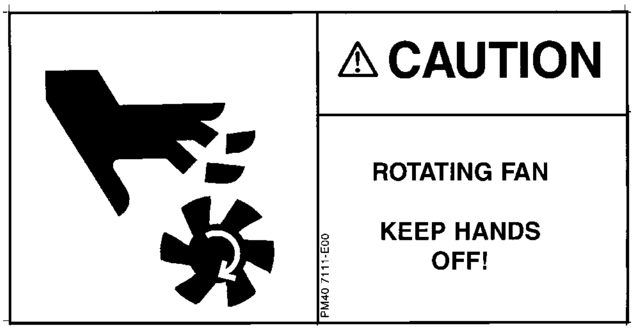

Fig. 11. PM40 7111-E00

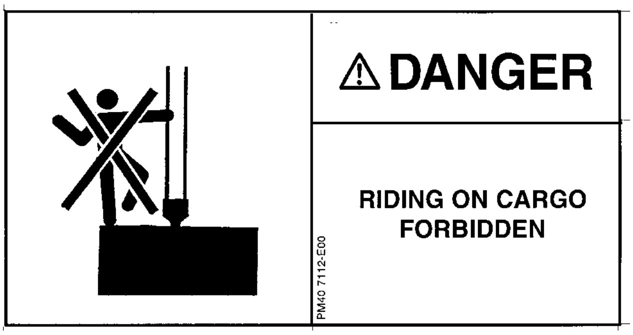

Fig. 12. PM40 7112-E00

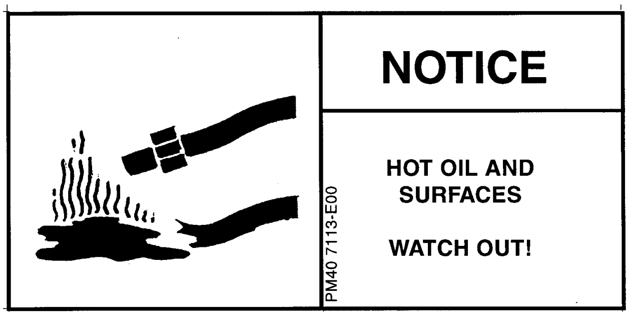

Fig. 13. PM40 7113-E00

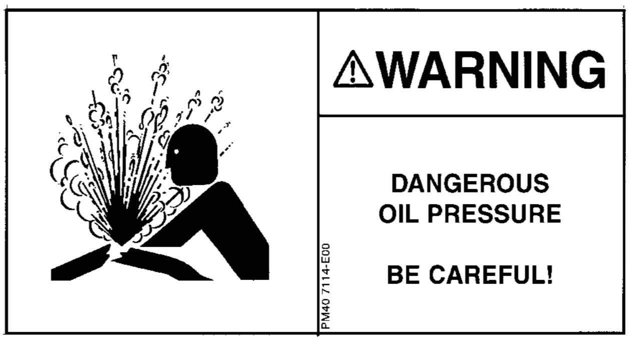

Fig. 14. PM40 7114-E00

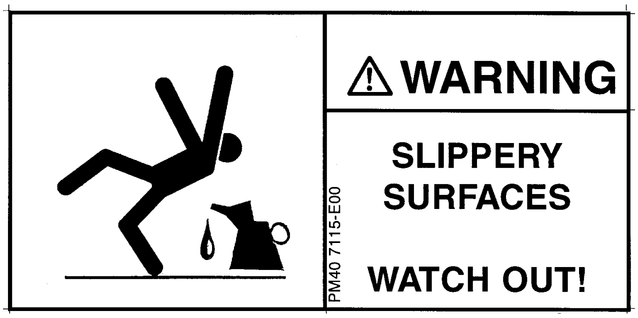

Fig. 15. PM40 7115-E00

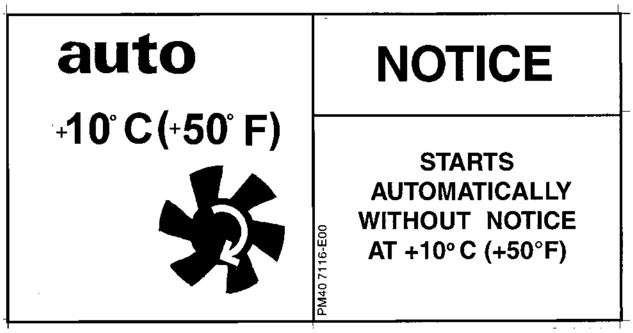

Fig. 16. PM40 7116-E00

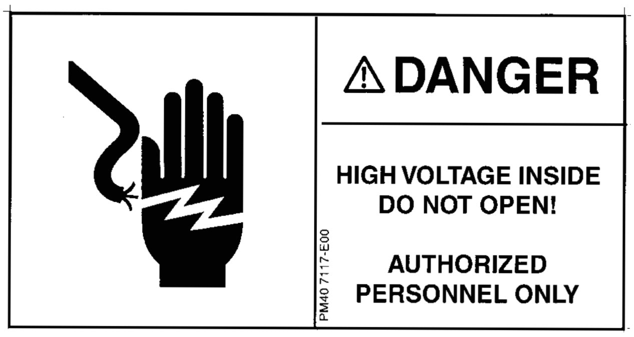

Fig. 17. PM40 7117-E00

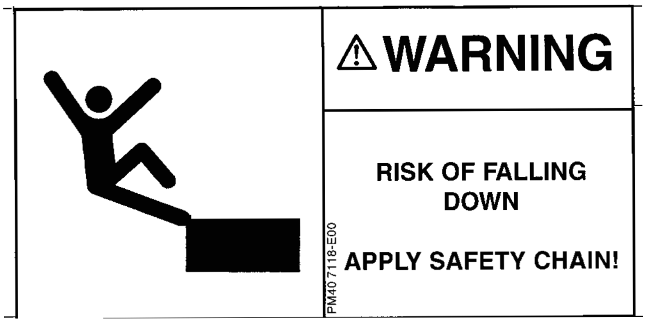

Fig. 18. PM40 7118-E00

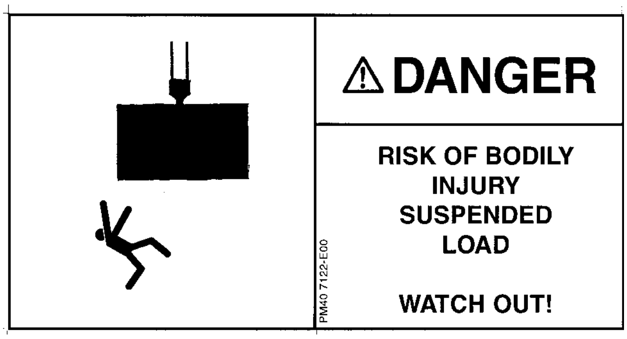

Fig. 19. PM40 7122-E00

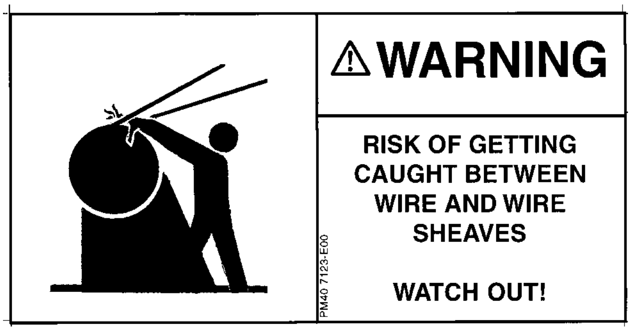

Fig. 20. PM40 7123-E00

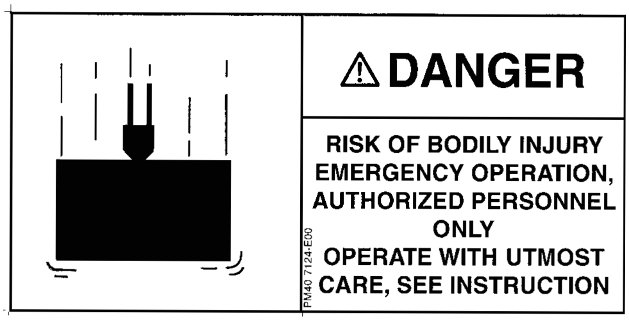

Fig. 21. PM40 7124-E00

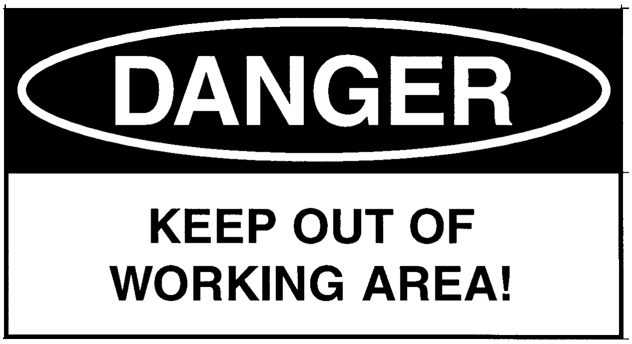

Fig. 22. PM40 7125-E00

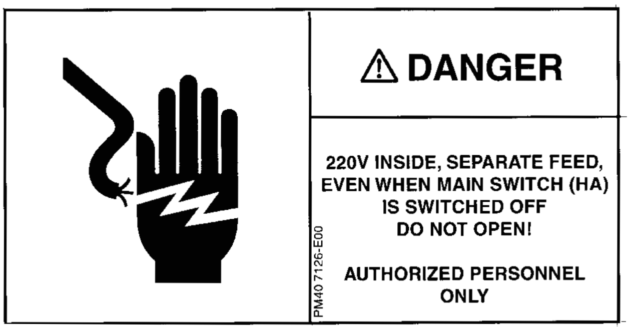

Fig. 23. PM40 7126-E00

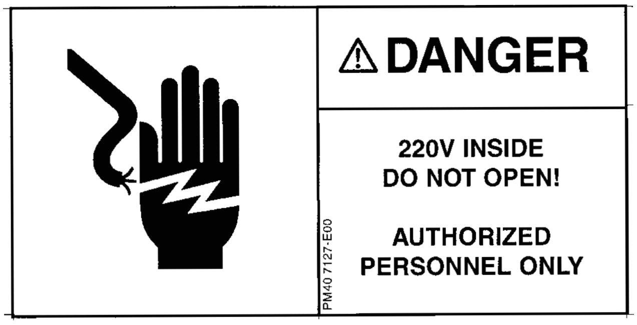

Fig. 24. PM40 7127-E00

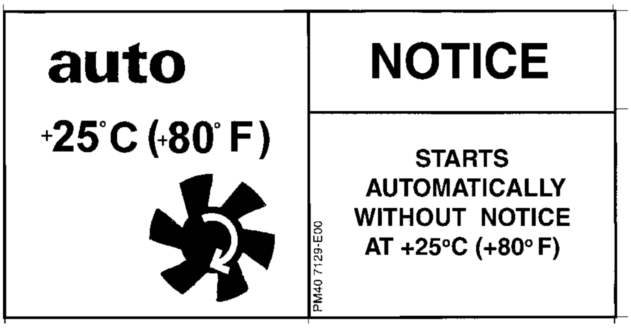

Fig. 25. PM40 7129-E00

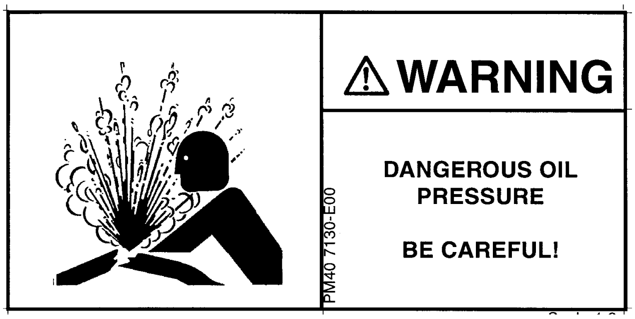

Fig. 26. PM40 7130-E00

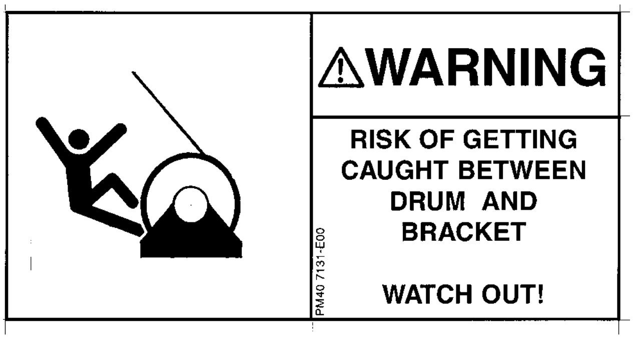

Fig. 27. PM40 7131-E00

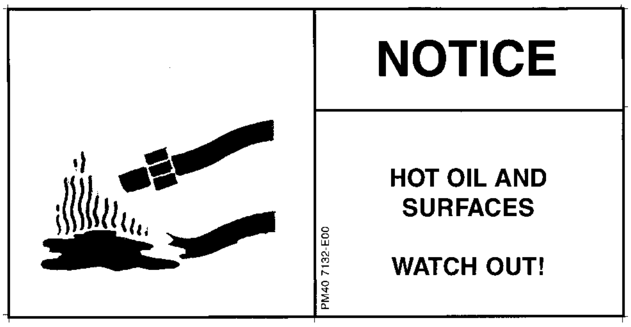

Fig. 28. PM40 7132-E00

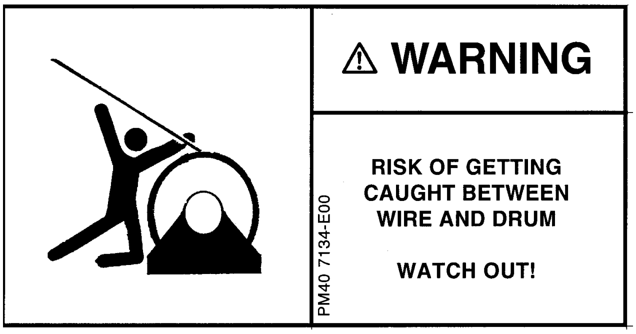

Fig. 29. PM40 7134-E00

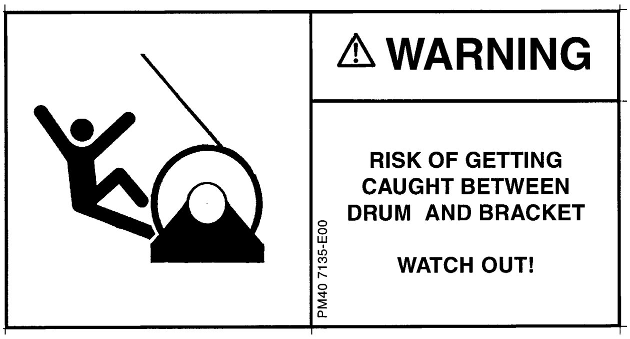

Fig. 30. PM40 7135-E00

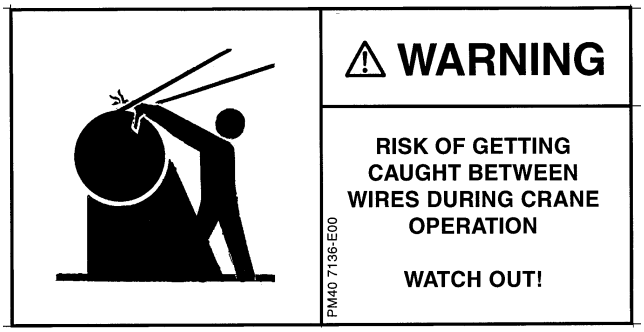

Fig. 31. PM40 7136-E00

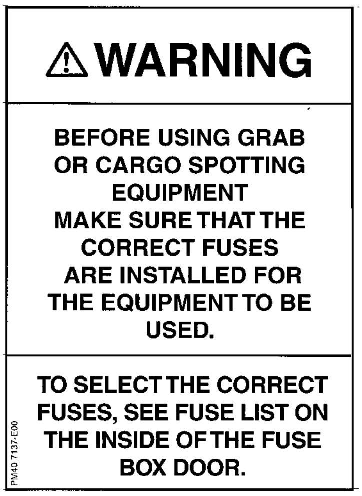

Fig. 32. PM40 7137-E00

Preface

This instruction manual covers the MacGREGOR-HÄGGLUNDS hydraulic deck crane. We hope that it will prove a valuable companion to everyone concerned with the operation of the crane or otherwise in a responsible position.

Every manual belongs to a specific crane or piece of equipment. The identification can be found on the label on the manual's back.

The manual contains nine sections. The various sections cover the following subjects:

General

Contents list; Safety; Conversion tables; Hydraulic and electric symbols.

Technical description

Technical data; description of crane design.

Function

Electric and hydraulic functions of the crane.

Operation

Operating instructions; crane operator's checklist.

Maintenance

Lubricating chart and maintenance instructions.

Service

General service instructions for the hydraulic system, mechanical, control system and electrical equipment.

Extra equipment

Extra equipment, if any; e.g. cargo handling equipment.

Appendices

Other information, which cannot be referred to any of the above mentioned sections.

Spare parts

General advice how to order spare parts; spare parts lists and illustrations.

General Instructions
Cleanliness in Hydraulic Systems

General

The fundamental and foremost rule in all work with hydraulic systems and components is absolute cleanliness.

Many components, e.g. valves and pumps, have very narrow tolerances, therefore microscopic impurities, invisible to the human eye, may have disastrous effect on the function of a hydraulic system.

The hydraulic system installation contains a cleaner with a cartridge designed to stop impurities produced within the hydraulic system during operation. Every care must be taken to prevent external impurities from entering the system when servicing or repairing it.

This also applies for components that have been removed and or to be reinstalled. Sooner or later, a component that has been replaced will be inspected in a test equipment somewhere. It is important that the component stays in the same condition as it was when it was removed, as it may otherwise be impossible to decide the real cause of a malfunction. It may also be possible that a component sent in for inspection is not defective at all, and will be returned.

Rules when working on the hydraulic system

The following fast rules shall be observed during working on the hydraulic system:

1. Carry out a rough general clean-up.
2. Protect the area where work shall be done. Use a sheet of plastic material or similar.
3. Clean well with white spirit or a similar solvent. Remember that it is not enough to clean only the parts immediately concerned, but also their surroundings from where accumulated dirt may be transferred in to the repair area. Do not forget to clean the tools to be used.
   Use a brush for cleaning, wipe off with a non-linting rag, repeat the cleaning if necessary. Finish by spraying the area of operations - hose connections etc. - with the solvent.
4. Mount protective covers immediately after removing a component, a pipe or a hose. Components, shall also be fitted with covers and plugs in order to prevent ingress of dirt.
   These covers must be kept in clean and closed packages. After use, covers are to be cleaned in the workshop.
5. Observe minute cleanliness when handling all types of quick-release connectors. Spray both connectors with solvent before making up a connection. Clean all covers before reinstallation.

Storing and filling up of hydraulic oil

1. Store barrels, hoses and pump as clean as possible and best of all in a warm room. The oil viscosity will then be considerably lower, which make the filling through filter easier.
2. Barrels, hoses and pump must be carefully handled and cleaned when necessary.
3. Store barrels horizontally, so plugs (or cocks) are below the liquid level and impurities (e.g., damped air) does not penetrate the barrels.
4. Observe minute cleanliness when filling up oil - carelessness is one of the greatest sources when impurities penetrates the hydraulic system.
5. Fill up hydraulic oil. See separate instruction.
6. Never use the last drop in the barrel, as it often consists of water and other impurities dependent on how many times the barrel has been opened.

Conversion Factors

Length

1 mm = 0,0393701 in (tum)
1 m = 3,28084 ft (fot)
1 in = 25,4 mm
1 ft (fot) = 304,8 mm

Volume

1 dm^3 = 1 litre = 0,219969 gallon (UK)
1 dm^3 = 1 litre = 0,264172 gallon (US)
1 gallon (UK)= 4,54609 dm^3
1 gallon (US)= 3,78541 dm^3

Mass

1 kg = 2,20463 lb
1 lb = 0,45359 kg
1 ton (metric) = 1000 kg
1 ton (UK) = 1 longton (US)
1 ton (UK) = 1016,05 kg

Force

1 kp = 2,20462 lbf
1 kp = 9,80665 N
1 lbf = 4,44822 N
1 lbf = 0,453592 kp
1 N = 0,101972 kp
1 N = 0,224809 lbf

Moment of force

1 kpm= 9,80665 Nm
1 kpm= 7,23301 lbf · ft
1 Nm = 0,737562 lbf · ft
1 lbf · ft = 0,135582 daNm
1 lbf · ft = 1,35582 Nm
1 lbf · ft = 0,13825 kpm
1 daNm = 1,01972 kpm
1 daNm = 7,37562 lbf · ft

Moment of inertia

1 kg · m^2 = 3417,17 lb · in^2
1 lb · in^2 = 0,00029264 kg · m^2

Pressure

1 atm = 14,6959 lbf/in^2 (psi)
1 atm = 1,01325 bar
1 atm = 1,03323 at
1 bar = 0,986923 atm
1 at = 14,2233 lbf/in^2 (psi)
1 at = 0,980665 bar
10 lbf/in^2 = 0,689476 bar
10 lbf/in^2 = 0,70307 at
1 bar = 1,01972 at
1 bar = 14,5038 lbf/in^2 (psi)
1 kp/cm^2 = 14,2233 lbf/in^2
1 bar = 100 kPa = 0,1 MPa
1 Pa = 1 N/m^2
1 Pa = $10,2 \cdot 10^{-6}$ kp/cm^2
1 Pa = $0,145 \cdot 10^{-6}$ lbf/in^2
1 Pa = $7,5 \cdot 10^{-3}$ mm Hg
1 kp/cm^2 = $98,07 \cdot 10^3$ Pa

Power

1 kW = 1,35962 hk
1 kW = 1,34102 hp (UK, US)
1 hk = 0,986320 hp (UK, US)
1 hp (UK, US) = 1,01387 hk
1 hk = 0,735499 kW
1 hp (UK, US) = 0,745700 kW

Temperature

0°C = 32°F
0°C = 273,15 K
0°F = -17,7778°C
0°F = 255,3722 K
0 K = -273,15°C
0 K = -459,67°F

$$°C = \frac{1}{1,8}(°F - 32) \qquad °F = 1,8\,(K - 273,15) + 32$$

$$°F = °C \cdot 1,8 + 32 \qquad K = \frac{1}{1,8}(°F - 32) + 273,15$$

$$°C = K - 273,15$$

$$K = °C + 273,15$$

Hydrauliska och Elektriska Symboler

Hydraulic and Electric Symbols

Hydraulische und elektrische Bildzeichen

Denna samling omfattar en del av de symboler och portmärkningar som förekommer i MacGREGOR Cranes hydrauliska och elektriska scheman i allmänhet, uppdelad i tre avsnitt:

This section covers a part of the symbols and port markings commonly used in MacGREGOR Cranes hydraulic and electric circuits, divided into three parts:

Diese Sammlung umfaßt einen Teil der allgemeinen Bild- und Anschlußzeichen, die in MacGREGOR Cranes hydraulischen und elektrischen Schemas vorkommen, unterteilt in drei Gruppen:

1. Hydrauliska symboler, enkla.

   Hydraulic symbols, basic.

   Hydraulische Bildzeichen, einfach.

2. Hydrauliska symboler, sammansatta.

   Hydraulic symbols, composite.

   Hydraulische Bildzeichen, kombiniert.

3. Elektriska symboler.

   Electric symbols.

   Elektrische Schaltzeichen.

Hydraulsymboler och ventilkombinationer
Hydraulic symbols and valve assemblies
Hydraulische Bildzeichen und Ventil-Baugruppen

1. Hydraulsymboler
Hydraulic Symbols
Hydraulische Bildzeichen

Huvudflöde.
Working oil line.
Arbeitsdruckleitung.

Styrtrycksledning.
Pilot line.
Steuerdruckleitung.

Dräneringledning.
Drain line.
Leckölleitung .

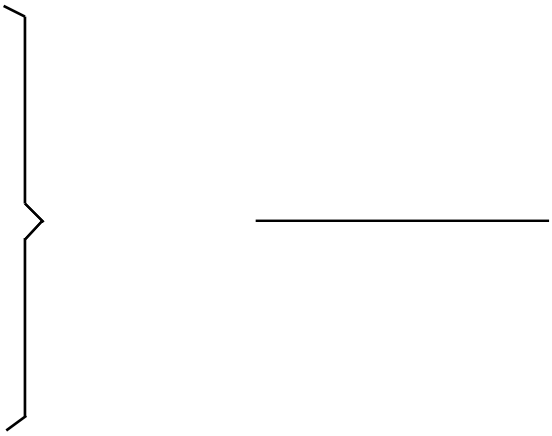

Propp, proppad anslutning (alternativa symboler).
Plug, plugged connection (alternative symbols).
Verschlußchraube, Sperrung (wahlweise).

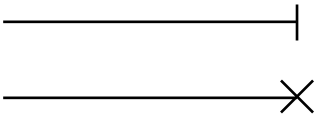

Fast strypning.
Fixed restriction.
Feste Blende, feste Drosselung.

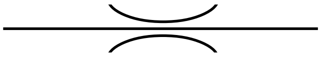

Variabel strypning.
Variable restriction.
Regelblende, regelbare Drosselung.

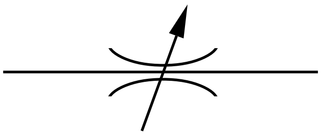

Viskositetsoberoende strypning.
Restriction unaffected by viscosity.
Temperaturunabhängige Drosselung.

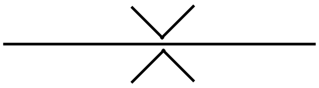

Slang.
Flexible hose.
Schlauch, biegsame Leitung.

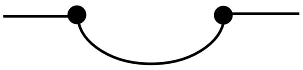

Ledningsförbindning.
Pipe junction.
Leitungsverbindung.

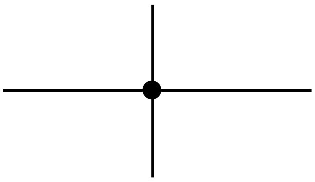

Korsande ledningar utan förbindning.
Crossed pipes, no connection.
Kreuzende Leitungen, ohne Verbindung.

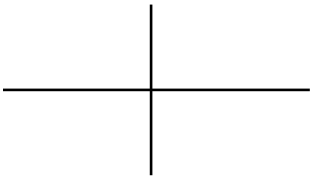

Vridbar anslutning.
Rotary connection.
Dreh-Verbindung.

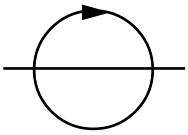

Tankledning, tank.
Tank line, tank.
Tankleitung, Tank.

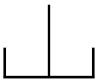

Styrbarhet eller inställbarhet.
Regulation or variability.
Regelbar oder nachstellbar.

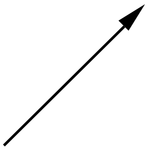

Gräns för flerkomponentenhet.
Enclosure of component assembly.
Umriß eines Mehrkomponentenblocks.

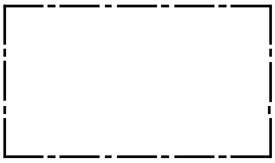

Mekanisk förbinding.
Mechanical connection.
Mechanische Verbindung.

Pump, fast deplacement, 1 flödesriktning.
Pump, fixed displacement, 1 direction of flow.
Pumpe, konstantes Deplacement, 1 Stromrichtung.

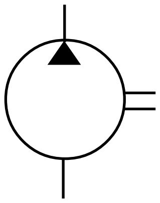

Pump, variabelt deplacement, 2 flödesriktningar.
Pump, variable displacement, 2 directions of flow.
Pumpe, variables Deplacement, 2 Stromrichtungen.

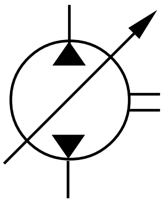

Motor, fast deplacement, 2 flödesriktningar, extern dränering.
Motor, fixed displacement, 2 directions of flow, external drain.
Motor, konstantes Deplacement, 2 Stromrichtungen, extern Drainierung.

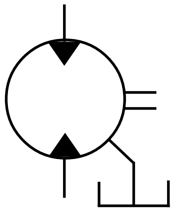

Motor, 2 deplacement, 2 flödesriktningar.
Motor, 2 displacement, 2 directions of flow.
Motor, 2 Deplacement, 2 Stromrichtungen.

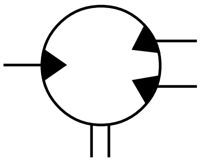

Motor, variabelt deplacement, 2 flödesriktningar.
Motor, variable displacement, 2 directions of flow.
Motor, variables Deplacement, 2 Stromrichtungen.

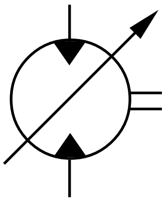

Elmotor.
Electric motor
Elektromotor.

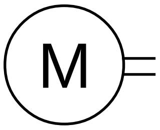

Filter.
Filter.
Filter.

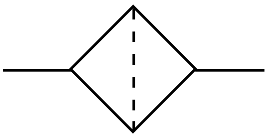

Filter med indikator.
Filter with contamination indicator.
Filter mit Anzeiger.

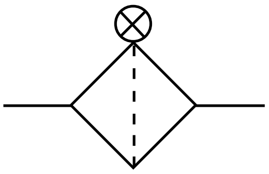

Filter med backventil.
Filter with by-pass valve.
Filter mit Rückschlagventil.

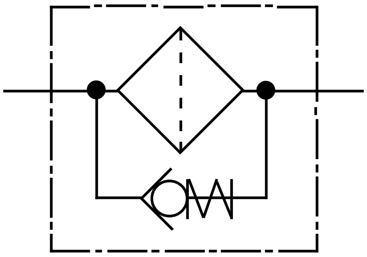

Luftfilter.
Air breather.
Luftreiniger.

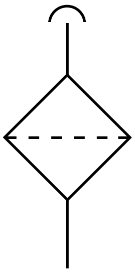

Tryckströmställare, normalt öppen.
Pressure electric switch, normally open.
Druckschalter, druckgesteuert, normal offen.

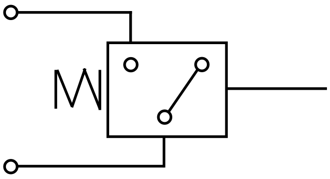

Tryckströmställare, normalt stängd, variabel inställning.
Pressure electric switch, normally closed, adjustable.
Druckschalter, druckgesteuert, normal geschlossen, regelbar.

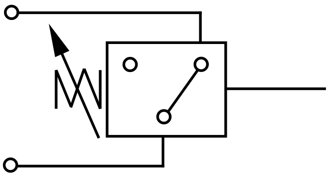

Backventil.
Non-return valve.
Rückschlagventil.

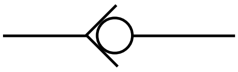

Backventil, fjäderförspännd med viss öppnings-tryckskillnad.
Non-return valve, spring loaded with certain opening pressure difference.
Rückschlagventil, federvorbelastet mit gewisser Öffnungsdruckdifferenz.

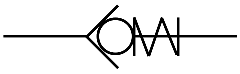

Backventil, fjäderförspännd med viss stängnings-tryckskillnad.
Non-return valve, spring loaded with certain closing pressure difference.
Rückschlagventil, federvorbelastet mit gewisser Schließungsdruckdifferenz.

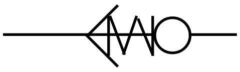

Snabbkoppling eller mätanslutning.
Quick coupling or gauge connection.
Schnellkupplung oder Meßanschluß.

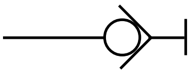

Mätanslutning.
Gauge connection.
Meßanschluß.

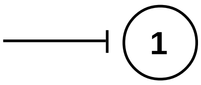

Avluftningsventil.
Ventilation valve.
Entlüftungsventil.

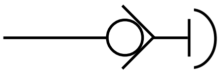

Backventil med variabel strypning.
Non-return valve with variable restriction.
Drosselrückschlagventil, regelbar.

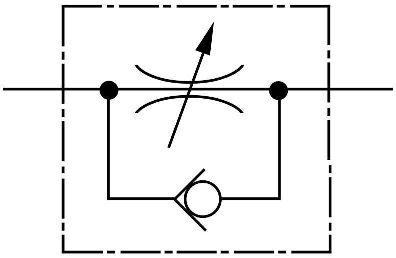

Växelventil.
Shuttle valve.
Wechselventil.

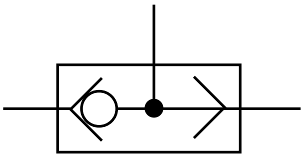

Bromscylinder.
Brake cylinder.
Bremszylinder.

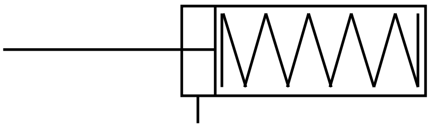

Cylinder, dubbelverkande med fast dämpning i ena läget.
Cylinder, double-acting with fixed cushion at one side.
Zylinder, doppelt wirkend mit konstanter Dämpfung auf einer Seite.

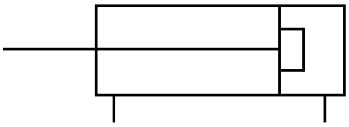

Manometer.
Pressure gauge.
Manometer.

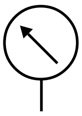

Termometer.
Thermometer.
Thermometer.

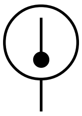

Oljekylare.
Oli cooler.
Ölkühler.

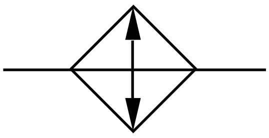

Ackumulator.
Accumulator.
Druckspeicher.

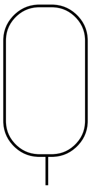

Avstängningsventil.
Shut-off valve.
Absperrventil.

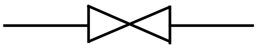

Ventil, grundsymbol.
Valve, basic symbol.
Schieber, Grundzeichen.

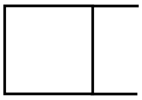

Elektromagnetiskt styrd ventil.
Electrically controlled valve.
Schieber, elektromagnetisch gesteuert.

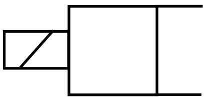

Tryckstyrd ventil.
Pressure controlled valve.
Schieber, druckgesteuert.

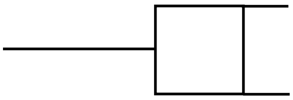

Pilotstyrd ventil, internt styrtryck.
Pilot controlled valve, internal pressure supply.
Schieber, vorgesteuert, interner Steuerdruck.

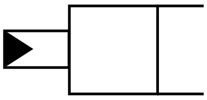

Pilotstyrd ventil, externt styrtryck.
Pilot controlled valve, external pressure supply.
Schieber, vorgesteuert, externer Steuerdruck.

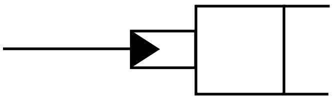

Spakstyrd ventil.
Lever controlled valve.
Schieber, knüppelgesteuert.

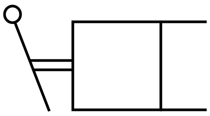

Mekaniskt styrd ventil.
Mechanically controlled valve.
Schieber, mechanisch gesteuert.

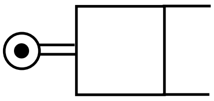

Ventil med fjäderretur.
Valve with spring return.
Schieber mit Feder-Rückführung.

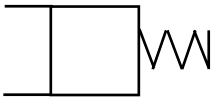

Ventil med lägeshållare.
Valve with detent.
Schieber mit Lagehalter.

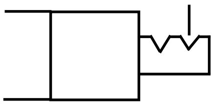

4/2-riktningsventil

Elstyrd fyrvägs tvålägesventil, med fjäderretur till neutralläge.

4/2 direction valve

Electrically controlled four-way, two-position valve, spring return to neutral position.

4/2-Wegeventil

4-Wegeschieber mit 2 Arbeitsstellungen, Feder-Rückführung in die Nullstellung.

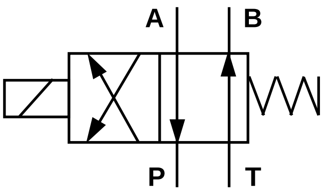

4/3-riktningsventil

Elstyrd fyrvägs trelägesventil med fjädercentrering i neutralläge.

4/3 direction valve

Electrically controlled four-way, three-position valve, spring centred.

4/3-Wegeventil

4-Wegeschieber mit 3 Arbeitsstellungen, feder-zentriert.

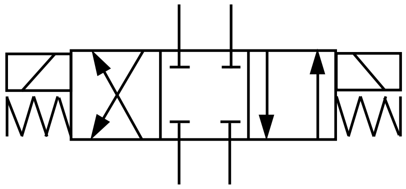

Proportionalventil.
Proportional valve.
Proportionalventil.

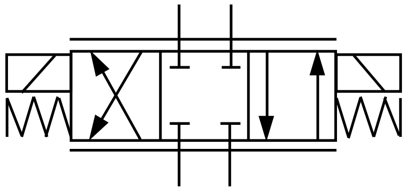

Servoventil.
Servo valve.
Servoventil.

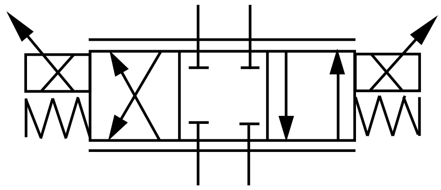

Balansventil (GP- och HH-kranar)
Balance valve (GP- and HH-cranes)
Ausgleichsventil(GP- und HH-Kräne)

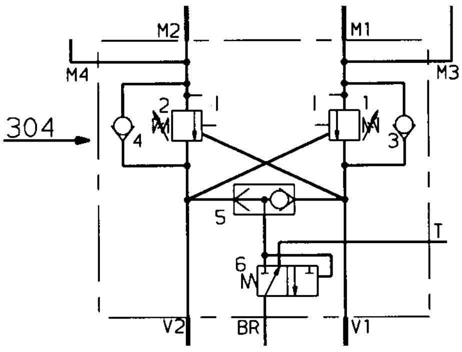

Volymströmsventil, variabel inställning.
Flow regulator, adjustable.
Fluß Regulator, regelbar.

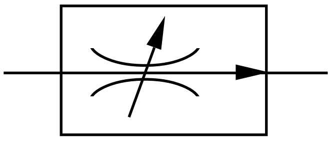

Volymströmsventil med backventil.
Flow regulator with non-return valve.
Fluß Regulator mit Rückschlagventil.

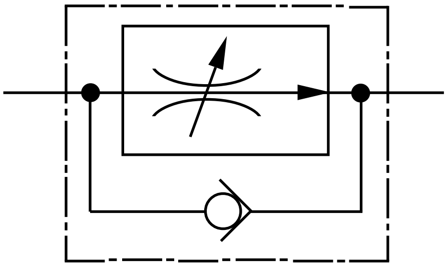

Patronventil.
Cartridge valve.
Patronenventil.

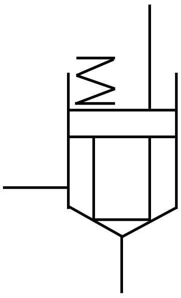

Tryckbegränsningsventil, variabel inställning.
Pressure limiting valve, adjustable.
Druckbegrenzungsventil, regelbar.

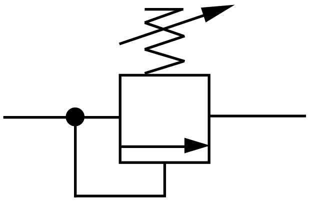

Tryckbegränsningsventil med utifrån styrd öppning eller stängning.
Pressure limiting valve with remote opening or closing.
Druckbegrenzungsventil mit ferngesteuerter Öffnung oder Schließung.

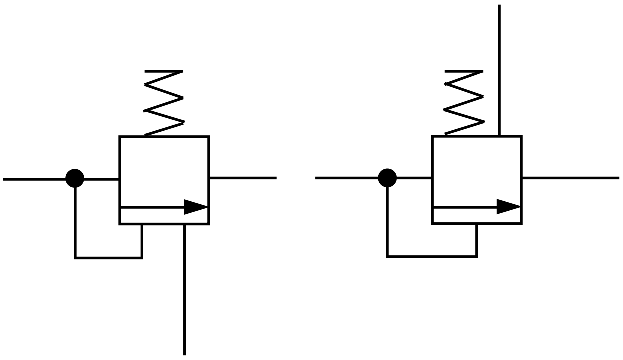

Tryckreduceringsventil.
Pressure reducing valve.
Druckreduzierventil.

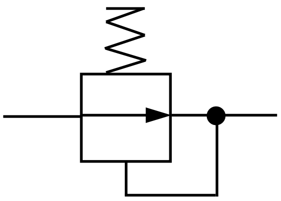

Tryckreduceringsventil med T-port.
Pressure reducing valve with T-port.
Druckreduzierventil mit T-Anschluß.

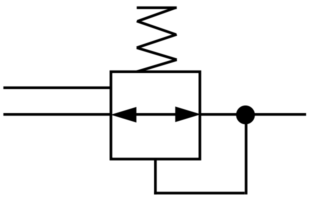

2. Ventilkombinationer
Valve Assemblies
Ventil-Baugruppen

Filterenhet med by-pass-ventil.
Filter unit with by-pass valve.
Filtereinheit mit Shuntventil.

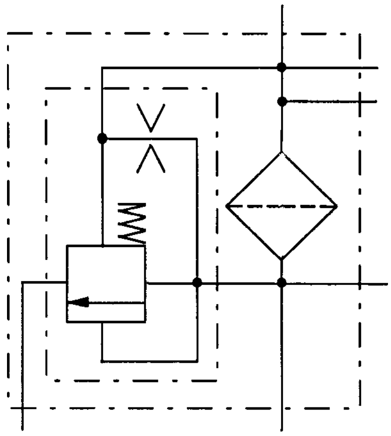

Filterenhet med by-pass-ventil och elektrisk filter-indikering.
Filter unit with by-pass valve and electric filter indication.
Filtereinheit mit Shuntventil und Zustandsanzeige.

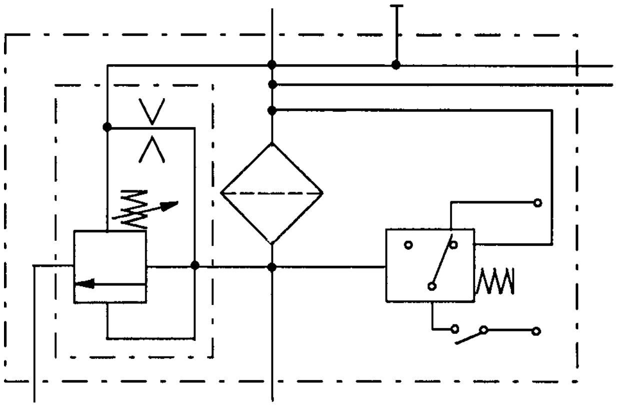

Filterenhet med tryckbegränsningsventil.
Filter unit with pressure limiting valve.
Filtereinheit mit Druckbegrenzungsventil.

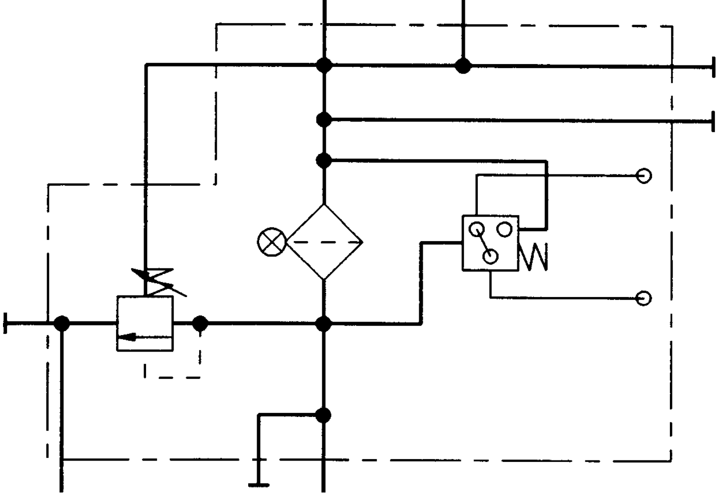

Filterenhet med tryckbegränsningsventil.
Filter unit with pressure limiting valve.
Filtereinheit mit Druckbegrenzungsventil.

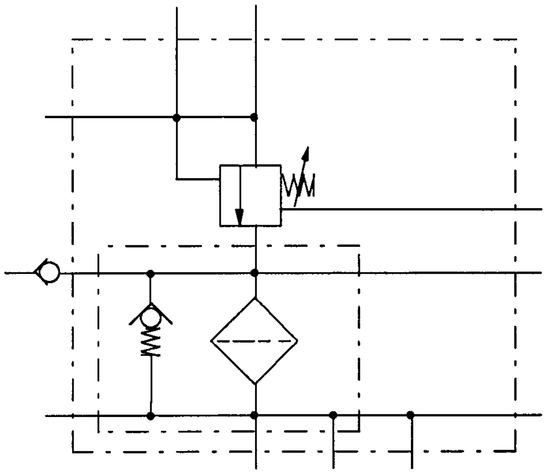

Filterenhet med by-pass-ventil och elektrisk filter-indikering.
Filter unit with by-pass valve and electric filter indication.
Filtereinheit mit Shuntventil und Zustandsanzeige.

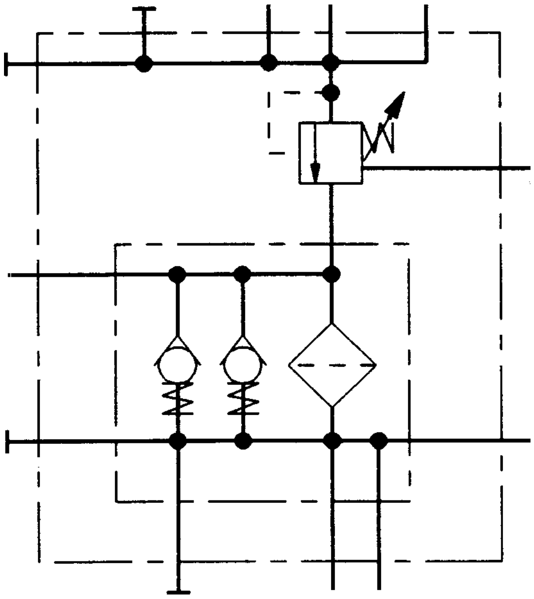

Pump för lastvinsch.
Pump for hoisting winch.
Pumpe für Lastwinde.

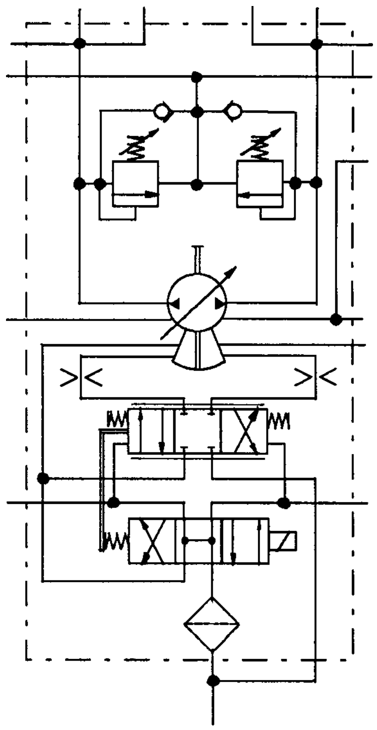

Pump för lastvinsch.
Pump for hoisting winch.
Pumpe für Lastwinde.

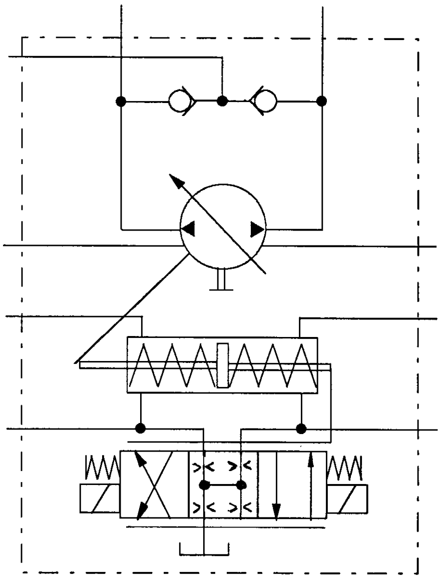

Pump för lastvinsch.
Pump for hoisting winch.
Pumpe für Lastwinde.

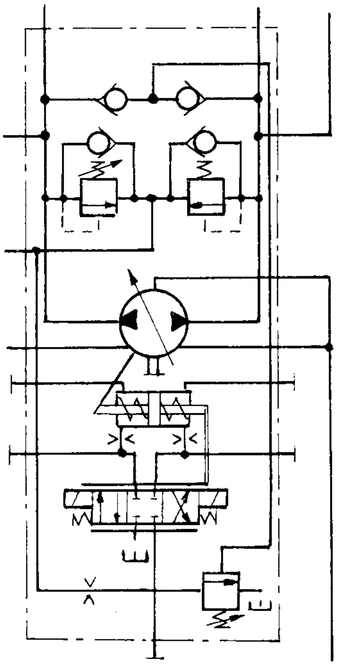

Pump för toppningsvinsch.
Pump for luffing winch.
Pumpe für Einziehwinde.

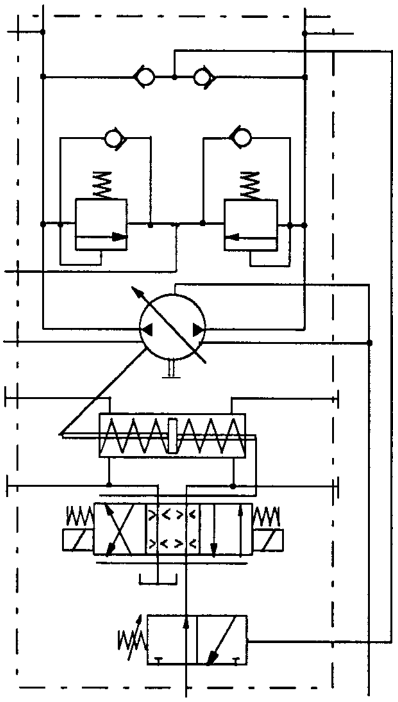

Pump för toppningsvinsch.
Pump for luffing winch.
Pumpe für Einziehwinde.

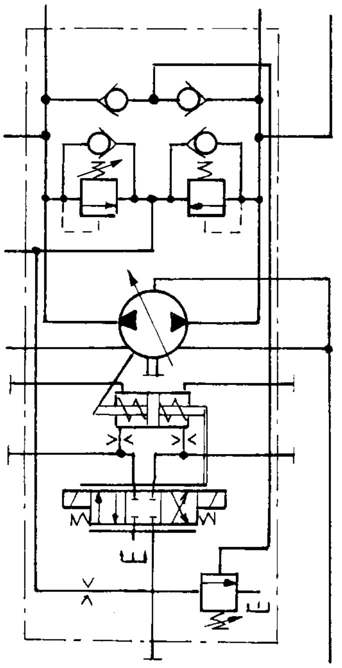

Pump för toppning eller vridning.
Pump for luffing or slewing.
Pumpe für Einziehwinde oder Schwenkantrieb.

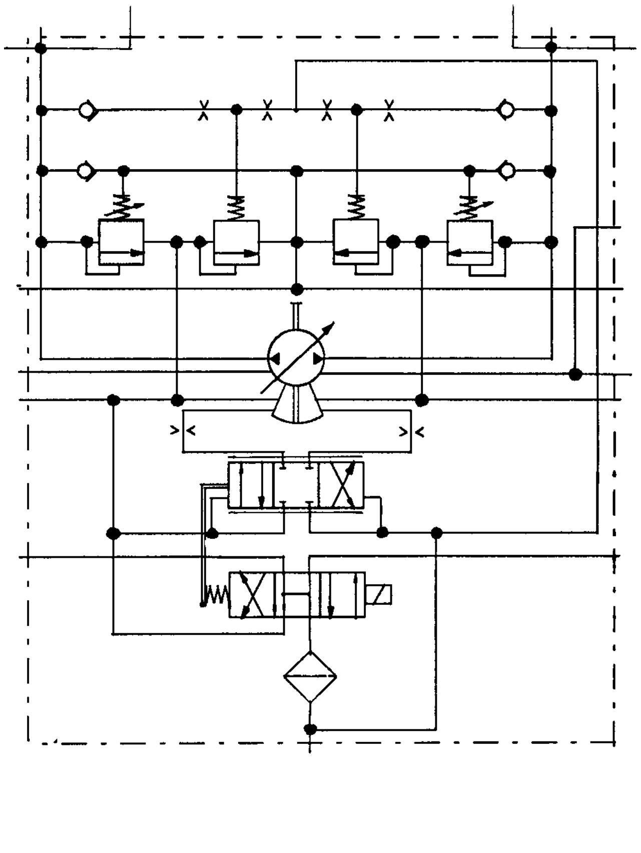

Pump för vridning eller toppning.
Pump for slewing or luffing.
Pumpe für Schwenkantrieb oder Einziehwinde.

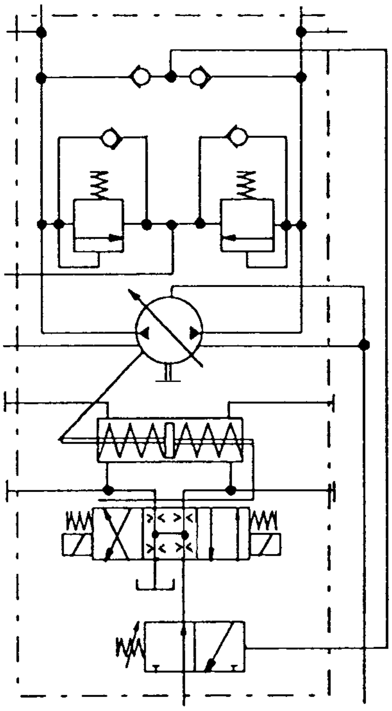

Pump för vridning.
Pump for slewing.
Pumpe für Schwenkantrieb.

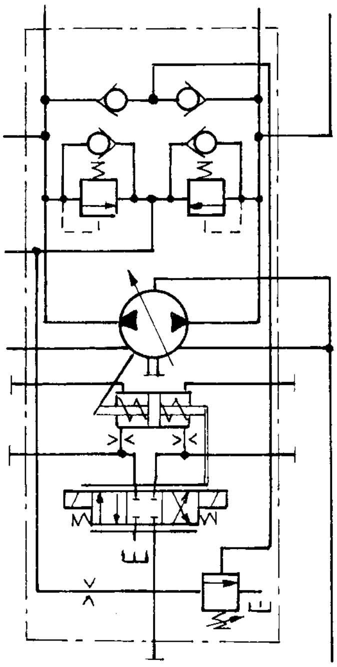

Lastvinsch med 84-seriens motor och motorventil.
Hoisting winch with 84-series motor and motor valve.
Lastwinde mit Motor Typ 84 und Motor-Umschaltventil.

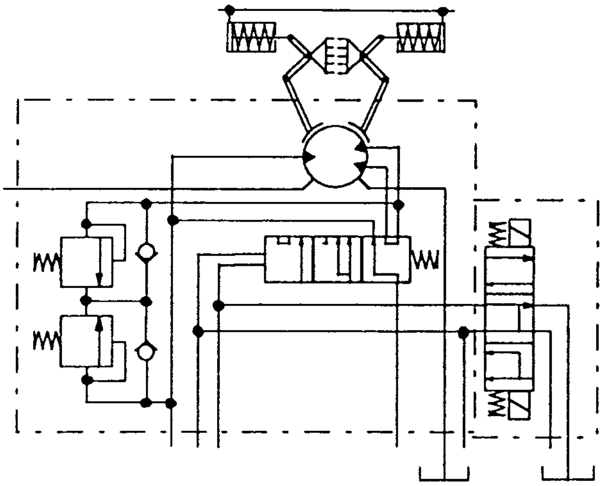

Lastvinsch med 63/64-seriens motor och motorventil.
Hoisting winch with 63/64-series motor and motor valve.
Lastwinde mit Motor Typ 63/64 und Motor-Umschalt-ventil.

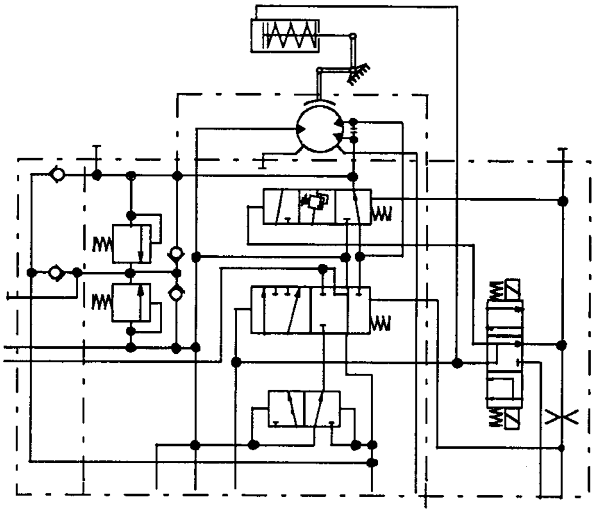

Lastvinsch med variabel högvarvig hydraulmotor.
Hoisting winch with variable high speed hydraulic motor.
Lastwinde mit variabel schnellaufendem hydraulischem Motor.

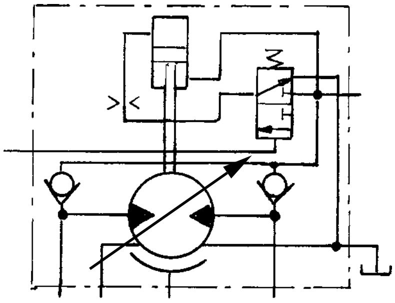

Toppningsvinsch med 63/64-seriens motor och motorventil.
Luffing winch with 63/64-series motor and motor valve.
Einziehwinde mit Motor Typ 63/64 und Motor-Umschaltventil.

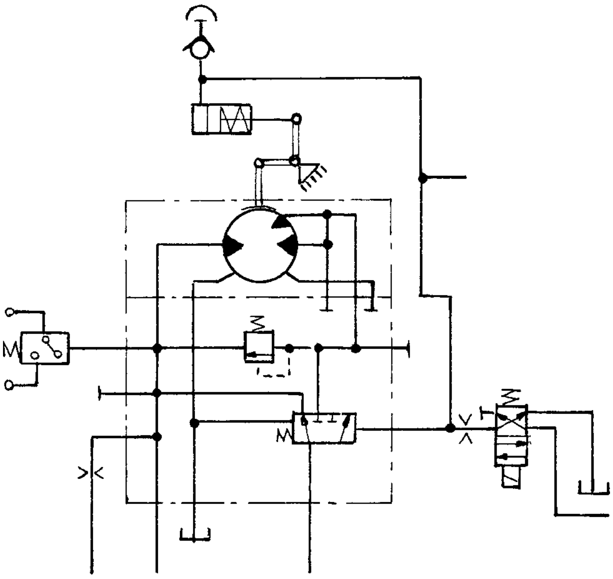

Avlastnings- och spolventil, vridning.
Unloading and flushing valve, slewing.
Entlastungs- und Spülventil, Schwenkantrieb.

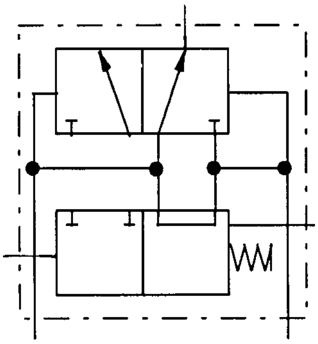

Avlastnings- och spolventil, lastvinsch och vridning.
Unloading and flushing unit, hoisting and slewing.
Entlastungs- und Spülventil, Lastwinde und Schwenk-antrieb.

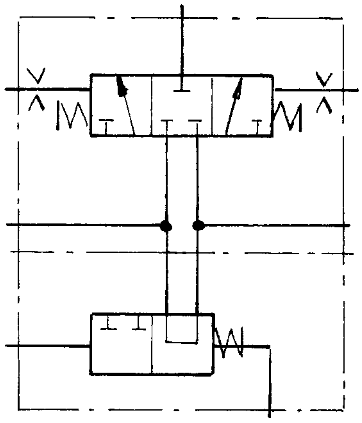

Spolventil.
Flushing valve.
Spülventil.

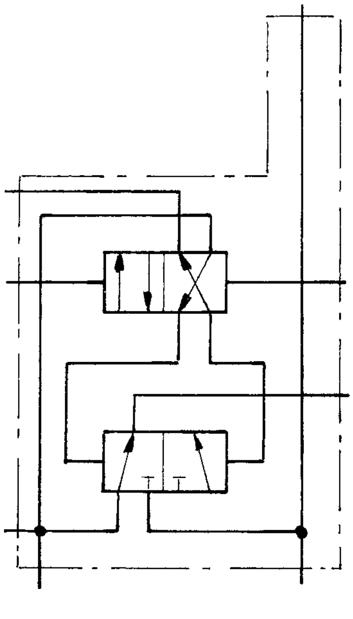

Ventilenhet för stabiliseringsvinsch.
Valve unit for stabilizing winch.
Ventileinheit für Stabilisierungswinde.

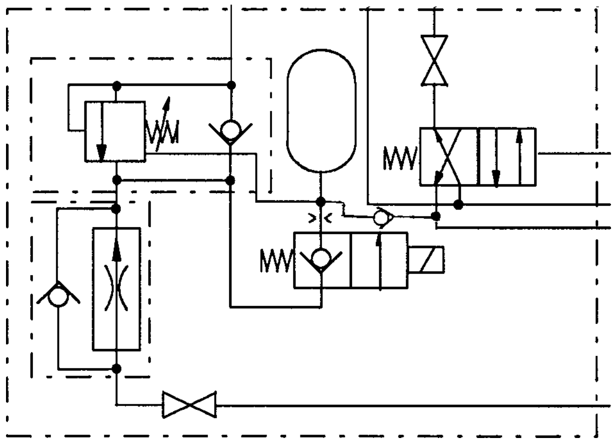

Avlastningsenhet för matarpump.
Unloading unit for feed pump.
Entlastungsventil für Speisepumpe.

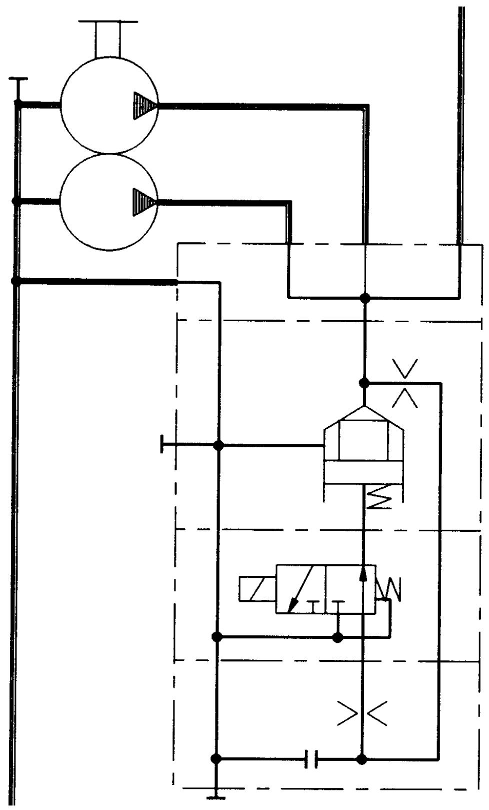

Ventilenhet, cylindertoppning.
Valve unit, cylinder luffing.
Ventileinheit, Zylinder-Einziehung.

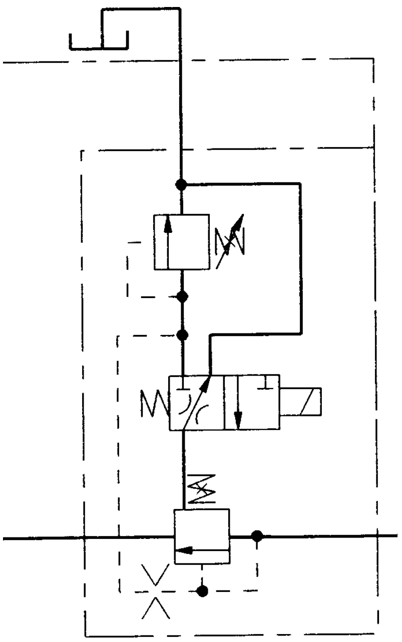

Ventilenhet, cylindertoppning.
Valve unit, cylinder luffing.
Ventileinheit, Zylinder-Einziehung.

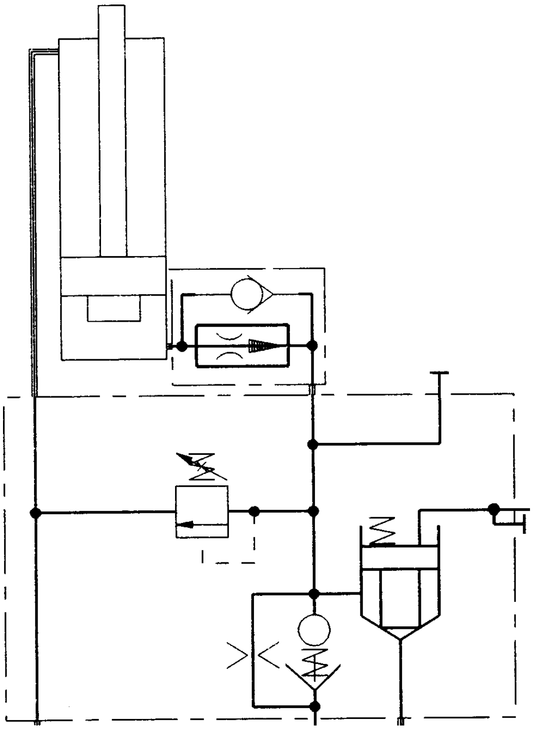

3. Elektriska symboler
Electric Symbols
Elektrische Schaltzeichen

Ledningsförbindning, fast.
Conductor junction, permanent.
Leitungsverbindung, fest.

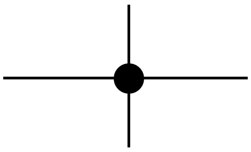

Ledningsförbindning, löstagbar.
Conductor junction, separable.
Leitungsverbindung, trennbar.

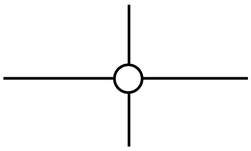

Korsande ledningar, utan förbindning.
Crossed conductor, no connection.
Kreuzende Leitungen, ohne Verbindung.

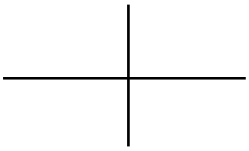

Släpring med borste.
Slip ring with brush.
Schleifring mit Bürste.

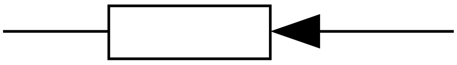

Säkring, matarsida markerad.
Fuse, supply side indicated.
Sicherung, Zuführungsseite markiert.

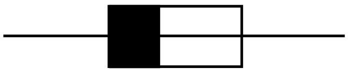

Relä, kontaktor.
Magnetic relay, contactor.
Steuergerät, elektromagnetische Erregerwicklung.

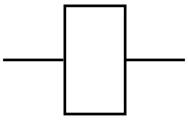

Slutkontakt.
Make contact.
Arbeitskontakt.

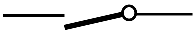

Brytkontakt.
Break contact.
Schaltkontakt.

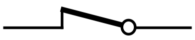

Huvudkontakt.
Main contact.
Hauptkontakt.

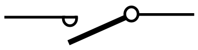

Kontakt, manuellt påverkad.
Contact, manually operated.
Kontakt, von Hand betätigt.

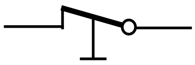

Kontakt, manuellt påverkad utan automatisk återgång.
Contact, manually operated without automatic return.
Kontakt, von Hand betätigt, nicht selbstrückstellend.

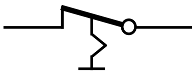
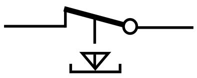

Nödstopp.
Emergency stop.
Notschalter.

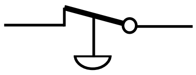
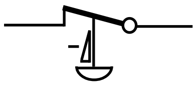

Kontakt med fördröjt frånslag.
Contact with delayd opening.
Kontakt mit Ausschalt-Verzögerung.

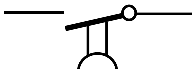

Kontakt med fördröjt tillslag.
Contact with delayed closing.
Kontakt mit Einschalt-Verzögerung.

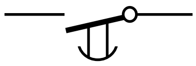

Kontakt med fördröjt till- och frånslag.
Contact with delayed opening/closing.
Kontakt mit Ein- und Ausschalt-Verzögerung.

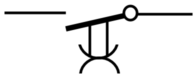

Gränslägesvakt.
Limit switch.
Endschalter.

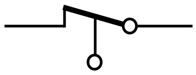

Kontakt, termiskt styrd.
Contact, thermal controlled.
Kontakt, thermisch gesteuert.

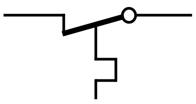

Pressostat.
Pressostat.
Pressostat.

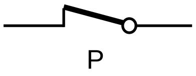

Nivåvakt.
Level switch.
Ölstandswächter.

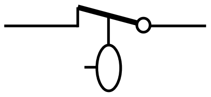

Termostat.
Temperature switch, thermostat.
Temperaturwächter, Thermostat.

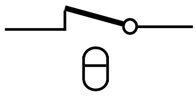

Beröringsfri givare.
Touch free contact.
Berührungsfreier Geber.

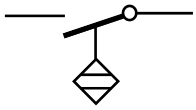

Induktiv givare.
Inductive contact.
Induktiver Geber.

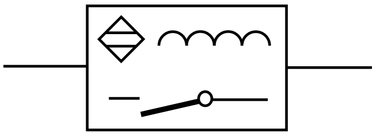

Amperemeter.
Ammeter.
Strommesser.

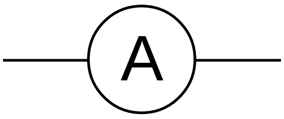

Voltmeter.
Voltmeter.
Voltmesser.

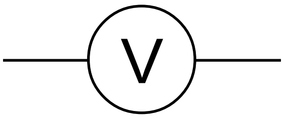

Timmätare.
Hour counter.
Stundenzähler.

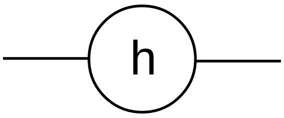

Jordanslutning (skrov).
Earth (hull) connection.
Massenverbindung (Rumpf).

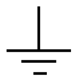

Likriktare.
Rectifier.
Gleichrichter.

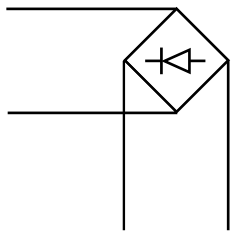

Transformator med två lindningar.
Transformer with two windings.
Transformator mit zwei Wicklungen.

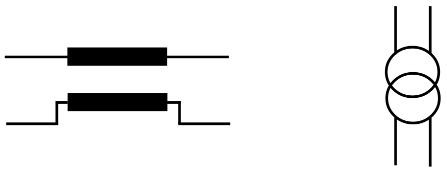

Transformator med flera sekundärlindningar, med skärm mellan primär- och sekundärlindningar.
Transformer with several secondary windings, with screen between primary and secondary windings.
Transformator mit mehreren Sekundärwicklungen, mit Schirm zwischen den Primär- und Sekundär-Wicklungen.

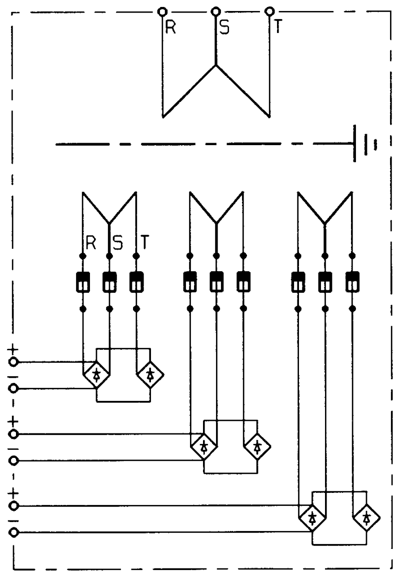

Trepoligt termiskt relä (överströmsskydd).
Triple-pole thermal relay (overcurrent) circuit breaker.
Dreipoliges thermisches Relais (Überstrom-Auslösung).

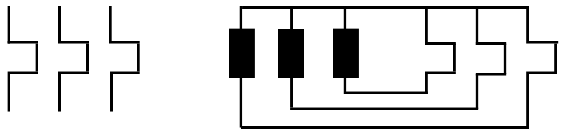

Diod.
Diode.
Diode.

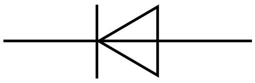

Lysdiod.
Light emitting diode.
Leuchtdiode.

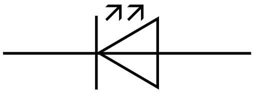

Signallampa.
Pilot lamp.
Meldeleuchte.

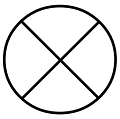

Belysning.
Light.
Beleuchtung.

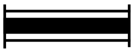

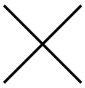

Strålkastare.
Floodlight.
Scheinwerfer.

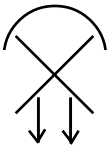

Alarmsignal.
Alarm signal.
Alarmsignal.

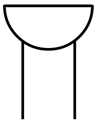

Alarmsirén.
Alarmsiren.
Alarmhupe.

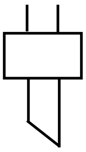

Alarmklocka.
Alarm bell.
Alarmglocke.

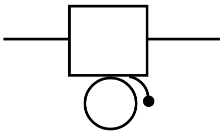

Enfas hylsdon.
Single-phase outlet.
Steckdose, einphasig.

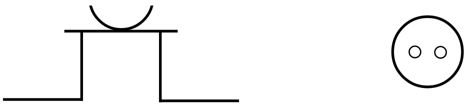

Lägesgivare, vinkelgivare.
Position encoder.
Drehfeldgeber.

Puls-/hastighetsgivare
Puls/speed encoder
Geschwindigkeitsgeber

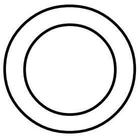

Skarvkontakt.
Flexible lead connector.
Steckverbindung.

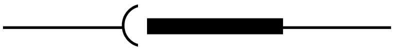

Motstånd.
Resistor.
Widerstand.

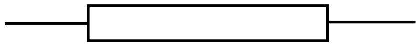

Motstånd, spänningsberoende.
Resistor, non-linear.
Widerstand, spannungsabhängig.

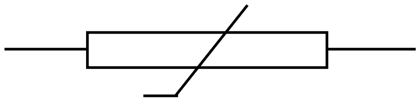

Variabelt motstånd.
Variable resistor.
Widerstand, regelbar.

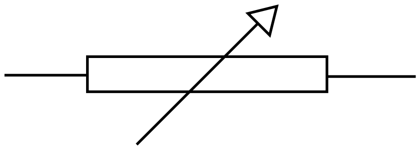

Potentiometer med fast och en rörlig kontakt.
Potentiometer with slider and a fixed tap.
Potentiometer mit Schleifer und einer Festanzapfung.

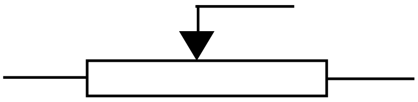

Värmare.
Heater.
Heizkörper.

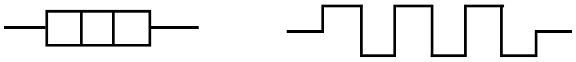

Motor.
Motor.
Motor.

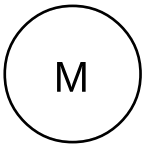

Kondensator.
Condenser.
Kondensator.

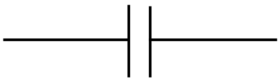

Värmare med fläkt.
Heater with fan.
Heizkörper mit Lüfter.

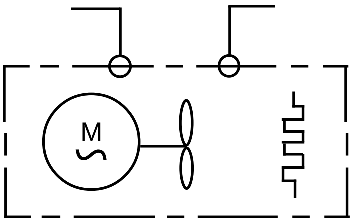

Luftkonditionering.
Air condition.
Klimaanlageapparat.

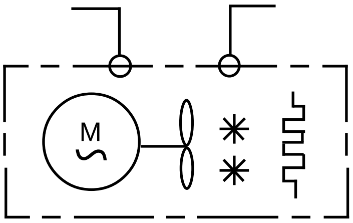

Magnetventil.
Solenoid valve.
Magnetventil.

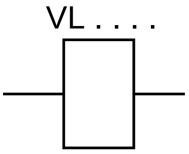

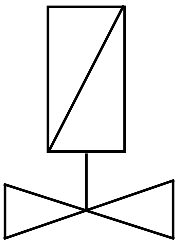

Körspak, lastvinch.
Controller, hoisting.
Steuerknüppel, Lastwinde.

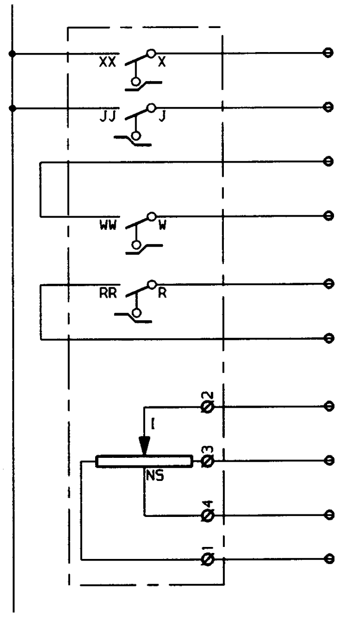

Körspak, toppning och vridning.
Controller, luffing and slewing.
Steuerknüppel, Einziehwinde und Schwenkantrieb.

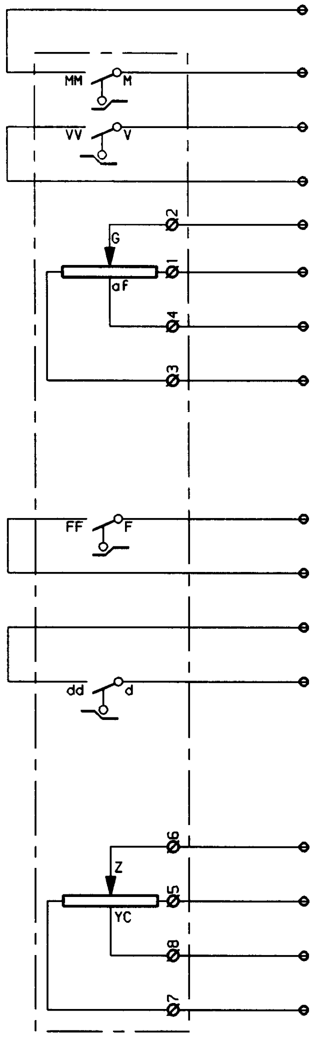

TECHNICAL DATA

GENERAL

Type of crane	GL 4028-2		
Work order	KR97038		
Serial number	62504309 - CRANE NO 1<br>62504310 - CRANE NO 2<br>62504311 - CRANE NO 3		
Weight		50	ton
Type of current		3 ~ 60	Hz
Supply voltage		440	V
Control voltage		230	V

MECHANICAL DATA

Jib radius		max	28.0	m
Jib radius		min	3.4	m
Jib radius, high hook limit		min	27	m
Lifting height		H	9	m
Lifting height		K	32	m
Hoisting capacity, single crane		max	40	ton
Hoisting capacity, single crane at jib radius 28.0 m		max	40	ton
Hoisting capacity, single crane at jib radius 3.4 m		max	40	ton
Hoisting speed at Low	0 - 40 ton		0 - 22	m/min
Hoisting speed at High	0 - 16 ton		0 - 44	m/min
Luffing time from max to min jib radius, single crane			60	sec.
Slewing speed, single crane		max	0.7	rev/min

ELECTRICAL DATA

MAIN ELECTRICAL MOTOR	No 388 6313-801	
Type of current	3 ~ 60	Hz
Rated power, cont.	150	kW
Rated power, S6 15% int.	225	kW
Speed	1785	rev/min
Rated voltage	440	V
Starting current	500	A

GEAR BOX DATA

GEAR BOX	No 388 5166-801	
Gear ratio	i = 0.692	
Speed	2580	rev/min

FEED AND CONTROL CIRCUIT

ELECTRICAL MOTOR	No 388 0369-801	
Type of current	3 ~ 60	Hz
Rated power, 2-pole	20	kW
Rated power, 4-pole	13	kW
Speed, 2-pole	3360	rev/min
Speed, 4-pole	1680	rev/min
Starting current, 2-pole	141	A
Starting current, 4-pole	107	A
FEED PUMP	No 388 1669-801	
Displacement	49.1	cm^3/rev.

HYDRAULIC DATA - HOISTING CIRCUIT

PUMP	No 287 9493-801	(2×)
Displacement	125	cm^3/rev.
MOTOR, HOISTING	No 178 2573-801	CA 420-400
Displacement	25130	cm^3/rev.
Torque theoretical	4000	Nm/MPa

HYDRAULIC DATA - LUFFING CIRCUIT

PUMP	No 287 9493-801	(1×)
Displacement	125	cm^3/rev.
MOTOR	No 178 1934-701	CA 140
Displacement	8800	cm^3/rev.
Torque theoretical	1400	Nm/MPa

HYDRAULIC DATA - SLEWING CIRCUIT

PUMP	No 287 9494-801	(1×)
Displacement	125	cm^3/rev.
MOTOR	No 388 3127-801	(3×)
Displacement	59.8	cm^3/rev.
Torque theoretical	9.5	Nm/MPa

WIRE ROPE

Wire rope, hoisting winch	Ø 34 mm x 270.5<br>No 388 9794-808	m
Wire rope, luffing winch	Ø 30 mm x 206<br>No 388 9795-807	m

OIL VOLUME / OIL FILLING

Oil tank	560	litres
Planetary gear box, module 16	16.0	litres
Axial thrust bearing, CA 100-CA 210	0.33	litres

Technical Description

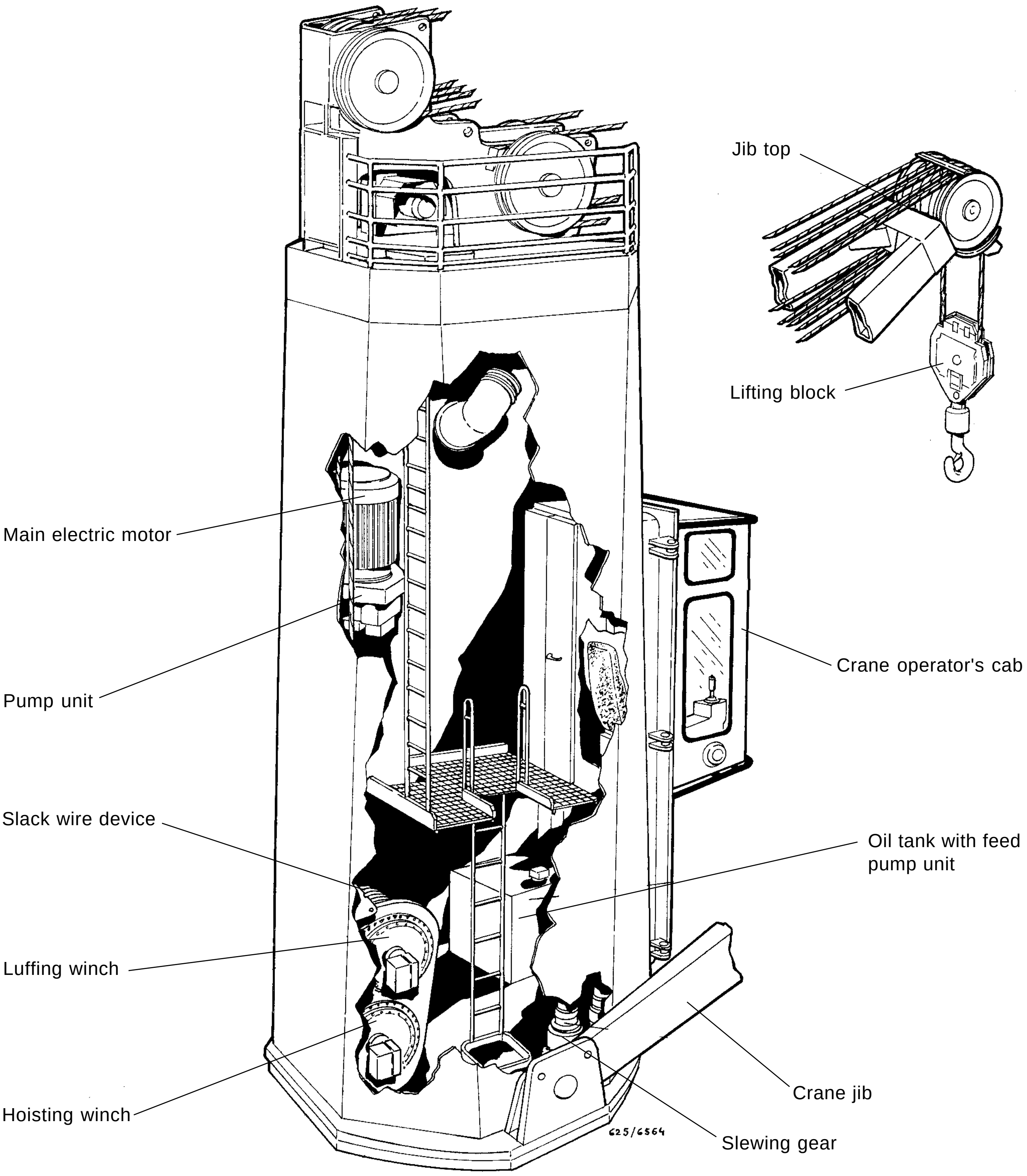

Fig. 1. Crane type GL-2

MacGREGOR hydraulic deck cranes are self-contained units with all machinery enclosed in the cranehouse and, thus, well protected against external abuse.

When delivered, the cranes are mechanically, hydraulically, and electrically complete in every respect and ready to be installed on a suitable foundation fixed in the ship's deck or on a crane carriage travelling on deck.

Construction

All crane movements - hoisting, luffing, and slewing - are driven by hydraulic motors. These are supplied with high pressure oil by a pump unit comprising separate pumps for each hydraulic circuit. The high pressure pumps are mounted in the same pump unit and are geardriven by a flange mounted electric motor.

The hoisting winch is driven by one or two hydraulic motors. A single-acting brake operates on the winch. When the winch is running, the brake is automatically released by a hydraulic unit. In order to prevent complete un- and over-winding of the drum, the winch is furnished with limit switches connected directly to the drum. A slack rope guard prevents slack wire rope.

Raising and lowering of the jib are done by means of a luffing winch, the design of which is similar in principle to that used for the hoisting movement.

The slewing drive of the crane uses a high-speed hydraulic motor, installed onto a planetary gearbox and fitted with a drive pinion. The pinion meshes with the internal gear teeth of the slewing ring which supports the cranehouse. On customer's order limit switches can be provided to define the slewing range of the crane.

To heat the oil to a suitable operating temperature, the separately driven feed pump of screw type generates the heat by circulating the oil in the system.

An oil cooler removes heat developed in the oil during its work cycle. If the oil temperature exceeds a certain level, a thermostat operates a contactor to stop the main drive electric motor.

Various pressure limits are provided to protect the crane against overload. All moving parts in the hydraulic system are lubricated by the hydraulic oil which minimises mechanical wear and ensures the added advantage of silent operation.

The cranehouse is an all-welded structure on a rigid baseplate, and encloses all mechanical and hydraulical machinery and associated electric equipment for adequate protection against rain, sleet, and waves breaking over the ship. Catwalks, ladders, platforms, and handrails facilitate maintenance of the crane.

The tops of the cranehouse and of the jib head are fitted with sheaves for the hoisting and luffing wire ropes. All sheaves run on sealed ball bearings, and their shafts are secured by lockplates.

Various limit switches are provided.

The cranes are supplied with test certificates to the requirements of National Swedish Board of Shipping and Navigation. The design meets the requirements of ILO and the relevant classification societies whose certificates will be furnished if specially ordered.

Crane operator's cab

The crane operator's cab affords an excellent allround view of the entire work area from the comfortable seat, and ready access to the two control levers, one controlling the hoisting winch, and the other, the luffing and slewing movements.

The cab also contains control panels with various switches, control buttons, and signal lamps.

Jib

For most crane types the jib is made of two box girders, joined by transverse tubular steel struts. The foot of the jib mounts on two trunnion journals at the sides of the cranehouse skirt. A mercury-lamp or sometimes Halogen floodlight is also mounted on the jib.

Electric equipment

Certain electric cables outside of the cranehouse, e.g. on the jib, are carried in steel conduit. All cables and electric accessories are specifically designed for marine use.

The slip-ring unit linking the crane network with the shipboard network, is in single cranes placed in the foundation and at the bottom centre of the twin platform in twin cranes, and is bolted to the bottom of the crane.

The contactor cabinets are placed on the inside wall of the cranehouse. The cabinets contain contactors, relays, fuses, and various other components of the electric installation. The main power switch is placed on the A cabinet.

Hydraulic hoses

The hydraulic hoses are high-pressure tested and carefully cleaned and inspected before assembly.

Cargo handling gear

The swivel, hook, shackles, rings, and chains all have ample safety margins for the maximum load to be handled by the crane. The non-rotatory, langs lay wire ropes are galvanised and oiled.

Surface treatment

All surfaces of the crane are carefully prepared by sandblasting before being given a coat of Primer. As standard the crane is painted with an anticorrosive system basically based on two-component epoxy paint and an acrylic finishing coat.

Twin cranes

A MacGREGOR hydraulic twin crane set consists of two single cranes mounted on a commmon platform. The cranes may be operated independently, or interconnected for twin operation. When operated in single, they function exactly like ordinary single cranes.

For twin operation, all movements of the two cranes are synchronised through the use of transmitters and receivers. The slewing of the twin combination is driven by hydraulic motors in the twin platform.

Hydraulic Function

General

The following description refers to the standard hydraulic system of MacGREGOR hydraulic deck cranes, normal version.

Minor variations in the hydraulic system may occur in individual cranes of the same type, and the objective of this description is only to provide a general orientation concerning the arrangement of the hydraulic system.

A complete hydraulic circuit diagram relating specifically to your crane will be found in the instruction manual, section “Spare Parts”, Group 9.4.

Three separate hydraulic work circuits, ie, the hoisting, luffing, and slewing circuits, plus feed and control-pressure circuits, make up the complete crane operating system.

Each of these work circuits may have one or more pumps located in the pump unit, in Figure 1 marked PHO, PLU, PSL. Pump PF for the feed and control-pressure system is located inside the hydraulic reservoir.

Identifying circuits in the hydraulic diagram

Figure 1 shows the arrangement of the three work circuits, hoisting, luffing, and slewing circuits, together with the feed and control-pressure circuit. The circuits appear in the same order in the hydraulic diagram.

Symbols

Every hydraulic component is represented by a special symbol in the circuit diagram. The meaning of the symbols is explained in section “General” in the Manual. The symbol for the reservoir is shown in several places in the diagram for greater simplicity of drawing; but there is naturally only one reservoir in actual fact.

Component identification

The main components are identified by four-digit numbers in the diagram and on the actual components in the crane. The first digit of each number signifies the circuit to which the component belongs:

1... Hoisting circuit
2... Luffing circuit
3... Slewing circuit
4... Feed and control-pressure circuit
5... Additional programming equipment *)
6... Twin crane hydraulic equipment
7... Other additional hydraulic equipment *)

*) Equipment installed at request of customer

Legend

MHO	Hydraulic motor, hoisting circuit
MLU	Hydraulic motor, luffing circuit
MSL	Hydraulic motor, slewing circuit
PHO	Pump, hoisting circuit
PLU	Pump, luffing circuit
PSL	Pump, slewing circuit
PF	Pump, feed circuit

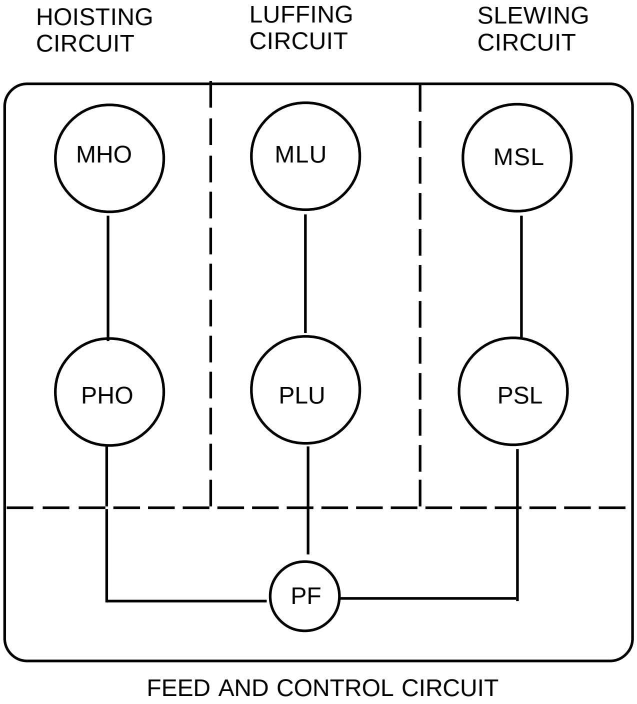

Fig. 1. Identifying hydraulic circuits

Feed and control circuits

Feed pressure circuit

The function of this circuit is mainly to replace the hot fluid leaking from the closed work circuits. The feed fluid is introduced through non-return valves into the low-pressure side of the system. The feed circuit operates at a pressure of 1.9-2.5 MPa (19-25 bar), which is set at valve 4148 and is measured at test point 1.

Control pressure circuit

The function of this circuit is to supply fluid to the brakes and pump servos, ie, to control winch speed, slewing speed and working direction. The system operates at 3.0-3.3 MPa (30-33 bar), and is measured at test point 2. Control fluid is provided to the pump servos via servo valves.

NOTE.

For exact pressure settings, see pressure list in the hydraulic diagram.

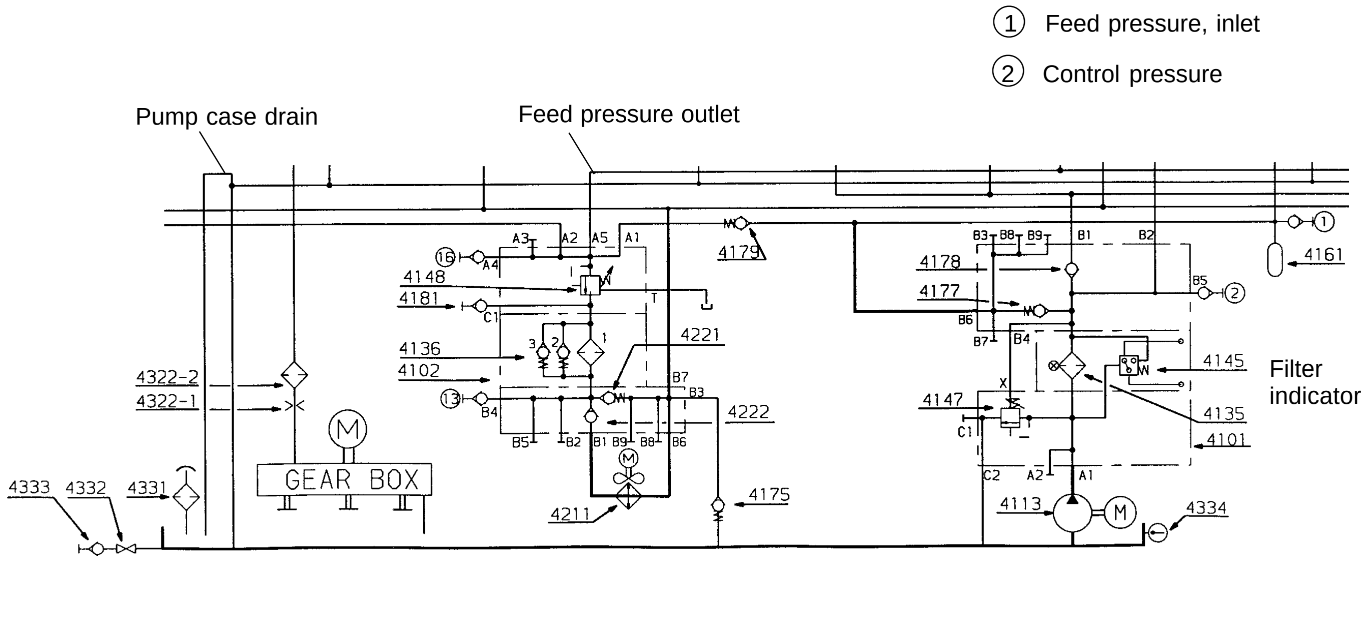

Fig. 2. Feed and control circuit

Hoisting winch circuit

Introduction

Figure 6 shows a hoisting winch circuit diagram, with the following main components:

Pumps	1111, 1112
Hydraulic motor	1141
Motor valve	1131
Brake cylinder	1211

Motor valve 1131 is mounted on the hydraulic motor. The brake is mounted to the winch drum on the opposite side of the motor.

Function

With the control lever in neutral, Figure 3, and the pump running, the hoisting circuit performs no actual work. A continuous supply of fluid flushes the circuit and leakage is compensated by fluid from the feed circuit.

Low-speed range, brake release

When the control lever is moved to the left for low-speed operation, Figure 4, valves 1221 and 1226 are actuated. Control pressure is thus admitted to the brake cylinder 1211 which release the brake, and also to valve 1128-1 which shifts from its unloading position.

With no load on the cargo hook and the control lever in brake release position, Figure 4, the hoisting winch should produce a slow hoisting movement, so called plussing. See instruction under section 6.1 "High pressure pumps".

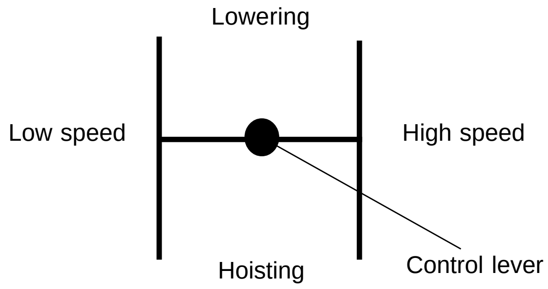

Fig. 3. Control lever in neutral, hoisting

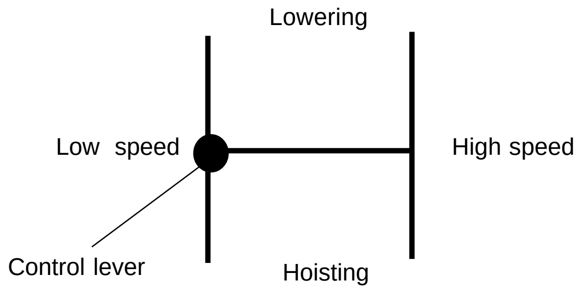

Fig. 4. Control lever, hoisting, brake release position

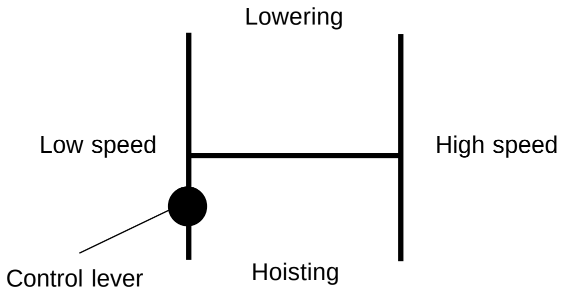

Fig. 5. Control lever, hoisting, low-speed hoisting

HOISTING CIRCUIT

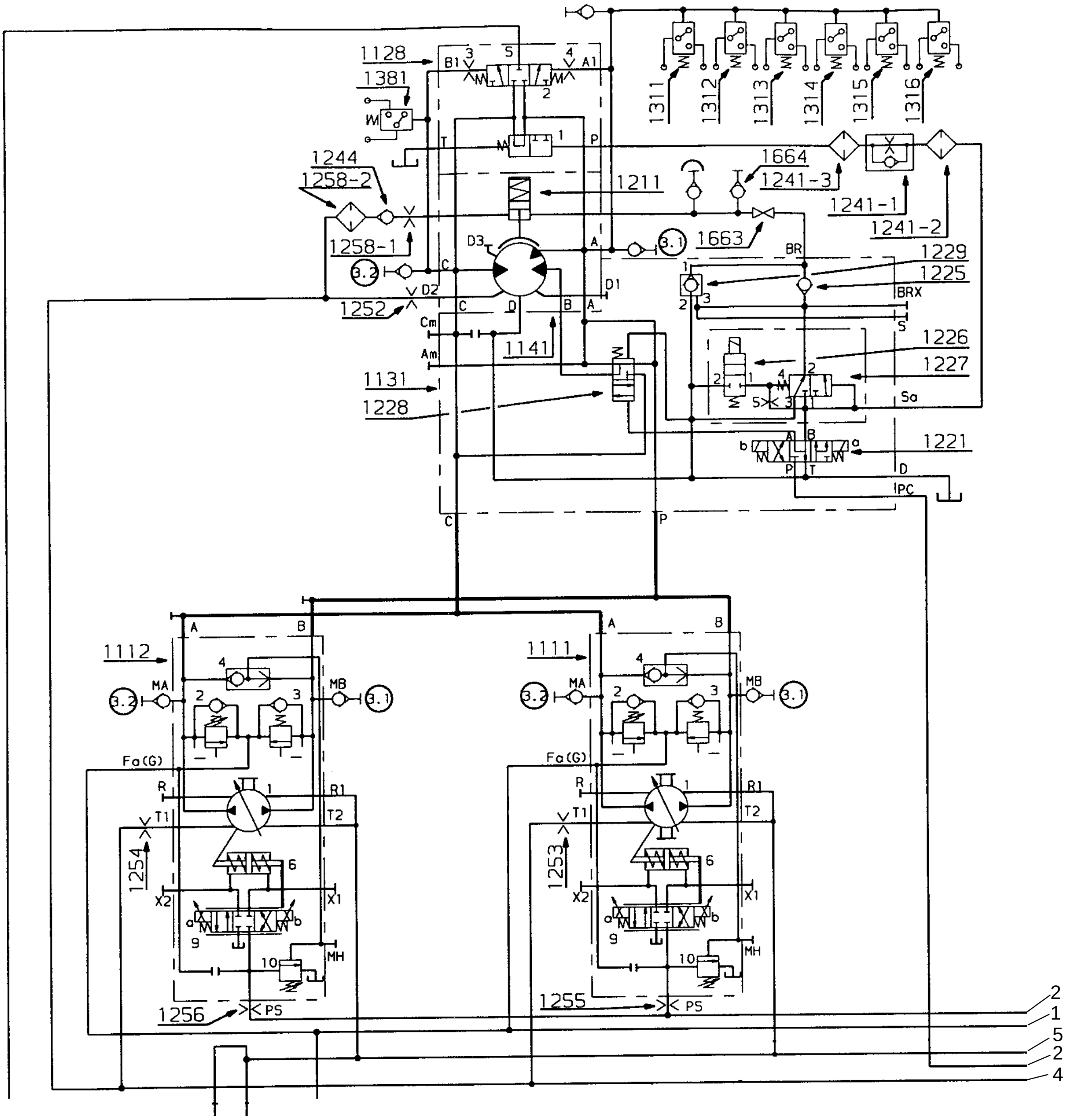

1 Feed pressure, inlet
2 Control pressure
4 Pump case flushing
5 Pump case drain

Fig. 6. Hoisting winch circuit

High speed range, brake release

When the control lever is moved to the right or left, Figures 4 and 8, valves 1221 and 1226 are actuated, (1226 slightly delayed in relation to 1221). Control pressure is connected to the brake valve 1226 which releases the brake, and also to valve 1128-1 which shifts from its relief mode.

With no load on the cargo hook and the control lever in brake release position, Figure 8, the hoisting winch should produce a slow hoisting movement.

High-speed range overload

If the load on the cargo hook is too heavy for handling in the high-speed range, the pressure switch 1311 will cause valve 1221 to shift to low-speed range. (Full displacement)

Hoisting and lowering

Pulling the control lever backwards from the brake release position will start the winch to lift the load, Figure 9. The servo valves 1111-9 and 1112-9 affect the servo and thus the pump displacement. The pumps 1111 and 1112 thereby produce a flow of fluid that is proportional to deflection of the control lever.

If, instead, the control lever is moved forward, the load will be lowered, ie, the function is the same as for hoisting, except for the direction of flow.

Safety functions

The hoisting winch circuit embodies three safety features:

- Main pressure limiting and shock absorbing valves 1111-2; 1111-3, 1112-2, 1112-3; 1111-10 and 1112-10
- Pressure switches 1311 and 1316 (overload valve) stops the crane in the event of overloading at low speed. Shifts to low-speed when running in the high-speed mode
- Pressure switch 1381 stops the crane in the event of insufficient feed pressure

Note! It is not allowed to adjust sealed safety valves without contacting MacGREGOR Cranes, Service Department.

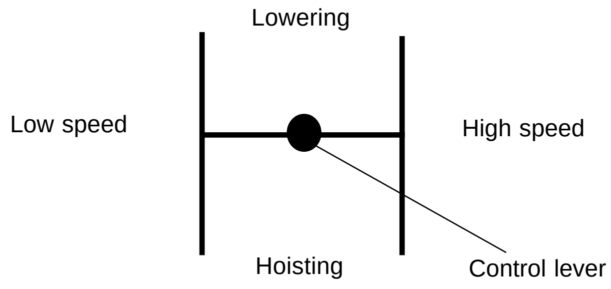

Fig. 7. Control lever in neutral, hoisting

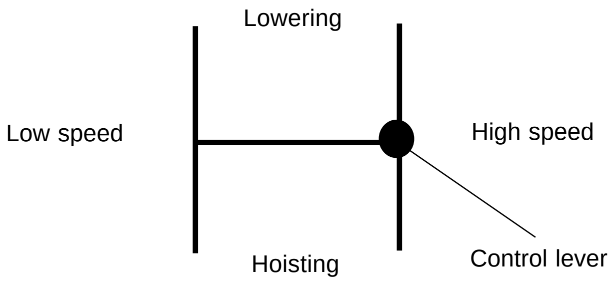

Fig. 8. Control lever, hoisting, brake release position

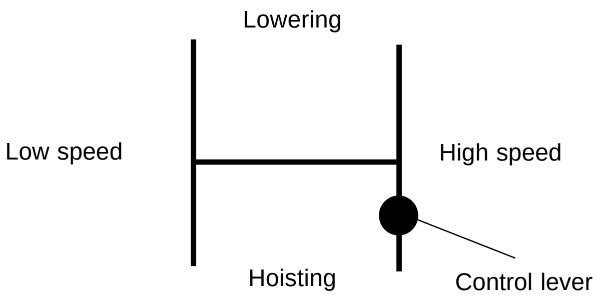

Fig. 9. Control lever, hoisting, high-speed hoisting

LUFFING CIRCUIT

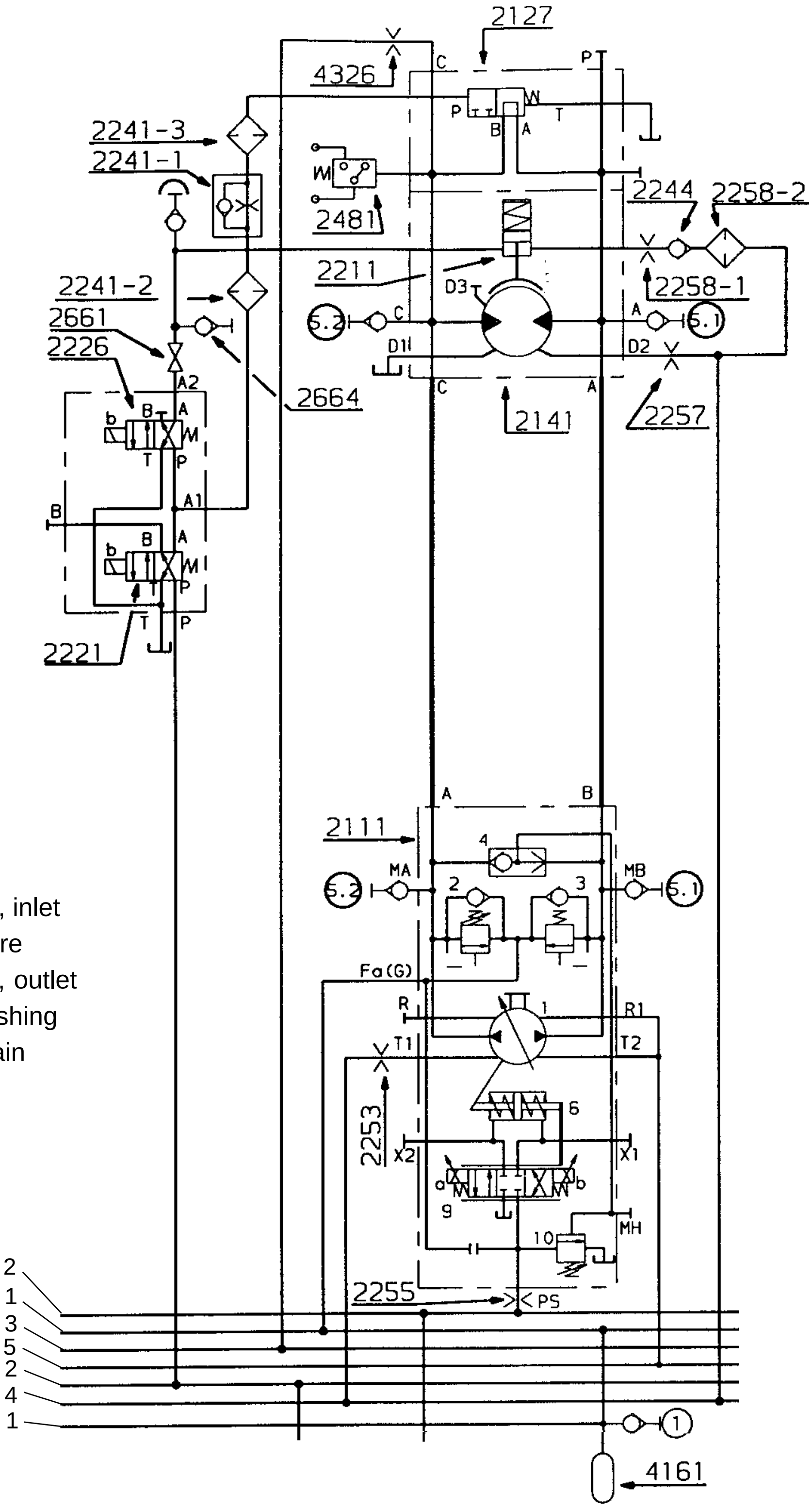

1 Feed pressure, inlet
2 Control pressure
3 Feed pressure, outlet
4 Pump case flushing
5 Pump case drain

Fig. 10. Luffing circuit

Luffing winch circuit

Introduction

Figure 10 shows a luffing winch circuit diagram, with the following main components:

Variable displacement pump	2111
Hydraulic motor	2141
Brake	2211

The luffing and slewing movements are generally operated by a common control lever, Figure 11.

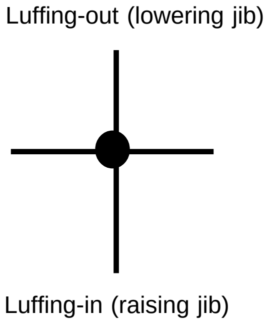

Fig. 11. Control lever, luffing/slewing

Brake release

Moving the control lever from neutral, eg, rearward to raise the jib, causes the valves 2221 and 2226 (2226 slightly delayed in relation to 2221) to shift position and control pressure is admitted to the brake 2211 and to valve 4326 which shifts position from the relief mode.

With the control lever in the brake release position, the luffing winch should produce a slow movement of the crane jib.

Raising and lowering the jib

Moving the control lever further backward starts operation of the luffing winch. The servo valve 2111-9 affects the servo and thus the pump displacement. The pump 2111 thereby produces a flow of fluid at a rate that is proportional to lever deflection.

To lower the jib, move the lever forward from neutral. The function is the same as for luffing-in, except for the direction of the fluid flow.

Safety functions

The luffing winch circuit embodies three safety features:

- Pressure limiting valves 2111-3, 2111-2
- Pressure switch 2481; stops the crane in the event of insufficient feed pressure
- Pressure cut-off valve 2111-10

SLEWING CIRCUIT

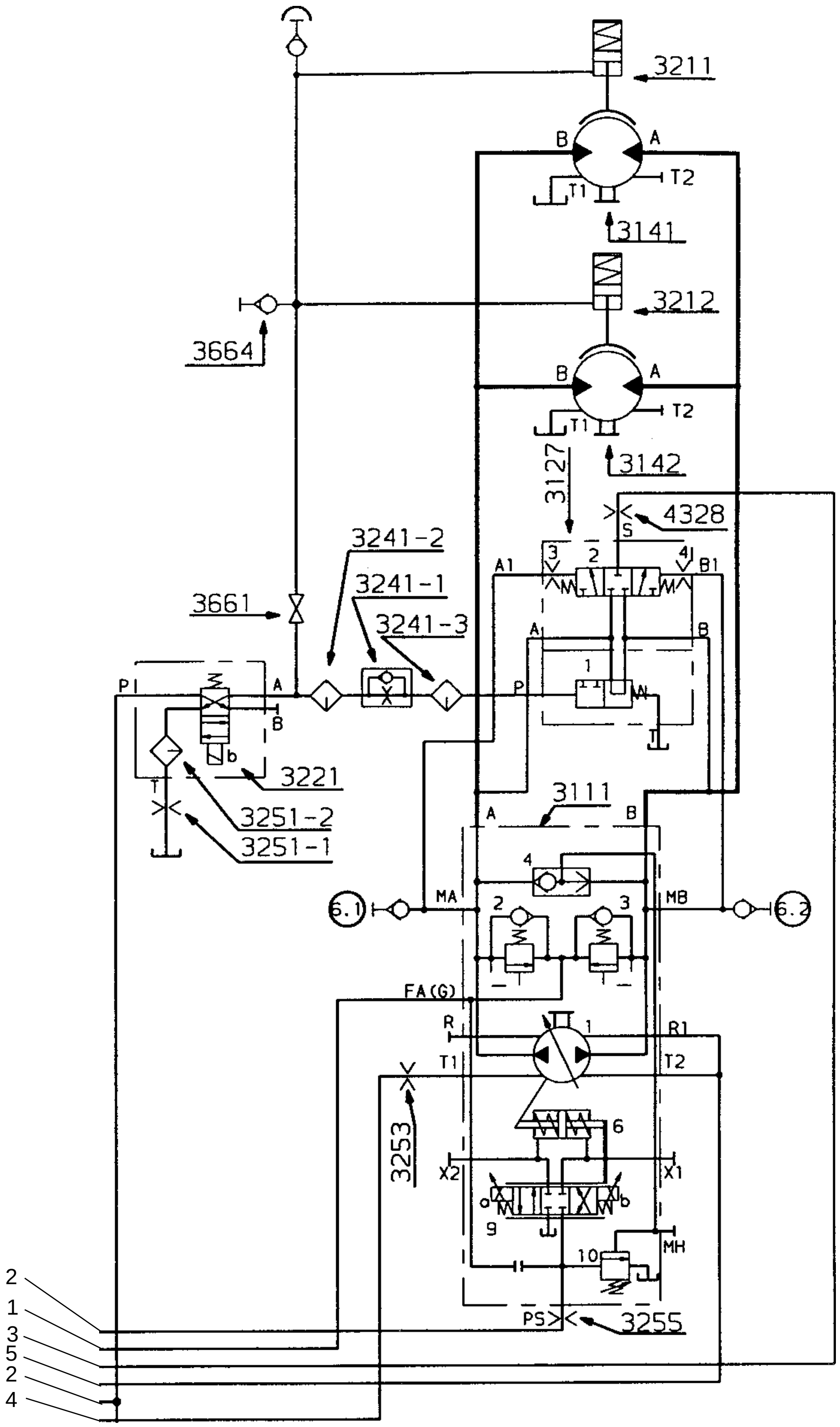

1 Feed pressure, inlet
2 Control pressure
3 Feed pressure, outlet
4 Pump case flushing
5 Pump case drain

Fig. 12. Slewing circuit

Slewing circuit

Introduction

Figure 13 shows a slewing circuit diagram for the crane, with the following main components:

Variable displacement pump	3111
Hydraulic motors	3141, 3142, 3143
Valve unit	3127
Brake cylinders	3211, 3212, 3213

The luffing and slewing movements are generally operated by a common control lever, Figure 13.

Brake release

Moving the control lever, eg, to the left for slewing left (anti-clockwise), will affect valve 3221. The brakes will be released and the unloading valve 3127-1 will be blocked.

Slewing left and right

By moving the control lever further to the side, the crane will start to slew in the direction chosen. The servo valve 3111-9 affects the servo and thus the pump displacement. The pump 3111 thereby produces a flow of fluid at a rate that is proportional to lever deflection.

To slew to the right (clockwise), move the lever to the right instead. The function is the same as for slewing left, except for the direction of the fluid flow.

Safety functions

The slewing circuit embodies two safety features:

- Main pressure limiting and pressure cut-off valves 3111-2, 3111-3 and 3111-10

Note! It is not allowed to adjust sealed safety valves without contacting MacGREGOR Cranes, Service Department.

Slewing mechanism (twin cranes only)

When the two cranes of a twin set are operated separately, their normal slewing mechanism is used.

When employed together as a twin crane, the slewing mechanism of the twin platform is connected to the No. 1 (Master) crane pump 3111.

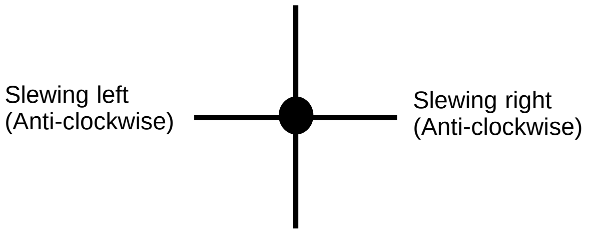

Fig. 13. Control lever, luffing/slewing

Starting and Stopping, Electric Safety Functions Single Cranes

Introduction

The following description refers to the starting and stopping, and electric safety functions, of a standard-type single crane. A complete electric circuit diagram relating specifically to the particular crane will be found in the section SPARE PARTS of the Manual.

The purpose of the present description is to furnish a general idea of the design and function of the electric system. The crane is supplied with electric power via the slip-ring unit and the main switch in the cranehouse. Cables lead from the main switch to: a transformer supplying power for all control circuits; a switch for the oil cooler fan motor; a Y/D switch for the main electric motor; a pole switch for the feed pump electric motor; and a thermostat switch for the ventilation fan in cranehouse.

All components are marked by symbols in the diagram, making it easier to follow the various stages of the starting sequence, e.g.

- Contactors: A1.K1 (N); A1.K3 (D); A1.K2 (Y)
- Contactors: A21; A22; A23
- Time relays: A1.K9; A27

Preparing for starting the crane

Summer

Outside temperature above +5°C

- Set ”WINTER/SUMMER” switch A34 to SUMMER.
- Close main switch HA - ON

Winter

Outside temperature below +5°C

- Set ”WINTER/SUMMER” switch A34 to WINTER.
- Close main switch HA - ON.
- Feed pump motor DD starts in 4-pole configuration via contactor A21 and runs at its lower speed of 1450 rpm (50 Hz) or 1750 rpm (60 Hz).
- Wait 24 hours.

  N.B. - The hydraulic oil must be warm before the crane may be started.

Starting and stopping the crane

Start sequence

- Press button XA10 "1" (Start)
- Warning lamp XA1 "Do not operate" goes on
- Feed pump motor DD is started in 2-pole configuration by contactors A22 and A23. The motor runs at its full speed of 2900 rpm (50 Hz) or 3500 rpm (60 Hz).

The feed circuit pressure build up its operating pressure. Pressostats FH - hoisting circuit, FJ - luffing circuit, close and start a delayed closing 8s time relay A27.

- After 8s, Y contactor A1.K2 switches on N contactor A1.K1; the main motor DB starts in Y connection.
- At the same time as the Y contactor was switched on, an 8s delayed-opening double relay A1.K9 was started.
- After 8s, the Y contactor drops, and D contactor A1.K3 is switched on by double time relay A1.K9 closing after 0.1s.

  The D contactor and self-holding N contactor switch main motor DB to delta configuration.
- Push button "1" (Start) closing prepares the energizing circuit of contactor A4 of oil COOL FAN MOTOR DA.

  Motor DA is started by thermostat FD if the temperature of the hydraulic oil exceeds +10°C.
- Warning light XA1 goes out. The crane is operational.

Stop sequence

- Press button XA10 "0" (Stop)
- All contactors of Y/D switches and all other motor contactors are de-energized. All motors stop.

- If the switch A34 is in ”winter” connection, feed pump motor DD does not stop but is switched to 4-pole configuration and continues to run at its lower speed of 1450 rpm (50 Hz) or 1750 rpm (60 Hz).
- The feed pump motor and the ventilation fan motor stops only when the main switch HA is switched OFF.

Other electric functions

Overheating protection

A thermostat FB in the hydraulic system of the crane opens the control circuits of the main electric motor and the oil cooler fan motor when the oil temperature exceeds +85°C.

The oil is cooled by setting the A34 switch to position "TEST" and pressing the START button on the control panel in the cab. The feed pump motor only then starts, together with the cooler fan.

Oil level switch

If the oil level in the hydraulic oil tank drops below the minimum permissible level, an alarm system is switched on by a float type oil level switch FD. Warning light XA2 goes on.

If the oil level sinks further, level switch FD opens the control circuit of the feed pump electric motor DD and main motor DB.

Siren

A pushbutton "SIREN" in the cab allows the operator to warn people on deck.

Ventilation fan

For all cranes a ventilation fan is fitted in the cranehouse. This is controlled by a +25°C thermostat A99 which is located in the A-box.

Lighting and heating

Electric power is supplied by separate slip-rings for lighting in the crane house, in the operator’s cab, and on the jib and heating in the cab.

The crane house lighting is operated by switches under the bottom plate. The cab lighting and the jib light are operated by switches in the cab.

Control system error

If an error is detected by the crane control system in the MB-box pilot light XA4 illuminates. Check the MB-box according to instruction "Crane Control System CC2000" under Section 6.3.

Crane Control System CC2000
Single crane type

General

The following description refers to the microcomputer based crane control system CC2000 for MacGREGOR Cranes hydraulic deck cranes, single crane versions.

Minor variations in the use of control system input and output signals due to differences in hydraulic and electric system may occur, the objective of this description is only to provide a general orientation concerning the principles of the control system.

For detailed information about the control system a complete electric circuit diagram, relating specifically to your crane, will be found in the instruction manual, section “Spare Parts“, Group 9.5. Also for detailed information about the hydraulic system a complete hydraulic circuit diagram will be found in section “Spare Parts“, Group 9.4.

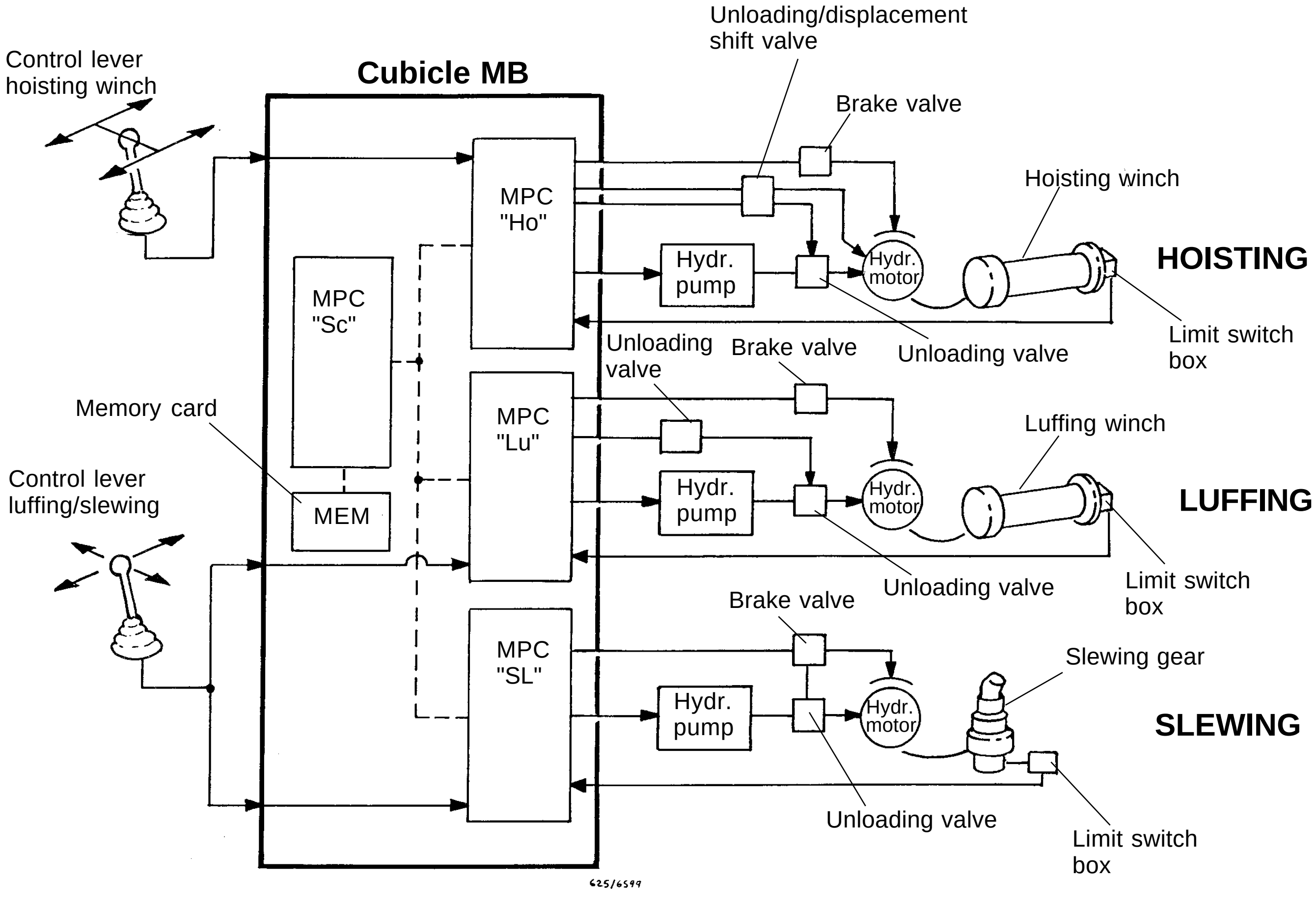

Fig.1. Crane control system with interfaces to control levers and hydraulic system

Crane control system - interfaces and principle of operation

Control levers (joysticks)

The right control lever controls the hoisting winch and the left control lever controls the luffing and slewing movements. The control levers linear potentiometers are fed with 0V, +6V and +12V. The output level is approx. +6V with the control lever in neutral. The output signal is controlled by the lever and is decreased to approx. +1,5V and increased to approx. +10,5V, depending on the lever movement.

The control lever for hoisting also gives 24V signals for selection of low speed/high speed and hoisting/ lowering.
The control lever for luffing/slewing also gives 24V signals for selecting luffing in/luffing out and slewing left/slewing right.

Crane control system in cubicle MB

The Crane control system consists mainly of four MPC - Micro Processor Cards. One MPC-card is used for internal communication between the cards and a parameter Memory Card. Three of the MPC-cards are controlling all input and output signals for one crane movement each, hoisting, luffing or slewing. Each MPC-card feeds a potentiometer in the control lever for the crane movement in control. Potentiometer and control lever direction signals go to a microprocessor with a software control program that handles brake release logic, overload logic and rampforming. The output from the microprocessor is fed to an voltage to current amplifier on the card.

The rampformer gives smooth acceleration and retardation to the crane movements. The brake release logic circuit releases and closes the brake in the correct sequence with the displacement/unloading signals when the hydraulic motor is started and stopped. The overload logic senses the overload input and switches from high speed to low speed or from low speed to stop. The voltage to current amplifier feeds the hydraulic pump solenoids.

Hydraulic pumps

The hydraulic pumps are variable displacement pumps (e.g., the flow is stepless variable from zero to max. rated flow). The pump oil flow is controlled with a 24V Pulse Width Modulated (PWM) current signal. The direction of the oil flow is dependent of which one of the two solenoid valves that are activated.

The speed of the winch, the pump displacement, the current through the solenoid coil and the lever movement are proportional to each other.

Hydraulic motors

The hydraulic motors may be reversed by changing the direction of the hydraulic pump oil flow. Each motor also having a low and a high speed range selected from the driver´s control lever.

Limit Switch Box, Hoisting

The limit switch box controls speed reduction and stop function for full or empty drum.

Limit switch box, luffing

The limit switch box controls speed reduction and stop function for all max. and min. outreach, and stop parking, if fitted.

Limit switches, slackwire

The limit switch for hoisting slackwire stops the lowering movement, and the limit switch luffing slackwire stops the luffing out movement when each wire is slacken.

Limit switches, slewing

If fitted, the limit switches for slewing controls speed reduction and stop function for each direction at maximum slewing range.

Computer system in cubicle MB, CC2000

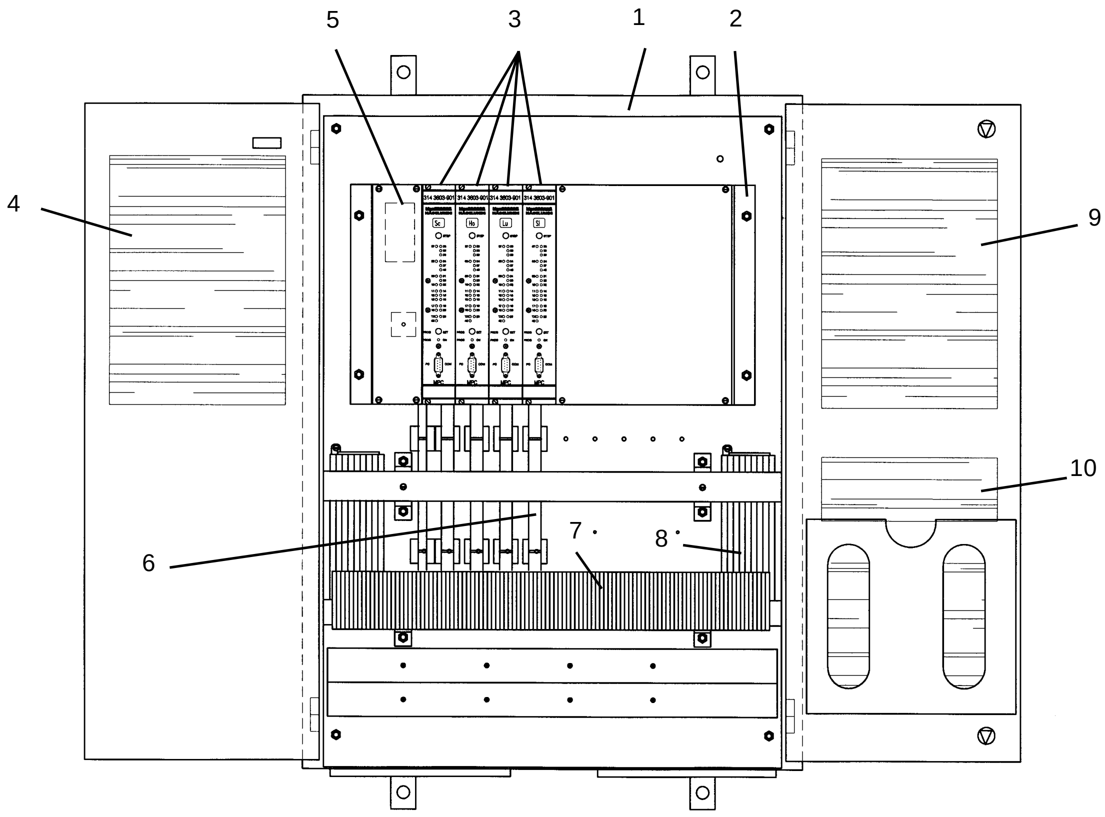

Fig. 2. Computer system in Cubicle MB, CC2000

1. Cubicle MB
2. 19“ rack with electronic cards
3. MPC-cards (Micro Processor Cards)
4. Parameter list 625 XXXXX
5. Parameter memory card (mounted on the backplane card)

6. Cables from MPC-cards to plinths
7 Plinths
8. Anti condensation heating
9. Sign: Signal overview
10.Sign: Error messages

Cubicle MB

The Crane control system CC2000 is protected against humidity by an casing with protection class IP54 (dust and sprinkle proof).

19“ subrack with electronic cards

All electronic cards are mounted in a standard 19 inch aluminium subrack. A backplane card is used for connecting power 8V and 24V to the MPC-cards. It also provides communication lines between the MPC-cards and between one MPC-card and the parameter memory card.

MPC - Micro Processor Card

Hardware

The control system consists mainly of four MPC-cards. All four cards are identical and 100% interchangeable.

Each MPC-card is a complete stand-alone microprocessor system with processor, several types of memories and communication channels, analog and digital inputs as well as analog and digital output current drivers.

Each card can fully handle all input and output signals needed for one hydraulic circuit, i.e. one crane movement. A separate safety system on each card gives processor-independent protection against hazardous operation at low feed pressure and overload pressure as well as it ensures stopping of the crane movement due to activated limit switches.

Software

All four MPC-cards are programmed with the same crane control software. Each card is sensing which rack-position and therefore which crane movement it is controlling.

All MPC-cards identifies themselves by showing a two-character abbreviation of the function/movement-name on the front panel display: Sc - supervisor, Ho - hoisting, Lu - luffing, SL - slewing.
The leftmost MPC-card, the MPC “Sc“, is only used for supervisory functions such as communication with other MPC-cards and distribution of control system parameters from the parameter Memory Card.

New versions of the crane control software can be loaded/programmed into the MPC-cards by using a standard notebook-PC.

Parameter list

The parameter list specifies all information details about the crane needed for the control system. It states the software version and software configuration parameters such as number of pumps, type of pumps, maximum speed, extra equipment etc.

Parameter values have both normal operation values and default values. Default values are used at “MPC default operation mode“, see page 6.

The parameter list identification number 625XXXXX, is the same as the crane manufacturing number seen on the sign in the driver's cabin (MFG NO.). All parameters are stored in the parameter memory card.

Parameter memory card

All parameter values from the parameter list are stored into the parameter memory card. The information is stored into electronic memories on the card. Stored parameter values are not dependent of power supply or battery. There are, however, a battery on the card used for a calendar, giving all MPC-cards date and time information. The card is located inside the rack and is mounted on the backplane card that also holds all the MPC-card connectors.
The lifetime of the battery is 10 years.

Cables from MPC-cards

Each MPC-card has a maximum of 42 cables connecting all the inputs and outputs between the card and its part of the plinth row. Cables are numbered 1 to 42 and put together into a braid.

Plinths

All external connections are made to a row of plinths in the lower part of the enclosure.

Each MPC-card has its own series of plinths. 101 - 142 for the MPC “Sc“, 201-242 for the MPC “Ho“, 301-342 for the MPC “Lu“ and 401-442 for the MPC “SL“. Note that there are not plinths for all numbers and that all plinths are not used on all types of cranes.

Plinth numbers from 1 to 99 are used for power and cross-connections for external limit-switches, key-switches, overload-switches etc.

Anti condensation heating

To avoid condensation on the electronic cards the temperature inside the enclosure is kept a few degrees higher than the temperature outside. Heating elements are therefore mounted behind the row of plinths. They are powered with 24V AC when the control system is switched off, when the crane is not operated.

Signal overview CC2000

Coloured LEDs

Signals to and from each MPC-card are indicated with green, yellow and red LEDs (Light-Emitting Diodes) on each MPC-card front panel.

Green are used for normal input and outputs. Yellow indicates that the crane motion concerned is driven with slower speed. Red indicates that the motion is stopped.

Signal overview sign

All signals indicated with LEDs are listed on the sign SIGNAL OVERVIEW inside cubicle MB, see section ”Service”, Group 6.3.

Identifying the plinth number corresponding to a LED

The plinth number is identified by a three digit number. The first digit of each number signifies the MPC-card to which the signal is connected:

1. Supervisory MPC-card, “Sc“
2. Hoisting MPC-card, “Ho“
3. Luffing MPC-card, “Lu“
4. Slewing MPC-card, “SL“

The last two digits signifies the LED number on each MPC-card front panel, showing the status of the 24V input and output signals for the card. The same two-digit number is used on the cables from the plinth to each MPC-card.

Error messages

Built-in diagnostics system

The CC2000 control system has its own built-in diagnostics system. The system is capable of detecting several types of malfunctions.

The system checks itself and external input and output signals. Internal problems like missing communication are continuously checked. Loose connections to pumps and valves are detected when the output is activated. Signal levels that does not correspond to normal levels, i. e. the potentiometer input from the driver's handle, are also detected.

When any type of malfunction is detected, the MPC-card flashes with its error LED, no 28. All MPC-card error LEDs are wired together to an error lamp in the drivers cabin, the “Control system error in MB-box“ lamp. To get the corresponding error message number, push the STEP push-button on the MPC-card with flashing LED.

If there are several errors, they are shown one at a time for each push on the STEP push-button. When there are no more error messages to read the display shows two hyphens “—“. The error LED is now either on or off. If it is on the error is still present but now acknowledged. If it is off the errors are not present at the time. When a new error is detected the error LED starts flashing again to indicate that a new error is found.

Error message sign

The corresponding error message text can be found on the sign ERROR MESSAGES in cubicle MB, see section ”Service”, Group 6.3.

Error priority level

Each error number has a corresponding priority level, ranging from PRI1 to PRI4, with PRI1 least, and PRI4 most serious. The priority level tells the control system how to behave when an error is detected depending on how serious the detected problem is.

- PRI1 is only for information and does not stop any crane operation.
- PRI2 stops the MPC-card that detected the error but it can be operated in “MPC default operation mode“.
- PRI3 stops the MPC-card both in normal and in default operation mode.
- PRI4 stops all the MPC-cards in the crane.

MPC default operation mode

A single crane normally uses four MPC-cards, but if one card is broken it is still possible to fully operate the crane with only three MPCs. The speed control is still stepless and safety functions as limit switches and overload protection are still working. The maximum speed of the crane is reduced but is still at least 50% of normal operation.

When one MPC-card is broken that card can be replaced by the leftmost MPC-card ”Sc”. It performes supervisory tasks but does not control any output signals. It distributes parameter values from the parameter memory card to the other MPC-cards, handles logged error messages etc.

Each crane movement can be operated stand-alone by its own MPC-card. The MPC “Sc“ must then be removed from its normal rack-position (probably replacing the broken card). An internal control signal then tells the MPC “Ho“, “Lu“ and “SL“ to operate slowly in “MPC default operation mode“, because the MPC “Sc“ is missing. Default operation means that each MPC-card uses its in-built default values for all control system parameters instead of the missing parameter values from the parameter memory card, normally distributed by the MPC “Sc“.

If two of MPC cards are broken, only two crane movements can be operated at the same time. The two remaining MPC cards can be used in any of the Ho, Lu and SL positions.

To activate the “MPC default operation mode“ see section “Service“, Group 6.3.

Emergency operation card

In case of MPC-card problems, there are also a possibility to replace the card with an Emergency operation card which is much less complex than the MPC-card. It is built up with relays that activates the outputs for brake, displacement/unloading and pump from the control levers 24V signals. The pump output is fixed, giving a constant speed of about 30% of maximum speed. Each motion is controlled by overload and limit switches, except the limit switch for high hook.

WARNING
When the emergency operation card is installed in the hoisting or luffing circuit, bear in mind that the high hook switch is inoperative.

Thermostatic Controlled Ventilation Fan

General

The ventilation fan is thermostatic controlled and situated inside the cranehouse top, see Fig. 1.

Function

With the main switch in position "1" (on-position) the ventialtion fan starts via a thermostat, positioned in the A-cubicle, see Fig. 2. The thermostat bulb is situated outside the cubicle.

The thermostat is activated at +25°C. The thermostat can be adjusted, see Fig. 3.

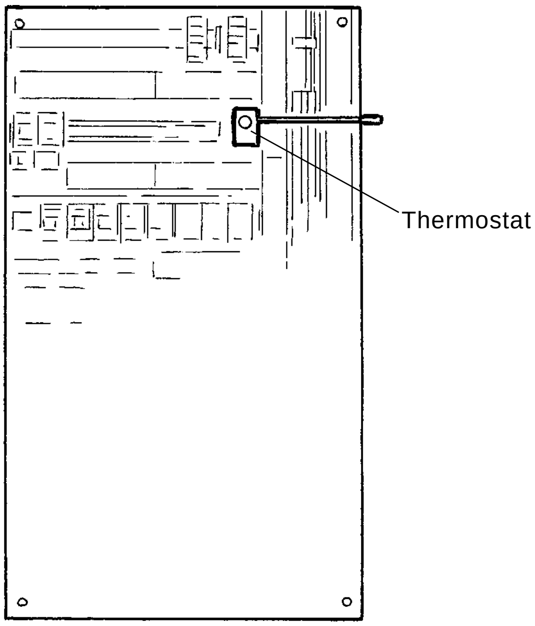

Fig. 2. Thermostat installed in A-cubicle

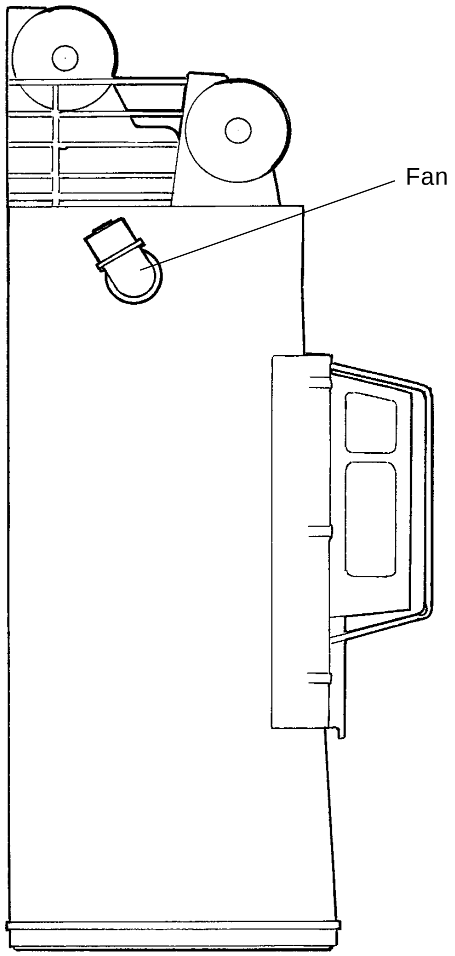

Fig. 1. Crane house

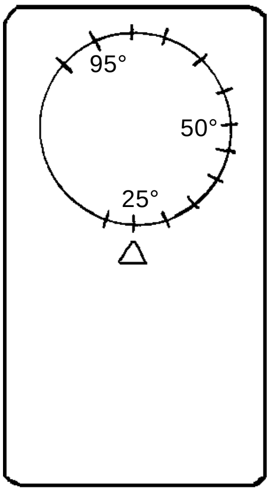

Fig. 3. Thermostat

Load Indication

The load indicator is located to the right and in front of the operator, see Fig. 1. The indicator is connected to the hoisting circuit. The load indicator comprises of a pressure manometer for the system, which measure the pressure in the hydraulic system corresponding the weight of the load.

The indicator value is valid at brake opening and full speed of the winch.

The indicator is fitted with a sign with a corresponding scale for the weight in metric tons.

The load indicator has **two main scales**, blue and red.

The **blue scale** is valid for the **low speed** 2 parts/4 parts driving mode and the **red scale** is valid for the **high speed** 2 parts/4 parts driving mode.

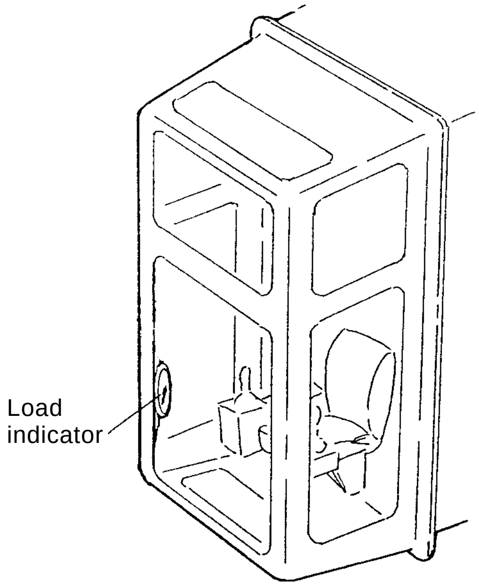

Fig. 1.

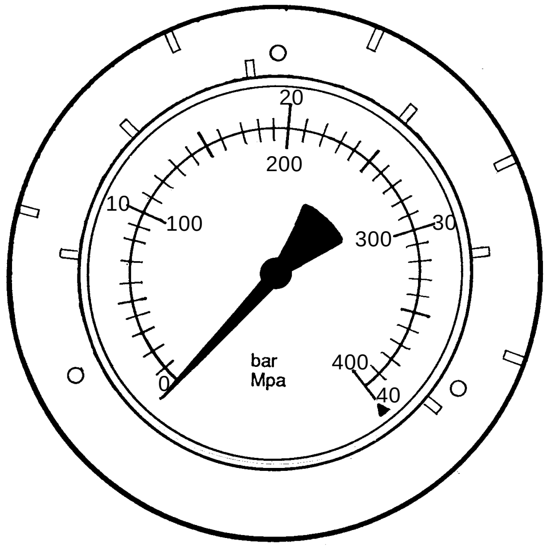

Blue = Low speed 2 parts / 4 parts
Red = High speed 2 parts / 4 parts

Fig. 2.

CREW CHECKLIST

BEFORE TAKING CRANE INTO OPERATION

Note! When the crane is rigged ready for operation, all keys for all lockable switches must be stored in a safe place by the officer responsible on board.

- Read sections Operation and Maintenance in the instruction manual and make sure that maintenance is performed according to Maintenance Chart.
- Make sure nobody enters the crane without permission from responsible officer on board.
- Make sure that there is nobody in the crane house who might be caught in the machinery or strangled by the wire ropes.

The following checks and procedures must be performed before taking crane into operation:

Summer

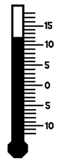

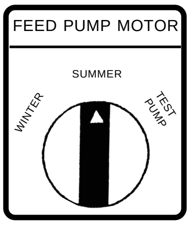

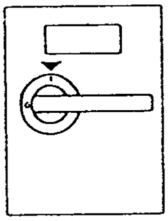

When outside temperature is above +5°C...	selector shall be set to SUMMER.	Switch main switch to position "1".

Winter

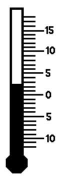

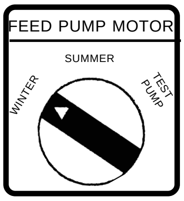

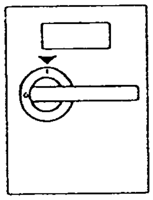

When outside temperature is below +5°C...	selector shall be set to WINTER.	Switch main switch to position "1" some 24 hours before operation.

Oil level and oil temperature

Verify hydraulic oil level and that the oil temperature is above +5°C in oil tank.
Also check oil level in slewing gearcase.

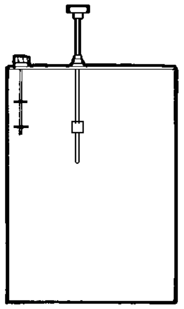

Unclamp jibs and loose gear

Unclamp jib and loose gear from its parking position.

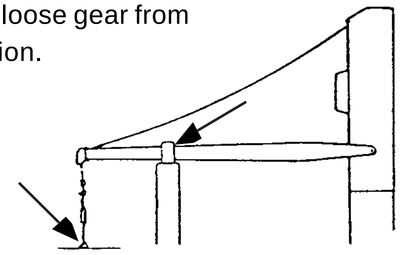

Wires and sheaves

Verify that wires run correctly in the sheaves and that the wire ends are securely clamped.

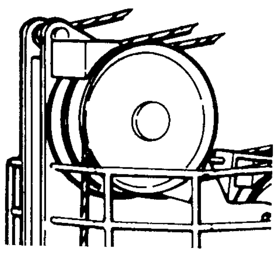

DURING OPERATION

List/trim

Verify that ship does not list more than 5 degrees and does not trim more than 2 degrees.

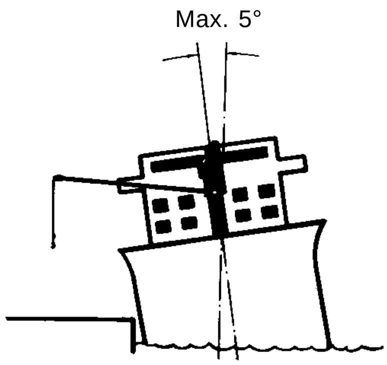

IF CRANE STOPS

Check the following:

a. Crane overheated

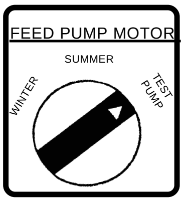

1. Set FEED PUMP MOTOR switch in TEST PUMP position.
2. Press START button (to start feed pump unit in high speed).
3. Check that oil cooler fan is running.
4. After approx. 30 min. set FEED PUMP MOTOR switch in SUMMER position.
5. Try to start the crane by pressing START button.

b. Oil level

1. Check oil level in oil tank.
2. Fill oil if necessary. (See Lubricating Chart).

c. Fuses

1. Check fuses in HF cabinet.
2. Change fuses if necessary.

AFTER OPERATION

Park the crane

Park the crane according to separate instruction in section 4 of the instruction manual.

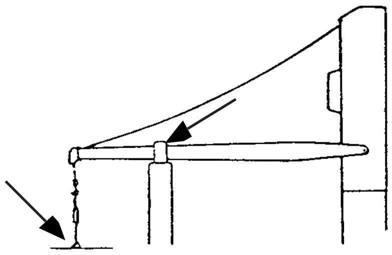

AFTER SEA STOWING

Anti condensation heating (if cubicle MB fitted)

Check that the heating elements (two large or three small) in cubicle MB are working, i.e. gets warm.

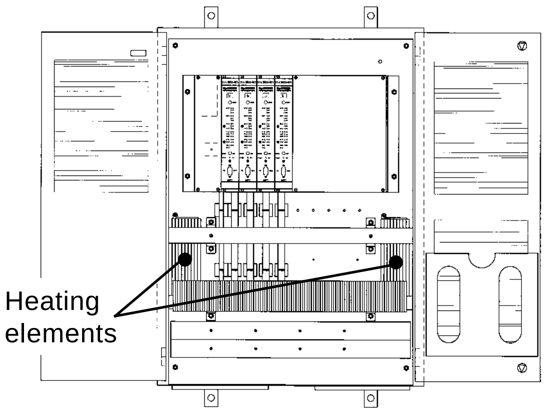

Also see instr. "Crane control system", section 3.

OPERATING INSTRUCTION

Handling of dangerous cargo

- Dangerous substances shall be handled in accordance with the relevant requirements of National or Local Regulations and applicable National and International standard, only under the supervision of a competent person who is familiar with the risks and the precautions to be taken.

- Further requirements are stated in ILO Codes of Practice, Safety and health in dock work.

The crane is **not** designed for transport of passengers.

OPERATING INSTRUCTION
START - STOP

MacGREGOR cranes are designed for operation in harbour or sheltered water environments where there is no significant movement of the ship due to wave action.

WARNING!
Only ship's crew is allowed to operate key switches. Keys must be stored in a safe place by responsible officer.

- Notify responsible officer onboard before taking crane into operation.
- Make sure that there is nobody in the crane house who might be caught in the machinery or strangled by the wire ropes.
- When using grab or magnet, handling log or palletized cargo the lifting capacity must be reduced to 80% of SWL if there is no "GRAB" switch in cabin.

BEFORE STARTING

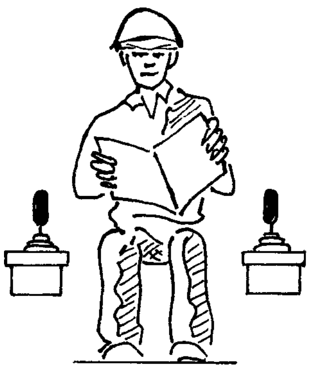

Read instructions carefully before taking crane into operation.

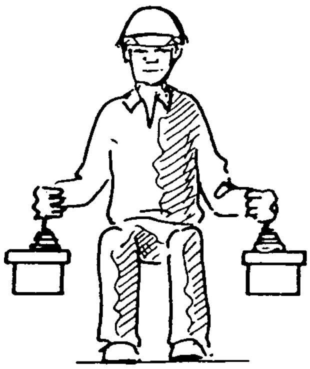

Check that control levers are in neutral. If not, notify responsible officer.

START

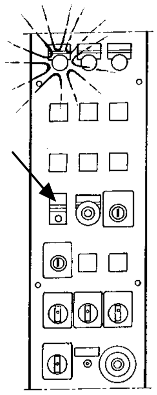

Set switch (automatic return) into position 1
The crane is operational when the red warning light goes out.
Do **not** operate on red light.

EMERGENCY STOP

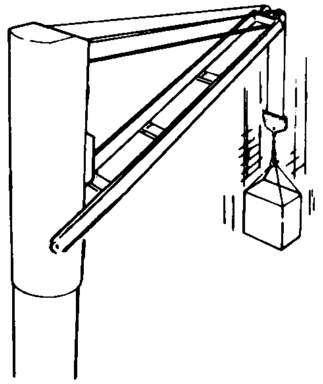

If the crane gets out of control...

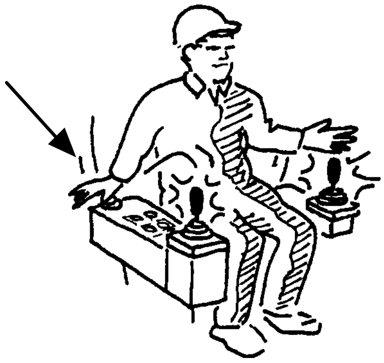

- LET GO both control levers; they will return automatically to their neutral positions.
- Press Emergency Stop button!

STOP

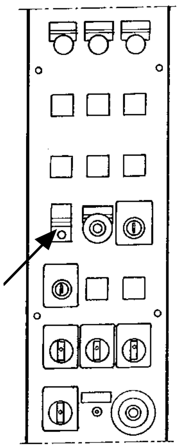

Set switch into position 0 (automatic return)

CRANE OPERATION

CONTROL LEVER, HOISTING / LOWERING

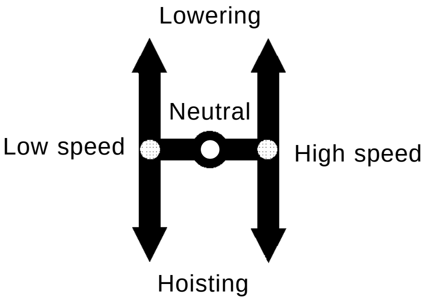

With the hoisting winch control lever in neutral, the winch brake is engaged and holds the load.

Moving the hoisting winch lever to one of the grid slots for high or low hoisting speed releases the winch brake; the winch starts hoisting at creep speed. This speed is depending on the load.

CONTROL LEVER, HOISTING / LOWERING

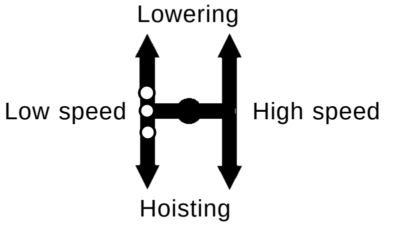

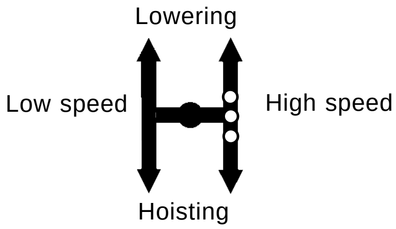

To be able to hold the load at constant height move the hoisting winch control lever to one of the positions shown above. The brake will then be realeased and the load will be held at constant height. The position of the lever is depending on the load on the hook.

CONTROL LEVER, SLEWING / LUFFING

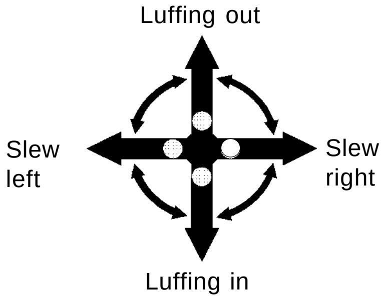

With the slewing/luffing control lever in neutral, the brakes are engaged.
Moving the lever a short distance from neutral releases the brakes.

Moving the lever forwards or backwards starts operation of the luffing winch.
Moving the lever left or right starts the crane to slew in the direction chosen.

LIST / TRIM

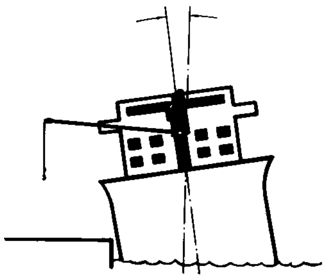

Verify that ship does not list more than 5 degrees and does not trim more than 2 degrees.

DRAGGING LOADS

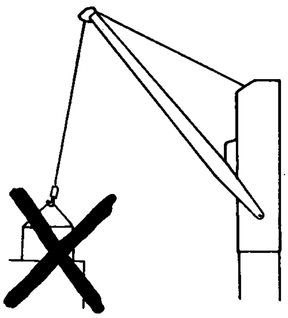

Dragging loads is **not** permitted!

Do not drag loads - lift them.

SHORT BREAKS IN OPERATION

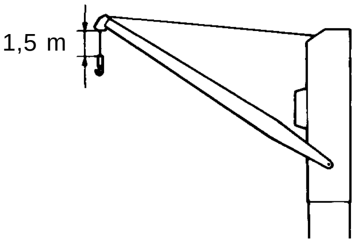

... or if a technical malfunction has developed:

- Raise swivel to approx. 1.5 m below jib head, if possible.
- Return control levers to neutral.
- Press STOP button.

OPERATING INSTRUCTION
START - STOP

MacGREGOR cranes are designed for operation in harbour or sheltered water environments where there is no significant movement of the ship due to wave action.

WARNING!
Only ship's crew is allowed to operate key switches. Keys must be stored in a safe place by responsible officer.

- Notify responsible officer onboard before taking crane into operation.
- Make sure that there is nobody in the crane house who might be caught in the machinery or strangled by the wire ropes.
- When using grab or magnet, handling log or palletized cargo the lifting capacity must be reduced to 80% of SWL if there is no "GRAB" switch in cabin.

BEFORE STARTING

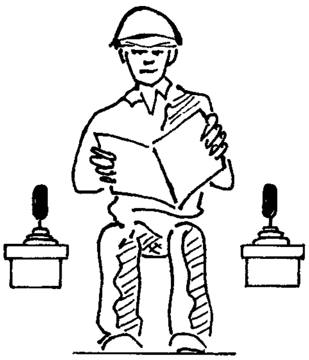

Read instructions carefully before taking crane into operation.

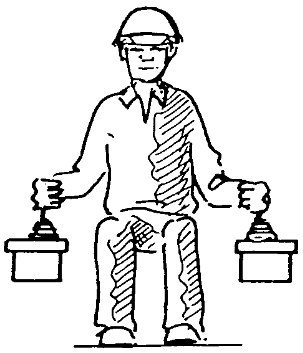

Check that control levers are in neutral. If not, notify responsible officer.

START

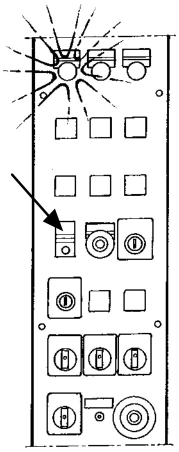

Set switch (automatic return) into position 1.
The crane is operational when the red warning light goes out.
Do **not** operate on red light.

STOP

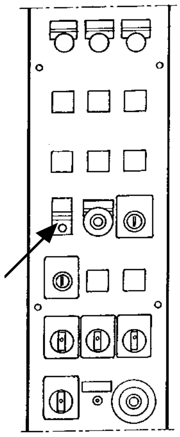

Set switch into position 0 (automatic return).

EMERGENCY STOP

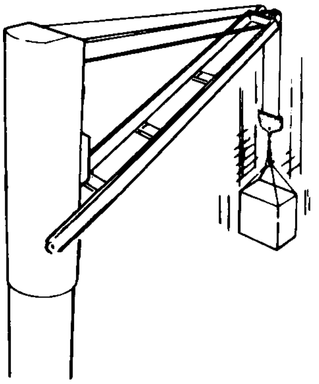

If the crane gets out of control...

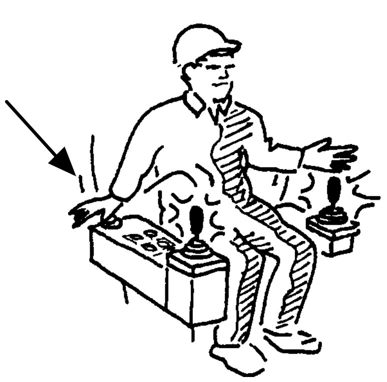

- LET GO both control levers; they will return automatically to their neutral positions.
- Press **Emergency Stop** button!

SLEWING OPERATION

Do not operate slewing if the red lamp "PARKING LOCK NOT ENGAGED" illuminates and the "ALARM" is activated or if the red lamp "PARKING LOCK ENGAGED" illuminates during normal cargo handling. Stop and check parking lock device.

See also separate instruction "Parking of crane in wires".

CRANE OPERATION

CONTROL LEVER, HOISTING / LOWERING

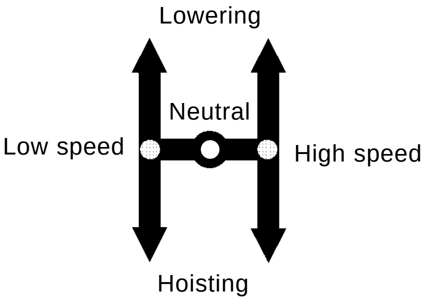

With the hoisting winch control lever in neutral, the winch brake is engaged and holds the load.

Moving the hoisting winch lever to one of the grid slots for high or low hoisting speed releases the winch brake; the winch starts hoisting at creep speed. This speed is depending on the load.

CONTROL LEVER, HOISTING / LOWERING

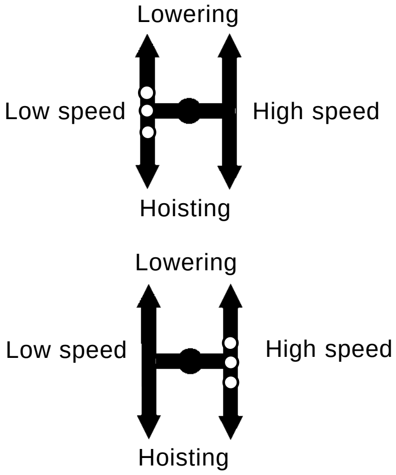

To be able to hold the load at constant height move the hoisting winch control lever to one of the positions shown above. The brake will then be realeased and the load will be held at constant height. The position of the lever is depending on the load on the hook.

CONTROL LEVER, SLEWING / LUFFING

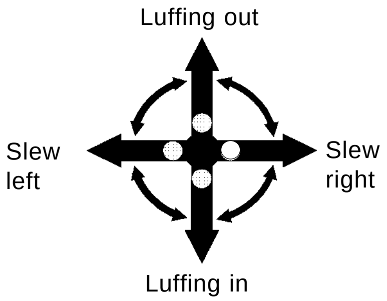

With the slewing/luffing control lever in neutral, the brakes are engaged. Moving the lever a short distance from neutral releases the brakes.

Moving the lever forwards or backwards starts operation of the luffing winch.
Moving the lever left or right starts the crane to slew in the direction chosen.

LIST / TRIM

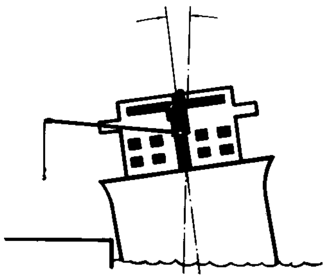

Verify that ship does not list more than 5 degrees and does not trim more than 2 degrees.

DRAGGING LOADS

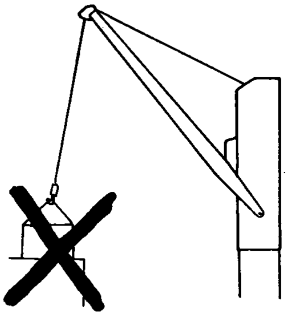

Dragging loads is **not** permitted!

Do not drag loads - lift them.

SHORT BREAKS IN OPERATION

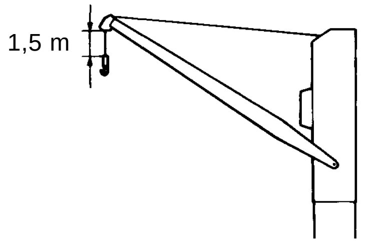

... or if a technical malfunction has developed:

- Raise swivel to approx. 1.5 m below jib head, if possible.
- Return control levers to neutral.
- Press STOP button.

Controls and Indicators

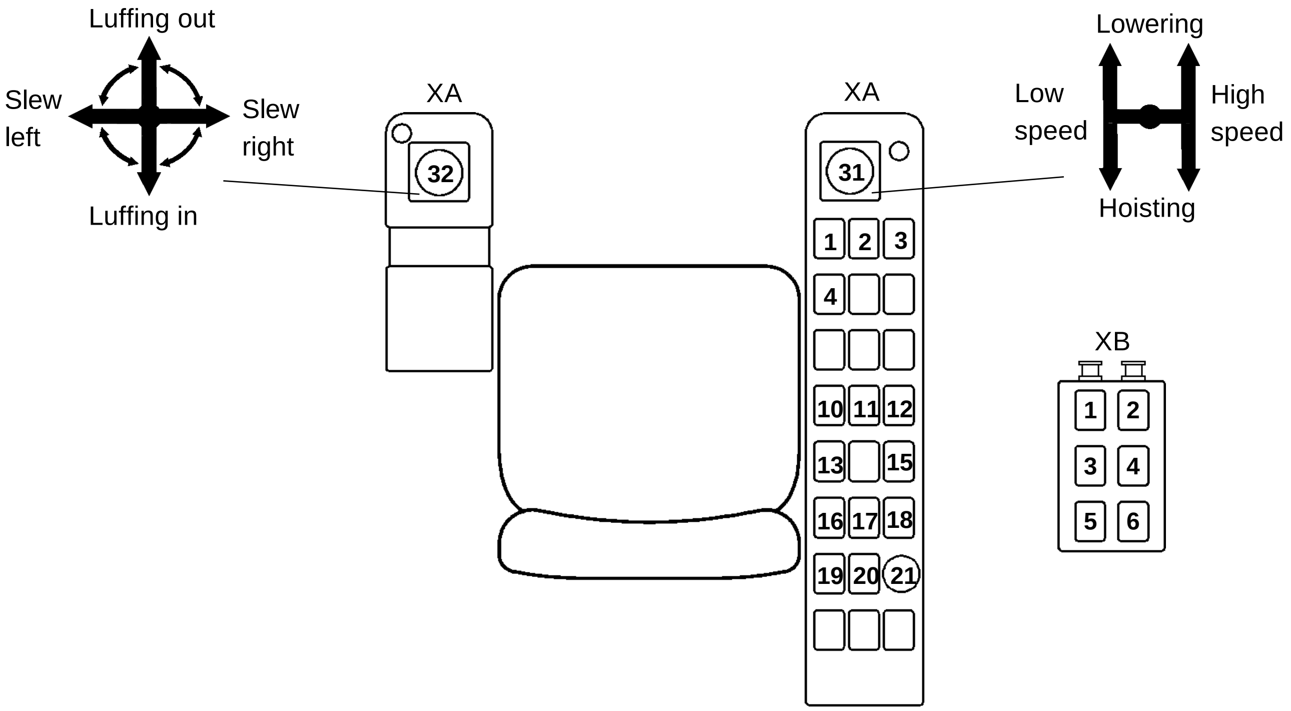

Fig. 1. Controls and indicators in the crane cabin.

> **WARNING!**
> Only ship's crew is allowed to operate key switches.

> **WARNING!**
>
> **Store Keys Safely**
>
> When the crane is rigged ready for operation, all keys for all lockable switches must be stored in a safe place by the responsible officer on board.

(XA) 1 DO NOT OPERATE
Pilot light. Illuminates during starting sequence of the crane (feed pump motor, main electric motor and supply to electronics).

(XA) 2 LOW OIL LEVEL
Pilot light. Illuminates as a pre-warning for low oil level in crane and the siren or bell, which is mounted under the crane cabin will sound. Refill oil tank. The pilot light and the siren/bell can be switched off by selecting the key float switch (XA) 12 in position ”Bell off”.

(XA) 3 CHECK OIL FILTER
Pilot light. Illuminates when the oil pressure over the oil filter is more than 5 bar.The oil filter is saturated and ought to be changed or hydraulic system is to be checked.

(XA) 4 CONTROL SYSTEM ERROR IN MB-BOX
Pilot light. Illuminates when an error is detected by the crane control system in the MB-box.

(XA) 10 START / STOP (1 / 0)
Pushbutton with two positions:
- Position "1" starts the complete crane. During the starting sequence the ”Do not operate” pilot light illuminates.
- Position "0" stops the complete crane. The power supply to the main electric motor, the feed pump motor, running in normal speed "Summer position", and electronics will be broken. When the feed pump is running in low speed, ”Winter position” (advised at an outside temperature lower than +5°C), then after stopping the crane, the feed pump continues to run.

(XA) 11 BELL
Pushbutton. The bell sounds when the pushbutton is activated.

(XA) 12 FLOAT SWITCH
This four positions **key switch** is normally in position "1".
Normal oil level is between MIN and MAX. If the oil level drops the indicator lamp ”Low oil level” illuminates and the alarm will sound.
A key is needed to select the following three positions:

1) The ”TEST” position is used to check the function of the siren/bell, as well as if the ”Low oil level” and the ”Do not operate” lamps illuminate.
2) The ”BELL OFF” position is used in an emergency. The siren/bell will be silent and the indicator lamp ”Low oil level” goes out. The operation of the crane may be continued for a short time. Check why oil level has dropped!
3) The ”BY-PASS” position is in **Extreme Emergency** to lower a load which is hanging in the hook. The ”Low oil level” lamp continues to illuminate and the crane siren/bell sounds.

(XA) 13 PARKING OF JIB
Key switch. Is normally in position "0".
When luffing out is stopped due to the maximum outreach limit switch, further lowering of the jib is possible (for parking purposes of jib) only if the key switch is turned to position "1".

(XA) 15 WARNING LIGHT JIB
Switch. Switch in position ”0” warning light is off. Switch in position ”1” warning light is on.

(XA) 16 HEATER CABIN
Switch. On/Off. Starts and stops the cabin heater.

(XA) 17 CAB LIGHT
Switch. On/Off.

(XA) 18 FLOODLIGHT
Switch. On/Off.

(XA) 19 WIPER
Switch. On/Off. Starts and stops the window wiper.

(XA) 20 RESET WIPER
Pushbutton. Resets the wiper motor (automatic fuse), when the wiper motor has stopped due to a too high current.

(XA) 21 EMERGENCY STOP
Pushbutton. Stops the complete crane in case of emergency. The pushbutton has to be reset, otherwise the crane cannot be restarted.

(XB) 1 PARKING LOCK ENGAGED
Pilot light.
See separate instruction PM46 2505-E00.

(XB) 2 PARKING LOCK NOT ENGAGED
Pilot light.
See separate instruction PM46 2505-E00.

(XB) 3 PARKING SLEWING LOCK
Key switch.
See separate instruction PM46 2505-E00.

(XB) 4 LOCK PINS
Switch.
See separate instruction PM46 2505-E00.

(XB) 5 PARKING POSITION SLEWING LOCK
Pilot light.
See separate instruction PM46 2505-E00.

(XB) 6 ALARM
Buzzer.
See separate instruction PM46 2505-E00.

Note! (XB) 1 - 6 valid for crane no. 2.

(XA) 31 CONTROL LEVER, HOISTING/LOWERING
When control lever is in neutral position the brake is engaged and holds the load.
Moving control lever in low speed or high speed position releases the winch brake.
Moving control lever in hoisting or lowering direction means continued speed increase from zero to max. speed.
To give a smooth acceleration and retardation to the winch movement the control system is fitted with a ramp former with a special ramp time.
The ramp time is the time it takes to increase the winch speed from standstill to maximum speed, when the control lever is moved rapidly from zero to maximum speed position. In the same way it also takes the corresponding ramp time to decrease the winch speed from maximum speed to standstill, if the control lever will be dropped.
The ramp time for the hoisting system is 2 sec.

Power swivel, slewing CW.
Press pushbutton on the top of the control lever. *

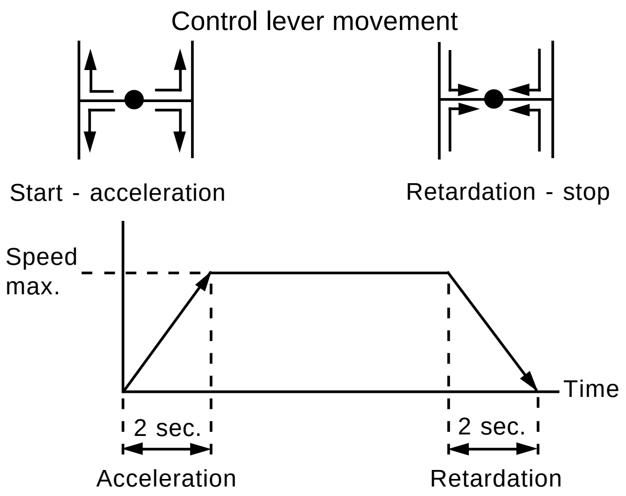

Fig. 2. Hoisting/lowering

(XA) 32 CONTROL LEVER, LUFFING/SLEWING
With the control lever in neutral position the brakes are engaged.
Moving the control lever short distance from neutral releases the brake for luffing winch or/and slewing machinery.
Moving the control lever furthermore in luffing or slewing direction means continued speed increase from zero to max. speed.
To give a smooth acceleration and retardation to the luffing winch movement and the slewing movement the control system is fitted with a ramp former with a special ramp time.
The ramp time is the same as it takes the luffing winch speed or the slewing machinery to increase from standstill to maximum speed when the control lever is moved rapidly from zero to maximum speed position.
In the same way it also takes the corresponding ramp time to decrease the winch speed from max. to standstill if the control lever will be dropped.
The ramp time for luffing is 2 sec. and for slewing 4 sec.

Power swivel, slewing CCW.
Press pushbutton on the top of the control lever. *

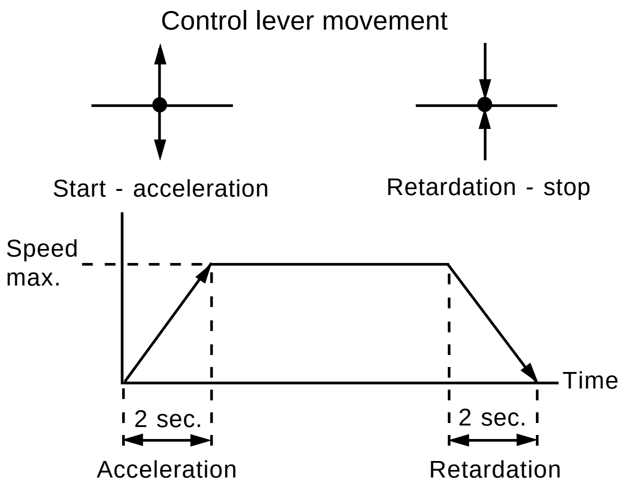

Fig. 3. Luffing

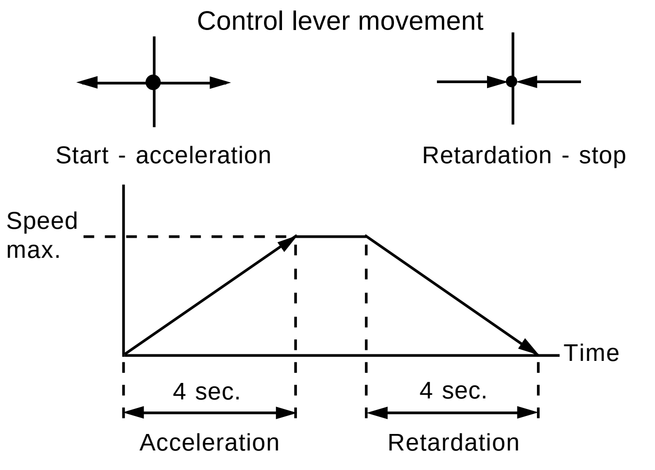

Fig. 4. Slewing

* Pushbuttons on top of the control lever to be used only when operating extra equipment e. g. power swivel.

Emergency Escape Instructions

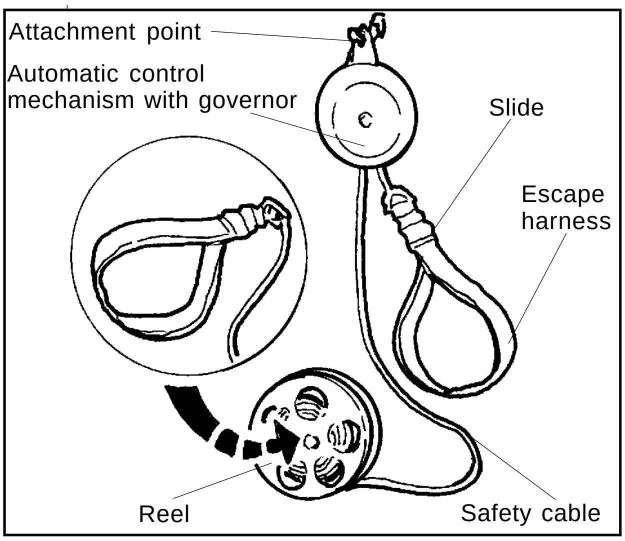

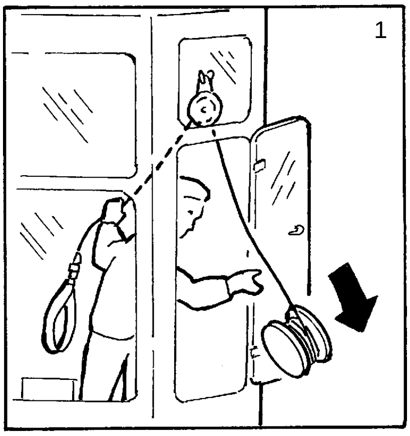

1. Untie escape harness wrapped around cable. Drop cable and reel to ground.

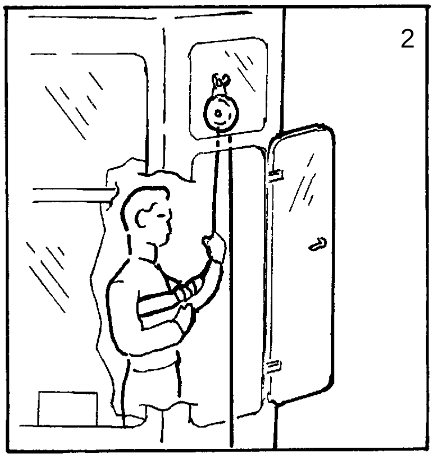

2. Place escape harness around body, underneath shoulders, and pull metal slide tightly to chest.

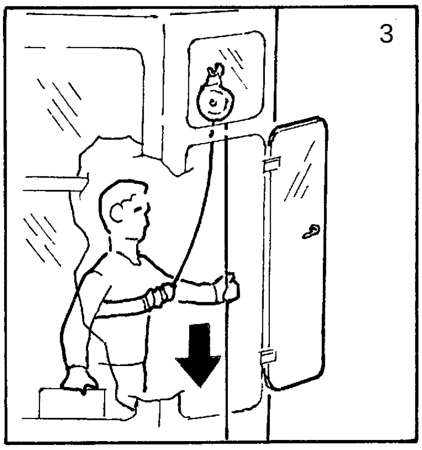

3. Pull harness tight to Rescumatic unit by pulling down on rope to ground.

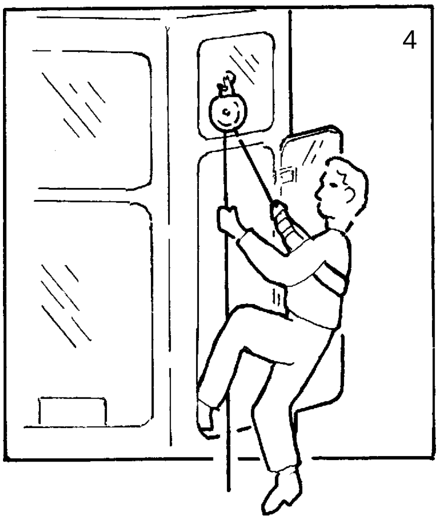

4. Step - DO NOT JUMP - from structure.

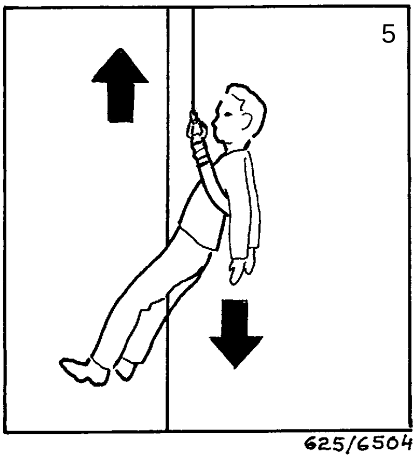

625/6504

5. The unit will then automatically lower user to ground. The second escape harness will ascend and can then be used for the next person.

Installations and warnings

Before anchoring the Rescumatic ensure that the anchorage is perfectly sound and comply with prEN 795 or subsequent standard. The device should always be anchored above the level of the user. Attention should be paid:

a) to ensure a clear and obstruction free path exists, down to ground level
b) that the device is anchored clear of elements which might damage or affect the correct safe functioning of the device (e.g. corrosive substances, moving machinery etc.) and avoid the risk of swing falls.

The device may be anchored semi-permanently by use of a Saflok Karabiner hook. However, where extreme vibration is likely, users are advised to have the control unit rigidly fixed to a solid mounting.

Obstruction warning

Ensure the Rescumatic is anchored so that the path of the descent is free of obstacles which might hinder swift and safe descent.

Free fall/jump warning

The Rescumatic should never be subjected to fall arrest or impact forces. Before making a descent always ensure that the user is directly below device and that the cable is taut.

Inspection

The Rescumatic device should be inspected on a regular basis:

1. Visual inspections should be carried out at least once a month with particular attention being paid to the condition of rope and escape harness.
2. At least once a year the device should be removed for service and returned to MacGREGOR-Hägglunds for servicing.

In environments where there is a higher risk of corrosion, due to a corrosive or chemical environment, the frequency of service and inspection may need to be increased.

Never use a device which shows signs of damage, corrosion, mechanical defect, etc.

Parking the Crane, Sea Stowing General Instruction

Introduction

Prior to sea voyage, the jib/jib extension should be put on its parking support (see Fig. 1). The hook is to be stowed to deck, fibrestrops with low ultimate load or manual stretching screw must be used (see Fig. 5). If the hook is not secured to deck a jib safety lock is recommended to lock the crane jib in the parking support. See Fig. 6.

Sea stowing with cargo hook secured to deck, see Fig. 1

1. Lower the jib into parking support, using the key switch "Parking of jib". See Fig. 2.
2. Run the block up to suitable height.
3. Secure hook to deck as shown in Fig. 5.

Sea stowing with cargo hook/block hoisted to jib top, see Fig. 6

1. Lower the jib into parking support, using the key switch "Parking of jib". See Fig. 2.
2. Run the block up against the jib. Do NOT tighten to hard. (The jib must still be resting on the support).
3. We recommend to secure the hook to the deck with a rope otherwise the hook will be swinging during sea voyage. See Fig. 3.

When parking the jib with the hook block hoisted to jib top it is important that the parking support is provided with an arrangement to lock the crane jib/jib extension. See Fig. 6.

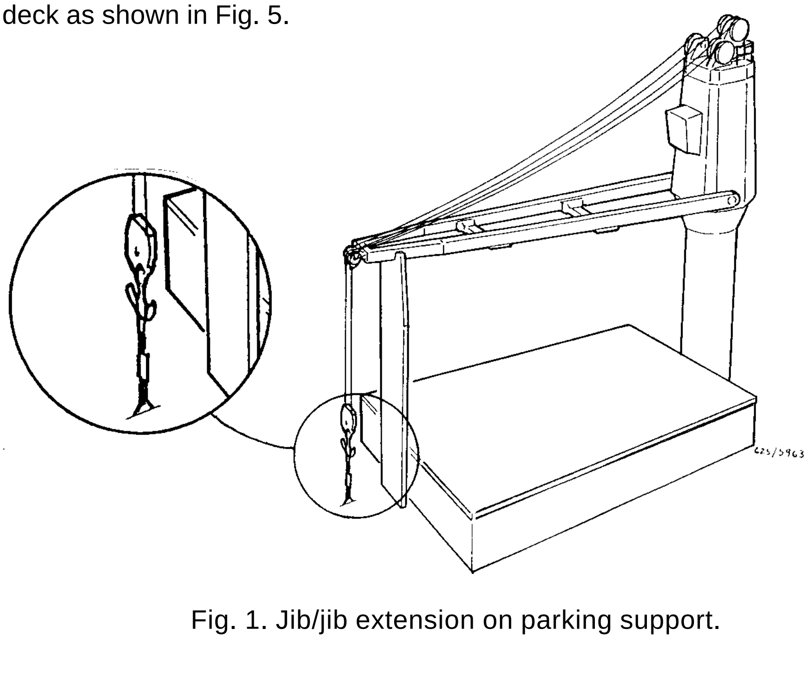

Fig. 1. Jib/jib extension on parking support.

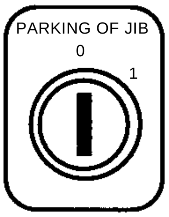

Fig. 2. Switch in cabin.

Important!

The wire ropes must not be tightened. The crane structure, wire ropes, sheaves, and winches are subject to heavy stress during sea voyage.

Note! Releasing the hook from sea stowing position must be done manually as the hook will start hoisting when the brake is released.

Prior to cargo handling

Make sure the hook and the crane jib are loosened before raising the jib from the parking support. Also ensure that the crane jib is luffed-in to its working area (e.g. within max. outreach).

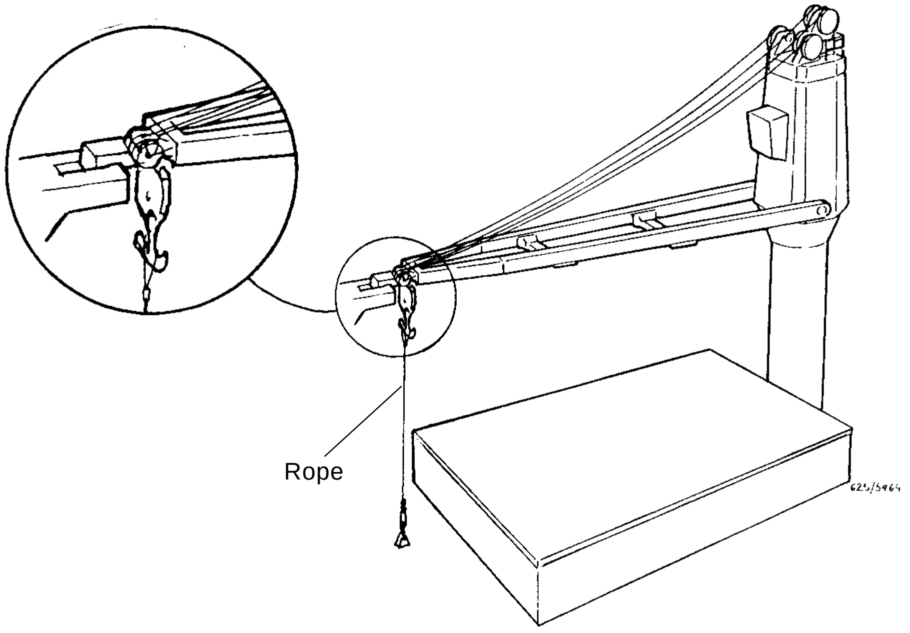

Fig. 3. Block secured to deck by using a rope.

Crane jib parked on next crane housing, see Fig. 4. (If no electrical interlocking.)

Example:
Before operating crane No. 1 carefully check that crane No. 2's jib has been removed from its parking support.

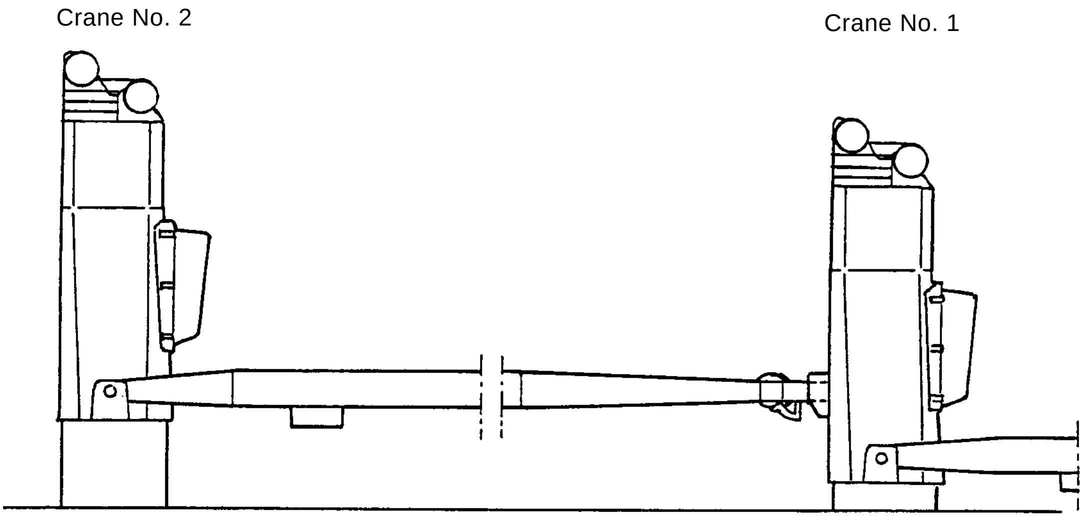

Fig. 4. Jib parked on crane housing

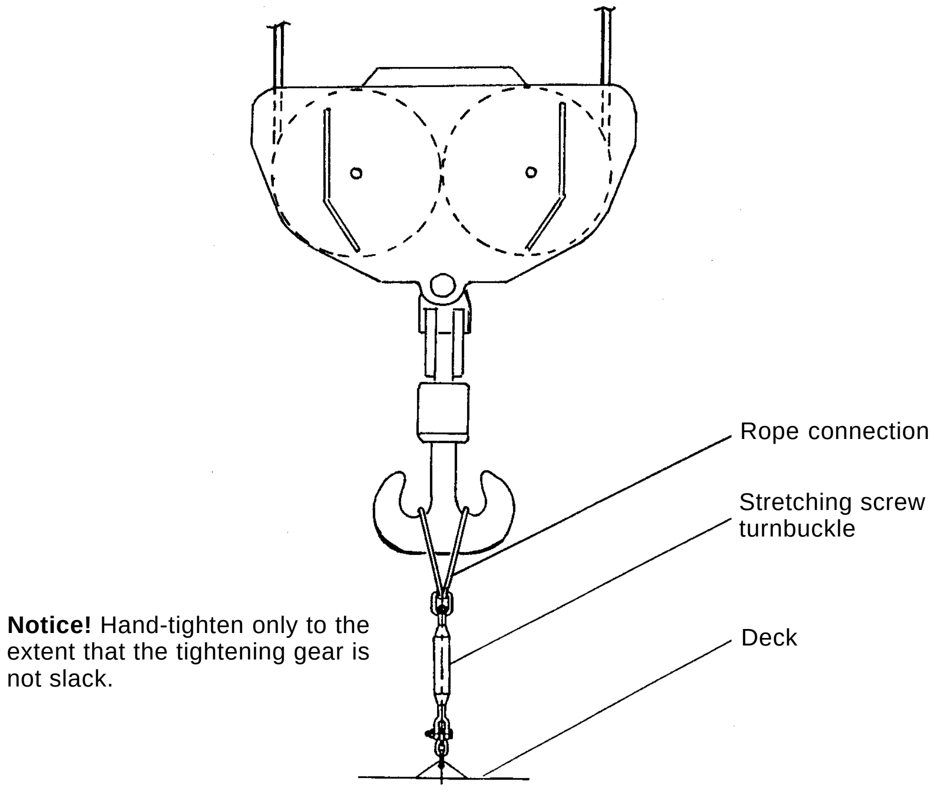

Fig. 5. Hook secured to deck.

Jib parking arrangement

Lifting block tightened to jib top with hoisting rope.

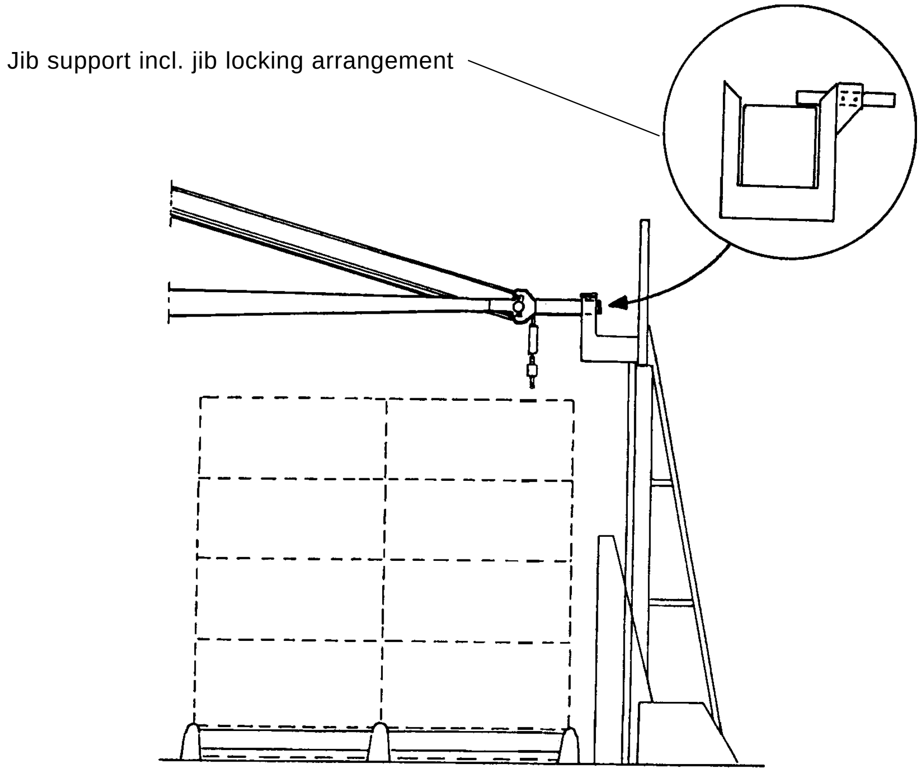

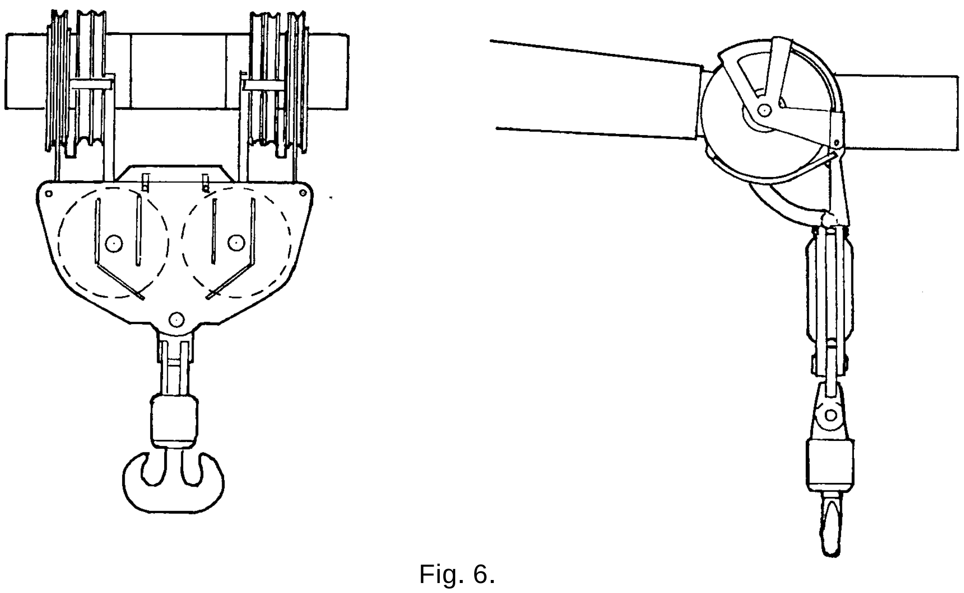

Fig. 6.

Parking suggestions of extra equipment

Example:
The grab should be lowered to the deck when the crane is parked, this to avoid load on the wires.

Important!
Power cable and stabilizing cable should be parked on jib top during sea voyage. Parking on hook block is for temporary use only.

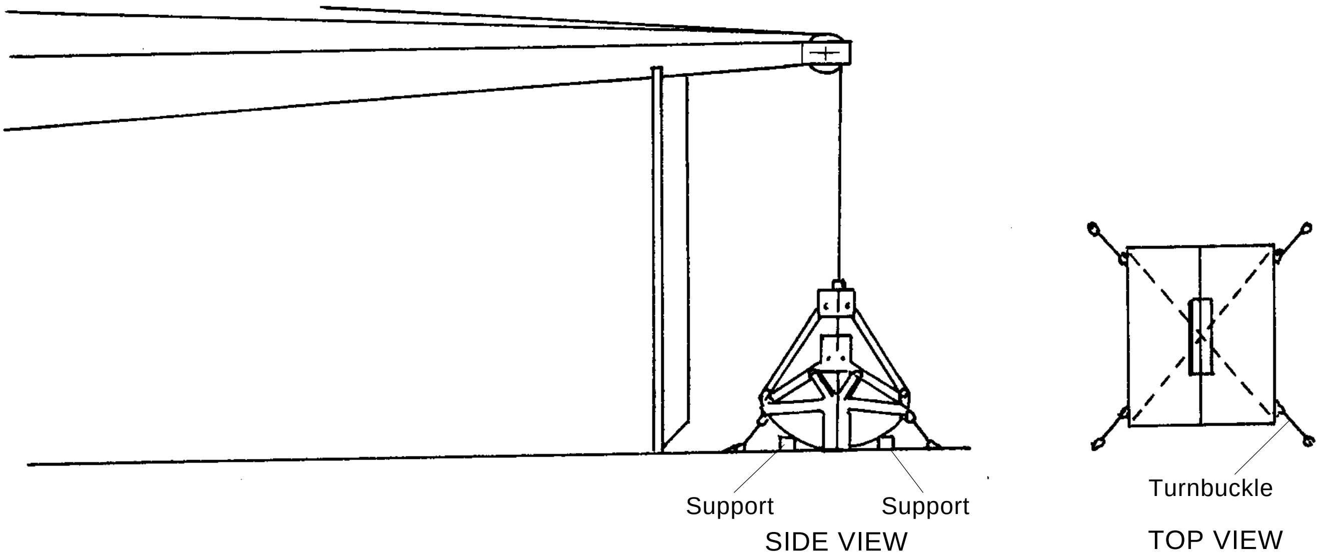

Fig. 7. Grab lowered to deck, fixed with suitable tightening equipment.

Example:

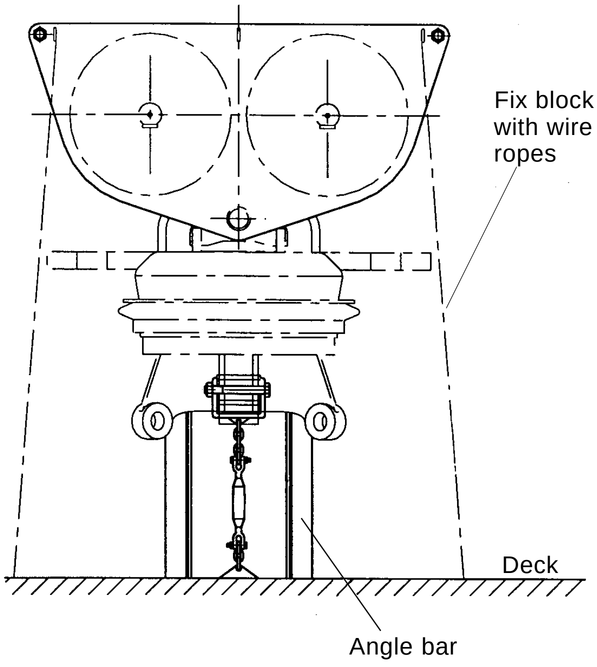

Fig. 8. Power swivel with lifting block secured to deck.

Example:

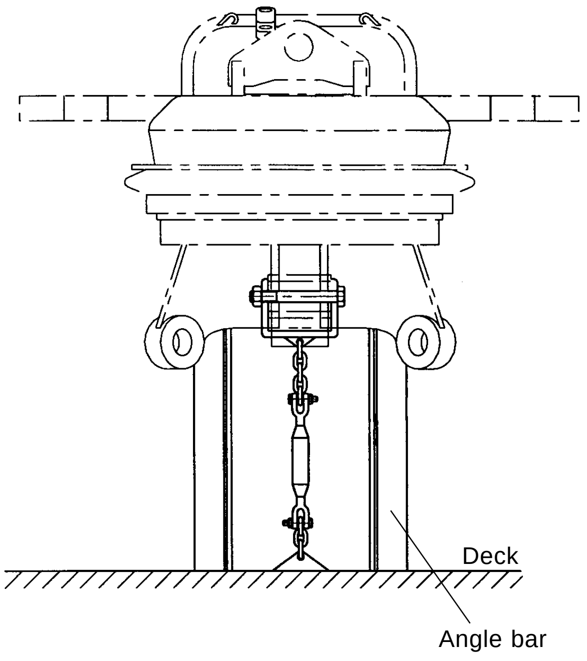

Fig. 9. Power swivel secured to deck.

Parking of Crane in Wires

Caution!
All keys for all lockable switches must be stored in a safe place by the officer responsible on board.

Slewing of crane to parking position

- Move the crane jib into min. jib radius position.
- Set the **key switch** "PARKING SLEWING LOCK" in position 1 (should be kept in this position until crane has been released from its parking position). See Fig. 1.
- Slew the crane until the signal lamp "PARKING POSITION" illuminates. See Fig. 1. The crane slews in speed reduce during this operation.
- Lock the crane by setting switch "PARKING LOCK PINS" in position "LOCK".
- The red lamp "PARKING LOCK NOT ENGAGED" and the "ALARM" remain active until the crane is locked in its parking position. If either (or both) of the lock pins is not in the exact locking position, the crane can be inched to the required position. See Fig. 1 and 2.
- The red lamp "PARKING LOCK PINS ENGAGED" should now illuminate. See Fig. 1 and 3.

Parking of jib and hook block

When the crane is locked in position, the jib and block are parked in accordance with the following.

- Move the jib into max. jib radius position (Max R).
- The spring loaded key switch "PARKING OF JIB" must be held open while parking the jib/hook block.
- Park the jib at max jib R. If possible, the load wire should be horizontal. See Fig. 4. When parking at an angle under max. R, the jib is to be positioned at reduced speed and without further stopping. (Existing stop, empty drum 0°.)
- Park the hook block by running it upward to the wire guides at the jib top. See Fig. 5. **Note:** The hook block should be tightened against the wire guides, but not so much that the luffing wire gets slack.
- Secure hook to deck with rope. (This is not really needed but recommended, otherwise the hook will be swinging during sea voyage).

Releasing of crane from parking position

- Move the crane jib into min. jib radius position.
- Set switch "PARKING LOCK PINS" in position "UNLOCK" and wait until lamp "PARKING LOCK NOT ENGAGED" goes out.
- Set the **key switch** "PARKING SLEWING LOCK" in 0-position.

Caution!

Normal operation

Do not operate slewing if the red lamp "PARKING LOCK NOT ENGAGED" illuminates and the "ALARM" is activated or if the red lamp "PARKING LOCK PINS ENGAGED" illuminates during normal operation of cargo. Stop and check parking lock device.

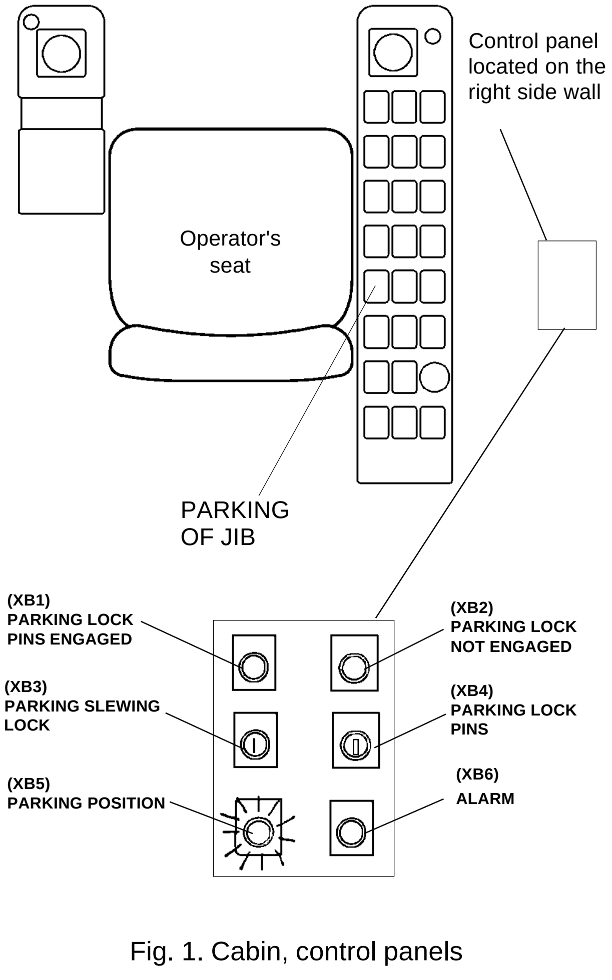

Fig. 1. Cabin, control panels

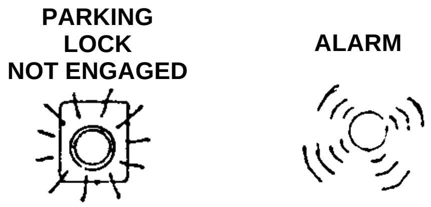

Fig. 2. Indications when parking lock is not engaged

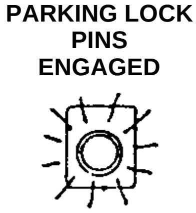

Fig. 3. Indication when parking lock pins is engaged

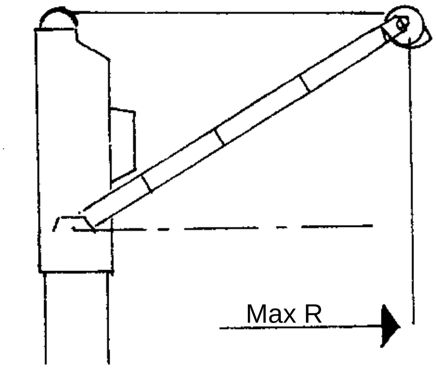

Fig. 4.

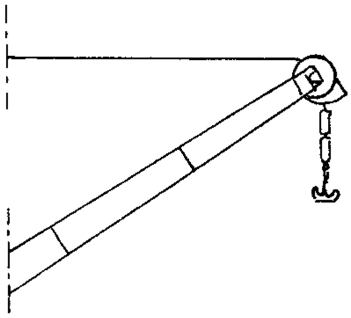

Fig. 5.

Handling Hatches by Deck Crane

Handling Hatches

When pulling hatch covers by means of deck cranes, it is very important that the pulley blocks are so placed that the hoisting wire can be maintained very nearly vertical during operation. The crane jib should be set at an angle where it will not require any subsequent shifting in order to achieve the required lifting height.

The included angle between the hoisting wire and the vertical centre line of the crane house must not exceed 5°.

Always check that the pulley blocks are correctly positioned, so that the hoisting ropes cannot deviate more than 5°, at most, from the crane house centre line.

N.B. The capacity of the wire pulling the hatch cover may limit the force which can be applied by the crane.

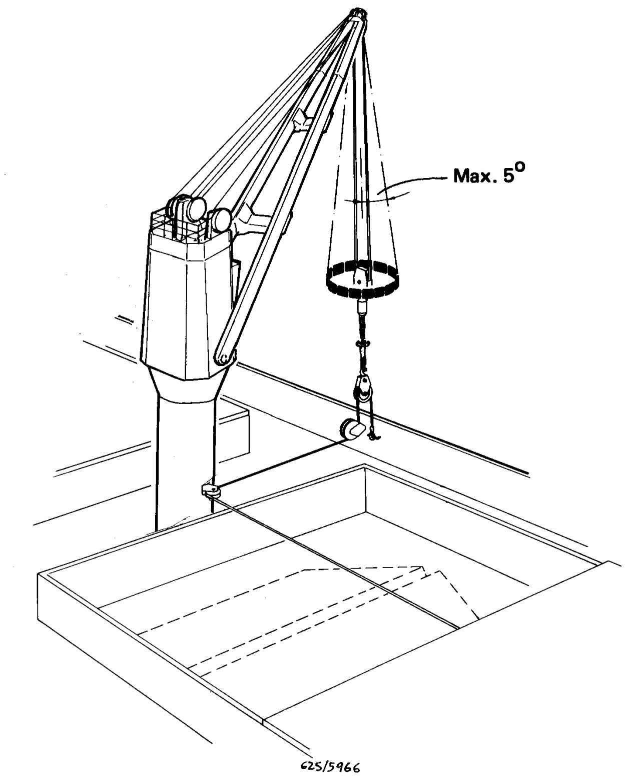

Fig. 1

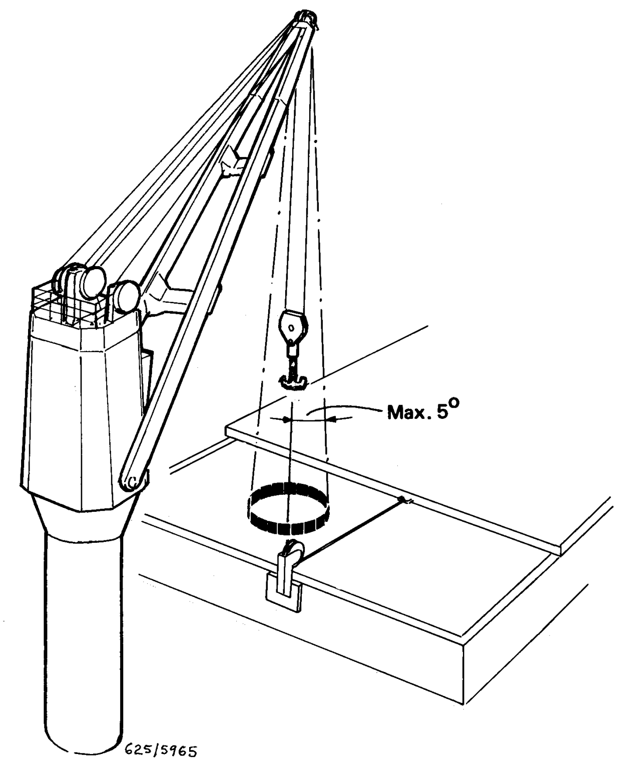

Fig. 2

Maintenance Chart

Lubricate crane according to LUBRICATING CHART.

Daily or before taking crane into operation

Hydraulic oil tank

- Check oil level.

Slewing gear case

- Check oil level. See instruction, section 6.1. The oil quantity is marked on the name plate and in instruction "Technical Data", section 2.

Filter service indicator

- Check filter service indicator
- If necessary change filter cartridges, see instruction "Filters", section 5.

Limit switches; compare section 6.3 instruction for stop position

- Testrun all functions and check that they are working properly.

Slack wire switch

- Function test, see instruction, section 6.3.

Loose gear

- Check that block, beam, hook, swivel and shackle are intact. Check according to instruction "Lifting block", section 6.2.

After 50 operating hours (running-in period)

Oil tank, power swivel (optional)

- The first oil change see instruction, section 7.

Every month

Escape device

- Visual inspections should be carried out with particular attention being paid to the condition of rope and escape harness.

Every 200 operating hours or every two months

Pumps

- Check leakage.

Multiple-disc brake, slewing gearset

- The drain plug shall be taken out every 200 hours of operation, to drain off oil that may have been collected.

Slewing bearing screws

- Inspect visually the slewing bearing screws from deck and inside pedestal. If any screw shows any tendency to slacken, tighten all screws with a torque wrench or a hydraulic tensioner according to drawing "Slewing bearing, yard mounting" and "Slewing bearing, mounting", section 9.3.

Wire rope sockets

- Check that wire rope thimbles are safely secured.
- Check split pin for wear and damage.
- Change if necessary.

After 200 operating hours

Slewing gear case

- The first oil change. (Running-in period 200 hours.)

See instruction, section 6.1.

Every 500 operating hours or every six months

Oil cooler

- Inspect oil cooler externally. Clean when required.

Wire ropes, wire rope clamps

- Inspect for wear. See instruction "Handling, Installation and Maintenance of Steel Wire Ropes" under section 6.2
- Wire ropes are inspected according to stipulations issued by Classification Associations and National Bodies.
- Check that wire rope clamps on wire drums are safely secured.

Wire sheaves

- Inspect for wear.
- Check that shafts are safely secured.

Slack wire switch

- Inspect rollers for wear or damage.

Electrical equipment including slip-ring unit

- Inspect according to “Electrical Equipment, General Maintenance” under section 6.3.

Heating elements, cubicle MB

- Check that the elements in cubicle MB is working i.e. gets warm.

Cranehouse, foundation and jib welding joints

- Inspect for any sign of crack.
  See "Inspection of Stressed Components and Structures", section 5.

Hydraulic system

- Measure the pressures according to the hydraulic circuit diagram’s adjoining list, section 9.4.
- Reset as required.

Oil analysis (oil tank)

- Sample oil and analyse every six months.

Pump servo

- Check plussing
- If necessary adjust.

Slewing gear, pinion

- Check backlash.

Slewing bearing

- Check seals.

Filter service indicator

- Change filter cartridge. See instruction "Filters", section 5.

Slip-ring unit

- Check brush holders and wire brushes according to instruction under section 6.3.

External acess ways

- Inspect for wear and damage.

Emergency escape ladder (optional)

- Inspect for wear and damage.

Every 1000 operating hours or at least every year

Escape device

- The device should be inspected by competent person or returned to MacGREGOR Cranes for servicing.

Slewing gear case

- Change oil. See instruction, section 6.1.

Air breather, hydraulic oil tank

- Check and change air breather on top of the tank

Hydraulic accumulators

- Check pressure, re-charge as required. See instruction “Hydraulic Accumulators”, section 6.1.

Cable winch (optional)

- V-belt, check tension
- Slipring unit, check
- Brake discs, check
- Brush holders, check

See instruction, section 7.

Every 2000 operating hours or every two years

Hydraulic oil tank

- Take out the drain plug
- Drain oil and clean the reservoir. (See “Hydraulic oil tank”, section 5.)
- Verify that the oil level float switch is working properly. This inspection is verified when changing oil or cleaning of oil tank
- Check air breather cap
- Top up with fresh oil according to “Lubricants for deck machinery”, section 5
- Change all oil filter cartridges.

Multiple disc brake, winch

- Check for leakage
- Check braking torque
- Check according to instruction "Winch" under section 6.1.

Multiple-disc brake, slewing gearset

- Check for leakage
- Check braking torque
- Check discs
- Check according to instruction "Slewing gearset", section 6.1.

Oil tank, cable winch (optional)

- Drain oil, turbo coupling
- Drain oil, spur gear

See instruction, section 7.

Oil tank, power swivel (optional)

- Drain oil and clean the reservoir. See instruction, section 7.

Every four years

Slewing bearing studs/screws

- Tighten all studs or screws with a hydraulic screw tensioner or a torque wrench. Tightening torque, see separate drawing, "Slewing bearing, yard mounting" and "Slewing bearing, mounting", section 9.3
- Consult MacGREGOR Cranes before replacing studs or screws with any other specifications than recommended.

Inspection of Stressed Components and Structures

Introduction

Stressed parts of the crane structure subjected to high loads require inspection for signs of cracks and other damage at regular intervals.

Cracks detected at an early stage do not generally impair the handling safety of the crane unless they are not repaired.

For safety reasons, service routines should include a regular inspection of the entire crane, with particular attention paid to stressed parts.

Inspection

A thorough visual inspection is fairly easy to carry out as cracks will generally show by cracking or flaking of the paint followed by a brownish discoloration by rust.
(Inspect welds and surrounding material.)

This inspection should be performed at intervals according to the Maintenance Chart.

Note!
Rust on a painted surface does not necessarily mean that there is a crack in the underlying material.

Remedy

When rust is found on top of or near a weld, grind the topmost surface layer away to expose the crack clearly. When in doubt, use Magnaflux to identify the crack.

Before repairing of a crack in a stressed part, always contact MacGREGOR Cranes, Service Department for advice.

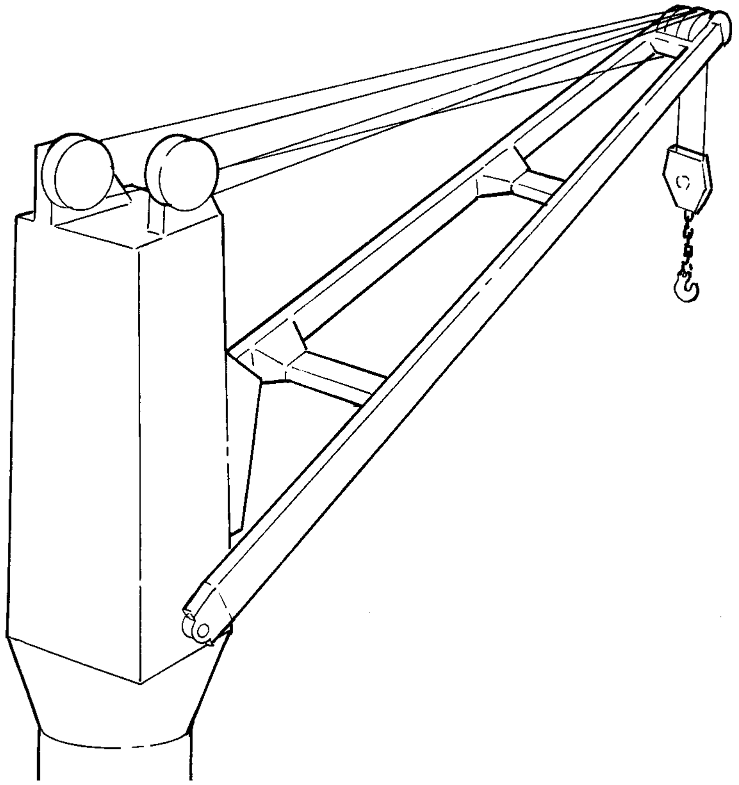

Fig. 1. Crane Structure.

Item	Part of crane (see page 2 for location)	a	b	c	d	e	f	g
1	Wire sheaves, Crane house top: 1 nipple/sheave (5-8 nipples) *				△			
2	Wire sheaves, Jib stay: 1 nipple/sheave (1-2 nipples)* (not on all cranes)				△			
3	Wire sheaves, Jib top: 1 nipple/sheave (4-9 nipples) *				△			
4	Wire sheaves, Lifting block: 1 nipple/sheave (1-2 nipples) *		△					
5	Wire rope sockets: 2 pcs (in some cases on jib or block) ***		▲					
6	Swivel, Lifting block (Single) or Lifting beam (Twin): 1 nipple		△					
7	All wire ropes				□			
8	Electric motor: 2 nipples				△			
9	Door hinges: 3 nipples				▲			
10	Jib bearings: 2 nipples			☆				
11	Oil tank	◒					⌫	
12	Air breather cap					⊙		
13	Oil filters	⊕						
14	Slewing gear case	◒				■		
15	Slewing bearing teeth			◇				
16	Slewing bearing - See drwg. section 9.3 for nipples **		△					
17	Hydraulic motors, Axial thrust bearing (not on motor CA420-400)							◁■
18	Parking cylinders: 4 nipples (optional)			△				

* Check what number of nipples that apply to your crane. It is of utmost importance that all nipples are greased.

** Relubrication should be performed under slewing motion. If that is not possible relubricate bearing through all grease fittings, than turn bearing approx. 200 mm and relubricate again. Repeat this procedure until overlap of greasing is achieved and the whole bearing circumference is filled with new grease at the gaps and seals. (Five rotations/ greasings should be sufficient.)

*** **Before greasing proceed as follows**
Crane jib parked on parking support:
Park the crane and slack the luffing and hoisting wire.

Crane jib parked in wires:
Hoisting wire - slacken the wire by lowering the lifting block to deck.
Luffing wire - run the block up against the jib top to slacken the luffing wire.

Key to symbols

- a Daily
- b Every 100 working hours
- c Every 200 working hours or every two months
- d Every 500 working hours or every six months
- e Every 1000 working hours or every year
- f Every 2000 working hours or every two years
- g Every five years

Key to symbols

- △ Grease for ball and roller bearings
- ▲ Grease for sleeve bearings
- □ Grease for wire ropes
- ◇ Grease for open spur gears
- ☆ Grease for jib bearings
- ◒ Oil level, check
- ⊕ Oil filters, check
- ⊙ Air breather cap, check
- ⌫ Hydraulic oil, oil change
- ◁■ Hydraulic oil change
- ■ Gear case oil, oil change
  The first oil change to be done after 200 working hours (running-in period)

Documents with supplementary data

- Oil quantity required:	"Technical data", Section 2.
- Maintenance intervals:	"Maintenance chart", Section 5.
- Location of nipples etc:	"Lubricating and maintenance", Section 5.
- Lubricants:	"Lubricants for deck machinery", Section 5.

Red lamp on right control panel in operator´s cab

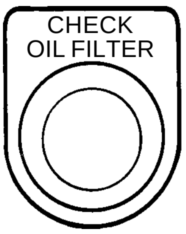

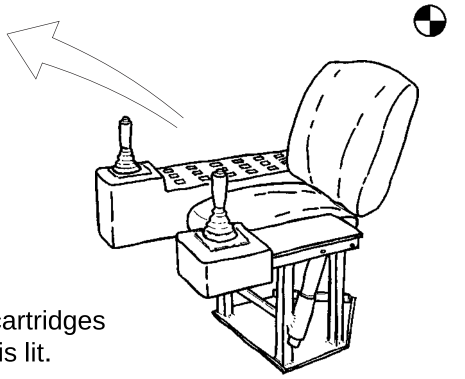

CAUTION!
Renew all filter cartridges when this lamp is lit.

Jib top with lifting block for single crane

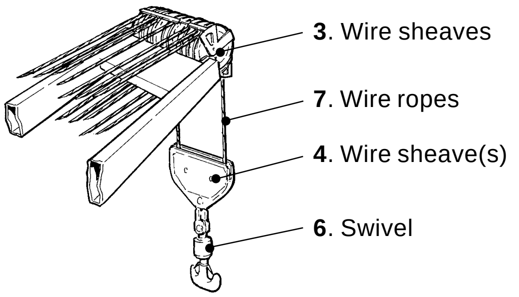

The lifting block can have either 1 or 2 wire sheave(s).

Wire sheave(s) on jib stay for cranes with special design

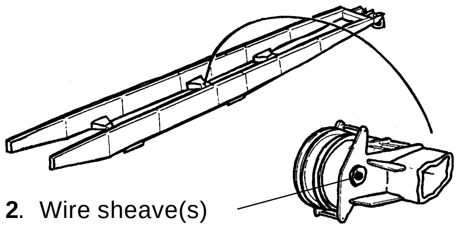

Lifting beam with hook for twin cranes

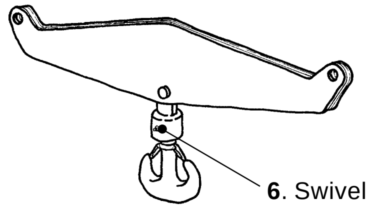

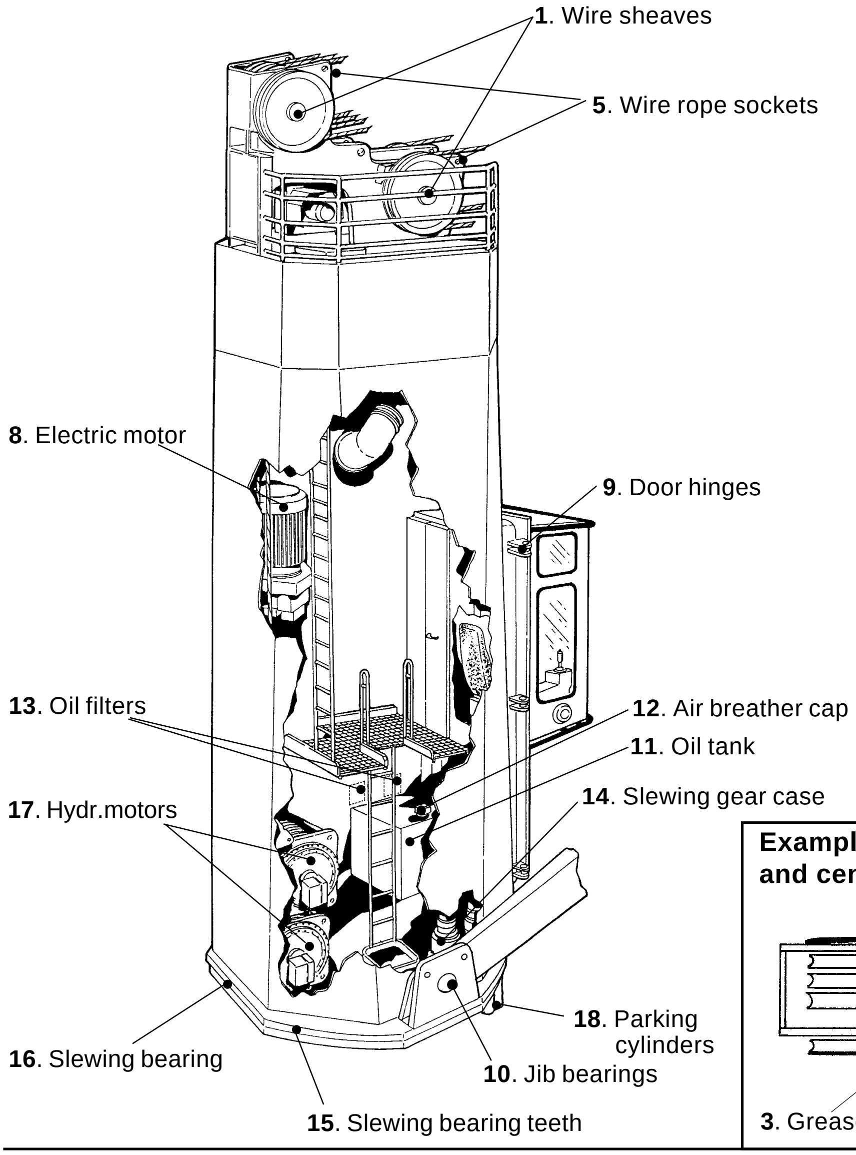

Central greasing, see examples below:

Example: Wire sheaves jib top, central greased.

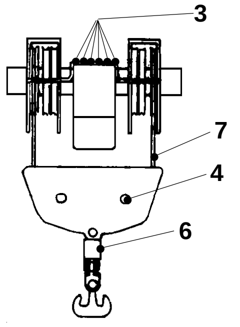

Example: Wire sheave(s) on jib stay central greased.

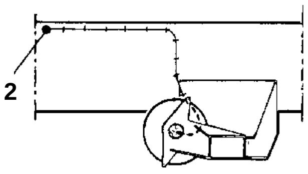

Example: Wire sheaves crane top, manual and central greased.

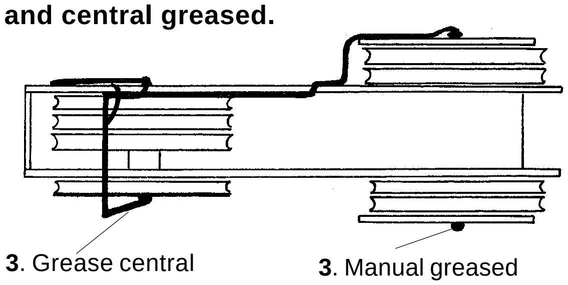

Lubricating and Maintenance
Cranes type GL

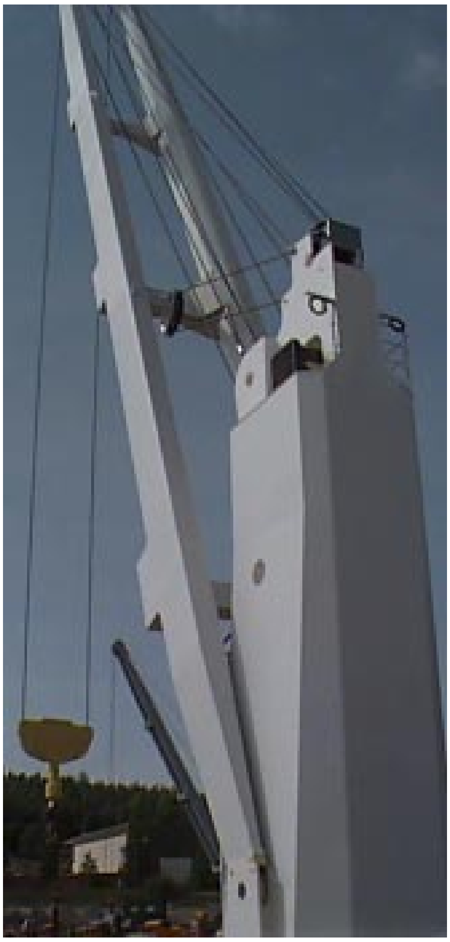

Contents

Door hinges 2
Electric motor 3
Slewing bearing 4
Jib bearing 5
Wire sheaves Jib 6
Wire sheaves Crane house 7
Wire rope sockets 8
Slewing gear case 9
Lifting equipment 10
Oil tank 11
Oil filter 12
Oil filter change 13
Wire ropes 14
Winch with high speed motors 15
Parking cylinders (optional) 16
Cable winch (optional) 17
Power swivel (optional) 18

Door hinges

Grease nipple;
Service door

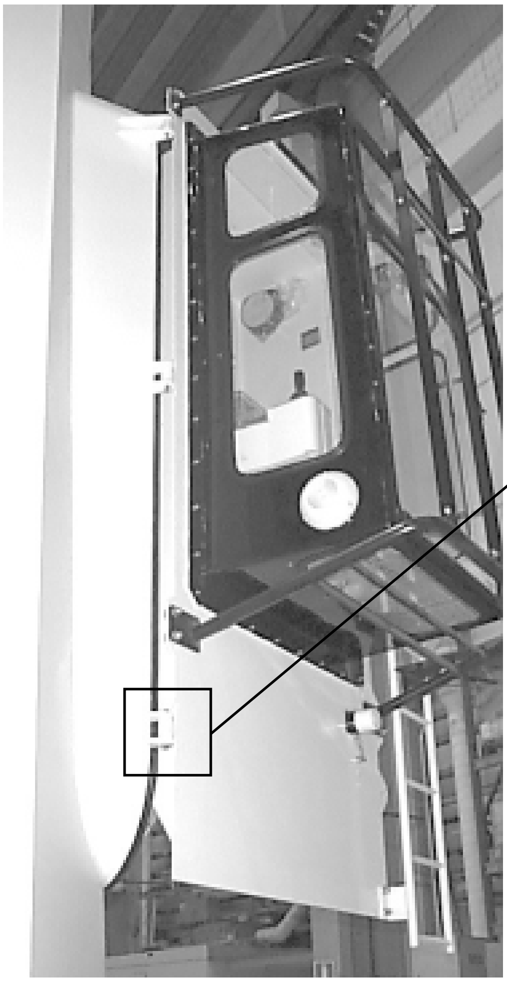

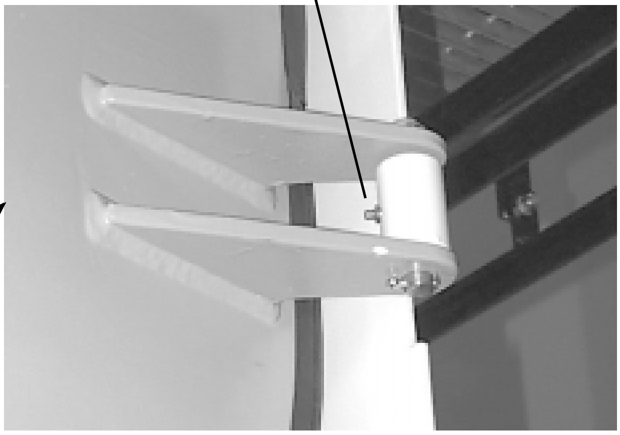

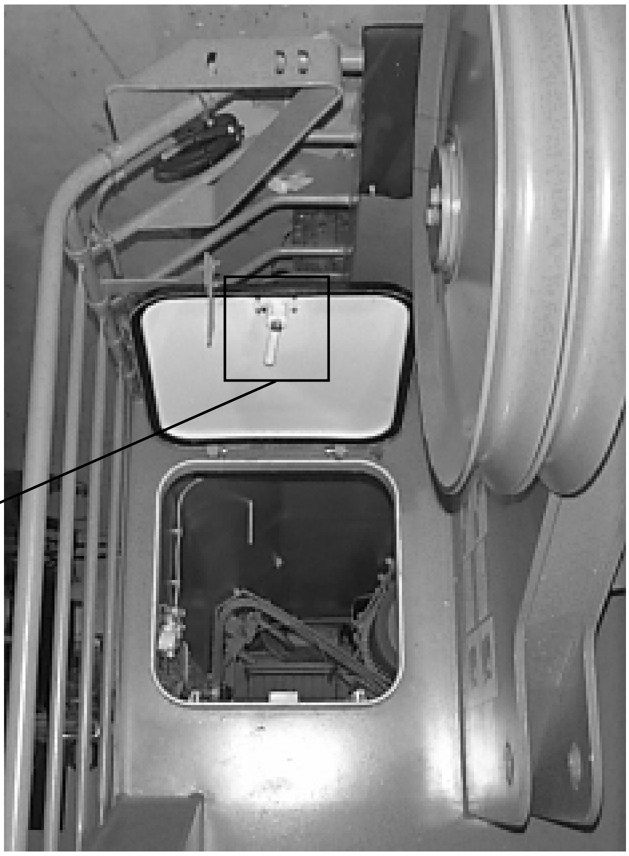

Grease nipple;
Door in crane house top

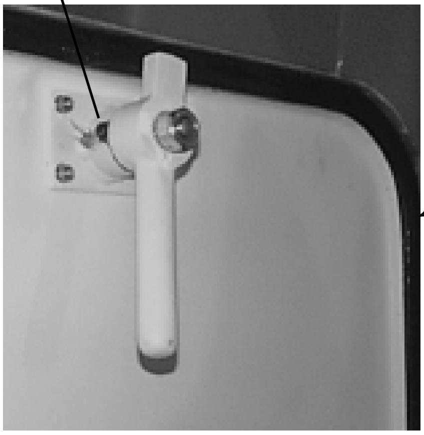

Electric motor

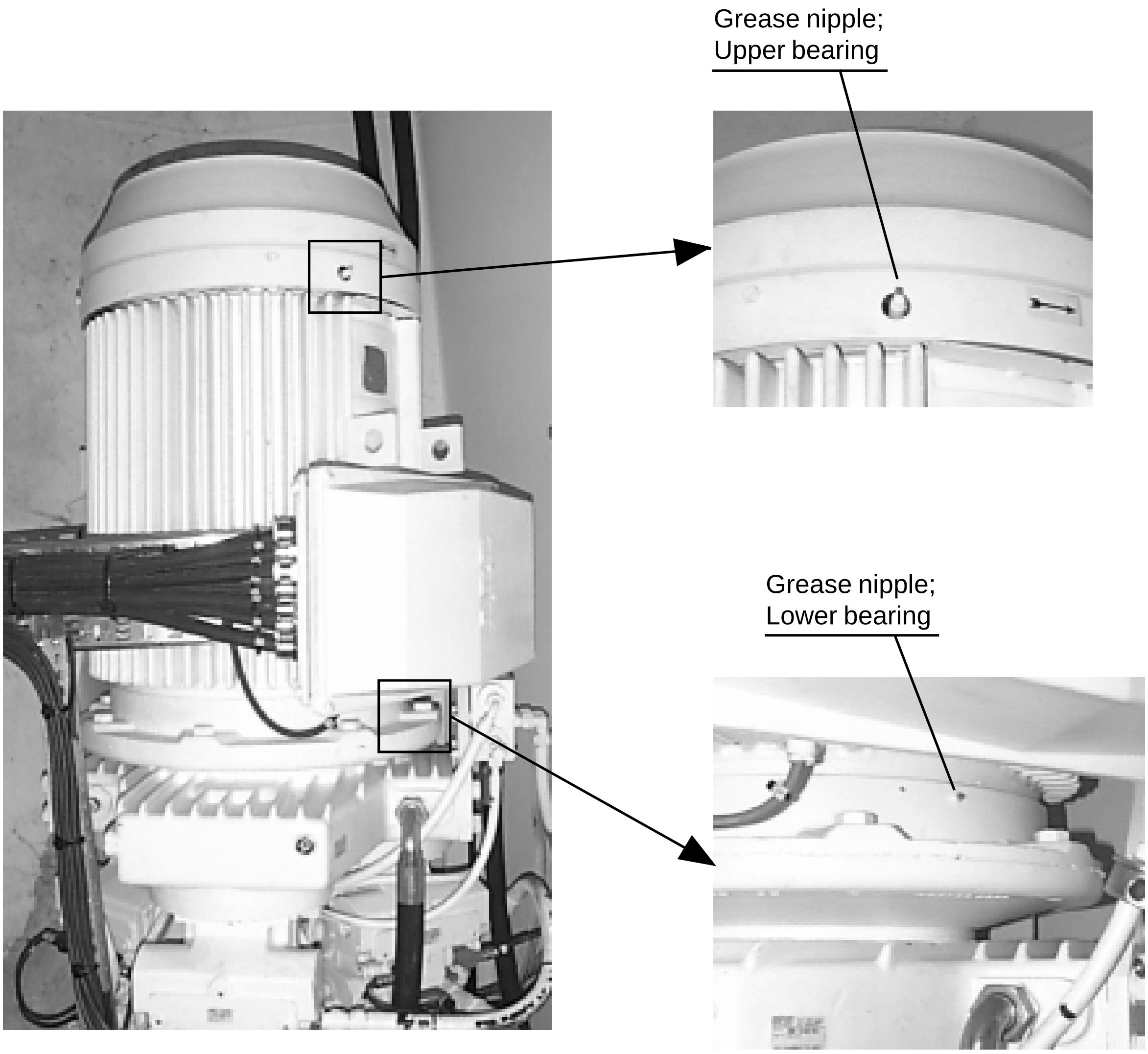

Slewing bearing

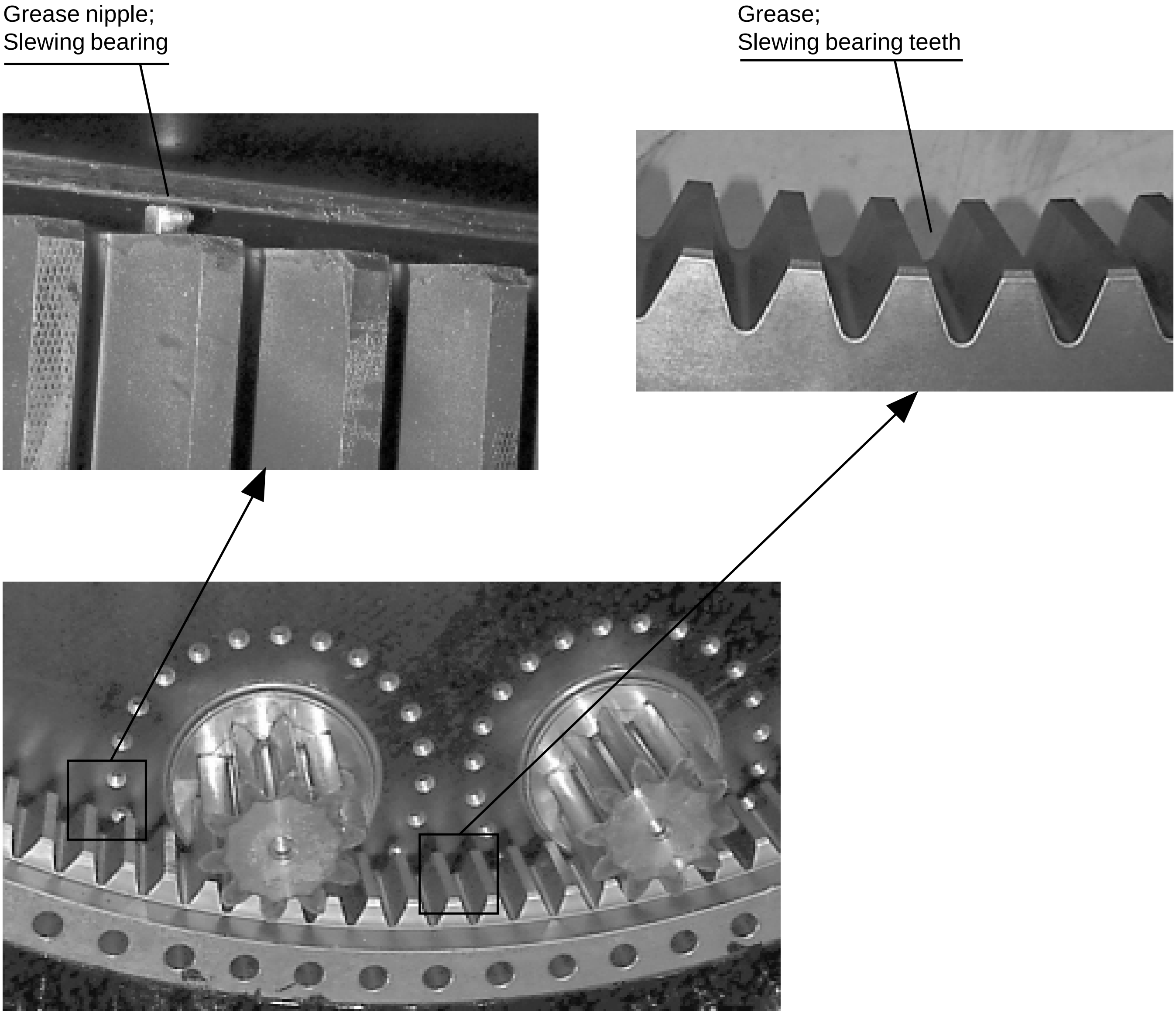

Jib bearing

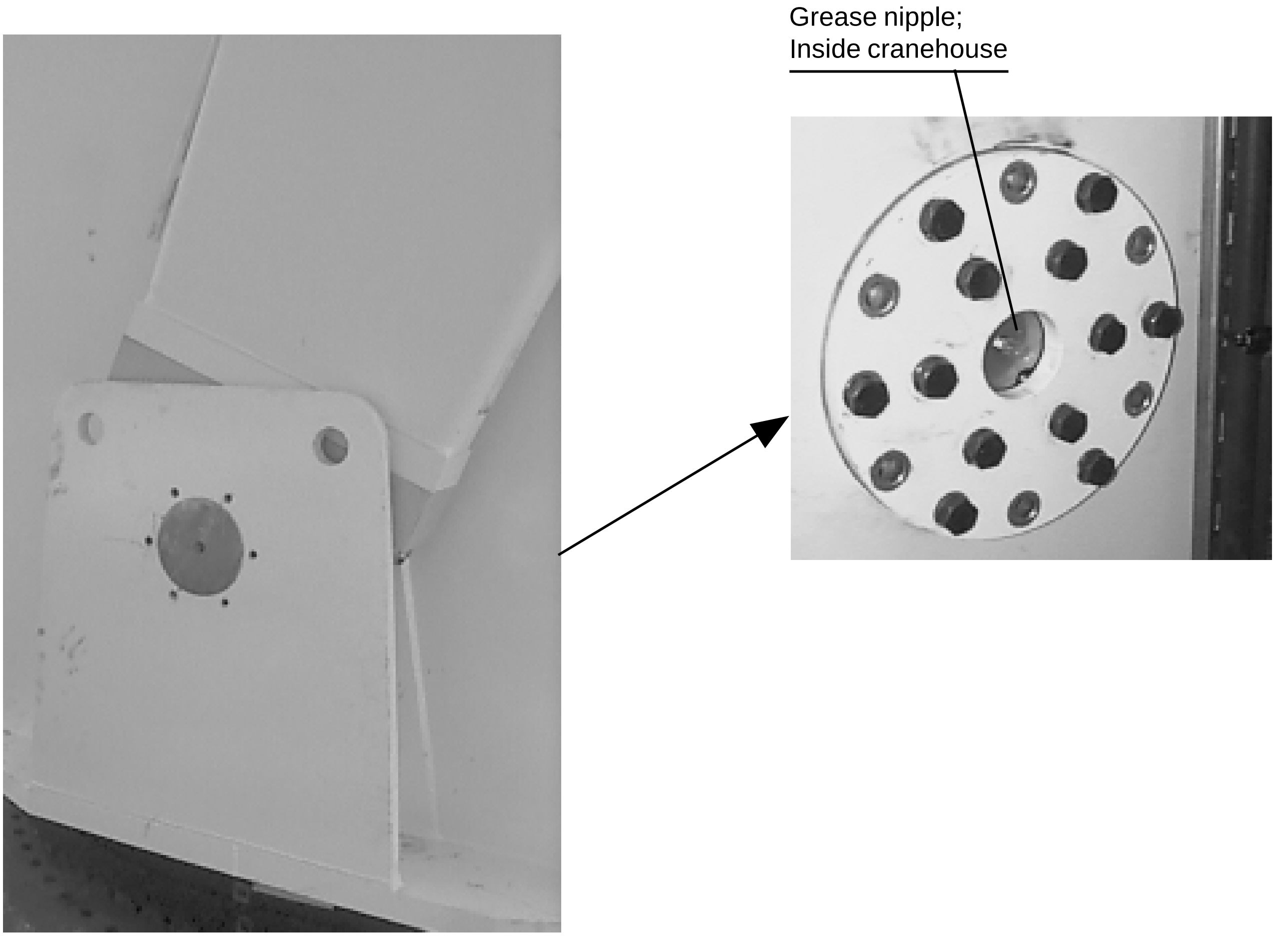

Wire sheaves Jib

Grease nipples; Jib top

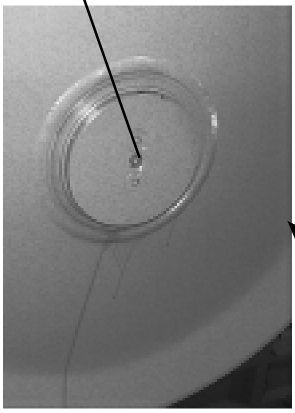

Grease nipples; Jib top, Central lubricating

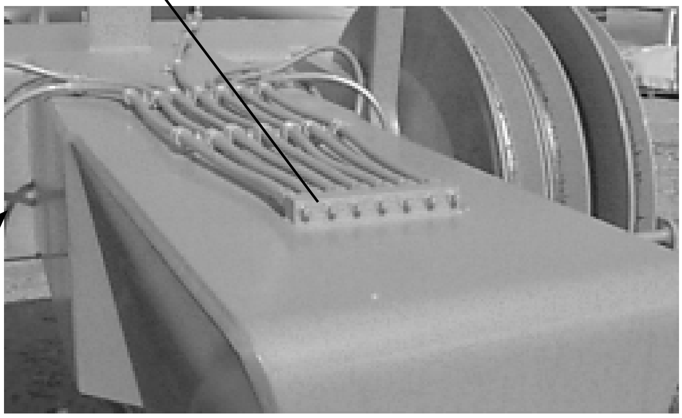

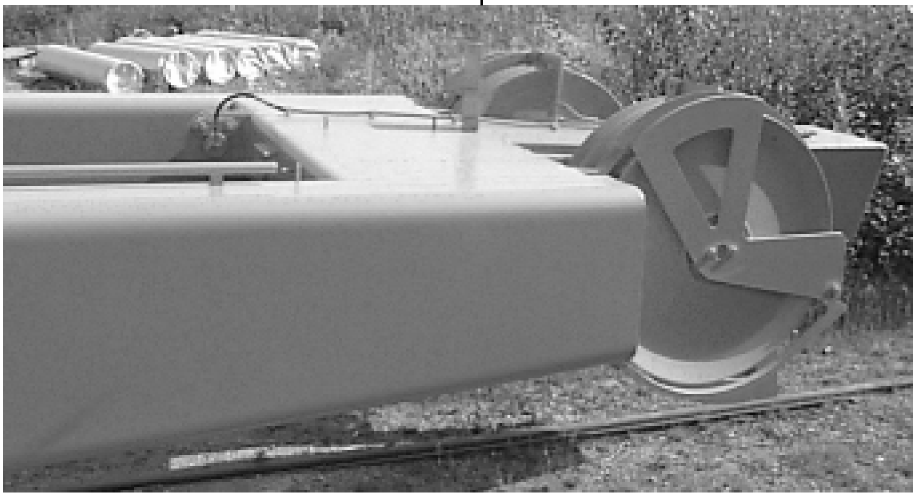

Grease nipple;
Jib stay

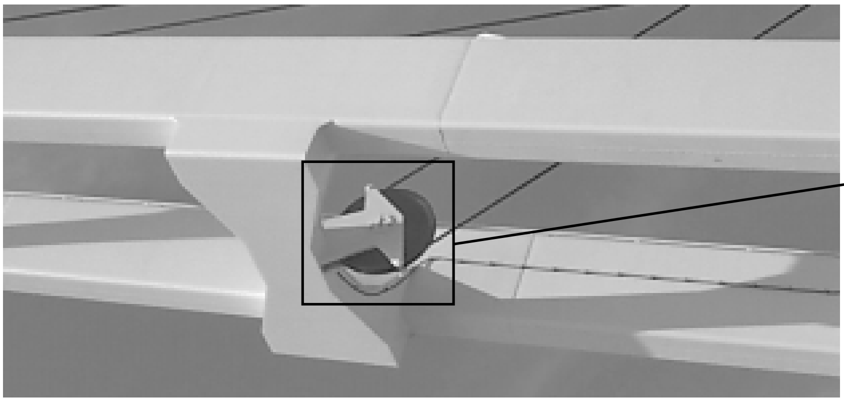

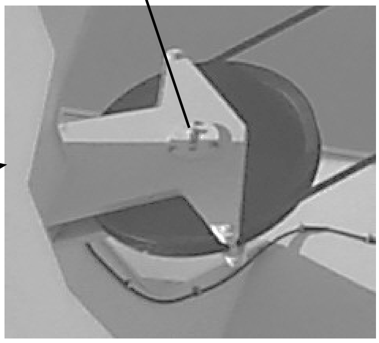

Wire sheaves Crane house

Grease nipple for one sheave

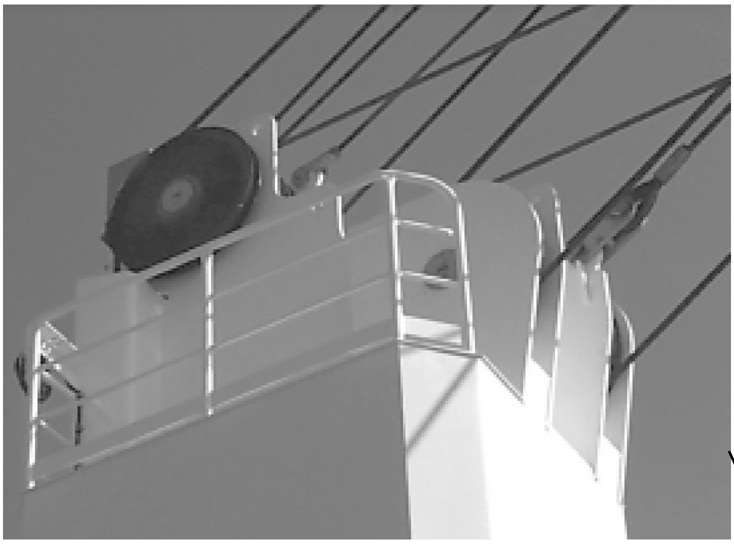

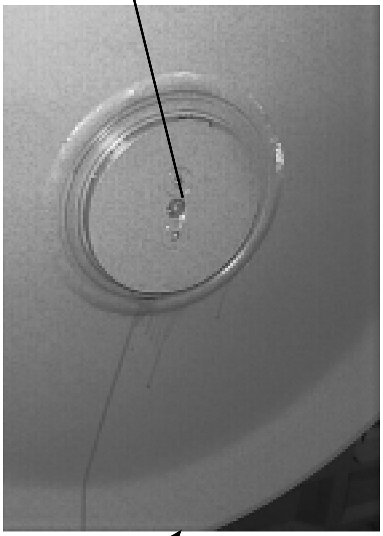

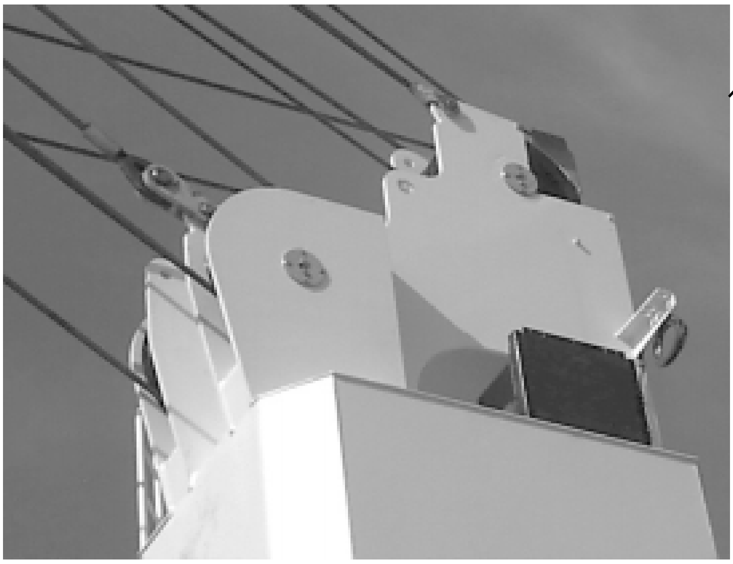

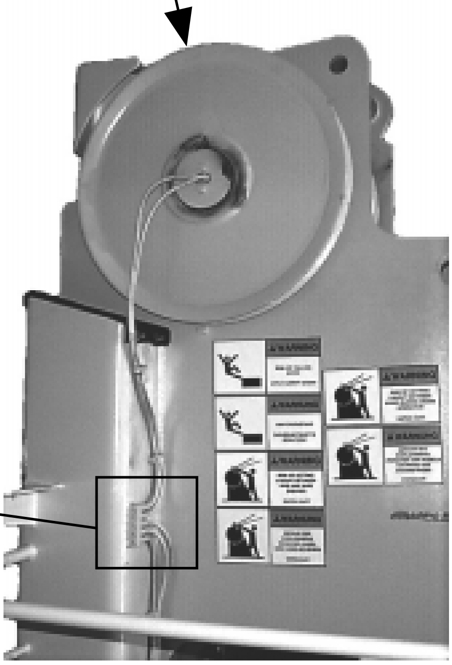

Grease central for several sheaves

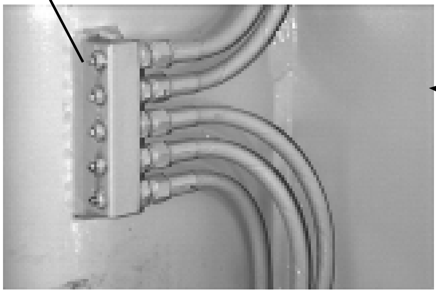

Wire rope sockets

The wire rope sockets can also be located on jib top or lifting block.

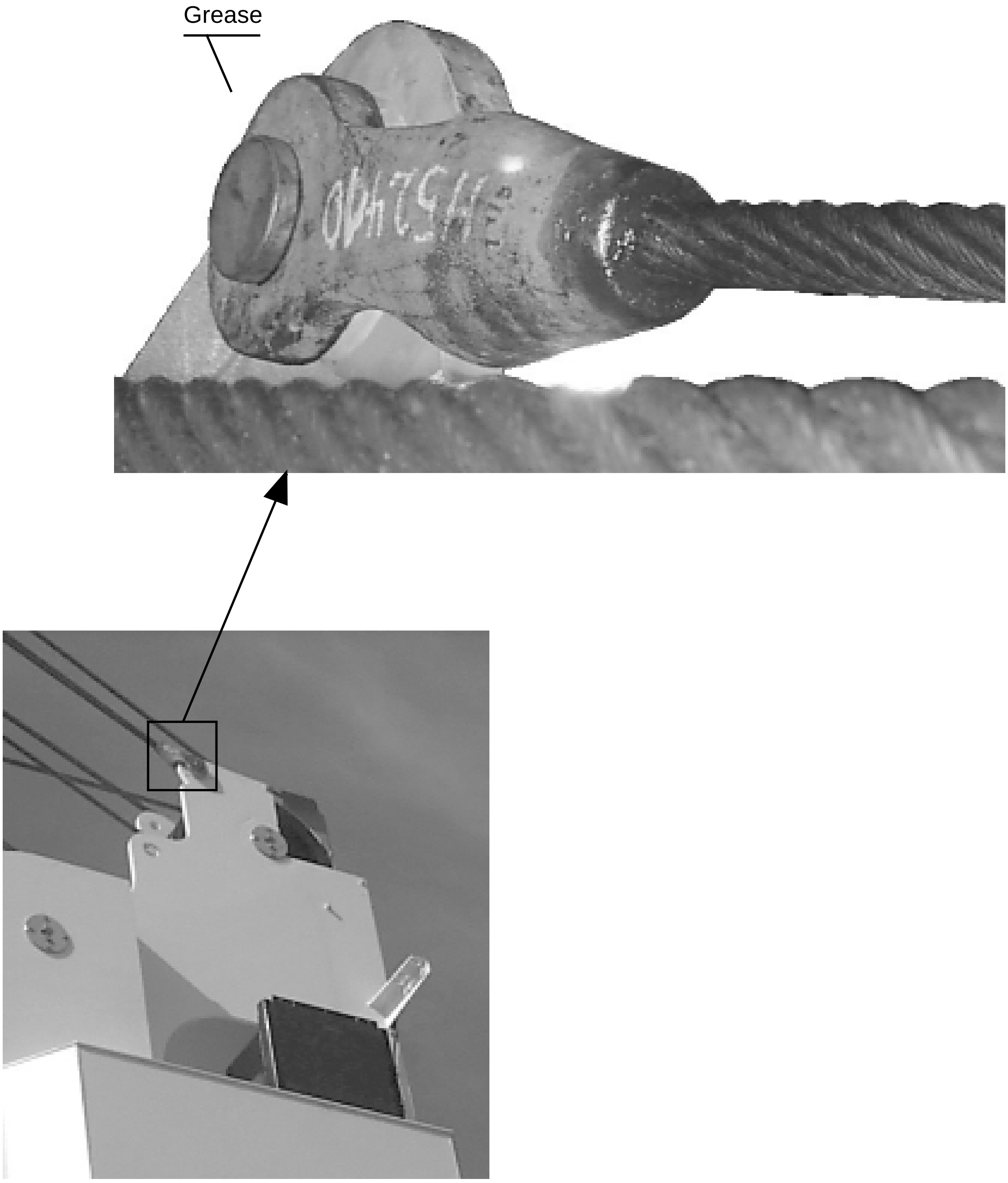

Slewing gear case

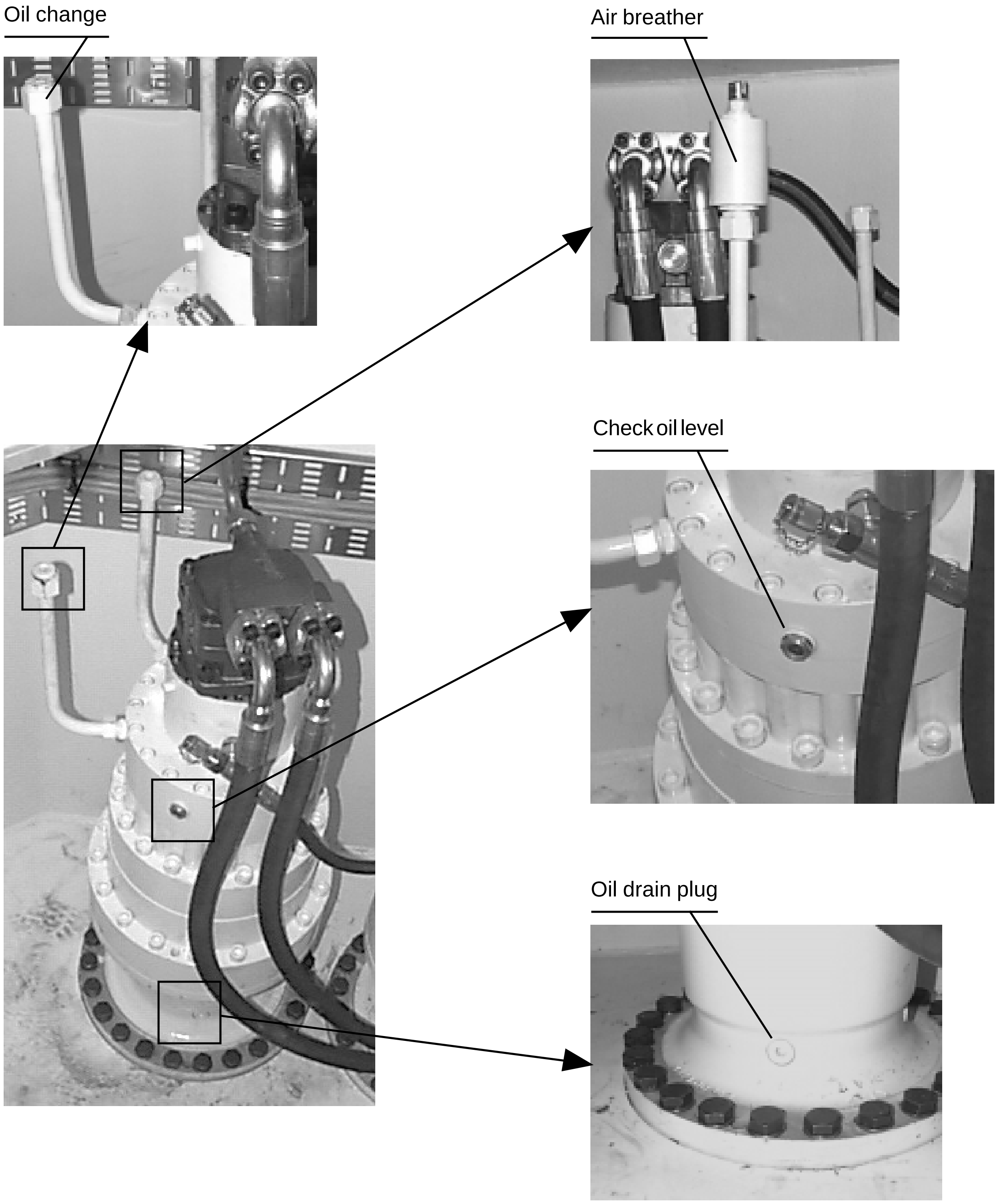

Lifting equipment

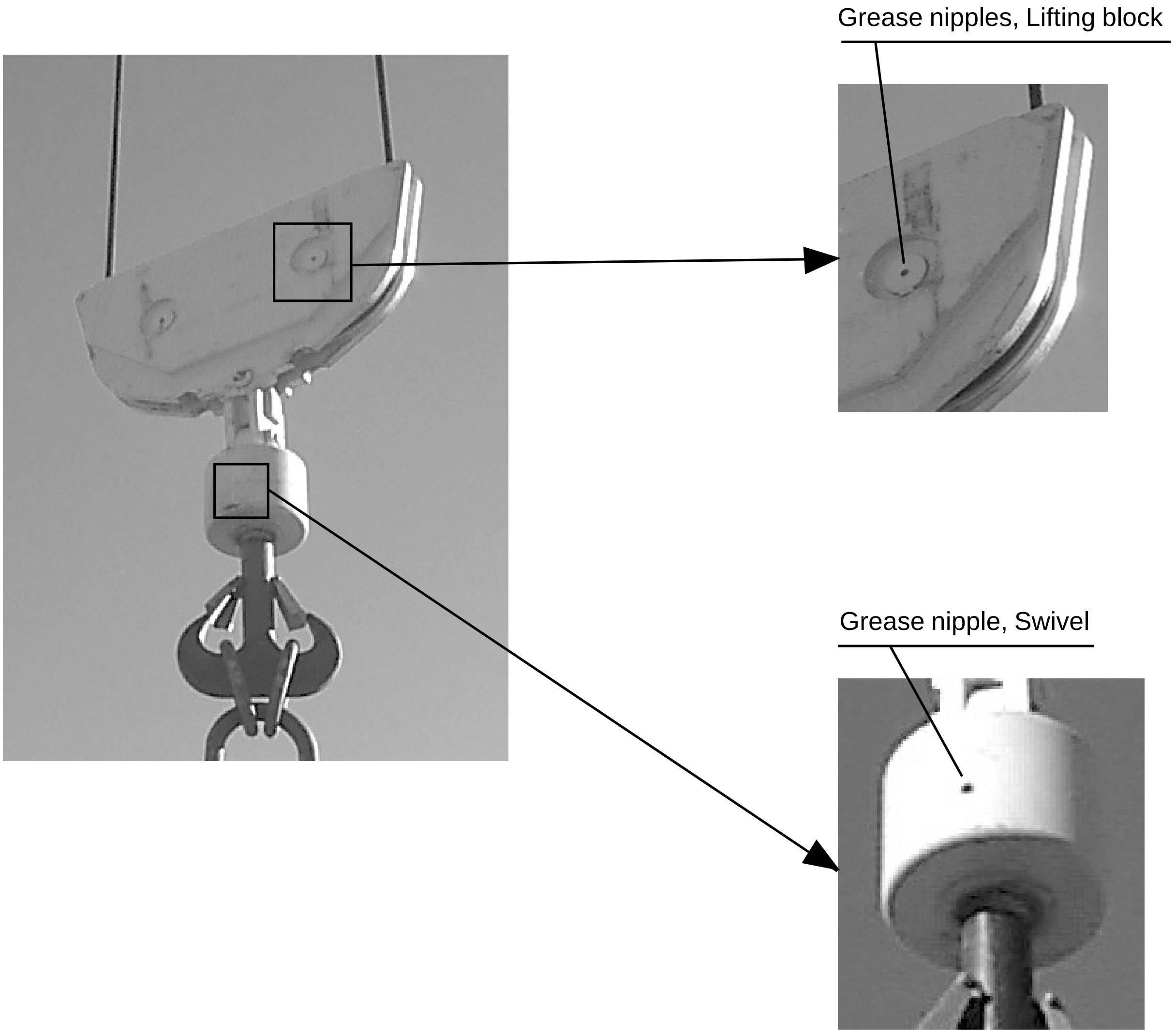

Oil tank

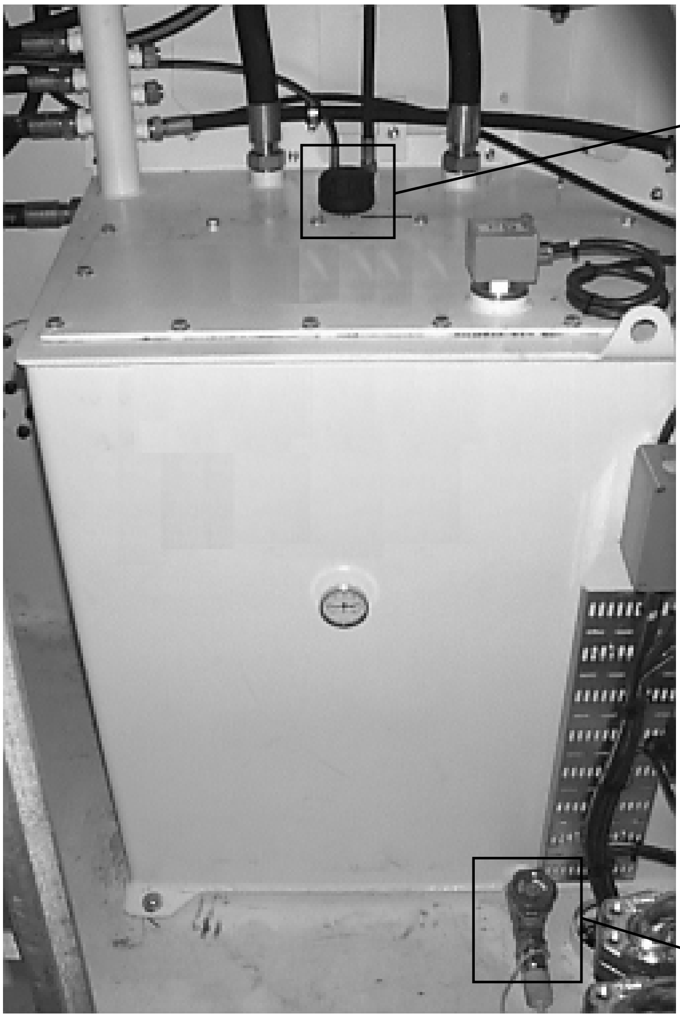

Check air breather
Check oil level (dipstick)

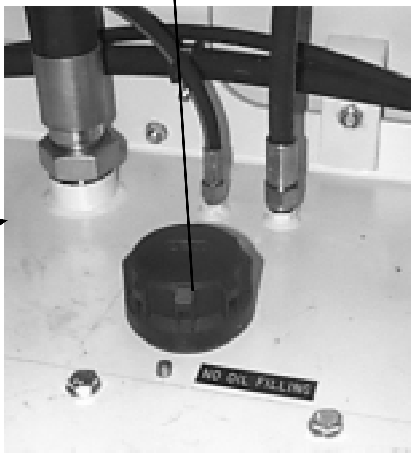

Drain oil tank

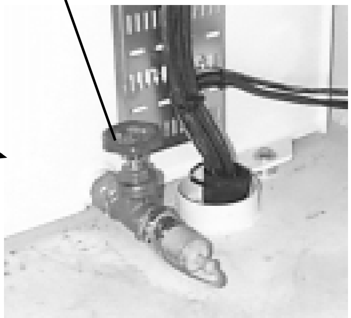

Oil filter

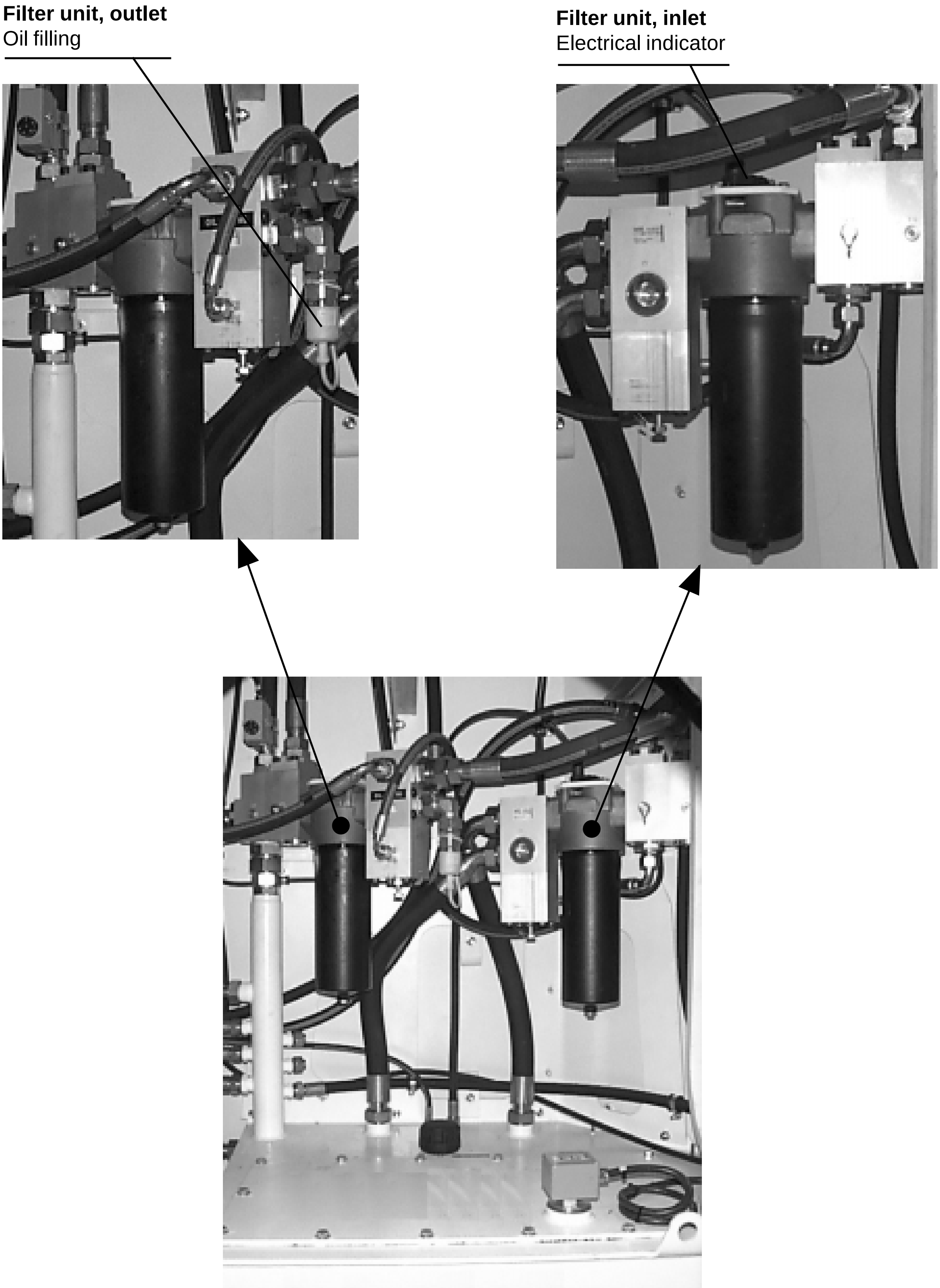
Filter unit, outlet
Oil filling
Filter unit, inlet
Electrical indicator

Oil filter change

Necessary equipment when changing filter cartridges:
- 2 new filter cartridges with O-rings.
- Spanner.
- Bucket.

1 Put the bucket under the filter unit. Loosen the drain-plug at the bottom of the filter unit using the spanner. (size 9/16" or 15 mm)

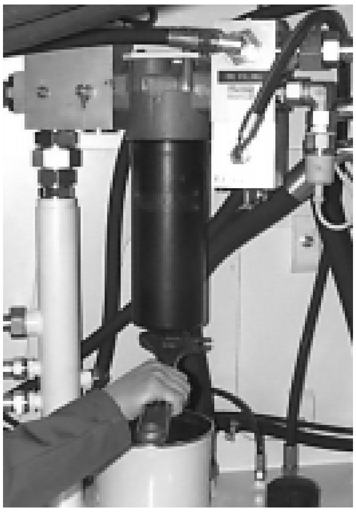

2 Remove the drain-plug by hand and let the oil run into the bucket. Make sure not to lose the O-ring.

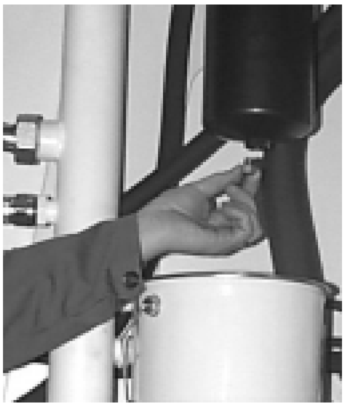

3 Loosen the filter cap using the spanner. (size 1" or 26 mm)

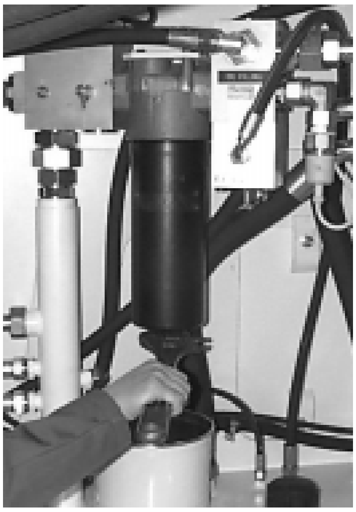

4 Unscrew the filter cap by hand. Be careful not to drop it. Remove the old O-ring between the filter cap and the filter head.

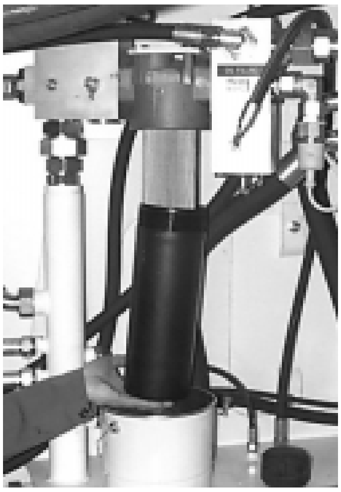

5 Remove the old filter cartridge. Install the new O-ring between the filter cap and the filter head. Install the new filter cartridge.

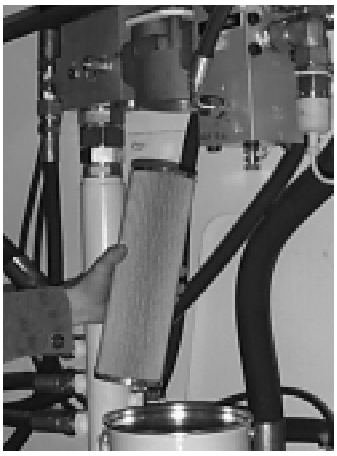

6 Mount the filter cap and tighten it using the spanner.

7 Mount the drain-plug and the O-ring on the filter cap and tighten it using the spanner.

Wire ropes

Relubricating of steel wire ropes, see instruction 6.222.1 E.

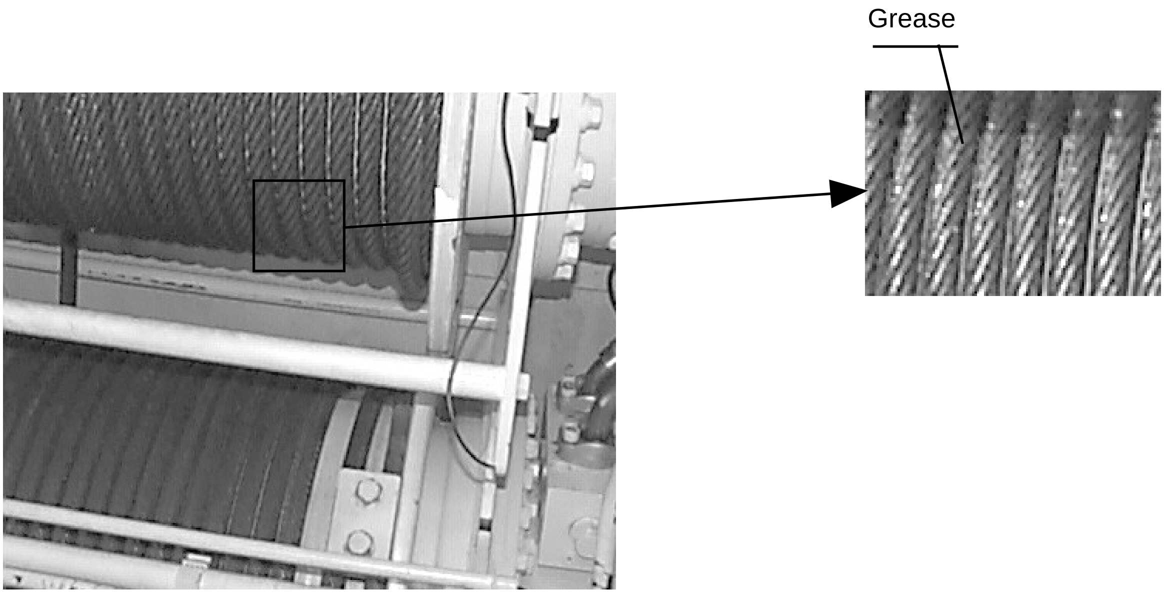

Winch with high speed motors

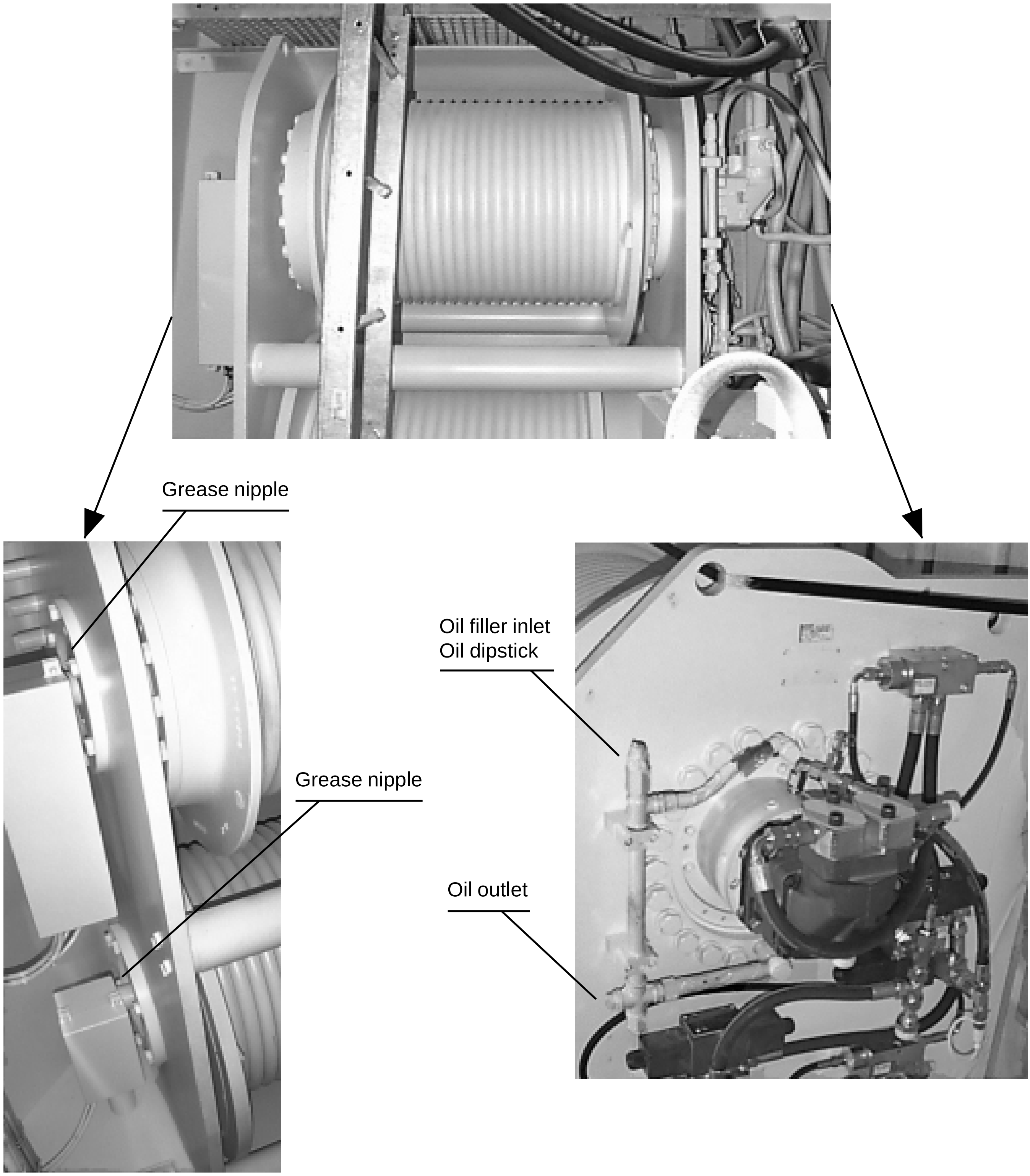

Parking cylinders (optional)

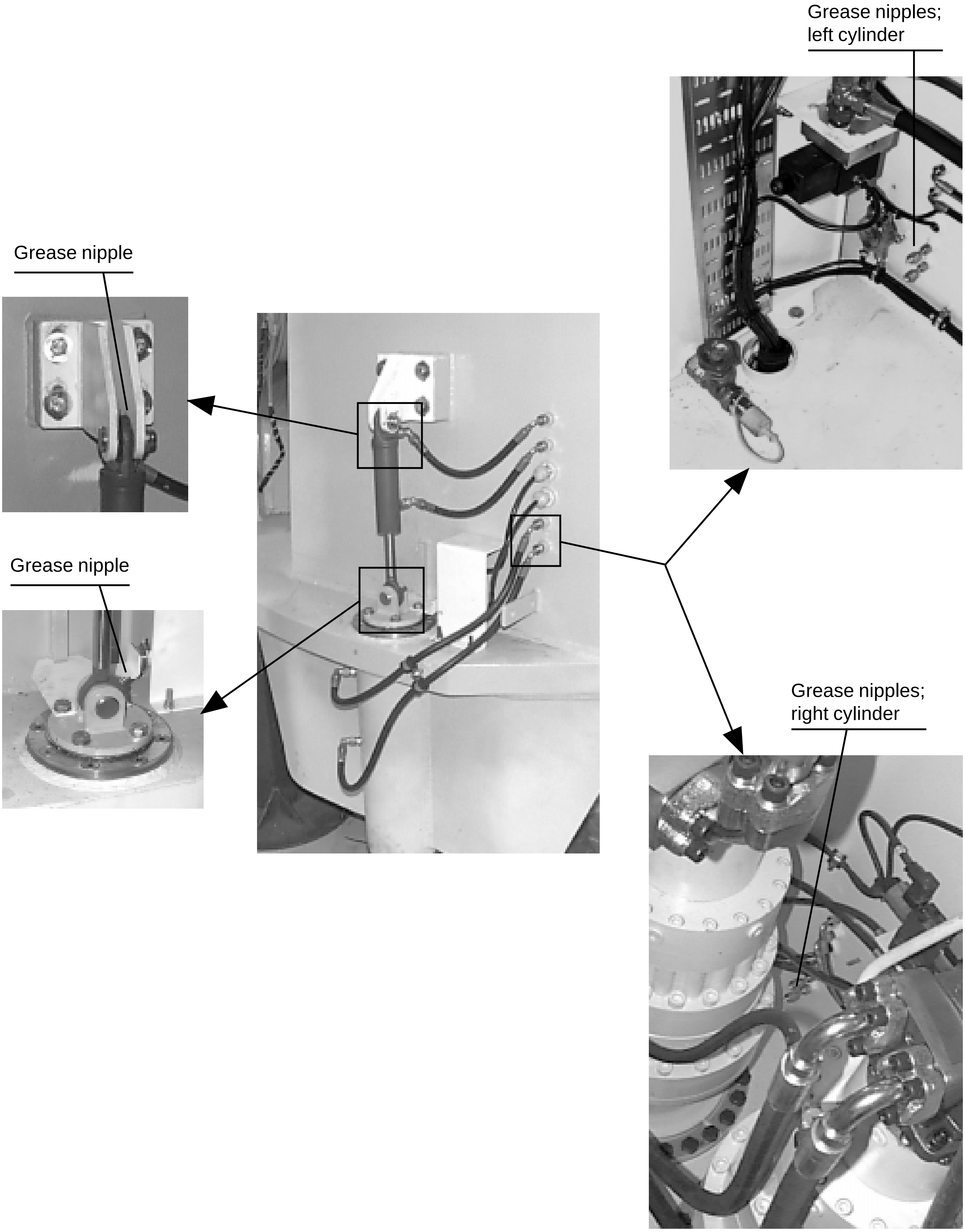

Cable winch (optional)

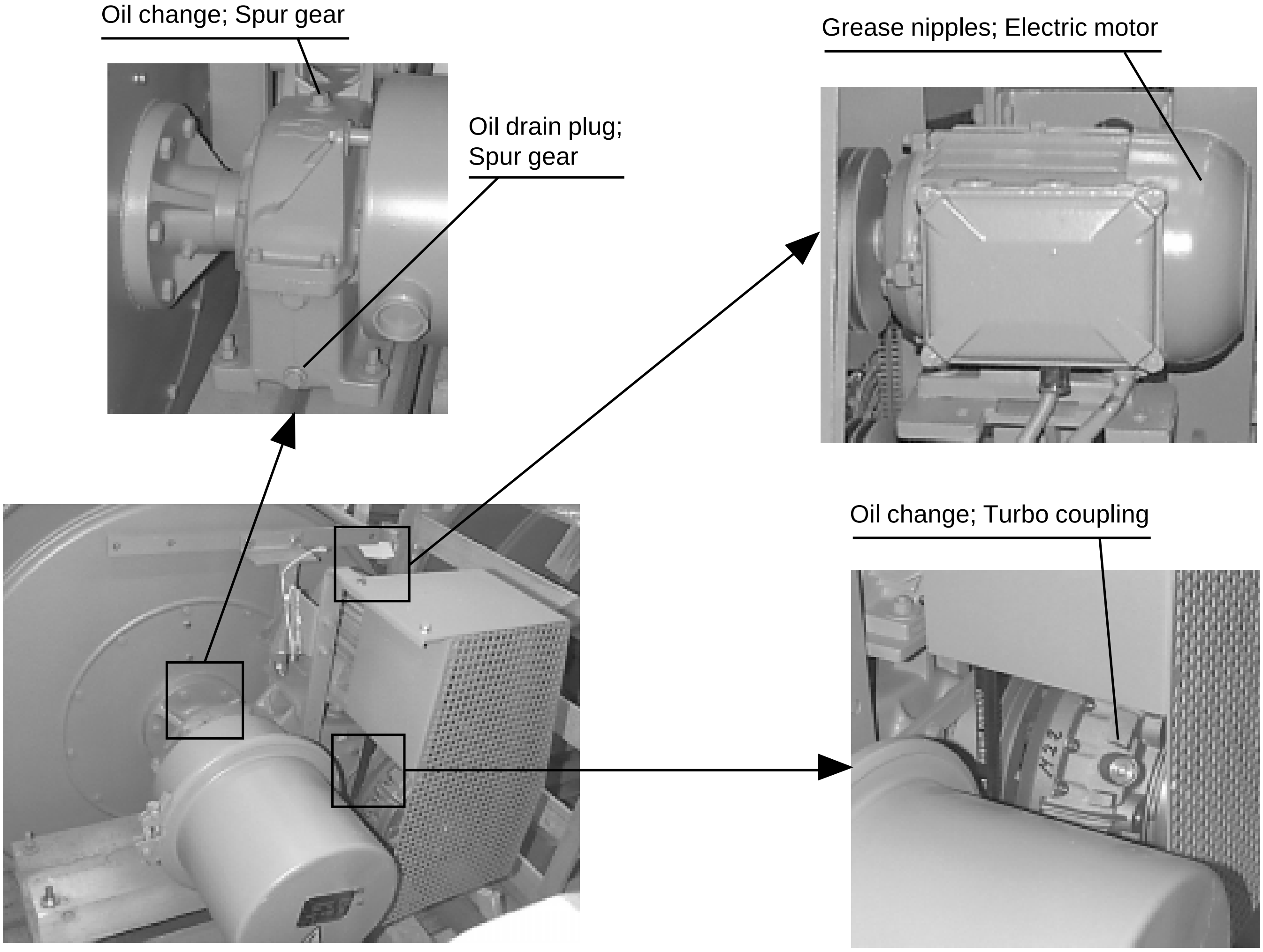

Power swivel (optional)

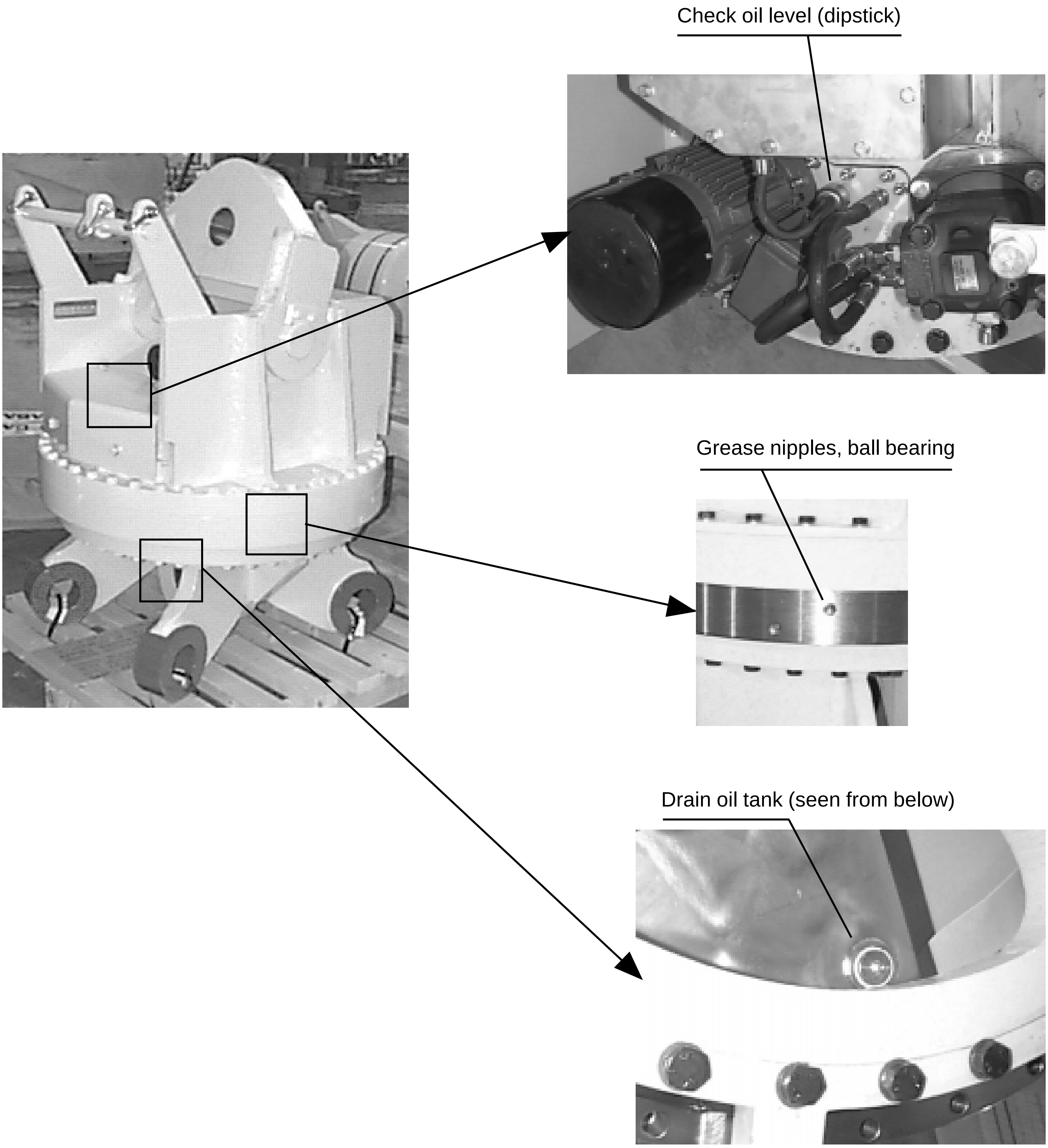

Filters

Introduction

For cleaning hydraulic oil, the hydraulic system is equipped with two filters. The two filters are marked 4135 and 4136.

Filter Indicators

The filter indicators function only when the crane is started.

Filter 4135 is equipped with two filter indicators. One indicator is electric and activates a warning lamp placed on the control panel in the cab (see Fig. 4).

The other indicator is visual (see Fig. 1) and should be read when the oil temperature is approx. 45°C. Upon reaching a certain degree of clogging, a red indication can be seen on the top of the filter indicator. Replace both filter cartridges in the system when the red indication shows.

Replacing Filter Cartridges

Both cartridges (see Fig. 3) in the crane should be replaced at the same time. Flush all other parts of the filters with white spirit when changing cartridges. Use new seals. Reassemble the filters.

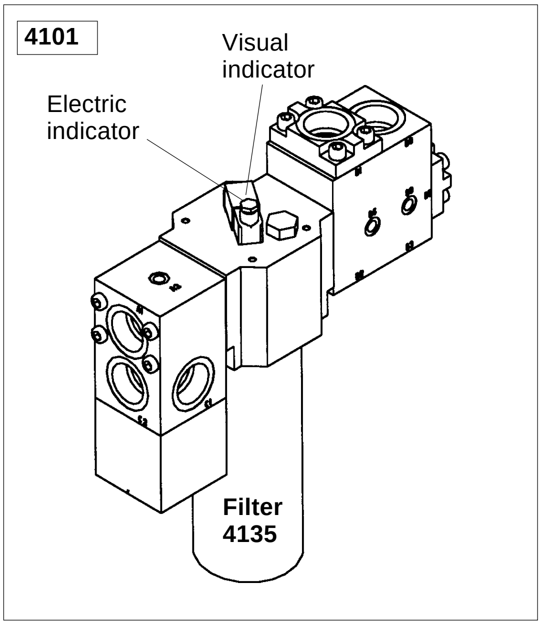

Fig. 1. Filter inlet

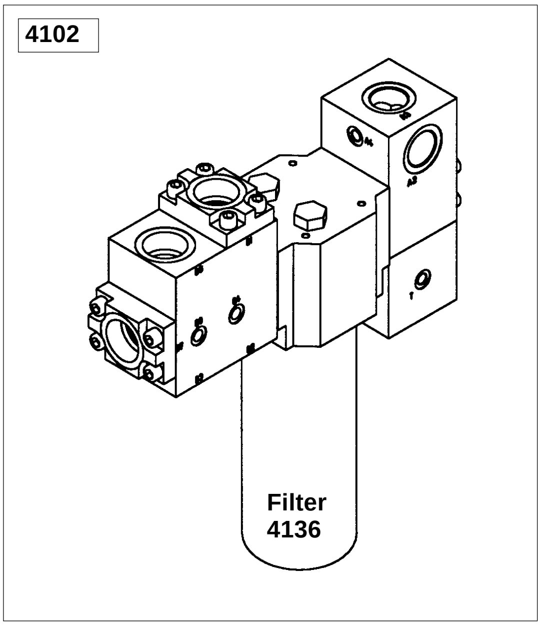

Fig. 2. Filter outlet

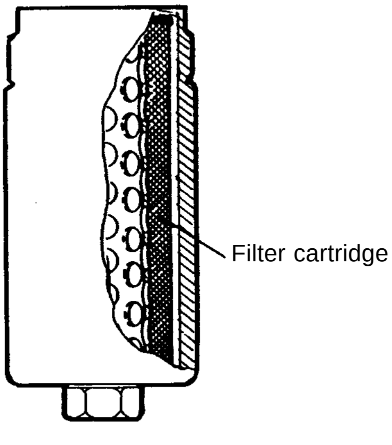

Fig. 3. Filter cartridge

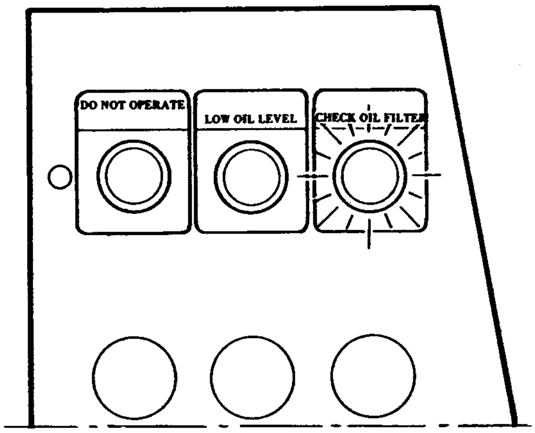

Fig. 4. Control panel

Hydraulic Oil Tank

The hydraulic oil tank is placed on the crane house floor. The feed pump is located inside the oil tank. The main pump unit drive gearing is continually lubricated by oil from the hydraulic system, surplus oil being returned to the tank by a drain line.

Checking oil level

The oil tank has a dipstick to indicate the oil level. Check that oil level is between min. and max on the dipstick.

Oil level float switch

If the oil level drops below a certain minimum level an oil level float switch installed in the oil tank, operates alarm signals on the control panel in the operator's cab.

Verify that the switch is working properly by starting the crane, loosen the nut that holds the switch and lifting it up slowly.

Filling oil

Oil is filled at an ”aero-quip” type quick-release coupling, placed on the filter unit, see Fig. 2. When filling oil the hydraulic system must be at rest and the electric power switched off.

Fill oil to just under the MAX mark on the dipstick. Use oil according to the ”Lubricating chart”.

NOTE! Oil **must not** be filled directly into the tank.

Cleaning the oil tank

Before cleaning the oil tank, immobilise the crane jib in a suitable manner, push the stop button and switch off the power. Drain off the oil into clean empty barrels or drums.

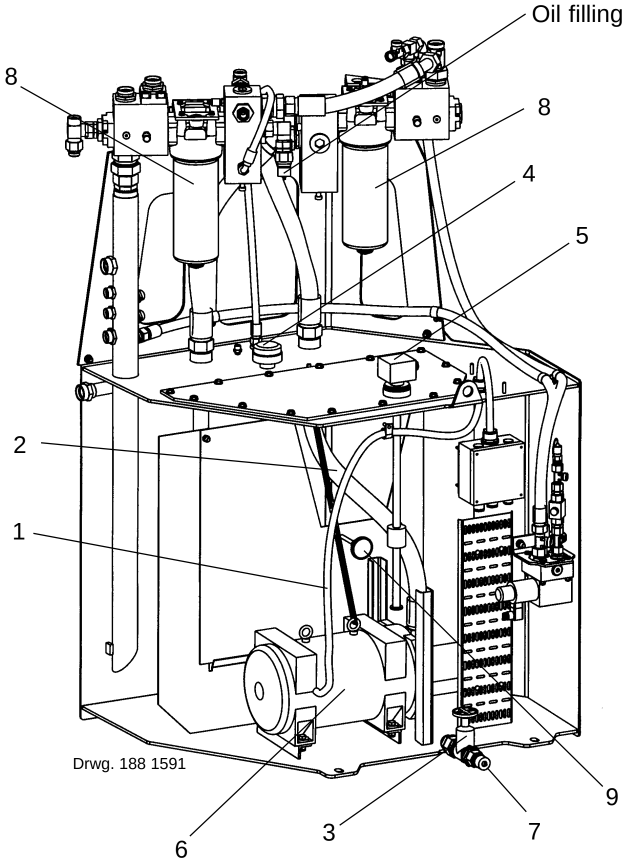

1.Radiator hose
2.Hydraulic hose
3.Vedge valve
4.Air filter with dipstick
5.Oil level and temperature guard
6.Feed pump
7.Quick coupling
8.Filter
9.Thermometer

Fig. 1. Tank with feed pump unit

Remove the tank cover. Scrape off sediment, if any, from the tank bottom and sides using a rubber scraper.

Flush the tank with white spirit.

Wipe the tank dry with clean rags. DO NOT USE COTTON WASTE, or other linting material. Apply new sealing compound and reinstall the tank cover.

If the drained oil is in good condition and can be used anew, it should be filtered before refilling.

Pump and hoses used for filling must be perfectly clean.

Fill oil to just under the MAX mark on the dipstick. Use oil according to the ”Lubricating chart”.

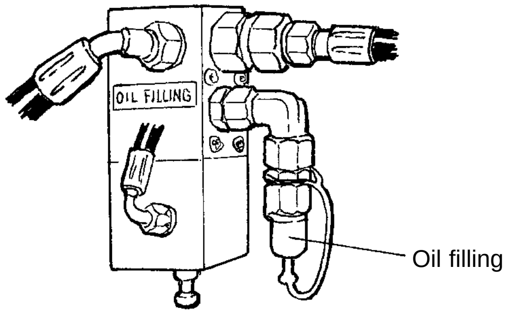

Fig. 2. Oil filter

Sampling of Oil to Verify the Cleanliness in Hydraulic System

General

This service information describes sampling oil from a hydraulic crane system.

This procedure is general and there can be some differences depending of crane type.

Type of oil to be used in the system can be found in Section 5.

When to Sample Oil

To verify the oil condition, an oil sample should be taken every six months and sent for analysis. This analysis is done by the oil companies and can be of different levels. When the result is received, a recommendation is normally given if the oil is approved or has to be changed.

For maximum practical contamination in the system, see Table 1.

Procedure for Oil Sampling

- Oil temperature in the system should be 40-50°C.
- Special oil sampling bottles should be used and can be purchased from oil companies.
- Connect a pressure gauge hose to gauge outlet 3.1 (hoisting circuit), (see Fig. 1).
- Start the crane and run the hosting winch in maximum speed hoisting.
- Bleed out 0.5 litre of oil in a bucket. This oil to be disposed.
- Fill the oil sample bottle.
- Send the oil sample to an oil company for analysis.
  The oil company will be able to recommend if the oil can be further used.
  Feedback on the oil analysis can also be obtained from MacGREGOR Cranes.

Procedure if Oil Sample is Showing Too High Contamination

If the oil analysis shows too high contamination, the oil and the filters should be changed. The crane should be driven for approx. 1 hr and a new oil sample should be taken and sent for analysis.

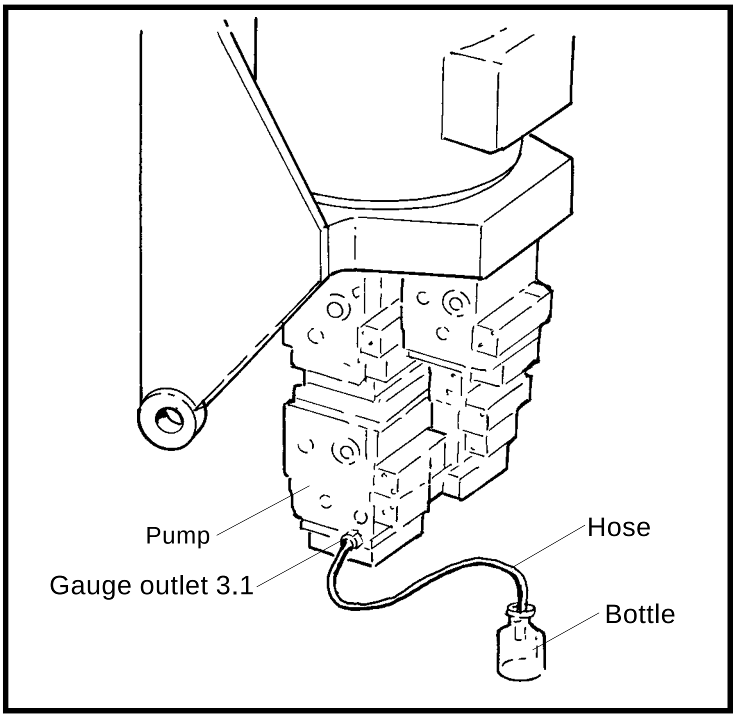

Fig. 1. Sampling Hydraulic Oil.

Not OK

OK

Cleanliness Level Correlation Table					
	Particles/millilitre				
ISO Code	≥5 Micro-meters	≥ 15 Micro-meters	ACFTD Gravimetric Level, mg/L	NAS 1638 (1964)	Disavowed "SAE" Level (1963)
26/23	640.000	80.000	1000		
25/23	320.000	80.000			
23/20	80.000	10.000	100		
21/18	20.000	2.500		12	
20/18	10.000	2.500			
20/17	10.000	1.300		11	
20/16	10.000	640	10		
19/16	5.000	640		10	
18/15	2.500	320		9	6
17/14	1.300	160		8	5
16/13	640	80	1	7	4
15/12	320	40		6	3
14/12	160	40			
14/11	160	20		5	2
13/10	80	10	0.1	4	1
12/9	40	5		3	0
11/8	20	2.5		2	
10/8	10	2.5			
10/7	10	1.3		1	
10/6	10	.64	0.01		

Table 1. Cleanliness Level Correlation Table.

Lubricants for Deck Machinery

A. OILS

The oil shall be refined oil containing additives against oxidation, rust and foaming, as well as wear inhibitor and/or EP additives.

■ **Enclosed spur and bevel gear units**
Oils with oil film reinforcing additives.
Viscosity 220 cSt/40°C approx. (ISO VG 220 HM).
Example:
SHELL OMALA 220. Solidification point -15° C
SHELL SPIRAX MB 90. Solidification point -21° C

Crane hydraulic system
For service in normal conditions:
Temperature range, -25°C - +45°C.
Viscosity 100 cSt/40° C approx. (ISO VG 100 HV).
Viscosity index (VI) min. 150.
Solidification point -35° C or better.
Example: SHELL TELLUS T100.

For service in arctic climate:
Viscosity APPROX. 46 cSt/40° C (ISO VG 46 HV).
Viscosity index (VI) approx. 150.
Solidification point -45° C or better.
Example: SHELL TELLUS T46.

Hydraulic motor, Axial thrust bearing
Synthetic gear oil for bearings.
Viscosity 680 cSt/40° C approx.
Viscosity index (VI) min. 165.
Solidification point -48° C or better.
Example: SHELL PAOLINA 680.

Load handling equipment, without oil heater

Viscosity 32 cSt/40°C approx. (ISO VG 32 HV).
Viscosity index (VI) min. 150.
Solidification point -45°C or better.
Example: SHELL Tellus T 32

Limit switch box, hoisting and luffing
Should not require further oil filling throughout its service life under normal driving conditions.
Filled with synthetic oil.

★ **Cable winch, turbo coupling**
Viscosity 22 cSt/40°C (ISO VG 22)
Example: SHELL MORLINA

◆ **Cable winch, spur gear**
Synthetic oil brand.
Example: MOBILE Glygoyle 30

B. GREASES

▲ **Plain bearings and wire rope sockets**
Grease containing molybdenum disulphide MoS_2.
Base oil viscosity 150 cSt/40°C.
Dropping point 180°C.
Example: MOBILGREASE Special 530303

☆ **Crane jib bearing**
Lithium/mineral oil-based grease with base oil viscosity 95 - 195 cSt/40°C.
NOTE: MUST NOT contain molybdenum disulphide.
Example:
SKF LGMT2 or SKF LGEP2. (-30°C to +110°C)
TEXACO MULTIFAC EP2. (-30°C to +100°C)

△ **Roller/ball bearings**
Grease with EP additives
Temperature range, -30 to +100°C.
Must withstand wet conditions.
Example: SHELL Alvania EP (LF) 2 (-20 to +130°C).

Arctic climate: SHELL Albida PPS2.

□ **Ropes**
Grease resistant to salt water and with antirust properties. (Very thin.)
Temperature range -30 to +100°C.
Viscosity 9.8 cSt/40°C.
Example: FUCHS Cedracon.

Arctic climate: FUCHS Ceplattyn.

◇ **Open spur gear units**
Grease with good adhesion properties.
Example: SHELL MALLEUS GL95 (-15 to +150°C).
SHELL MALLEUS GL65 (-30 to +150°C).

MacGREGOR

SERVICE

How to order spare parts 6.0

Hydraulic system 6.1

Mechanical equipment 6.2

Electrical equipment 6.3

Address:
MacGREGOR Cranes AB
S-891 85 ÖRNSKÖLDSVIK, Sweden
Telephone: 46 - 660 - 29 40 00
Telefax: 46 - 660 - 139 77 (Spare Part & Service Dept.)
Telefax: 46 - 660 - 29 42 81 (Technical Info. Dept.)
www.macgregor-group.com/cranes

E 2003-01-31

The Original Manufacturer of HÄGGLUNDS Cranes

How to Order Spare Parts

Important details when enquiring or ordering spare parts

To speed up the process we request you to provide us with below information:

- ☐ State name of company as well as your name
- ☐ Vessel's name and IMO number (and/or Lloyd's number)
- ☐ Crane type (see manual or name plate in operator's cabin)
- ☐ Crane place on the vessel. No. 1 counted from the fore
- ☐ Crane serial number (see manual or name plate in operator's cabin)
- ☐ Spare part figure or drawing number. To be found in section 9 in the manual
- ☐ Part description
- ☐ Item number from spare part figure or drawing respectively
- ☐ Part number, if available, from spare part figure or drawing respectively
- ☐ Quantity required
- ☐ Deadline - when or where in the world do you need the parts?
- ☐ Delivery details such as: consignee as well as phone and fax numbers
- ☐ Terms of delivery: by DHL, air freight direct or consignment, by truck or shipping freight

The more you can provide us with above details the faster and more reliable replies and deliveries will be made.

Address:
MacGREGOR Cranes AB
S-891 85 ÖRNSKÖLDSVIK, Sweden
Telephone: 46 - 660 - 29 40 00
Telefax: 46 - 660 - 139 77

MacGREGOR
S-891 85 ÖRNSKÖLDSVIK SWEDEN

Tel: 46 - 660 - 29 40 00
Direct: Fax: 46 - 660 - 139 77

Date: ....

Spare parts/Service specification

Please use this form whenever assistance is needed.

Service request ☐

Confirmation of phone call/conv. ☐

From: .. .. Fax no: ....

.... Tlx no: ....

....

Re: M/V .... IMO /Lloyd's no.: ....

Crane type:.. Serial no: ....

Crane place: .... Our ref.: ....

For following spares we would like to:

☐ have your quotation

☐ place order, deadline for arrival :..............................

Part no.	Qty	Description	Fig./Drwg.	Item
1.	.....	....	....	....
2.	.....	....	....	....
3.	.....	....	....	....
4.	.....	....	....	....
5.	.....	....	....	....

6. See enclosure ☐

Delivery/Agent's address: ....

....

....

....

Name/ Title/

Statement Regarding Delivery of Spare Parts to Swivels, Lifting Block, Lifting Beams etc.

Reference is made to ILO regulations "Safety and Health in Dock Work" paragraphs 190 and 191.

Paragraph 190

Every chain, ring, hook, shackle or swivel shall, after it has been lengthed, altered or repaired through welding shall be tested and examined in the manner specified in paragraphs 188 or 189.

Paragraph 191

(1) In case of important alterations or renewals of loading and unloading machinery and gear, and also after an accident, a test as required in paragraph 184 shall be carried out if considered necessary.

(2) A record of the test shall be entered in the appropriate register.

Paragraphs 184, 188 and 189 specify how the proof load and the inspection afterwards shall be performed.

Due to difficulties for the shipowner to perform adequate proof load test certified by a competent person, in general, MacGREGOR Cranes AB cannot supply separate spare parts to hook blocks, swivels, lifting beams etc. to the owners or managers. However, it is possible to deliver such parts to the MacGREGOR service organization, if they can guarantee that proof load test and appropriate inspection according to ILO will be performed under their responsibility.

MacGREGOR Cranes AB
Quality Assurance Department

Kjell-Erik Sellgren
Quality Manager

MacGREGOR - Cranes AB
ÖRNSKÖLDSVIK, SWEDEN

RETURN FORM

How to return parts from deck crane manufacturing, installation or guarantee exchange

Delivery address:

MacGREGOR Cranes AB
Service Department 220
S-891 85 ÖRNSKÖLDSVIK
SWEDEN

Transport means, if necessary:

Advised by us on request.
Tel. no 46-660-294049
Fax no 46-660-294288

Identification:
shipment,

Absolutly necessary is a paper inside the return

shortly describing
1) the part.
2) from where it has been taken and
3) why it is returned.

Claim:
have

Detailed claim can be sent separately and does not

to be enclosed.
The above refers only to the return shipment as such.

RETURN FORM

To be packed inside return box or parcel

Date	
Sent by	
Newbuilding no<br>Ship's name<br>Lloyd no	
Crane place on board<br>Crane serial no	
Returned part's drawing or article no, serial no<br>Part description	
Serial no of unit from which the part is taken<br>Unit description	
Reason for return *	

*) Please note: To be filled in with **short but specific** details. "Faulty" is not enough, a much better description is "leaking", "not adjustable", "cracked", "earthing fault", "short circuit" if the reason is obvious, or, a circumstantial evidence pointing to the returned part as the reason for malfunction, like "luffing movement too slow in one direction".

(222/IN: 662 5000-706)

Global Service

MacGREGOR

6.003.1 E rev.: k 2000-01-11

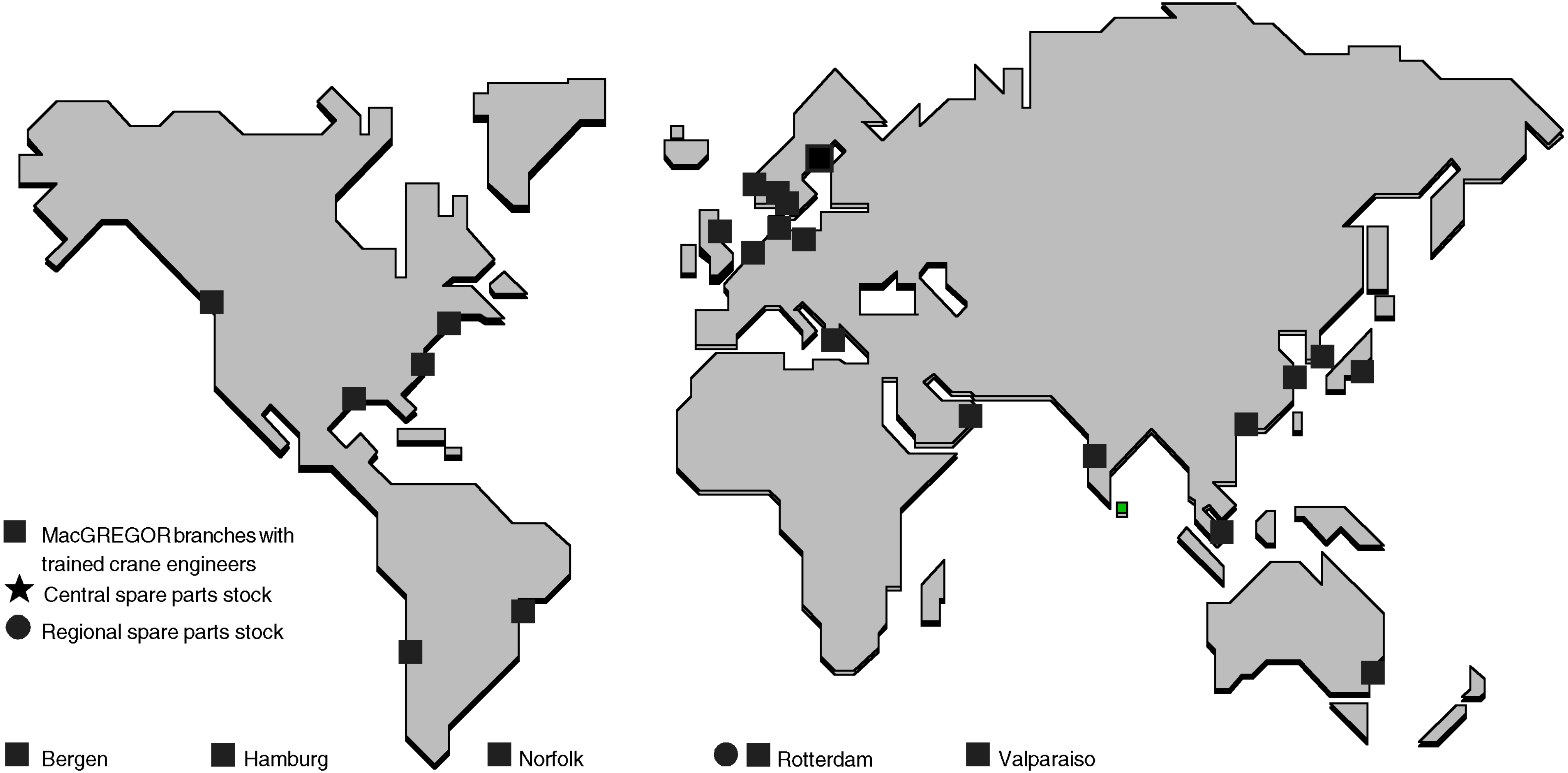

■ Bergen	■ Hamburg	■ Norfolk	● ■ Rotterdam	■ Valparaiso
■ Bombay	■ HongKong	■ Oslo	■ Shanghai	■ Vancouver
■ Copenhagen	■ Newcastle	■ Piraeus	● ■ Singapore	★ ■ ÖRNSKÖLDSVIK
■ Dubai	■ New Orleans	■ Pusan	■ Sydney	
■ Gothenburg	■ New York	■ Rio de Janeiro	■ Tokyo	

MacGREGOR Cranes AB
S-891 85 ÖRNSKÖLDSVIK
Sweden
Tel +46 660 29 40 00
Fax +46 660 12 455

The Original Manufacturer of HÄGGLUNDS Cranes

MacGREGOR Global Services

AUSTRALIA
Sydney, (S)
MacGREGOR (AUS) Ltd.
Unit 5 / 5 Samatha Place
Smeaton Grange
N.S.W. 2567
Phone: (+61) 2-4648 5977
Telefax: (+61) 2-4648 5966

BELGIUM
Antwerpen, (P)
MacGREGOR (NLD) B.V.
Luithagen-Haven 2E
B-2030 Antwerpen
Phone: (+32) 3-542 5112
Telefax: (+32) 3-542 4772

BRAZIL
Rio de Janeiro, (S)
Offshore Reparos Navais
Rua Delegado Waldir Guilherme 14
Ilha da Conceicao
CEP: 24.50-170
Niteroi - RIO DE JANEIRO
Phone: (+55) 21-2717 2673
Telefax: (+55) 21-2620 0740

CANADA
Vancouver, (S)
Nagesco Ltd.
961A West 1st Street
NORTH VANCOUVER, B.C.,
V7P 1A5
Phone: (+1) 604-985 61 73
Telefax: (+1) 604-980 44 76

CHILE
Santiago, (S)
EXIMTEC Ltda.
Neveria 4444 - Piso 9
Las Condes
SANTIAGO
Phone: (+56) 2-207 65 90
Telefax (+56) 2-207 65 91

CHINA
Hong Kong, (S) (P)
MacGREGOR (HKG) Ltd.
Unit 3511-12, 35/F, Tower 1,
Millennium City 1,
388 Kwun Tong Road
KOWLOON
Phone: (+852) 2394 1008
Telefax: (+852) 2787 7652
Telefax: (+852) 2787 9878 (cranes)

Shanghai, (S) (P)
MacGREGOR (SHANGHAI) Trading Co. Ltd.
Room 1701-1704, Pidemco Tower
318 Fuzhou Road
SHANGHAI 200001
Phone: (+86) 21-6391 2798
Telefax (+86) 21-6391 2276

CROATIA
Rijeka, (P)
MacGREGOR (HRV) d.o.o.
Vrh Martinscice 93B
HR-51221 RIJEKA-KOSTRENA
Phone: (+385) 51-289 717 / 718
Telefax: (+385) 51-287 154

CYPRUS
Limassol, (P)
MacGREGOR (CYPRUS) Ltd.
Titos Building, Office 201
58 Gladstone Street
CY 3041 LIMASSOL
Phone: (+357) 5 763 670
Telefax (+357) 5 763 671

DENMARK
Copenhagen, (S) (P)
MacGREGOR (DNK) A/S
Smedeholm 11,
2730 HERLEV
Phone: (+45) 44-53 84 84
Telefax: (+45) 44-53 84 10

FINLAND
Turku, (P)
MacGREGOR (FIN) OY
Hallimestarinkatu 6
FIN-20780 KAARINA
Phone: (+358) 2-412 11
Telefax: (+358) 2-4121 517

FRANCE
Marseille, (P)
MacGREGOR (FRA) S.A.
Porte 4, Cap Janet
13314 MARSEILLE Cedex 15
Phone: (+33) 491-09 52 52
Telefax: (+33) 491-09 52 40

GERMANY
Hamburg, (S) (P)
MacGREGOR (DEU) GmbH
Normannenweg 24
D-20537 HAMBURG
Phone: (+49) 40-25 44 40
Telefax: (+49) 40-25 44 44 44

GREECE
Piraeus, (S) (P)
MacGREGOR (GRC) Ltd.
4-6 Efplias Street
GR-185 37 PIRAEUS
Phone: (+30) 210-42 83 838
Telefax: (+30) 210-42 83 839

INDIA
Bombay, (S)
Renascent Enterprises
Flat 8, Building D/6-9
Radha Nagar, Barave Road
KALYAN (WEST), Pin - 421 301
Phone: (+91) 251 232 30 65
Telefax: (+91) 251 232 06 43

Codes for available services from branches

(S)	=	Branch with specially trained crane service engineers
(CS)	=	Central spare parts stock
(RS)	=	Regional parts stock, servicing many countries in the same region
(P)	=	Branch with spare parts sales resources

ITALY

Genoa, (P)
MacGREGOR (ITA) S.p.A.
Via Al Molo Giano
16128 GENOA/PORTO
Phone: (+39) 010-2770 412
Telefax: (+39) 010-24 61 194

JAPAN

Tokyo, (S) (P)
MacGREGOR-Kayaba Ltd. - Head Office
9/F, Suzue Baydium Bldg.
1-15-1, Kaigan
Minato-Ku
TOKYO 105 - 0022
Phone: (+81) 3-5403 1966
Telefax: (+81) 3-5403 1953

KOREA

Pusan, (S) (P)
MacGREGOR (KOR) Ltd.
Room No 1101, Dong Ju Building
13-5 ka, Jungang-dong, Jung-ku
PUSAN
Phone: (+82) 51-441 0805
Telefax: (+82) 51-442 5777

NETHERLANDS

Rotterdam, (S) (P) (RS)
MacGREGOR (NLD) B.V.
Albert Plesmanweg 95-97
3088 GC ROTTERDAM
Phone: (+31) 10-2832 121
Telefax: (+31) 10-4293 219

NORWAY

Oslo, (P)
MacGREGOR (NOR) A/S
Malerhaugveien 25
N-0602 OSLO
Phone: (+47) 23-10 34 00
Telefax: (+47) 23-10 34 01

Bergen, (S)
MacGREGOR (NOR) A/S
Bredalsmarken. 15-17
N-5006 BERGEN
Phone: (+47) 23-30 25 50
Telefax: (+47) 23-30 25 51

POLAND

Gdynia, (P)
MacGREGOR (POL) Sp. z o.o.
Ul. Energetykow 5
PL-81-184 GDYNIA
Phone: (+48) 58-7855 110
Telefax: (+48) 58-7855 111

SINGAPORE

Singapore, (S) (P) (RS)
MacGREGOR (SGP) Pte. Ltd.
No. 12 Benoi Crescent
629975 SINGAPORE
Phone: (+65) 6265 2322
Telefax: (+65) 6264 1261

SPAIN

Bilbao, (P)
MacGREGOR (ESP) S.A.
Edificio Inbisa
Amaya, 2 - 1°
ES-48940 LEIOA - VIZCAYA
Phone: (+34) 94-480 7339
Telefax: (+34) 94-431 6945

Alicante, (S)
Stanley van der Pers
c/o Vanpershydro S.L.
Apartado de correos No. 7
ES-03170 ROJALES - ALICANTE
Phone/telefax: (+34) 9667 13665
Mobile: (+34) 620 953 722

SWEDEN

Örnsköldsvik, (S) (P) (CS)
MacGREGOR Cranes AB
Björnavägen 14
SE-891 85 ÖRNSKÖLDSVIK
Phone: (+46) 660-29 40 00
Telefax: (+46) 660-139 77
www.macgregor-group.com/cranes

Gothenburg, (S) (P)
MacGREGOR (SWE) AB
Atlashuset, Banehagsliden 2
SE-414 51 GOTHENBURG
Phone: (+46) 31-85 07 00
Telefax: (+46) 31-42 49 46

U. A. E.

Dubai, (S) (P)
MacGREGOR (ARE) LLC.
P.O. Box 30029 Dubai
Al Jadaf Shipyard area / GMMOS Yard
DUBAI
Phone: (+971) 4-3241 335
Telefax: (+971) 4-3241 271
24h phone: (+971) 50-65 10 371

UNITED KINGDOM

Newcastle, (P)
MacGREGOR (GBR) Ltd.
86/90 Front Street
Whitley Bay
NE25 8DN TYNE & WEAR
Phone: (+44) 191-253 4611
Telefax: (+44) 191-251 5585

USA

New York, (S) (P)
MacGREGOR (USA) Inc.
20 Chapin Road, Unit 1012
New Jersey 07058, PINE BROOK
Phone: (+1) 973-244-4100
Telefax: (+1) 973-244-4101

Norfolk, (S)
MacGREGOR (USA) Inc.
508 John Etheridge Road
CHESAPEAKE, VA 23322
Phone: (+1) 757-421-9406
Telefax: (+1) 757-421-2026

New Orleans, (S)
MacGREGOR (USA) Inc.
5600 Jefferson Highway
Suite W1-186
HARAHAN, LA 70123
Phone: (+1) 504-733-3321
Telefax: (+1) 504-733-3360

Codes for available services from branches

(S) = Branch with specially trained crane service engineers
(CS) = Central spare parts stock
(RS) = Regional parts stock, servicing many countries in the same region
(P) = Branch with spare parts sales resources

Introduction

Before proceeding to trouble-shooting, the crane operator and the service staff should familiarize themselves with the construction and functions of the crane. It is a bit late in the day to learn how to operate the crane when it has already broken down.

Begin by learning the hydraulic and electric symbols used in the circuit diagrams to facilitate tracing the probable fault in the circuit diagrams, Fig. 1 and 2, checking the findings afterwards in the crane proper.

Trouble-shooting may thus be divided into a theoretical and a practical part, each subdivided into hydraulic, electric, and mechanical trouble-shooting. The hydraulic and electric operations presuppose knowledge of the locations of various points of measurement and checking.

Take care always to have correct circuit diagrams available on board. If a diagram has gone astray, a new one can be had from MacGREGOR-HÄGGLUNDS. Proper measuring instruments must always be available, e.g. pressure gauges and voltmeters.

The trouble-shooting method described here is only intended as a general guide. Methods and practices may vary, but the purpose is always the same: to find the fault quickly and methodically in order to put it right.

When a crane function ceases to operate properly, it generally does so in actual operation. This means that the crane operator will often be able to give valuable suggestions, e.g.:

- **Did the crane function fail without warning?**
- **Was the operating speed first reduced, or did the movement run irregularly?**
- **What manoeuvre immediately preceded the failure?**

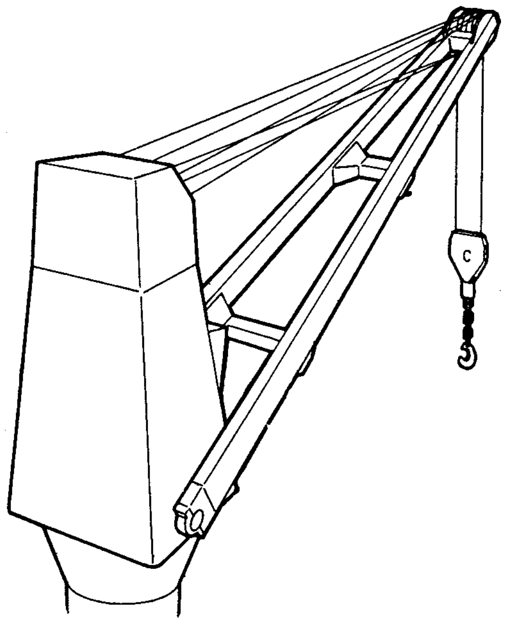

Fig. 1.

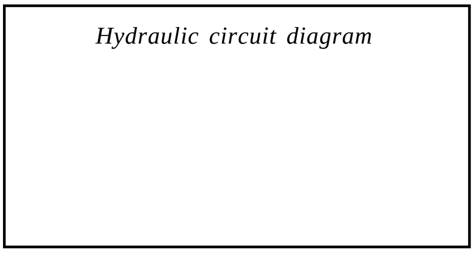

Fig. 2.

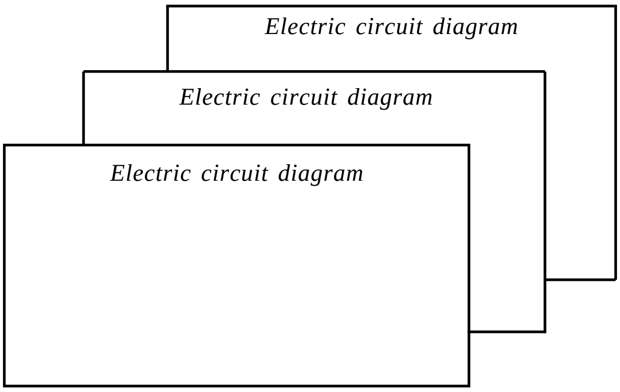

Fig. 3.

As a first check always try to operate the crane and at the same time check the status of the trouble-shooting panel in the B-box and BB-box, (see instruction under section 6.3).

Also measure the feed pressure at gauge outlets 1 and 16. Check the control pressure at gauge outlet 2. Pressure setting levels can be found in the pressure setting list on the hydraulic circuit diagram.

When consulting MacGREGOR-HÄGGLUNDS for service or advice, always give the following information at once:

1. Is the fault electrical, electronic, hydraulic, or mechanical in nature?
2. In which function does the fault appear:
   - Hoisting circuit
   - Luffing circuit
   - Slewing circuit
3. Is the trouble limited to:
   - Raising or lowering of the load
   - Raising or lowering of the jib
   - Slewing right or left
4. Special observations:
   - Is the hoisting, luffing, or slewing capacity restricted?
   - Do disturbances occur only at certain speeds?
   - In the event of abnormal noise:
   - To which of the main functions does the noise pertain?
   - Is the frequency of the noise high or low?
   - Try to describe the noise (hissing, blowing, humming, rasping etc.)
5. What is the status of the electrical trouble-shooting panel in B-box?
6. Have pressures been measured?
   - Feed circuit pressure
   - Control circuit pressure
   - Hoisting circuit high pressure
   - Luffing circuit high pressure
   - Slewing circuit high pressure

Analysis of information collected

By methodical trouble-shooting, it is often possible to find, at an early stage, the defect producing the faulty operation, thereby avoiding unnecessary waste of time.

> All important pressure adjusting devices are sealed. Breaking a seal voids the guarantee unless done with the explicit consent of MacGREGOR-HÄGGLUNDS.

Measures Before Starting, During and After Completed Service/Maintenance Work

WARNING !
When it is necessary to search for/or correct faults in the crane's electrical, hydraulic or mechanical systems with the crane powered up, only authorized personnel may carry out such work.

The following measures must be taken before any service or maintenance work is started.

1. The jib must be secured preferably in the parking support, (see separate instruction).

2. All lifting equipment must be parked/secured.

3. All power supplies to the crane must be cut.

   A. Cut the main power supply to the crane and all separate supplies such as heating, lighting etc., with the power switch(es) in the ship’s engine room.

   B. Warning signs must be hung on the switches involved.

4. Make sure that the crane is completely without power. If necessary, lock switches, take out fuses etc. so nobody by mistake can start the crane.

5. When special work has to be done see separate instruction or contact our service department.

6. If some special arrangements are done for testing during the service, these arrangements have to be removed after completed service.

7. Function test safety items i.e. limits, brakes etc. after completed work.

8. Protections i.e. covers, rails etc. to be reinstalled after completed work.

Trouble-Shooting Chart, Overheating

Fault	Probable cause	Remedy
**A.** Overheating in hydraulic system.	Oil cooler fan motor not working.	Check connections, contactor, effect repairs as required.
	Oil cooler flanges loaded with dirt.	Dismantle and clean cooler.
	Pressure control valves incorrectly set.	Correct settings.
	Internal leakage.	Trace leaks and replace defective seals, packings, and other parts.
**B.** Overheating caused by pump, valving, or hydraulic motor.	Internal leakage because of wear.	Contact MacGREGOR Cranes.

N.B.
When the hydraulic system is overheated thermostat BT2 (+85°C) opens. To cool the system set the switch "Winter-Summer-Test" in "Test" position and press the "Start" button on driver's panel. Only cool fan and feed pump start and oil circulates in the system.

Trouble Shooting, Pumps

Pumps, general

Fault	Probable cause	Remedy
A. Pump not supplying any fluid.	Servo error.	Contact MacGREGOR Cranes
	Worn or cracked parts in the pump.	Measure leakage, contact MacGREGOR Cranes
B. Excessive wear of the pump.	Hydraulic fluid contains abrasive particles.	Check filters. Change cartridge, if necessary. Analyze sample of the fluid.
	Low feed pressure.	Check pressure switch 1381 and 2481.
C. Fracture of parts in the pump.	Pressure shocks.	Check valve functions and valve settings.
	Low feed pressure.	Check pressure switch 1381 and 2481.

More detailed information regarding the pumps will be found in the instruction manual.

Slewing Gearset
(Pinion module 14 and 16)

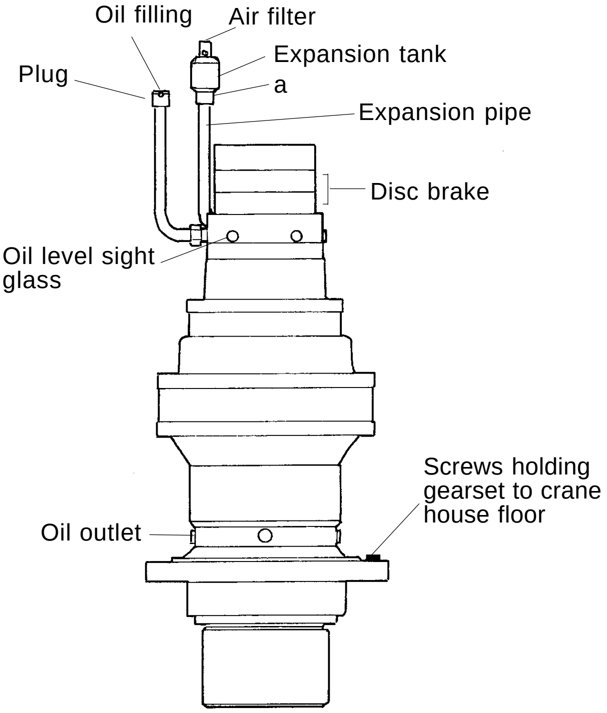

Fig. 1. Slewing gearset.

Assembly of slewing gearset

- Install slewing gearset in crane without using force.
- Use hand pump to release disc brake hydraulically. Input drive sleeve must turn easily.
- Check slewing gear backlash (pinion-to-rim gear). See description under Checking and adjusting slewing rim backlash.
- The gear backlash may be adjusted by rotating the gearset one or more screw hole divisions.
- Tighten the screws to torque recommended, secure with Loctite 242 (or a similar compound). Applying a suitable sealing compound, such as Loctite 640, on the mating surfaces of the flanged connection, is recommended.
- Remove threaded plug from top of expansion pipe (for transport sealing only).
- After installation of gear unit fit supplied air filter with oil expansion tank, at marked position a (optional to fit with a tube turn). See Fig. 1.
- Fill gear oil as required (see lubricating instructions for appropriate type; the approximate quantity needed is given on the nameplate).
- Fill brake disc chamber with hydraulic oil; e.g. 0,1 litre ATF-oil, type A.
  Brake surface sealed with Loctite type 573.
- Before assembling the drive motor, grease shaft and gear teeth with a suitable grease for protection against corrosion; we suggest OPTIMOLY PASTE White T.
- Check function of slewing gearset with the drive motor running, paying particular attention to the function of the brake and the correct control of the motor.

Maintenance

Lubrication

The slewing gearset is splash lubricated. The output shaft roller bearing is ”for-life” lubricated with ball bearing grease.

Oil level check

Inspect oil level when the gear is at rest with the brake engaged.

Oil changes

Whenever practicable, let the oil drain off while still hot; remove also the filler plug(s).

For oil change open both pipes. Remove air filter with oil expansion tank at marked pos. a, see Fig 1.

At low ambient temperatures, flush out the gearcase with a quantity of fresh oil to remove abraded matter and contamination products.

Gearcase and oil inspection

Sampling oil

Withdraw a sample of gearcase oil for inspection from the filling drained off at intervals as described in the Maintenance Chart. To obtain a representative sample, drain the gearcase during operation and with the oil at operating temperature.

Oil discoloration

A darkish or black colour of the used oil suggests that temperatures in excess of 100° C did occur in operation, causing accelerated aging of the oil and impaired lubricating properties. In this case the oil should be changed at shorter intervals.

If the oil sample displays a cloudy aspect, it may be that water has managed to seep into the oil. The oil must then be changed **at once,** and the gearcase flushed out very thoroughly several times with fresh oil.

The gearcase should preferably be dismantled for inspection beginning at the input side, and the components examined for signs of corrosion.

Foreign solid matter

- As a first simple test, drop some of the used oil onto the filter fleece specified. Coarse foreign matter, if present, will then show up on inspection, preferably through a magnifying glass.
- A better test may be made by diluting an oil sample with n-heptane, passing it through a diaphragm filter, washing and drying it, and weighing it to determine the mass difference.

Overall assessment

The maximum permissible amount of foreign matter carried by the oil is some 0.15 per cent of the total mass of the oil in the gearcase. These impurities must be, exclusively, very fine abraded matter (max 25 µ). If the contamination keeps within these limits, continued operation of the gearset is permitted after the oil has been changed.

If coarser abraded matter (above 25 µ) or flake off material from the gear teeth is found, the gearset must be dismantled, irrespective of the actual amount of foreign matter present, beginning at the input end of the gearset.

Hydraulic-Release Multiple-Disc Brake

Technical description

The brake comprises the following major components:
- Brake piston
- Brake cylinder
- Disc carrier
- Internal discs
- External discs
- Sealings
- Thrust spring

The brake housing is mounted on top of the gearcase, with long assembly screws through the motor adapter housing and both parts of the brake housing.

The sleeve disc carrier, with the internal discs, is fixed on the driven shaft with a shaft key or by splines.

The disc assemblies are pressed together by a set of thrust springs and the brake is disengaged by hydraulic power.

Maintenance

The brake is self-adjusting.

Trouble-shooting

a. Braking torque insufficient

Test of brake torque may be performed according to following:
- Drive hydraulic motors against closed brakes. Brakes shall be able to withstand motor torque without slipping.

Note! Test procedure shall be performed only for a short period, a few seconds.

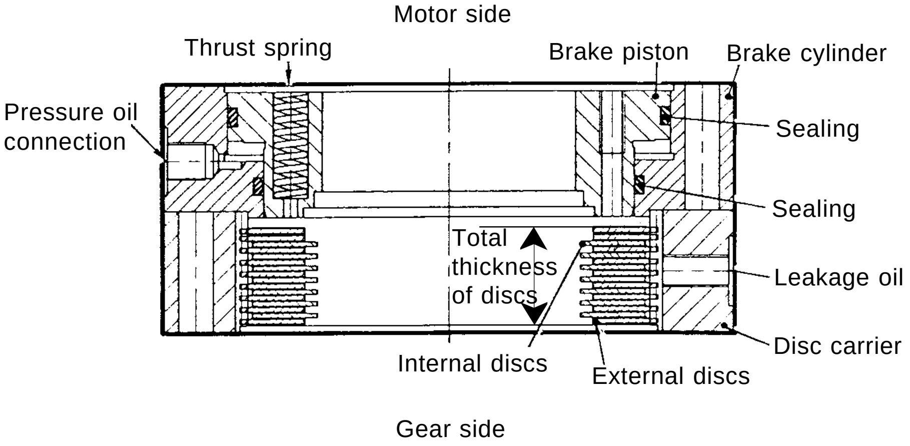

Fig. 2. Disc brake.

Attention: Please select the requisite number of thrust springs from parts list "gear".

- Inspect brake discs and replace if excessively worn.
- If discs are coloured dark yellow to blue, the discs have been overheated. Replace discs and springs.
- Inspect sealings for wear. If sealings are defect replace brake cylinder and piston with sealings to new ones.

b. Brake heats
Check the hydraulic pressure to make sure that it is sufficient in order to disengage the brake completely.

Checking and setting slewing rim backlash

An oversize backlash between the pinion and the slewing rim will cause irregularities in the slewing movement of the crane. Read this instruction carefully prior to checking or resetting!

Checking backlash

The backlash should be checked by a dial gauge or a feeler. When slewing the crane, the backlash may vary from one position to another due to some slight excentricity of the slewing rim. It is therefore recommended to check the backlash at four positions of the slewing rim.

Proceed as follows:
- Check the backlash at one spot.
- Slew the crane 90 degrees and check.
- Proceed to check the backlash at four 90-degree position in the rim.
- Put the dial gauge on the tooth of the pinion. By manual turning at the input, the pinion can be moved in both directions up to contact with the teeth. Now, the tooth flank clearance can be exactly measured on the dial gauge.
- Check the backlash at one spot
- Slew the crane 90°. Check again
- Proceed to check the backlash at four 90-degree positions in the rim
- Compare the four backlash values.

  Backlash values:
  Module 14 and 16 0.45 ±0.20mm

The backlash shall be set at the position of the slewing rim with the lowest backlash value.

Setting backlash

The backlash may be set by turning the entire gearset one or more screw hole divisions.

Proceed as follows:
- Slew the crane into the position with the lowest backlash value.
- Remove all screws holding the gearset to crane house floor.
- Release the disc brake hydraulically by means of a manual pump.
- Turn the entire gearset one or more screw hole

to achieve the desired setting.
To turn the gearset, remove the hydraulic motor and turn the spline sleeve on top of the gear.

Note!
The turning of the entire gearset may be restricted by the hoses connected or by space limitations. To overcome this problem, release the upper part of the gearset by just removing the holding screws, see Fig. 3 ”Slewing gearset”.

Make sure that the upper part can be turned freely. Turn the entire gearset (except the upper part), until the desired setting is achieved. Watch the hoses!

- Replace and tighten the screws holding the gearset to the crane house floor using ”Loctite 242” or a similar compound.

If the upper part has been removed

If the upper part has been removed, clean its contact surfaces and apply Loctite 640 Sealant or a similar compound. Make sure not to strain the hoses . Apply Loctite 242 on the screws holding the upper part, reinstall and tighten.

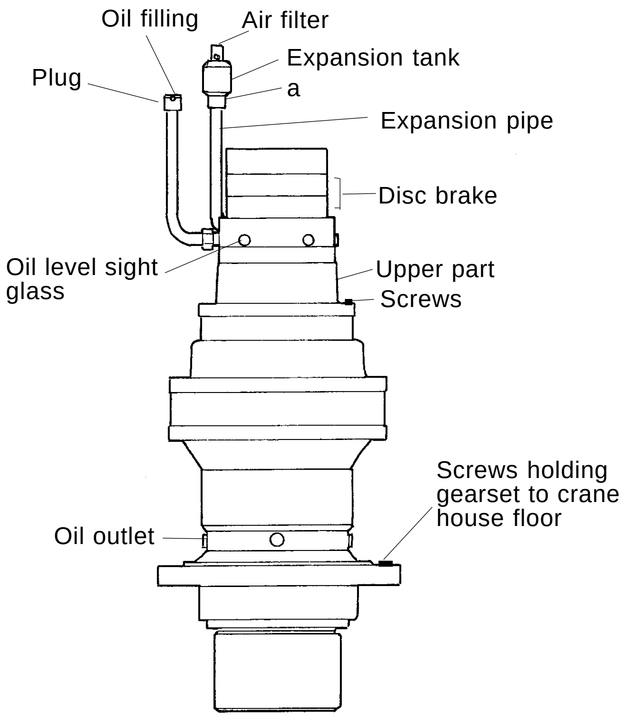

Fig. 3. Slewing gearset.

Hydraulic Motor
287 6935 Displacement: 58 cc
388 3127 Displacement: 59.8 cc

Before the first operation the hydraulic motor housing should be pre-filled with hydraulic oil.

The type of hydraulic oil must agree with the Lubricating Chart.

Pre-filling volume: 0.5 litre.

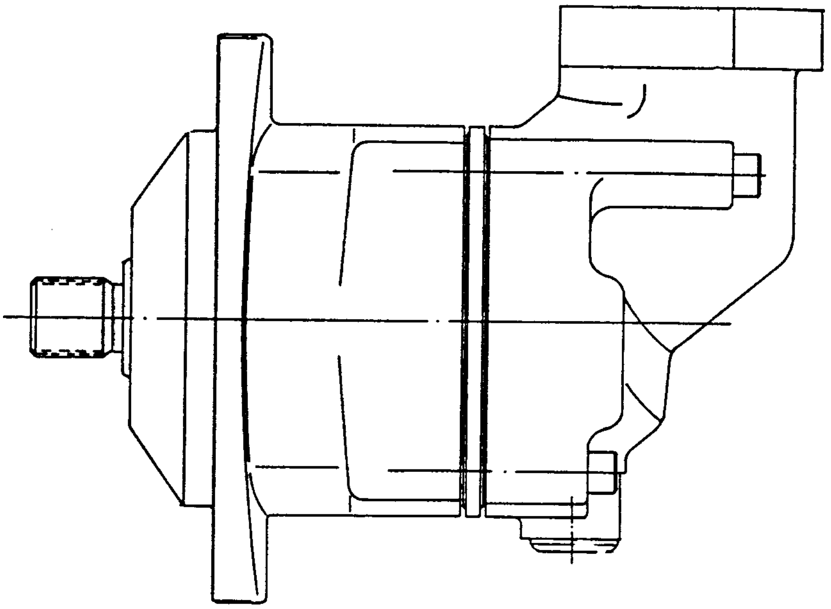

COMPACT Winch

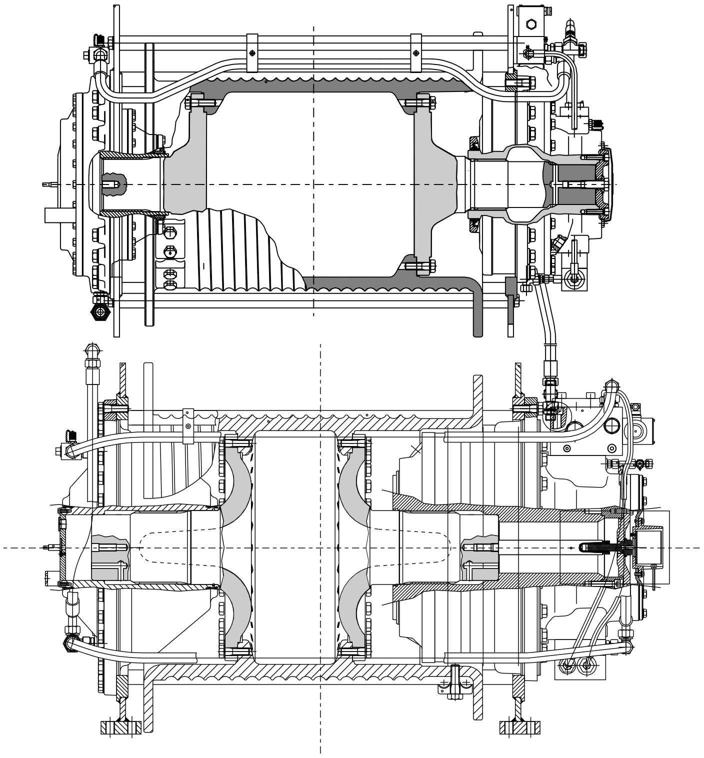

Contents

Preface 2
Safety precautions 2
The COMPACT winch 3
Mounting the motor onto the winch 4
Mounting the brake onto the winch 5
Mounting valve blocks, winch drums and brackets 6

1. Preface

Our high quality winch systems, are based upon our unique hydraulic piston motors, developed through a wealth of experience accumulated over 30 years in marine and industrial areas. Today this ongoing development work has resulted in the powerful COMPACT winch. New, as well as established technical solutions, contribute to the creation of this product. The most desirable features and operating reliability have been designed in this winch.

This instruction provides necessary information for installation of the winch. In order to find particular information, just search for the wanted section as listed in the table of contents. However, changes in the equipment may occur. We therefore reserve the right to introduce amendments in the manual as we deem necessary without notice or obligations.

2. Safety precautions

It is of high importence that the safety precautions are always followed, if you are unsure about something, please don´t hesitate to contact your nearest MacGREGOR-office for advice.

2.1 Warning signs

In this instruction you will find the following signs which indicate a potential hazard, which can or will cause personal enjury or substantial property damage. Depending on the probability of the hazard, and how serious the injury or property damage could be, there are three levels of classification.

DANGER!
Is used to indicate the presence of a hazard which will cause severe personal injury, death, or substantial property damage if the warning is ignored.

WARNING!
Is used to indicate the presence of a hazard which can cause severe personal injury, death, or substantial property damage if the warning is ignored.

CAUTION!
Is used to indicate the presence of a hazard which will or can cause minor personal injury or property damage if the warning is ignored.

2.2 Application area

All new and rebuild applications, should always be approved and supervised by MacGREGOR personel.

2.3 Mounting

Carefully follow the instructions and be aware of the high weights and forces during lifting.

2.4 Before starting up

Before starting up new, rebuild or just worked on applications, all accessories and safety arrangements functions, should be controlled/tested.

2.5 Periodic maintenance

Notice the intervals in Maintenance chart, section 5, and keep a record.

2.6 Dismounting

Carefully follow the instructions and be aware of the high weights and forces during lifting.

3. The COMPACT winch

This COMPACT winch instruction is including the COMPACT motor and the MDA brake. The COMPACT motor has also a seperate instruction, section 6.1. The MDA brake has also a seperate instruction, section 6.2.

The COMPACT winches features both hoisting and luffing winches. The drums are guided at the bearings inside motors and brakes.The pulling force is transmitted by spline shafts in the centre of the drums, to the motors, brakes and brackets.

The hoisting winch (Fig. 2) stand on feet bolted to the floor, and the luffing winch (Fig. 1) hang in brackets on the wall.

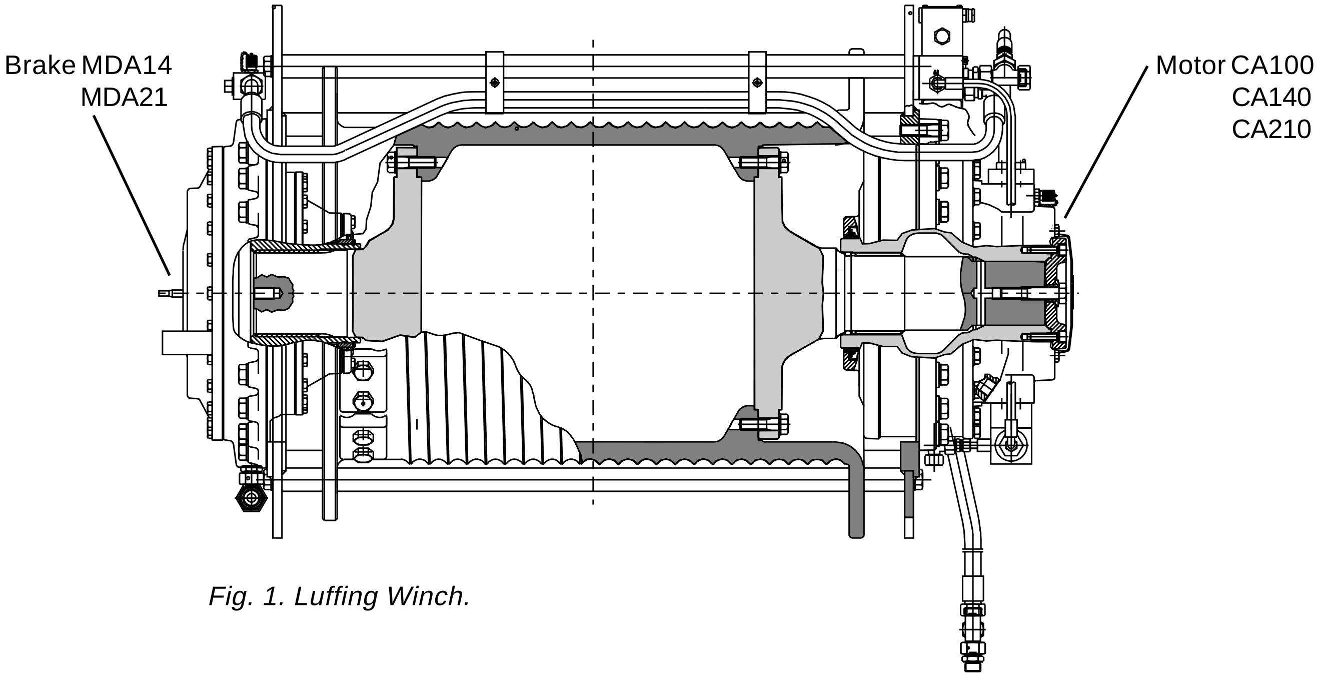

Fig. 1. Luffing Winch.

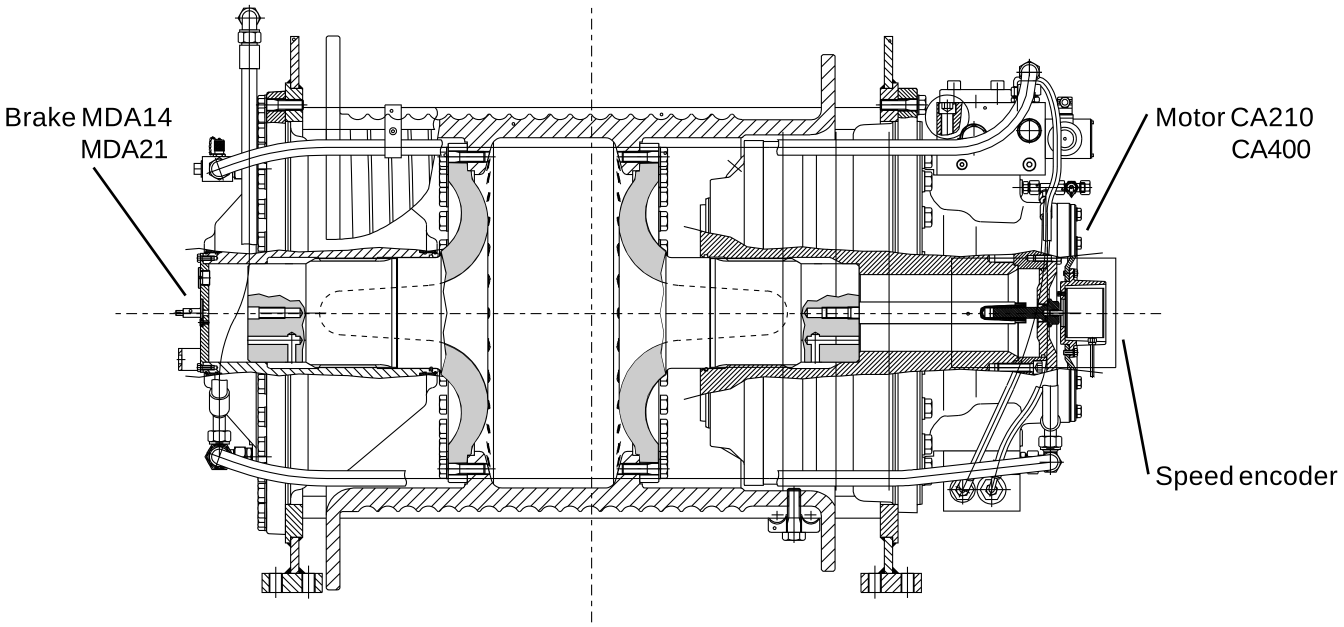

Fig. 2. Hoisting Winch.

4. Mounting the Motor onto the Winch

4.1 Luffing winches

- Remove the end cover together with screws and washers.
- Align the motor with the driven shaft (Fig. 3).
- Push on the motor by hand.
- The motors, CA 100/140/210 are mounted with M6S 20x70 (strength class 10.9). Tightening torque 540 Nm.
- Fill up the inside of the motor with hydraulic oil. CA 100/140/210 is filled with 1.0 litre.
- Refit the end cover together with screws and washers.
- Mount the screw on the shaft, M6S 20x100 (strength class 10.9). Tightening torque 540 Nm.

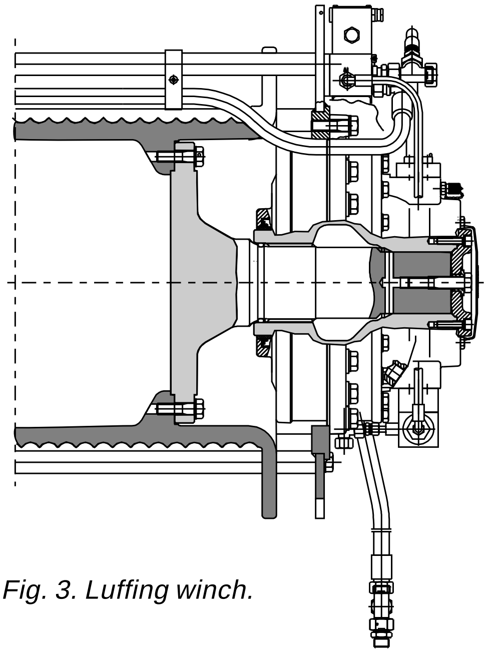

Fig. 3. Luffing winch.

4.2 Hoisting winches

- Remove the speed encoder kit (Fig. 5) together with screws and washers.
- Align the motor with the driven shaft (Fig. 4).
- Push on the motor by hand.
- The motors, CA 210/400 is mounted with M6S 20x70 (strength class 10.9). Tightening torque 540 Nm.
- Mount the distance rod on to the driven shaft. Tightening torque 385 Nm.
- Fill up the inside of the motor with hydraulic oil. CA 210 is filled with 1.0 litre. CA 400 is filled with 2.5 litre.
- Refit the speed encoder kit together with screws and washers. Tightening torque for special centre bolt, 385 Nm.

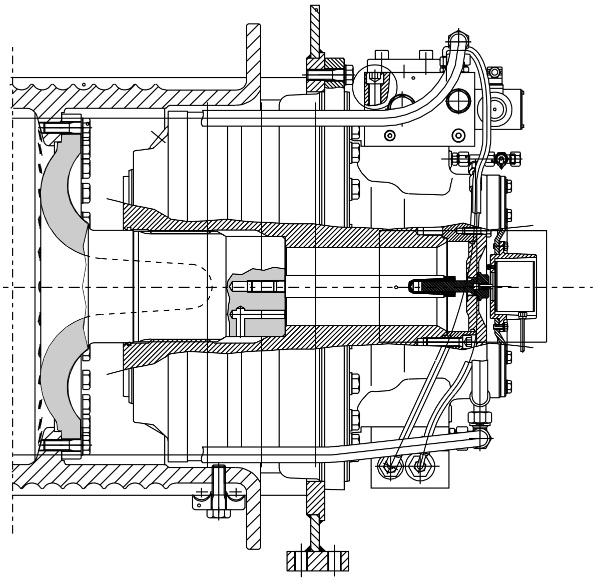

Fig. 4. Hoisting winch.

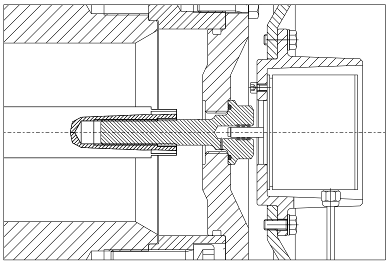

Fig. 5. Speed encoder kit, hoisting winch.

5. Mounting the Brake onto the Winch

5.1 General

The brake is of the fail safe multi disc type. During normal operation hydraulic pressure keeps the discs within the brake separated, allowing shaft rotation. If hydraulic pressure is lost, springs force the discs together, stopping rotation of the winch drum.

5.2 MDA 14 & 21

- Grease the spline on the driven shaft with Texaco Multifac EP2 or equivalent.
- Align the brake with the driven shaft (Fig. 6).
- Push on the brake by hand.
- Open the brake with hydraulic pressure, 20 bar, turn and align with the M20-holes.
- MDA 14 & 21 are mounted with M20x70 (strength class 10.9). Tightening torque 540 Nm.

Note:

Be careful not to damage the pin in the centre of the brake!

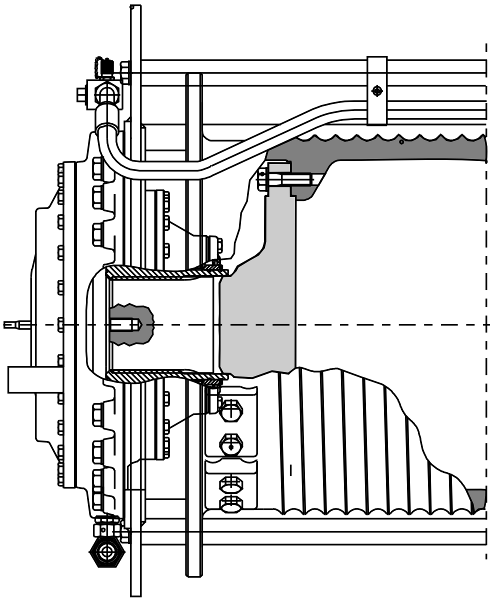

Fig. 6. Brake MDA 14 & 21.

5.3 MDA 42

- Align the brake with the driven shaft (Fig. 7).
- Push on the brake by hand.
- Open the brake with hydraulic pressure, 20 bar, turn and align with the M20-holes.
- MDA 42 are mounted with M20x70 (strength class 10.9). Tightening torque 540 Nm.
- Fill up the brake with hydraulic oil, 1 litre.

Note:

Be careful not to damage the pin in the centre of the brake!

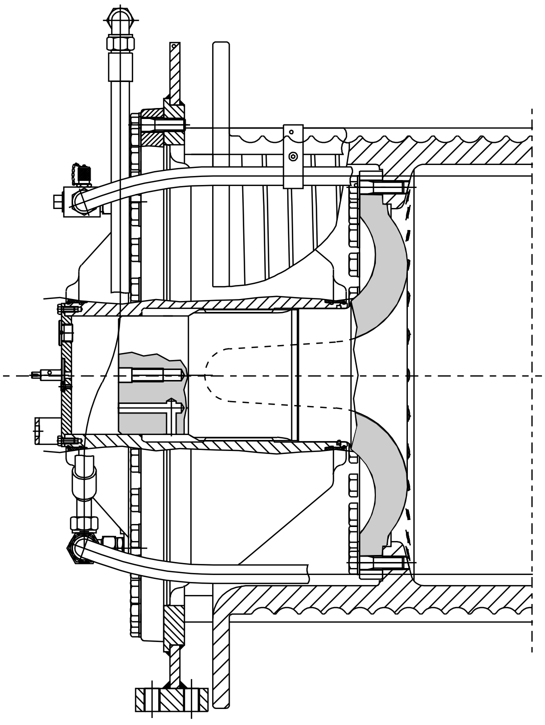

Fig. 7. Brake MDA 42.

6. Mounting Valve blocks, Winch drums and Brackets

6.1 General

- The winch drums are mounted with centre shafts inside the drums. Bolts M20x70 (strength class 10.9). Tightening torque 540 Nm.
- The brackets are connected with Æ 40 steel bars and bolted with M20x70 (strength class 10.9). Tightening torque 540 Nm.

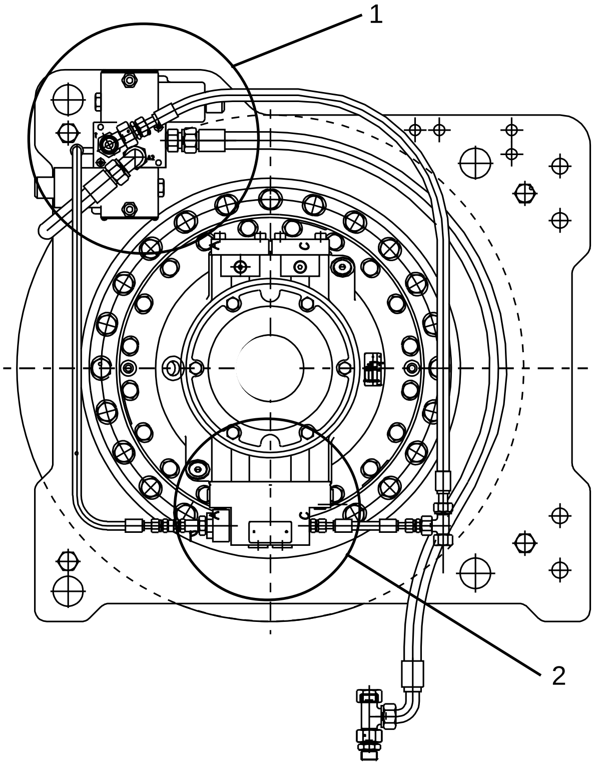

Fig. 8. Luffing winch, motor side.

6.2 Luffing winch

- The valve unit (Fig. 8, Item 1) is mounted on to the bracket with a distance plate and M6S 8x100 (strength class 8.8). Tightening torque 24 Nm.
- The Unloading valve (Fig. 8, Item 2) is mounted on to the motor with UC6S 1/2" UNCx51 (strength class 12.9). Tightening torque 163 Nm.

Note:

Be careful not to damage the O-rings between motor and valve.

6.3 Hoisting winch

- The 2-speed valve (Fig. 9, Item 3) is mounted on to the motor with MC6S 20x140 (strength class 12.9). Tightening torque 385 Nm.
- The Flush-unloading valve (Fig. 9, Item 4) is mounted on to the motor with UC6S 5/8" UNCx51 (strength class 12.9). Tightening torque 323 Nm.

Note:

Be careful not to damage the O-rings between motor and valve.

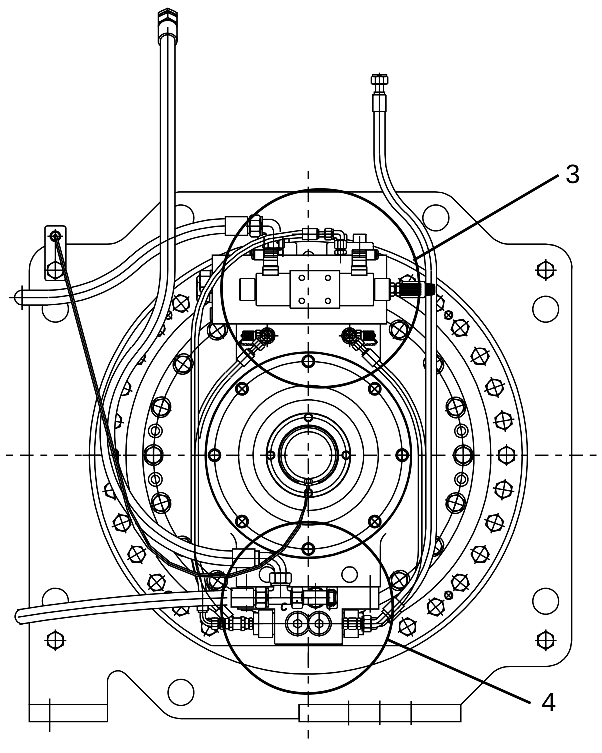

Fig. 9. Hoisting winch, motor side.

Trouble-Shooting Chart, Hydraulic Motors

Fault	Probable cause	Action
The motor does not run.	Mechanical stop in the drive.	Check system pressure. If the pressure has risen to the relief valve setting, remove the load from the drive.
	The motor does not deliver enough torque because the pressure difference across the motor is not great enough for the load.	Investigate the pressure level in the system and correct the setting of the pressure limiting valve if necessary.
	Insufficient or no oil being supplied to motor.	Check the hydraulic system. Check the external leakage of the motor. (The D connection)
Motor rotates in wrong direction.	Oil supply connections to motor incorrectly connected.	Connect the oil supply correctly.
Motor runs jerkily.	Pressure or flow fluctuations in the hydraulic system.	Find the cause in the system or in the driven unit.
Noise in the motor.	The motor is being operated with the charge pressure too low.	Adjust the charge pressure to the correct level. See 9.4 Hydraulic Circuit - gauge connection 1.
	Internal faults in the motor.	Investigate the drain oil, if necessary. Put a magnetic plug in the oil flow and check the material that sticks to the magnet. Steel particles indicate damage. Note that fine material from the castings may be deposited and does not mean internal damage in the motor.
External oil leakage on the motor.	The radial lip seal is worn.	Replace the radial lip seal.

Hydraulic motor with MDA brake

Fault	Probable cause	Action
Insufficient braking torque.	The brake cylinder is not drained in the described manner, excessive counter pressure in drainage line.	Remove the cause of the pressure.
	The brake linings or the discs are worn out.	Dismantle the brake and replace the worn discs.
The brake does not open.	Insufficient brake opening pressure.	For the required opening pressure see the section dealing with brake MDA. See 9.4 Hydraulic Circuit - gauge connection 2.
	Seals or piston damage.	Replace seals. Replace the piston. IMPORTANT! The spring in the brake cylinder is tensioned.

Hydraulic motor with two-speed valve

Fault	Probable cause	Action
Motor only works at one speed.	Pilot pressure low.	Suitable pilot pressure. See 9.4 Hydraulic Circuit - gauge connection 2.
	The valve piston has stuck in single-speed position due to impurities in the oil.	Inspect piston and remove impurities.

Hydraulic Motor COMPACT

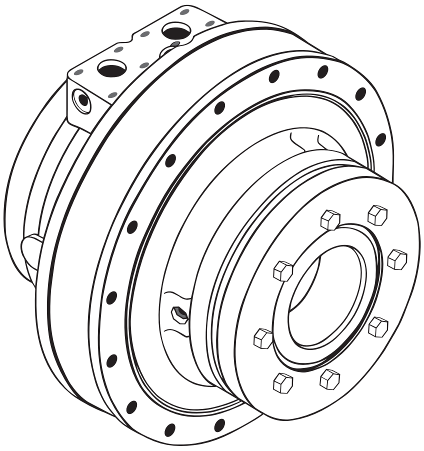

Contents

Functional description 2
Safety precautions 3
Before commissioning 3
Commissioning 4
Periodic maintenance 4
Lifting methods 5
Standing the motor on a flat surface 6
Axial thrust bearing 6
Storage 7
Hoisting motor with 2-speed valve 8
Displacement shift 9
Direction of rotation of motor shaft 10
Draining and venting the motor 10
Oil filters 10
Speed encoder for hoisting motor 11

Functional description

The hydraulic industrial motor COMPACT is of the radial-piston type with a rotating cylinder block/hollow shaft and a stationary housing. The cylinder block is mounted in fixed roller bearings in the housing. An even number of pistons are radially located in bores inside the cylinder block, and the valve plate directs the incoming and outgoing oil to and from the working pistons. Each piston is working against a cam roller.

When the hydraulic pressure is acting on the pistons, the cam rollers are pushed against the slope on the cam ring that is rigidly connected to the housing, thereby producing a torque. The cam rollers transfer the reaction force to the piston which are guided in the rotating cylinder block. Rotation therefore occurs, and the torque available is proportional to the pressure in the system.

Oil main lines are connected to ports A and C in the connection block and drain lines to ports D1, D2 or D3 in the motor housing.

The motor is connected to the shaft of the driven machine through the hollow shaft of the cylinder block.The torque is transmitted by using a mechanical shaft coupling, or alternatively by splines.

The symmetrical design of the motor has made it possible to design it as a two displacement motor. This means that two different speeds can be obtained for a given flow. The simplest way of performing displacement change is by connecting a special valve, direct to the flange face on the connection block.

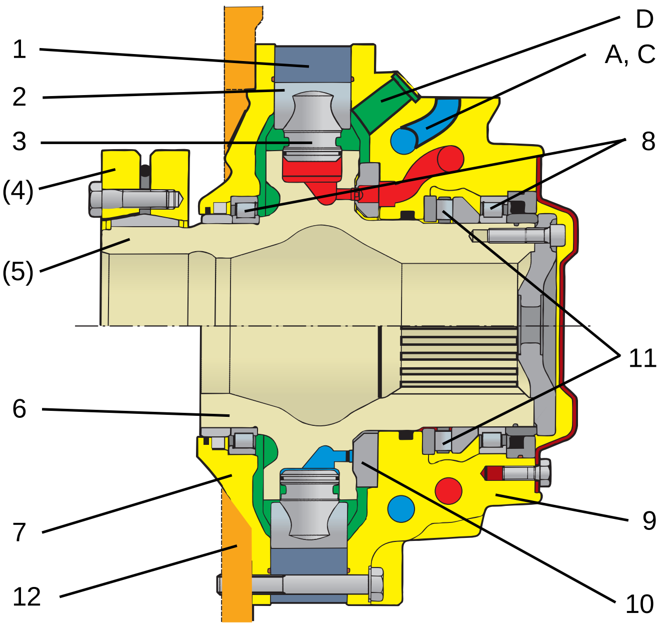

1. Cam ring
2. Cam roller
3. Piston
4. Shaft coupling (not used on winch)
5. Cylinder block / hollow shaft (not used on winch)
6. Cylinder block / spline
7. Front end cover
8. Roller bearing
9. Connection block
10. Valve plate
11. Axial bearing
12. Bracket

A = Inlet or outlet port "A"
C = Inlet or outlet port "C"
D = Drain port

Fig. 1. Compact Motor components.

Safety precautions

It is of high importence that the Safety precautions are always followed, if you are unsure about something, please don´t hesitate to contact your nearest MacGregor-office for advice.

Warning signs

In this instruction you will find the following signs which indicate a potential hazard, which can or will cause personal enjury or substantial property damage. Depending on the probability of the hazard, and how serious the injury or property damage could be, there are three levels of classification.

DANGER!

Is used to indicate the presence of a hazard which will cause severe personal injury, death, or substantial property damage if the warning is ignored.

WARNING!

Is used to indicate the presence of a hazard which can cause severe personal injury, death, or substantial property damage if the warning is ignored.

CAUTION!

Is used to indicate the presence of a hazard which will or can cause minor personal injury or property damage if the warning is ignored.

Before commissioning

Check the following points before commissioning the motor, i.e. before starting the first time:

- Check that the motor is connected to give the correct direction of rotation.
- Select the hydraulic fluid in accordance with the recommendations. See "Lubricants for deck machinery", section 5.
- Fill the motor housing with hydraulic fluid via a filter into the drain outlets D1, D2 or the vent hole (depending on how the motor is mounted).
- Check the drain line to ensure that excessive pressure does not build up in the motor housing.
- Check that the motor is protected from overloads.
- Check that the charge pressure conforms to the charge pressure curve. See "Hydraulic circuit", section 9.4.
- Check that all hydraulic couplings and plugs are properly tightened to prevent leakage.

Commissioning

- During initial starting and the period immediately after it, any hydraulic installation must be regularly and carefully checked at frequent intervals.
- The working pressure and charge pressure must be checked to ensure that they correspond to the contracted values.
- The pressure in the drain line measured at the motor must be less than 3 bar. This pressure limit is important for the life of the motor seals.
- If leakage occurs, correct the fault and carry out new measurements.
- Check all lines, connections, screws, etc. and correct if necessary.
- Check other possible leakage points and replace faulty parts.
- During the start up period, dirt particles in the system are removed by the filters. The filter cartridges have to be changed after the first 100 working hours and after that according to the "Maintenance chart", section 5.

NOTE:

When starting up the motor it is important that the motor output power is limited to 75% of max power according to technical data.

A not run-in motor in combination with dirt particles in the oil can badly affect the sliding surfaces in the motor. This is valid during the first 100 working hours.

Periodic maintenance

When a hydraulic system has been in service for some time, it must undergo periodic maintenance and servicing at intervals which depend on the equipment and the type of duty.

This periodic maintenance must include the following operations:

- Check the hydraulic system for leakage. Tighten the screws, replace faulty seals and keep the drive clean.
- Inspect and clean all air, oil and magnetic filters; replace all filter cartridges for which a filter clogged indication has been given; inspect tank, pump, filters etc. and clean if necessary.
- Check the pressure and temperature of the hydraulic fluid and carry out routine opera- tions. Adjust valves etc. if necessary.
- Check the hydraulic fluid.
- Check that no dirt or other contaminations enter the system during inspection.Check that the outside of the hydraulic motor in an installation is kept free of dirt; thus leakage and faults will be detected earlier.
- We recommend that a running log be kept and that planned inspections are carried out at set intervals.

Lifting methods

Always make sure where the centre of gravity is before any lifting.

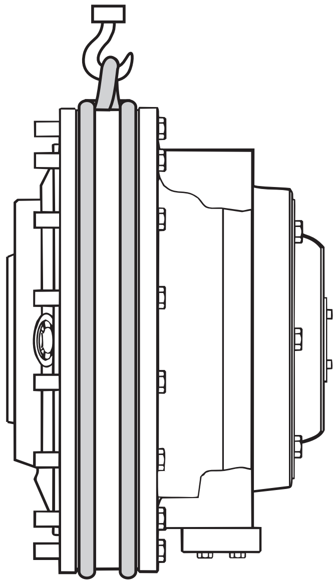

Fig. 2. Lifing motor vertically.

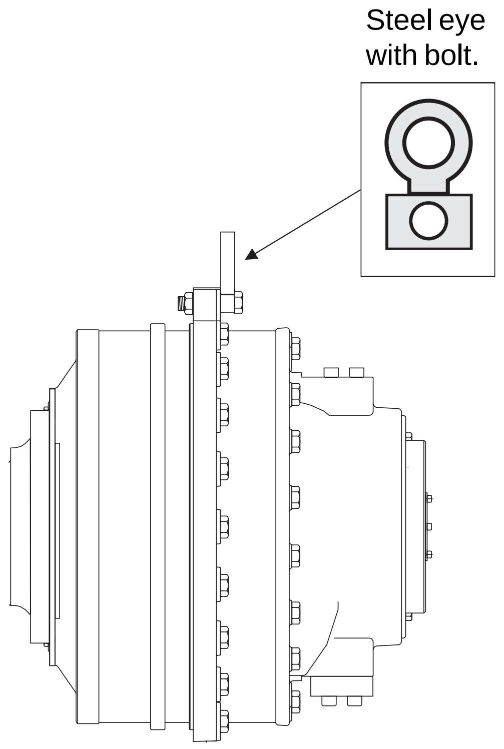

Fig. 3. Lifing motor vertically with steel eye.

Note: *Motor lifted without coupling fitted, danger of slipping off.*

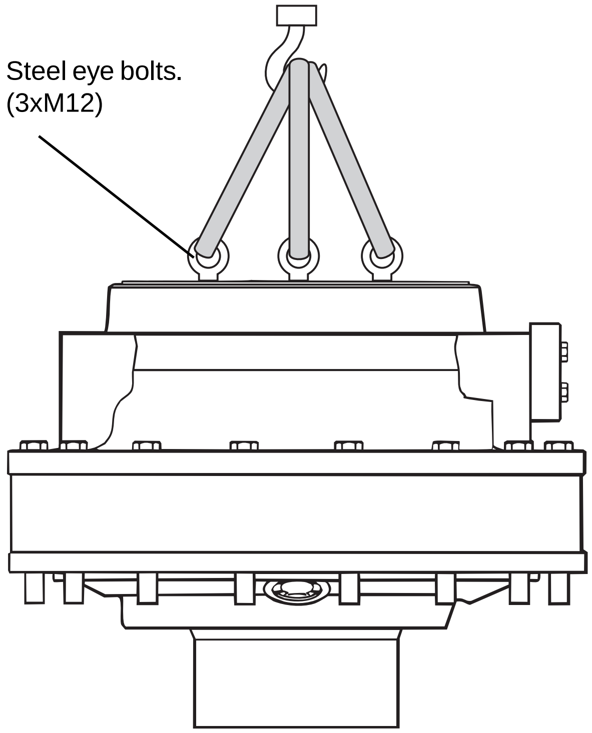

Fig. 4. Lifing motor horizontal.

Note: *The End cover and screws must be removed before mounting the Steel eye bolts. After the lift refit the End cover and screws with torque 81 Nm.*

DANGER!

Always make sure where the centre of gravity is before any lifting. Never stand below the motor.

WARNING!

Always make sure that the lifting equipment is strong enough to handle the weight of the motor.

Standing the motor on a flat surface

When the motor is placed on a flat surface such as a floor, it must stand either on its outer diameter or on the suitably protected end face of the hollow shaft.

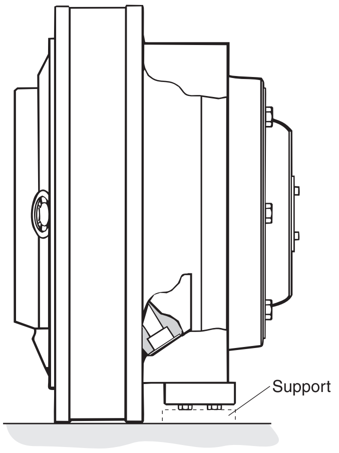

Fig. 5. Motor placed vertically on support.

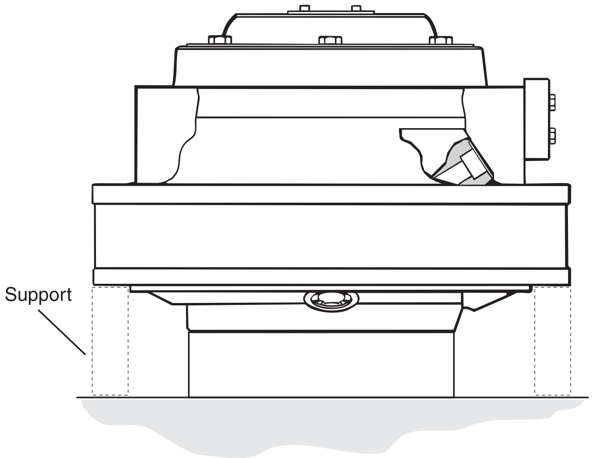

Fig. 6. Motor placed horizontal on support.

WARNING!
Lifting straps must be chosen with reliable saftey margin over the <u>total</u> weight of the lifted object.

NOTE:

The motor must not be placed on the end face of the hollow shaft when the coupling is fitted, since this may cause damage to the coupling.

When in storage, the motor must always be placed on the end face to the hollow shaft. It is also advisable to provide supports at the mounting surface of the motor. See Fig. 6.

Axial thrust bearing

The motors CA100 - CA210 have separate oil for Axial thrust bearing. Oil must be changed every 5 years.

Shell Paolina 680 or equivalent must be used. Volume needed: 0,32 L.

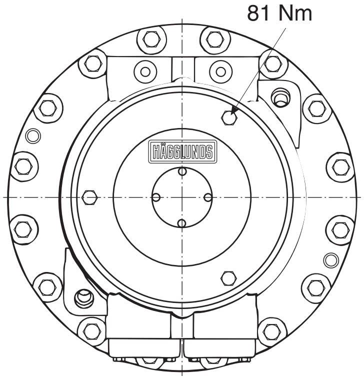

Fig. 7. Removal of motor end cover.

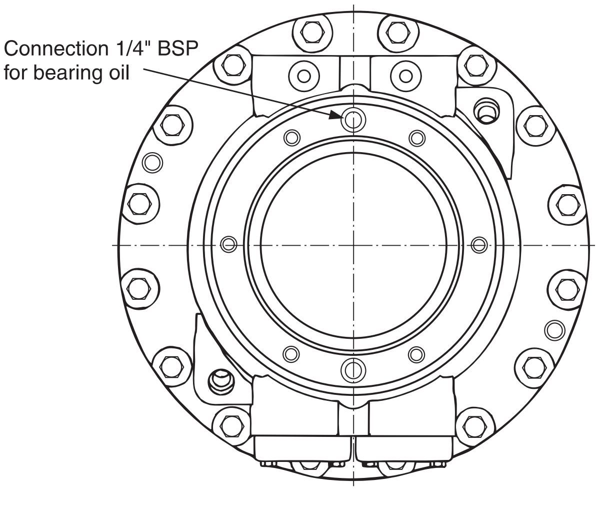

Fig. 8. Connection for bearing oil.

Storage

The motor is delivered with internal protection in the form of an oil film and external protection in the form of an anti-rust film. This provides sufficient protection for indoor storage in normal temperatures for about 12 months.

Note: the anti-rust protection must be touched-up after transport and handling.

If the motor is stored for more than 3 months in unheated premises or more than 12 months in heated premises, it must be filled with oil and positioned as shown in Fig. 9.

Place the motor as shown in Fig 8, fill the motor with filtered oil in the following order: D1, A1, C1. Take extreme care to ensure that no contamination enters the motor with the oil.

Seal connections A and C with the cover plate fitted to the connection surface at delivery. Check that the O-rings or rubber seals are in postion in the cover plate.

Fit the plug to D1, the table, Fig. 11, below states the amount of oil needed to fill the various types of motors.

If the motor is to be stored stationary for a longer period than about 1 month, it must be protected from internal rust. This can be done as follows:

1. Mix anti-rust additive with the hydraulic fluid of the system. Use 5% of Rust Veto Concentrate (manufactured by E F Houghton & Co, Philadelphia, USA). This additive gives rust protection for up to about 1 year, after which time the motor must be turned a few revolutions.
2. If no additives are used, the motor must be regularly turned a few revolutions.
3. If it is not possible to turn the motor, plug all connections, open drain outlet D1 or D2 on the port end housing (or if the motor is mounted vertically. Flushing connection F on the shaft end housing) and fill the motor with hydraulic fluid. See Fig. 10 and 12.

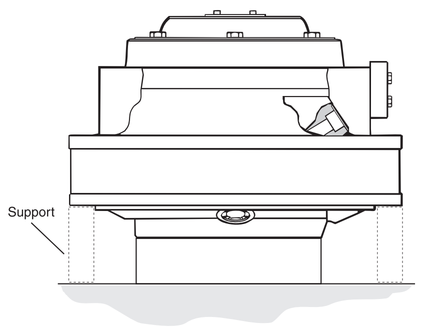

Fig. 9. Motor placed on support for storing.

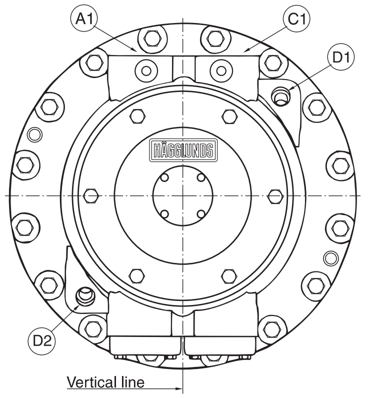

Fig. 10. Motor oil connections.

Motor	Oil volume approx.
CA 100	3.7 litres
CA 140	5.0 litres
CA 210-180	6.8 litres
CA 210	6.8 litres
CA 420-400	14.0 litres

Fig. 11. Motor oil filling volume.

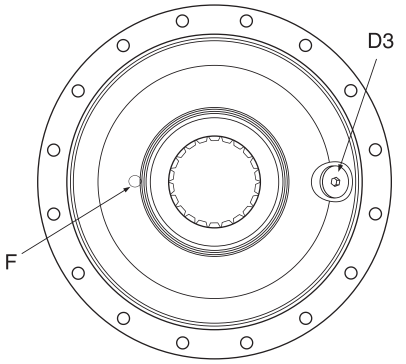

Fig. 12. Flushing connection.

Hoisting motor with 2-speed valve

General

Motors with 2-speed function must be ordered with correct direction of rotation. With wrong direction, load on the piston will be increased 3 times, which can give overheating of the pistons. With the high pressure supply connected to A-port, the motor shaft rotates in the directions shown by arrows. R-motor rotates clockwise, and L-motor counter-clockwise, wiewed from the motor shaft side. If the motor is working in half displacement, and in not prefered "direction of rotation", allowed pressure is max 210 bar.

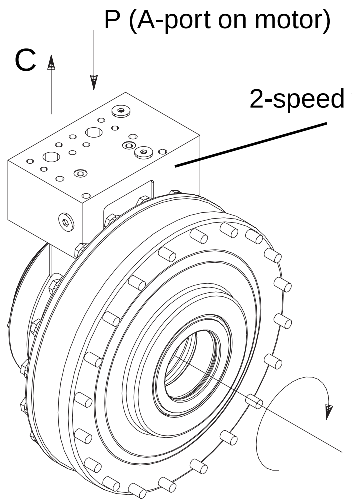

Fig. 13. Motor prepared for valve, direction of rotation, Clockwise = R.

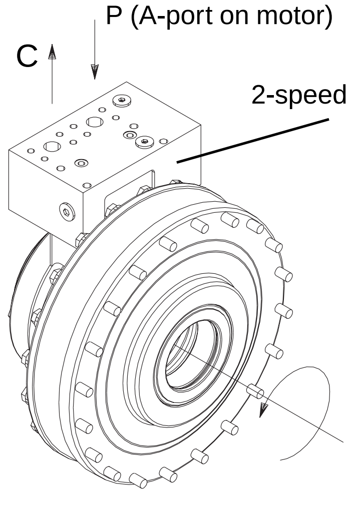

Fig. 14. Motor prepared for valve, direction of rotation, Counter-clockwise = L.

Mounting of 2-speed valve on motor

Remove the protective cover from motor mounting surface, place the O-rings (included in delivery) in their proper position on the valve mounting surface. Use grease to keep O-ring fixed. Mount valve against the motor with the ports in corresponding position.

4 pcs 1/2 UNC x 140 (5,5") included in delivery, strength class 10.9 (ISO 898/1) Tightening torque: 131 Nm.

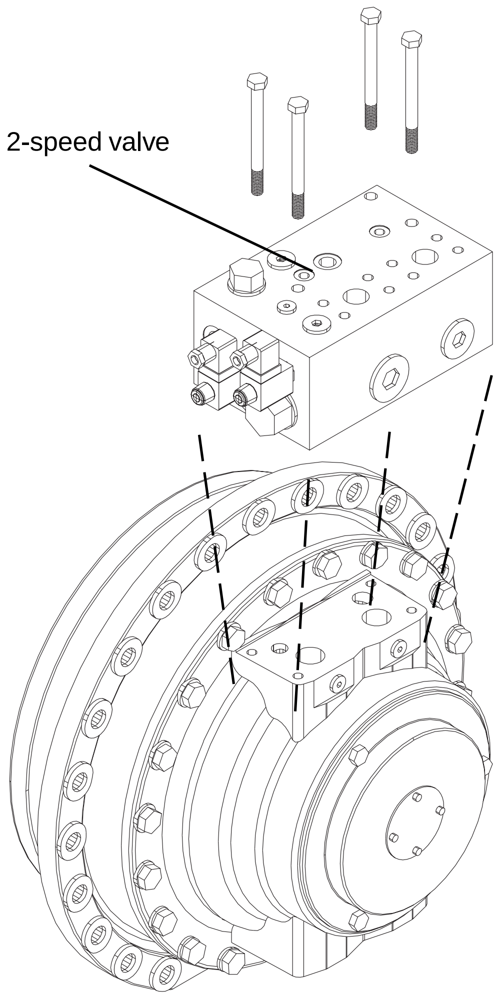

Fig. 15. Mounting of 2-speed valve.

WARNING!

VTCA 600 will not automatic shift from half to full displacement at overload.

Displacement shift

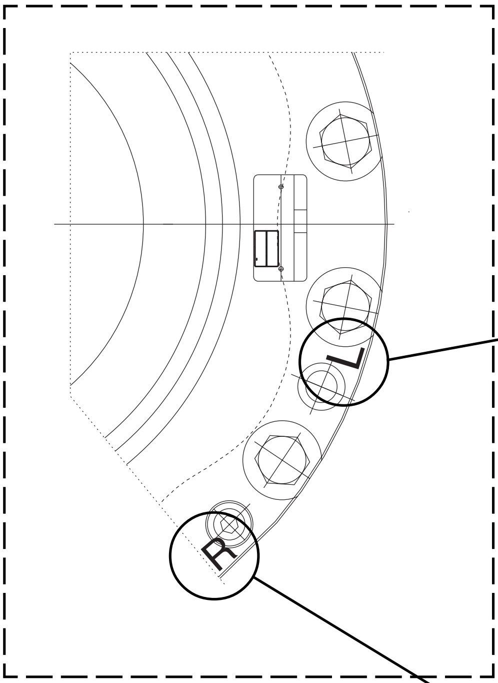

Fig. 16. Changing direction of rotation.

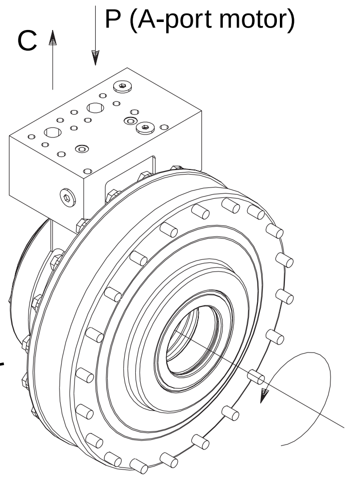

Fig. 17. Left hand motor assembled with the roll pin and screw against "L".

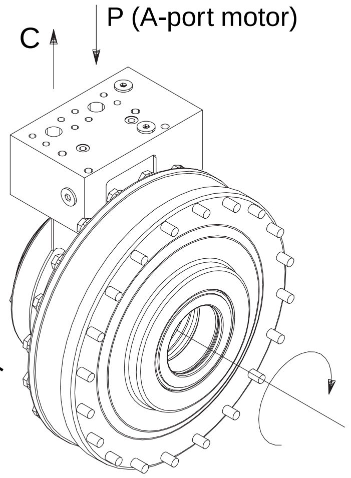

Fig. 18. Right hand motor assembled with the roll pin and screw against "R".

> **CAUTION!**
> **Check direction of rotation.**

Direction of rotation of motor shaft

With the inlet pressure supply connected to A port, the motor shaft rotates in the direction shown by the arrow, anti-clockwise viewed from the motor shaft side.

With the inlet pressure supply connected to C port, the motor shaft rotates clockwise viewed from the motor shaft side.

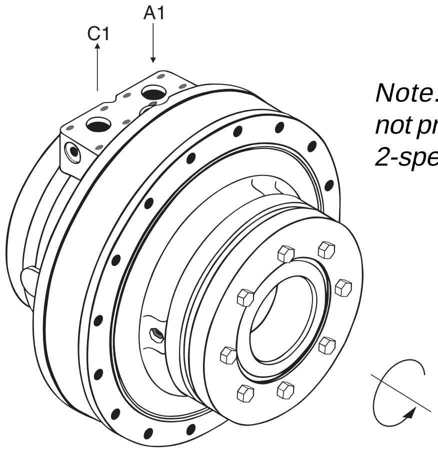

Note: Motor is not prepared for 2-speed valve

Fig. 19. Direction of rotation.

Draining and venting the motor

When the motor is installed with the shaft in the horizontal plane, it can be drained without special provisions for venting. The highest of the three drain outlets D1, D2 or D3 must always be used.

Drain line must be connected direct to the tank with a minimum of restrictions, to ensure that the maximum housing pressure is not exceeded.

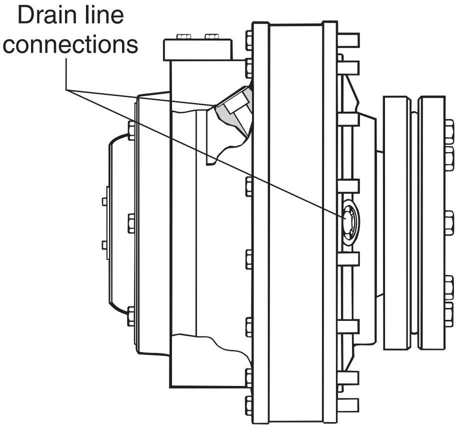

Fig. 20. Motor draining.

Oil filters

Filters must be changed after the first 100 working hours and the second change is to be carried out after 3 months or 500 working hours whichever is earlier. They must then be changed at regular intervals of 6 months or 4000 working hours.

> **CAUTION!**
> **All hydraulic fluids are affected differently. Obtain the advice of your oil supplier or by nearest MacGregor Cranes representative.**

Speed encoder for hoisting motor

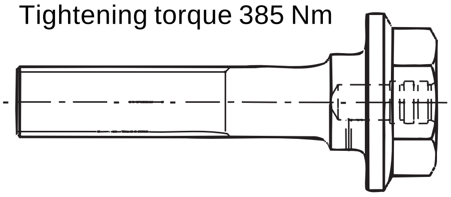

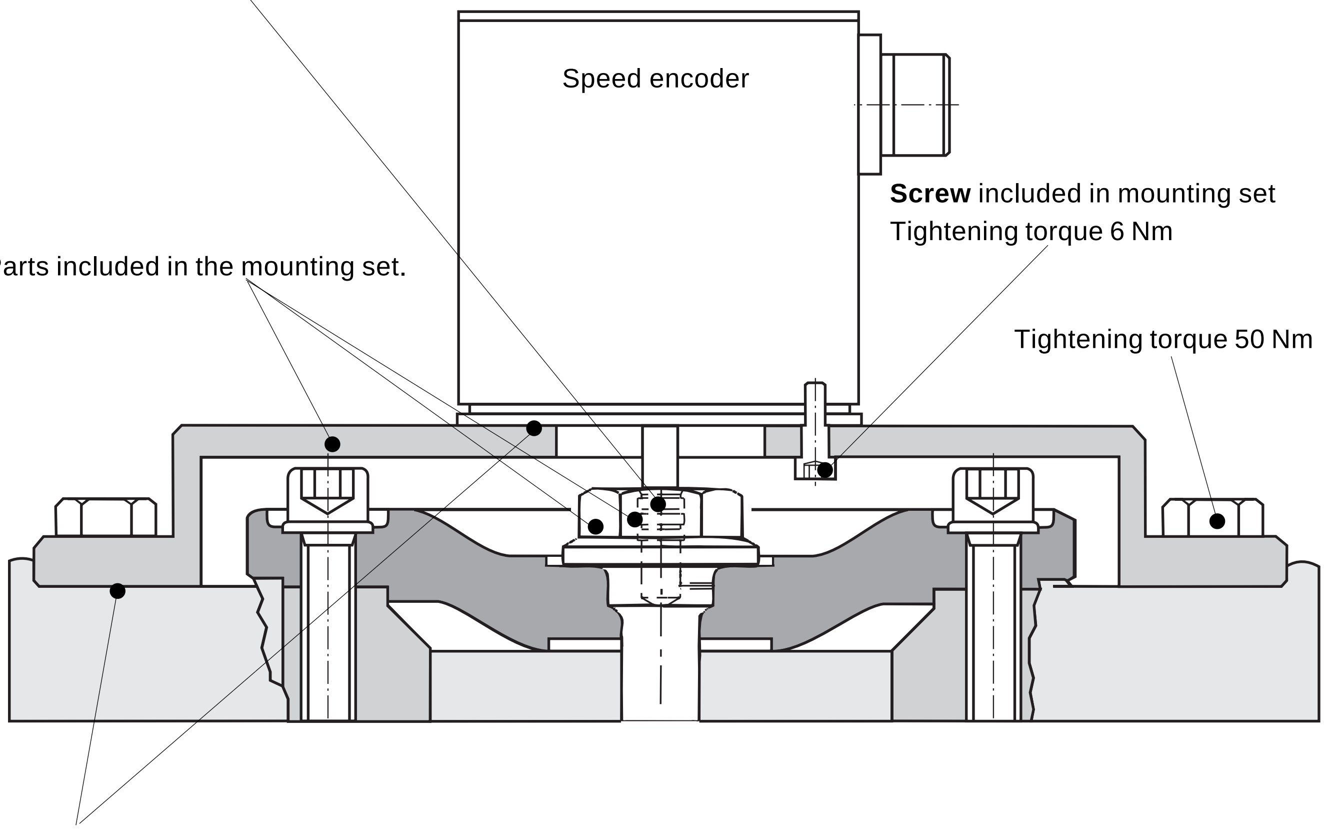

Feed Pump Unit

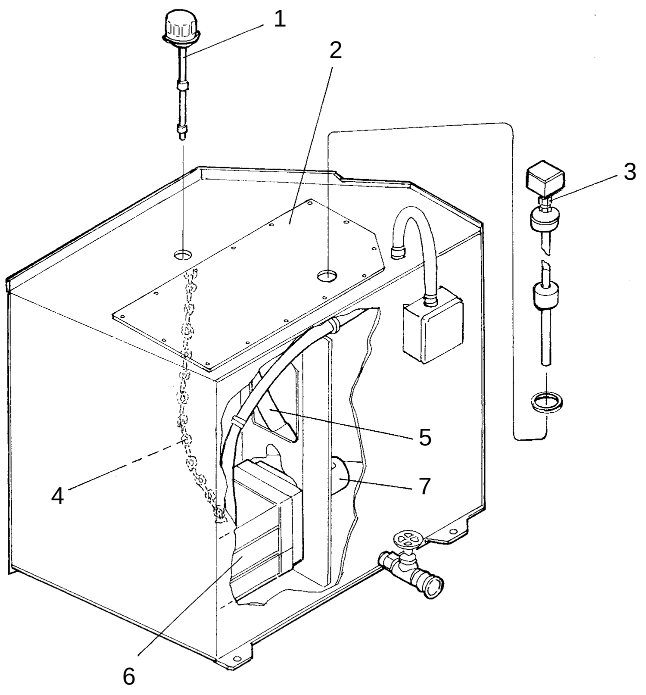

Fig. 1. Tank with feed pump unit

1. Air filter with oil dipstick
2. Tank cover
3. Oil level and temperature guard
4. Chain
5. Hose
6. Electric motor
7. Pump

Introduction

The pump is a screw-type pump with fixed displacement which supplies the feed and control circuits of the crane. The pump is submerged in the hydraulic oil. Thus, one pipe only connects the pump to the hydraulic system.

The pump is separately driven by an electric motor which is mounted in the oil tank.

To overhaul the pump or the electric motor, the entire pump unit - motor and pump - should be taken out.

Caution!

Maintain cleanliness throughout!

1. Removing and dismantling pump unit

- Park crane jib, stop crane, switch off main switch
- Remove tank cover (2)
- Loosen the upper end of the hose (5)
- Disconnect the electric connections to the electric motor (6)
- Lift electric motor (6) with pump (7), using the chain (4)
- Remove mounting screws holding electric motor (6) onto the pump (7)
- Withdraw pump (7) from motor (6).

2. Reassembling pump

- Reassemble pump unit in reverse order to dismantling.
- Replace pump unit. Reinstall and reconnect hydraulic pipes and components. Reconnect electric motor power cable.
- Jog start pump unit to check rotation.

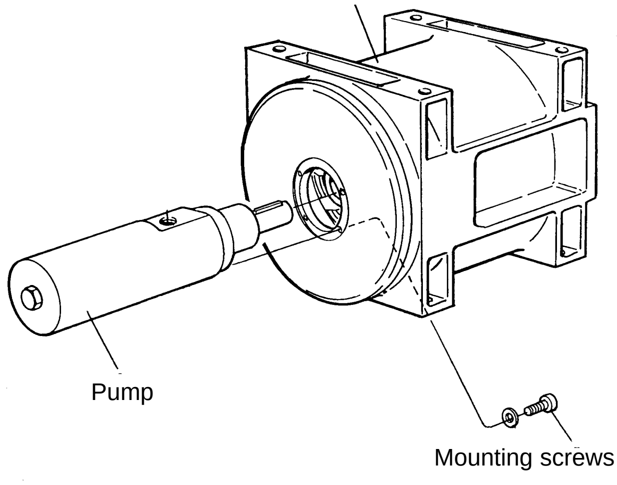

Fig. 2. Pump with electric motor

High Pressure Pumps

General

All pumps are the straight axial piston type with variable displacement controlled by a servo valve mounted on the pump housing.

The pumps are equipped with two adjustments, one for “plussing“ and the other for pressure adjustment (max. working pressure). “Plussing“ of the pumps is done to compensate for internal leakage in pumps and motors to prevent load drops (see Fig. 1).

Function checking/Adjusting of “plussing“

Hoisting winch

- No load on hook.
- Start the crane.
- Install a pressure gauge 0-400 bar at connection 3.1.
- Close valve 1663 (see Fig. 6).
- Move control lever for the hoisting winch to the brake open position (see Fig. 3).
- Check the pressure. See hydraulic circuit diagram.
- If the pressure is incorrect, an adjustment must be made using the “zero setting“ screw (see Fig. 1). If there is more than one hoisting pump, the lower pump should be adjusted.
- Stop the crane.
- Open valve 1663.

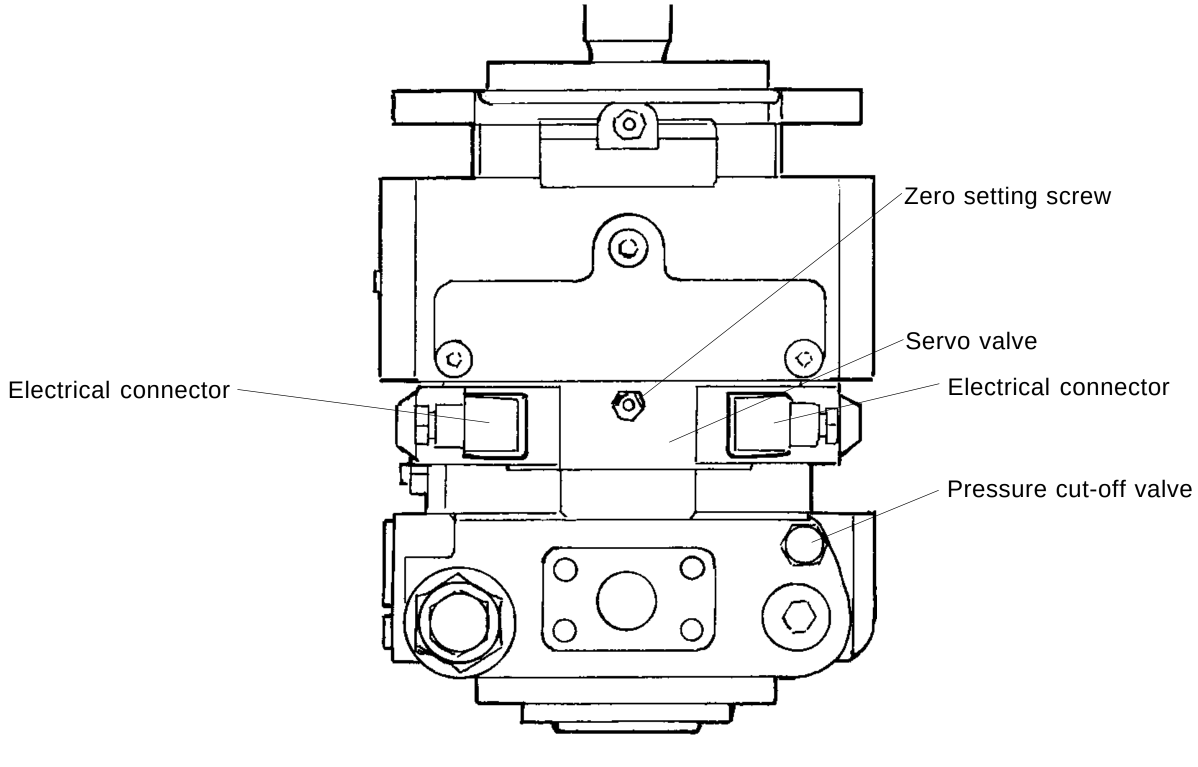

Fig. 1. Pump A4VG 125.

Luffing winch

- No load on hook.
- Park the crane or move the hook block close to the jib top.
- Stop the crane.
- Open main switch HA.
- Open box MB and pull out the first electronic card (see Fig. 2).
- Close main switch HA.
- Remove the two electrical connectors from the pump (see Fig. 1).
- Start the crane.
- Install a pressure gauge, 0-400 bar, in outlet 5.1, (see Fig. 7).
- Close valve 2661 (see Fig. 7).
- Move the control lever for the luffing winch for luffing-in (see Fig. 4).
- Check the pressure. See hydraulic circuit diagram.
- If the pressure is incorrect, an adjustment must be made using the “zero setting“ screw (see Fig. 1).
- Stop the crane.
- Open main switch HA.
- Reinstall the first electronic card (see Fig. 2).
- Close box MB.
- Close the main switch HA.
- Reinstall the electrical connectors on the pump.
- Open valve 2661.

Slewing machinery

- No load on hook.
- Stop the crane.
- Open main switch HA.
- Open box MB and pull out the first electronic card (see Fig. 2).
- Close main switch HA.
- Remove the electrical connectors from the pump (see Fig. 1).
- Start the crane.
- Move the control lever for the slewing machinery for slewing left or right (see Fig. 5).
- Ensure that the crane does **not** move in any direction.
- If the crane does move, an adjustment must be made using the “zero setting“ screw (see Fig. 1).
- Stop the crane.
- Open main switch HA.
- Reinstall the first electronic card (see Fig. 2).
- Close box MB.
- Close the main switch HA.
- Reinstall the electrical connectors on the pump.

Installation of a new servo or complete pump

When a new servo unit or a complete pump is installed, a function check of the "plussing" has to be carried out. Proceed according to “Function checking/Adjusting of “plussing”.

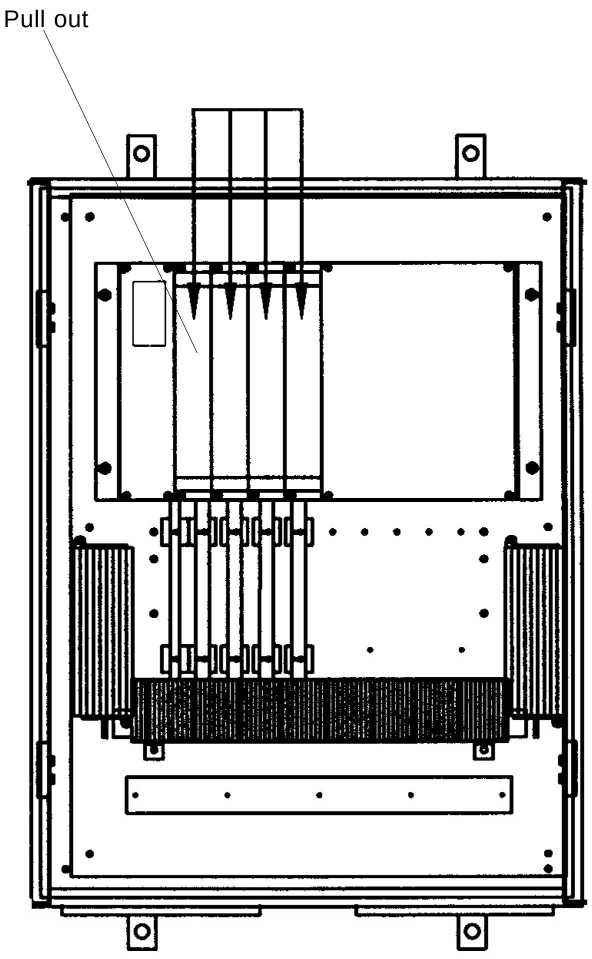

Fig. 2. MB-Box

Adjustment of cut-off valves

Hoisting pump

- This valve has a setting of 100 - 420 bar. By turning the adjustment screw 1 revolution the setting is changed approx. 90 bar.
- Back off the adjustment screw.
- Install a pressure gauge 0-400 bar at connection 3.1.
- Close valve 1663 (see Fig. 6).
- Drive in high speed mode and at max. 25 % speed.
- Adjust the pressure cut-off valve on pump 1111. See pressure setting list for hydraulic circuit diagram 1111-10.
- Open valve 1663.

With two hoisting pumps

- Turn the valve on pump 1112 to its maximum setting.
- Adjust valve on pump 1111 as described in above paragraph "Hoisting pump".
- Adjust valve on pump 1112 by backing off the setting screw until a little lower pressure can be read.
- Turn the screw 1/4 revolution.

Luffing pump

- Start the crane.
- Install a pressure gauge, 0-400 bar, in outlet 5.1, (see Fig. 6).
- Close valve 2661 (see Fig. 7).
- Back off adjustment screw (pressure cut-off valve).
- Drive the crane with 25 % speed (see Fig. 4).
- Adjust pressure cut-off valve 2111-10. See hydraulic circuit diagram for pressure setting.
- Stop the crane.
- Open valve 2661.

Slewing pump

- Start the crane.
- Install a pressure gauge, 0-400 bar, in outlet 6.1 or 6.2.
- Close valve 3661 (see Fig. 8).
- Back off adjustment screw.
- Drive crane with 25 % speed (see Fig. 5).
- Adjust pressure cut-off valve 3111-10, see hydraulic circuit diagram for pressure setting.
- Stop the crane.
- Open valve 3661.

Installation of a complete new pump

When a new pump is installed, the pressure cut-off valve has to be adjusted (see "Adjustment of the pressure cut-off valves").

N.B. These valves are sealed and the seals must not be broken.
Adjustment and replacement of these valves must be done by qualified personnel only.
Contact the Service Department at MacGREGOR Cranes for information about valve adjustment.

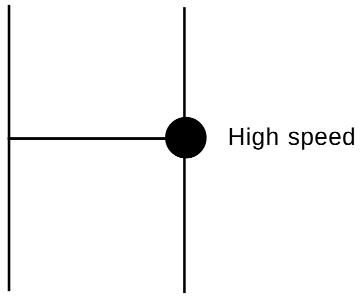

Fig. 3. Hoisting position.

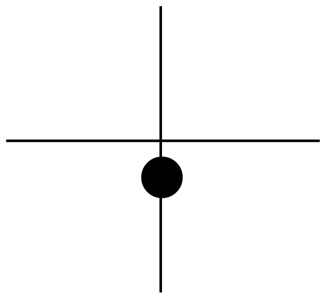

Fig. 4. Luffing position.

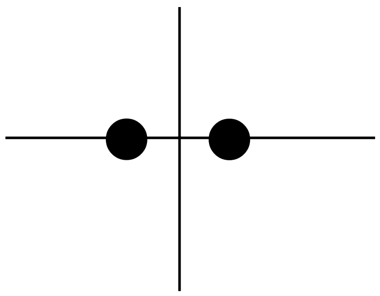

Fig. 5. Slewing left or right.

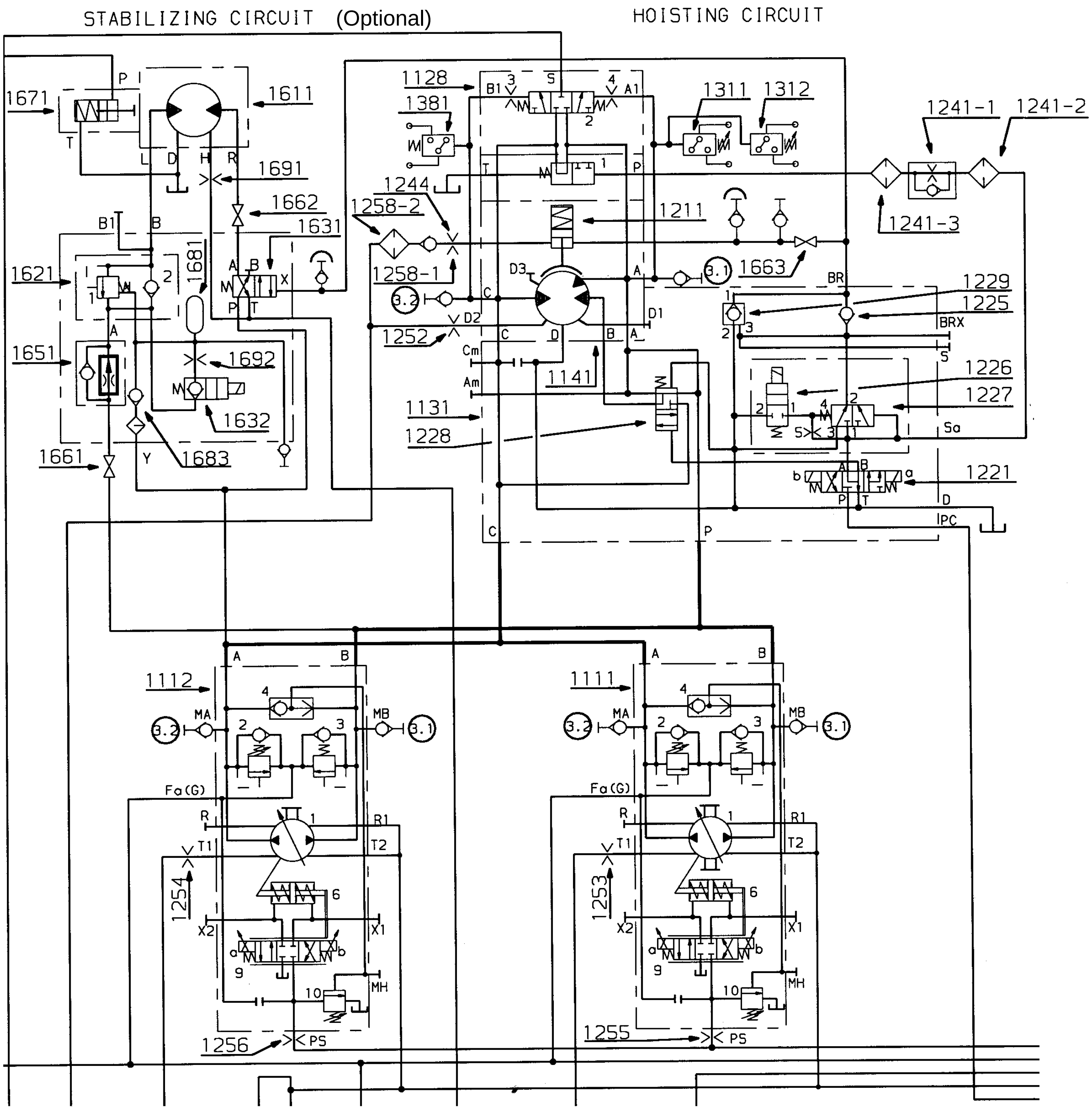

Fig. 6. Part of hoisting circuit

LUFFING CIRCUIT

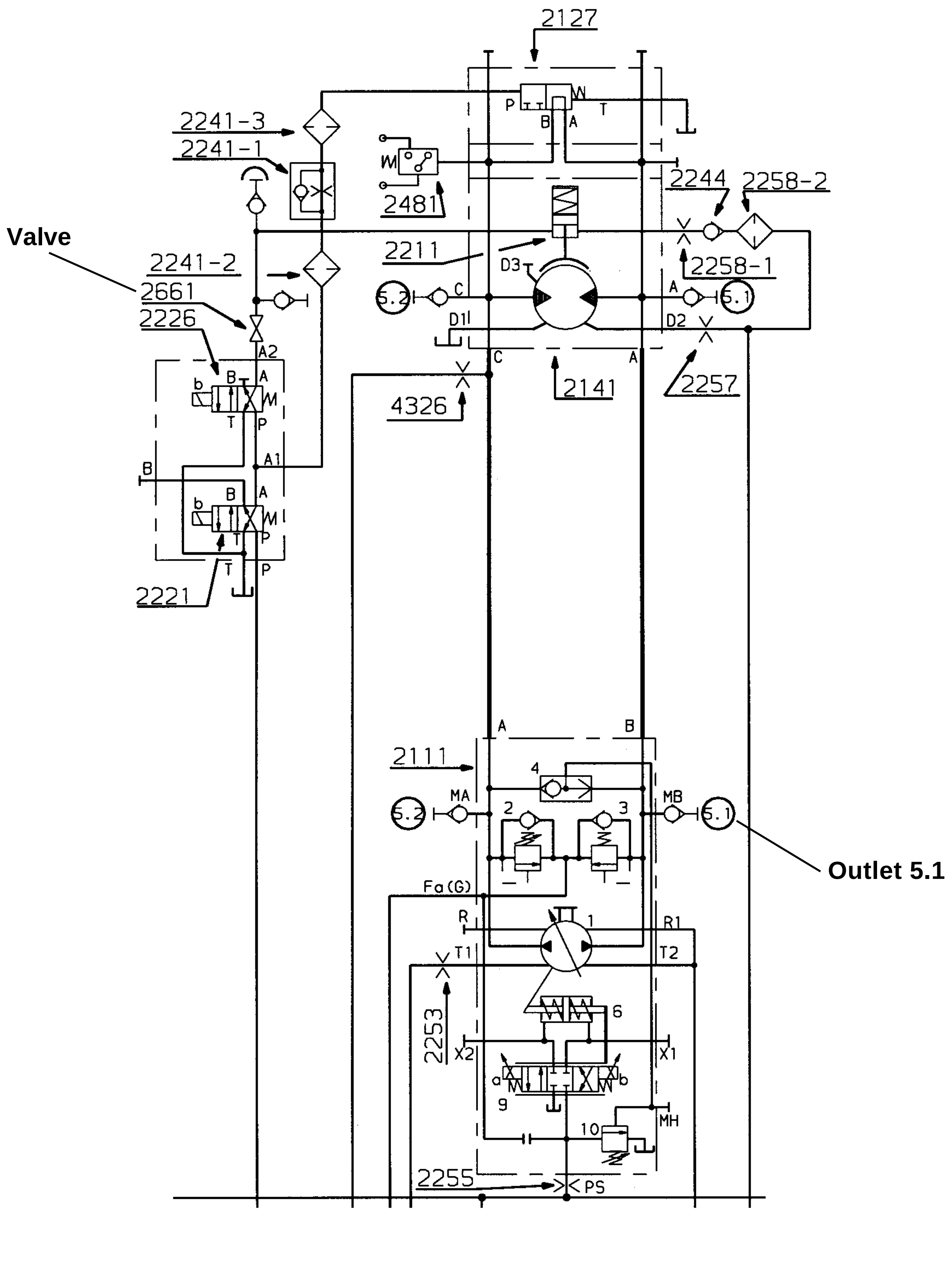

Fig. 7. Part of luffing circuit

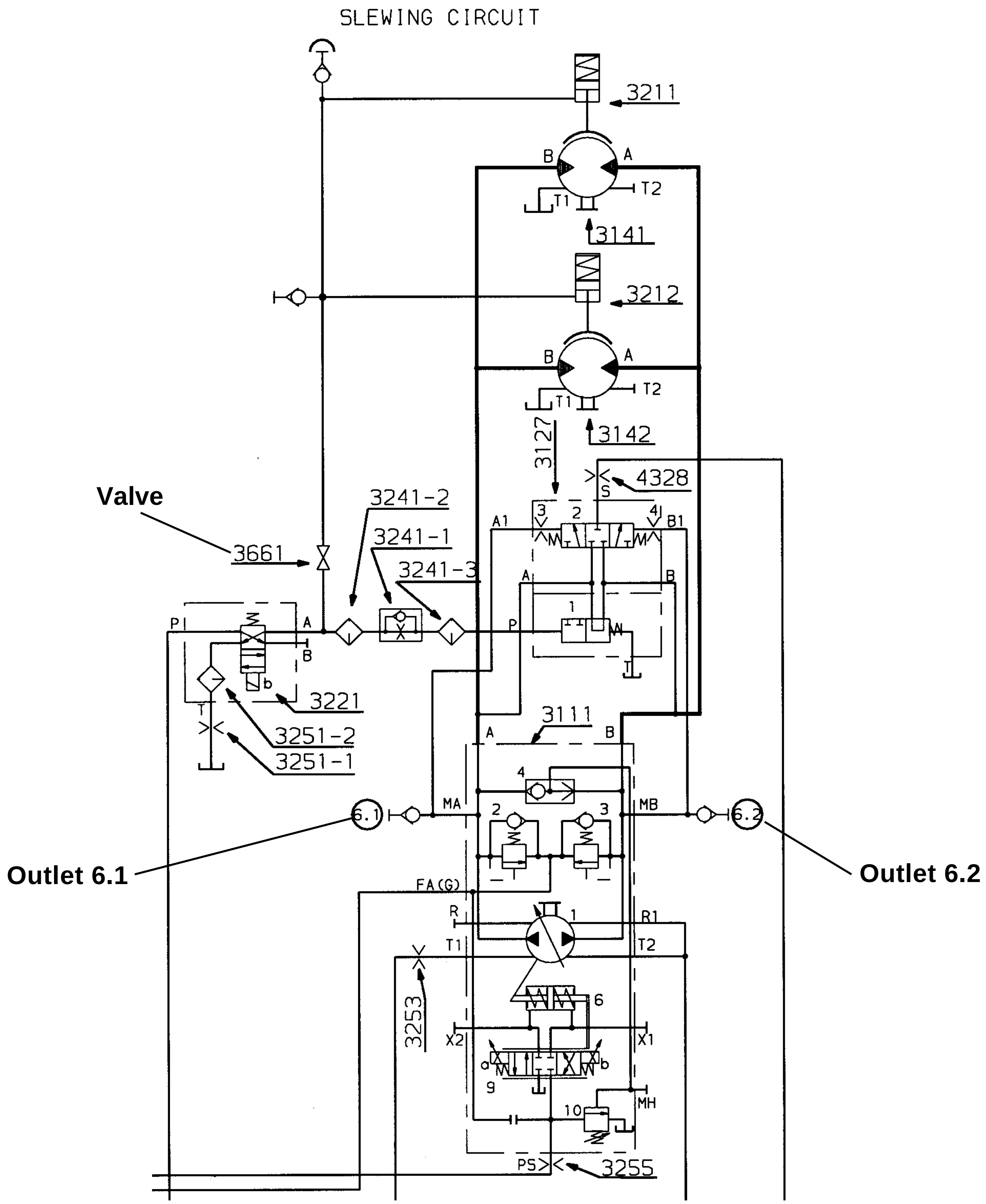

Fig. 8. Part of slewing circuit.

Hydraulic Piston Accumulator 388 0362-801, -802, -803

Function

A hydraulic accumulator functions as a source of pressurized fluid. Conversely, when the pressure falls off, the accumulator supplies oil to the hydraulic system.

N.B. - Do not bleed hydraulic accumulators!

Charging equipment

To charge hydraulic accumulators, art. no. 388 0362, MacGREGOR Cranes supply a charging valve and associated equipment, Fig. 2, comprising:

- a three-way valve
- a 0-25 bar pressure gauge
- a 1-metre hose with a W24 connector for nitrogen cylinder
- two reduction connectors, W24/32M and W24/14D.

Charging pressure for 388 0362-801: 15±1 bar at 20°C.
Charging pressure for 388 0362-802: 28±1 bar at 20°C.
Charging pressure for 388 0362-803: 20±1 bar at 20°C.

Charging hydraulic accumulators with nitrogen (N_2)

All crane motors must be immobilized.

1. Dismount valve guard and unscrew the protective cap from gas valve.
2. New design, see Fig. 1. Connect the three-way valve to the gas valve of the accumulator. The bleed valve should be tightly closed.

2a. Old design, see Fig. 2. Connect the three-way valve to the gas valve of the accumulator. The filling screw of the three-way valve shall be backed out completely, and the bleed valve tightly closed.

3. Connect the charging valve, see Fig. 3 to the reducing valve at the nitrogen cylinder.
4. Back out the adjusting screw of the reducing valve on the nitrogen cylinder until the valve spring is unloaded.
5. Open the nitrogen cylinder valve.
6. Adjust the reducing valve to the correct charging pressure.

New design

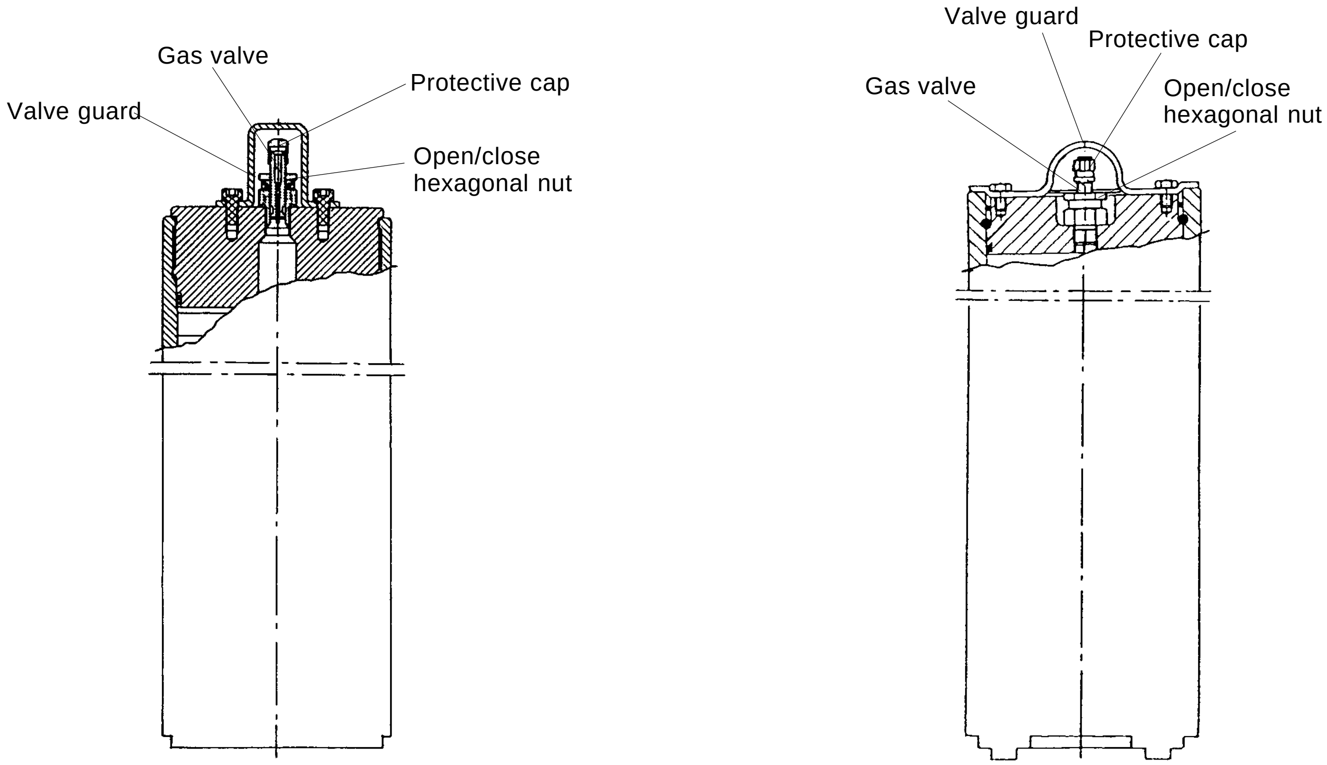

Fig. 1. New design. Piston accumulators 388 0362-801, -802, -803.

7. New design, see Fig. 1. Open the hexagonal nut on the gas valve counter-clockwise to stop, by using a 19 mm fork wrench.
7a. Old design, see Fig. 2. Screw in the filling screw of the three-way valve but not so tightly that the gas valve of the accumulator is damaged.
8. Fill nitrogen.
9. New design, see Fig. 1. Check the nitrogen pressure when the temperature and the pressure of the gas become stable. When the correct charging pressure has been obtained, close the gas valve by turning the hexagonal nut clockwise by use of the 19 mm fork wrench.
9a. Old design, see Fig. 2. Check the nitrogen pressure when the temperature and the pressure of the gas have become stable. When the correct charging pressure has been obtained, again back out the filling screw of the three-way valve.
10. Close the nitrogen cylinder valve.
11. Open the bleed screw of the three-way valve to discharge the pressure in the filling hose.
12. Adjust the reducing valve to zero.
13. Unscrew the charging equipment from the nitrogen cylinder and from the accumulator.
14. Check tigthness of accumulator gas valve, using soap water.
    N.B. - The protective cap shall be fitted with an O-ring which must fit correctly into its groove.
15. Screw on protective cap of the gas valve.
16. After charging the accumulator, fit a lable carrying the charging date and signed by the operator.

N.B. For charging, it is not necessary to install a reducing valve on the nitrogen cylinder.

Old design

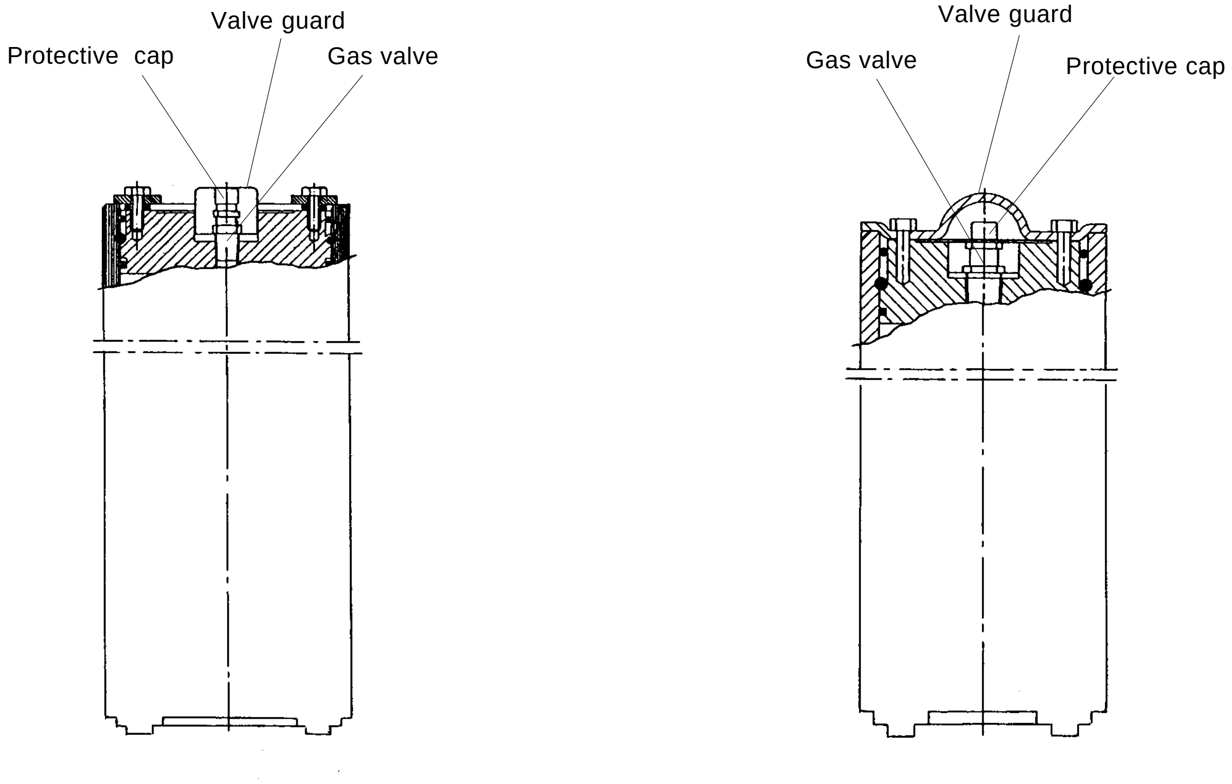

Fig. 2. Old design. Piston accumulators 388 0362-801, -802, -803.

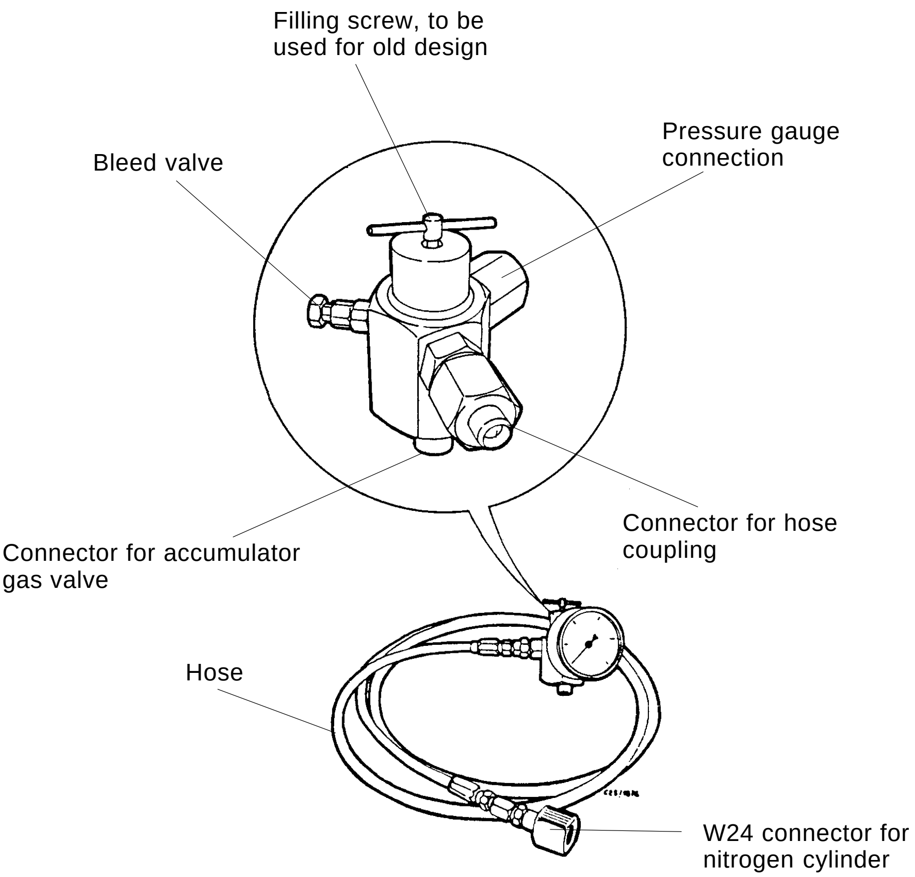

Fig. 3. Charging equipment.

Hydraulic Brake
MDA

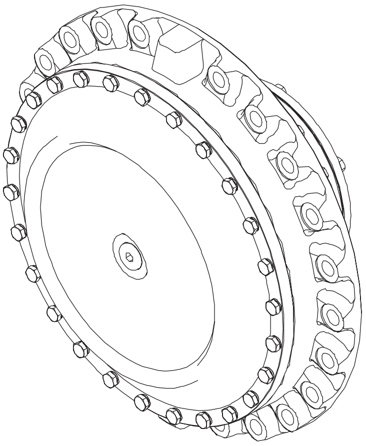

Contents

Functional description 2
Safety precautions 2
Technical data 3
Lifting of MDA brake 4
Disassembly and assembly of MDA 14 & 21 5
Disassembly and assembly of MDA 42 6

Functional description

The brake is of the fail safe multi disc type. During normal operation hydraulic pressure keeps the discs within the brake separated, allowing shaft rotation. If hydraulic pressure is lost, springs force the discs together, stopping rotation of the shaft.

- The brake is intended to be used as a parking brake or emergency brake.
- The hydraulic connection must be on top of the brake, to avoid air. Air gives a slow function of the brake.

Safety precautions

It is of high importance that the Safety precautions are always followed, if you are unsure about something, please don't hesitate to contact your nearest MacGREGOR-office for advice.

Warning signs

In this instruction you will find the following signs which indicate a potential hazard, which can or will cause personal enjury or substantial property damage. Depending on the probability of the hazard, and how serious the injury or property damage could be, there are three levels of classification.

DANGER!
Is used to indicate the presence of a hazard which will cause severe personal injury, death, or substantial property damage if the warning is ignored.

WARNING!
Is used to indicate the presence of a hazard which can cause severe personal injury, death, or substantial property damage if the warning is ignored.

CAUTION!
Is used to indicate the presence of a hazard which will or can cause minor personal injury or property damage if the warning is ignored.

Technical data

MDA 14 and 21

Max external load: 200 kN
External load: 110 kN according to FEM M5: (L2:T5).

MDA 42

Max external load: 253 kN
External load: 142 kN according to FEM M5: (L2:T5).

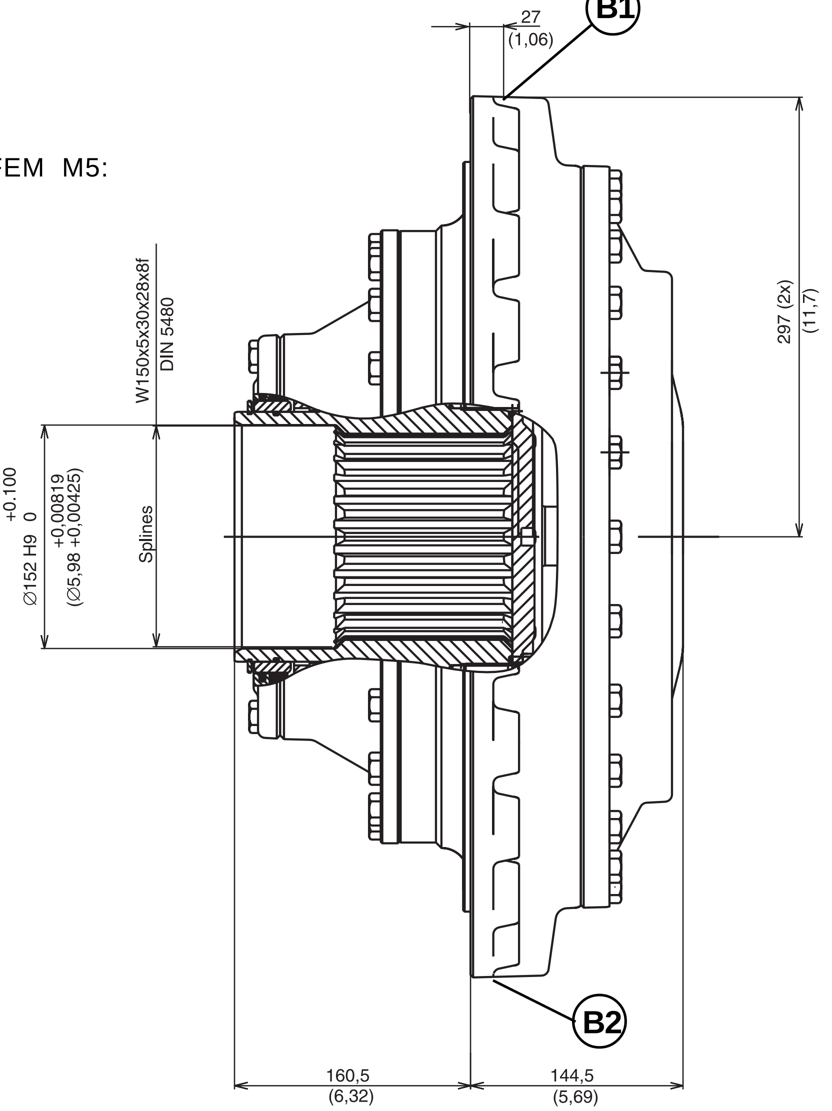

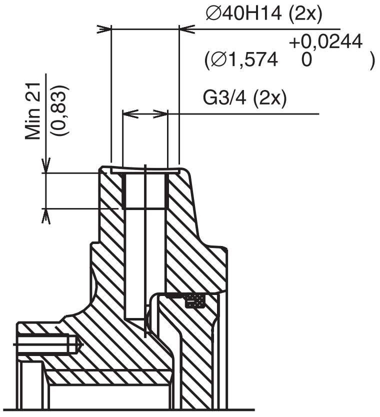

B1 and B2

Brake	Motor	Braking torque*	Begins to open at	Fully open at	Displ.	Max. allowed pressure	Weight
		Nm	bar	bar	cm³	bar	kg
**MDA 14**	CA 140	54 000	15	20	300	50	230
**MDA 21**	CA 210	77 500	15	20	300	50	230
**MDA 42**	CA 420	140 500	15	20	400	50	510

* Valid at wet running. Friction coefficient μ = 0,12. Pressure in brake cylinder = 0 bar.
Hydraulic oil with AW-additives.

Lifting of MDA brake

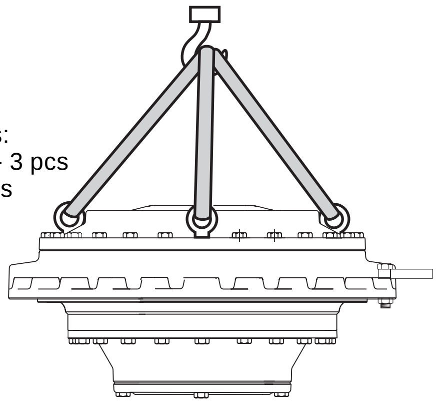

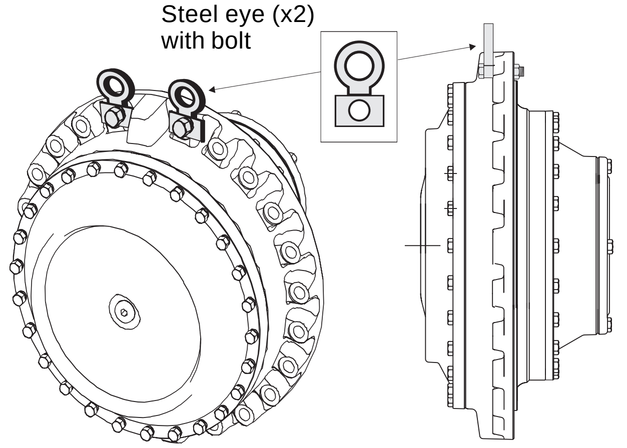

WARNING!

Always make sure that the lifting equipment is strong enough to handle the weight of the brake.

Disassembly and assembly of MDA 14 & 21

1. Start the disassembly by slightly loosening the screws (40). Not more than 1 turn each. Continue with 1 turn of each screw around the cover until the pre-load of the Belleville springs (315)(Cup springs) is zero. After that the screws can be removed and the Brake cover (314) lifted off.
2. The Brake piston (313) can be removed by installing 3 screws (M8) in the centre holes and be lifted off by using the screws.
3. Inner and outer discs (318, 320) can be removed and the spacer (3xx) in place can be removed.
4. Loosen the screws (43), and take off the seal retainer (25).
5. Control the seal and mount seal retainer (25) as figure. Oil the screws (43) and assemble with torque 114 Nm.

6. Mount spacer for MDA 14. Mount first one outerdisc, then innerdisc, outerdisc until all discs are mounted. MDA 14 has 8 outerdiscs and 7 innerdiscs. MDA 21 has 11 outerdiscs and 10 innerdiscs.
7. Grease the seals with Texaco Multifak EP2 or an equivalent grease without solid additives and mount the piston (313).
8. Grease the springs (315) with Texaco Multifak EP2 or an equivalent grease without solid additives, put the springs on the Brake cover (314). The springs shall be mounted against each other in the innerdiameter.
9. Mount the brake cover (314) on the brake, oil the screws and assemble the screws. Start mounting by slightly tightening the screws, no more than one turn each. Continue with one turn of each screw around the cover, tightening torque is 114 Nm.

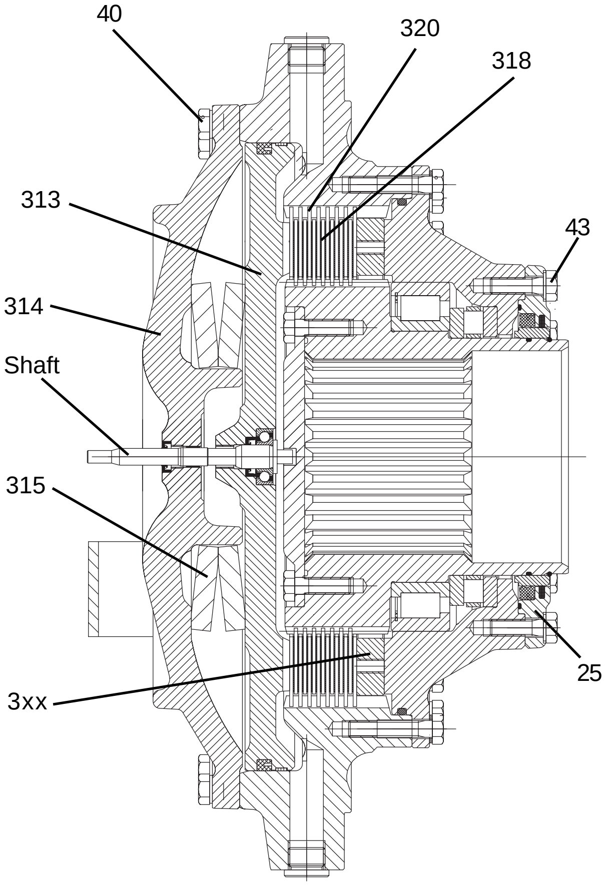

DANGER!
Please read this instruction carefully before starting the disassembly!

Disassembly and assembly of MDA 42

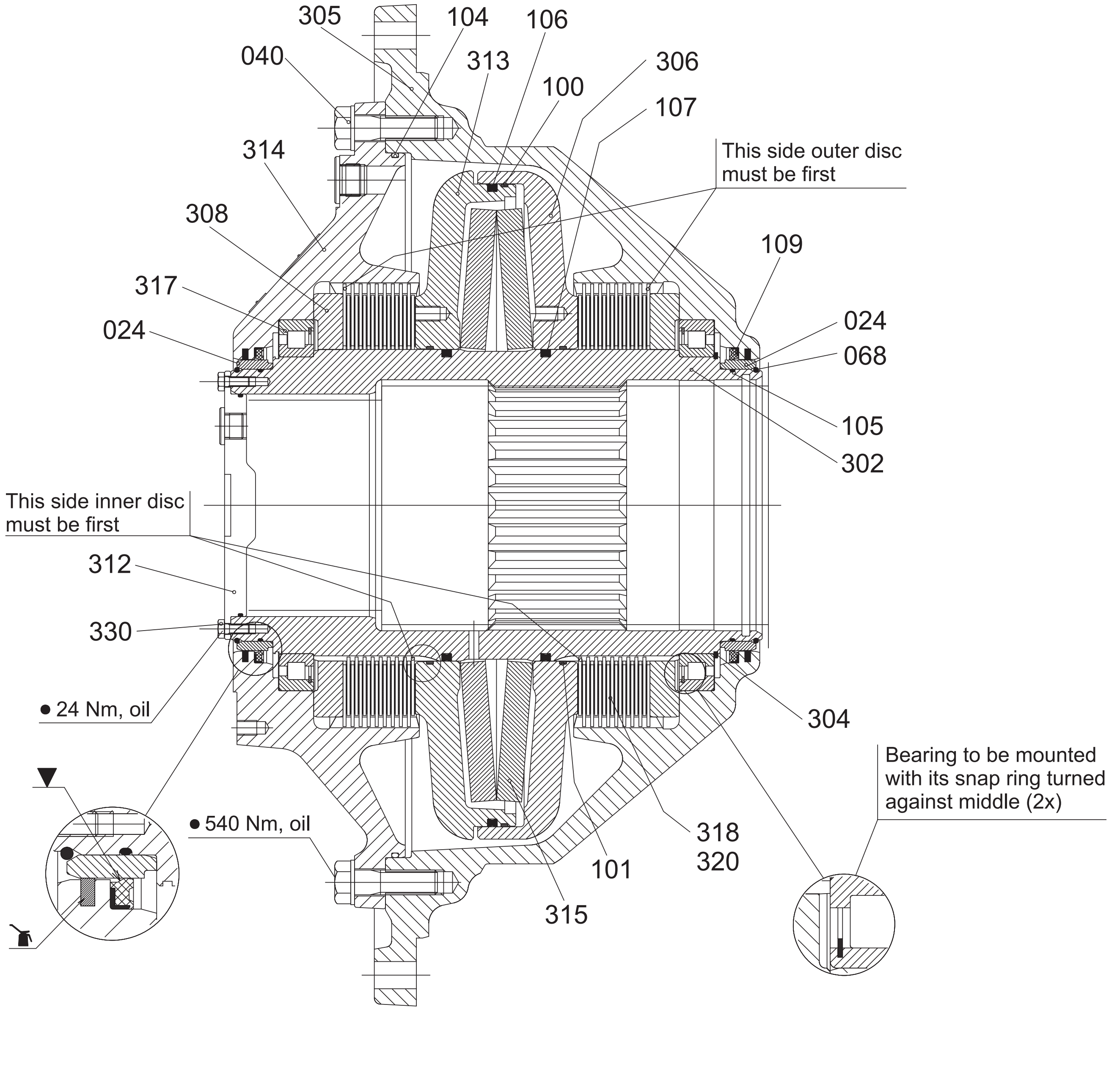

DANGER!

Please read this instruction carefully before starting the disassembly!

Disassembly of MDA 42

1. Start the disassembly by slightly loosening the screws (40). Not more than 1 turn each. Continue with 1 turn of each screw around the cover until the pre-load of the Belleville springs (315) cup springs is zero. After that the screws can be removed and the brake cover (314) lifted off, use lifting eye M16.
2. Remove the cover (312) by loosening the screws (330).
3. Remove the lock ring (068). Remove the wear ring (024). Use a screwpuller.
4. Use tool D 30170 D 119, screwpuller, to remove the bearing (317).
5. Remove the spacer ring (308). Inner and outer discs (318, 320) can be removed.
6. The inner piston (313) can be removed, be careful with the guide strings. Use lifting eye M12.
7. Remove the two Belleville springs (315).
8. Remove the outer piston (306), be careful with the guide strings. Lifting eye M12.
9. Lift out the disc centre (302) and turn it 180°, use lifting eyes M8.
10. Remove the lock ring (068). Remove the wear ring (024). Use a screwpuller.
11. Remove the lock ring (304).
12. Use tool D 30170 D 119, screwpuller, to remove the bearing (317).
13. Remove the spacer ring (308).
14. Inner and outer discs can now be removed.

Assembly of MDA 42

1. Mount disc set (318, 320) on the disc centre (302), threaded side M8 (6x) downwards. Note: inner disc against piston, then outer disc, inner disc until all discs are mounted.
2. Mount the spacer ring (308).
3. Mount the bearing (317).
   Note: the snap ring turned against middle. The bearing must be heated up before mounting. Mount the lock ring (304).
4. Grease O-ring (105). Mount wear ring (024), wear ring must be heated up before mounting. Mount lock ring (068).
5. Grease the lip seal (109) in the brake house (305), use Texaco Multifak EP2, Shell Grease 1344 LiEP 2, or an equivalent grease without solid additives.
6. Turn the disc centre 180°, threaded side M8 (6x) upwards.
7. Grease O-ring (105). Mount wear ring (024), wear ring must be heated up before mounting. Mount lock ring (068).
8. Centre the discs (318, 320) around the disc centre (302) and adjust them so that all spline teeth are above each other. Use a round bar (approx. 7-9 mm) and insert it in the spline gap, hit gently with a hammer on it, continue to do so on several places around the diameter.
9. Lift down the disc centre (302) into the brake house (305), use lifting eye M8. Check that all discs are in position.
10. Before lifting down outer piston (306), grease piston seal (107) and guide string (101). Use lifting eyes M12.
11. Grease the springs (315) before mounting them.

12. Grease piston seal (106, 107) and guide string (100, 101) on inner piston (313) before mounting it.

13. Mount disc set (318, 320), see paragraf No. 1.

14. Centre the discs around the disc centre, see paragraf No. 8.

15. Grease the lip seal (109) and O-ring (104) on cover (314).

16. Mount the cover, use lifting eye M16, check that all discs are engaged. Turn the cover with the discs so that the centre line between the two oil plug holes is parallel with the flat side of the outer diameter of the brake. Check that you can mount a screw (040).

17. Take off the cover carefully without touching the discs.

18. Mount spacer ring (308) and centre it around disc centre.

19. Mount the bearing. Note: the snap ring turned against middle. The bearing must be heated up before mounting.

20. Mount the cover, oil the screws, and assemble the screws and washers. Start mounting by slightly tightening the screws, no more than one turn each. Continue with one turn of each screw around the cover until final tightening torque 540 Nm is reached.

21. Mount the cover (312), oil the screws and assemble. Tightening torque 24 Nm.

Tightening Torques
Studs/Screws and Bolts

Introduction

This instruction applies to studs, screws and bolts generally, if separate recommendations are not given. Apply Molycote 1000 Lubrication on the threads and under the bolt head. For stainless elements and hydraulic pipe couplings apply Gleitmo paste.

Slewing bearing, studs/screws

See drawings "Slewing bearing mounting" and "Slewing bearing yard mounting" in Section 9.3.

Note!
Threads that have been rolled after hardening have a lower surface notch indicating that the permitted tension amplitude at fatigue can be about doubled compared to threads that have been rolled before hardening. If the screw has been loaded beyond the yield limit, this feature will disappear.

Phosphatized bolts greased with Molycote 1000
(Also valid for bolts greased with Loctite 243 or oil.)

Thread		Tightening torque in Nm<br>10 Nm ≈ 1 kpm		
M	UNC	8.8	10.9	12.9
4		2.5	3.4	4.2
5		4.9	7.0	8.3
6		8.4	12.0	14.6
7		13.8	19.8	23.2
8		20.6	28.4	34.4
10		40.4	55.9	67.9
12		69.7	98	117
	1/2"	80.8	114	136
14		110	156	187
	5/8"	159	224	269
16		169	238	286
	3/4"	280	393	471
18		237	332	398
20		331	465	558
22		445	626	752
24		572	804	963
	1"	670	942	1131
27		826	1161	1393
30		1127	1582	1901
	1 1/4"	1308	1840	2208
33		1522	2133	2563
36		1961	2761	3311
39		2520	3543	4248
42		3130	4395	5280

Untreated bolts greased with Molycote 1000
(Also valid for bolts greased with Loctite 243 or oil.)

Thread		Tightening torque in Nm<br>10 Nm ≈ 1 kpm		
M	UNC	8.8	10.9	12.9
4		2.9	4.0	4.9
5		5.7	8.1	9.7
6		9.8	14.0	17.0
7		16.1	23.0	27.0
8		24.0	33.0	40.0
10		47.0	65.0	79.0
12		81.0	114	136
	1/2"	94.0	132	158
14		128	181	217
	5/8"	185	260	312
16		197	277	333
	3/4"	325	457	548
18		275	386	463
20		385	541	649
22		518	728	874
24		665	935	1120
	1"	779	1095	1315
27		961	1350	1620
30		1310	1840	2210
	1 1/4"	1521	2140	2567
33		1770	2480	2980
36		2280	3210	3850
39		2930	4120	4940
42		3640	5110	6140

As a rule, always change screws with specified tightening torque on cranes in operation if the joint has to be opened for some reasons.

If any joint has been opened apply Intertuf after tightening the joint to avoid rust damage.

SAE flange for high pressure hose couplings

Flange 6000 psi	Screw	Tightening torque in Nm 10 Nm ≈ 1 kpm Class H 10.9 and 12.9
3/4"	M10	54
1"	M12	94
1 1/4"	M14	150
1 1/2"	M16	230
2"	UNC 3/4	377

Stainless bolts and hydraulic pipe couplings greased with Gleitmo paste

Thread d	Tightening torque in Nm 10 Nm ≈ 1 kpm Austenite (A)		
	50	70	80
6	3.3	7	9.3
8	7.8	17	22
10	15	33	44
12	27	57	76
14	43	91	121
16	65	140	187
18	91	195	260
20	127	273	364
22	171	367	490
24	220	472	629
27	318	682	909
30	434	930	1240
33	585	1250	1670
36	755	1620	2160
39	969	2080	2770

Strength of screws and bolts

Strength class	Min. ultimate strength N/mm²	(kp/mm²)	Min. tensile strength N/mm²	(kp/mm²)	Corresponding strength class of nuts
4.6	400	(40)	240	(24)	4
5.8	500	(50)	400	(40)	5
6.6	600	(60)	360	(36)	6
8.8	800	(80)	640	(64)	8
10.9	1000	(100)	900	(90)	10
12.9	1200	(120)	1080	(108)	12

Examples of markings:

Hexagonal screw

Strength class 4.6:

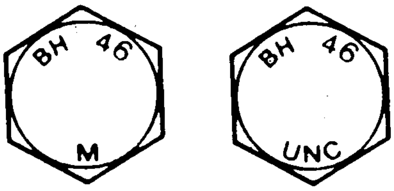

Strength class 8.8:

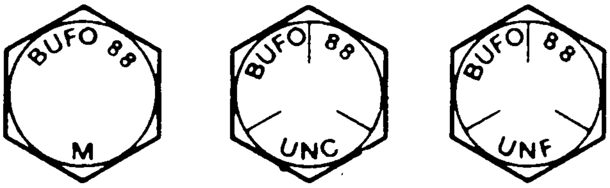

Strength class 10.9:

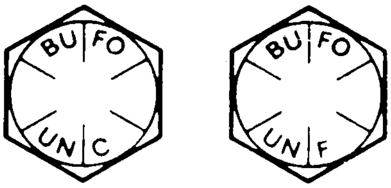

Allen screw

Strength class 12.9:

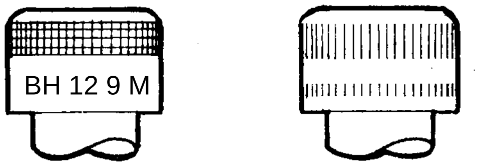

Hexagonal nut

Strength class 8:

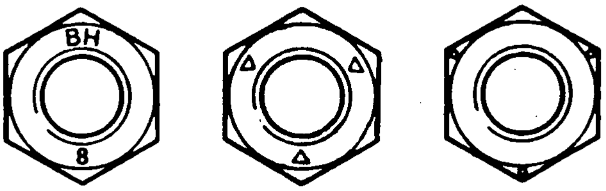

M-thread

UNC- and UNF-thread Cold forged

UNC- and UNF-thread Forged

Strength class 10:

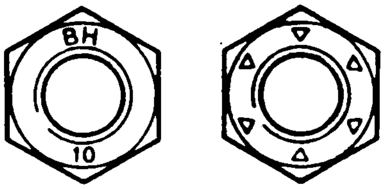

M-thread

UNC- and UNF-thread Forged

Tightening Torques
Hydraulic Couplings

The values below presume greasing of threads when assembling.

Type of coupling	Thread M and R	Tightening torque in Nm
GE 10-LM	M 14x1,5	ca 39
GE 10-LR	R 1/4	ca 39
GE 12-LM	M 16x1,5	ca 59
GE 12-LR	R 3/8	ca 59
GE 15-LM	M 18x1,5	ca 69
GE 15-LR	R 1/2	ca 108
GE 18-LM	M 22x1,5	ca 108
GE 18-LR	R 1/2	ca 108
GE 22-LM	M 26x1,5	ca 128
GE 22-LR	R 3/4	ca 157
GE 28-LM	M 33x2	ca 216
GE 28-LR	R 1	ca 265
GE 35-LM	R 42x2	ca 353
GE 35-LR	R 1 1/4	ca 392
GE 42-LM	M 48x2	ca 491
GE 42-LR	R 1 1/2	ca 491

Table 1.

Type of coupling	Thread M and R	Tiightening torque in Nm
GE 10-SM	M 16x1,5	ca 59
GE 10-SR	R 3/8	ca 69
GE 12-SM	M 18x1,5	ca 78
GE 12-SR	R 3/8	ca 69
GE 14-SM	M 20x1,5	ca 108
GE 14-SR	R 1/2	ca 128
GE 16-SM	M 22x1,5	ca 128
GE 16-SR	R 1/2	ca 128
GE 20-SM	M 27x2	ca 196
GE 20-SR	R 3/4	ca 206
GE 25-SM	M 33x2	ca 294
GE 25-SR	R 1	ca 314
GE 30-SM	M 42x2	ca 491
GE 30-SR	R 1 1/4	ca 491

Table 2.

Example: GE 10 - LM

d = 10

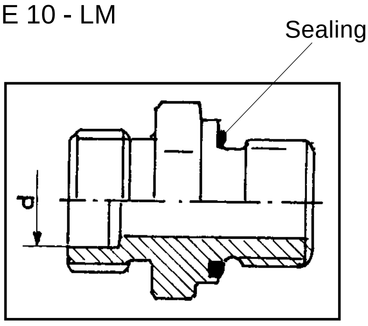

Fitting

Sealing cone

Insert the sealing cone into the 24-degree coupling fitting, firmly tighten the coupling by hand. Using a wrench, tighten the coupling nut another 1/4 to 1/2 turn . The O-ring should preferably be oiled.

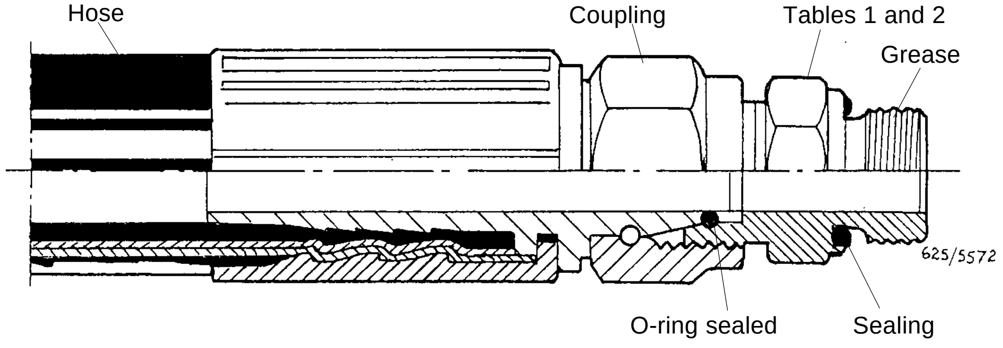

Rope Reeving Diagram for Cranes Type GL-2 (new version)

Luffing **Hoisting**

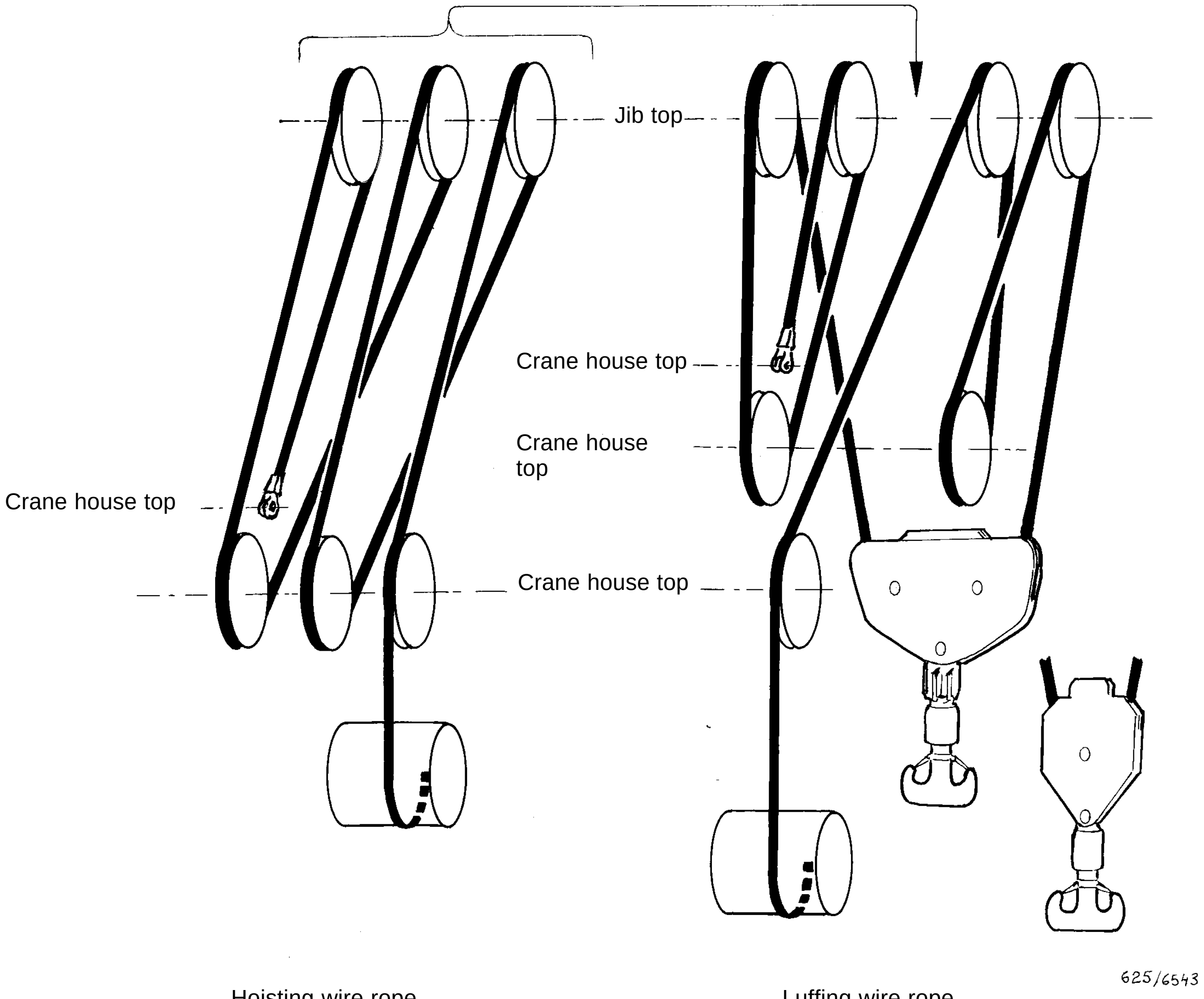

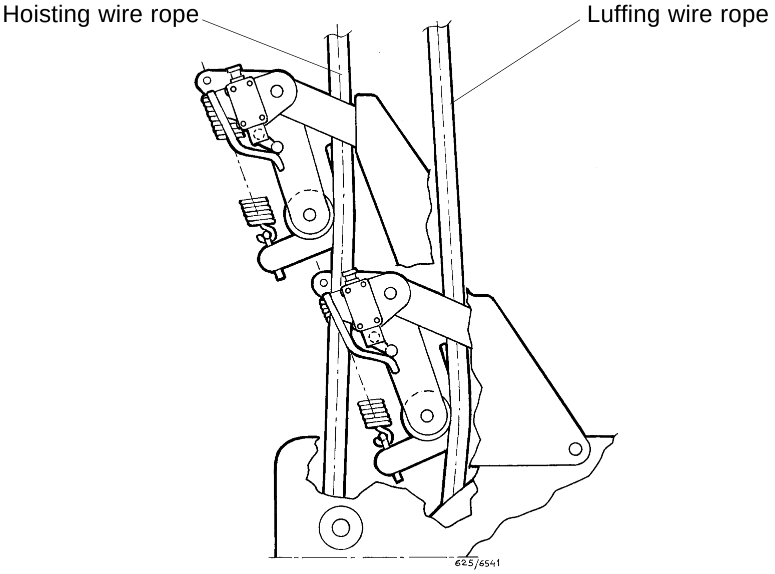

Slack wire safety switch, hoisting and luffing. Slack wire condition

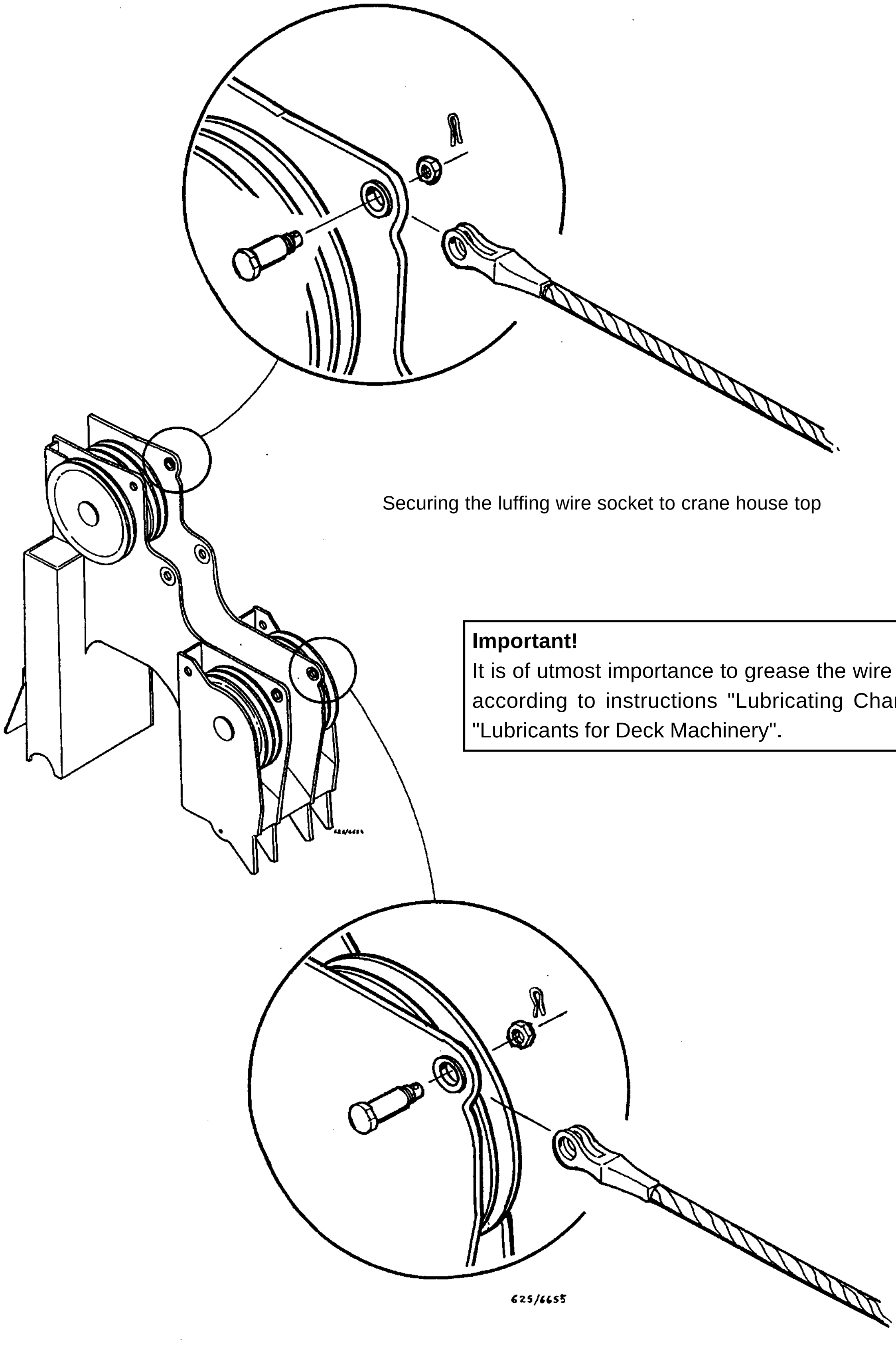

Securing the luffing wire socket to crane house top

Important!
It is of utmost importance to grease the wire socket according to instructions "Lubricating Chart" and "Lubricants for Deck Machinery".

Securing the hoisting wire socket to crane house top

Note! The wire rope socket should be safely secured.

Handling, Installation and Maintenance of Steel Wire Ropes

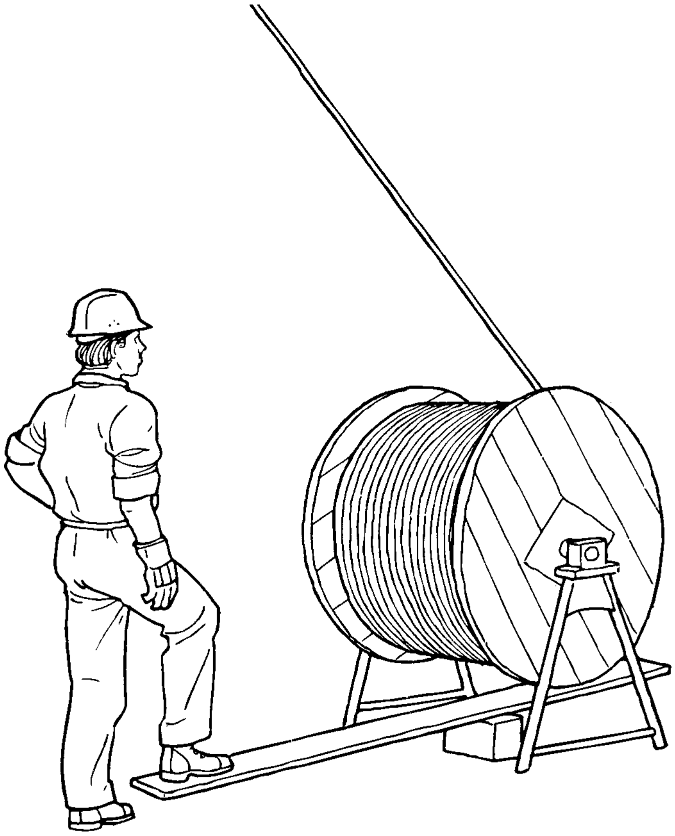

Unloading steel wire ropes

When handling a steel wire rope, the first trouble often occurs immediately upon receiving it: the fork of the fork lift truck is either placed under the reel or inside the coil.

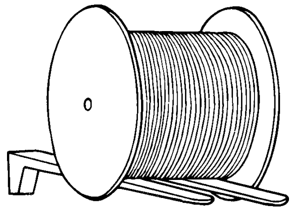

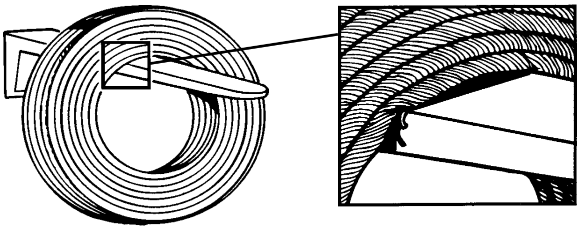

In both cases it might damage the surface of the rope. The damage may not be discovered until much later and it could happen that the manufacturer of the wire rope is held responsible.

If possible, the rope, when recieved on cils or reels, should not have any contact with a metal hook or the fork of a fork lift truck.

Instead, it should be lifted by means of a wide textile webbing sling.

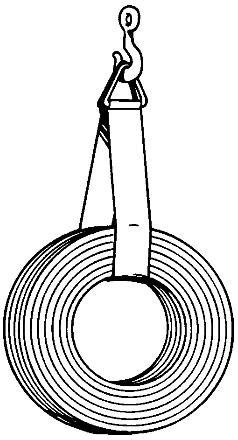

It is advisable to lift a reel by means of a shaft which is put through its axis bore.

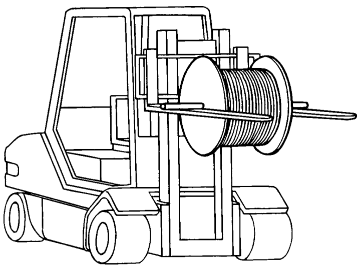

If the fork of the fork lift truck is longer than the width of the reel, the reel can also be lifted at the flanges.

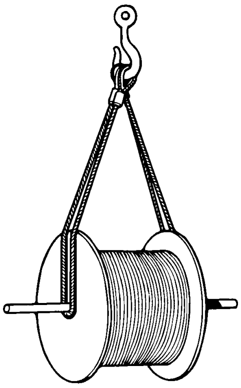

Storing steel wire ropes

Steel wire ropes should be stored in a clean, cool, dry place indoors. The ropes must not be allowed to rest on the floor. They can be placed on pallets.

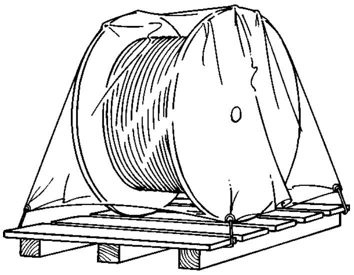

If outdoor storage cannot be avoided the ropes must be covered in a way that moisture cannot create corrosion problems. Although plastic foil protects the ropes from rain, condensation from beneath might not be able to escape and could damage the ropes permanently. To avoid condensation problems, it is advisable to use breathable water-proof fabric covers readily available from tarpaulin manufactures.

When storing a number of spare ropes, the following rule should be applied: first in - first out. This means, the ropes should be used in the order of delivery. In this way it can be avoided that certain ropes are only put in service after being stored for many years.

It is self-evident that the different ropes in stock must be clearly marked to avoid the possibility of confusion (e.g. if similar ropes of diffferent tensile strength are stored).

In addition proper records have to be kept which make it possible to trace the "history" of any rope back to the manufacturer on the basis of storing number, specification, date of order and date of delivery.

Installing steel wire ropes

When installing steel wire ropes, extra care must be taken that the ropes are unwound from the ring or reel without torsions and without any outer damage. The same applies to reeving the ropes into the system.

Unwinding steel wire ropes from the coil

If a rope is delivered on a coil, it is either unwound on a turntable or the coil is rolled along the ground like a hoop. In the latter case ensure that the surface is clean; sand or grit that sticks to the lubricant might damage the wires when the rope travels or sheaves.

Unwinding steel wire ropes from the reel

An unreeling stand (turnable) should be used to unwind a wire rope from its reel.

Another accepted unreeling method is to mount the reel on a shaft supported by two jacks or a stand.

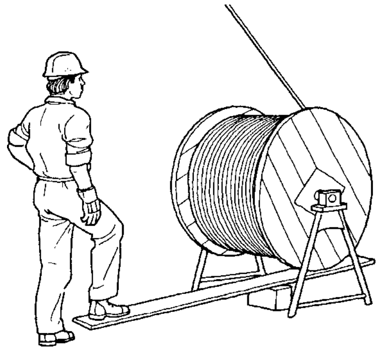

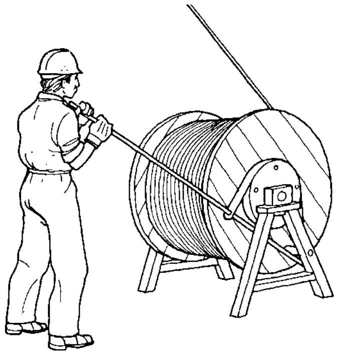

Rolling the wire rope along the floor, as is sometimes recommended in the relevant literature, does not work very well in practice because the reel always unwinds less wire than the distance the reel travels, so that with this method the rope has to be dragged along the worker.

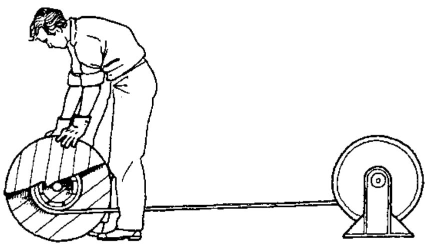

Under no circumstances must the rope be pulled off a coil while it is lying on the ground or looped over the head of the reel, because this will inevitably induce one torsion per wrap into the rope.

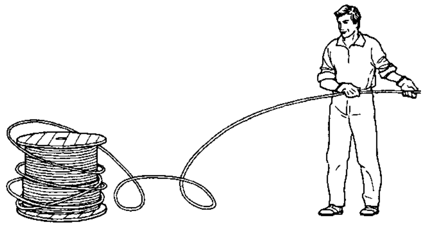

Every torsion will change the lay lengths of the stands and of the wire rope; at the same time the proportions of lengths of the rope elements and finally the distribution of load within the rope are changed. A rope that is unwound at the sides of a coil or reel will try to resist the enforced torsions and form loops. When pulled taut these loops will result in irreparable kinks.

Steel wire ropes with kinks are not safe to operate and must be discarded.

The installation procedure

The most advantageous way of installing a steel wire rope varies from crane to crane. In any case a procedure should be chosen that (under justifiable expenditure) guarentees the least risk of torsions and avoids damage to the rope by contact with parts of the construction.

With some cranes it may be advisable to discard the old rope first and to install the new one afterwards. With other cranes, particularly with bigger ones, it might be better to pull in the new rope attached to the old one. Another possibility is to use a thinner rope by which the wire rope proper is pulled into the system. This method is often used with new equipment.

In every single case careful consideration is necessary as to whether the wire rope should be pulled through the whole reeving system or whether it should first be wound from the coil or reel onto the drum and afterwards be reeved into the system.

If one end of the ropes has a fitting attached, as it is mostly the case with deck cranes, there is no other possibility than pulling the loose rope end through the whole reeving system.

The most common rope fittings for deck cranes are shown below.

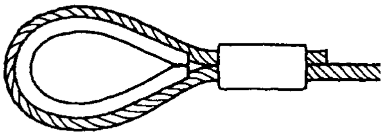

The aluminum ferrule with mechanical splice.

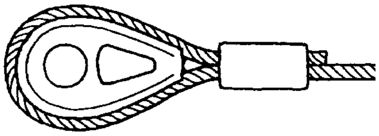

The cast steel ferrule with mechanical splice.

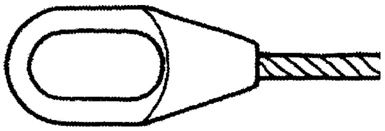

The spelter socket.

Typical deck crane example

Below is shown an example of a typical deck crane, where the rope must be spooled from the reel via sheaves S1 through S9 onto the drum.

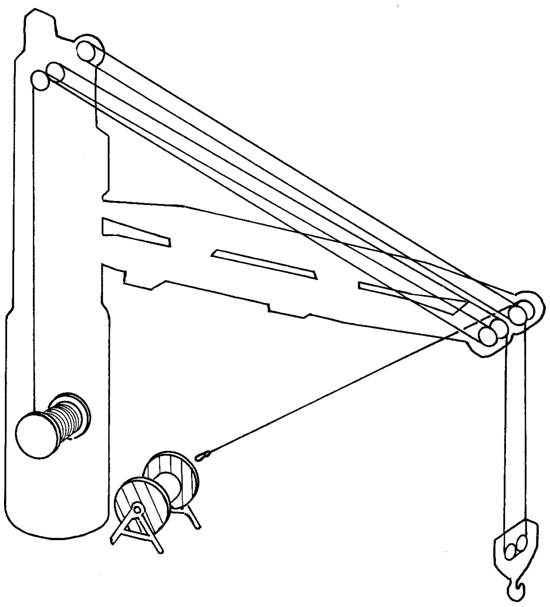

Here, special attention must be paid to the hook block. It must be secured in an upright position so that the rope can be spooled without any fleet angles occuring between the rope and the block´s sheaves.

Winding the steel wire rope from the reel onto the drum

During the manufacturing process every steel wire rope receives its preferred bending direction when being drawn from the wire rope closer by means of a capstan. When delivered to the customer the rope is bent in that direction. Make certain that it bends in the same direction when it is wound from the reel onto the drum.

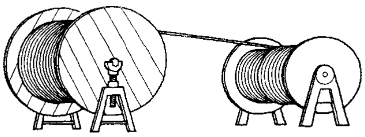

If the rope is wound at the bottom of the drum, it should leave the reel at the buttom and vice versa: i.e., always reel from top to top or from bottom to bottom.

If this procedure is not strictly followed, the rope will either try to twist between reel and drum or it will later try to regain its preferred position when in practical service. In both cases structural changes of the rope may occur.

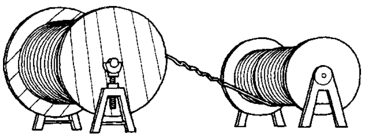

Installing the rope with the help of the old one or by a thinner rope

If the new rope is pulled in by the old one or by a thinner rope one must make sure that the connection between these ropes is absolutely safe. In addition it must be ensured that the thinner rope cannot rotate. Rotation-resistant steel wire ropes or three-strand ibre ropes for instance, can be recommended for this purpose. When using conventional wire ropes one must at least make sure that they have the same direction of lay as rope to be installed.

If the new rope is pulled in with help of the used one, the two rope ends are often buttwelded together. A connection of that kind can transfer the twist of the old rope, built up in the reeving system, into the new rope. By that method of installation the new rope may be extremely damaged.

There are even more reasons why that procedure is highly problematic: It is true, that when using special electrodes the welded connection presents acceptable results in a pull test with a straight rope; but because of the great length of rigid connection zone the very same connection could break due to the enormous bending stresses when running over sheaves.

If that connection is applied, its safety should be increased by using a Chinese finger. Fewer problems are caused by connecting wire ropes with welded-on pad eyes or chain links, which are joined by either strands or thin wire ropes.

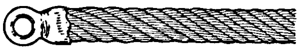

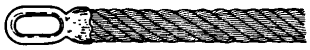

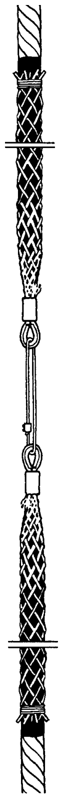

This connection provides satisfactory load capacity, it is flexible and prevents the transfer of twist from the old rope into the new one. When using two strands to pull the rope into place, these will indicate the intensity of twist in the old rope on the basis of the number of turns they have made during the installation procedure.

Another possibility is connecting the rope ends with Chinese fingers. These are tubes made out of braided strands, which are pulled over the rope ends and then secured at their ends with tape or seizing wire. Under load the Chinese fingers will contract and hold the rope ends by friction.

When installing a lang lay rope one must take care that the Chinese finger cannot unwind from the rope like the nut from a screw. It is recommended to wrap a tape around the whole rope length to be held by the Chinese finger to increase the friction.

Installing under load

To achieve perfect spooling of the rope on the drum it is very important to apply a tensioning load to the wire ropes during the installation.

This is particularly important with drums spooling in two or more layers, which, however, is not very common with deck cranes. If the first layer(s) are not under tension, they might be too loose, so that the top layers might be wedged into the bottom layers under load. This could seriously damage the rope. The unwinding rope might even be clamped, so that the direction of spooling could suddenly be reversed during the course of unwinding. The result could be the abrupt lifting of the load that was actually travelling downwards.

The tensioning load should range from 1% to 2% of the minimum breaking load of the wire ropes. In many cases it might suffice to wind the rope quite normally in order to unwind it and then rewind it with the help of an outer load.

In other cases, however, the procedure mentioned above is not possible. In these cases the tensioning load must already be applied when installing the rope.

Ample rope tension can be provided by a simple plank bearing against the reel flanges or by a braking disk attached to the reel.

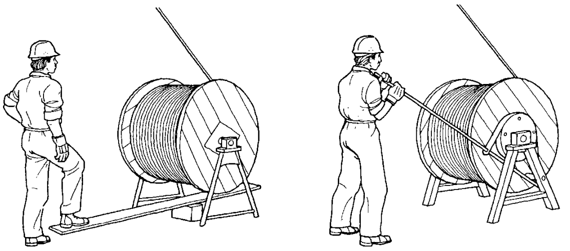

Under no circumstances should one attempt to generate the tensioning load by jamming the rope, for instance between two boards. Structural changes would deform the rope beyond repair.

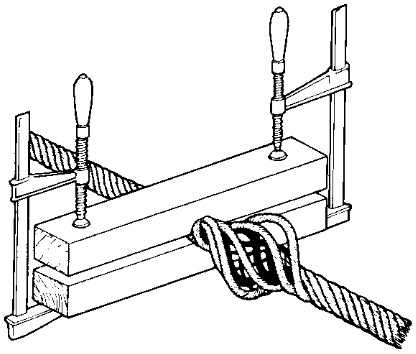

Attaching the rope termination to the fix point

After the rope has been pulled through the reeving system, the rope termination (e.g. the mechanical splice with the aluminum ferrule) must be brought up to the fix point. A pulling jack can be used to pull the rope termination to the fix point, where it must secured with a bolt.

Prior to fixing, if necessary, the rope termination may be aligned with the fix point by using a steel bar. The bar should be attached to the rope by means of a short length of chain. Under no circumstances should the rope be gripped with a wrench, as this will damage the outer wires.

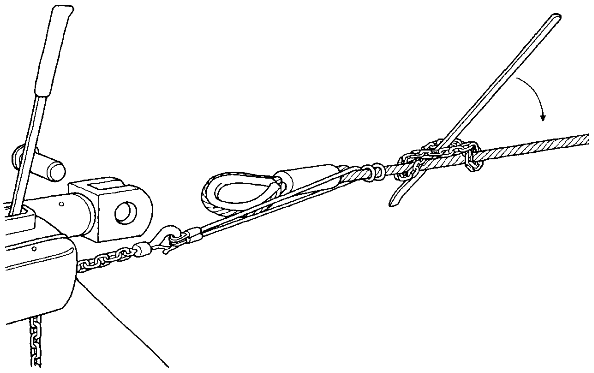

"Breaking-in" the steel wire rope

After the rope has been installed and before it is going to do its proper job, several run-throughs of the normal operational circle should be carried out under light load. The new rope should be "broken-in", so that the component parts can settle and adjust themselves to the actual operating conditions. It is most unfortunate that in practice only too often the exact opposite of this recommendation is performed: quite frequently after installing the rope overload tests are carried out with loads beyond the safe working load of the system.

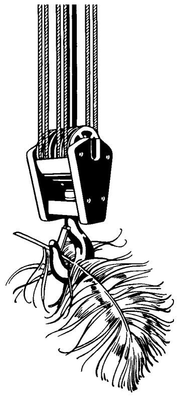

Cutting steel wire ropes

In some cases the user must cut steel wire ropes. Hand cutters are sufficient for rope diameters up to 8 mm. Mechanical or hydaulic cutters will be required for larger sizes.

The best method is to use a high speed disk cutter. Unless the rope is being scrapped the use of flame cutting equipment is not recommended.

Careless cutting can result in the balance of tension in the rope being destroyed. This is particularly important when cutting rotation resistant ropes where the strands may have been deliberately nonpreformed as part of the manufacturing specification.

In every case, each side of the cut must be properly seized to prevent strand disturbance. Insulating tape cannot prevent strand movement, so annealed (iron) wire should always be used.

After marking the position of the cut the end of the seizing wire is laid along the rope axis leaving sufficient length to secure both ends by twisting when the seizing complete.

The rope and this wire end are now wrapped moving away from the location of the intended cut.

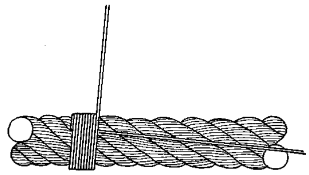

The rope is tightly wrapped for a distance of approx. three rope diameters.

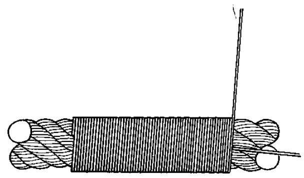

Both ends of the seizing wire are then pulled tight and twisted together for a length of one rope diameter.

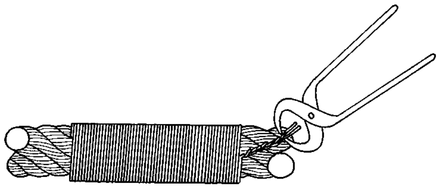

The twisted connection is then hammered into a gusset between the strands. After preparing the other side of the intended cut accordingly the rope can now be cut.

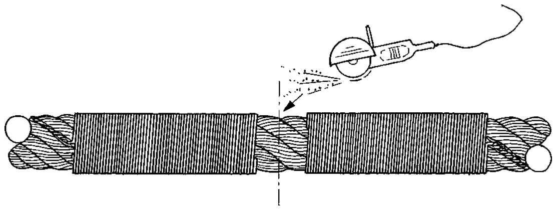

Instead of using one long seizing it is also possible to apply at least three seizing the size of one rope diameter each on both sides of the intended cut.

The maintenance of steel wire ropes

Steel wire ropes must be serviced regularly, the kind of maintenance depending on the lifting device, its use and the selected rope. Regular maintenance may considerably increase the service life of a steel wire rope.

Relubricating steel wire ropes

During production the rope receives intensive lubrication. This in-process treatment will provide the rope with ample protection against corrosion and is meant to reduce the friction between the elements which make up the rope as well as the friction between rope and sheaves or drums. This lubrication, however, only lasts for a limited time and should be re-applied periodically.

German Standard DIN 15 020, e. g. specifies: "Steel wire ropes must be relubricated at regular intervals, depending on their use, particularly along the zones subjected to bending. If for operational reasons relubrication cannot be carried out, shorter service life of the rope is to be expected and the inspection intervals have to be arranged accordingly."

When choosing the relubricant, it must be ensured that it is in accordance with the recommendations of the rope manufacturer.

There are several techniques of lubricant application: The most common ones at present are painting or swabbing.

Quite often the lubricant is applied at a sheave, sometimes a continuous drip method is used. If only a little lubricant is required, pressure spray nozzles can be applied.

Maximum penetration of the lubricant into the gaps of the rope, can only be quaranteed if high pressure lubrication is applied with the help of a pressure lubricator.

With this method the two halfs of a sleeve, which is equipped with rubber sealings, are clamped round the rope and screwed together. While the rope runs through the lubricator the lubricant is pressed into the sleeve at a pressure upto about 30 bars.

It is important with all different methods of relubrication of steel wire ropes that they are carried out regularly right from the beginning of the service life of the rope and not only after the first damage has been ascertained.

Cleaning steel wire ropes

DIN 15 020 recommends: "From time to time very dirty steel wire ropes should be cleaned externally".

This applies particularly to ropes operating in extremely abrasive conditions and to those that take up chemicals.

Effective cleaning without proper tools is quite a laborious job. For cleaning steel wire ropes the Canadian Rigging Manual recommends an appliance with three rotating wire brushes and an air blast drying system to follow. An American manufacturer offers a "rope porcupine", a sleeve equipped with brushes, which is drawn along the steel wire rope.

Removing broken wires

If during an inspection ends of broken wires are detected which might cross adjacent wires and destroy them when running over sheaves, these broken wire ends must be removed.

Under no circumstances should the broken wire ends be pinched off with a pair of nippers. The best method is to move the wire ends backwards and forwards until they break deep in the valley between two outer strands. With thicker wires a tool should be moved backwards and forwards on the surface of the rope, thus bending the wires until they break.

Cutting or shifting steel wire ropes

Very often wire ropes must be discarded although only short rope sections, e. g. the one that climbs to the second layer on the drum, are seriously damaged, while the rest of the rope is still in perfect condition.

In cases such as this the service life of wire ropes can be enormously increased by shortening, shifting them at the fixing point by a span that removes the section of the rope which has had most abuse out of the critical zone.

After this procedure an adjacent section will be subjected to the abuse.

Another typical local damage occurs on the drum at those sections where the rope rubs against the adjacent winding (crossover point) and must be deflected to the side. If the damage caused in these sections is the main reason for discarding the rope, several cuttings or shiftings will move the stresses to different rope zones and possibly multiply the service life of the rope.

Examples of damage to steel wire ropes

The following pages illustrate some typical examples of damage to steel wire ropes which should always decree immediate rejection of the rope.

Surfacelayer wires frayed by abrasion; wire ropes has been running over sharp edges under load.

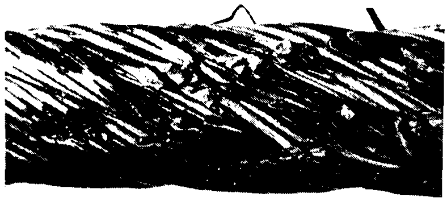

Localized wear caused by chafing against adjacent steel structures possibly because of vibrations of the length of wire rope laying between drum and sheave.

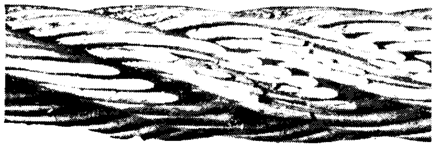

Heavy wear caused by high contact pressure in sheave groove.

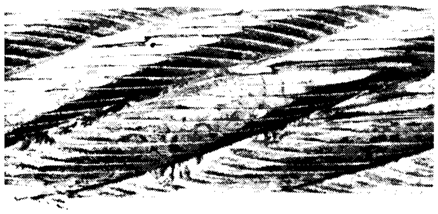

Severe corrosion.

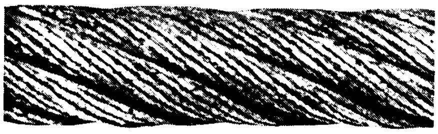

Typical wire rupture caused by bending fatigue.

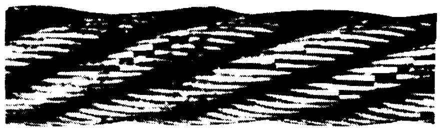

Typical example of localized wear and indentation caused by a kink in the rope.

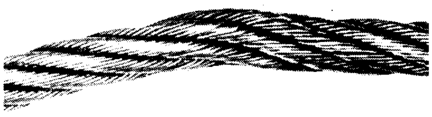

Nonrotating multi-strand wire rope with outer strand partially disengaged because of nonuniform torsional stress.

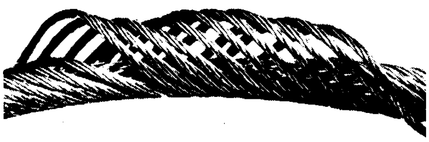

High shock loads have caused outer layer to split open exposing steel wire rope core between strands that have come apart.

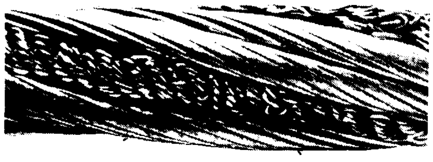

Some examples of wire rupture

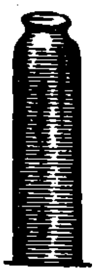

Contraction rupture caused by excessive stress, (e.g., if a slack line suddenly snaps taut under full load).

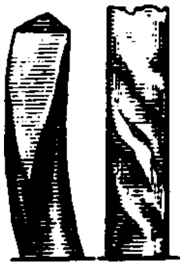

Wear and identations of surface wires also promote fatigue and lead to premature wire rupture.

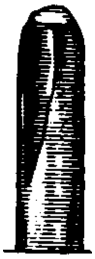

Crown wires of outer strands may wear so thin that the residual cross sectional area of steel in the rope will not support the normal design load and contraction rupture may ensue.

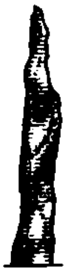

Corrosion may cause wire ropes to break. As a safety measure, inspect ropes regularly and carefully to prevent catastrophes; observe manufacturer's instructions

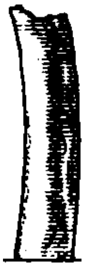

Rupture from fatigue will often occur when undersize sheaves are used. Vibrations and torsional stress loads in the rope accelerate fatigue rupture.

Replacement of Wires
One or Two Fall Rigging

General

This instruction covers almost all MacGREGOR cranes, which are equipped with limit switch boxes containing electric limit switches. The procedure is general and should be seen as a guidance only.

Hoisting winch / wire

For cranes with one or two fall rigging proceed as follows:

- Place the jib in the jib rest or park the crane (if applicable), slewing position locked with cylinders and the jib at max. outreach parked on top of the hoisting block.
  See special instruction "Parking of crane in wires".
- Disengage the slack wire switch by blocking the spring tensioned roller, which can be done with a piece of wood, see Fig. 1.
- Loosen the wire rope socket attached to the crane house top, see Fig. 5 .
- Run the winch by paying out the hoist wire, and at the same time pulling on the loose wire end.
- Stop the winch just before it enters into the empty drum limit.
- Mark the position of the wire drum in relation to the bracket, this to avoid setting of all hoisting limits again after installing the new hoist wire.
- Loosen the bolt that holds the limit switch box and fold out the box so much so that the coupling/gear wheel, between the limit switch box and the wire drum, divides, see Fig. 2.
  NB. - Mark the position of the coupling/gear wheel halves in relation to each other before dividing.
- Note how many revolutions of the wire there are left on the drum at this position.
- Secure the wire so the wire clamps that hold the end of the wire can be removed safely.
- Attach a long rope (longer than the wire rope) to the wire end, and place it 4 to 5 turns around the wire drum.
- Run the winch by paying out the old (damaged) wire until it is on the deck.
- Attach the new hoist wire to the same rope by using tool 662 0754-000, see Fig. 4.
- Run the winch by pulling in the new hoist wire, so that there are the same numbers of revolutions of wire on the wire drum as noted earlier and that the winch corresponds to the position marked earlier .
- Attach the wire rope end to the wire drum with the wire rope clamps and tighten the bolts.
- Assemble the limit switch box in its proper place, the marking on the couplings must correspond.
- Run in the rest of the wire. **NB.** The wire socket should be safely secured, see Fig. 5.
- Remove the wooden piece holding the slack wire roller.
- Test the winch and check all limits, which should now function correctly, if the new wire is the same length as the old one. If necessary, adjust the limits, see instruction under section 6.3.

Luffing winch / wire

- Place the jib in the jib rest, if applicable or park the jib on top of the hoisting block. See instruction "Parking of crane in wires".
- Disengage the slack wire switch by blocking the spring tensioned roller, which can be done with a piece of wood, see Fig. 1.
- Loosen the wire rope socket attached to the cranehouse top / jib, see Fig. 5 and 6.
- Lower the luffing wire rope to the deck by means of a rope.
- Mark the position of the wire drum in relation to the bracket.
- Loosen the bolt that holds the limit switch box/gear box and fold out the box/gear box so much so that the coupling/gear wheel, between the limit switch box/gear box and the wire drum, divides, Fig. 3.
  NB. - Mark the position of the coupling/gear wheel halves in relation to each other before dividing.
- Note how many revolutions of the wire there are left on the drum at this position.

- Secure the wire so the wire clamps that hold the end of the wire can be removed safely.
- Attach a long rope to the wire end (longer than the wire rope) and place it 4 to 5 turns around the wire drum.
- Run the winch by paying out the old,damaged luffing wire, and at the same time pulling on the loose wire end until it is on the deck.
- Attach the new luffing wire to the rope, by using tool 662 0754-000.
- Run the winch by pulling in the wire, so that there are the same numbers of revolutions of wire on the wire drum as noted earlier and that the winch corresponds to the position marked earlier as it was when the limit switch box/gear box was disconnected.
- Attach the wire rope end to the wire drum by the wire rope clamps and tighten the bolts.
- Assemble the limit switch box/gear box in its proper place.
- Run in the rest of the wire.
- Attach the wire rope end to the cranehouse top or jib, see Fig. 5 or 6.
  NB. The wire rope socket should be safely secured to the cranehouse top/jib, see Fig. 5 or 6.
- Disconnect the rope holding the slack wire roller.
- Test the winch and check all limits, which should now function correctly, if the new wire is the same length as the old one. If necessary, adjust the limits, see instruction under section 6.3.

<u>Tightening torque</u>: In the first place look for tightening torque on the winch spare part picture, in the second place see instruction 6.214 E.

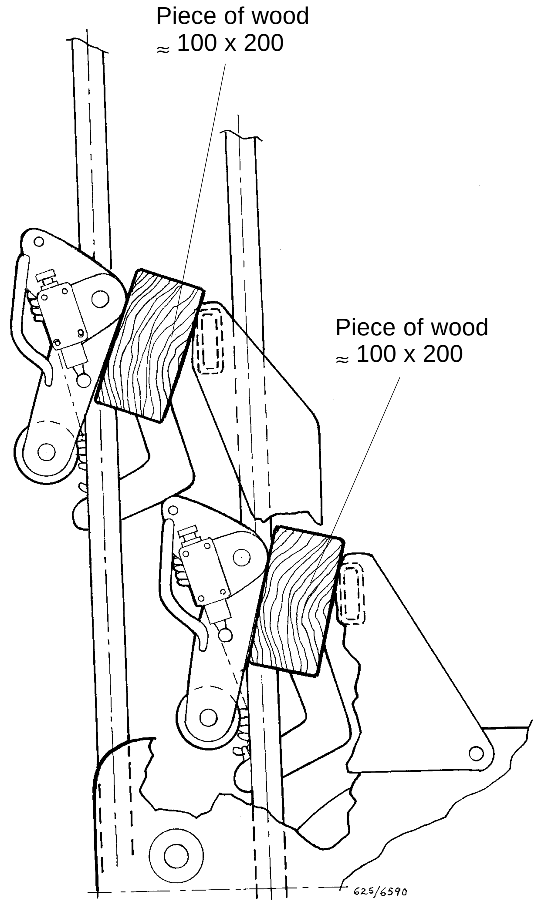

Fig. 1. Slack wire safety switch, hoisting.

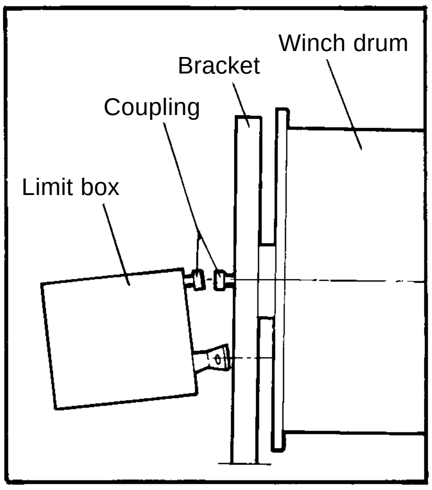

Fig. 2. Limit box, hoisting winch.

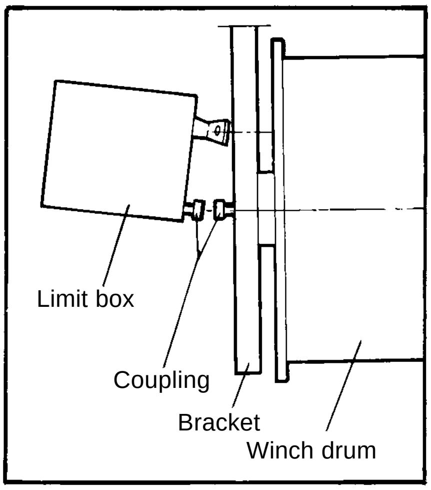

Fig. 3. Limit box, luffing winch.

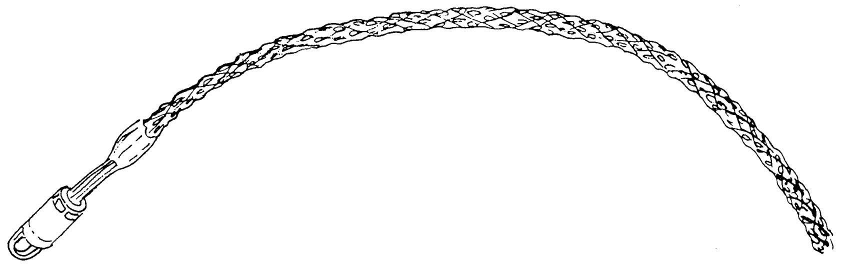

Fig. 4. Tool, wire rigging sock, 662 0754-000.

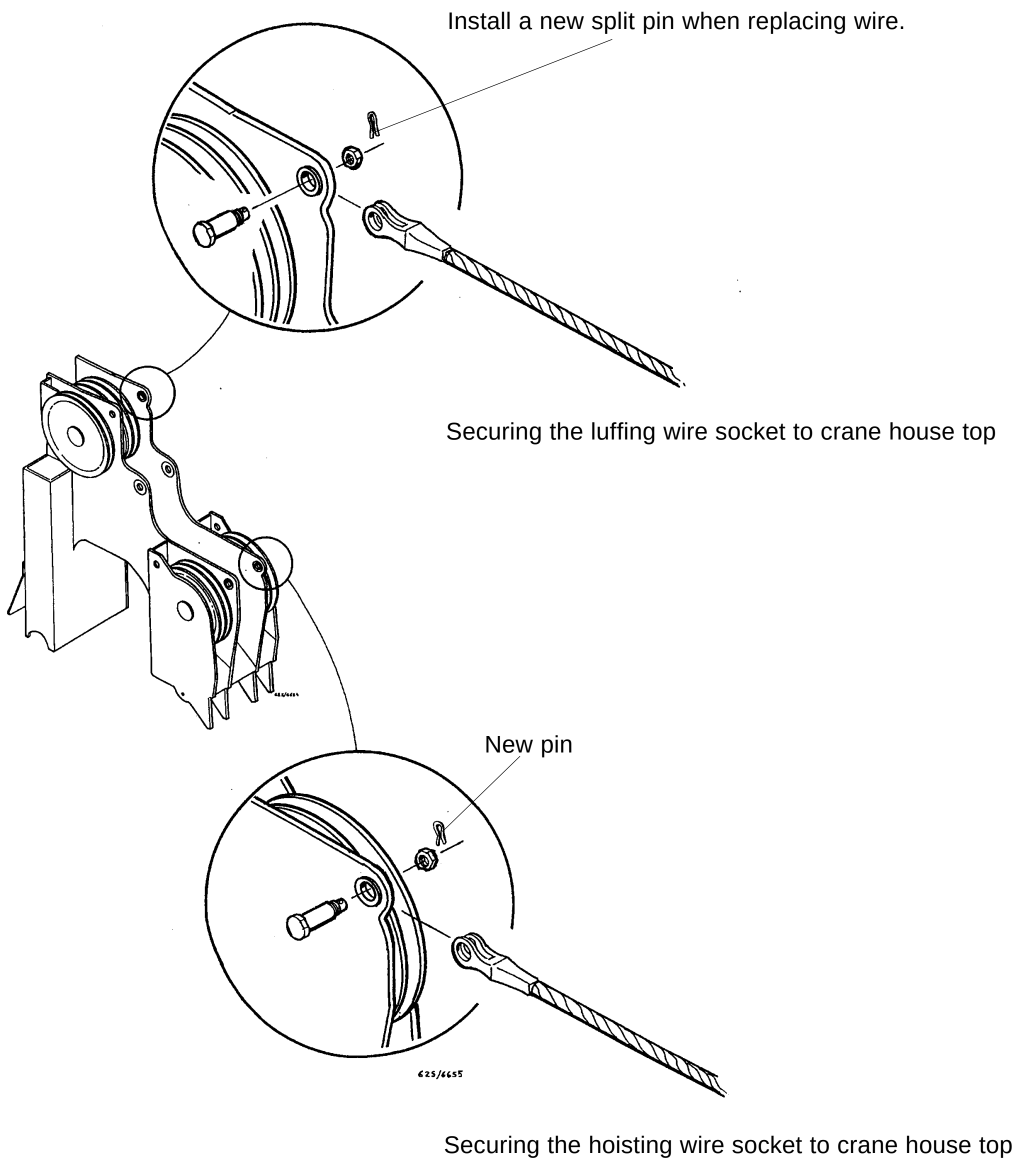

Fig. 5. Securing wire sockets to crane house top.

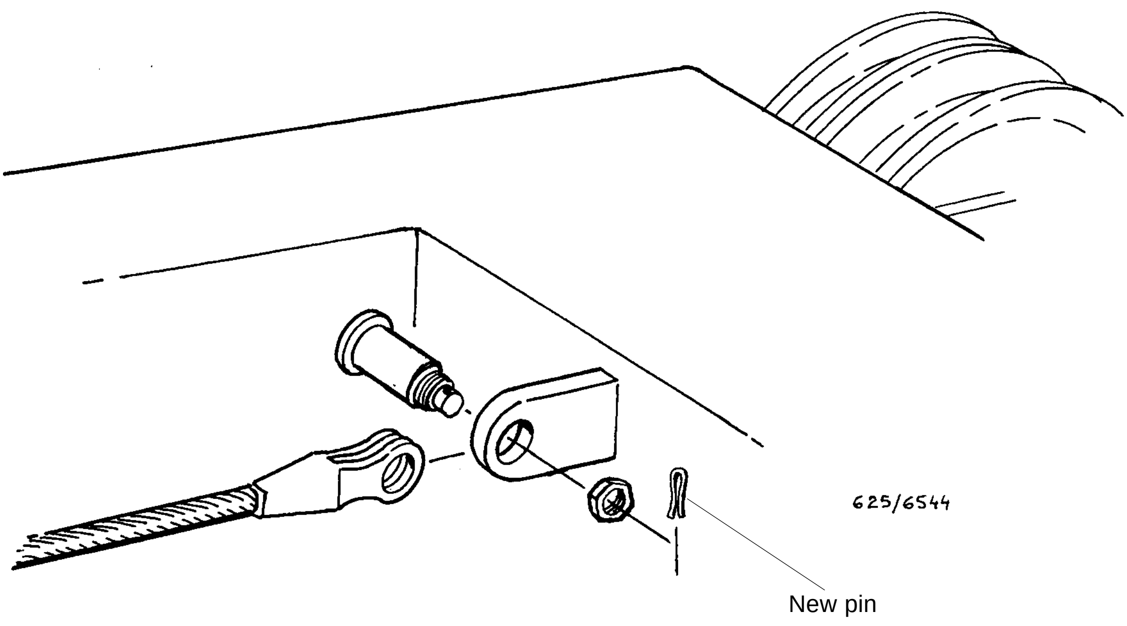

Fig. 6. Securing the luffing wire socket to crane jib.

625/1560 E rev.: b 2003-03-20

Lifting Block

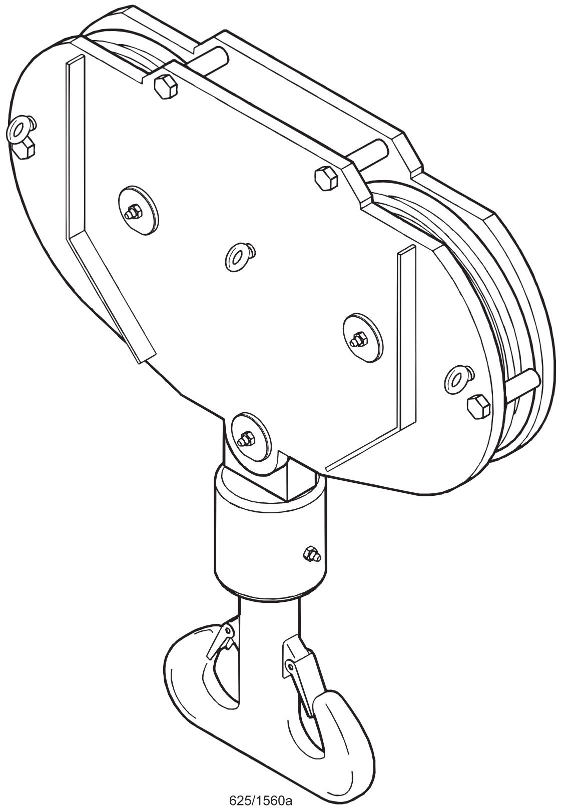

625/1560a

Warning!
Where equipment has swivelling and moving parts there are potential safety hazards.Care should be taken when working with or repairing such equipment. If used incorrectly breakage could occur inflicting injury or death.

General Safety

When equipment is in use, do not put hands:

1. Between sheaves, sideplates and guards.
2. In area of becket, hook, hooknut and crosshead

Take great care to avoid clothing becoming trapped.

Repair and reeving should be carried out by trained personnel only. Power should be switched off before operations are carried out. Work should only take place when equipment is supported on a firm surface.

Maintenance and Service

Inspection should be carried out, on every occasion before taking into operation.

Particular attention should be paid to the following:
1. Wear in hook, centre pin, becket and threads in hook and nut.
2. Play in sheave bushes or bearings.
3. Spacer bolts, nuts and lynch pins.
4. Check for cracks in welds.
5. Condition of safety catch and grease nipples.
6. Wear to holes in sideplates and becket.

If cracks or heavy gouges appear, the equipment should not be used and qualified opinion should be sought. If grooved and the section reduced by more than 5% the item should be replaced.

Repairs should be effected by grinding. **NO** welding should be carried out unless prior authoritly is obtained.
If the holes in the crosshead, sideplates, becket, eyes or yaws are enlarged by more than 5%, the part should be replaced.

All repairs should be carried out by responsible personnel, and great care should be taken in the re-assembly of the equipment and retaining parts, i. e., grub screws, lynch pins, etc. Check and refit only correct sizes and threads.

Important:
When replacing genuine parts an overload test of the complete unit must be performed and new certificate must be issued.

Lubrication

As a general rule sheaves, crossheads and bodies should be oiled through nipples every 100 working hours, see also separate lubrication chart.

Limitation of use

1. Safe working load should never be exceeded.
2. Crane blocks should be used in vertical lift only.
3. Rigging blocks should be used only as in design specifications.
   Blocks should not be used for towing, unless specifically designed and marked for that purpose.
4. Swivels should be used in either the vertical or horizontal plane only.
5. Horizontal and vertical lead sheaves should only be used as indicated in the description.
6. Shock or side loading should not be applied, unless equipment is desigend for that purpose.
7. Load should always be in seat of hook or eye.

Allowed wear on lifting eyes and links

Wear and tear is allowed to take away maximum 5% of the diameter. Thereafter the component has either to be replaced or repaired. The repair recommended is a hardened cage, which shall be shrinked according to the instruction from MacGREGOR Cranes.

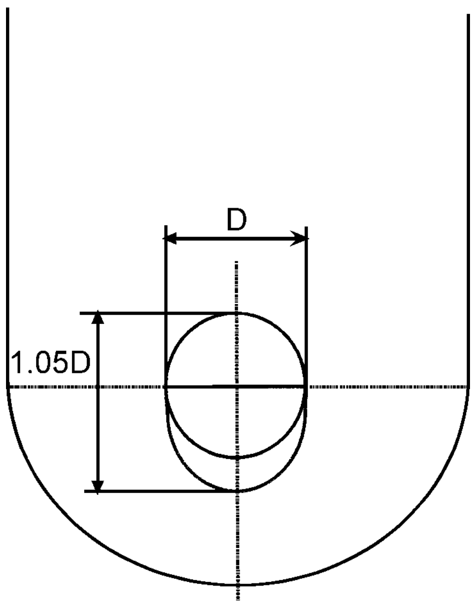

Fig. 1. Lifting eye.

Emergency Operation Card CC2000
314 3615-801

Purpose

In case of MPC-card problems in the crane control system CC2000, cubicle MB, there are always a possibility to replace the MPC-card with an emergency operation card, see Fig. 1.

The emergency operation card is plugged in instead of one (or more) of the movement control MPC-cards for hoisting "Ho", luffing "Lu" and slewing "SL", see Fig. 2.

The emergency operation card can not replace the leftmost MPC-card "Sc", used for internal communication and distribution of control system parameter values.

Low complexity - fixed speed

The emergency operation card is much less complex than the MPC-card. It is built up with relays that activates the outputs for brake, displacement/ unloading and pumps from the control levers 24V signals. The pump output is fixed, giving a constant speed of about 30% of maximum speed. Each motion is controlled by overload, low pressure and limit switches except the limit switch for high hook.

When using the emergency operation card the lamp "Control system error in MB-box" is flashing because the MPC-card "Sc" gives an error message "Communication error" for the movement that the emergency operation card is controlling.

Use of emergency operation card

- Switch main switch OFF
- Remove the Micro Processor Card (MPC) for the faulty motion and plug in the emergency operation card instead.
- Switch the main switch ON.
- Run the desired motion.

WARNING!
The installation of an emergency operation card must be regarded only as a temporary measure to operate the crane in an emergency.

When the emergency operation card has been installed in the hoisting or luffing circuit, bear in mind that the high hook switch is inoperative.

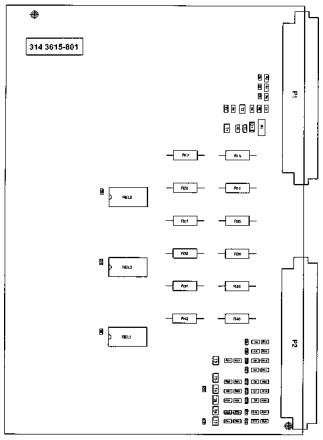

Fig. 1. Emergency operation card.

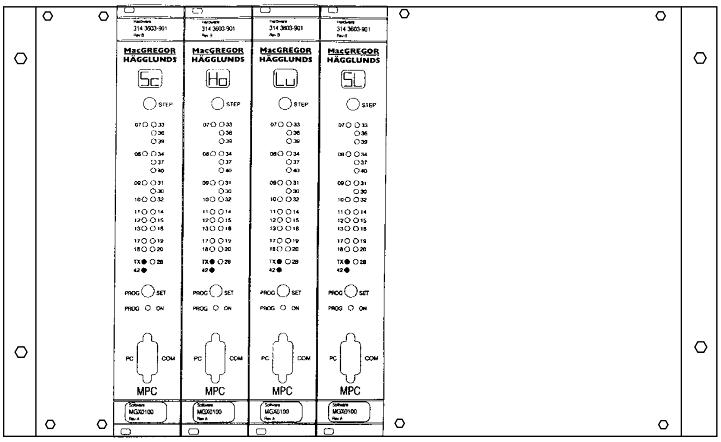

Fig. 2. MPC-cards in cubicle MB.

Crane Control System CC2000

Single Cranes Type GL and LC

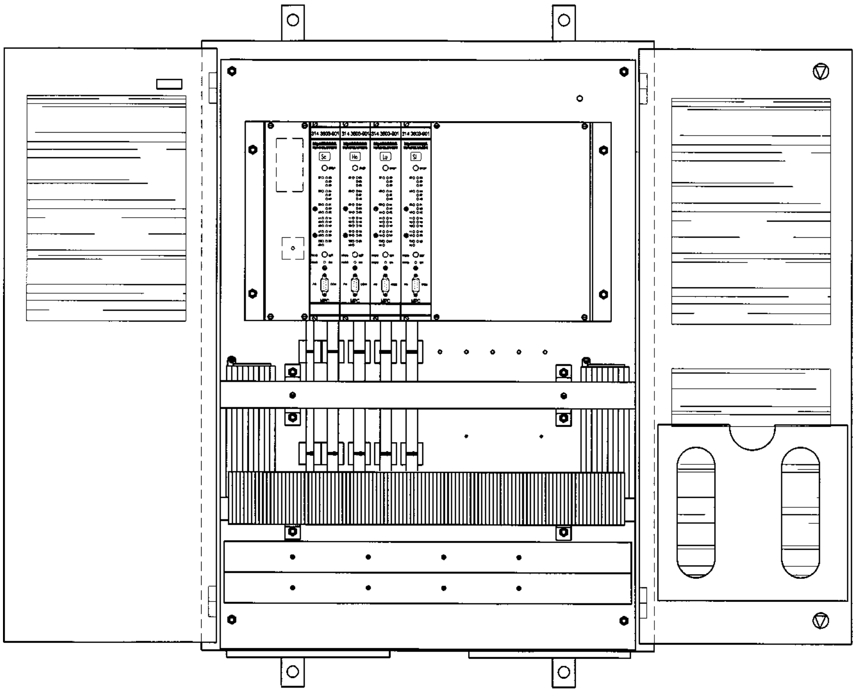

Contents

Description of input and output signals 2
Control system input and output signal levels 5
Signals indicated on the MPC-card front panels 9
Troubleshooting the Control system 12
Operating the crane with less than four MPC-cards 14
Spare parts 15

1. Description of input and output signals

1.1 General

The following description refers to the microcomputer based crane control system CC2000 for MacGREGOR Cranes hydraulic deck cranes, single crane versions.
Minor variations in the use of control system input and output signals due to differences in hydraulic and electric system may occur, the objective of this description is only to provide a general orientation concerning the principles of control system interface signals and of troubleshooting.
For a general description of the principles of the Crane Control system CC2000 see section "Function", Group 3. For detailed information about the control system a complete electric circuit diagram, relating specifically to your crane, will be found in the instruction manual, section "Spare Parts", Group 9.5. Also for detailed information about the hydraulic system a complete hydraulic circuit diagram will be found in section "Spare Parts", Group 9.4.

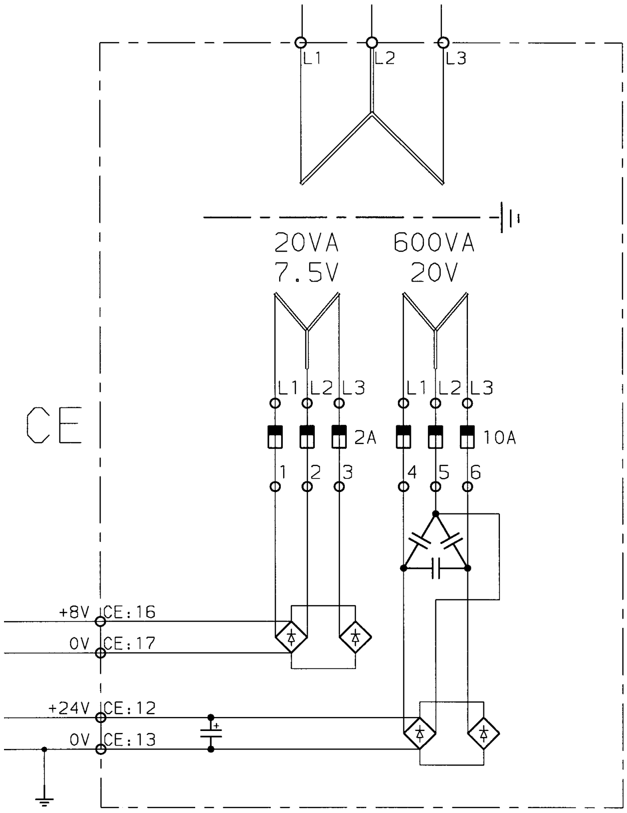

Fig. 1. CC2000 Power supply in cubicle CE.

1.2 Power supply, Control system operation

The power supply in cubicle CE transforms the input 380/400/440V AC to 24 and 8V DC. See fig 1. It consists mainly of a three phase transformer, rectifier bridges and fuses. The output 24V DC is used for the control system input and output signals. The 8V DC is used by each MPC-card to generate 5V DC for its microprocessor.
The output voltage has a ripple with 6 times the frequency of the input power line. The ripple level is approx. 15%.
The 0V level for 24V DC is grounded in cubicle CE and the 0V for 8V is grounded via all four MPC-cards in cubicle MB.

1.3 Power supply, Control system anti condensation heating

The anti condensation heating in cubicle MB is powered with 24V AC when the control system is switched off, when the crane is not operated. A separate transformer is used for all anti condensation heating in the crane. The transformer is fed by a separate power line.

1.4 Control lever (joystick) potentiometers, outputs to and inputs from

The control levers linear potentiometers are fed with 0V, +6V and +12V from their appropriate MPC-card. The potentiometer output is approx. +6V with the lever in neutral. The output signal is controlled by the lever and is depending on the direction of lever movement. The output signal is increased to approx. +10.5V for max. speed hoisting, luffing in and slewing left respectively. The output signal is decreased to approx. +1,5V for max. speed lowering, luffing out and slewing right respectively.

1.5 Control levers (joysticks) direction signal inputs

The control lever for hoisting selects two out of four 24V direction signals, for selection of low speed/ high speed and hoisting/lowering. See fig 2.
The control lever for luffing/slewing gives two out of four 24V direction signals for selecting luffing in/ luffing out and slewing left/slewing right.

1.6 Pressure/force sensor input

If fitted, a sensor giving 4-20 mA is used to weigh the load.

1.7 Pump solenoid outputs

Each hydraulic pump having two solenoid valves, one for each direction of the oil-flow. The solenoids are controlled with a 24V PWM (Pulse Width Modulated) signal with current feed-back control. If wanted output current is not reached (due to short-circuit, open-circuit or MPC-card failure) the system then gives an error message, see sign ERROR MESSAGES.

Each MPC-card is capable of controlling a maximum of three pumps with a total of six output drivers, one for each solenoid valve. Each driver is capable of outputting a maximum current of 1A. To measure the current without opening the circuit a measuring point is available for each pump. Using a Volt meter, the reading in mV equals the current in mA within approx. 5%.

1.8 Brake release/direction valve outputs

Each MPC-card having an output driver for the brake release signal (or direction valve for cr cranes equipped with cylinder luffing). The system is capable of detecting short-circuited output and then giving an error-message, se sign ERROR MESSAGES.

1.9 Displacement/unloading outputs

Each MPC-card having two separate outputs. They are used for selecting low speed or high speed for the hoisting movement and for the unloading function for the luffing movement. Each output giving a fixed 24V output with a maximum current of 2 A.

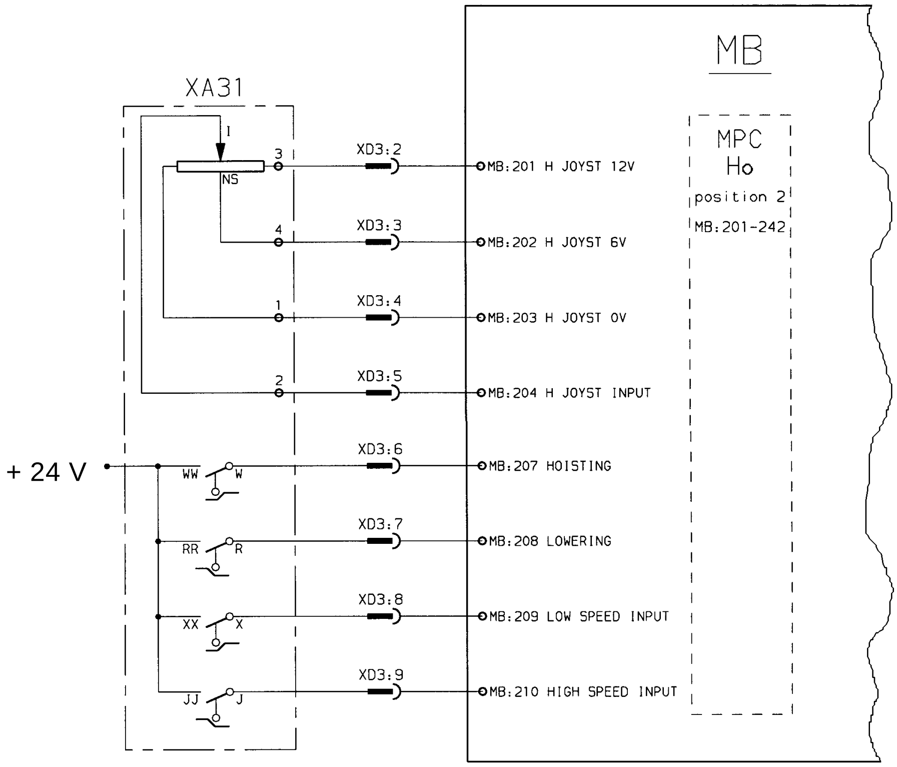

Fig. 2. Control lever signals, hoisting movement.

1.10 Low feed pressure input

The low feed pressure switch indicates that the pump feed pressure is to low for safe operation. Output signals to pumps, displacement/unloading valves and brake relief valves are immediately switched off by a separate safety system on the MPC-card.
If the low pressure signal comes back within approx 0,3 s the crane operation continues with the same output signal levels as before the pressure drop. However, if short pressure drops are repeated 4-5 times within a short period of time the crane operation is stopped and starts 4-5 seconds after the feed pressure has a stable high level again.

1.11 Overload input

The overload pressure switch makes the MPC-card control program switch over from high speed to low speed operation or, if already in low speed, stopping the hoisting and luffing crane movement. The input signal from the overload pressure switch is often only seen as a short pulse.

1.12 Lamp output "Error in control system MB-box"

All MPC-card error LEDs, no 28, are wired together to a lamp in drivers cabin. When an error first occurs the lamp flashes. When the error is acknowledged by pushing the STEP push-button on the appropriate MPC-card the lamp is on if the error remains or out if the error is not present for the moment. See sign ERROR MESSAGES for detailed information about error messages.

1.13 "Speed dependent" overload function / Pressure switch switching

Some cranes have a system that switches from one overload pressure switch to another depending of the speed of the hoisting motor, due to differences in efficiency between low and high speed. At speeds above a programmed limit value the normal pressure switch is used, but at low speed a pressure switch with approx. 20% higher setting is used in the overload system. The control system uses a digital speed encoder to sense the speed of the motor. At speeds lower than approx. 3 rpm, the output signal MB:229 activates a relay in the A-box bypassing the pressure switch 1312. See parameter "speed dependent overload" in cubicle MB.

2. Control system input and output signal levels

Control system interface signal	Plinth no	MPC-card	Signal level
Power for control system input and output signals	MB:42<br>MB:50	All	+24V.<br>0V, connected to ground.
Anti-condensation heating	MB:44<br>MB:46	-	24V AC, powered when crane stopped.
Power for microprocessors on MPC-cards	MB:58 (with fuse)<br><br>MB:54	All	+8V, nominally 9V, minimum 7V.<br>0V.
Cross-connection plinths	MB:60-99	-	Cross-connections for external limit-switches, key-switches, overload-switches etc.
Outputs to control lever potentiometer hoisting	MB:201<br>MB:202<br>MB:203	Ho	12V from MPC "Ho".<br>6V from MPC "Ho".<br>0V from MPC "Ho".
Control lever potentiometer input hoisting	MB:204	Ho	Analogue input signal 1.5 to 10.5V with 6V in neutral position
Control lever direction inputs hoisting<br>Hoisting<br>Lowering<br>Low Speed<br>High speed	 MB:207<br>MB:208<br>MB:209<br>MB:210	 Ho<br>Ho<br>Ho<br>Ho	24V when control lever actuated<br>hoisting<br>lowering<br>low speed<br>high speed
Outputs to control lever potentiometer Luffing	MB:301<br>MB:302<br>MB:303	Lu	12V from MPC "Lu".<br>6V from MPC "Lu".<br>0V from MPC "Lu".
Control lever potentiometer input signal luffing	MB:304	Lu	Analogue input signal 1.5 to 10.5V with 6V in neutral position.

Control system interface signal	Plinth no	MPC-card	Signal level
Control lever direction inputs slewing<br>Luffing In<br>Luffing Out	 MB:307<br>MB:308	 Lu<br>Lu	24V when control lever actu-ated<br>luffing in<br>luffing out
Outputs to control lever potentiometer slewing	MB:401<br>MB:402<br>MB:403	SL	12V from MPC "SL".<br>6V from MPC "SL".<br>0V from MPC "SL".
Control lever potentiometer input signal slewing	MB:404	SL	Analogue input signal 1.5 to 10.5V with 6V in neutral position.
Control lever direction inputs slewing<br>Slewing Left<br>Slewing Right	 MB:407<br>MB:408	 SL<br>SL	24V when control lever actu-ated slewing left<br>slewing right
Pressure/force sensor input	MB:206	Ho	Current signal 4-20mA.
Brake relief valve - outputs<br>Hoisting<br>Luffing - (direction valve if cylinder luffing)<br>Slewing	 MB:230<br>MB:330<br>MB:430	 Ho<br>Lu<br>SL	24V, max. 2A, open circuit gives error message.
Hoisting displacement low speed output	MB:231	Ho	24V, max. 2A, open and short circuit gives error message.
Hoisting displacement output high speed	MB:232	Ho	24V, max. 2A, open and short circuit gives error message.
Luffing unloading output	MB:331	Lu	24V, max. 2A, open and short circuit gives error message.
Pump PWM signals for Hoisting Pump1<br>Hoisting output<br>Lowering output<br>Current return and current measure input	 MB:233<br>MB:234<br>MB:235	 Ho<br>Ho<br>Ho	24V, 0 to approx. 600 mA, error message if wanted current not achieved.<br>1mA gives approx. 1mV.

Control system interface signal	Plinth no	MPC-card	Signal level
Pump PWM signals for hoisting pump2 (if fitted)<br>Hoisting output<br>Lowering output<br>Current return and current measure input	MB:236<br>MB:237<br>MB:238	Ho<br>Ho<br>Ho	24V, 0 to approx. 600 mA, error message if wanted current not achieved.<br>1mA gives approx. 1mV.
Pump PWM signals for luffing pump1<br>Luffing In output<br>Luffing Out output<br>Current return and current measure input	MB:333<br>MB:334<br>MB:335	Lu<br>Lu<br>Lu	24V, 0 to approx. 600 mA, error message if wanted current not achieved.<br>1mA gives approx. 1mV.
Pump PWM signals for luffing pump2 (if fitted)<br>Luffing In output<br>Luffing Out output<br>Current return and current measure input	MB:336<br>MB:337<br>MB:338	Lu<br>Lu<br>Lu	24V, 0 to approx. 600 mA, error message if wanted current not achieved.<br>1mA gives approx. 1mV.
Pump PWM signals for slewing pump1<br>Slewing Left output<br>Slewing Right output<br>Current return and current measure input	MB:433<br>MB:434<br>MB:435	SL<br>SL<br>SL	24V, 0 to approx. 600 mA, error message if wanted current not achieved.<br>1mA gives approx. 1mV.
Pump PWM signals for slewing pump2 (if fitted)<br>Slewing Left output<br>Slewing Right output<br>Current return and current measure input	MB:436<br>MB:437<br>MB:438	SL<br>SL<br>SL	24V, 0 to approx. 600 mA, error message if wanted current not achieved.<br>1mA gives approx. 1mV.
Limit switch inputs hoisting<br>Hoisting<br>Lowering, slack wire<br>High hook	MB:214<br>MB:215<br>MB:216	Ho<br>Ho<br>Ho	24V at normal operation, 0V at limit. At limit movement is stopped and brake closed.
Speed-reduce limit inputs hoisting<br>Hoisting<br>Lowering<br>High hook	MB:211<br>MB:212<br>MB:213	Ho<br>Ho<br>Ho	24V at normal operation, 0V at limit.
Limit switch inputs luffing<br>Luffing In<br>Luffing Out, slack wire<br>High hook	MB:314<br>MB:315<br>MB:316	Lu<br>Lu<br>Lu	24V at normal operation, 0V at limit. At limit movement is stopped and brake closed.

Control system interface signal	Plinth no	MPC-card	Signal level
Speed-reduce limit inputs luffing<br>Luffing In<br>Luffing Out<br>High hook	 MB:311<br>MB:312<br>MB:313	 Lu<br>Lu<br>Lu	 24V at normal operation,<br>0V at limit.
Limit switch inputs slewing<br>Slewing left<br>Slewing right	 MB:414<br>MB:415	 SL<br>SL	24V at normal operation,<br>0V at limit. At limit movement<br>is stopped and brake closed.
Speed-reduce limit inputs slewing<br>Slewing left<br>Slewing right	 MB:411<br>MB:412	 SL<br>SL	 24V at normal operation,<br>0V at limit.
Pressure switch input for low feed pressure	MB:219<br>MB:319<br>MB:419	Ho<br>Lu<br>SL	24V at normal operation,<br>0V at low feed pressure.
Pressure switches for overload	MB:220<br>MB:320	Ho<br>Lu	24V at normal operation,<br>0V at overload.
Output "Error in control system MB"	MB:128<br>MB:228<br>MB:328<br>MB:428	All	24V outputs wired together to error lamp in drivers cabin.
Digital speed encoder hoisting<br>(if fitted)	MB:221<br>MB:222<br>MB:223<br>MB:224	Ho	Digital speed encoder giving four digital 24V pulse inputs.
Output for "Speed dependent" overload function (if fitted).	MB:229	Ho	24V when the hoisting speed is less than approx. 3 rpm.
Output for "Hoisting Overload" indication lamp (if fitted)<br>- valid from software MGX 02.00.	MB:242	Ho	24V when the hoisting pressure exceeds adjusted value.
Speed reduction, input signal ext. 1 (bit 0) to reduce normal operation speed (utilization).	MB:217<br>MB:317<br>MB:417	Ho<br>Lu<br>SL	24V input from cabin seitch, such as Grab operation, Parking Lock Slewing, Power Limiter, Twin operation, etc.
Speed reduction, input signal ext. 2 (bit 1) to reduce normal operation speed (utilization). Same as above, but could be used with different utilization.	MB:218<br>MB:318<br>MB:418	Ho<br>Lu<br>SL	24V input from cabin switch, same as above.

3. Signals indicated on the MPC-card front panels

3.1 Coloured LEDs

24V signals to and from each MPC-card are indicated with green, yellow and red LEDs (Light-Emitting Diodes) on each MPC-card front panel.

Green are used for normal input and outputs. Yellow indicates that the crane motion concerned is driven with slower speed. Red indicates that the motion is stopped.

Normal operation green signals are shown with inputs to the left and the corresponding outputs to the right of the input. They are on when the signal is active, 24V.

Speed reduce limit switches are shown with yellow LEDs when the 24V signal is missing. Speed reduction inputs from key-switches are shown with yellow LEDs when the input is active, 24V.

Stop limit switches, low feed pressure and overload are indicated with red LEDs when the 24V signal is missing.

All signals indicated with LEDs are listed on the sign SIGNAL OVERVIEW inside cubicle MB, see section "Service", Group 6.3.

3.2 LEDs shows the microprocessor information

Signals are indicated at the logic 5V level, close to the microprocessor chip.

When an Input LED is on, that signal has passed the optoisolated 24V to 5V conversion and reached the microprocessor.

When an Output LED is on, the microprocessor outputs the signal at 5V level, but it has not yet passed the 5V to 24V output driver stages. Check the signal output level on the corresponding plinth if missing output is suspected.

3.3 Identifying the plinth number corresponding to a LED

The plinth number is identified by a three digit number. The first digit of each number signifies the MPC-card to which the signal is connected:

1.. Supervisory MPC-card, "Sc".
2.. Hoisting MPC-card, "Ho".
3.. Luffing MPC-card, "Lu".
4.. Slewing MPC-card, "SL".

The last two digits signifies the LED number on each MPC-card front panel, showing the status of the 24V input and output signals for the card. The same two-digit number is used on the cables from the plinth to each MPC-card.

3.4 MPC-card normal readouts at idling

At idling two LEDs are expected on each MPC-card. The green LED no 42 (+24V) is on and the green LED Tx is flashing/glowing. See Fig. 3 and Table 1. (Tx indicates internal communication between the MPC-cards. Tx on the MPC "Sc" is glowing stronger than the others because it sends data three times more often than the others.)
MPC-card displays must show "Sc", "Ho", "Lu" and "SL" respectively.

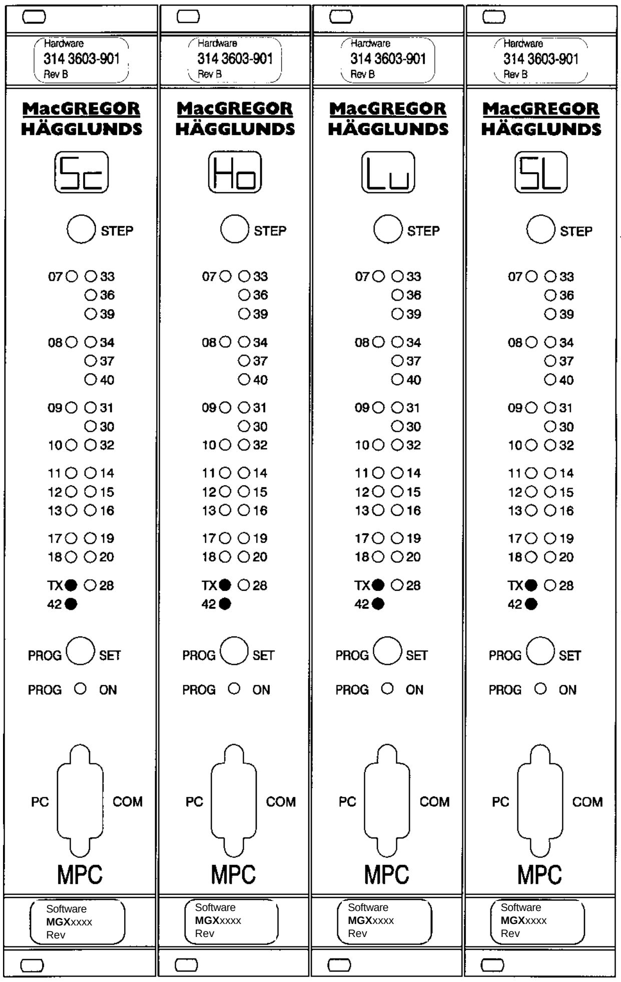

Fig. 3. MPC-card displays and LEDs for idling crane.

MPC "SC"		MPC "Ho"		MPC "Lu"		MPC "SL"	
**Input**	**Output**	**Input**	**Output**	**Input**	**Output**	**Input**	**Output**
42 (+24V)		42 (+24V)		42 (+24V)		42 (+24V)	
Tx		Tx		Tx		Tx	

Table 1. MPC-card readouts for idling crane.

3.5 MPC-card normal readouts when hoisting, luffing and slewing

When hoisting at low speed, luffing in and slewing left the following LEDs are on for each MPC-card (crane with one pump per movement and unloading on luffing). See Fig. 4 and Table 2.

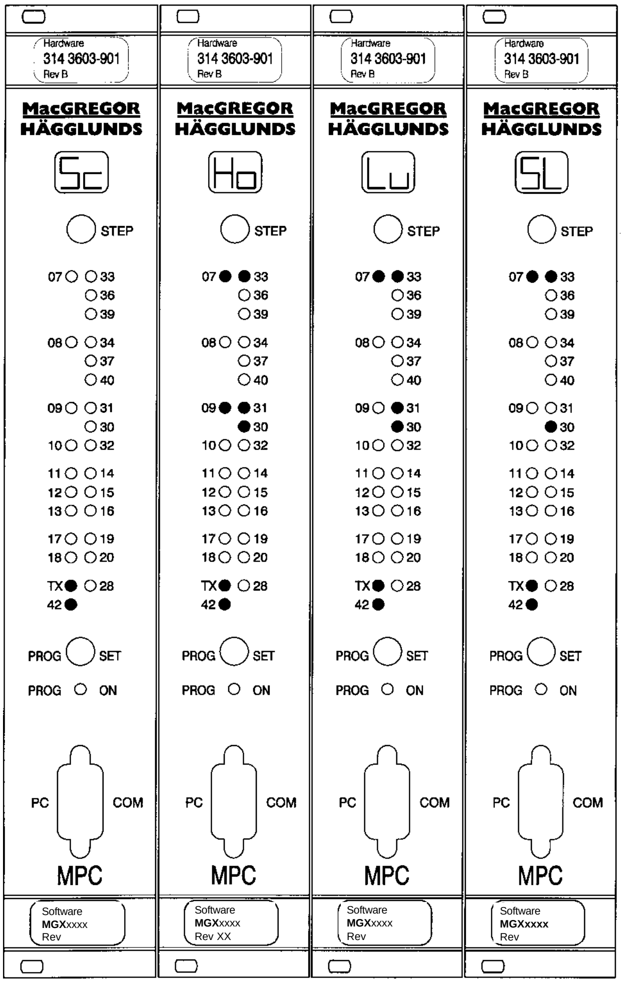

Fig 4. MPC-card displays and LEDs when hoisting at low speed and luffing in and slewing left.

MPC "SC"		MPC "Ho"		MPC "Lu"		MPC "SL"	
**Input**	**Output**	**Input**	**Output**	**Input**	**Output**	**Input**	**Output**
		07 (hoisting)	33 (hoisting pump1)	07 (luffing in)	33 (luffing in pump1)	07 (slewing left)	33 (slewing left pump1)
		09 (low speed)	31 (low speed) 30		31 (unloading) 30 (brake)		30 (brake)
42 (+24V)		42 (+24V)	(brake)	42 (+24V)		42 (+24V)	
Tx		Tx		Tx		Tx	

Table 2. MPC-card readouts when hoisting at low speed and luffing in and slewing left.

4. Troubleshooting the Control system

4.1 General

This section provides information for identifying and correcting malfunctions which may develop while operating the Cargo Crane. Before performing troubleshooting, read and follow all safety instructions found in section "General", Group 1, of this manual. This chapter cannot list all malfunctions that may occur, nor all inspections and corrective actions. If a malfunction is not listed, or is not corrected by the listed corrective actions, notify your supervisor.

When troubleshooting a malfunction: Locate the symptom or symptoms that best describes the malfunction. If the appropriate symptom is not listed, notify your supervisor.

Perform each step in the order listed until the malfunction is corrected.

Note!
Read this section in conjunction with the electrical and hydraulic schemes provided in Group 9.5 and 9.4.

4.2 Hoisting, Luffing or Slewing Movement Do Not Function

(a) Power supply 24V and 8V to MB-cubicle.
- (1) Check overcurrent relay HC3 in HC-cubicle.
- (2) Check fuse in plinth MB:58 in MB-cubicle, only 8V.
- (3) Check fuses, 1 to 6 inclusive, in CE-cubicle.
- (4) Check rectifier unit in CE-cubicle.
- (5) Check relay A90 in A-cubicle (see Circuit Diagram Crane).

(b) Control system, MB-cubicle.
- (1) Check control system signals in MB-cubicle.
  - (a) No LEDs on and no display readout - indicates missing 8V.
  - (b) Check for Red LEDs - indicating stop limits.
    - (1) If LED 28 on or flashing, see sign ERROR MESSAGES.
  - (c) Check for Yellow LEDs - indicating speed reduces.
  - (d) Check for missing Green input signal LEDs - from joystick.
  - (e) Check analogue input signal from joystick potentiometer.
  - (f) Check for missing Green output signal LEDs.
    - (1) Exchange the MPC-card with other movement MPC-card.
    - (2) Replace broken MPC-card with a new one.
    - (3) Replace broken MPC-card with MPC "Sc" and use "MPC default operation mode" temporarily.
    - (4) Use an Emergency operation card, temporarily.

- (2) Check control lever (joystick).
  Check electric cable connections.

- (3) Check limit switches.
  Check electric cable connections.
  Adjust limit switches.

- (4) Check valves 1221, 1226, 2221, 2226 and 3221.
  Check electric cable connections.
  Replace electric coil.
  Replace valve.

- (5) Check pump servo valves 1111-9, (1112-9), 2111-9, (2112-9) and 3111-9.
  Check electric cable connections.

5. Operating the crane with less than four MPC-cards

5.1 General

With one or more broken MPC-cards the crane can temporarily be operated with remaining cards. Each MPC-card can operate stand-alone in "MPC default operation mode". Each card (1, 2 or 3 cards totally) operates stand alone with its in-built default parameter values instead of the normal parameter values distributed by the MPC "Sc". The speed control is still stepless and safety functions as limit switches, low pressure and overload protection are still working. The maximum speed of each crane movement is reduced but is still at least 50% of normal operation.

5.2 Actions taken to activate the "MPC default operation mode":

- Switch off the power to the MB-box by stopping the crane.
- Remove the MPC "Sc" from the rack.
- Replace the broken MPC "Ho"/"Lu"/"SL"-card with MPC "Sc". Leave the MPC "Sc"-position empty.
- Start the crane, it can be operated as normal but with lower maximum speed and the error lamp "Control system error in MB-box" is flashing.

The error lamp "Control system error in MB-box" is now flashing, indicating that there are no communication with (the missing) MPC "Sc". Acknowledge these errors by pushing the STEP push-button twice on each MPC-card. First the error number 41 should be displayed and at the second push the sign "—" that indicates the end of error list. If there are any other error numbers than 44 the crane may still be inoperable, otherwise the error lamp 28 on each MPC-card and the lamp in the cabin shall be on, not flashing.
For crane movements with less than three pumps (normally 1 or 2 pumps) these MPC-cards gives error messages when operating their movement. Error messages are given for unused pumps because in this mode the MPC-card tries to use all three pumps even if there are no pump2 and pump3 connected. However the crane can still be operated because these error messages are only for information in "MPC default operation mode".

5.3 Emergency operation card

In case of MPC-card problems, there are also a possibility to replace the card with an Emergency operation card which is much less complex than the MPC-card. It is built up with relays that activates the outputs for brake, displacement/unloading and pump from the control levers 24V signals. The pump output is fixed, giving a constant speed of about 30% of maximum speed. Each motion is controlled by overload, low pressure and limit switches except the limit switch for high hook.

WARNING!
When the emergency operation card is installed in the hoisting or luffing circuit, bear in mind that the high hook switch is inoperative.

6. Spare parts

6.1 MPC-card, 314 3603-8xx

The ready-to-use MPC-card, no. 314 3603-8xx, (where -8xx means anyone of -801, -802, -803 etc.) consists of a hardware electronic card loaded with software. For correct art. no. see spare part list, section 9.5.

When ordering and MPC-card without knowing the art. no. (-8xx) specify the software version needed proceed as follows.

Look for:
- the software version specified on the replaced card.
- the software version specified on the parameter list in the MB-box.

or:
- refer to the crane's mfg. no., which can be found on the sign in crane operator's cabin.

Customers that purchase a replacement card will receive a credit note of 15% of the sales price when the damaged card has been returned to MacGREGOR Cranes in Örnsköldsvik.

6.2 Parameter Memory Card, 314 3605-801

The parameter Memory Card, ordering no **314 3605-801, is not a general spare part.** Each parameter Memory Card needs to be programmed with the crane's unique parameter values (see parameter list in MB-box). When ordering a parameter Memory Card always specify the manufacturing number (Mfg. no.), which can be found in the MB-box or on the sign in the crane operator's cabin.

The programmed Memory Card delivered by us, will be labelled with the crane mfg. no.

MacGREGOR
HÄGGLUNDS

Memory Card 314 3605-801

consists of

Hardware: **314 3605-901**
Software: **6250xxxx**

6.3 Emergency operation card, 314 3615-801

The emergency operation card is used instead of one of the three MPC-cards for hoisting, luffing or slewing movement.

6.4 Return report

It is essential that the service department gets information about when and why the card is removed from the crane system. Fill out the Return form, see Group 6.0, and send it back with the exchanged card.

6.5 Conductive transit boxes

To protect all types of electronic cards from damages from ESD (Electro Static Discharge) it is important to use pink anti-static plastic bags and conductive transit boxes of the same type as used for delivered original spare parts.

MacGREGOR	SIGNAL OVERVIEW	*Seite* 1 (1)	*Datum* 2001-12-12
	Single and Twin Cranes **Crane Control System CC2000**	*Nr* 4146848-001	*Revision* F

LEDs
24V Input and Output signals are indicated with coloured LEDs.

Colour	Indicating	LEDs on when
Green	Normal inputs and outputs	+24V
Yellow, 11,12,13	Speed reduce limits	0V
Yellow 17,18	Speed reduction switches	+24V
Red	Limits switches, low pressure and overload	0V

LED 42 connected to plinth number 42 and indicates power +24V DC to the card.

LED Tx indicates that the card is transmitting serial data to other cards on an internal data bus.

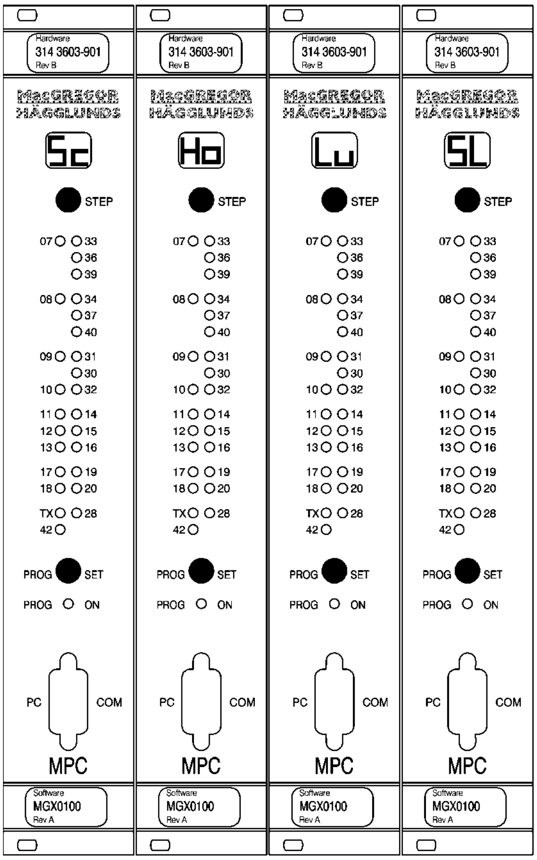

Plinth number
Combine card no with LED no

Sc, Supervisory card	card no 1	1
Ho, Hoisting card	card no 2	2
Lu, Luffing card	card no 3	3
SL, Slewing card	card no 4	4

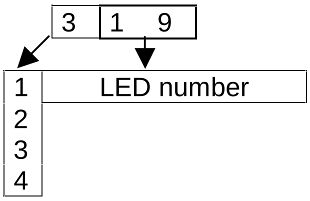

	Sc - supervisory		Ho - hoisting		Lu - luffing		SL - slewing
**Plinth no**	**Signal description**	**Plinth no**	**Signal description**	**Plinth no**	**Signal description**	**Plinth no**	**Signal description**
**107**	Twin control on, input	**207**	Hoisting, input	**307**	Luffing in, input	**407**	Left, input
**108**	Twin reset, input	**208**	Lowering, input	**308**	Luffing out, input	**408**	Right, input
**109**	Twin, encoder calibration	**209**	Low speed, input				
**110**	Not used	**210**	High speed, input				
**111**	Connected to +24V	**211**	Speed reduce hoisting	**311**	Speed reduce luffing in	**411**	Speed reduce left
**112**	Connected to +24V	**212**	Speed reduce lowering	**312**	Speed reduce luffing out	**412**	Speed reduce right
**113**	Connected to +24V	**213**	Speed reduce high-hook	**313**	Speed reduce high-hook		
		**214**	Limit switch hoisting	**314**	Limit switch luffing in	**414**	Limit switch left
		**215**	Limit switch lowering	**315**	Limit switch luffing out	**415**	Limit switch right
		**216**	Limit switch high-hook	**316**	Limit switch high-hook		
**117**	Not used	**217**	Speed reduction, ext1	**317**	Speed reduction, ext1	**417**	Speed reduction, ext1
**118**	Not used	**218**	Speed reduction, ext2	**318**	Speed reduction, ext2	**418**	Speed reduction, ext2
		**219**	Low pressure switch	**319**	Low pressure switch	**419**	Low pressure switch
		**220**	Overload switch input	**320**	Overload switch input		
**128**	Alarm (error in CT2(MB))	**228**	Alarm (error in CT2(MB))	**328**	Alarm (error in CT2(MB))	**428**	Alarm (error in CT2(MB))
**130**	Not used	**230**	Hoisting brake, output	**330**	Luffing brake, output	**430**	Slewing brake, output
**131**	Not used	**231**	Low speed, output	**331**	Unloading, output		
**132**	Not used	**232**	High speed, output				
**133**	Not used	**233**	Pump1 hoisting, output	**333**	Pump1 luff in, output	**433**	Pump1 left, output
**134**	Not used	**234**	Pump1 lowering, output	**334**	Pump1 luff out, output	**434**	Pump1 right, output
		**236**	Pump2 hoisting, output	**336**	Pump2 luff in, output	**436**	Pump2 left, output
		**237**	Pump2 lowering, output	**337**	Pump2 luff out, output	**437**	Pump2 right, output
		**239**	Pump3 hoisting, output				
		**240**	Pump3 lowering, output				
**42**	+24V	**42**	+24V	**42**	+24V	**42**	+24V

No	Description	Priority
**0x,1x**	**SYSTEM**	
01	Program restart error (reset)	**PRI 3**
02	Address error (MPC-address not valid)	**PRI 3**
05	Stack overflow error	**PRI 1**
09	No MPC-software	**PRI 3**
10	Temperature error	**PRI 1**
11	Battery low on parameter memory card	**PRI 1**
12	Ref voltage (5V) error	**PRI 3**
13	24 V error	**PRI 3**
14	MPC relay error	**PRI 3**
**2x,3x**	**PARAMETER ERROR**	
20	Parameter error gl (global)	**PRI 1**
21	Parameter error Sc (supervisor)	**PRI 1**
22	Parameter error Ho (hoisting)	**PRI 1**
23	Parameter error Lu (luffing)	**PRI 1**
24	Parameter error SL (slewing)	**PRI 1**
25	Parameter error Cl (closing)	**PRI 1**
26	Parameter error Au (auxiliary)	**PRI 1**
30	Parameter missing	**PRI 1**
31	Parameter error twin calibration	**PRI 1**
**4x,5x**	**COMMUNICATION**	
40	Comm. Error RS485 (general)	**PRI 1**
41	Comm. Error RS485 Sc (pos 1) (in all MPC)	**PRI 1**
42	Comm. Error RS485 Ho (pos 2) (only in Sc)	**PRI 1**
43	Comm. Error RS485 Lu (pos 3) (only in Sc)	**PRI 1**
44	Comm. Error RS485 SL (pos 4) (only in Sc)	**PRI 1**
45	Comm. Error RS485 Cl (pos 5) (only in Sc)	**PRI 1**
46	Comm. Error RS485 Au (pos 6) (only in Sc)	**PRI 1**
50	Comm. Error RS232 (terminal)	**PRI 1**
51	Comm. Error MEM_I^{2}C (par. and log. mem)	**PRI 1**
52	Comm. Error EXP_I^{2}C (expansion i^{2}c)	**PRI 1**
53	Comm. Error COM1 (encoder)	**PRI 1**
54	Comm. Error COM2 (twin crane CIC card)	**PRI 1**
55	No speed detected from speed encoder	**PRI 1**
56	4-20 mA signal error, input X06	**PRI 1**
**6x,7x**	**EXTERNAL signals**	
60	Joystick level error (normal is1,5 to 10,5V)	**PRI 3**
61	Joystick logic error LOR	**PRI 3**
62	Joystick logic error HIL	**PRI 3**
63	Brake signal error (short circuit)	**PRI 3**
64	Low speed/unload signal error (short circuit)	**PRI 3**
65	Low speed/unload signal error (open circuit)	**PRI 3**
66	High speed signal error (short circuit)	**PRI 1**
67	High speed signal error (open circuit)	**PRI 1**
74	PWM1 LOR current signal error	**PRI 2**
75	PWM1 HIL current signal error	**PRI 2**
76	PWM2 LOR current signal error	**PRI 2**
77	PWM2 HIL current signal error	**PRI 2**
78	PWM3 LOR current signal error	**PRI 2**
79	PWM3 HIL current signal error	**PRI 2**
**8x,9x**	**Function generated errors**	
80	This MPC-card generated system stop	**PRI 3**
81	System stop	**PRI 3**
82	RTS overflow (programming error)	**PRI 2**
90	Error in twin slave crane	**PRI 1**
91	Overspeed limit exceeded	**PRI 2**
92	Wrong direction according to joystick	**PRI 2**
93	Faulty position encoder	**PRI 1**

How to display error messages

When any type of malfunction is detected the MPC-card flashes with its error LED, no 28. All MPC-card error LEDs are wired together to an error lamp in the drivers cabin, the ”Control system error (in MB-box)” lamp. To get the corresponding error message number, push the STEP push-button on the MPC-card with flashing LED.

If there are several errors they are shown one at a time for each push on the STEP push-button. When there are no more error messages to read, the display shows two hyphens ”--”. The error LED is now either on or off. If it is on the error still is present, but now acknowledged. If it is off the errors are not present at the time. When a new error is detected the error LED starts flashing again to indicate that a new error is found.

Flashing LED no 28:	New error, not acknowledged.
LED no 28 on:	Error remains, now acknowledged.
LED no 28 off:	Error not present at the moment.

Five seconds from last push of the push-button ”STEP” the display goes back to its normal mode (Sc,Ho,Lu,SL).

If the system is restarted (i.e. 8V power supply is switched off for a short duration) all alarms will disappear and they are not coming back until the error occurs again.

Error priority

PRI 1	Only for information.
PRI 2	The movement with error is stopped unless the crane is put into MPC Default Operation Mode.
PRI 3	The movement with error is stopped also in MPC Default Operation Mode.

MPC Default Operation Mode

If one MPC card is broken/faulty the crane can be operated with only three MPC cards. Replace the broken card with MPC ”Sc” by proceeding as follows.
Turn off the power, by stopping the crane.
Pull out the MPC ”Sc”-card.
Turn the power on again.
Now the crane can be operated, but with lower speed.

Abbreviations

HIL	**H**oisting, Luffing **I**n or Slewing **L**eft movement
LOR	**L**owering, Luffing **O**ut or Slewing **R**ight movement
PWM1, PWM2 or PWM3	**P**ulse **W**idth **M**odulated 24V output signal to pump 1, pump 2 and pump 3.
RS232, RS485, I2C	Communication protocols

Electrical Equipment, General Maintenance

Introduction

General maintenance of the electric equipment of the crane is important in order to prevent unexpected breakdowns. The following general advice may be useful. Relevant block diagrams and detailed circuit diagrams are placed in section 9, SPARE PARTS.

Start the crane and try out all combinations of movements that are practicable. This will show if the electric functions are satisfactory.

Inspect all electrical equipment cabinets, and components. Any defective component must be repaired or replaced without delay. A detailed inspection and overhaul at two-year intervals is recommended. Check that all screws and nuts are securely tightened in the contactor cabinets, that all switches and pushbuttons operate correctly, and that cable connections are perfectly tight and all insulating parts intact. Make sure that inspection door gaskets seal perfectly.

Danger!
Electrical equipment can be alive unless the main switch and the separate supply is disconnected.

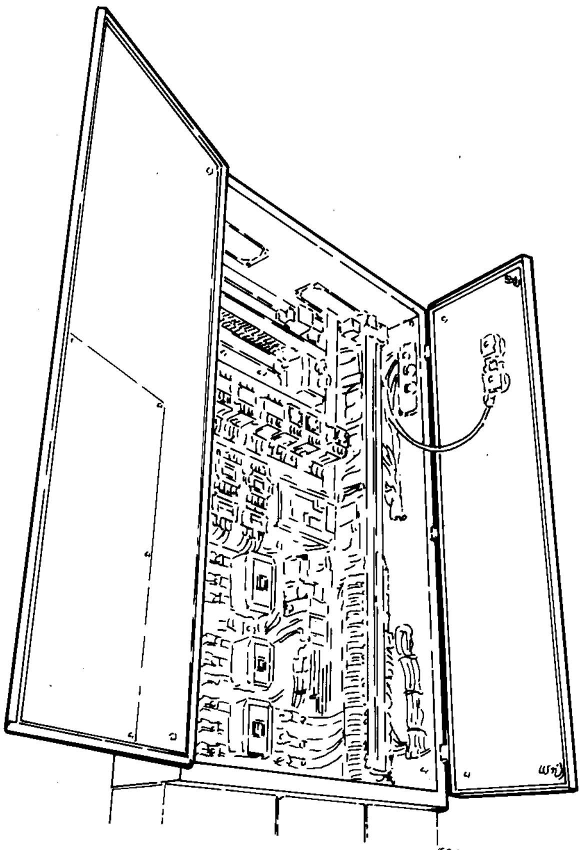

Fig.1. Electrical equipment cabinet

Earth connection, hull

Verify that the protective earth connection of the crane is made to the ship's hull.
Clean contact surfaces, when required.

Earth connection, crane

Check crane system earth connections, and clean contact surfaces as required.

Portable control boxes

Control panels and remote control boxes may be subjected to abuse an abnormal wear, and should be inspected after every use. The control boxes must be stored in a compartment that is well protected against moisture. Remove moisture that may have collected in the boxes, and find out how it entered.

- Clean contacts, insulating parts, cams, contact rolls, and rotary potentiometers.
- Make sure that all cable connnections are clean and securely tightened.
- Clean the control levers, and grease their sliding surfaces.
- Check on the return springs and grease lightly so that no grease transfers to the contacts.

Jib floodlight

Make sure that connections in the junction box are clean and well tightened.

- Remove moisture and dirt.
- When necessary, clean contact surfaces of terminal strips and of lamp holder.
- Make sure that lamp holder gasket seals correctly.
- Make sure that the work light can be rotated.

Slip-ring unit

Danger!
Before opening the slip-ring unit, make sure that power supplied by separate transformers is switched off in the ship's engine room. Also check by means of a voltmeter that the slip-ring unit is not alive.

- Verify that slip ring contact surfaces are smooth and even, clean as required.
- Check contact pressure of brushes.
- Replace carbon brushes or entire slip ring assemblies as required.
- Make sure that cable connections are securely tightened.
- Make sure that rocker arm mounting screws are securely tightened.

The removal and installation of the slip-ring unit is the subject of a separate description.

Oil thermostat

The task of the oil thermostat is to protect the crane against overheating.

Verify that the thermostat is set to +85°C, and that it functions correctly. The thermostat is placed on the oil cooler inlet line.

Slip-Ring Unit

Danger!
Before opening the slip-ring unit, make sure that power supplied by separate transformers is switched off in the ship's engine room. Also check by means of a voltmeter that the slip-ring unit is not alive. Make sure that the current can not be switched on again during the maintenance operation.

Work on a slip-ring assembly may only be carried out by a qualified electrician or by persons under the supervision of a qualified electrician and only according to accepted electrical safety procedures.

The function of the slip-ring assembly may not be changed in any way. Especially safety devices must not be made ineffective. Other than during maintenance and inspection work the housing must always remain closed.

Use only insulated tools and original spare parts.

No responsibility will be assumed for damage to property or injury to persons caused by improper use of the slip-ring assembly.

Removal

1. Park the jib in a suitable manner.
2. Disconnect the ship's power supply at all points of feed.
3. Disconnect all the cables connection from crane- and ship supply.
4. Remove the driver connecting the slip-ring unit and the foundation. Note the cables mounting sequence.
5. Remove mounting screw holding the slip-ring unit on cranehouse bottom. See Fig. 1.
6. Remove the slip-ring unit.

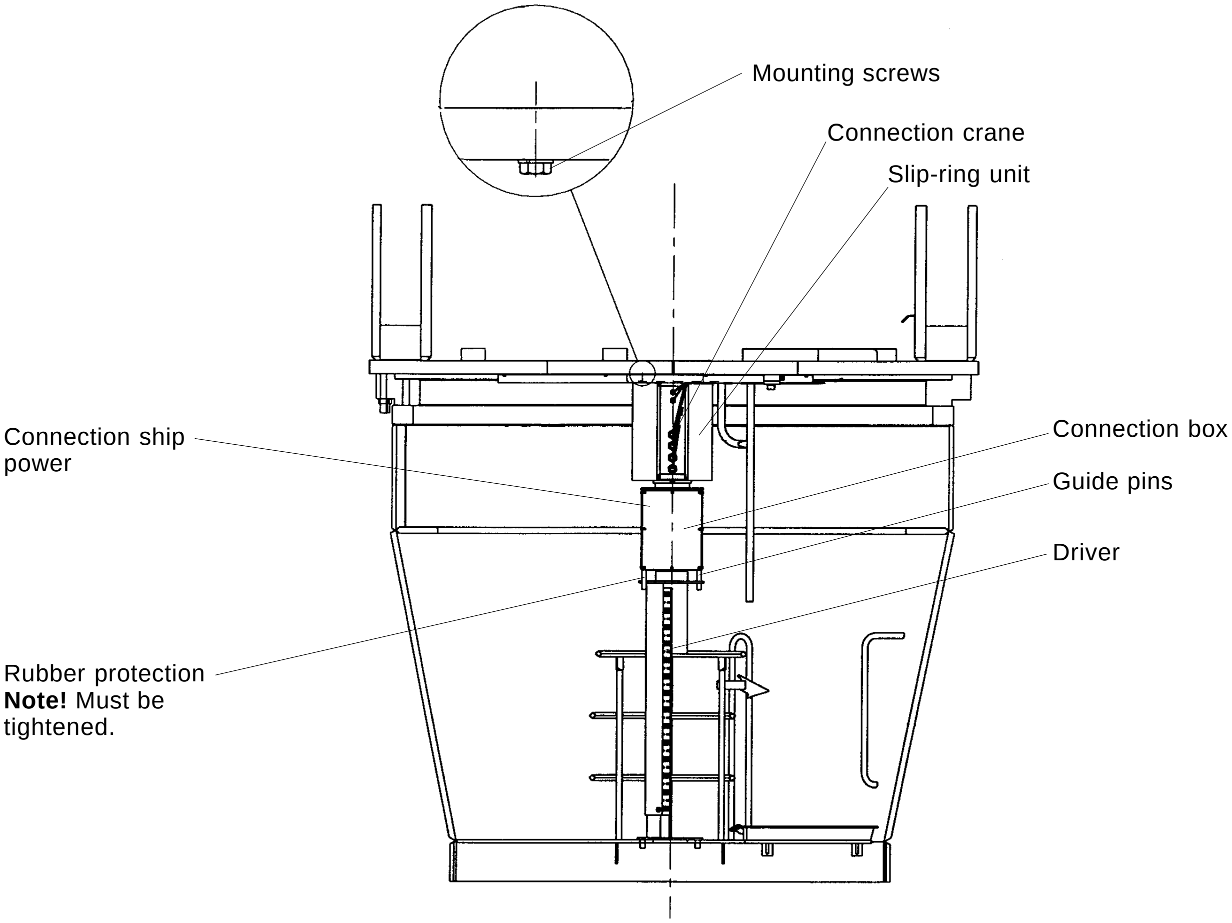

Fig. 1. Slip-ring unit.

Any extra equipment such as extra limit switches fitted on top of the slip-ring unit are removed prior to removal of the entire slip-ring unit.

Installation

Clean all contact surfaces between slip-ring unit and cranehouse bottom. Tighten all screws properly. See instruction "Tightening torque".

- Assemble the slip-ring unit under crane bottom
- Assemble the driver to the bottom of the foundation
- Remove the cover around the slip-ring unit
- Fit the slip-ring unit guide pins to the driver's slotted holes by pulling apart the driver and lock it with help of the through bolt
- Install the cables against the driving bar
- Connect the crane cables from the crane on correct mounting sequence
- Remove the connection box plates
- Connect the cables to the plinths

After installation

- Check tightness of all electric and mechanical fasteners regularly for the first working period.
- Check alignment. The slip-ring unit must not be allowed to slope in relation to the driver.
- Rotate the crane ensure that no mechanical side forces are transferred to the unit.
- Tighten the rubber protection , see Fig. 1.

Maintenance

Always cut current before accessing the collector for inspection or maintenance. Make sure that the current can not be switched on again during the maintenance operation.

At regular intervals

- Check collector for damaged parts
- Check contact pressure of brushes
- Check brush holders

Carbon brushes

- The carbon brushes must never wear down so far that metal parts of the brush holder come into contact with the slip-ring.
  The carbon dust is to be removed with dry, oil free, compressed air or with a vacuum cleaner.
  No substance containing oil or grease may come into contact with the brushes or the slip-rings.

Wire brushes

- Check wire brushes.
  Debris caused by wear is to be removed with dry, oil free, compressed air or with a vacuum cleaner.
  Afterwards the slip-ring ways and wire brushes should be lightly sprayed with "Cramolin B" or equivalent contact spray.

Others

- Check tightness of electric and mechanical fasteners.
- Check gaskets and cable glands for tightness.

Keep grease and oil products away from the plastic insulating parts in the collector.

Oil Level Float Switch and Overheating Protector

Introduction

To prevent operation of the crane with too little oil in the hydraulic oil tank, an oil level float switch is installed in the oil tank. The level float switch operates alarm signals on the control panel in the crane operator's cab and a siren. Warning lamps and a selector switch are placed on the right-hand control panel in the cab (see Fig. 1). In normal operation, the switch should be in the position shown in the illustration.

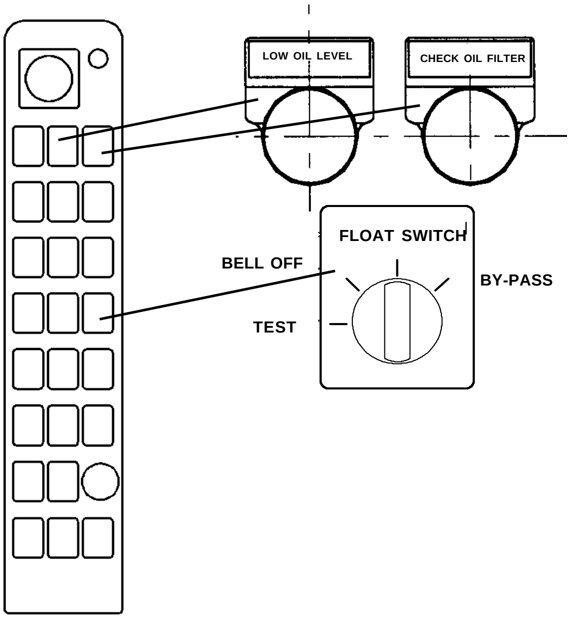

Fig. 1. Right Hand Control Panel.

Fig. 2 shows the design of the oil level float switch. Normally, the float is at its topmost postion (i.e. when the level in the hydraulic tank is sufficient).

The float controls switches which in turn, control the warning lamps and the bell.

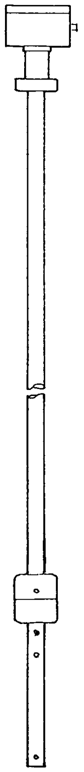

Fig. 2. Oil Level Float Switch.

Oil Cooling

The oil cooler starts when the oil temperature exceeds 10^0C.

Normal Oil Level

Normal oil level is between marks MIN and MAX and LOW OIL LEVEL warning lamp is extinguished. Selector FLOAT SWITCH (see Fig. 3.03) must be in position shown.

Oil Level Float Switch Function

A low oil level should be remedied as soon as possible operating conditions permit. If this is not practicable, certain emergency measures may be undertaken as described below.

Oil Level MIN

If the oil level drops to MIN, the float activates the upper switch and lamp LOW OIL LEVEL goes on. At the same time, the alarm bell sounds.

Oil Level MIN, Continued Operation

In an emergency, the operation of the crane may be continued for a short time provided that there is no important leakage of oil. In this case, turn selector FLOAT SWITCH to position BELL OFF; the bell will then be silent and lamp LOW OIL LEVEL goes out.

Oil Level Below MIN

When the oil level has dropped a certain distance below MIN level, the float activates the lower switch and stops the crane. LOW OIL LEVEL lamp goes on.

Oil Level Below MIN, Emergency Operation

In an extreme emergency (e.g. if the crane has stopped with a load suspended on the hook) the crane can be started anew and operated if selector FLOAT SWITCH is turned to position BY-PASS. The lamp LOW OIL LEVEL goes on and the alarm bell sounds. This is permissible if no important leakage is present, but is entirely at the risk and **responsibility of the crane operator or the officer who gives the order.**

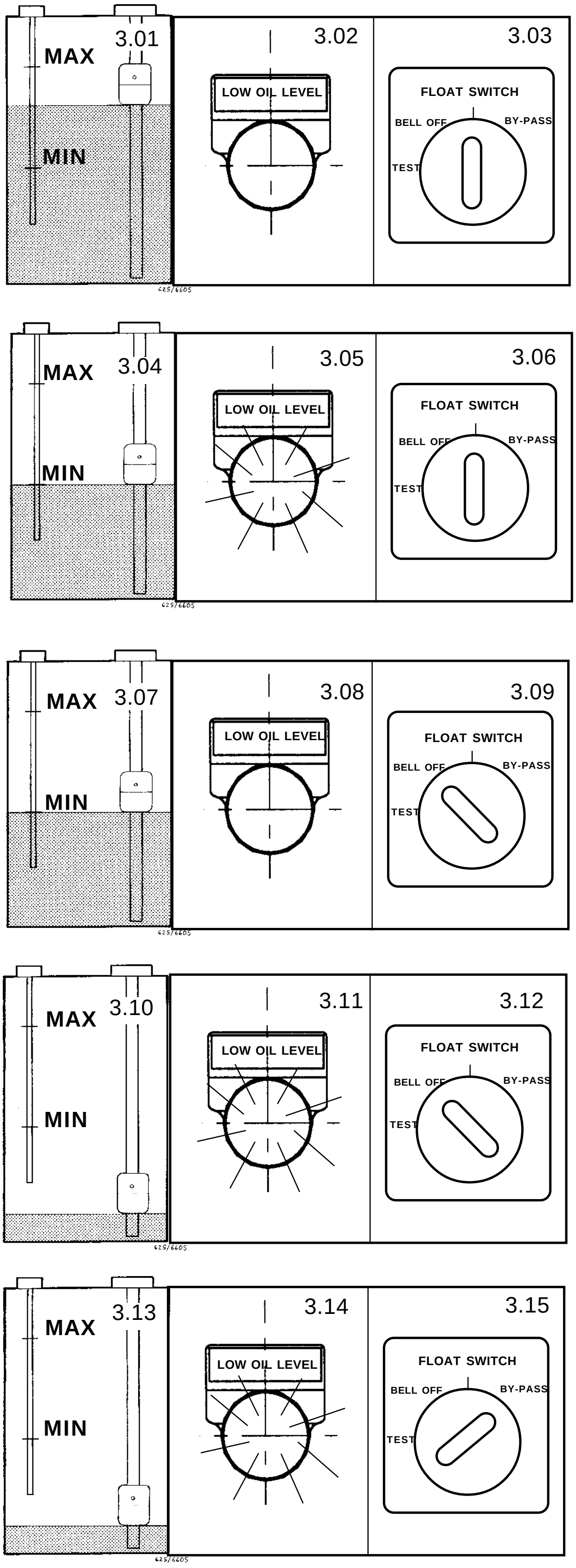

Fig. 3. Sub-Level Operation.

Testing warning lamps

With selector FLOAT SWITCH in TEST position (see Fig. 4) DO NOT OPERATE and LOW OIL LEVEL lamps Illuminate and the alarm bell sounds. The function and continuity of these lamps is checked in this manner.

Testing Oil Level Float Switch

The float of the level float switch is fitted with a built-in magnet which, makes a control circuit when the oil level drops below certain levels. The function of the float is preferably tested when performing oil changes or tank cleaning. For the purpose of this test, the main switch will have to be temporarily switched on.

Overheating Protector

The crane stops automatically when the oil temperature exceeds 85°C. The overheating protector is fitted on the inlet line of the oil cooler (see Fig. 6).

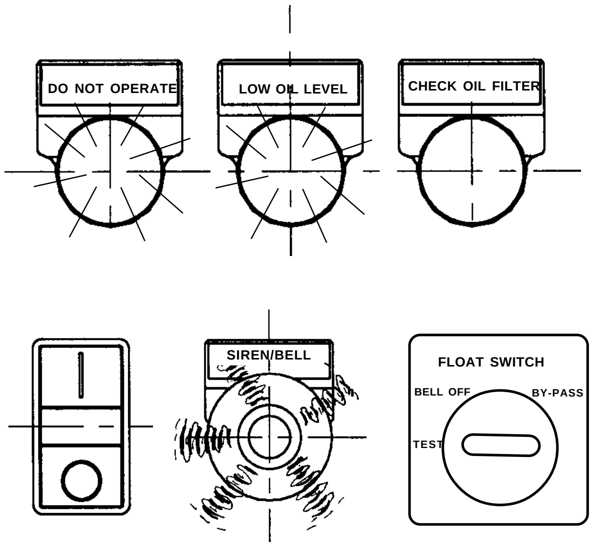

Fig. 4. Selector FLOAT SWITCH, Position TEST.

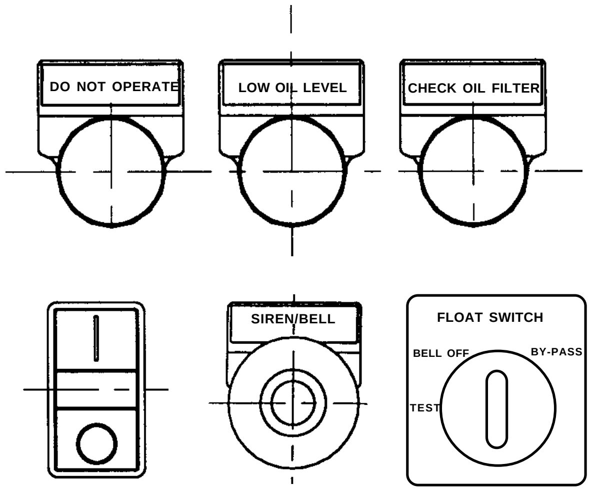

Fig. 5. Part of Right hand Control Panel.

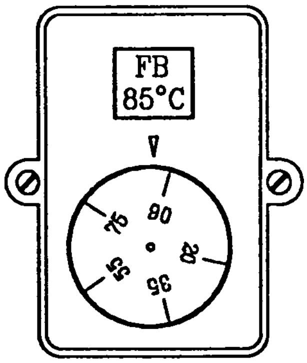

Fig. 6. Overheating Protector.

Limit Switches in Hoisting and Luffing Winch Systems

The limit switches of the hoisting and luffing winches are contained in switch boxes in the cranehouse, one box for each winch system. Every switch function is set separately by means of a rotating cam which controls a microswitch. The cams are driven by the winches through gearboxes.

The location of the switch boxes is shown in the figure.

The limit switch system performs the following functions:

1. Stop function - hook and jib movements are halted at chosen safety limits.

2. Speed reduction - the speed of a hook or jib movement is reduced to 25-30% at a predetermined point before the stop position is reached.

3. Position control function - the jib working radius and the level of the crane hook are controlled in relation to each other.

The limit switch boxes of the hoisting and luffing winches are similar except for the demultiplication ratios of their driving gearboxes.

The cams in the luffing winch limit box are marked GD1, GD2, GD3 etc.; those of the hoisting winch box, GE1, GE2, GE3 etc.

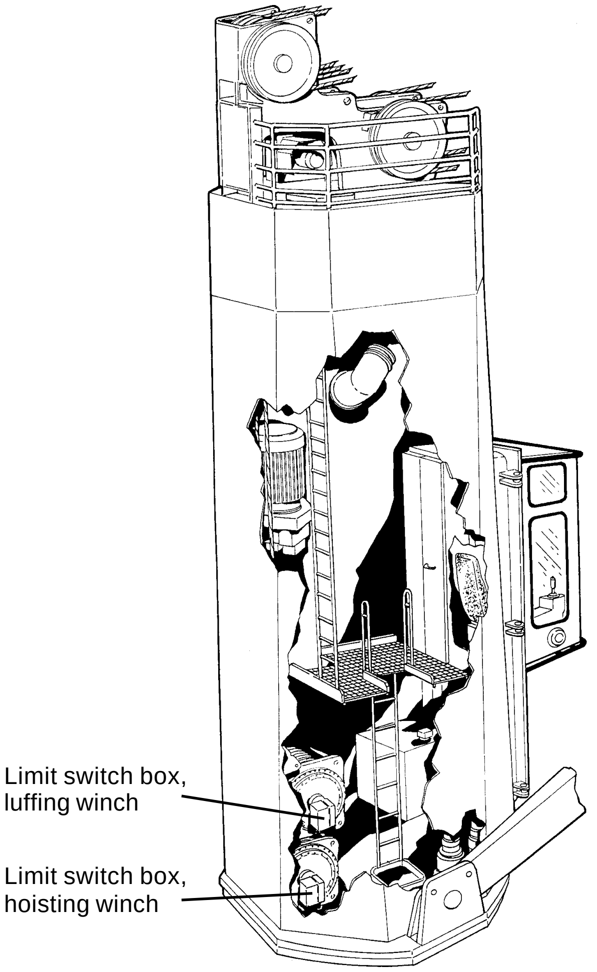

Limit switch boxes in cranehouse

CAUTION!

Store Keys Safely

When the crane is rigged ready for operation, all keys for all lockable switches must be stored in a safe place by the responsible officer on board.

Setting limit switches

Back off the lockscrews of the switch cams in the limit switch boxes, using a 10 mm articulated wrench; the cams are then easily rotated to their proper settings.

After setting the cams correctly, retighten the lockscrews and operate all crane movements, beginning at reduced speed and finishing at maximum speed to check cam settings.

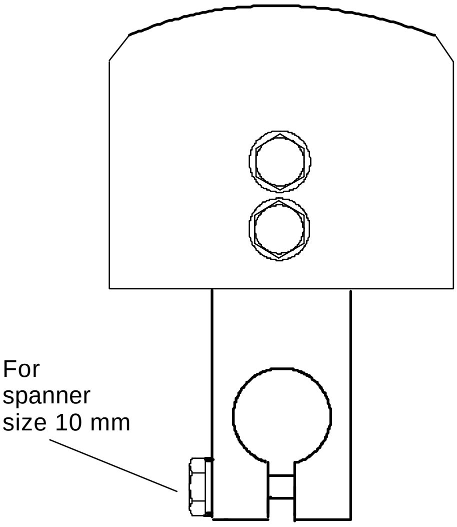

Cam

Measure references

The following measures are stated in the document TECHNICAL DATA under the section 2 TECHNICAL DESCRIPTION.

Jib Radius, Max	(m)
Jib Radius, Min	(m)
Jib Radius, High Hook Limit	(m)
Lifting Height, H	(m)
Lifting Height, K	(m)

Other measures are stated in the figures.

Please note that the Jib radius measures refer to the crane house slewing centre (Not to the jib bearing centre!). The measures H and K refer to the jib bearing centre.

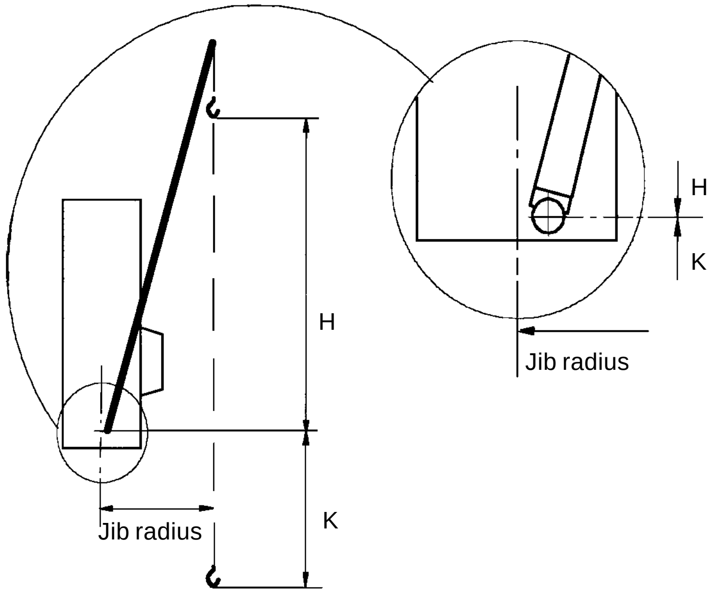

Jib radius lifting height

Luffing winch limit switches

The included limit switches are shown in the below figs. Note that switches GD2 and GD4 actuate speed reduction of the crane jib.

NB. When adjusting the luffing limits it is important to start with GD1 and to continue with GD2, GD3 GD4 and so on.

When changing wires it is important that the procedure in instruction "Replacement of Wires", Section 6.2, is followed, thus to avoid readjustments of limits.

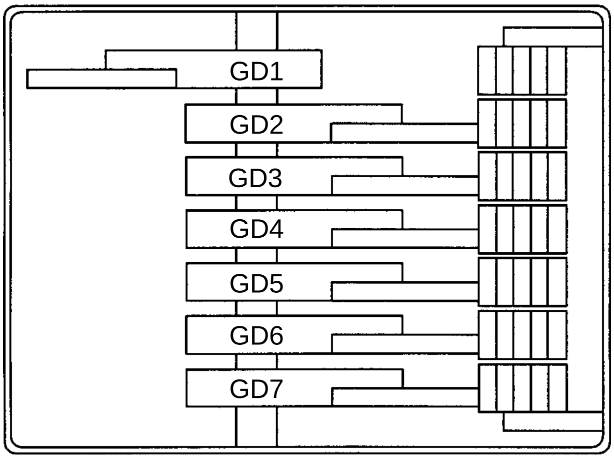

Limit switch box on the luffing winch

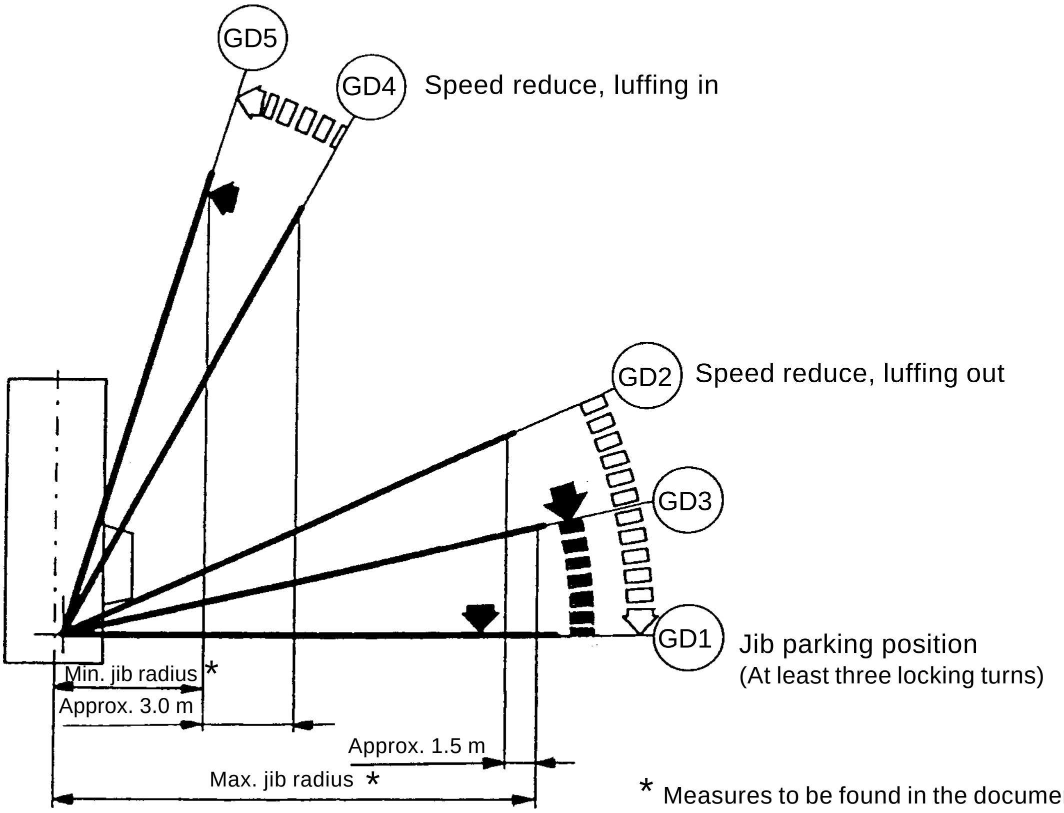

* Measures to be found in the document Technical Data, section 2.

Luffing winch basic limit switches

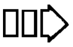 = Speed reduce

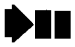 = Stop. To by-pass those positions correct operation mode has to be selected by help of the key switch in cabin.

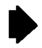 = Stop.

Hoisting winch limit switches

Raise the jib into the MIN Jib radius position, which is the correct jib position for setting the limit switches according to the below figs. Note that the switches GE2 and GE3 actuate speed reduction of the crane hook.

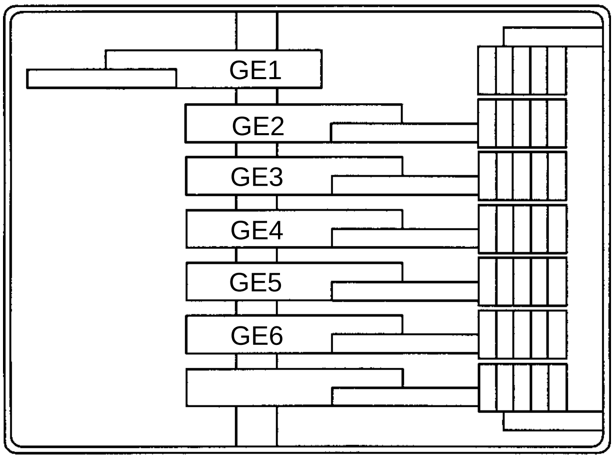

Limit switch box on the hoisting winch

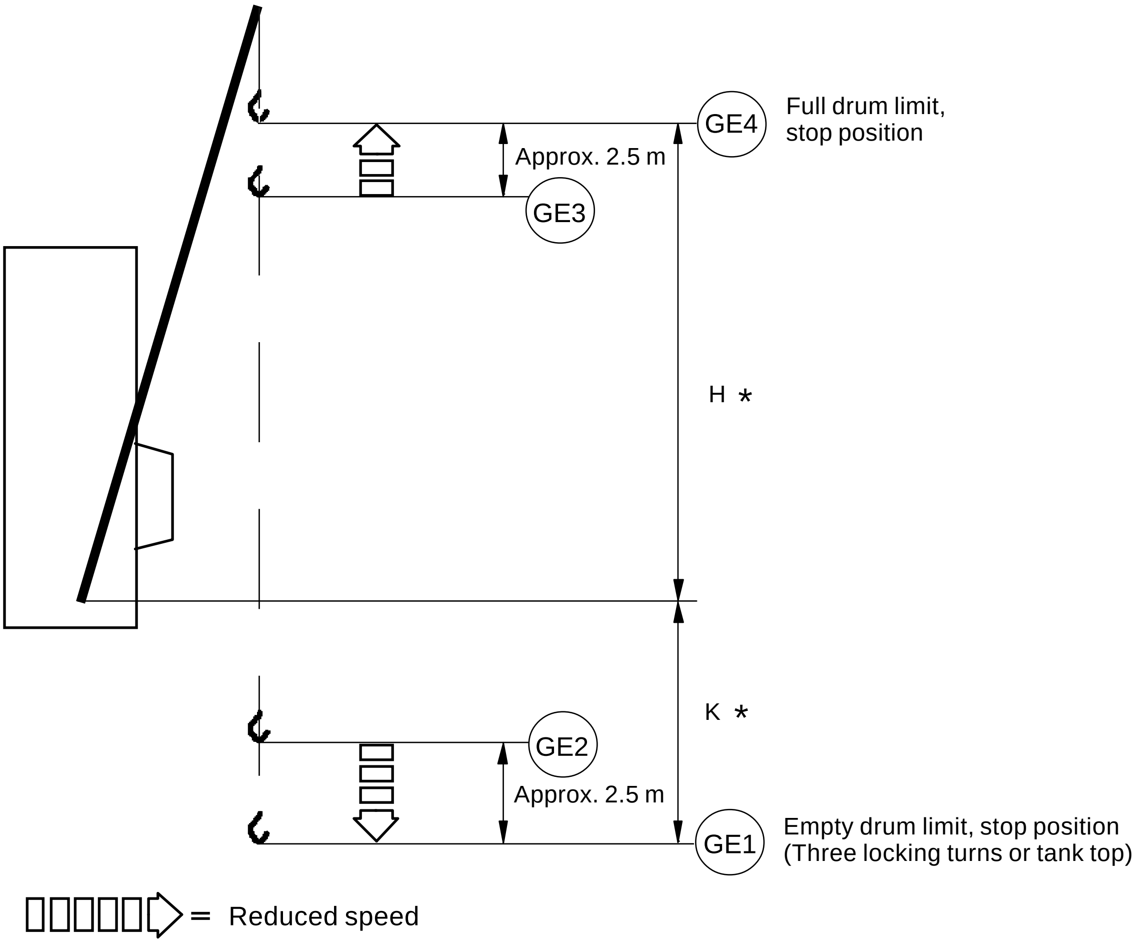

* Measures to be found in the document Technical Data, section 2.

Hoisting winch basic limit switches

Extra limit switches, high hook

This function prevents the jib from mechanical touching of the hook block when luffing out.

Limit switch GE5 (speed reduce switch, hoisting) takes care of the area [hatched area]

Limit switch GE6 (detector switch) takes care of the area [cross-hatched area] and gives signal to GD7 (stop cam, luffing) if the hook block is in the wrong area.

There is no stop function on the hoisting winch.

Speed reduce, luffing out

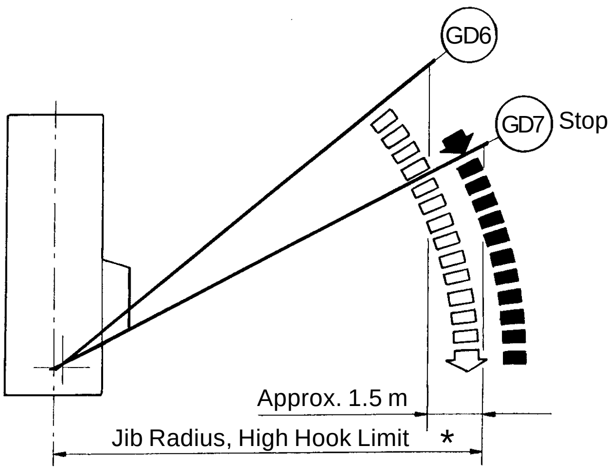

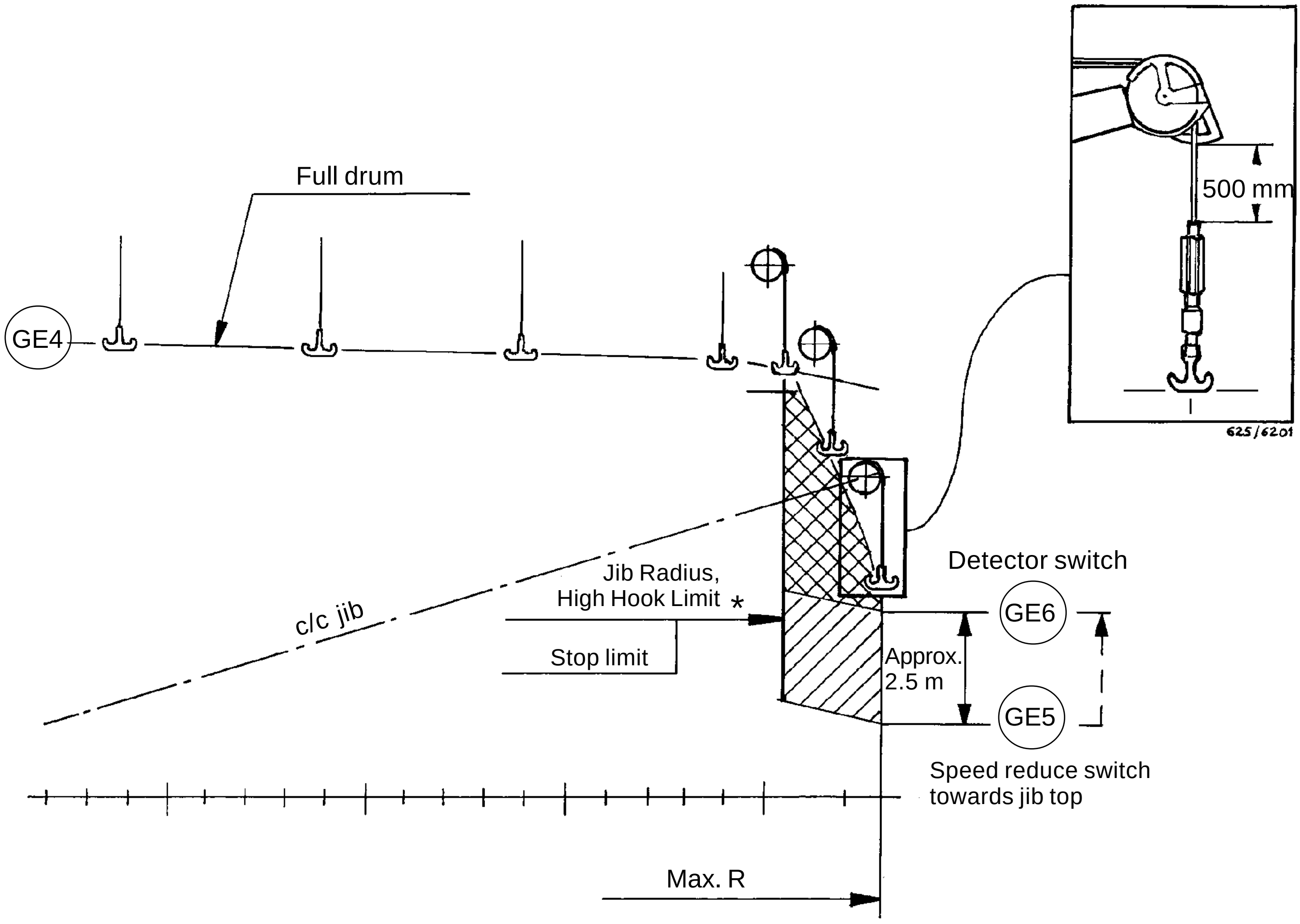

* Measures to be found in the document Technical Data, section 2.

Slack Wire Safety Switch Hoisting and Luffing Winches

Function

A slacking luffing or hoisting wire rope shall stop the respective winch movement before the spooling of the rope on the drum has been damaged. The adjoining figs. show the arrangement of the wire sensing device with built-in switch. When the wire slackens, the spring-loaded pressure roll forces the wire towards the wire drum, at the same time swinging the arm to actuate the built-in switch. Furthermore the hydraulic motor valve and the pump return to zero, and the winch stops.
The slack wire function is approved as long as the wire is spooling properly on the drum.

Functional test, hoisting

Lower the hook on to the deck to slacken the hoisting wire rope. Hoist the hook in order to check that the switch returns properly.

Functional test, luffing

Run the hoisting wire block/swivel slowly against the jib top and lift the jib, to cause the luffing wire to slacken. The hoisting winch shall then stop. Some cranes are equipped with safety switches to prevent the block from touching the jib. This safety switch must be made inoperative. Lower the hoisting wire block/swivel so that the luffing wire rope is taut. Check that the switch returns properly.

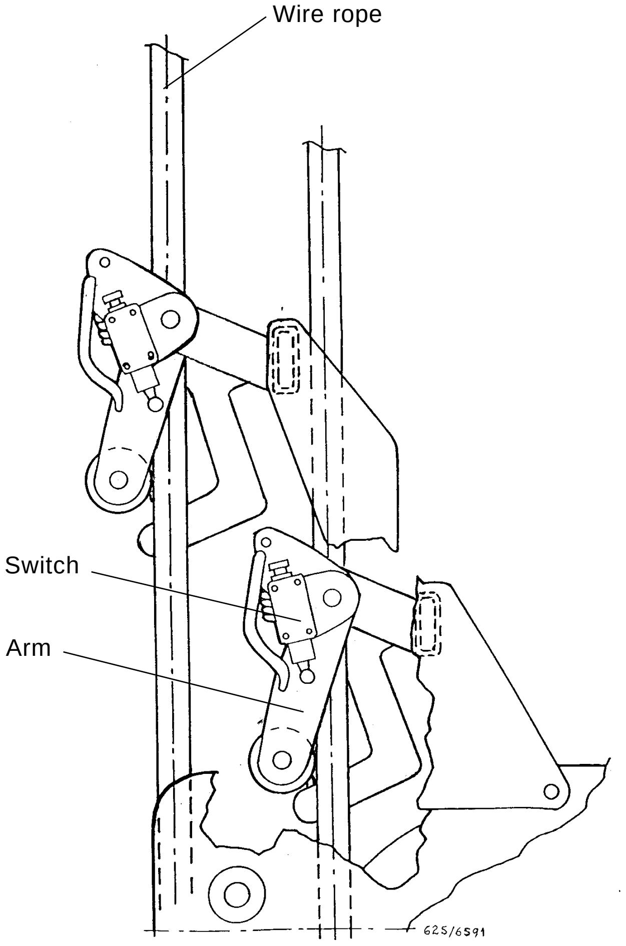

Slack wire safety switch. Normal Condition.

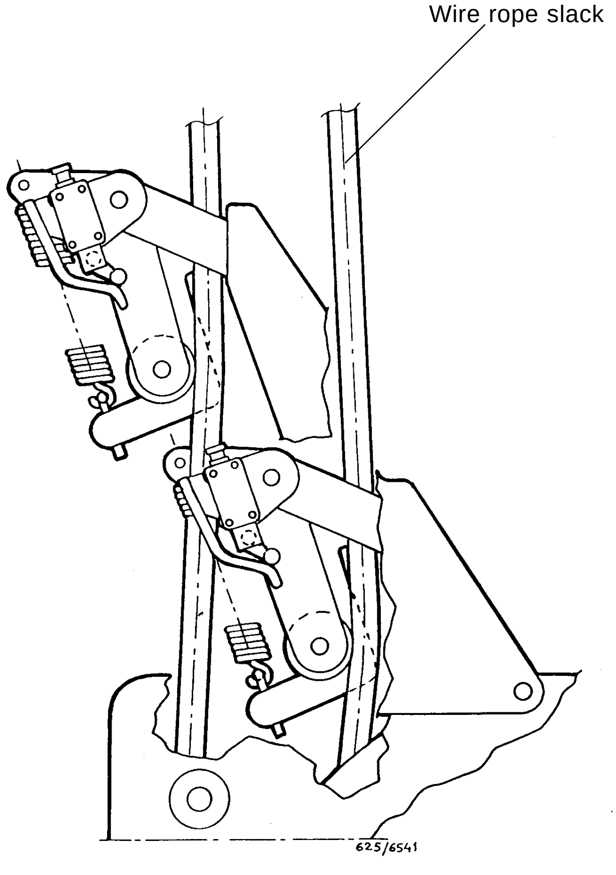

Slack wire safety switch. Slack wire condition.

Emergency Release of Parking Lock in Case of Power Failure, by Use of a Hand Pump (Optional)

General

In case of a power failure the parking lock may still be operated.
The release of parking lock is facilitated by use of the excisting hand pump on board and by installing a replacement set for this purpose (288 1632-801)

Preparation

- Install the replacement set. See Fig. 1.
- Connect the hand pump to the connection between valves 6731 and 6751 as well as between brake 3211 and valve 3661. See Figs. 1 and 3.
- Shut valve 3661.

Operation

- Push valve 6731 to "Unlock" position by use of a screw driver. See Fig. 2 and 3.
- Use the hand pump to release the cylinders 6711 and 6712. Check by sight that the cylinders are lifted up with the bolts.
- Now it is possible to slew the crane (jib) to parking position.
- Now the bolts can be lowered by opening the release valve on the hand pump.
- Open valve 3661 after finished slewing.

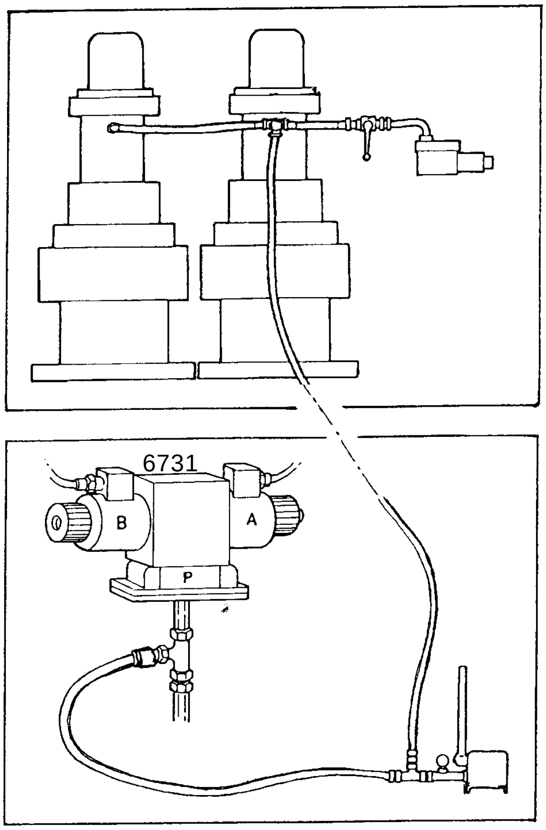

Fig. 1. Installing replacement set

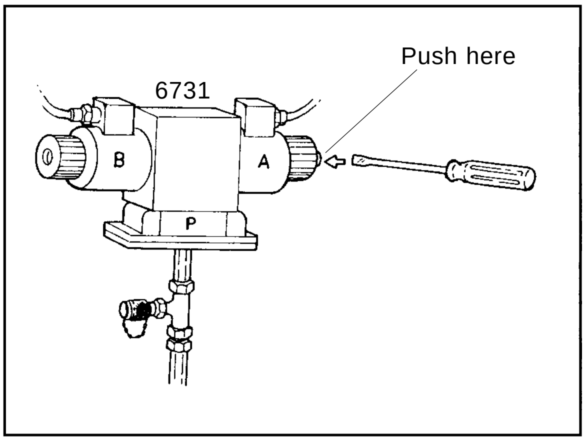

Fig. 2. Connection of hand pump

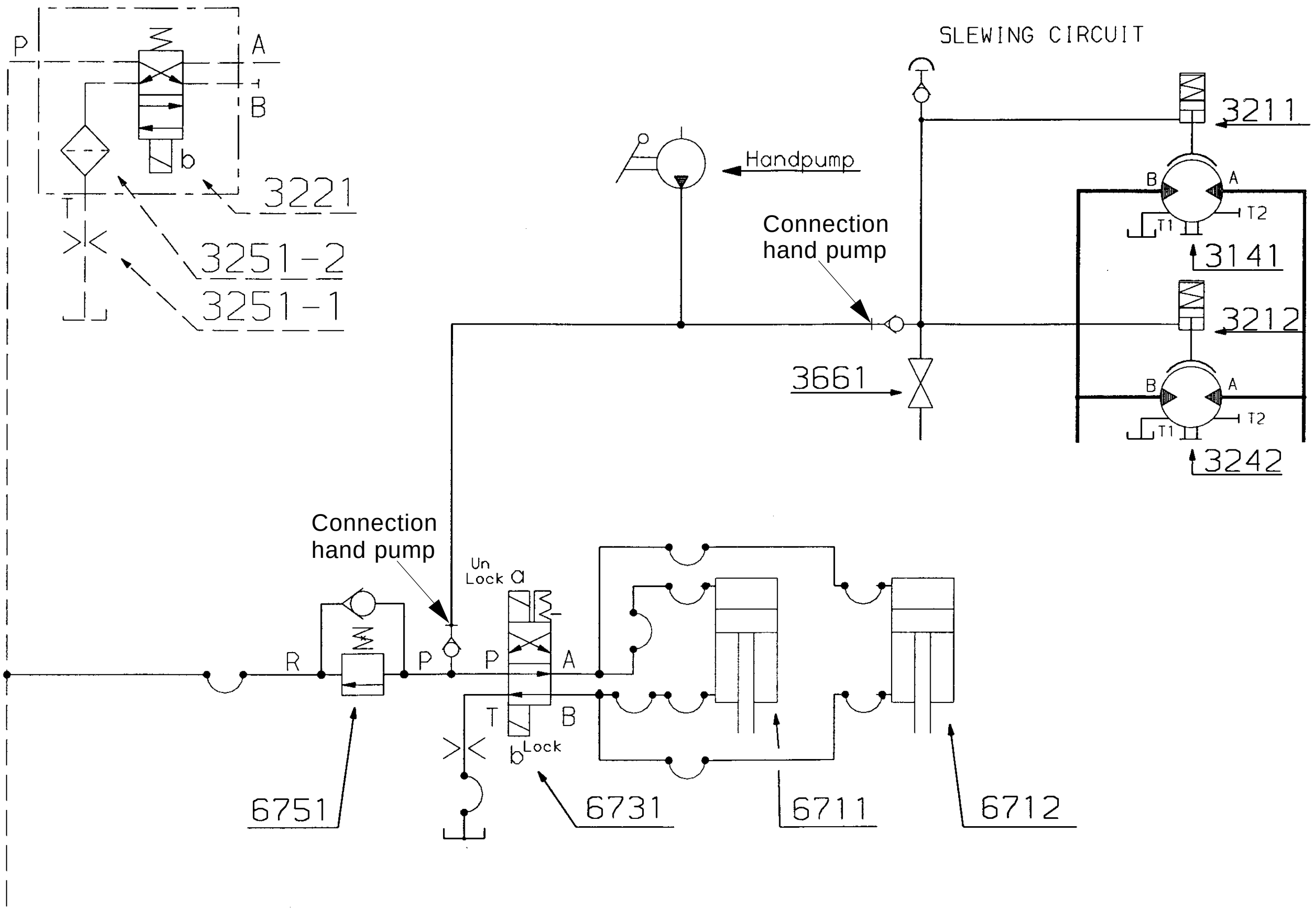

Fig. 3. Parking and slewing circuit diagram

Emergency Brake Release in Case of Complete Power Failure Operation of Crane Using a Hand Pump (Optional)

Introduction

In the case of a power failure the hoisting, luffing and slewing circuits of a deck crane may still be operated to a limited extent by releasing their respective brakes, while observing the greatest care.

The release of the brakes of the hoisting, luffing, and slewing functions is facilitated by employing a hand pump designed for this purpose, a quick coupling and a shut-off valve is also installed.

Twin operation

When the cranes are to be used in twin operation the brakes must be released by using two hand pumps, one for each crane. The necessary crew for this operation is; two "brake releasers" and one crane operator.

Contact between the two "brake releasers" and the crane operator is absolutely essential, e.g. by use of head-sets.

All necessary material for this emergency equipment including the hydraulic pressure source is included the supply. See Fig. 1.

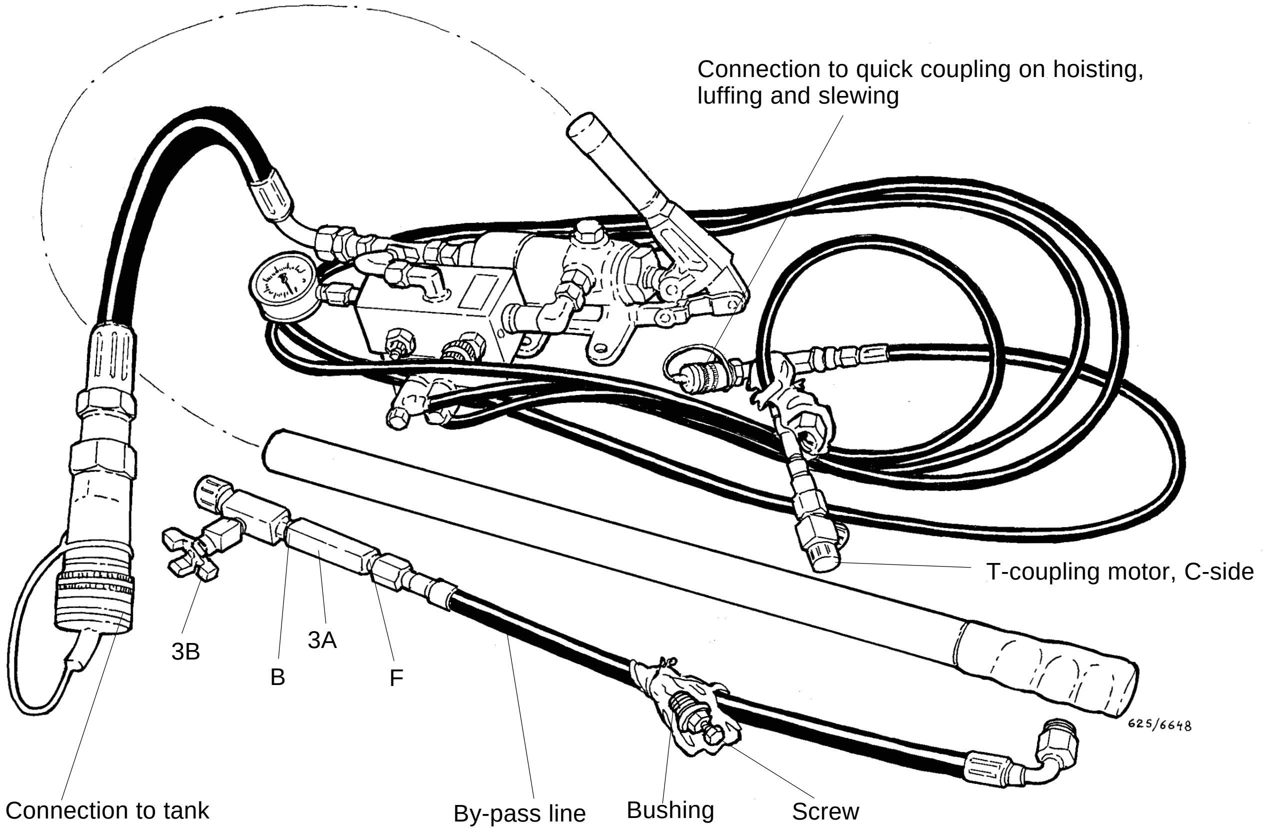

Fig. 1. Hand pump (optional)

CAUTION!

- This work has to be done with **great caution by skilled** persons.
- Only one function to be activated at the time.
- Verify that no persons are within the working area of the crane.
- Valves 1663, 2661 and 3661 **must not** be actuated except in emergency brake release operation.

Hoisting circuit, see Fig. 4 and 5

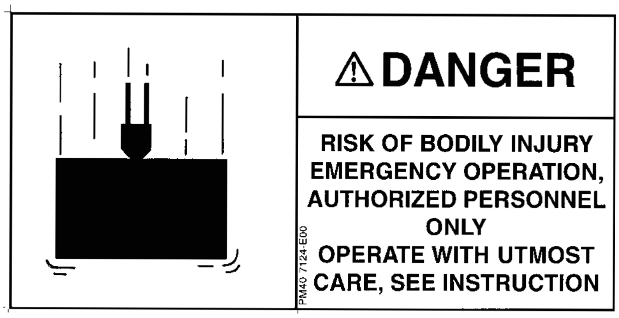

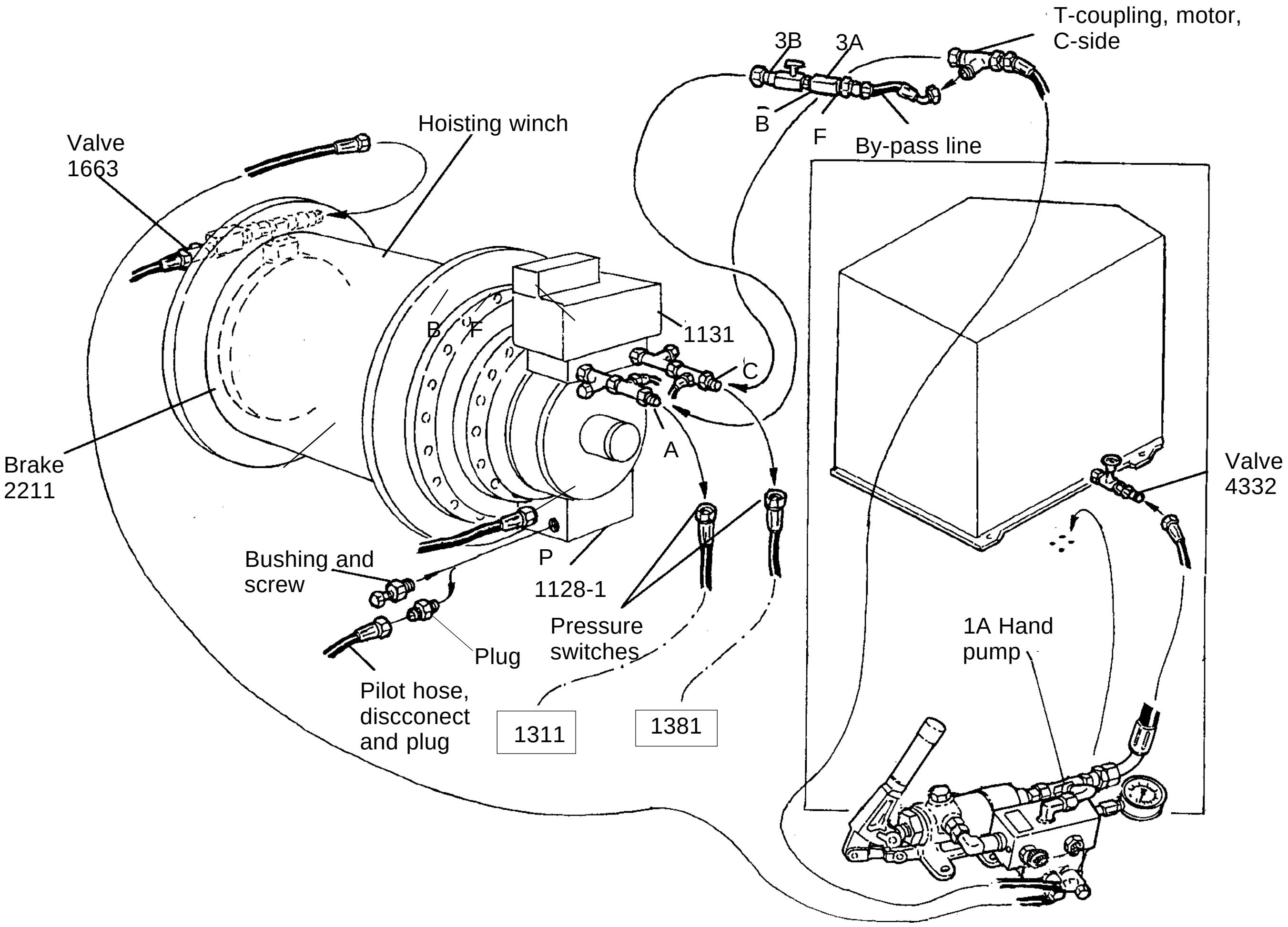

Fig. 2. Connection of hand pump, hoisting winch.

Connection

- Install the hand pump to the base plate by use of screws provided. See Fig. 2.
- Disconnect the pressure switches from valve 1131 A and C. See Fig. 2 and 5.
- Connect the motor by-pass line. See Fig. 2 and 4.
- Connect the hoses from the hand pump as shown in Fig. 2 and in the hydraulic diagram Fig 4.
- Disconnect the pilot hose from connection “P”, valve 1128-1.
  Note! The spacer ring can fall out when disassembling. Plug the disconnected hose. See Fig. 2 and 4.
- Attach the bushing and screw into connection “P”, valve 1128-1. Screw home the screw to actuate the piston to blocked position. See Fig. 2 and 3.
- Close valve 1663 and open valve 4332. See Fig. 2 and 4.

Operation

Release the brake by pumping oil from the hand pump. The load must be lowered slowly and under strict control.

Twin operation

The twin beam must be as horizontal as possible during lowering of the load winches.

Function

The emergency lowering system means that the motor hydraulically controls the load. Hand pump 1A puts pressure on the low side of the motor (C-side) and on the brake 1211 which opens at ~1.5 Mpa (1D). Oil from the high side of the motor (A-side) goes through needle valve 3B and volume control valve 3A to the C-side. Valve 3A is independent of the load. With a load on the cargo hook, valve 3B can be completely or partially closed to reduce the speed.

Cautious pumping can cause jerky lowering due to the pressure on the C-side of the motor dropping and the brake closing. The connection between the C-side and the brake is thus a very important safety function against uncontrolled lowering.

After operation

After the lowering operation is completed, reset the crane to “**NOT** EMERGENCY COUPLED“ and close valve 4332 and open valve 1663. See Fig. 2.

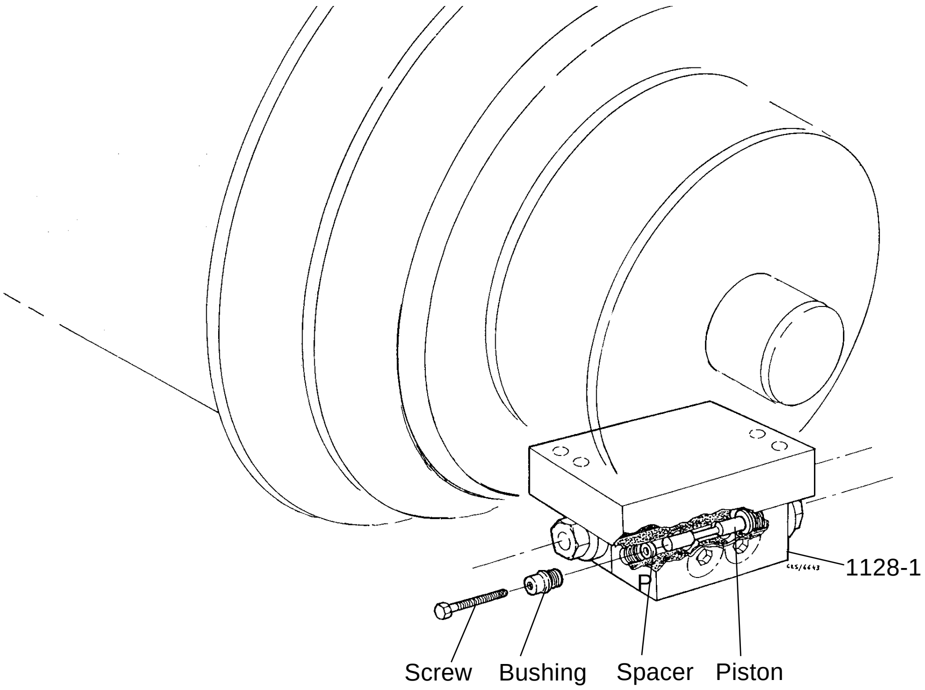

Fig. 3. Valve 1128

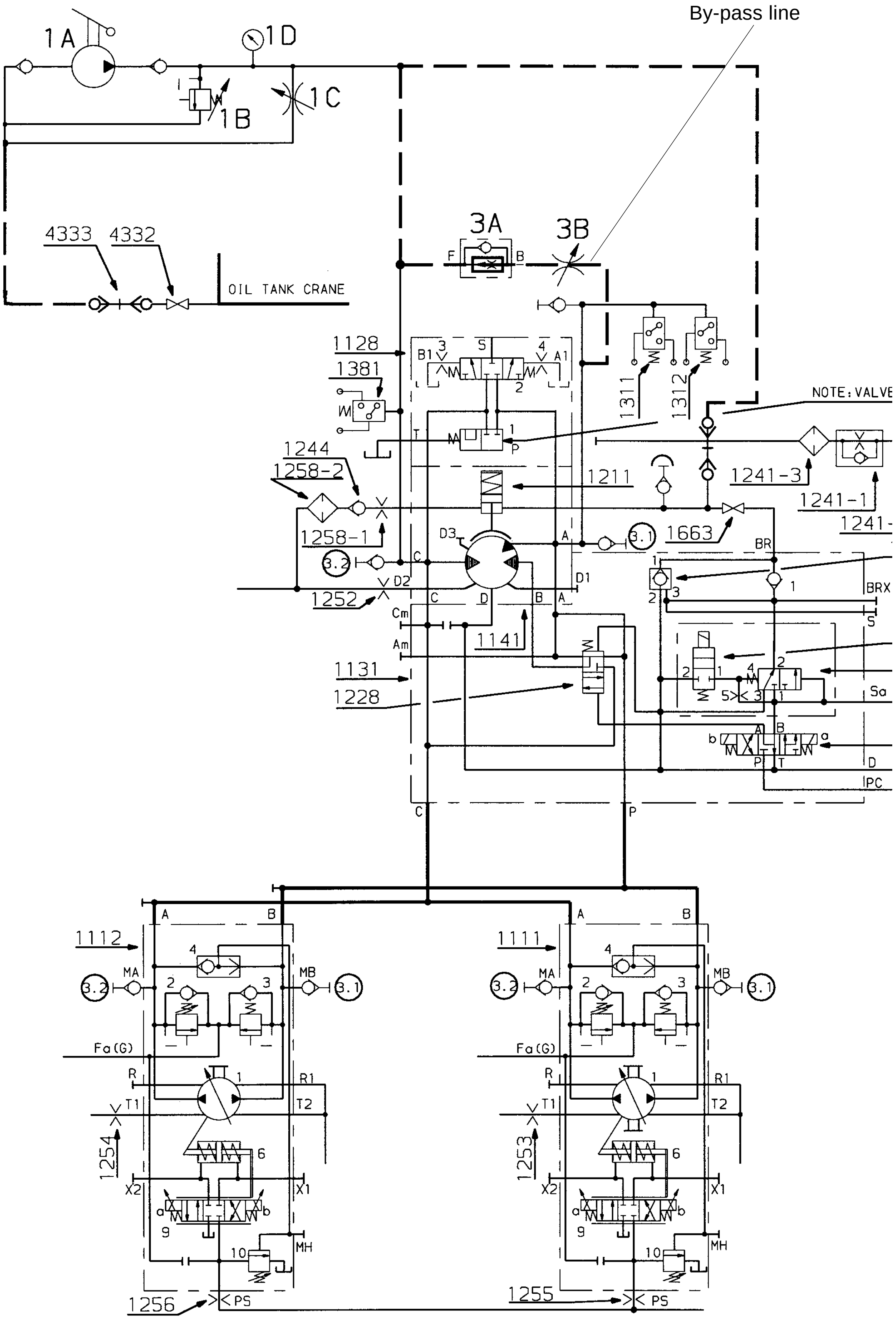

Fig. 4. Hydraulic circuit, hoisting. Emergency coupled.

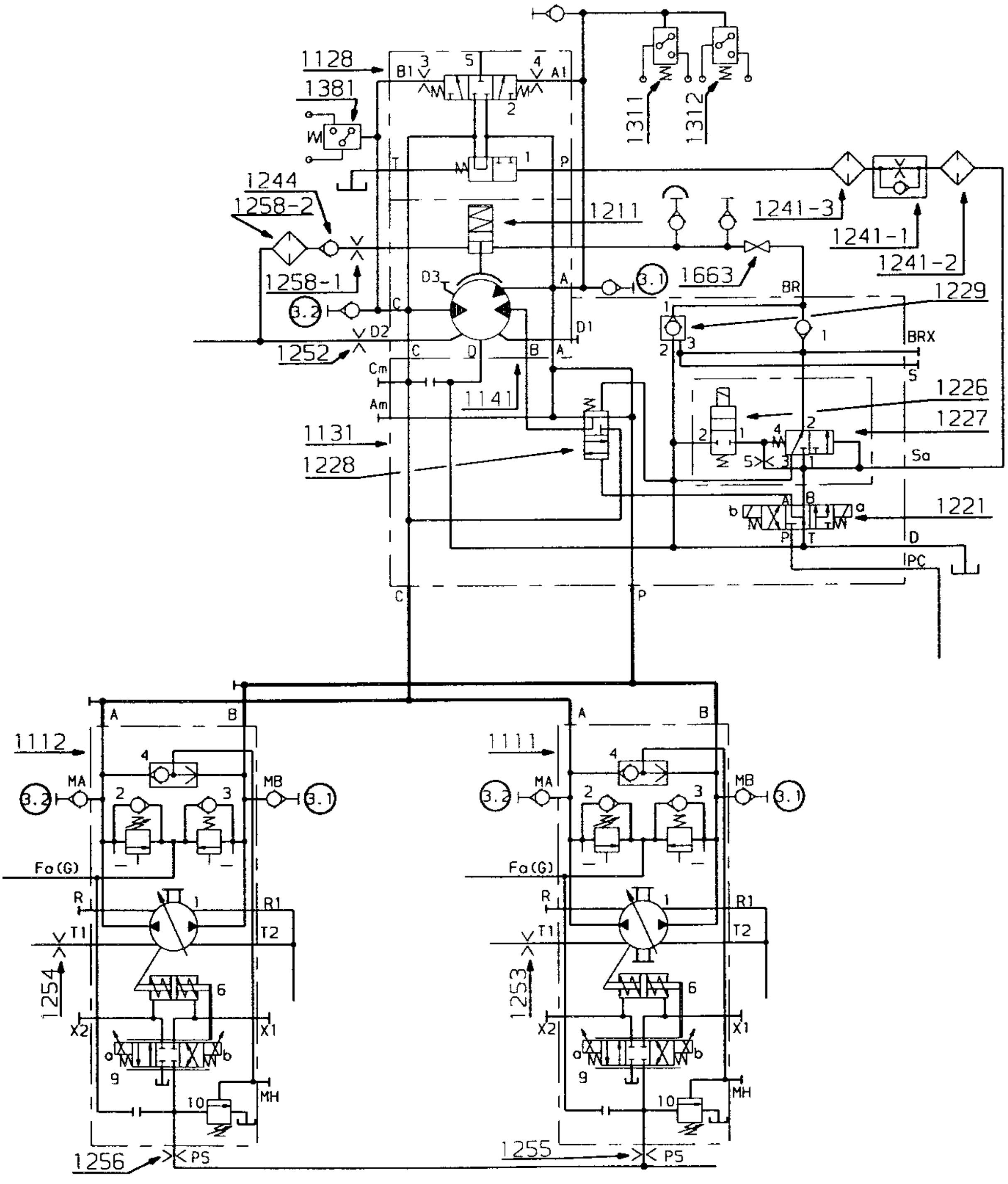

Fig. 5. Hydraulic circuit, hoisting. **NOT** emergency coupled.

Luffing circuit, see Fig. 8 and 9

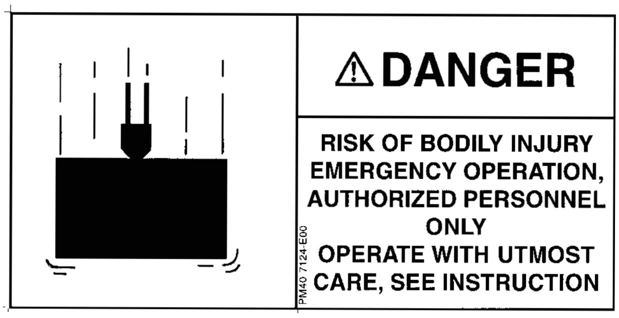

Connection

- Install the hand pump to the base plate with screws provided. See Fig. 6.
- Remove existing plugs from motor connections A and C. See Fig. 6.
- Connect the motor by-pass line. See Fig. 6 and 8.

Caution!
It is of most importance that the hoses to A and C are **NOT** switched, because the load could be dropped.

- Connect hoses from the hand pump as shown in Fig. 6 and hydraulic diagram Fig. 8.
- Disconnect the pilot hose from connection P, valve 2127.
  Note! The spacer can fall out when disassembling. Plug the disconnected hose. See Fig. 6 and 7.
- Attach bushing and screw into connection P, valve 2127. Screw home the screw to actuate the piston to blocked position. See Fig. 6 and 7.
- Close valve 2661 and open valve 4332. See Fig. 6 and 8.

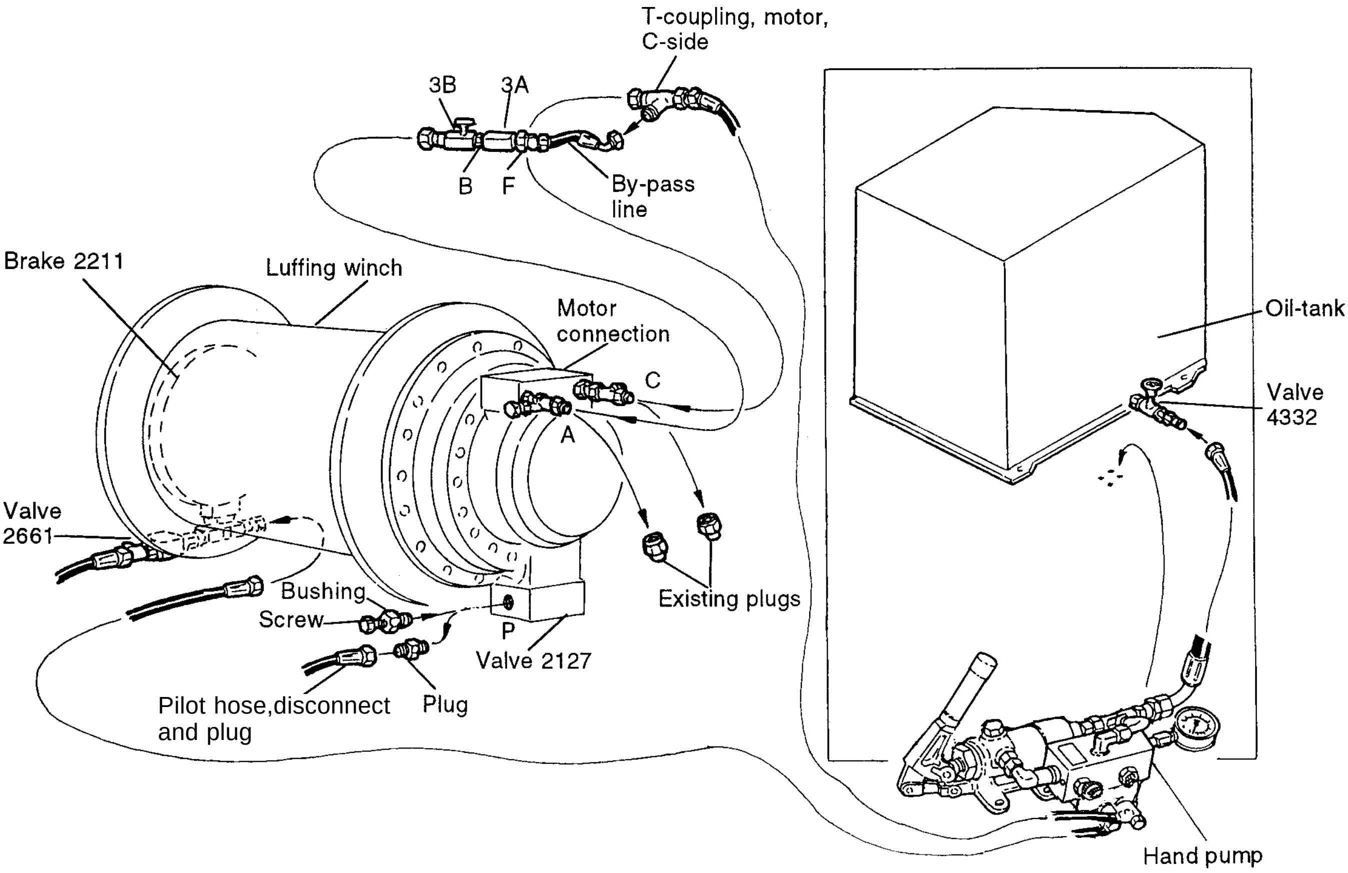

Fig. 6. Connection of hand pump, luffing winch

Operation

- Release the brake by pumping oil from the hand pump.
- The jib must be lowered slowly and under strict control.

Twin operation

The jibs must be as parallel as possible to each other during the lowering of the jibs.

If only the luffing circuit is out of operation, try to lower the load on deck or quay with the hoisting winch. Remove the twin beam and lower the jib one by one.

Function

The emergency lowering system means that the motor hydraulically controls the jib. Hand pump 1A puts pressure on the low side of the motor (C-side) and on the brake 2211 which opens at ~1.5 Mpa (1D). Oil from the high pressure side of the motor (A-side) goes through needle valve 3B and volume control valve 3A to the C-side. Valve 3A is independent of the load. With a load on the cargo hook, valve 3B can be completely or partially closed to reduce the speed.

Cautious pumping can cause jerky lowering due to the pressure on the C-side of the motor dropping and the brake closing. The connection between the C-side and the brake is thus a very important safety function against uncontrolled lowering.

After operation

After the lowering operation is completed, reset the crane to "**NOT** EMERGENCY COUPLED," valve 4332 must be closed and valve 2661 must be open. See Fig. 6.

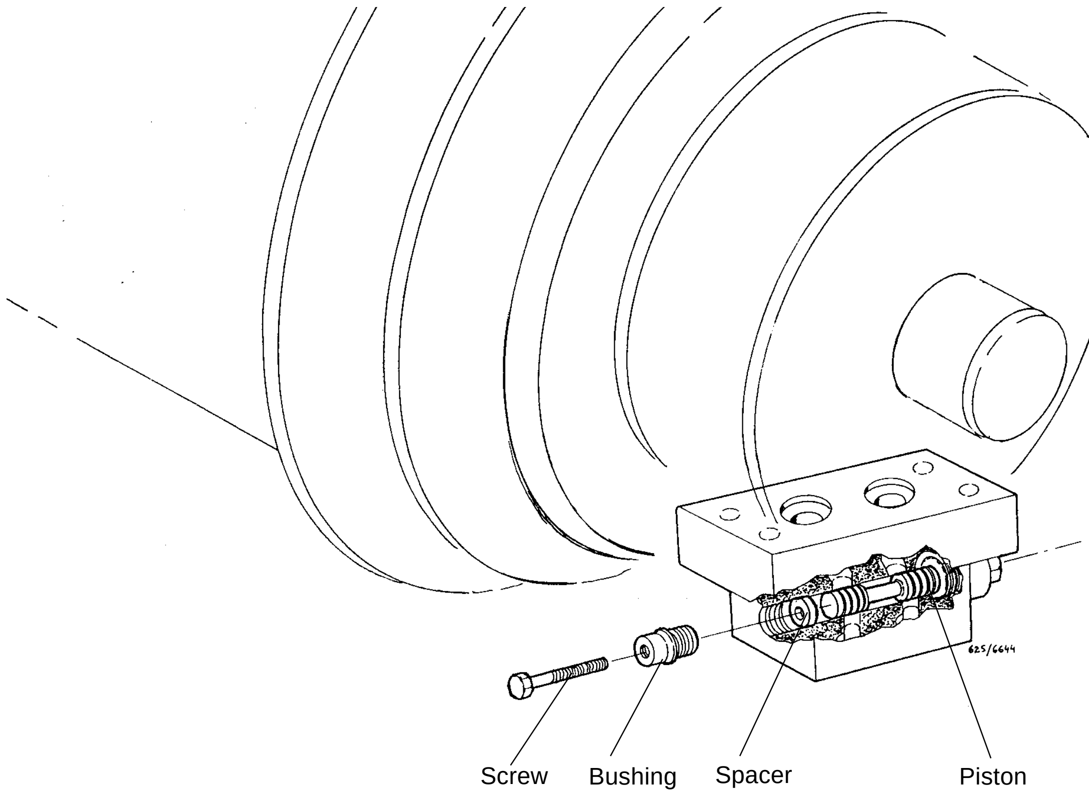

Fig. 7. Valve 2127.

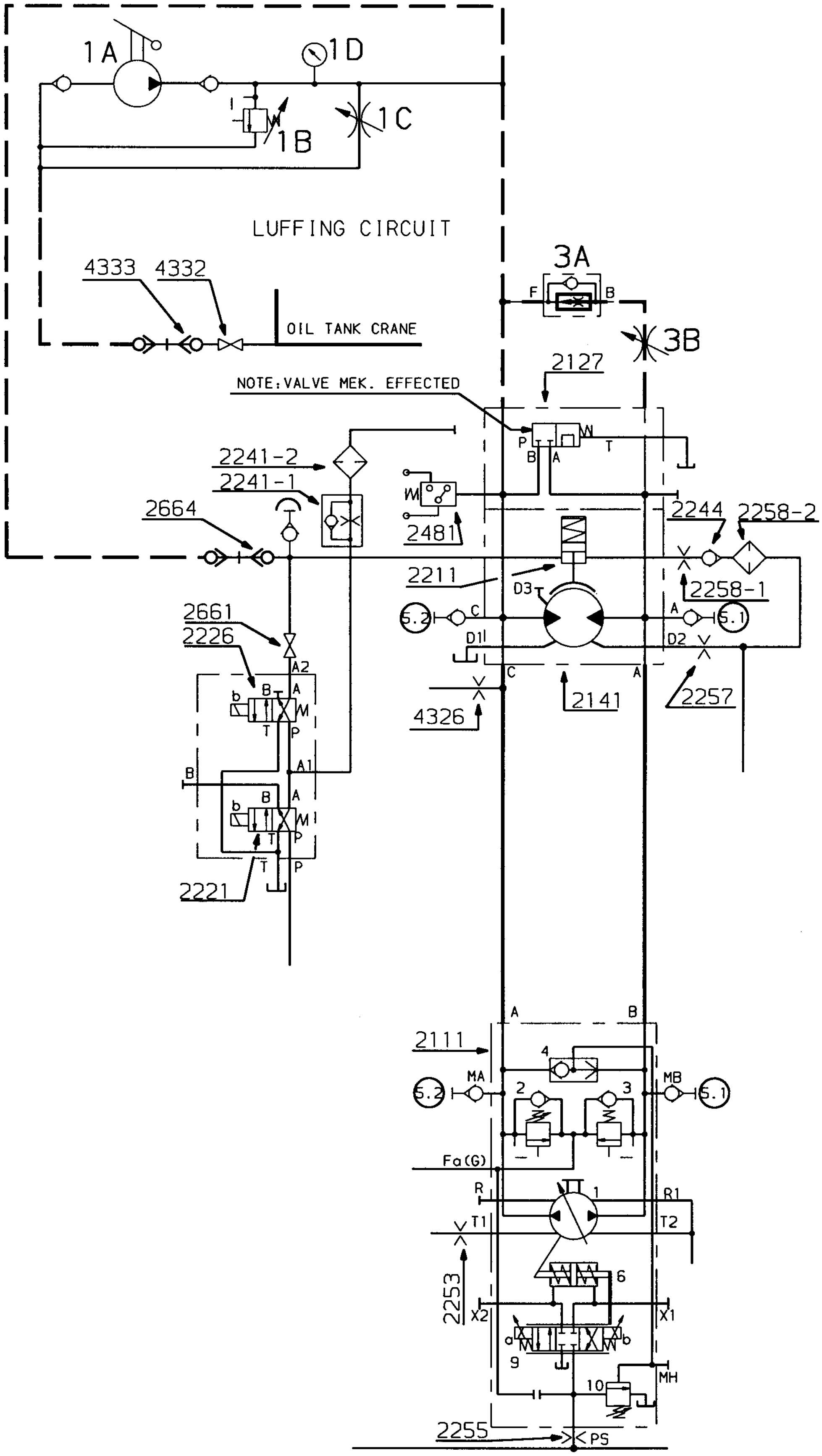

Fig. 8. Hydraulic circuit, luffing. Emergency coupled.

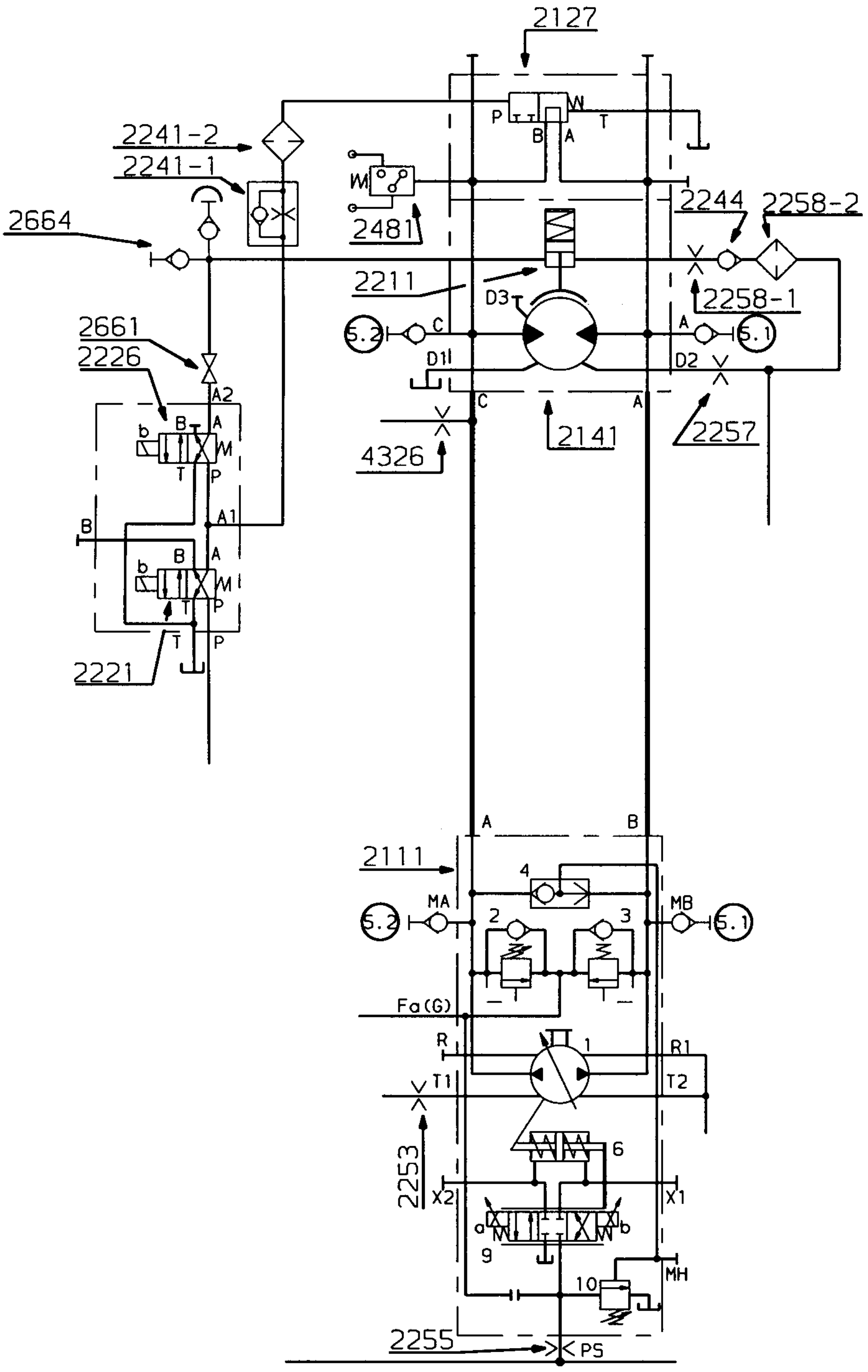

Fig. 9. Hydraulic circuit, luffing. **NOT** emergency copuled.

Slewing circuit, see Fig. 11 and 12

Connection

- Mount the hand pump to the base plate with the screws provided. See Fig. 10.
- Connect the hoses from the hand pump as shown in Fig. 10 and hydraulic diagram Fig. 12.
- Close valve 3661 and open valve 4332. See Fig. 10.

Operation

- Release the brake by pumping oil from the hand pump.
- The crane may now be slewed to a set position with the help of another crane. Use caution.

Note:

The brake opens at approximately 13 bar.

Warning!

If the crane is tilted, the brake must be released with great caution. If the speed tends to accelerate too much, the brake can be activated by stopping the pumping or opening the valve 3661.

After operation

After the slewing operation is completed reset the crane to **"NOT** EMERGENCY COUPLED". Valve 4332 must be closed and valve 3661 must be opened. See Fig. 10.

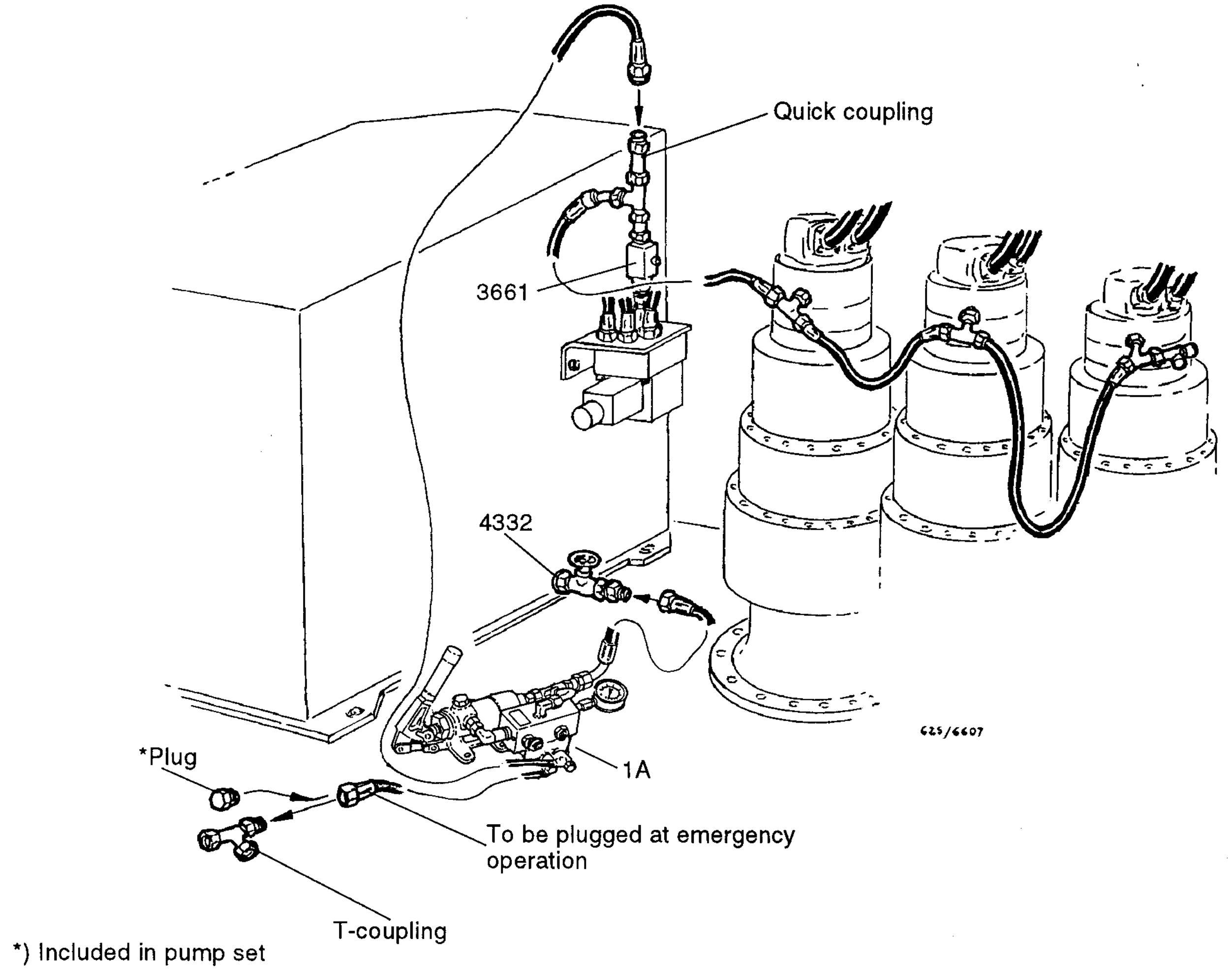

Fig. 10. Hand pump connected to slewing machinery

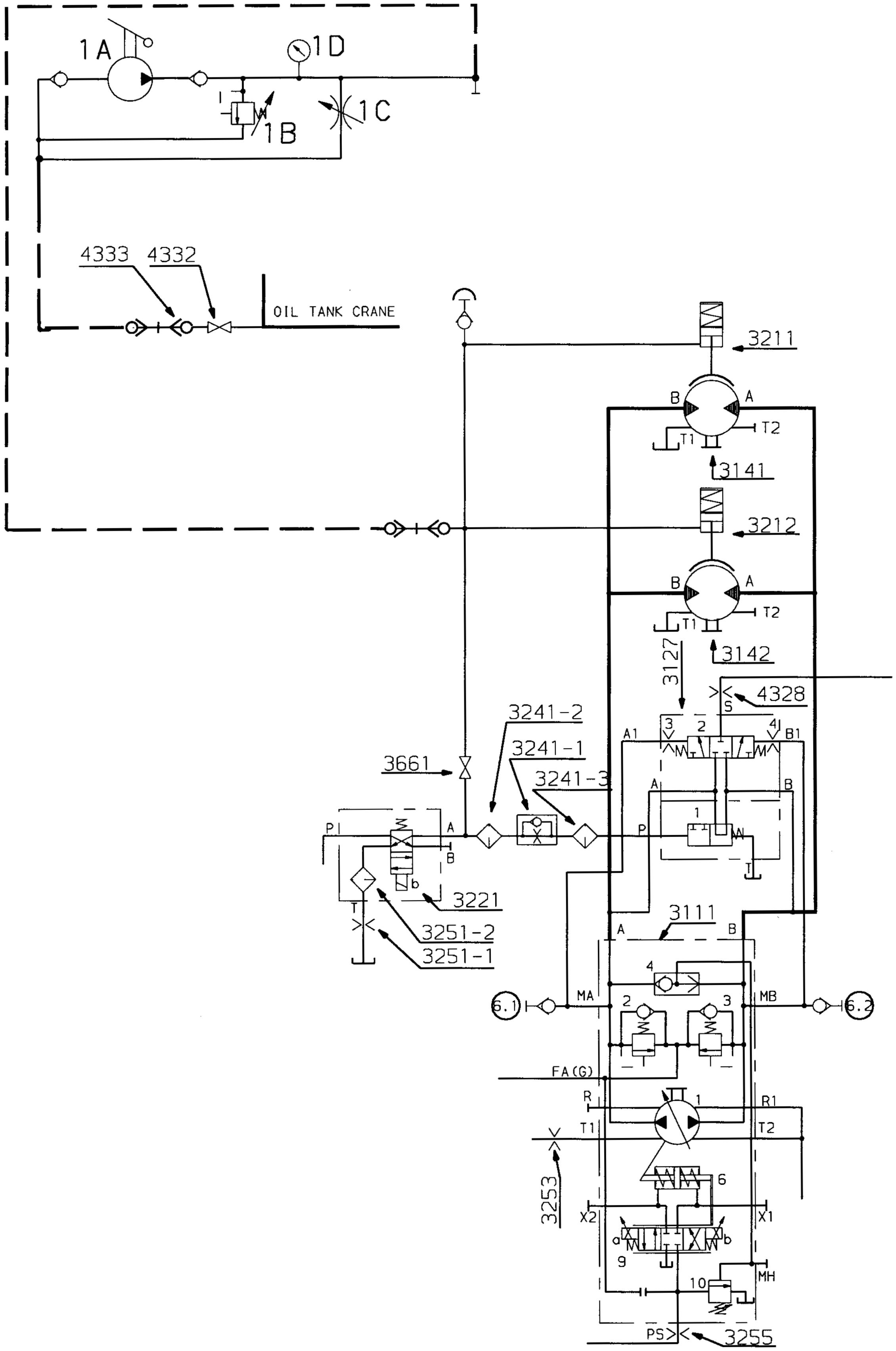

Fig. 11. Hydraulic circuit, slewing. Emergency copuled.

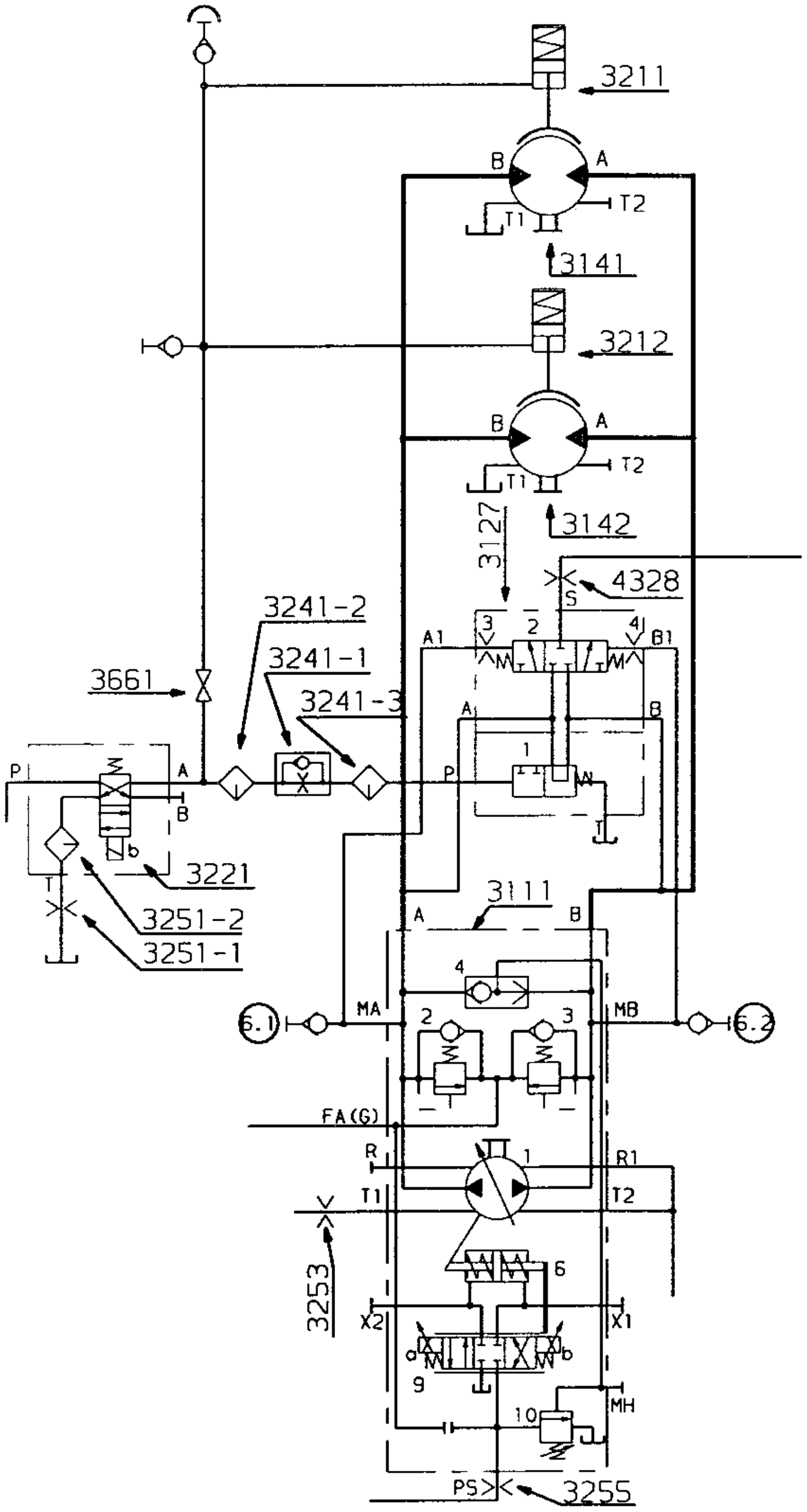

Fig. 12. Hydraulic circuit, slewing. **NOT** emergency copuled.

E 625-0014

MacGREGOR

Parts Manual

9.0 How to order spare parts

9.1 Machinery

9.2 Pump unit Oil cooler

9.3 Mechanical equipment

9.4 Hydraulic components

9.5 Electric components

9.6

9.7 Extra equipment

Address:
MacGREGOR Cranes AB
S-891 85 ÖRNSKÖLDSVIK, Sweden
Telephone: 46 - 660 - 29 40 00
Telex: 6050 haegg s
Telefax: 46 - 660 - 29 42 88 (Service Dept.)
Telefax: 46 - 660 -139 77 (Spare Part Dept.)
Telefax: 46 - 660 - 29 42 91 (Technical Info. Dept.)

E 1999-06-14

The Original Manufacturer of HÄGGLUNDS Cranes

How to Order Spare Parts

Important details when enquiring or ordering spare parts

To speed up the process we request you to provide us with below information:

- ☐ State name of company as well as your name
- ☐ Vessel's name and IMO number (and/or Lloyd's number)
- ☐ Crane type (see manual or name plate in operator's cabin)
- ☐ Crane place on the vessel. No. 1 counted from the fore
- ☐ Crane serial number (see manual or name plate in operator's cabin)
- ☐ Spare part figure or drawing number. To be found in section 9 in the manual
- ☐ Part description
- ☐ Item number from spare part figure or drawing respectively
- ☐ Part number, if available, from spare part figure or drawing respectively
- ☐ Quantity required
- ☐ Deadline - when or where in the world do you need the parts?
- ☐ Delivery details such as: consignee as well as phone and fax numbers
- ☐ Terms of delivery: by DHL, air freight direct or consignment, by truck or shipping freight

The more you can provide us with above details the faster and more reliable replies and deliveries will be made.

Address:
MacGREGOR Cranes AB
S-891 85 ÖRNSKÖLDSVIK, Sweden
Telephone: 46 - 660 - 29 40 00
Telefax: 46 - 660 - 139 77

S-891 85 ÖRNSKÖLDSVIK SWEDEN
Tel: 46 - 660 - 29 40 00
Direct: Fax: 46 - 660 - 139 77

Date: ....

Spare parts/Service specification

Please use this form whenever assistance is needed.

Service request ☐

Confirmation of phone call/conv. ☐

From: .. .. Fax no: ....

.... Tlx no: ....

....

Re: M/V .... IMO /Lloyd's no.: ....

Crane type:... Serial no: ....

Crane place: .... Our ref.: ....

For following spares we would like to: ☐ have your quotation

☐ place order, deadline for arrival :..............................

Part no.	Qty	Description	Fig./Drwg.	Item
1.	.....	....	....	....
2.	.....	....	....	....
3.	.....	....	....	....
4.	.....	....	....	....
5.	.....	....	....	....

6. See enclosure ☐

Delivery/Agent's address: ....

....

....

....

Name/ Title/

HOISTING WINCH

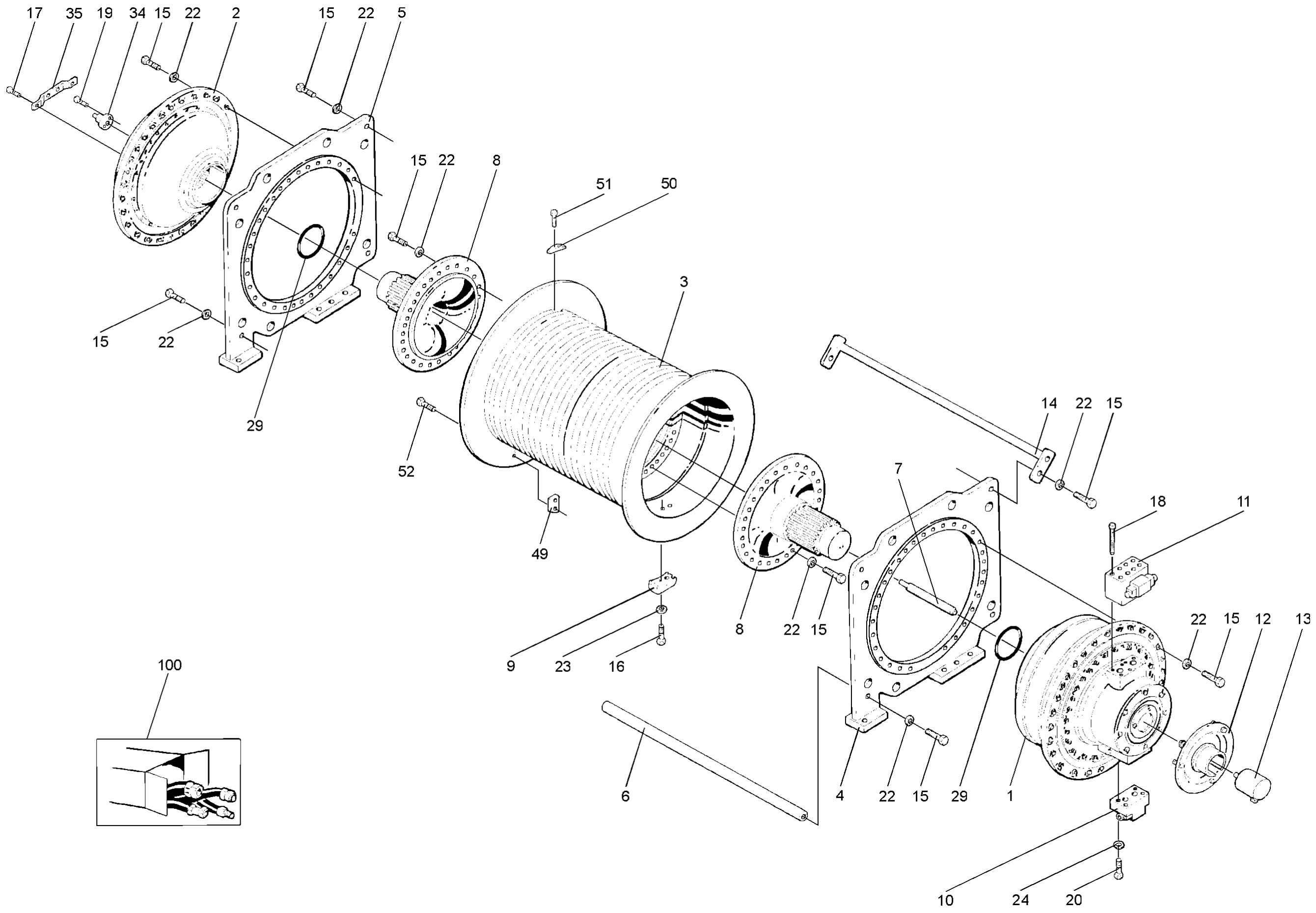

HOISTING WINCH

Item	Qty	Article no	Description	Supplementary data
000	1	188 0533-801	HOISTING WINCH	
001	1	875 10400-003	. HYDRAULIC MOTOR	CA 420-400; SEE FIG. 625-1420.008
002	1	875 10800-003	. MULTI DISC BRAKE	MDA 42; SEE FIG. 625-1430.006
003	1	178 2558-001	. HOISTING DRUM	
004	1	178 2599-801	. BRACKET MOTOR SIDE	
005	1	178 2601-801	. BRACKET BRAKE SIDE	
006	4	178 2419-001	. SUPPORT	
007	1	378 2260-001	. ADAPTER	
008	2	178 2567-001	. CENTRE SHAFT	
009	2	389 0188-006	. WIRE CLAMP	
010	1	875 11000-006	. FLUSH - UNLOADING UNIT	SEE FIG. 625-7802.008
011	1	875 11000-003	. TWO SPEED VALVE	SEE FIG. 625-7239.004
012	1	875 10600-002	. SPEED ENCODER, MOUNTNG	SMBA; SEE FIG. 625-1421.004
013	1	376 0052-801	. SPEED ENCODER	
014	1	378 2263-801	. PROTECTIVE TUBE	
015	122	2121 2037-678	. SCREW	M6S 20 x 70 -10.9
016	4	2121 2031-724	. SCREW	M6S 24 x 80 -8.8
017	2	2121 2032-532	. SCREW	M6S 12 x 20 -8.8 FZB
018	4	2121 2541-691	. SCREW	MC6S 20 x 140 -12.9
019	2	2121 2634-368	. SCREW	MFS 6 x 16 -5.8 FZB
020	4	2121 2561-381	. SCREW	UC6S 5/8" UNC x 51 -12.9
022	122	2151 2022-192	. WASHER	BRB 21 x 36 FZB; DIN 125A
023	4	2151 2022-198	. WASHER	BRB 25 x 45 FZB; DIN 125A
024	4	487 2826-002	. WASHER	

HOISTING WINCH

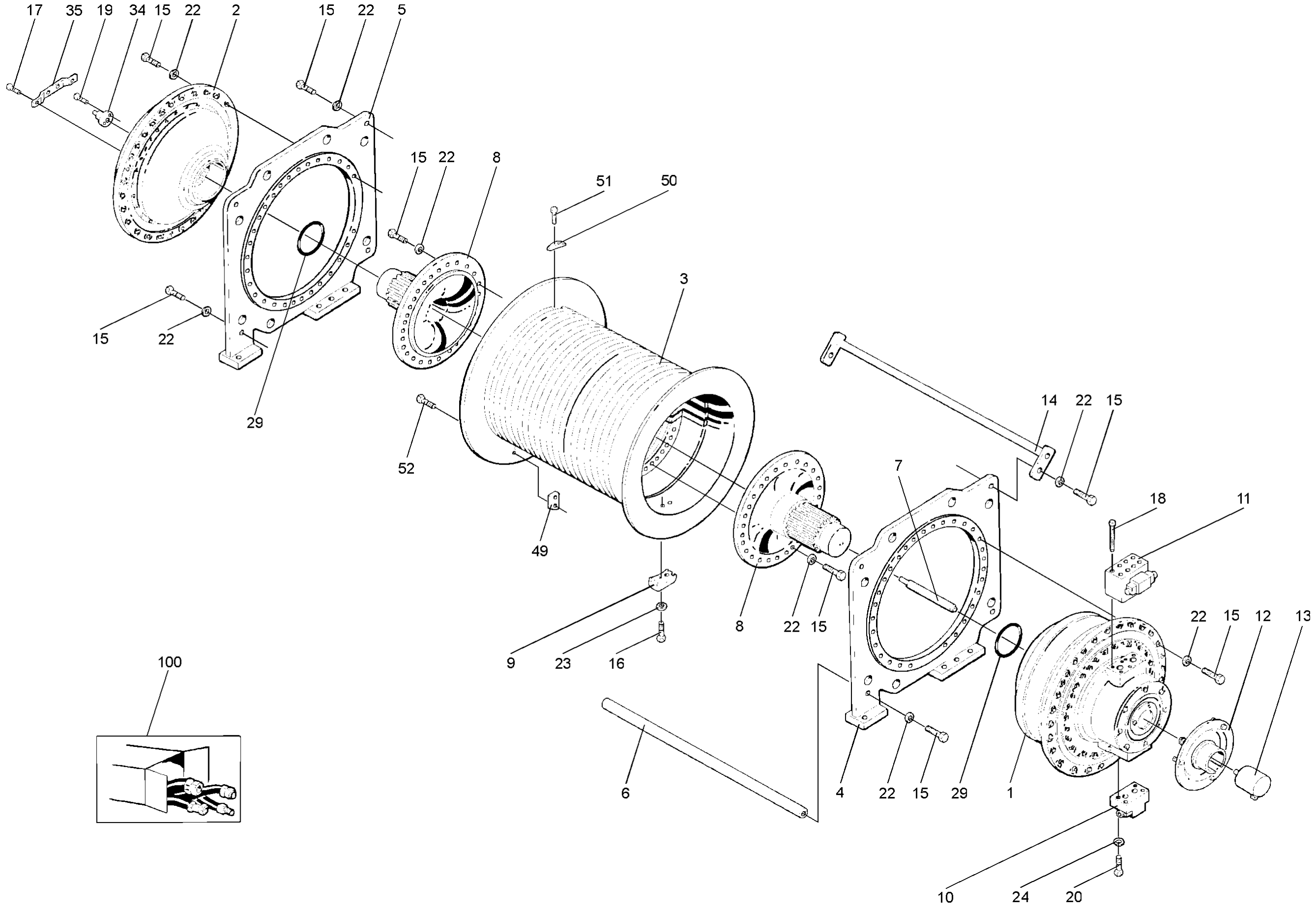

HOISTING WINCH

Item	Qty	Article no	Description	Supplementary data
029	2	2152 2115-541	. O-RING	
034	1	488 9974-001	. SHAFT END	
035	1	178 2642-001	. BRACKET	
049	1	378 2281-001	. KEY	
050	1	178 2613-001	. PITCH KEY	
051	2	2121 2532-453	. SCREW	MC6S 8 x 25 -8.8 FZB
052	2	2121 2032-461	. SCREW	M6S 8 x 50 -8.8 FZB
100	1	875 10200-001	. HYDRAULIC KIT, HOISTING WINCH	SEE FIG. 625-1411.004

HYDRAULIC KIT, HOISTING WINCH

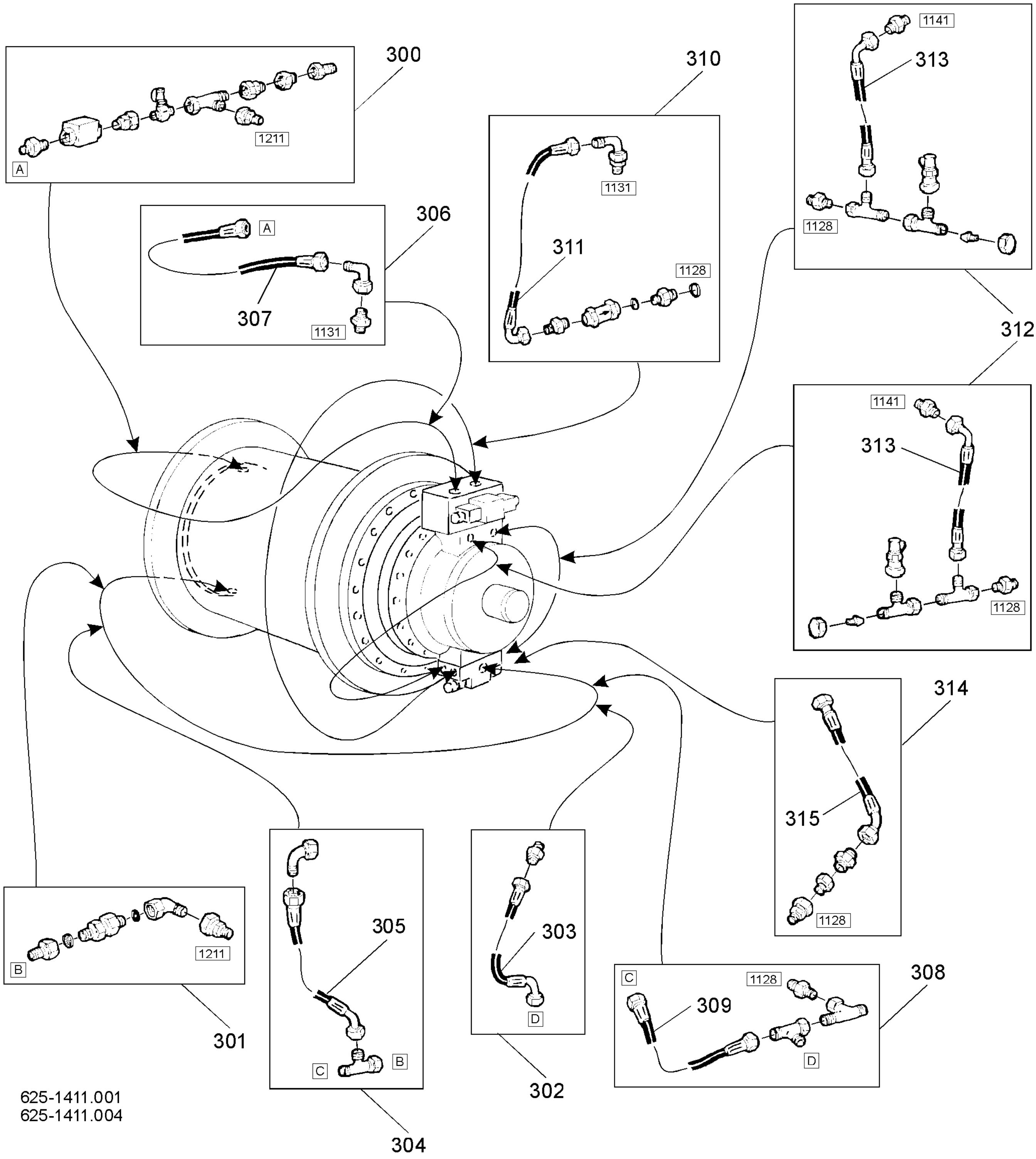

625-1411.001
625-1411.004

HYDRAULIC KIT, HOISTING WINCH

Item	Qty	Article no	Description	Supplementary data
000	1	478 3728-801	HYDRAULIC KIT, HOISTING WINCH	
300	1	178 2450-801	. ASS. KIT HOISTING BRAKE	
301	1	278 2122-801	. ASS. KIT HOISTING BRAKE	
302	1	178 2594-801	. HYDRAULIC HOSE, COMPLETE	
303	1	2515 6244-135	.. HYDRAULIC HOSE	
304	1	178 2618-801	. HYDRAULIC HOSE, COMPLETE	
305	1	2515 6144-095	.. HYDRAULIC HOSE	
306	1	178 2619-801	. HYDRAULIC HOSE, COMPLETE	
307	1	2515 6044-230	.. HYDRAULIC HOSE	
308	1	178 2620-801	. HYDRAULIC HOSE, COMPLETE	
309	1	2515 6044-220	.. HYDRAULIC HOSE	
310	1	178 2621-801	. HYDRAULIC HOSE, COMPLETE	
311	1	2515 6231-100	.. HYDRAULIC HOSE	
312	2	178 2622-801	. HYDRAULIC HOSE, COMPLETE	
313	2	2515 8131-085	.. HYDRAULIC HOSE	
314	1	178 2445-801	. HYDRAULIC HOSE, COMPLETE	
315	1	2515 6143-120	.. HYDRAULIC HOSE	

HYDRAULIC MOTOR

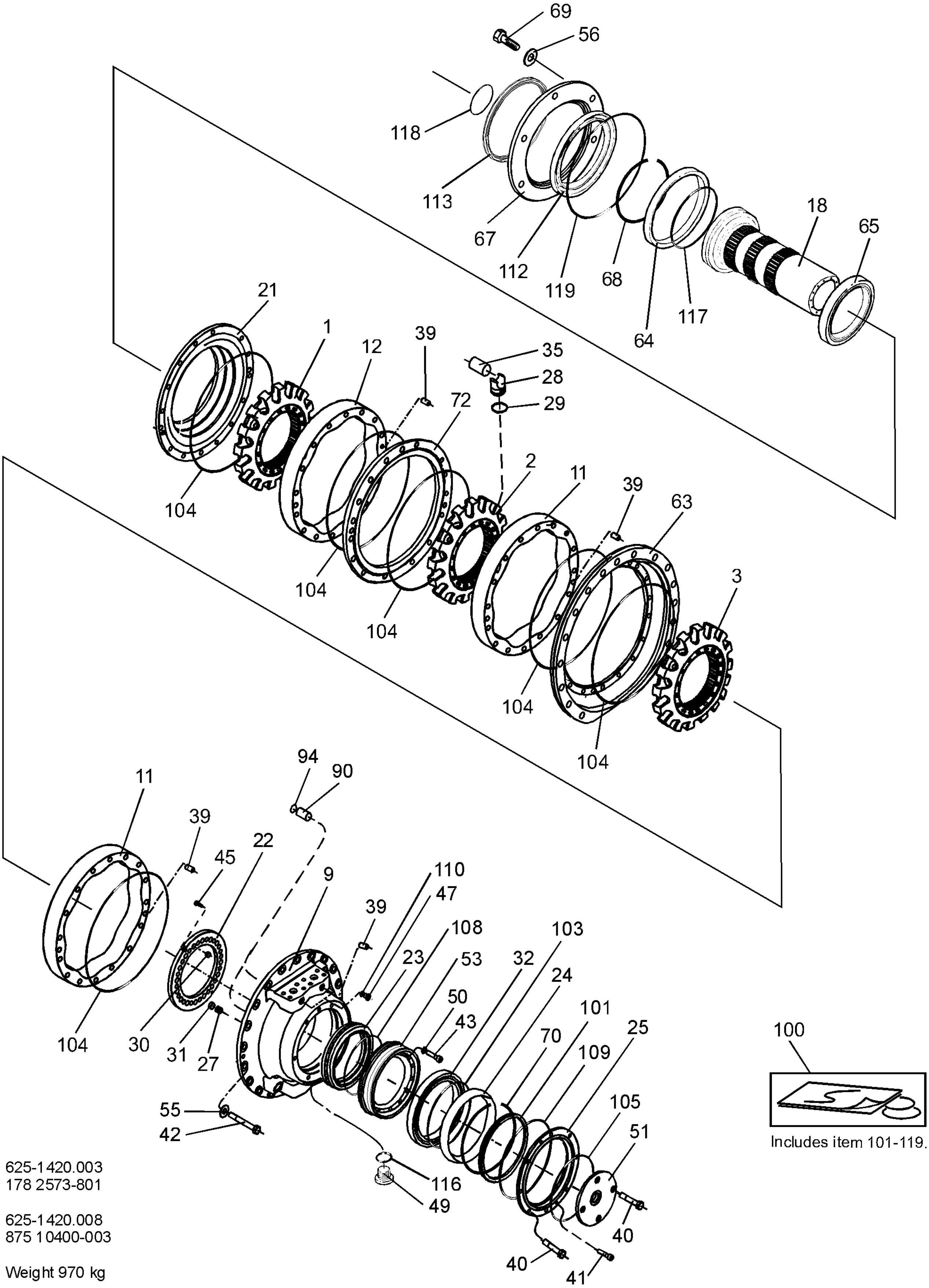

Includes item 101-119.

625-1420.003
178 2573-801

625-1420.008
875 10400-003

Weight 970 kg

HYDRAULIC MOTOR

Item	Qty	Article no	Description	Supplementary data
000	1	178 2573-801	HYDRAULIC MOTOR	CA 420-400
001	1	178 2485-004	. CYLINDER BLOCK, SHAFT END	
002	1	178 2485-003	. CYLINDER BLOCK, MIDDLE	
003	1	178 2485-001	. CYLINDER BLOCK, PORT END	
009	1	178 2500-004	. CONNECTION BLOCK	
011	2	178 2496-001	. CAM RING	140
012	1	178 2541-001	. CAM RING	120
018	1	178 2531-002	. CENTRE SHAFT	
021	1	178 5025-001	. SHAFT END HOUSING	
022	1	178 2487-001	. VALVE PLATE	
023	1	2213 2725-260	. CYL. ROLL. THRUST BEARING	
024	1	478 2003-001	. WEAR RING	
025	1	178 2556-001	. SEAL RETAINER, PORT SIDE	
027	20	478 3717-001	. COMPR. SPRING	
028	48	378 2242-001	. PISTON	
029	48	478 3721-005	. PISTON RING	
030	20	478 3718-001	. BALANCING PISTON	
031	20	378 2241-001	. BALANCING SLEEVE	
032	1	2213 2582-026	. CYL. ROLLER BEARING	
035	48	478 3722-001	. CAM ROLLER	
039	8	2111 2111-563	. ROLL PIN	FRP 16 x 40 SMS 1663
040	12	2121 2032-542	. SCREW	M6S 12 x 50 -8.8 FZB
041	2	2121 2532-532	. SCREW	MC6S 12 x 20 -8.8 FZB; DIN 912
042	20	2121 2040-701	. SCREW	M6S 20 x 330 -12.9

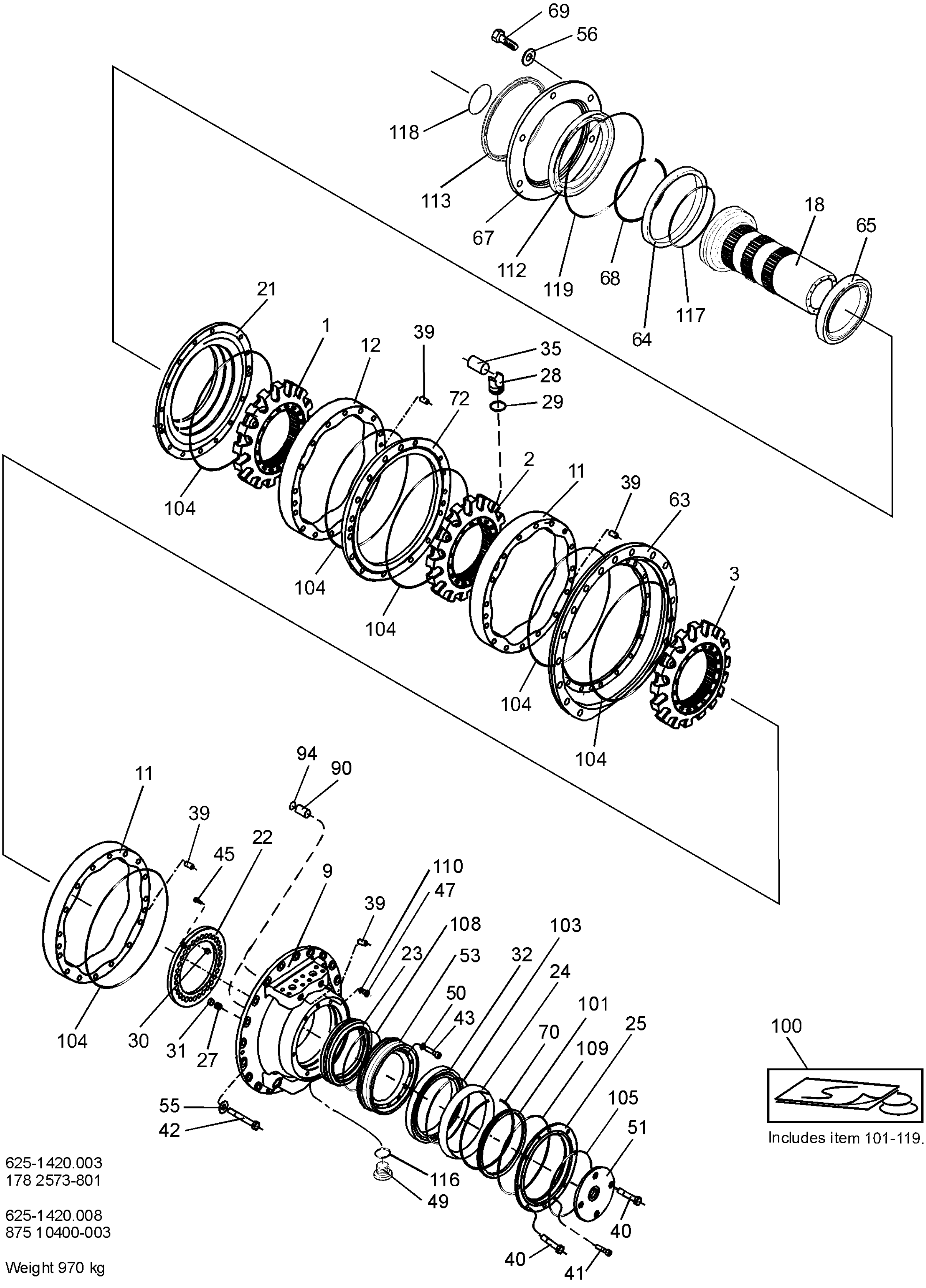

100

Includes item 101-119.

625-1420.003
178 2573-801

625-1420.008
875 10400-003

Weight 970 kg

HYDRAULIC MOTOR

Item	Qty	Article no	Description	Supplementary data
043	12	2121 2541-633	. SCREW	MC6S 16 x 65 -12.9
045	2	2121 2592-371	. LOCATING BOLT	MC6S 6 x 23
047	2	2522 2111-110	. PLUG	BSP 1/4" (Item 110 incl.)
049	1	2522 2111-116	. PLUG	R 1"
050	12	487 2826-002	. WASHER	
051	1	278 2118-001	. SHAFT COVER	
053	1	178 2540-001	. BEARING RETAINER	
055	20	487 2826-009	. WASHER	
056	6	2151 2022-173	. WASHER	BRB 10.5 x 22 FZB
063	1	178 2489-001	. ATTACHMENT RING	
064	1	378 2254-001	. WEAR RING	
065	1	378 2276-801	. CYL. ROLL. BEARING	
067	1	178 5026-001	. SEAL RETAINER, SHAFT SIDE	
068	1	478 3726-001	. LOCK RING	
069	6	2121 2032-505	. SCREW	M6S 10 x 70 -8.8 FZB
070	1	478 3725-001	. LOCK RING	
072	1	178 2491-001	. SPACER RING	
090	2	478 3729-001	. 2-SPEED PLUG	
094	2	2154 2431-126	. LOCK RING	
100	1	278 2155-803	. GASKET SET, NITRIL	Incl. item 101-119.
101	1	478 1200-001	.. RADIAL LIP SEAL	
103	1	2152 2115-944	.. O-RING	
104	6	2152 2115-894	.. O-RING	
105	1	2152 2115-531	.. O-RING	

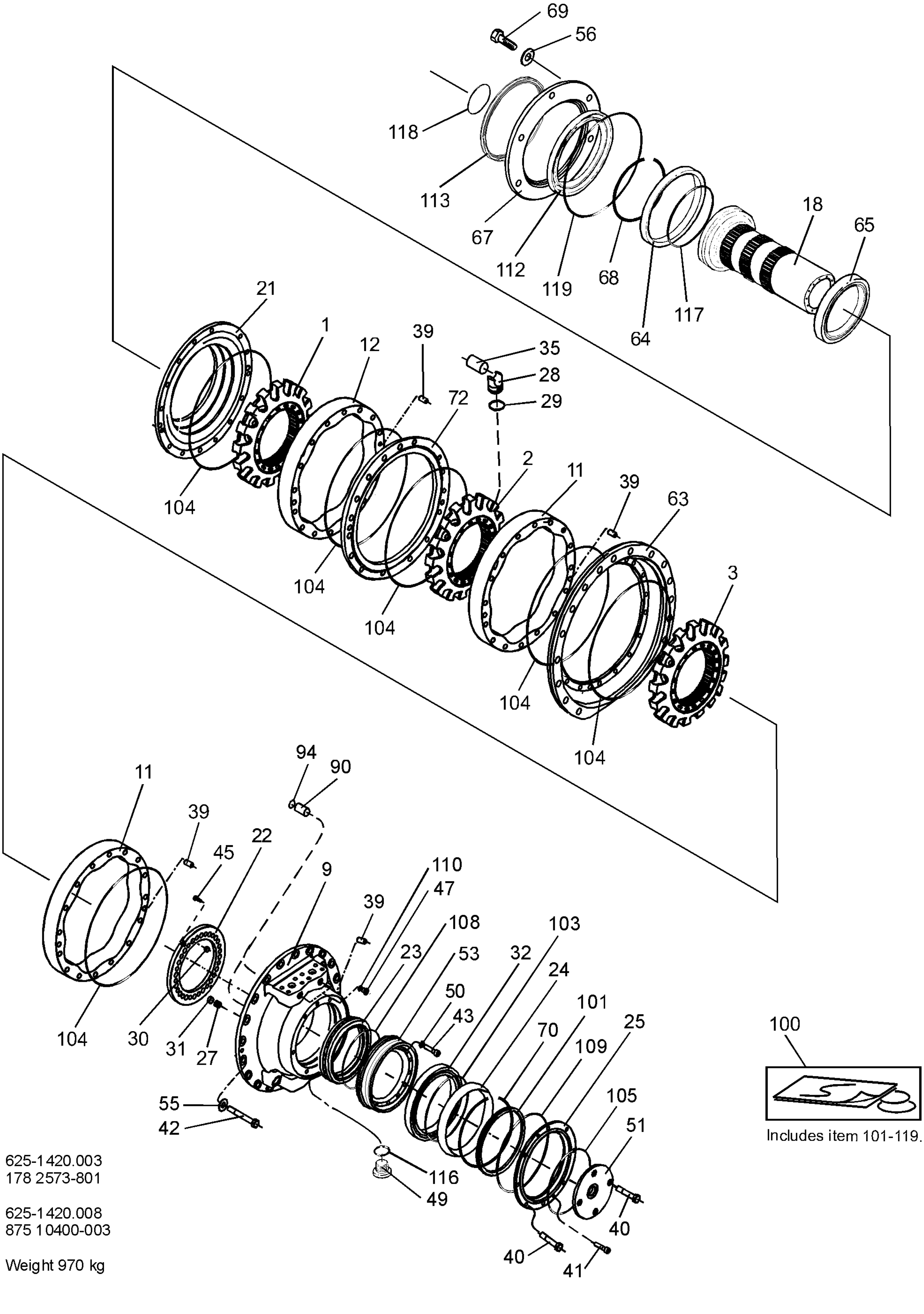

Includes item 101-119.

625-1420.003
178 2573-801

625-1420.008
875 10400-003

Weight 970 kg

HYDRAULIC MOTOR

Item	Qty	Article no	Description	Supplementary data
108	1	2152 2115-847	.. O-RING	
109	1	2152 2115-930	.. O-RING	
110	2	2152 2115-809	.. O-RING	
112	1	478 5001-801	.. RADIAL LIP SEAL	
113	1	378 2252-001	.. DUST SEAL	
116	1	2152 2115-830	.. O-RING	
117	1	2152 2118-887	.. O-RING	
118	1	2152 2115-541	.. O-RING	
119	1	2152 2115-954	.. O-RING	

SPEED ENCODER, MOUNTING

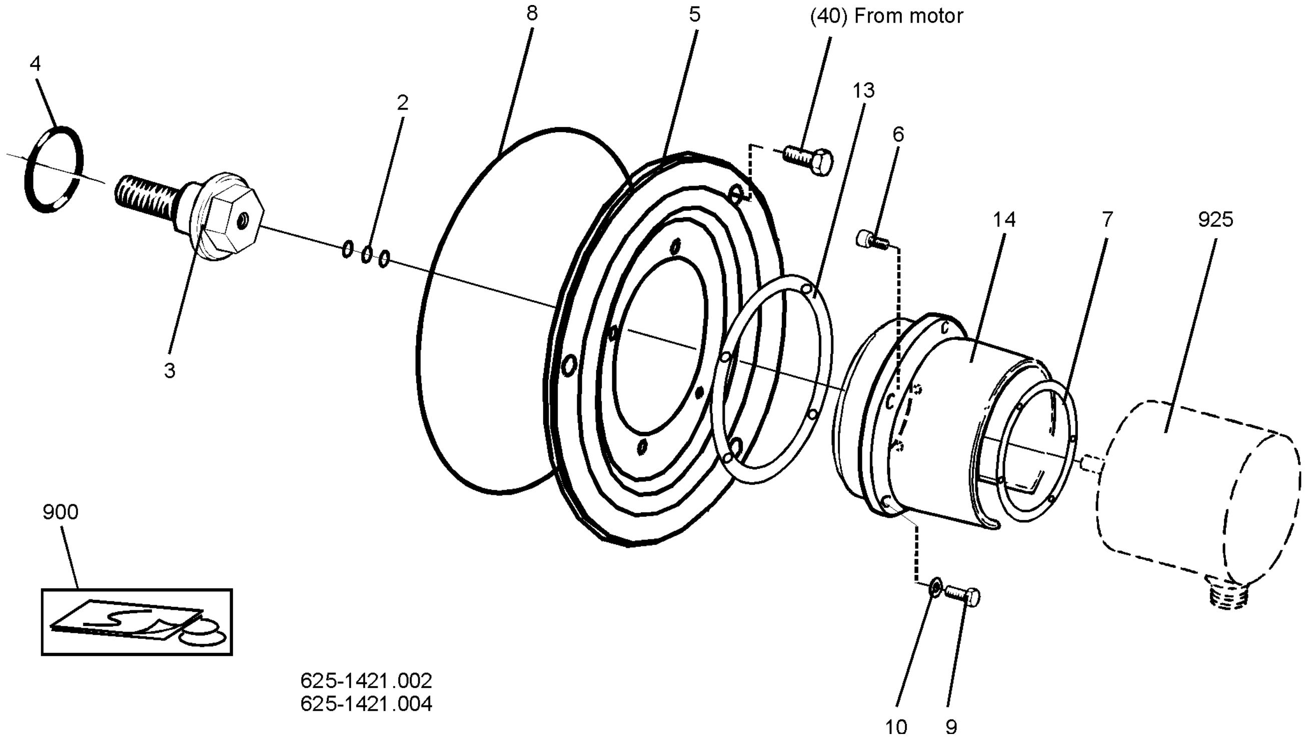

SPEED ENCODER, MOUNTING

Item	Qty	Article no	Description	Supplementary data
000	1	378 2264-801	SPEED ENCODER, MOUNTING	SMBA
002	3	2152 2118-305	. O-RING	Not sold separately. See item 900
003	1	378 2018-001	. DRIVE SCREW	
004	1	2152 2115-411	. O-RING	Not sold separately. See item 900
005	1	178 2609-001	. END COVER	
006	4	2121 2532-329	. SCREW	MC6S 5 x 16 -8.8 G
007	1	478 3376-001	. GASKET	Not sold separately. See item 900
008	1	2152 2115-930	. O-RING	Not sold separately. See item 900
009	4	2121 2032-451	. SCREW	M6S 8 x 20 -8.8 G
010	4	2151 2022-164	. WASHER	BRB 8.4 x 16 G
013	1	478 3742-001	. GASKET	Not sold separately. See item 900
014	1	278 2121-001	. HOUSING	
040	3	2121 2037-540	. SCREW	M6S 12 x 40 -10.9
900	1	378 2273-801	. GASKET SET	Incl. item 002, 004, 007, 008, 013
925			SPEED ENCODER	See 9.1 Winch

MULTI DISC BRAKE

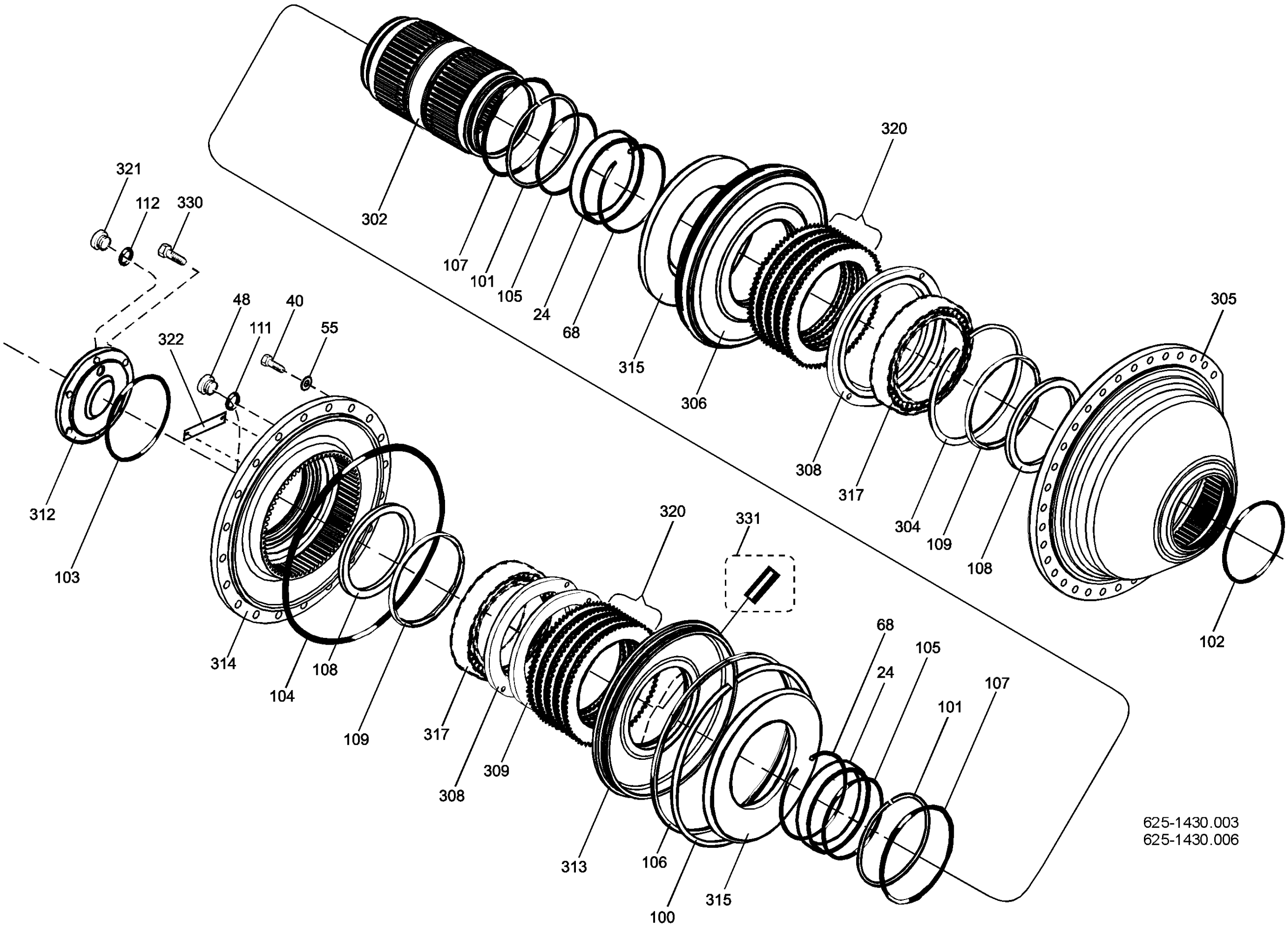

MULTI DISC BRAKE

Item	Qty	Article no	Description	Supplementary data
000	1	178 2581-702	MULTI DISC BRAKE	MDA 42
024	2	378 2086-001	. WEAR RING	
040	20	2121 2037-678	. SCREW	M6S 20 x 70 -10.9
048	2	2522 2111-115	. PLUG	R 3/4" (Item 111 incl.)
055	20	487 2826-009	. WASHER	
068	2	478 3702-001	. LOCK RING	
100	2	478 3750-002	. GUIDE STRING	Not sold separately. See item 900.
101	2	478 3750-001	. GUIDE STRING	Not sold separately. See item 900.
102	1	2152 2115-541	. O-RING	Not sold separately. See item 900.
103	1	2152 2118-889	. O-RING	Not sold separately. See item 900.
104	1	2152 2115-894	. O-RING	Not sold separately. See item 900.
105	2	2152 2118-725	. O-RING	Not sold separately. See item 900.
106	1	478 3737-001	. PISTON SEAL	Not sold separately. See item 900.
107	2	478 3734-001	. PISTON SEAL	Not sold separately. See item 900.
108	2	378 2256-001	. DUST SEAL	Not sold separately. See item 900.
109	2	378 2255-001	. RADIAL LIP SEAL	
111	2	2152 2115-822	. O-RING	Not sold separately. See item 900.
112	2	2152 2115-810	. O-RING	Not sold separately. See item 900.
302	1	178 2593-002	. DISC CENTRE	
304	1	478 3733-001	. LOCK RING	
305	1	178 2587-001	. BRAKE HOUSING, SHAFT END	
306	1	178 2608-002	. PISTON, SHAFT END	
308	2	278 2116-001	. SPACER RING	
312	1	378 2258-001	. COVER	
313	1	178 2608-001	. PISTON, PORT END	
314	1	178 2591-001	. BRAKE HOUSING, PORT END	
315	2	278 2120-001	. CUP SPRING	
317	2	378 2259-801	. CYL. ROLLER BEARING	
320	2	378 2257-801	. DISC SET MDA 42	NB! Matched disc set.
321	1	2522 2111-113	. PLUG	
322	1	487 0179-001	. WARNING SIGN	
330	6	2121 2032-451	. SCREW	M6S 8 x 20 -8.8 FZB; DIN 933
331	2	2111 2111-336	. SPRING PIN	6x14; Valid up to individ K28A 00199.
900	1	478 3740-802	. GASKET SET	Incl. items 100-112

LUFFING WINCH

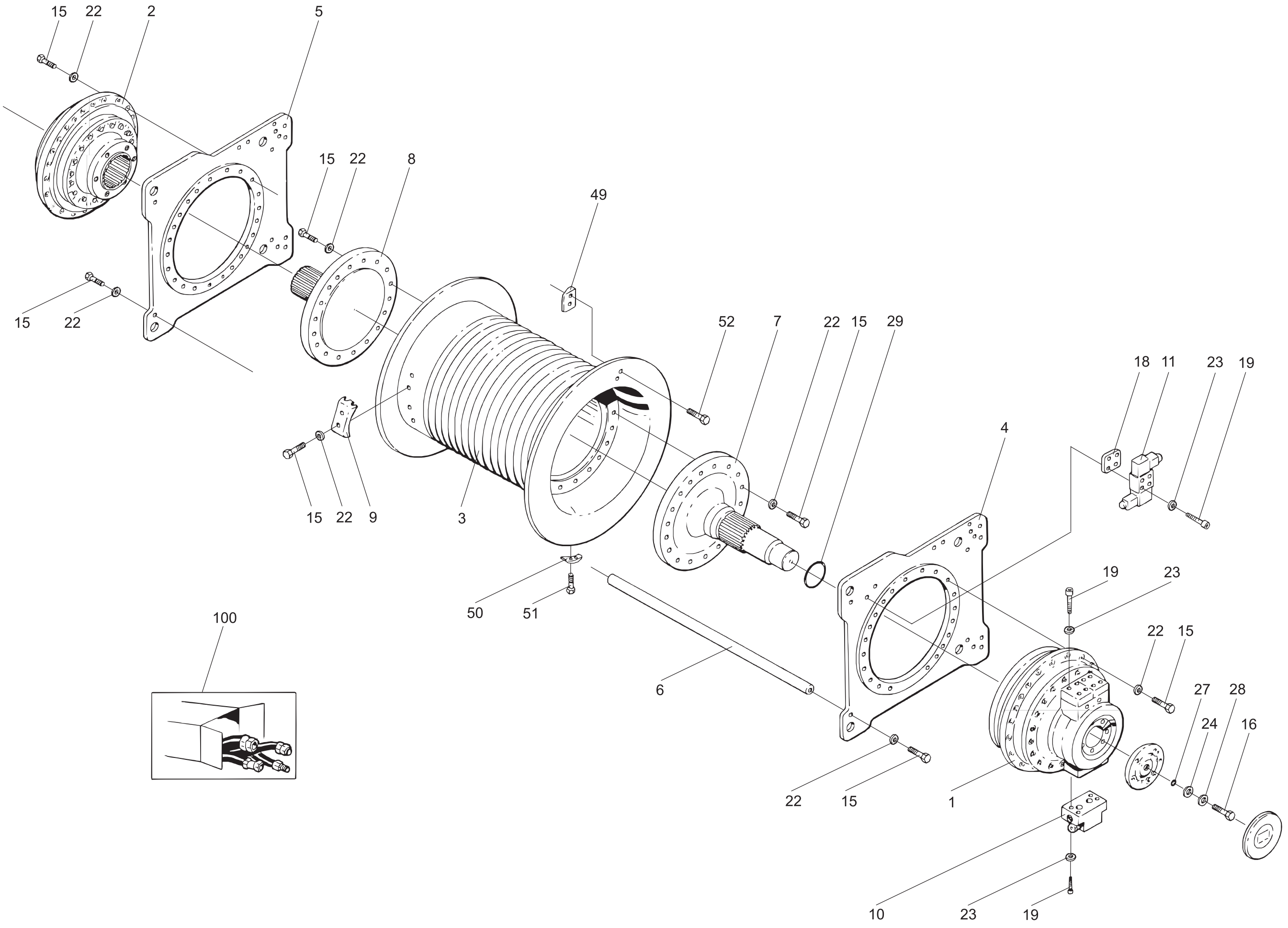

LUFFING WINCH

Item	Qty	Article no	Description	Supplementary data
000	1	188 0538-801	LUFFING WINCH	
001	1	875 10400-004	. HYDRAULIC MOTOR	CA 140; SEE FIG. 625-1420.009
002	1	875 10800-001	. MULTI DISC BRAKE	MDA 21; SEE FIG. 625-1430.004
003	1	178 2560-001	. LUFFING DRUM	
004	1	178 2562-801	. BRACKET PLATE	MOTOR SIDE
005	1	178 2563-801	. BRACKET PLATE	BRAKE SIDE
006	4	178 2419-001	. SUPPORT	
007	1	178 2564-001	. CENTRE SHAFT	140
008	1	178 2415-001	. CENTRE SHAFT	MDA21
009	2	278 2068-981	. WIRE CLAMP	
010	1	875 11000-005	. UNLOADING UNIT	SEE FIG. 625-7802.007
011	1	875 11000-001	. VALVE UNIT	SEE FIG. 625-7207.004
015	91	2121 2037-678	. SCREW	M6S 20 x 70 -10.9
016	1	2121 2037-684	. SCREW	M6S 20 x 100 -10.9
017	2	2121 2532-471	. SCREW	MC6S 8 x 100 -8.8 FZB
018	1	478 3739-001	. SPACER PLATE	
019	12	2121 2561-289	. SCREW	UC6S 1/2 UNC x 51 -12.9
022	91	2151 2022-192	. WASHER	BRB 21 x 36 FZB; DIN 125A
023	12	487 2826-007	. WASHER	13.5 x 19 x 2
024	1	478 3603-001	. WASHER	
027	1	2152 2115-404	. O-RING	
028	1	2152 2115-411	. O-RING	
029	1	2152 2115-607	. O-RING	
048	2	2166 4127-284	. TUBE CLIP	
049	1	378 2281-001	. KEY	
050	1	278 2137-001	. PITCH KEY	
051	2	2121 2532-453	. SCREW	MC6S 8 x 25 -8.8 FZB
052	2	2121 2032-461	. SCREW	M6S 8 x 50 -8.8 FZB
100	1	875 10200-002	. HYDRAULIC KIT, LUFFING WINCH	SEE FIG. 625-1411.005

HYDRAULIC KIT, LUFFING WINCH

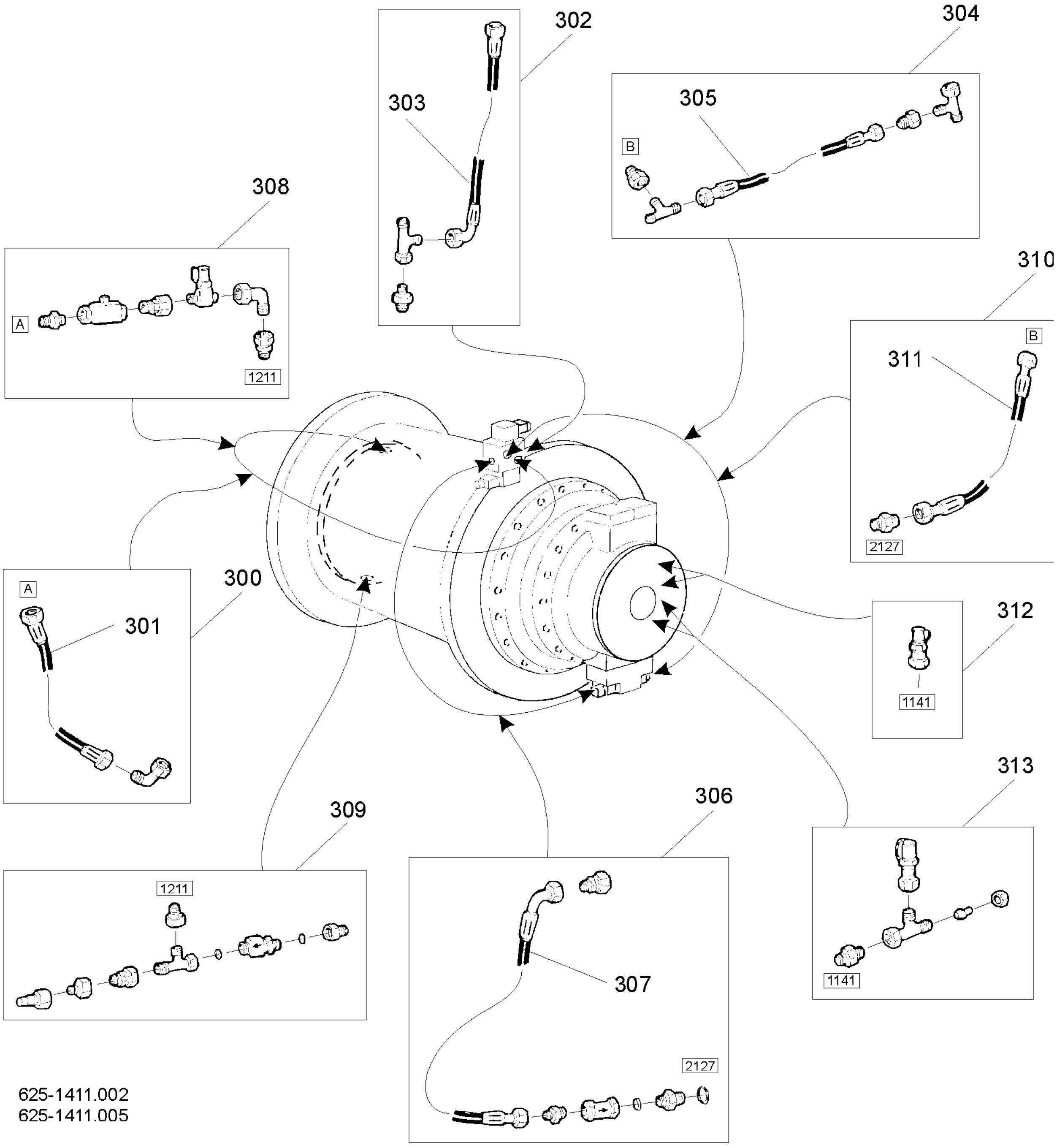

625-1411.002
625-1411.005

HYDRAULIC KIT, LUFFING WINCH

Item	Qty	Article no	Description	Supplementary data
000	1	478 3719-801	HYDRAULIC KIT, LUFFING WINCH	
300	1	178 2440-801	. HYDRAULIC HOSE, COMPLETE	
301	1	2515 6044-180	.. HYDRAULIC HOSE	
302	1	178 2623-801	. HYDRAULIC HOSE, COMPLETE	
303	1	2515 6244-140	.. HYDRAULIC HOSE	
304	1	178 2628-801	. HYDRAULIC HOSE, COMPLETE	
305	1	2515 6043-110	.. HYDRAULIC HOSE	
306	1	178 2446-801	. HYDRAULIC HOSE, COMPLETE	
307	1	2515 6231-085	.. HYDRAULIC HOSE	
308	1	178 2624-801	. ASS. KIT LUFFING BRAKE	
309	1	178 2625-801	. ASS. KIT LUFFING BRAKE	
310	1	178 2474-801	. HYDRAULIC HOSE, COMPLETE	
311	1	2515 6031-020	.. HYDRAULIC HOSE	
312	2	2529 2412-110	. MEASURE NIPPLE	
313	2	278 2143-801	. ASS. KIT LUFFING MOTOR	

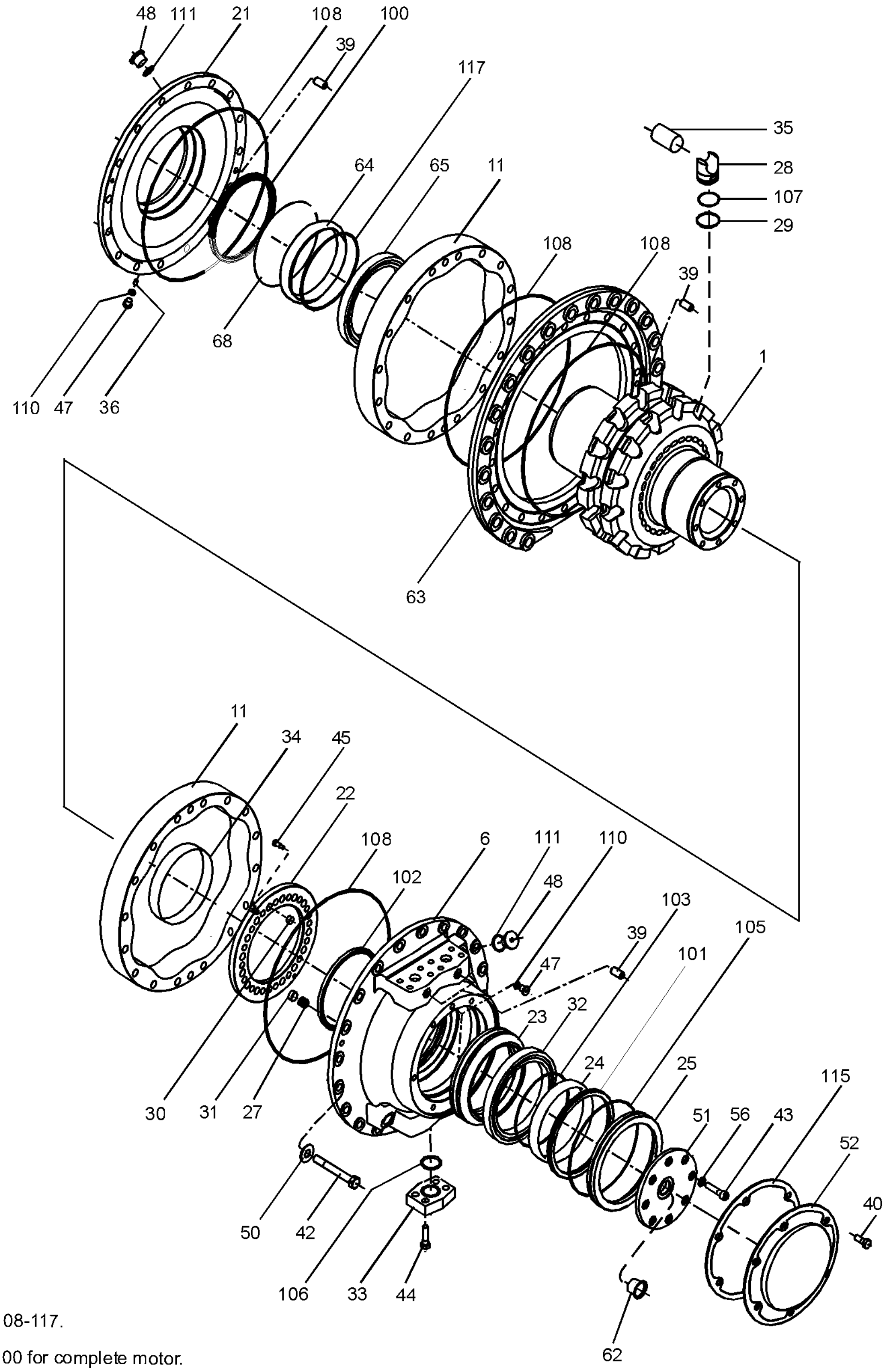

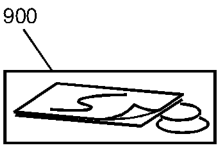

Note 1: Incl. 101-106, 108-117.

Add 1 of item 100 for complete motor.

Item 900 is an universal kit that fit all Compact Motors, less 1 main seal, that have to be added.

625-1420.004
625-1420.009

HYDRAULIC MOTOR

Item	Qty	Article no	Description	Supplementary data
000<br>CA 140	1	178 1934-701	HYDRAULIC MOTOR	CA 140
001	1	178 1931-001	. CYLINDER BLOCK	
006	1	178 1874-001	. CONNECTION BLOCK	
011	2	178 1949-001	. CAM RING	
021	1	178 1958-001	. SHAFT END HOUSING	
022	1	178 1919-001	. VALVE PLATE	
023	1	378 1651-801	. CYL. ROLL. THRUST BEARING	
024	1	378 1659-001	. WEAR RING	
025	1	378 1661-001	. SEAL RETAINER	
027	20	478 3292-001	. COMPR. SPRING	
028	32	278 1374-001	. PISTON	
029	32	478 3335-002	. PISTON RING	
030	20	478 3353-001	. BALANCING PISTON	
031	20	478 3354-001	. BALANCING SLEEVE	
032	1	478 3355-801	. CYL. ROLLER BEARING	
033	4	478 3356-001	. BLIND FLANGE	
034	1	478 3357-001	. WEAR RING	
035	32	478 3358-001	. CAM ROLLER	
036	1	487 5073-001	. ORIFICE PLUG	Diam. 1 mm
039	6	2111 2111-563	. ROLL PIN	FRP 16 x 40 SMS 1663
040	6	2121 2037-540	. SCREW	M6S 12 x 40 -10.9
042	20	2121 2040-652	. SCREW	M6S 16 x 190 -12.9
043	8	2121 2541-542	. SCREW	MC6S 12 x 50 -12.9
044	16	2121 2552-288	. SCREW	UC6S 1/2" UNC x 45 -8.8 G
045	2	2121 2592-371	. LOCATING BOLT	MC6S 6 x 23

HYDRAULIC MOTOR

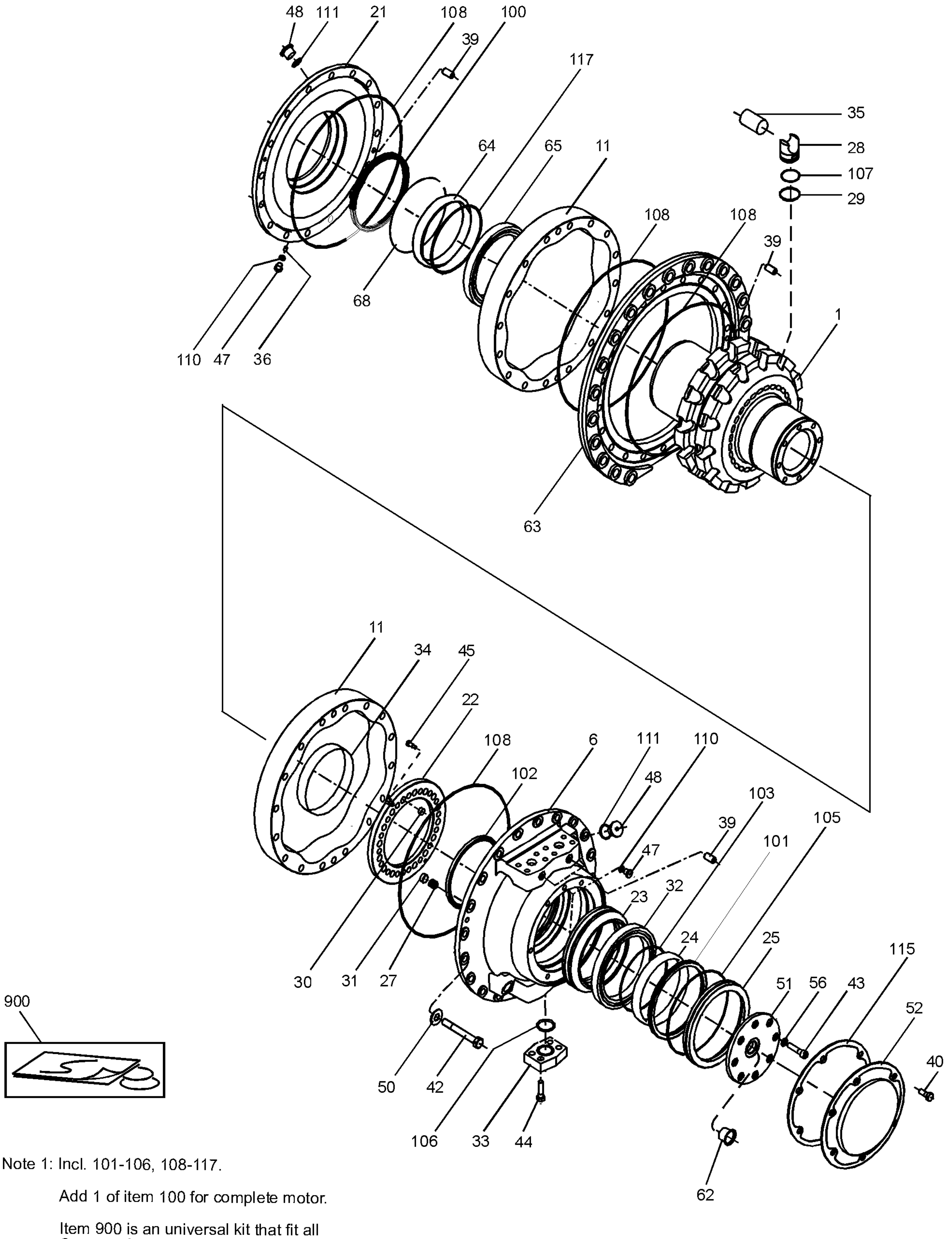

Note 1: Incl. 101-106, 108-117.

Add 1 of item 100 for complete motor.

Item 900 is an universal kit that fit all Compact Motors, less 1 main seal, that have to be added.

625-1420.004
625-1420.009

HYDRAULIC MOTOR

Item	Qty	Article no	Description	Supplementary data
047	5	2522 2111-110	. PLUG	BSP 1/4" (Item 110 incl.)
048	3	2522 2111-115	. PLUG	R 3/4" (Item 111 incl.)
050	20	487 2826-002	. WASHER	
051	1	278 1371-001	. BEARING RETAINER	
052	1	278 2073-001	. END COVER	
056	8	487 2826-007	. WASHER	
062	1	2522 4506-205	. PROTECTIVE CAP	
063	1	178 1956-001	. ATTACHMENT RING	
064	1	378 1737-001	. WEAR RING	
065	1	478 3371-801	. CYL. ROLL. BEARING	
068	1	478 3701-001	. LOCK RING	
100	1	478 3374-001	. RADIAL LIP SEAL	
101	1	378 1978-001	. RADIAL LIP SEAL	
102	1	378 1979-001	. RADIAL LIP SEAL	
103	1	2152 2118-889	. O-RING	Not sold separately. See item 900.
105	1	2152 2118-725	. O-RING	Not sold separately. See item 900.
106	4	2152 2115-832	. O-RING	Not sold separately. See item 900.
107	32	2152 2115-696	. O-RING	
108	4	875 11201-001	. O-RING	Not sold separately. See item 900.
110	5	2152 2115-809	. O-RING	Not sold separately. See item 900.
111	3	2152 2115-822	. O-RING	Not sold separately. See item 900.
115	1	278 2072-001	. SEAL	Not sold separately. See item 900.
117	1	2152 2118-899	. O-RING	Not sold separately. See item 900.
900	1	378 1729-803	. BASIC SEAL KIT, NITRIL	See Note 1

MULTI DISC BRAKE

Important

Always state the Individ-No of the brake, when ordering spare parts.

When changing to new Disc Centre item 303, also change Lock Ring from item 302 (rectangular cross-section) to item 304 (cirkular cross-section).

When changing to new Brake Housing item 306, also change Screws from item 041 to item 042.

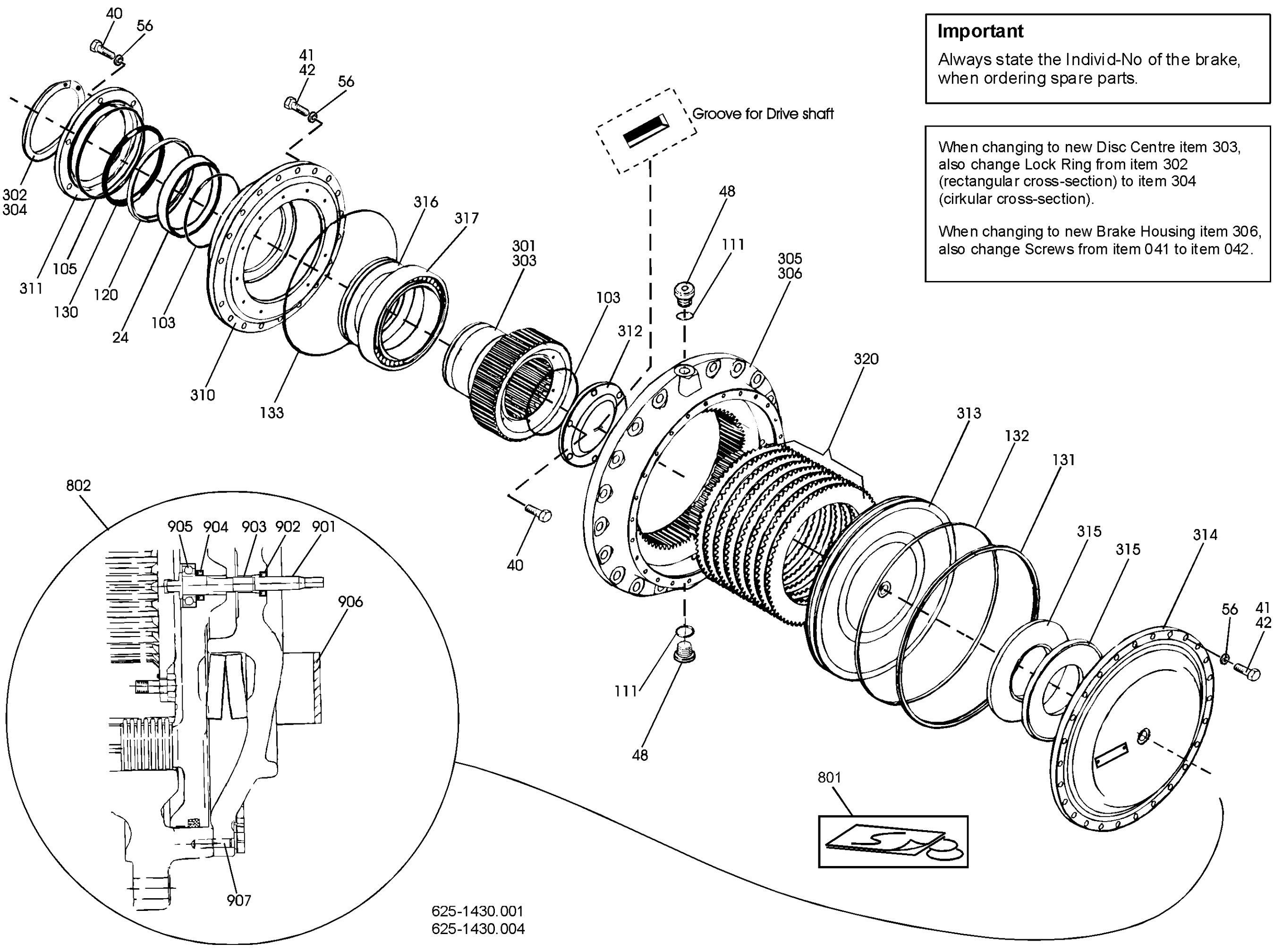

MULTI DISC BRAKE

Item	Qty	Article no	Description	Supplementary data
000	1	178 2268-722	MULTI DISC BRAKE	MDA 21
024	1	378 1659-001	. WEAR RING	
040	12	2121 2037-540	. SCREW	M6S 12 x 40 -10.9
041	46	2121 2037-540	. SCREW	M6S 12 x 40 -10.9. Valid up to K21A00299
042	46	2121 2037-544	. SCREW	M6S 12 x 60 -10.9. Valid from K21A00300.
048	2	2522 2111-115	. PLUG	R 3/4" (Item 111 incl.)
056	52	487 2826-007	. WASHER	
103	2	2152 2118-889	. O-RING	Not sold separately. See item 801.
105	1	2152 2118-725	. O-RING	Not sold separately. See item 801.
111	2	2152 2115-822	. O-RING	Not sold separately. See item 801.
120	1	378 1654-001	. RADIAL LIP SEAL	
130	1	378 1656-001	. DUST SEAL	
131	1	378 2064-001	. PISTON SEAL	
132	1	478 3360-002	. GUIDE STRING	
133	1	2152 2115-556	. O-RING	Not sold separately. See item 801.
301	1		. DISC CENTRE	Valid up to individ K21A00199.
302	1	478 3660-001	. LOCK RING	Valid up to individ K21A00199.
303	1	178 2687-801	. DISC CENTRE	Valid from individ K21A00200.
304	1	478 3681-001	. LOCK RING	Valid from individ K21A00200.
305	1		. BRAKE HOUSING	Valid up to individ K21A00299.
306	1	178 2791-001	. BRAKE HOUSING	Valid from individ K21A00300.
310	1	178 2271-001	. BEARING HOUSING	
311	1	178 2279-001	. SEAL RETAINER	
312	1	378 2060-001	. COVER	

MULTI DISC BRAKE

Important

Always state the Individ-No of the brake, when ordering spare parts.

When changing to new Disc Centre item 303, also change Lock Ring from item 302 (rectangular cross-section) to item 304 (cirkular cross-section).

When changing to new Brake Housing item 306, also change Screws from item 041 to item 042.

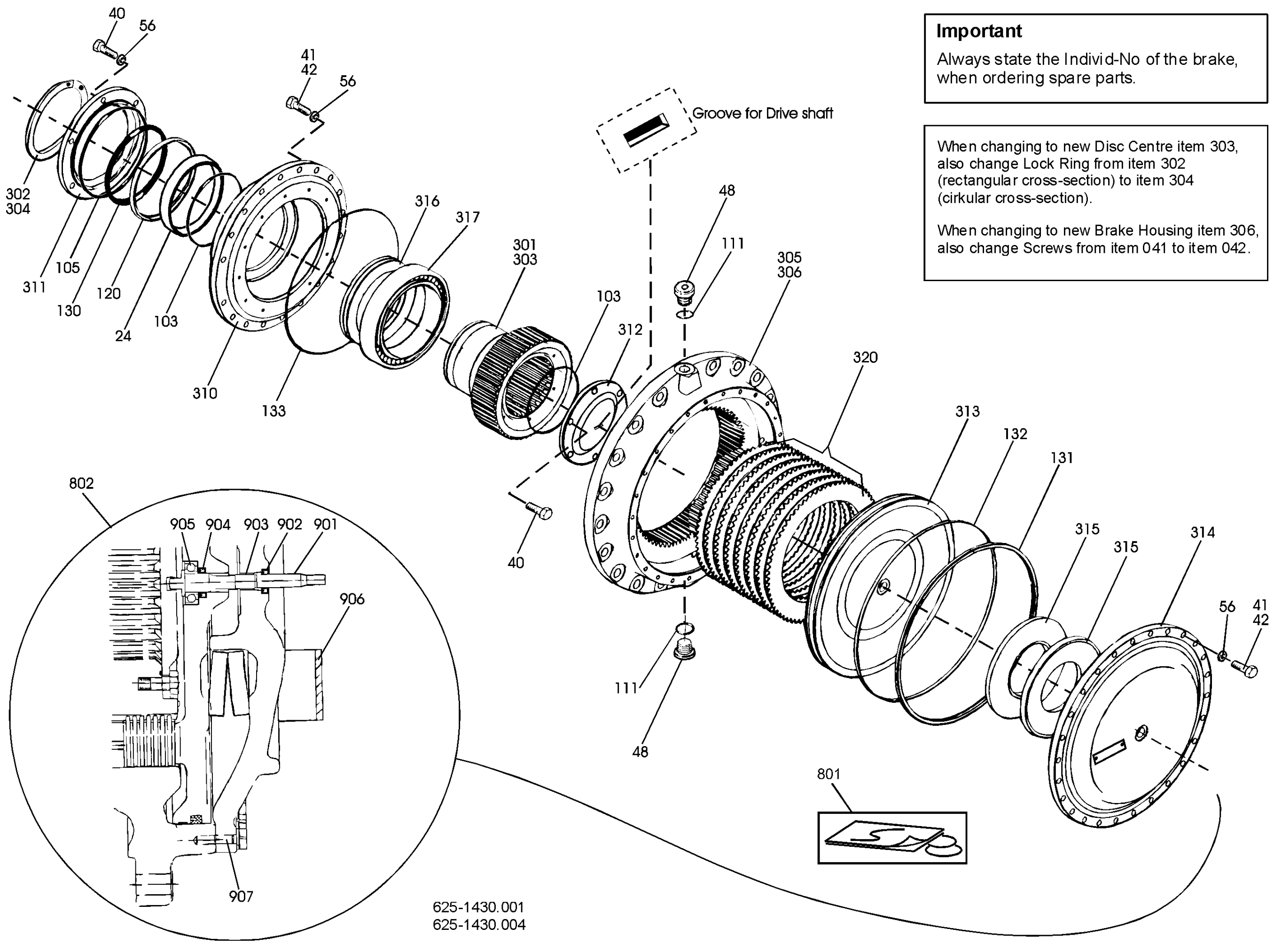

MULTI DISC BRAKE

Item	Qty	Article no	Description	Supplementary data
313	1	178 2275-001	. BRAKE PISTON	
314	1	178 2277-001	. BRAKE COVER	
315	2	278 1997-001	. CUP SPRING	
316	1	378 1651-802	. CYL. ROLL . THRUST BEARING	
317	1	378 2066-801	. CYL. ROLLER BEARING	
320	1	378 2121-802	. DISC SET MDA 21	NB! Matched disc set.
801	1	478 3659-802	. GASKET SET	Incl. 103-133, 902, 904.
802	1	278 2096-801	. DRIVE SHAFT COMPLETE	Incl. 901-907.
901	1	378 2221-001	.. DRIVE SHAFT	
902	1	2186 2117-126	.. RADIAL LIP SEAL	Not sold separately. See item 801/802.
903	1	2211 2219-131	.. BEARING BUSHING	
904	1	478 3779-001	.. RADIAL LIP SEAL	
905	1	2213 2304-171	.. ANG. CONT. BALL BEARING	Not sold separately. See item 802.
906	1	278 2095-001	.. BRACKET	
907	2	2111 2015-407	.. PARALLEL PIN	CP-M6 -8 x 35 SMS 2374.

SLEWING GEAR

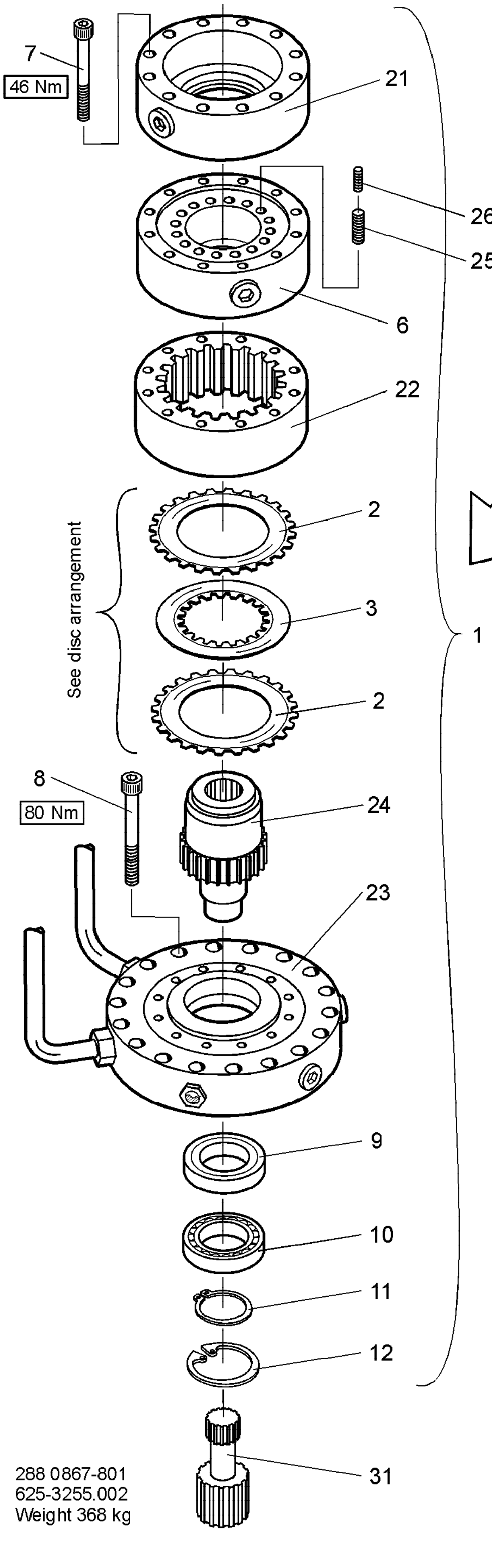

288 0867-801
625-3255.002
Weight 368 kg

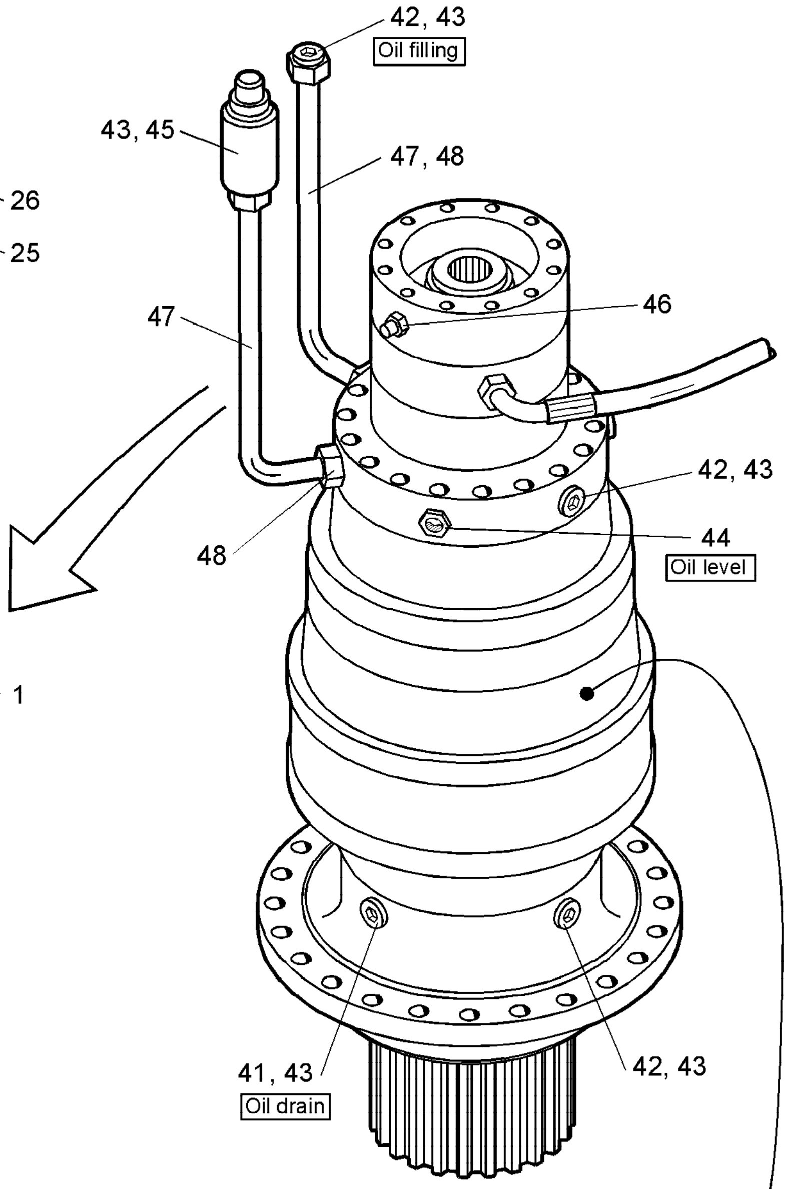

If the gearbox requires repair, please contact MacGREGOR Cranes to discuss what measures that should be taken.

Disc arrangement, item 2 + 3

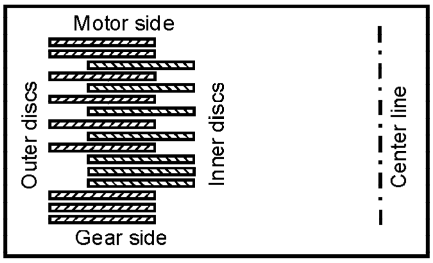

SLEWING GEAR

Item	Qty	Article no	Description	Supplementary data
000	1	288 0867-801	SLEWING GEAR	MODULE 16
001	1	875 13004-001	. DRIVE IN, COMPLETE	Includes item 2-24. Also order springs
002	9	875 13001-001	.. MULTIPLE DISC, OUTER	Change all 16 discs at the same time
003	7	875 13001-002	.. MULTIPLE DISC, INNER	Change all 16 discs at the same time
006	1	875 13003-001	.. PRESSURE UNIT	
007	12	2121 2532-515	.. SCREW	MC6S 10 x 120 -8.8 FZB; DIN 912
008	18	2121 2532-554	.. SCREW	MC6S 12 x 110 -8.8 FZB; DIN 912
009	1	875 13007-002	.. SHAFT SEALING	
010	1	875 13011-001	.. BEARING	
011	1	875 13012-001	.. CIRCLIP	
012	1	875 13012-002	.. CIRCLIP	
021	1	875 13027-006	.. MOTOR FLANGE	
022	1	875 13028-002	.. DISC CARRIER	
023	1		.. BRAKE FLANGE	Not sold separately. See item 1.
024	1		.. DRIVING SLEEVE	Not sold separately. See item 1.
025	18	875 13002-001	. SPRING	Change all 36 springs at the same time
026	18	875 13002-002	. SPRING	Change all 36 springs at the same time
031	1		. SUN WHEEL	Not sold as spare part.
041	1	875 13006-001	. PLUG, MAGNETIC	M22
042	6	875 13006-002	. PLUG	M22
043	8	875 13007-001	. SEALING	
044	2	875 13005-001	. OIL SIGHT GLASS	M22
045	1	875 13008-002	. VENTILATION FILTER	M22
046	1	875 13008-001	. VENTILATION FILTER	M10
047	2	875 13009-001	. PIPE	
048	2	875 13010-001	. COUPLING	

HYDRAULIC MOTOR

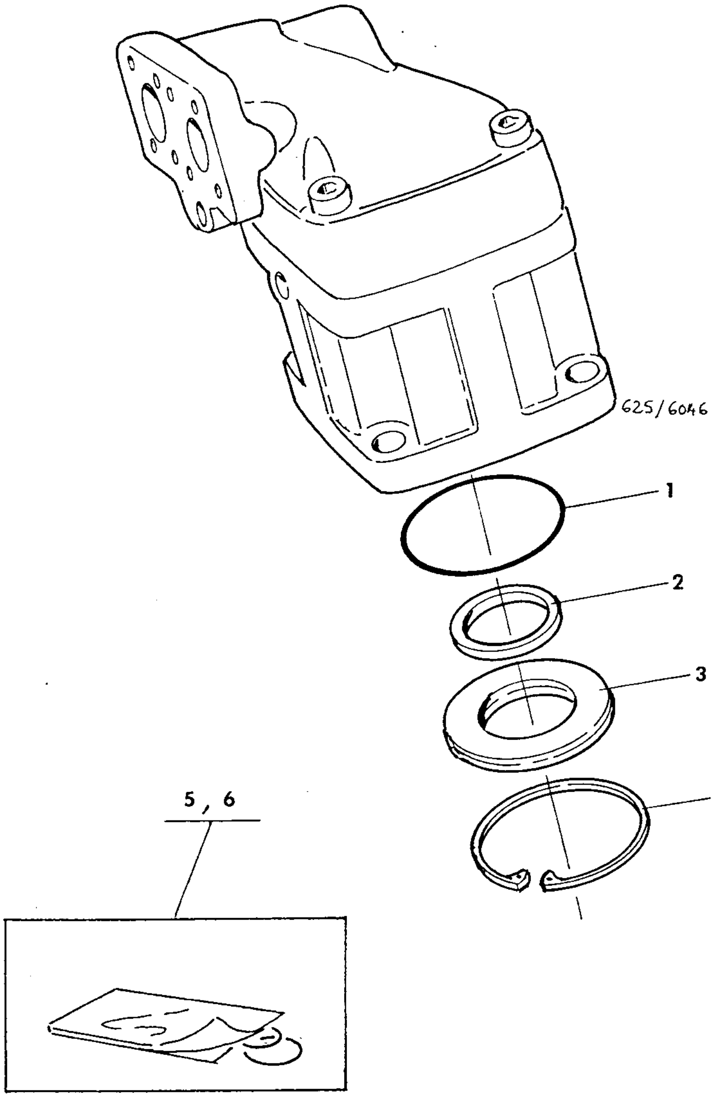

HYDRAULIC MOTOR

Item	Qty	Article no	Description	Supplementary data
000	1	388 3127-801	HYDRAULIC MOTOR	
001	1		. O-RING	
002	1		. SEAL CARRIER	
003	1		. SHAFT SEAL	
004	1		. RETAINING RING	
005	1	489 3716-801	. GASKET SET	INCLUDES ITEMS 001 AND 003
006	1	489 3716-802	. GASKET SET	INCLUDES ITEMS 001-004

PUMP UNIT

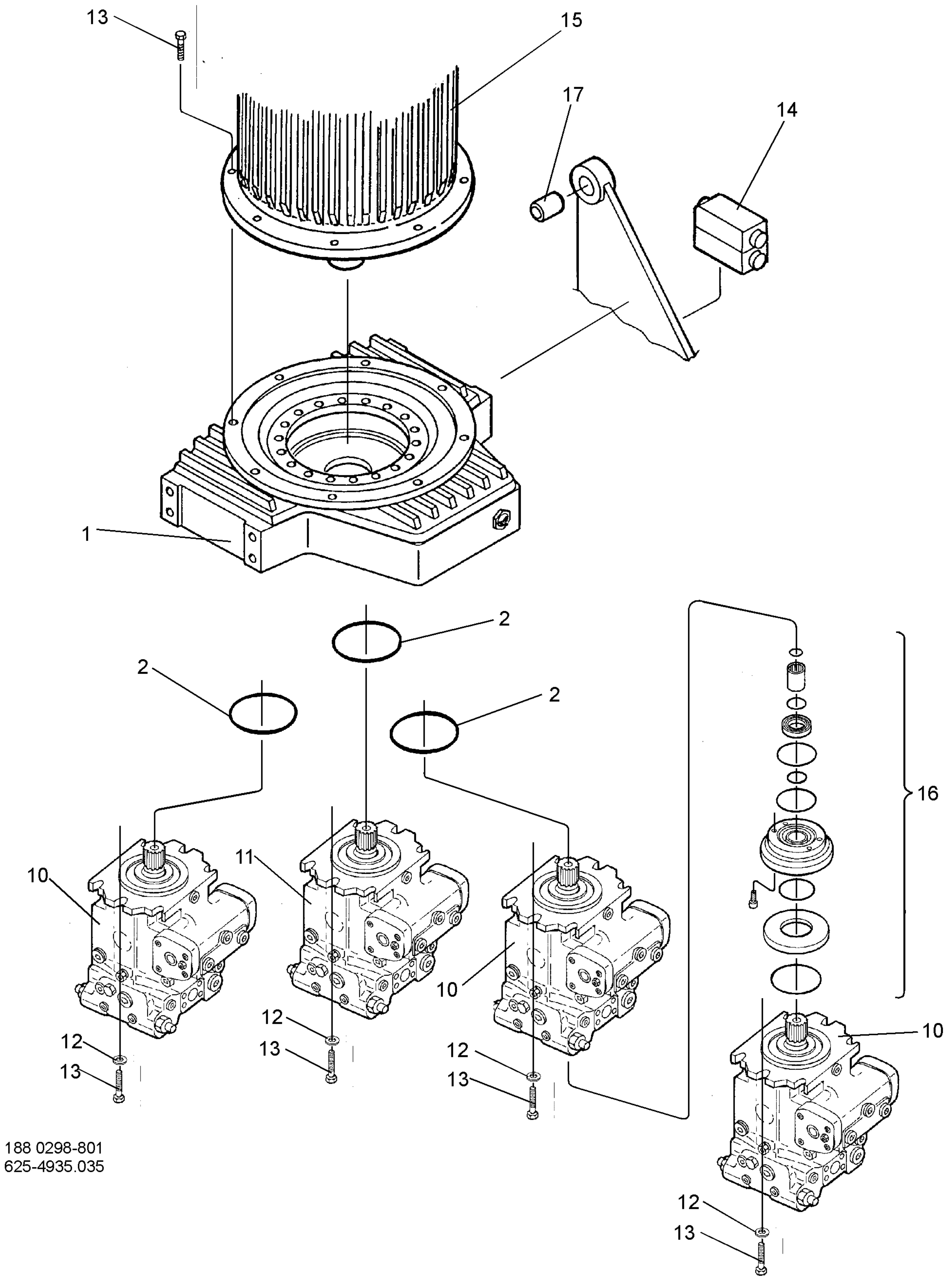
13
15
17
14
1
2
2
2
16
10
11
10
10
12
13
12
13
12
13
12
13
188 0298-801
625-4935.035

PUMP UNIT

Item	Qty	Article no	Description	Supplementary data
000	1	188 0298-801	PUMP UNIT	
001	1	388 5166-801	. GEAR BOX	SEE FIG. 625-4940.001
002	x		.. O-RING	SEE FIG. 625-4940.001
010	3	287 9493-801	. PUMP A4VG 125	SEE FIG. 625-4945.002
011	1	287 9494-801	. PUMP A4VG 125	SEE FIG. 625-4945.003
012	8	2151 2052-192	. WASHER	TBRB 21 x 36 FZB
013	24	2121 2032-674	. SCREW	M6S 20 x 50 -8.8 FZB; DIN 933
014	1	388 3580-801	. FLUSH AND UNLOADING UNIT	
015	1	388 6313-801	. ELECTRIC MOTOR	
016	1	388 5167-801	. TANDEM ASSEMBLY KIT	SEE FIG. 625-4963
017	4	2197 2525-055	. RUBBER BUSHING	

PUMP A4VG 125

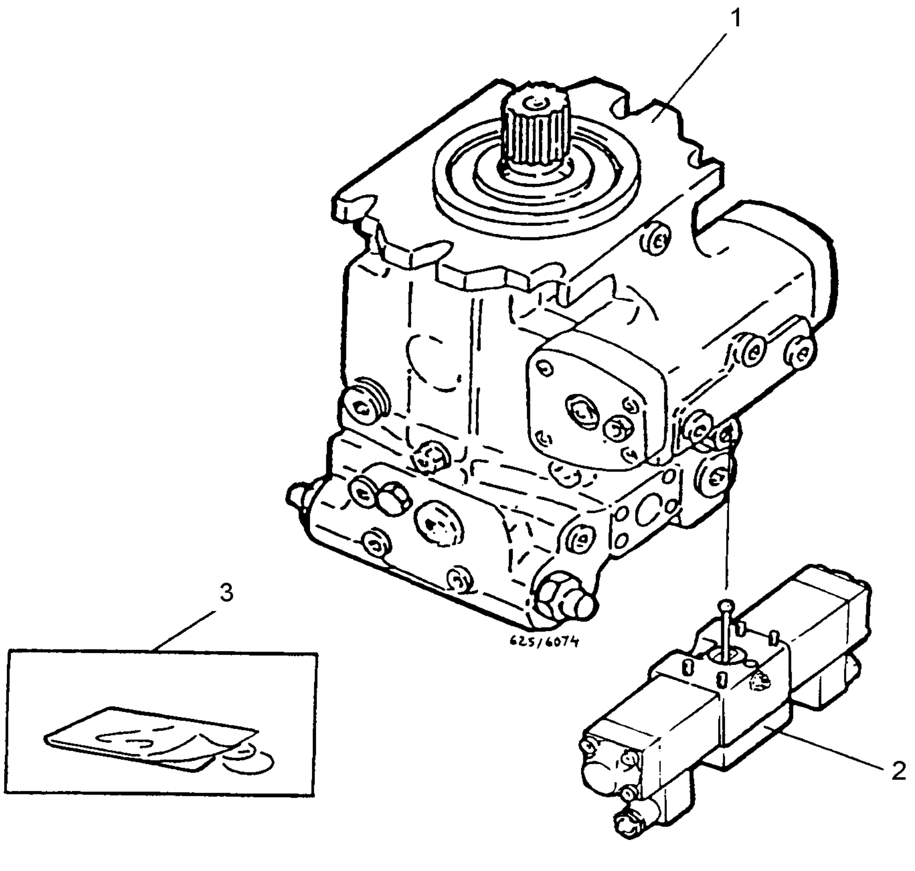

PUMP A4VG 125

Item	Qty	Article no	Description	Supplementary data
000	1	287 9493-801	PUMP A4VG 125	
001	1	287 9467-801	. PUMP	
002	1	388 7174-801	.. SERVO VALVE, C	
003	1	388 7173-803	.. GASKET SET	

PUMP A4VG 125

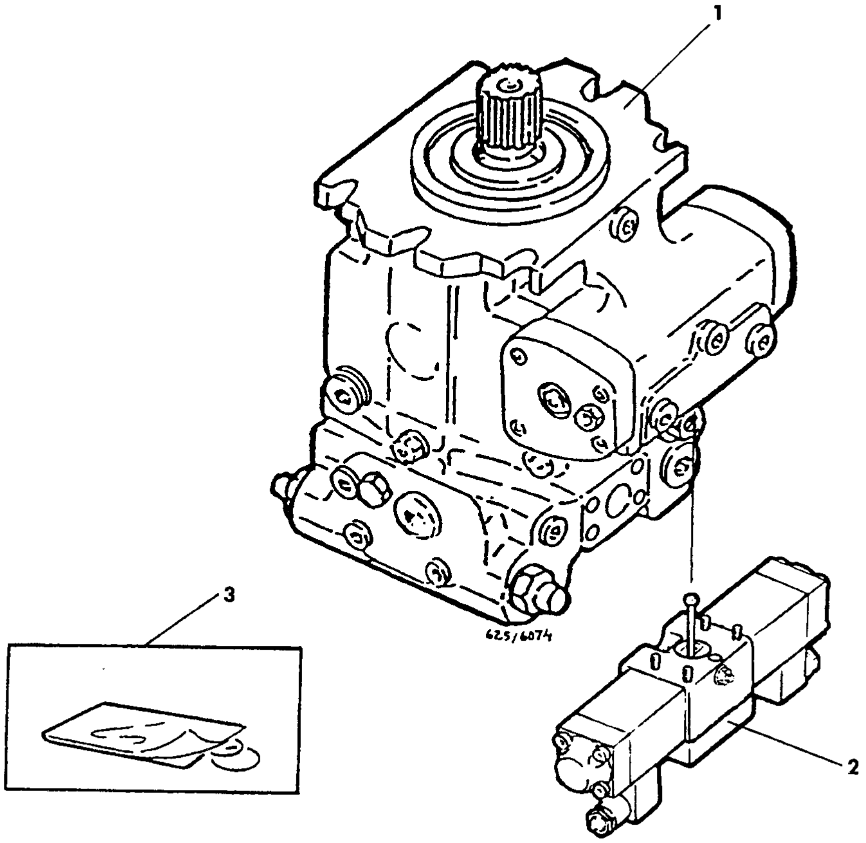

PUMP A4VG 125

Item	Qty	Article no	Description	Supplementary data
000	1	287 9494-801	PUMP A4VG 125	
001	1	287 9467-801	. PUMP	
002	1	388 7174-801	.. SERVO VALVE, C	
003	1	388 7173-803	.. GASKET SET	

TANDEM ASSEMBLY KIT

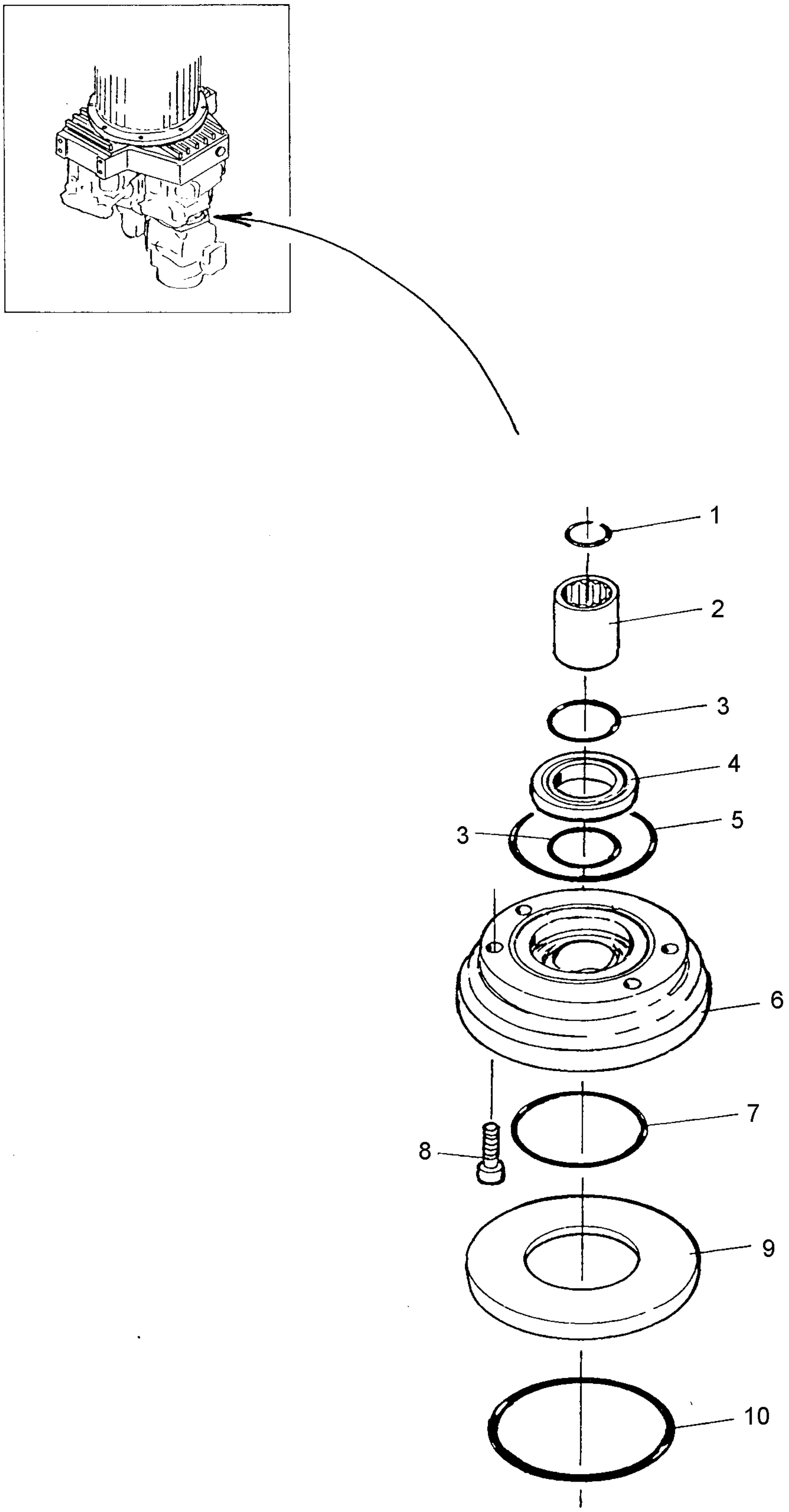

388 5167-801
625-4963

TANDEM ASSEMBLY KIT

Item	Qty	Article no	Description	Supplementary data
000	1	388 5167-801	TANDEM ASSEMBLY KIT	
001	1	388 5167-006	. LOCKING RING	
002	1	388 5167-003	. SPLINES CAGE	
003	2	388 5167-008	. O-RING	
004	1	388 5167-004	. SEALING RING	
005	1	388 5167-007	. O-RING	
006	1	388 5167-001	. PUMP FLANGE	
007	1	388 5167-009	. O-RING	
008	4	388 5167-005	. SCREW	MC6S 12 x 35 -10.9
009	1	388 5167-002	. PUMP RING	
010	1	388 5167-010	. O-RING	

OIL COOLER ASSEMBLY

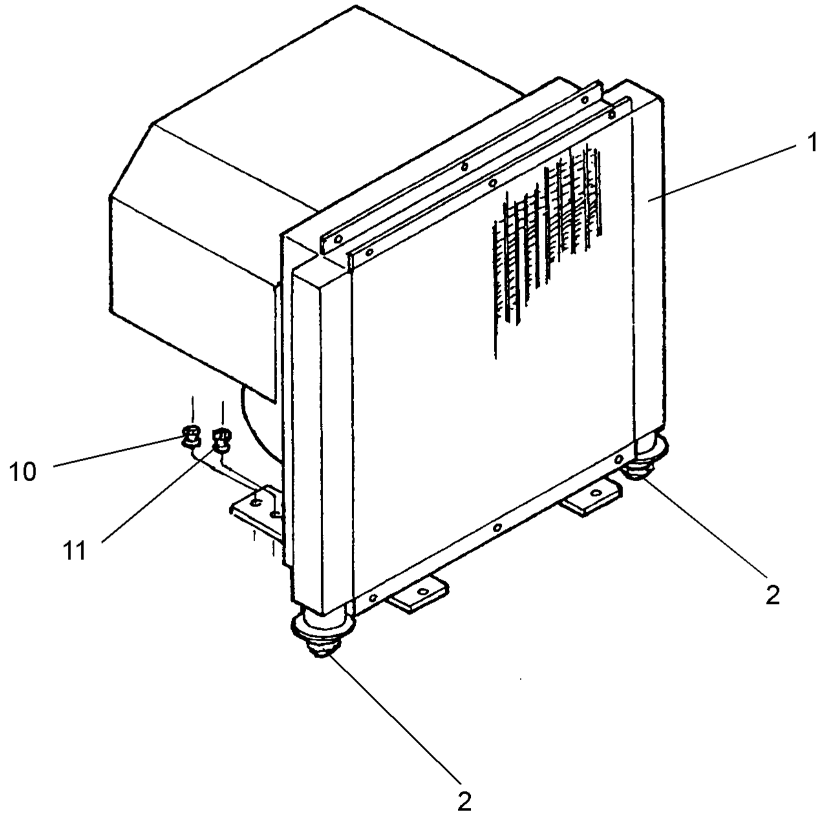

388 9899-801
625-5740.001

OIL COOLER ASSEMBLY

Item	Qty	Article no	Description	Supplementary data
000	1	388 9899-801	OIL COOLER ASSEMBLY	
001	1	289 2405-801	. OIL COOLER	
002	2	2528 2512-635	. COUPLING	
010	1	2166 2051-821	. FITTING	
011	1	2166 2051-822	. CABLE FITTING	

OIL COOLER

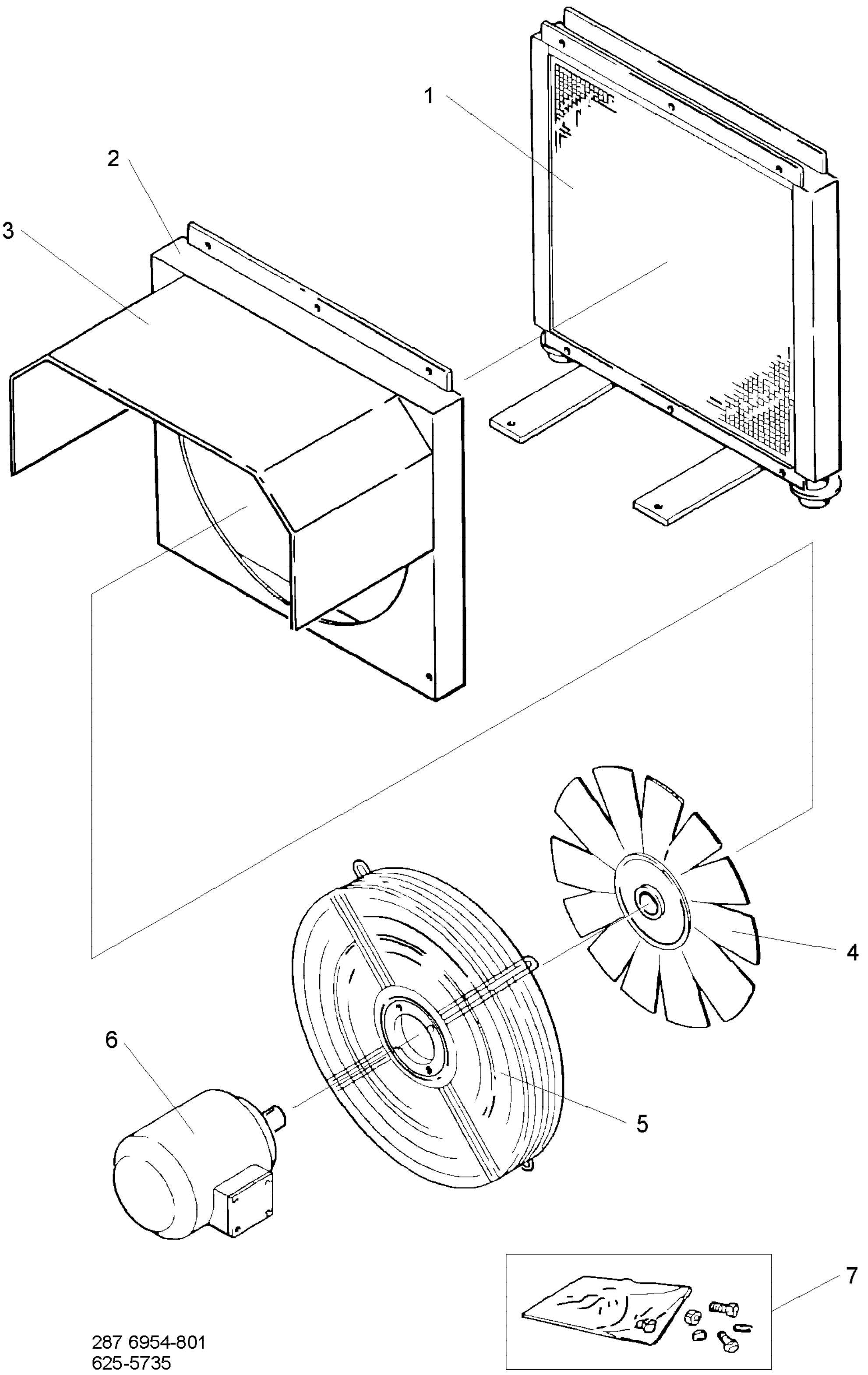

287 6954-801
625-5735

OIL COOLER

Item	Qty	Article no	Description	Supplementary data
000	1	287 6954-801	OIL COOLER	
001	1	287 6977-801	. COOLER ELEMENT	
002	1	875 18002-002	. ELEMENT COVER	
003	1	875 18004-001	. MOTOR COVER	
004	1	875 18005-001	. FAN WHEEL	
005	1	875 18003-001	. FAN WHEEL COVER	
006	1	875 18001-001	. ELECTRIC MOTOR	
007	1	490 0197-801	. MOUNTING SET	Incl. screws, nuts and washers

CRANE HOUSE MAIN COMPONENTS

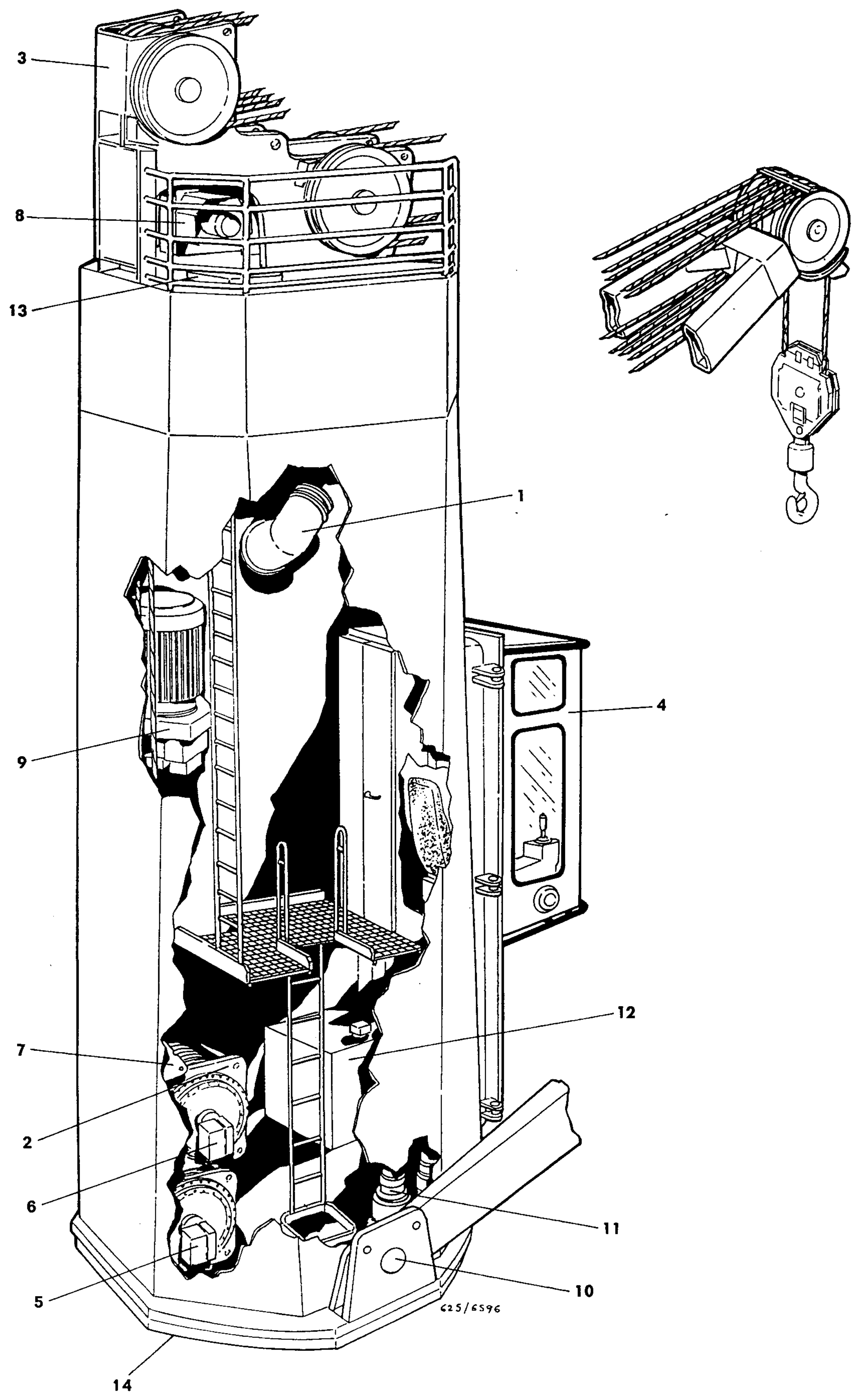

CRANE HOUSE MAIN COMPONENTS

Item	Qty	Article no	Description	Supplementary data
000	1	188 0718-801	CRANE HOUSE MAIN COMPONENTS	
001	1	288 0946-801	. VENTILATION FAN, MOUNT	
002	1	288 1412-801	. WINCH MOUNTING	
003	1	288 1878-801	. CRANE TOP MOUNTING	SEE FIG. 625-6459.007
004	1	288 1449-801	. CAB MOUNTING	
005	1	288 1103-803	. LIMIT SWITCHES BOX, HOISTING	
006	1	288 1104-804	. LIMIT SWITCHES BOX, LUFFING	
007	1	288 1441-801	. SLACK WIRE DEVICE	
008	1	388 9468-801	. OIL COOLER MOUNTING	
009	1	389 1447-801	. PUMP UNIT MOUNTING	
010	2	389 1451-801	. JIB BEARING MOUNTING	SEE FIG. 625-6170.018
011	1	389 1405-801	. SLEWING GEAR MOUNTING	
012	1	389 1448-801	. OIL TANK MOUNTING	
013	1	389 1828-801	. COVER MOUNTING	
014	1	389 1628-801	. SLEWING BEARING MOUNTING	

CRANE HOUSE MAIN COMPONENTS

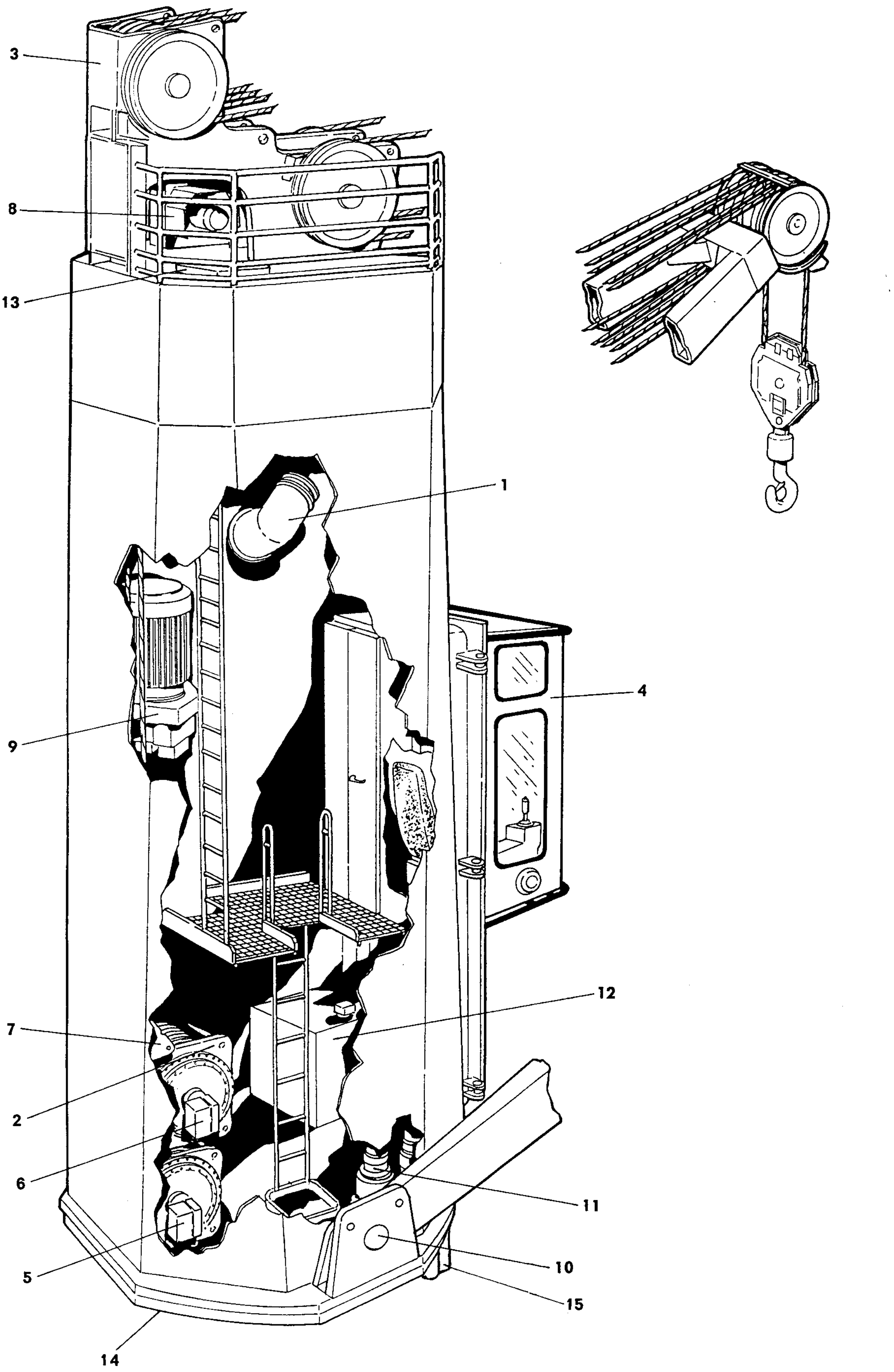

CRANE HOUSE MAIN COMPONENTS

Item	Qty	Article no	Description	Supplementary data
000	1	188 0719-801	CRANE HOUSE MAIN COMPONENTS	
001	1	288 0946-801	. VENTILATION FAN, MOUNT	
002	1	288 1412-801	. WINCH MOUNTING	
003	1	288 1878-801	. CRANE TOP MOUNTING	SEE FIG. 625-6459.007
004	1	288 1449-801	. CAB MOUNTING	
005	1	288 1103-803	. LIMIT SWITCHES BOX, HOISTING	
006	1	288 1104-804	. LIMIT SWITCHES BOX, LUFFING	
007	1	288 1441-801	. SLACK WIRE DEVICE	
008	1	388 9468-801	. OIL COOLER MOUNTING	
009	1	389 1447-801	. PUMP UNIT MOUNTING	
010	2	389 1451-801	. JIB BEARING MOUNTING	SEE FIG. 625-6170.018
011	1	389 1405-801	. SLEWING GEAR MOUNTING	
012	1	389 1448-801	. OIL TANK MOUNTING	
013	1	389 1828-801	. COVER MOUNTING	
014	1	389 1628-801	. SLEWING BEARING MOUNTING	
015	1	288 1444-801	. PARKING LOCKING MOUNTING	SEE FIG. 625-6160.001

PARKING LOCKING MOUNTING

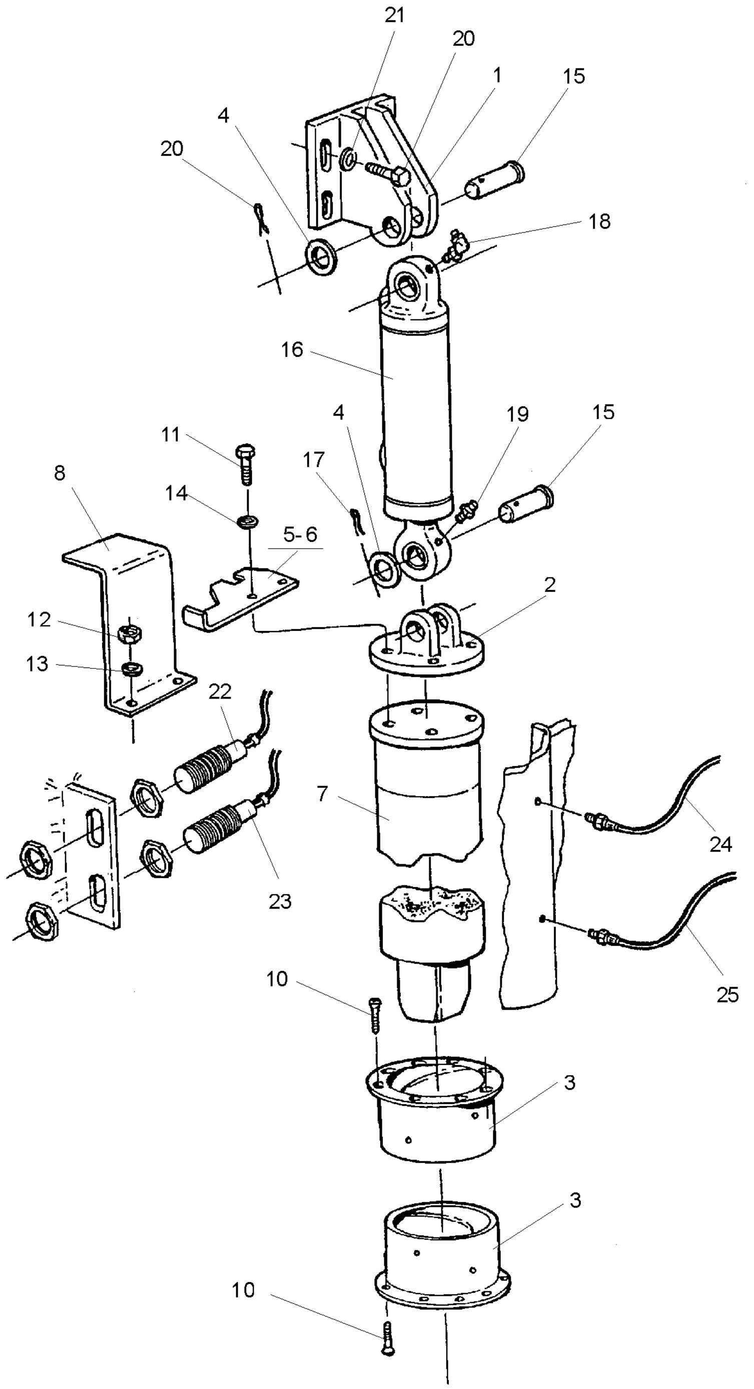

PARKING LOCKING MOUNTING

Item	Qty	Article no	Description	Supplementary data
000	1	288 1444-801	PARKING LOCKING MOUNTING	
001	2	287 7218-801	. BRACKET	
002	2	287 7218-802	. BRACKET	
003	4	388 2650-001	. BUSHING	
004	4	2151 2027-192	. WASHER	RB 12 x 37 A4
005	1	388 5064-001	. GUIDE PLATE	
006	1	388 5064-002	. GUIDE PLATE	
007	2	388 5097-001	. PIN	
008	2	489 3365-001	. PROTECTOR PLATE	
009	4	004 8742-801	. GREASE NIPPLE	
010	32	2121 2615-493	. SCREW	MF6S 10 x 25 -A4-80
011	16	2121 2034-538	. SCREW	M6S 12 x 35 -A4 -80
012	4	2126 2034-118	. NUT	M6M 8 -A4 -80
013	4	2151 2025-164	. WASHER	BRB 8.4 x 16 SSt
014	8	2151 2025-178	. WASHER	BRB 13 x 24 SSt
015	4	488 8930-001	. CYLINDRIC BOLT	
016	2	388 5046-801	. HYDRAULIC CYLINDER, C	SEE FIG. 625-4493
017	4	2114 2014-201	. SPLIT PIN	SP 4 x 40 A4
018	1		. GREASE NIPPLE	
019	1		. GREASE NIPPLE	
020	8	2121 2034-538	. SCREW	M6S 12 x 35 -A4 -80
021	16	2151 2059-179	. WASHER	TBRSB 13.5 x 36 RF
022	2		IMPULSE UNIT	SEE EL. INSTALL. CRANE PARKING LOCK
023	2		IMPULSE UNIT	SEE EL. INSTALL. CRANE PARKING LOCK
024	2		HYDRAULIC HOSE	
025	2		HYDRAULIC HOSE	

CRANE TOP MOUNTING

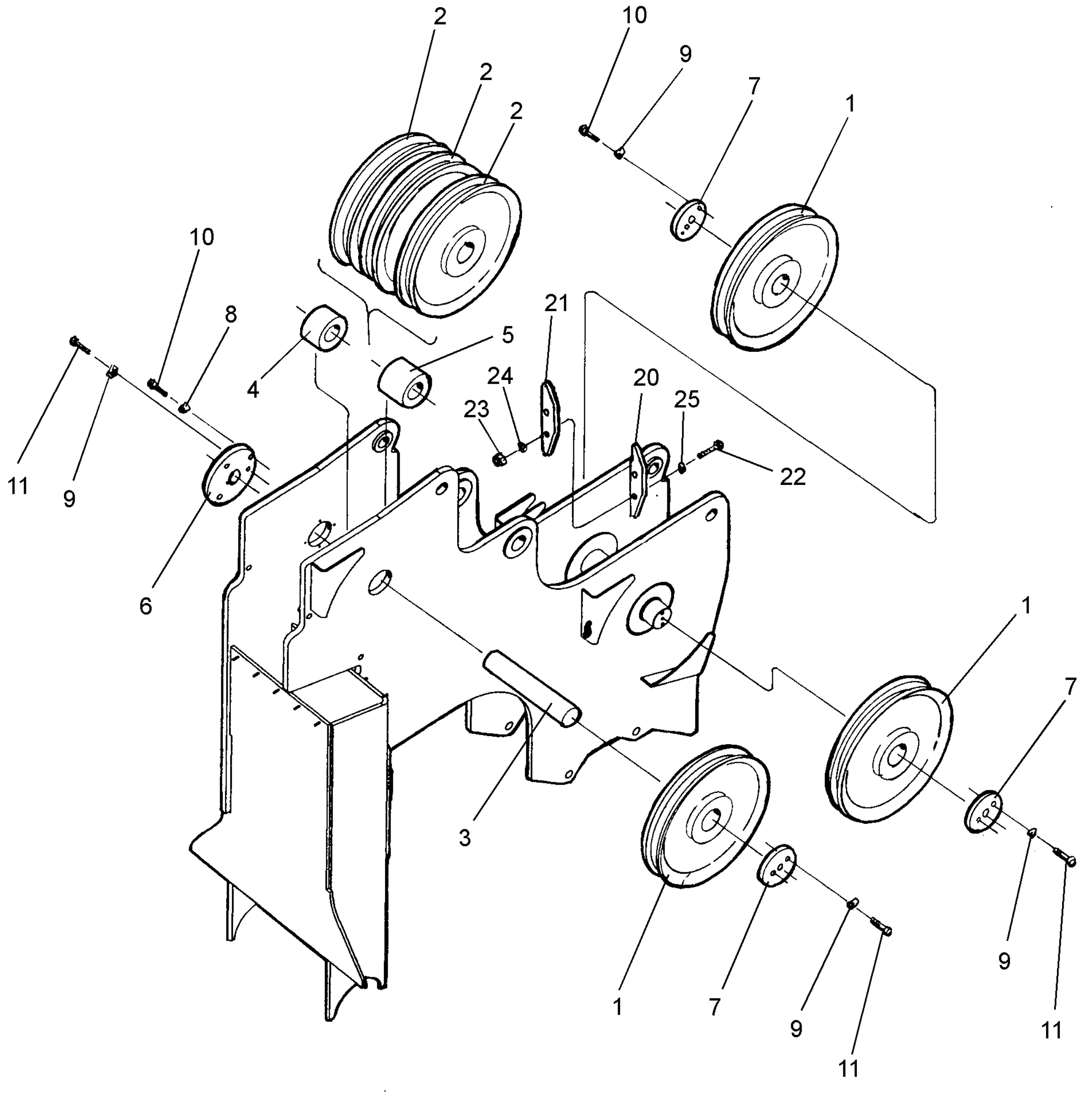

288 1878-801
625-6459.007

CRANE TOP MOUNTING

Item	Qty	Article no	Description	Supplementary data
000	1	288 1878-801	CRANE TOP MOUNTING	
001	3	489 5708-801	. WIRE SHEAVE	
002	3	489 5707-801	. WIRE SHEAVE	
003	1	288 1593-001	. SHAFT	
004	1	389 1913-002	. SPACER	
005	1	389 1913-005	. SPACER	
006	1	489 5691-001	. COVER	
007	3	489 5336-001	. COVER	
008	3	489 5334-001	. LOCK WASHER	
009	8	489 5334-002	. LOCK WASHER	17 SSt
010	4	2121 2032-495	. SCREW	M6S 10 x 30 -8.8 FZB
011	8	2121 2032-628	. SCREW	M6S 16 x 40 -8.8 FZB; DIN 933
020	1	289 2265-801	. SLIDING PLATE MOUNT	
021	1	490 3597-001	.. SLIDING PLATE	
022	2	2121 2034-507	.. SCREW	M6S 10 x 80 A4-80
023	2	2126 2634-120	.. NUT	NYLOC-M6M 10-A4-80
024	2	2151 2054-173	.. WASHER	TBRSUB 10.5 x 28 FZV
025	2	2151 2027-173	.. SPRING WASHER	RB 10.5 x 20 -A4

OIL TANK

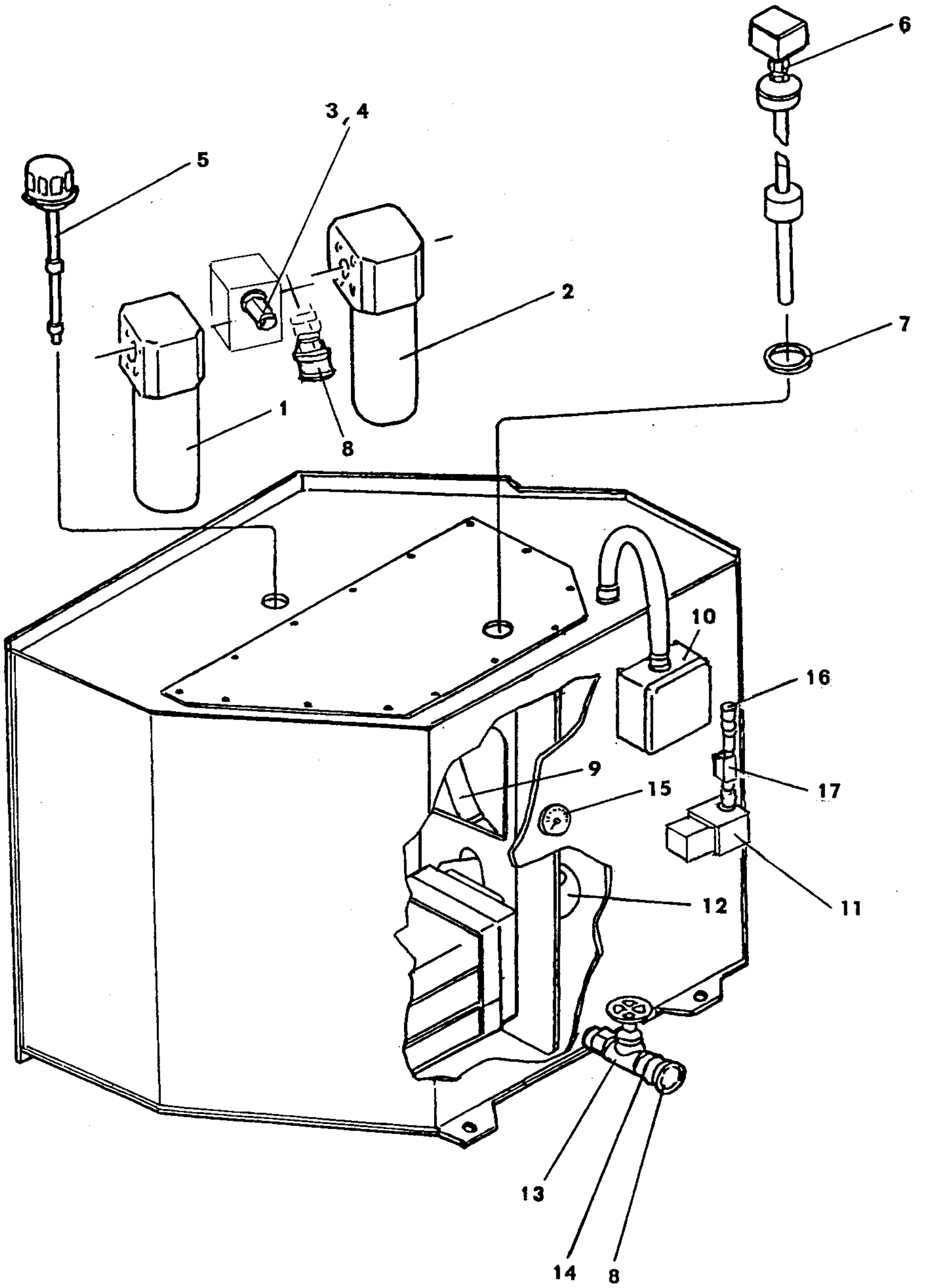

OIL TANK

Item	Qty	Article no	Description	Supplementary data
000	1	188 0343-801	OIL TANK	
001	1		. FILTER UNIT, OUTLET	SEE COMP. LIST HYDR. CIRCUIT DIAGRAM
002	1		. FILTER UNIT, INLET	SEE COMP. LIST HYDR. CIRCUIT DIAGRAM
003	1	2152 2151-200	. SEAL RING	
004	1	488 9540-801	. FLOW REGULATOR	SEE FIG. 625-7812
005	1		. AIR FILTER WITH DIPSTICK	SEE COMP. LIST HYDR. CIRC. DIAGRAM
006	1	214 1793-803	. LEVEL AND TEMPERATURE GUARD	
007	2	2152 2151-228	. SEAL RING	
008	2	2529 2558-116	. QUICK COUPLING	R 1"
009	1	2515 6148-090	. HYDRAULIC HOSE	
010	1	314 3672-801	. CONNECTION BOX	
011	1	287 5950-801	. DIRECTION VALVE	
012	1	388 0364-802	. FEED PUMP UNIT	SEE FIG. 625-5181.001
013	1	2541 2506-117	. VEDGE VALVE	1 1/4"
014	1	2521 2310-220	. NIPPLE	R 1 1/4" x 1"
015	1	489 5906-801	. THERMOMETER	Glued w. Bostic 10 or similar
016	1	2529 2558-111	. CONNECTING NIPPLE	
017	1	2541 4129-113	. BALL VALVE	

FEED PUMP UNIT

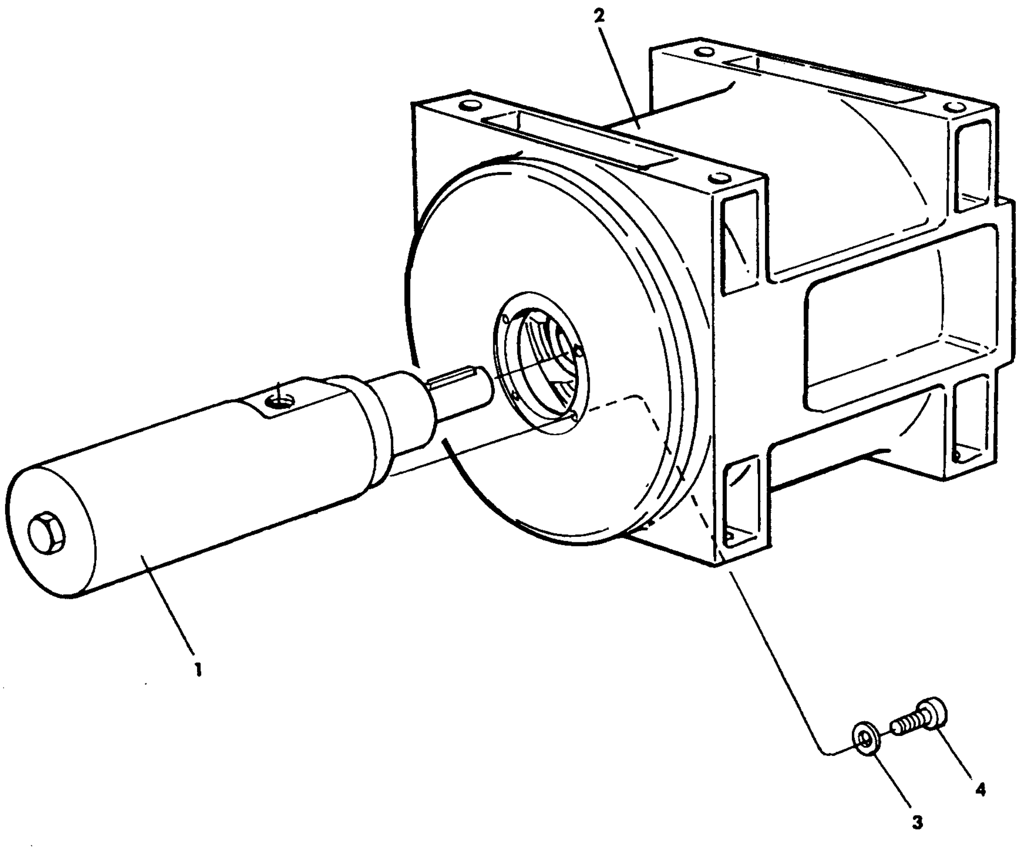

388 0364-802
625-5181.001

FEED PUMP UNIT

Item	Qty	Article no	Description	Supplementary data
000	1	388 0364-802	FEED PUMP UNIT	
001	1	388 1669-801	. PUMP	
002	1	388 0369-801	. ELECTRIC MOTOR	
003	4	2151 2052-165	. WASHER	TBRB 8.4 x 16 FZB
004	4	2121 2532-451	. SCREW	MC6S 8 x 20 -8.8 G

SLACK WIRE DEVICE

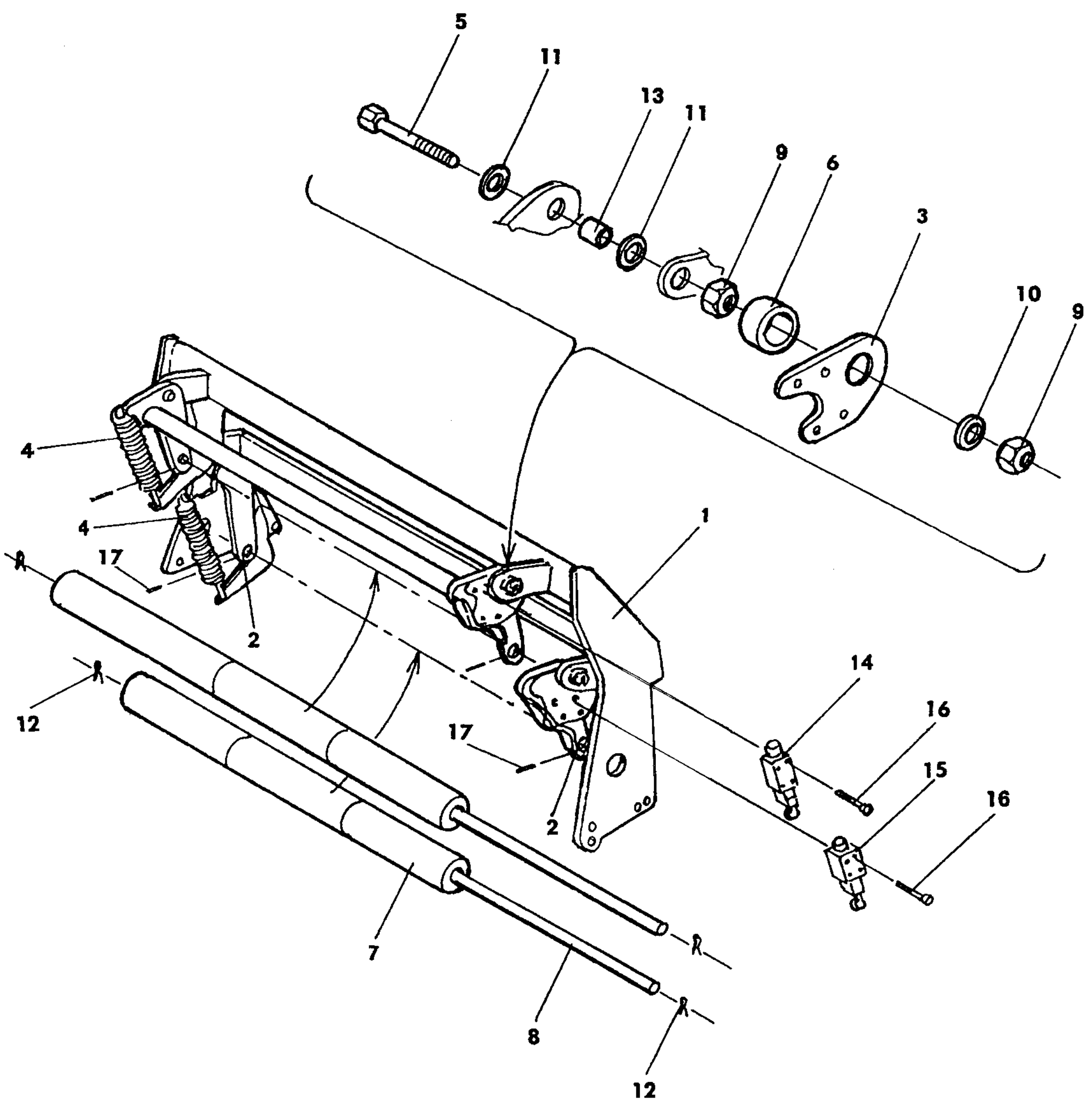

SLACK WIRE DEVICE

Item	Qty	Article no	Description	Supplementary data
000	1	288 1616-801	SLACK WIRE DEVICE	
001	1	288 1617-801	. SUPPORT	
002	2	288 1869-801	. BRACE	
003	2	388 3290-004	. LIMIT SWITCH PART	
004	2	488 3003-001	. SPRING	
005	2	489 4601-001	. SCREW	
006	2	489 4604-001	. SPACER	
007	6	489 5469-001	. ROLLER	
008	2	489 5470-001	. SHAFT	
009	6	2126 2636-124	. LOCKING NUT	NYLOC-M6M 16 -8.8 G
010	4	2151 2022-185	. WASHER	BRB 17 x 30 G
011	8	2151 2022-196	. WASHER	BRB 23 x 40 G
012	4	2114 2014-201	. SPLIT PIN	SP 4 x 40 A4
013	4	1865 2311-243	. BUSHING	PVSL 24/18 x 3 L = 18
014	1	5661 4116-512	. LIMIT SWITCH, HOISTING	
015	1	5661 4126-001	. LIMIT SWITCH	
016	8	2121 2550-339	. SCREW	MC6S 5 x 40 A4
017	4	2122 2515-327	. STOP SCREW	SK6SS 5 x 12

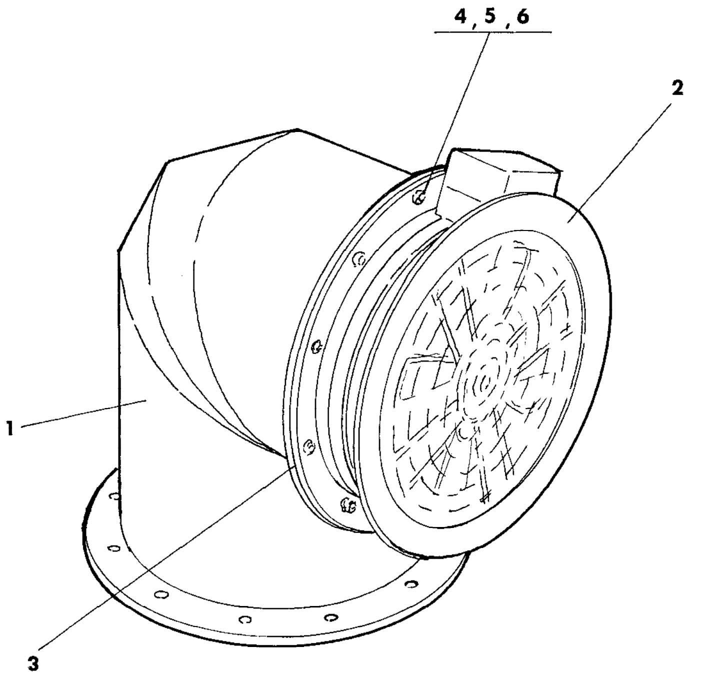
4, 5, 6
2
1
3

VENTILATION FAN, ASSEMBLY

Item	Qty	Article no	Description	Supplementary data
000	1	288 1118-801	VENTILATION FAN, ASSEMBLY	
001	1	288 0945-801	. TUB FOR FAN	
002	1	388 9464-801	. VENTILATION FAN	
003	x	1236 2296-020	. SEALING COMPOUND	
004	8	2121 2032-459	. SCREW	M6S 8 x 40 -8.8 FZB
005	8	2126 2636-118	. NUT	NYLOC -M6M 8 -8 FZB
006	8	2151 2058-165	. WASHER	TBRSB 8.4 x 26 FZB

CAB

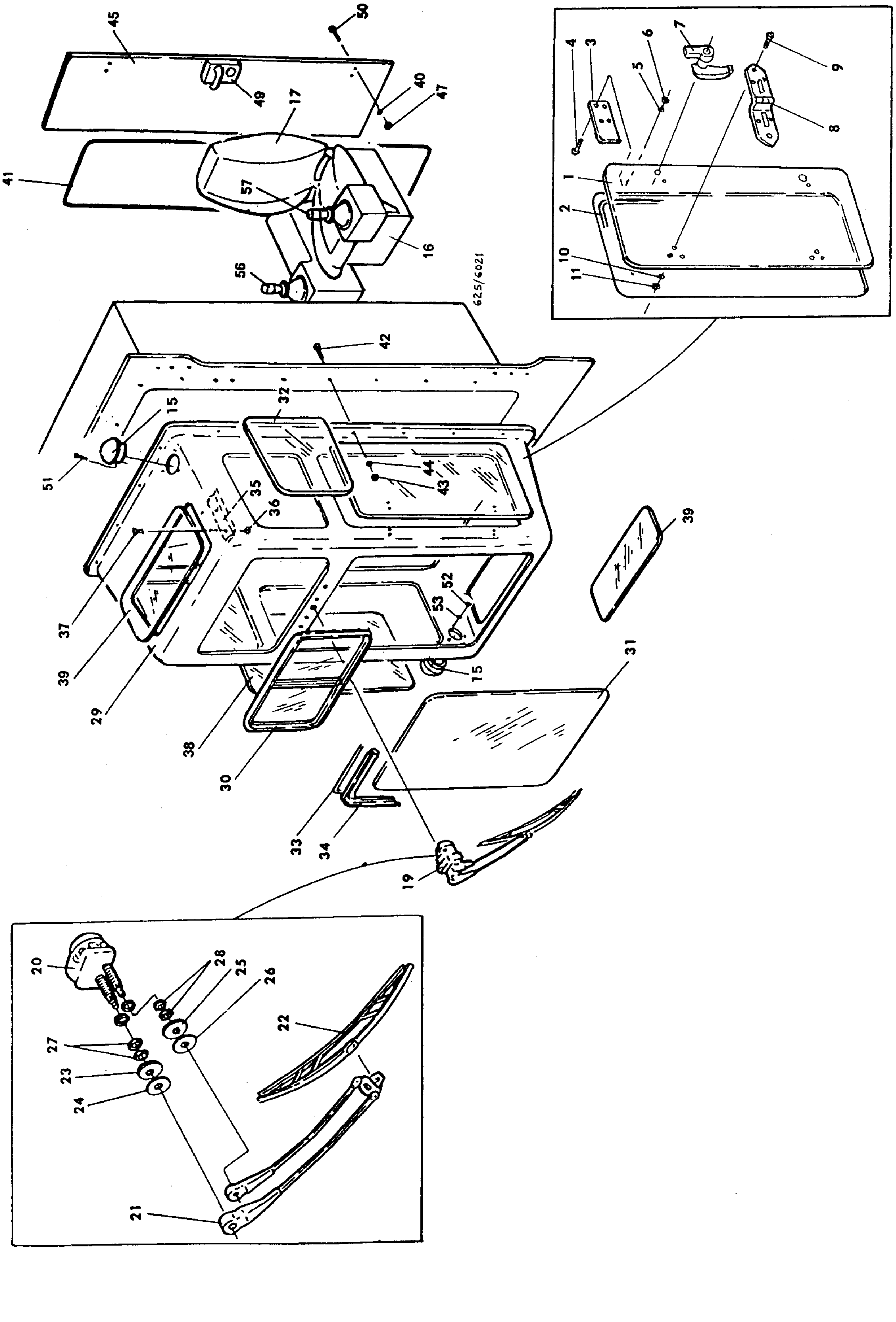

CAB

Item	Qty	Article no	Description	Supplementary data
000	1	188 0375-801	CAB	
001	1	489 5671-001	. WINDOW	
002	1	1856 2141-006	. RUBBER MOULDING	L = 2800 mm
003	2	489 5674-001	. PLATE	
004	8	2121 2844-378	. SCREW	MVBF 6 x 40 -A2-70
005	8	2151 2027-153	. WASHER	RB 6.4 x 12 -A4
006	8	2126 2634-116	. NUT	NYLOC-M6M 6 -A4-80
007	2	389 1581-001	. HANDLE	
008	2	489 5392-001	. HINGE	
009	12	2121 2636-331	. SCREW	MFS 5 x 20 -A4-80
010	12	2151 2027-146	. WASHER	RB 5.3 x 10 -A4
011	12	2126 2634-114	. NUT	M6M 5 -A4 -80
015	2	488 3152-801	. VENTILATOR, C	
016	1	114 1210-801	. DRIVER'S DESK	
017	1	488 6922-001	.. DRIVER'S SEAT	
019	1	387 6227-802	.. WINDSCREEN CLEANER	
020	1	6179 2151-280	... WIPER MOTOR	
021	1	6179 2152-500	... ARM	L = 500
022		6179 2153-700	... WIPER BLADE	L = 700 mm
023	2	488 3099-001	.. WASHER	
024	2	488 3099-002	.. RUBBER WASHER	
025	2	488 3098-001	.. WASHER	
026	2	488 3098-002	.. RUBBER WASHER	
027	2	6179 2151-617	. NUT	M20
028	2	6179 2151-624	. NUT	M16
029	1	288 1465-801	. CAB CUPOLA, ASSEMBLY	

CAB

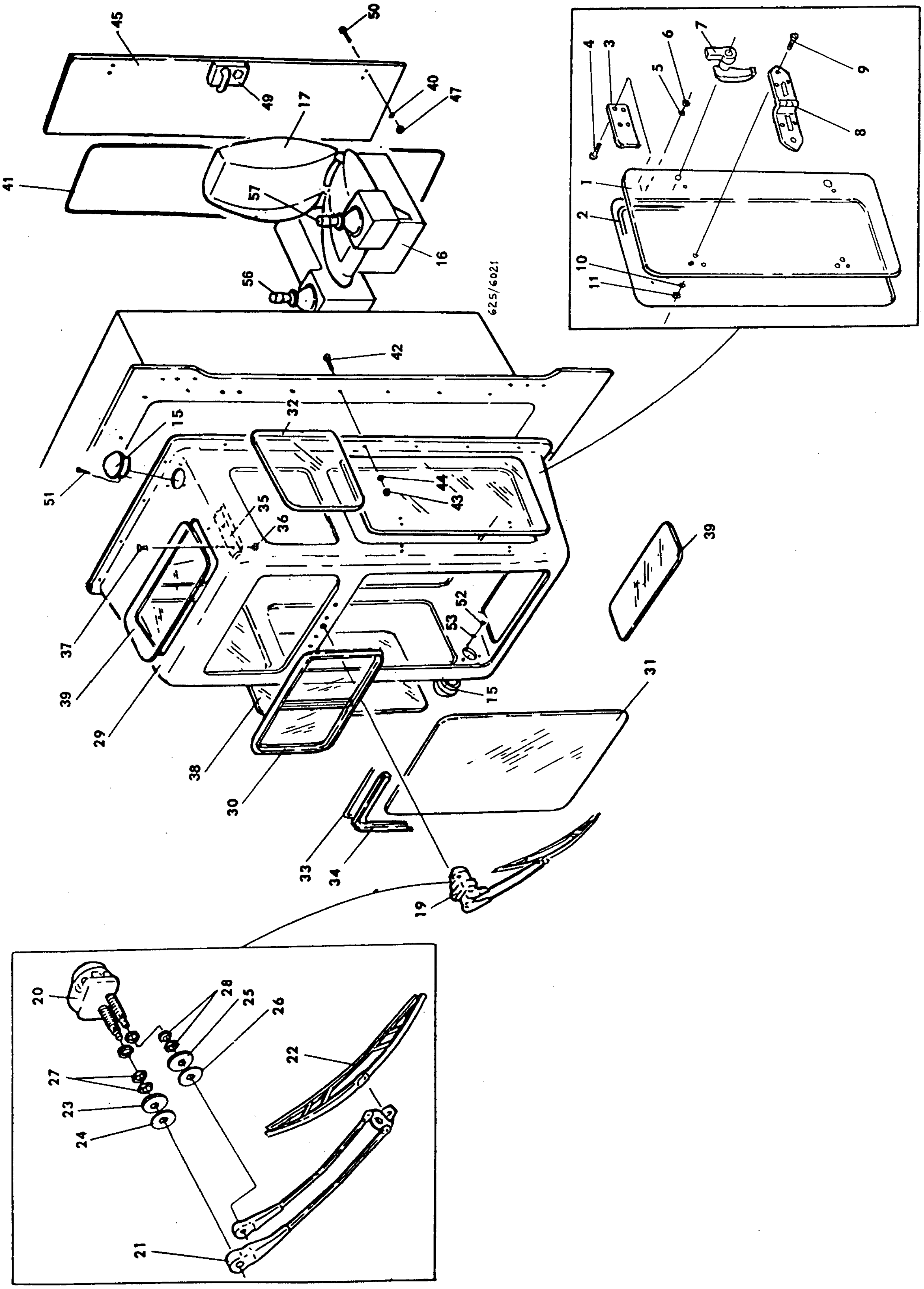
625/6021
1
2
3
4
5
6
7
8
9
10
11
15
16
17
19
20
21
22
23
24
25
26
27
28
29
30
31
32
33
34
35
36
37
38
39
40
41
42
43
44
45
47
49
50
51
52
53
56
57

CAB

Item	Qty	Article no	Description	Supplementary data
030	1	387 6167-803	.. SLIDE WINDOW	
031	1	388 0145-003	.. GLASS	
032	2	388 0145-004	.. GLASS	
033	1	487 2153-001	.. LOCKING LIST	L = 18000 mm
034	1	487 2152-001	.. RUBBER MOULDING	L = 10500 mm
035	2	489 3656-001	.. HOLDER	
036	13	2126 2034-116	.. NUT	NYLOC M6M 6 -A2-70
037	13	2121 2844-374	.. SCREW	MVBF 6 x 30 -A2 -70
038	1	388 0223-001	.. GLASS	
039	2	388 0145-002	.. GLASS	
040	12	2151 2022-153	. WASHER	BRB 6.4 x 12 G
041	1	1856 2141-006	. RUBBER MOULDING	L = 5310 mm
042	39	2121 2034-495	. SCREW	M6S 10 x 30 -A2-70
043	39	2126 2634-120	. NUT	NYLOC-M6M 10 -A2-70
044	39	2151 2027-173	. SPRING WASHER	RB 10.5 x 22 A4
045	1	287 8377-001	. DOOR	
047	18	2126 2634-116	. NUT	M6M 6 -A4-80
049	1	388 3233-801	. DOOR LOCK	
050	6	2121 2550-374	. SCREW	MC6S 6 x 30 A4 -80
051	6	2121 2250-338	. SCREW	MC6S 5 x 40 A4
052	6	2126 2634-114	. NUT	M6M 5 -A4 -80
053	6	2151 2027-146	. WASHER	RB 5.3 x 10 -A4
056	1		EL. CONTROLLER	SEE CONTENTS LIST, 9.5
057	1		EL. CONTROLLER	SEE CONTENTS LIST, 9.5

JIB TOP MOUNTING

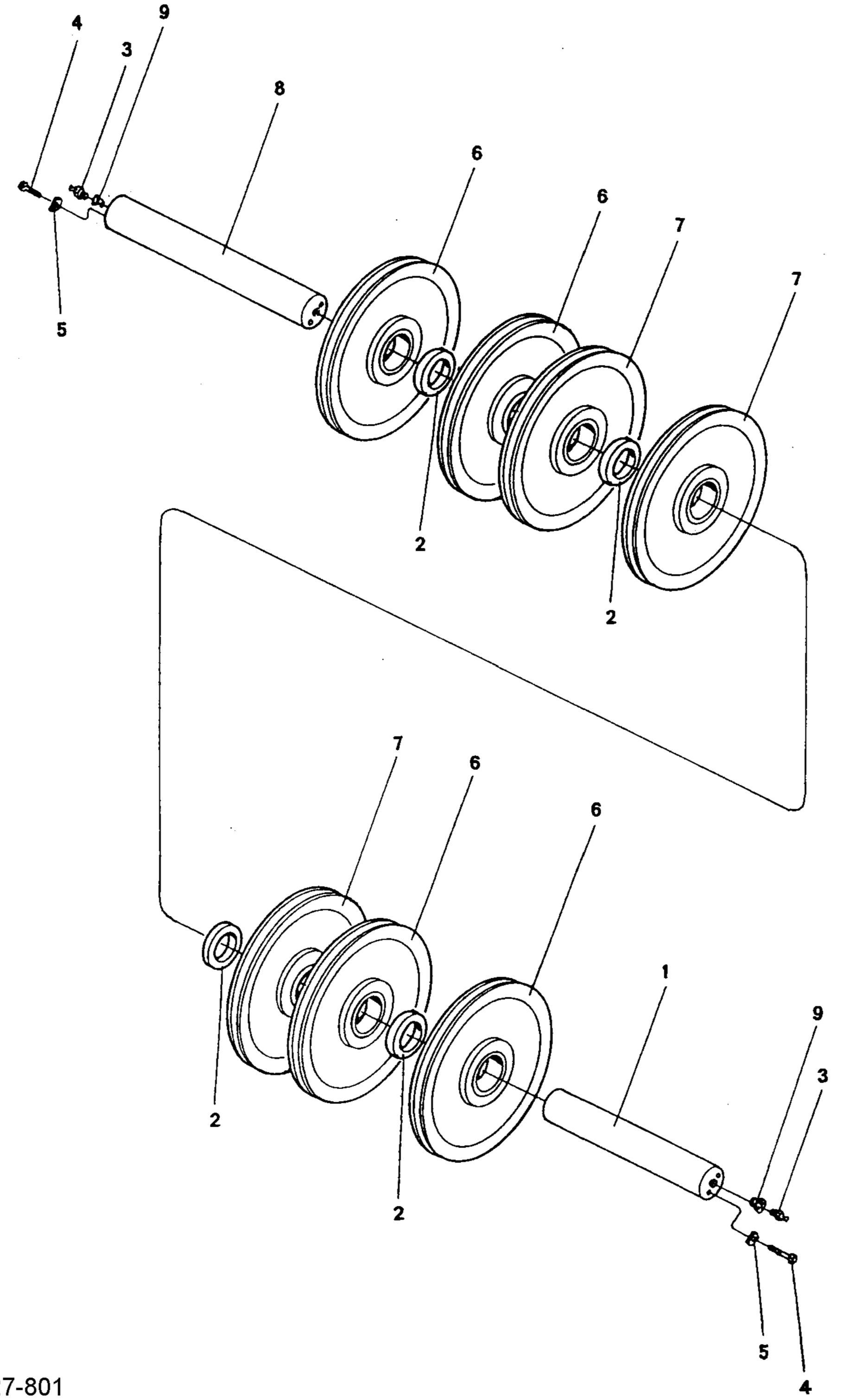

288 1527-801
625-6244.007

JIB TOP MOUNTING

Item	Qty	Article no	Description	Supplementary data
000	1	288 1527-801	JIB TOP MOUNTING	
001	1	389 1557-001	. SHAFT	
002	4	489 5666-001	. SPACER	
003	7	488 1677-002	. NIPPLE	
004	8	2121 2032-628	. SCREW	M6S 16 x 40 -8.8 FZB; DIN 933
005	8	489 5334-002	. LOCK WASHER	17 SSt
006	4	489 5708-801	. WIRE SHEAVE	
007	3	489 5707-801	. WIRE SHEAVE	
008	1	389 1554-001	. SHAFT	
009	7	2152 2151-181	. SEAL RING	

JIB BEARING MOUNTING

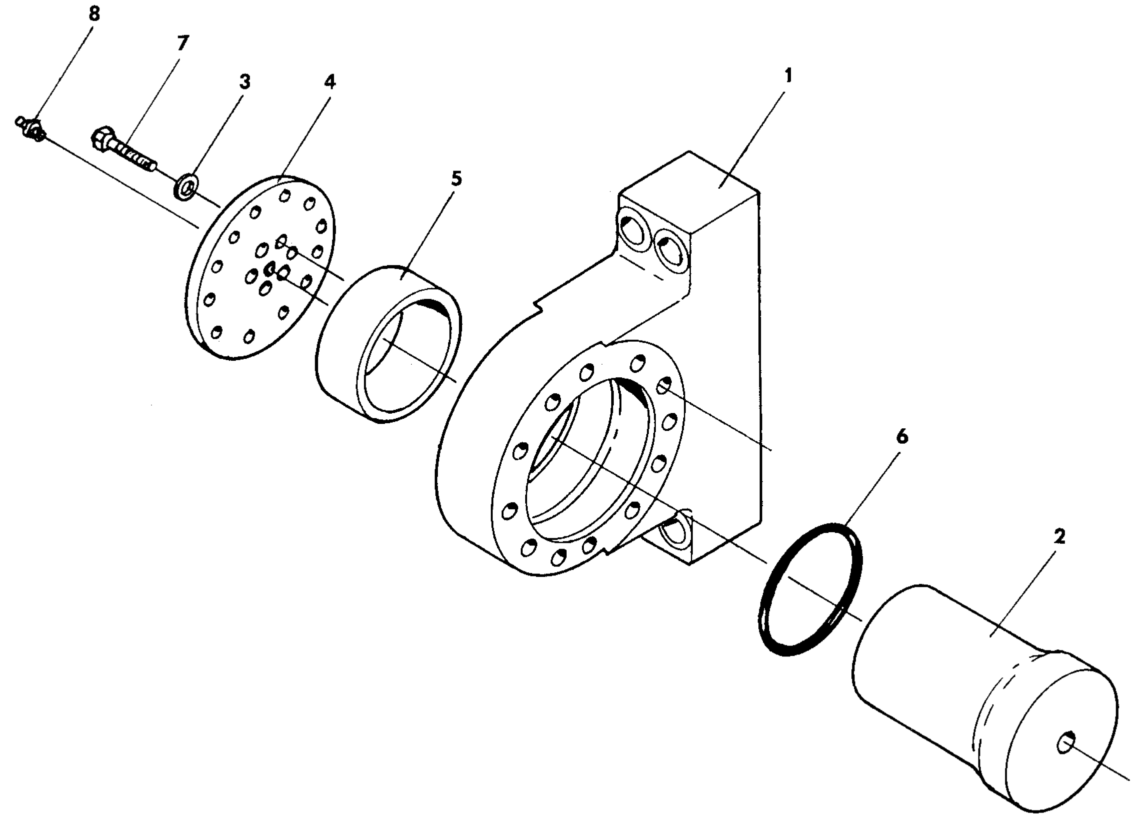

JIB BEARING MOUNTING

Item	Qty	Article no	Description	Supplementary data
000	1	389 1451-801	JIB BEARING MOUNTING	
001	1	389 1454-801	. JIB BEARING HOUSING	
002	1	389 1376-001	. SHAFT	
003	12	487 2826-002	. WASHER	
004	1	489 3303-001	. COVER	
005	1	489 3304-001	. SPACER RING	
006	2	2152 2115-533	. O-RING	
007	12	2121 2037-630	. SCREW	M6S 16 x 50 -10.9 G
008	1	2545 2011-025	. GREASE NIPPLE	AH M10 x 1

JIB BEARING HOUSING

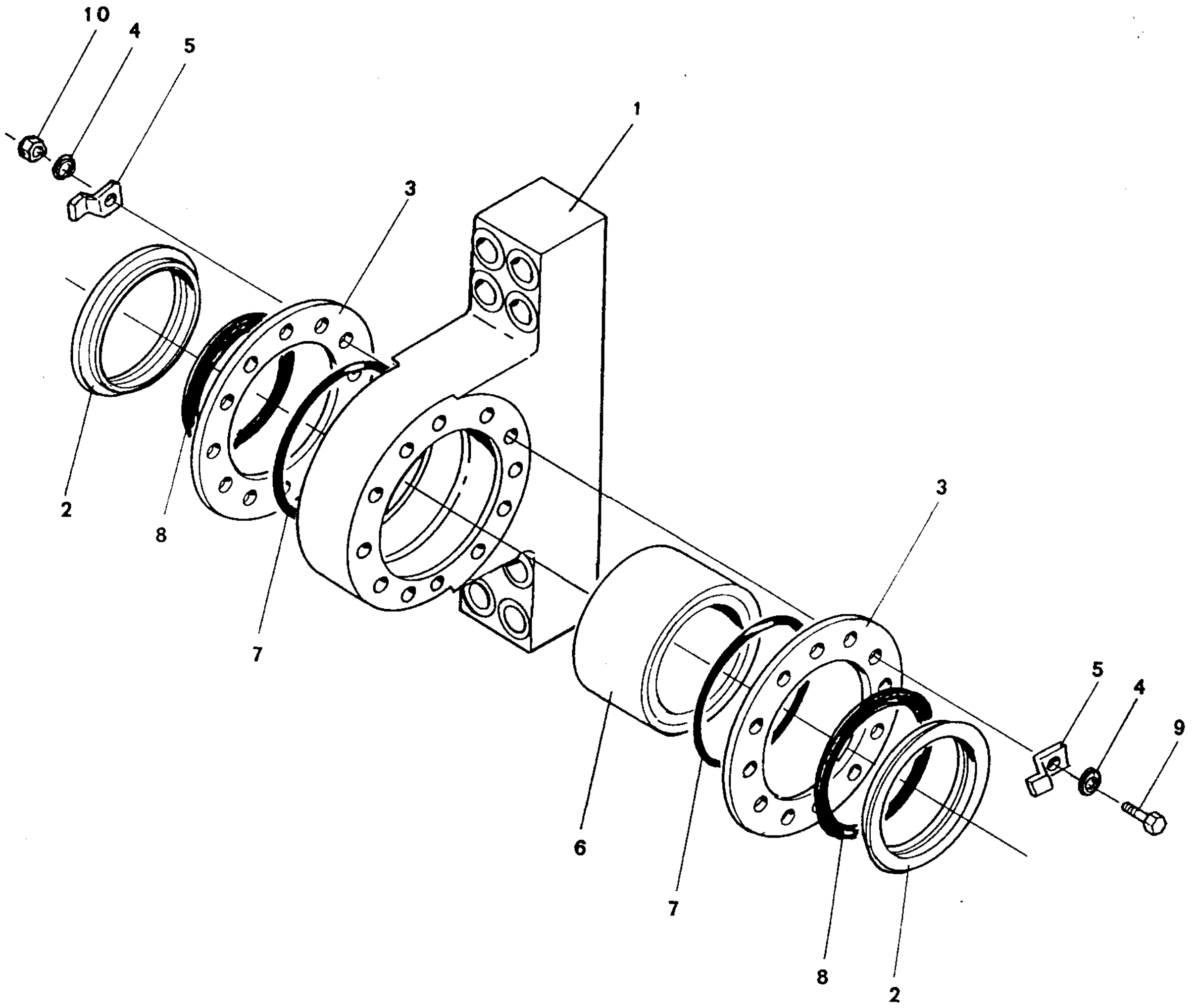

JIB BEARING HOUSING

Item	Qty	Article no	Description	Supplementary data
000	1	389 1454-801	JIB BEARING HOUSING	
001	1	287 8998-001	. BEARING HOUSE	
002	2	388 4795-001	. RING	
003	2	388 4796-001	. WASHER	
004	24	487 2826-002	. WASHER	
005	6	489 3362-001	. CLIP	
006	1	489 3305-801	. BEARING	
007	2	2152 2115-546	. O-RING	
008	2	2186 2187-200	. SEAL RING	
009	12	2121 2030-650	. SCREW	M6S 16 x 160 -10.9
010	12	2126 2038-124	. NUT	M6M 16 -10

CRANE JIB, YARD MOUNTING

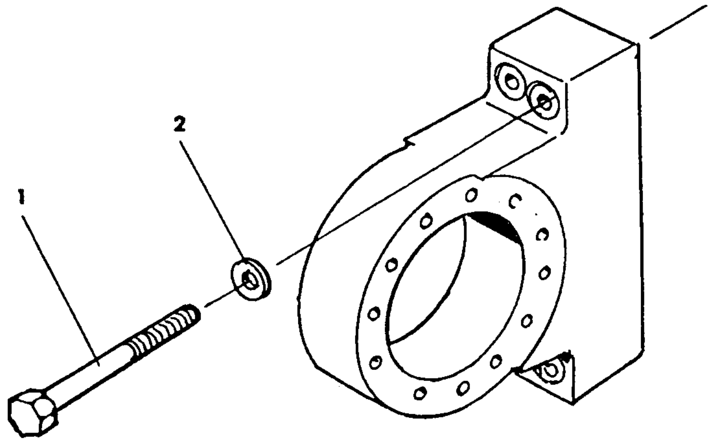

CRANE JIB, YARD MOUNTING

Item	Qty	Article no	Description	Supplementary data
000	1	489 5409-801	CRANE JIB, YARD MOUNTING	
001	8	388 7147-009	. SCREW	M6S 30 x 210 -10.9 spec.
002	8	488 8518-006	. WASHER	

Important!
When replacing genuine parts, an overload test of the complete unit has to be done. New certificate to be issued. See instruction 6.000.1 E.

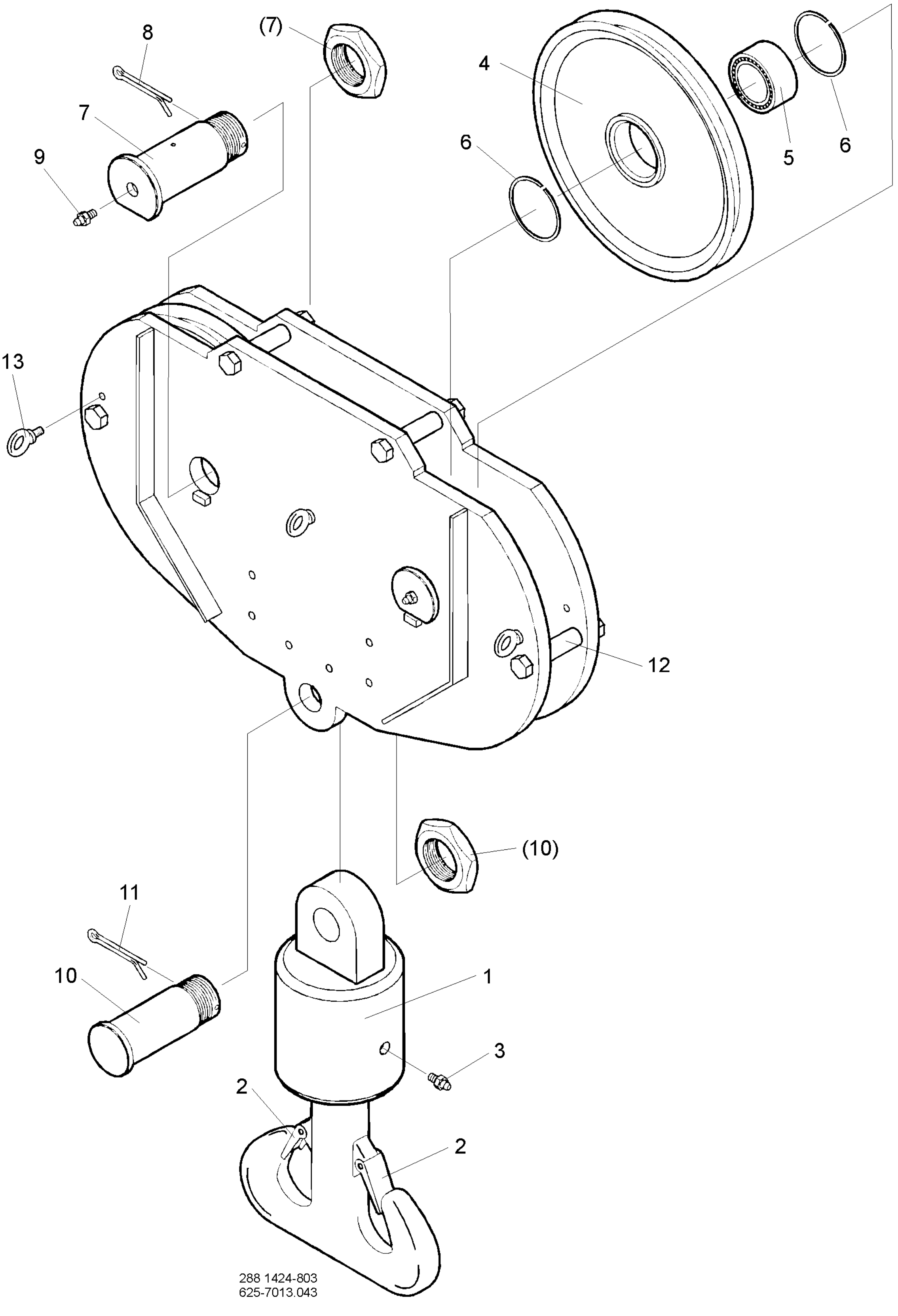

LIFTING BLOCK

Item	Qty	Article no	Description	Supplementary data
000	1	288 1424-803	LIFTING BLOCK	SWL 45 tonnes
001	1	875 12411-003	. SWIVEL WITH HOOK, COMPLETE	Incl. safety latch kit.
002	1	875 12064-001	.. SAFETY LATCH KIT	Incl. spring, screw, nut.
003	1	2545 2011-025	.. GREASE NIPPLE	M10 x 1; DIN 71412
004	2	875 12401-001	. SHEAVE, COMPLETE	Incl. bearing and lock rings.
005	2	875 12054-001	.. BEARING	
006	4	875 12062-001	.. LOCK RING	
007	2	875 12402-001	. SHAFT, COMPLETE	Incl. nut, split pin, nipple.
008	2	2114 2014-258	.. SPLIT PIN	8 x 120; DIN 94
009	2	2545 2011-025	.. GREASE NIPPLE	M10 x 1; DIN 71412
010	1	875 12403-001	. BOLT, COMPLETE	Incl. nut, split pin.
011	1	2114 2014-258	.. SPLIT PIN	8 x 120; DIN 94
012	4	875 12406-001	. SPACER, COMPLETE	Incl. spacer tube, screw, nut.
013	3	2183 2022-126	. EYE BOLT	M20; DIN 580

LIMIT SWITCHES BOX, HOISTING

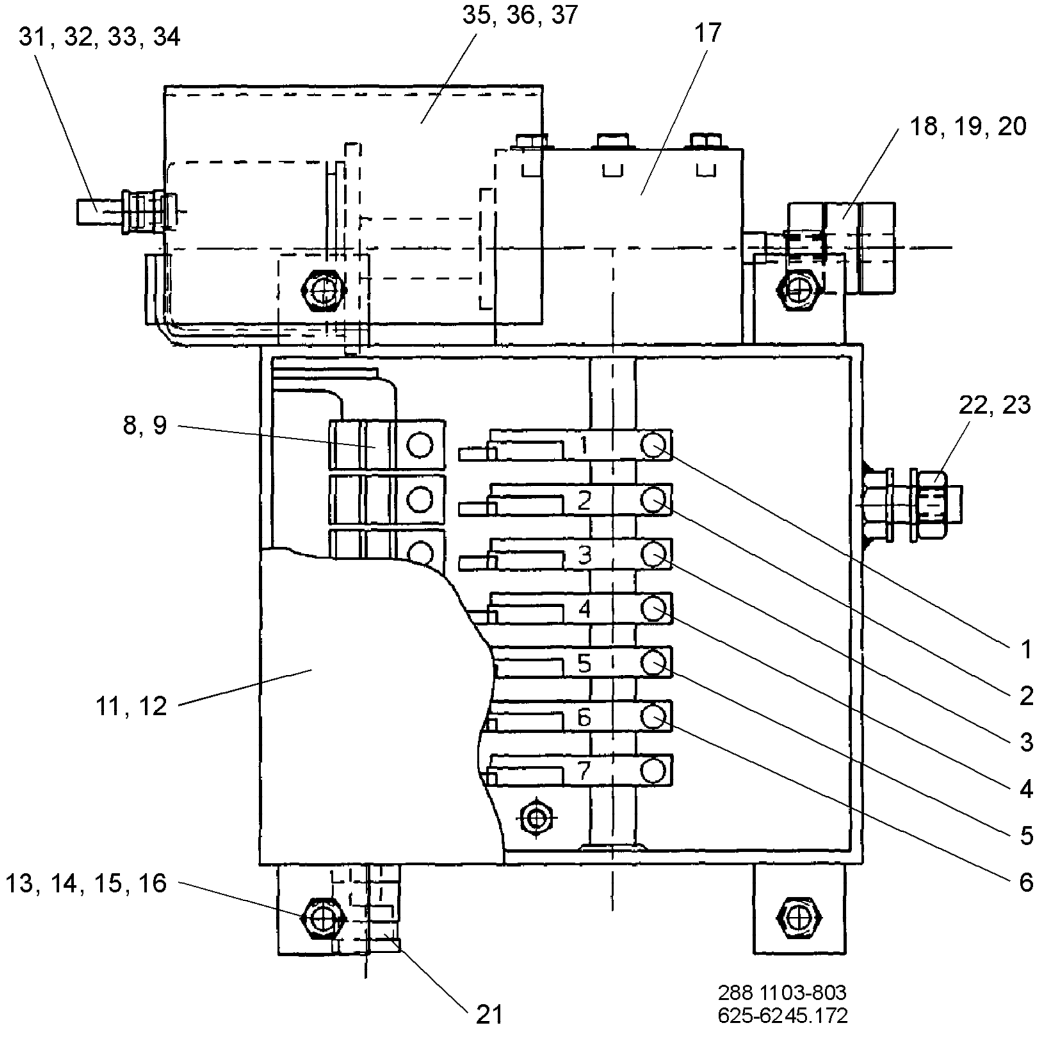

LIMIT SWITCHES BOX, HOISTING

Item	Qty	Article no	Description	Supplementary data
000	1	288 1103-803	LIMIT SWITCHES BOX, HOISTING	
001	1	875 15005-001	. CAM DISC WITH ARM, COMPLETE	10°
002	1	875 15005-002	. CAM DISC WITH ARM, COMPLETE	40°
003	1	875 15005-002	. CAM DISC WITH ARM, COMPLETE	40°
004	1	875 15005-001	. CAM DISC WITH ARM, COMPLETE	10°
005	1	875 15005-004	. CAM DISC WITH ARM, COMPLETE	80°
006	1	875 15005-003	. CAM DISC WITH ARM, COMPLETE	60°
008	6	875 15001-001	. MICRO SWITCH	1 A
009	2	875 15001-002	. THREADED STUD WITH NUTS	M3. Cut to right length.
011	1	875 15003-003	. COVER, COMPLETE	Incl. screws and sealing.
012	1	875 15003-002	.. SEALING	L = 1500 mm
013	4	2121 2034-457	.. SCREW	M6S 8 x 35 -A4-80
014	4	2126 2034-118	.. NUT	M6M 8 -A4 -80
015	4	2151 2027-164	.. WASHER	BRB 8.4 x 16 -A4
016	4	2126 2432-118	.. CAP NUT	MHM 8 -6 FZB
017	1	875 15012-002	. WORM GEAR, COMPLETE	Incl. screws. Ratio 40:1.
018	1	875 15006-002	. SHAFT COUPLING	
019	1	875 15008-001	. KEY	3 x 3 x 15
020	2	875 15009-001	. SCREW	SK6SS 6 x 8 - A2
021	2	875 15002-001	. CABLE GLAND WITH NUT	
022	2	2126 2634-122	. NUT	M6M 12 -A4 -80
023	4	2151 2022-178	. WASHER	BRB 13 x 24 FZB
031	1	414 4787-801	. ANGLE INDICATOR	
032	3	414 4809-001	. CLAMP	
033	3	2121 2254-226	. SCREW	MCS 3 x 12 - 4.8 FZB
034	1	2241 2101-120	. SHAFT COUPLING	
035	1	875 15010-001	. PROTECTIVE CAP	
036	2	2121 2032-447	. SCREW	M6S 8 x 12 -8.8 FZB
037	2	2151 2022-164	. WASHER	BRB 8.4 x 16 FZB; DIN 125A

LIMIT SWITCHES BOX, LUFFING

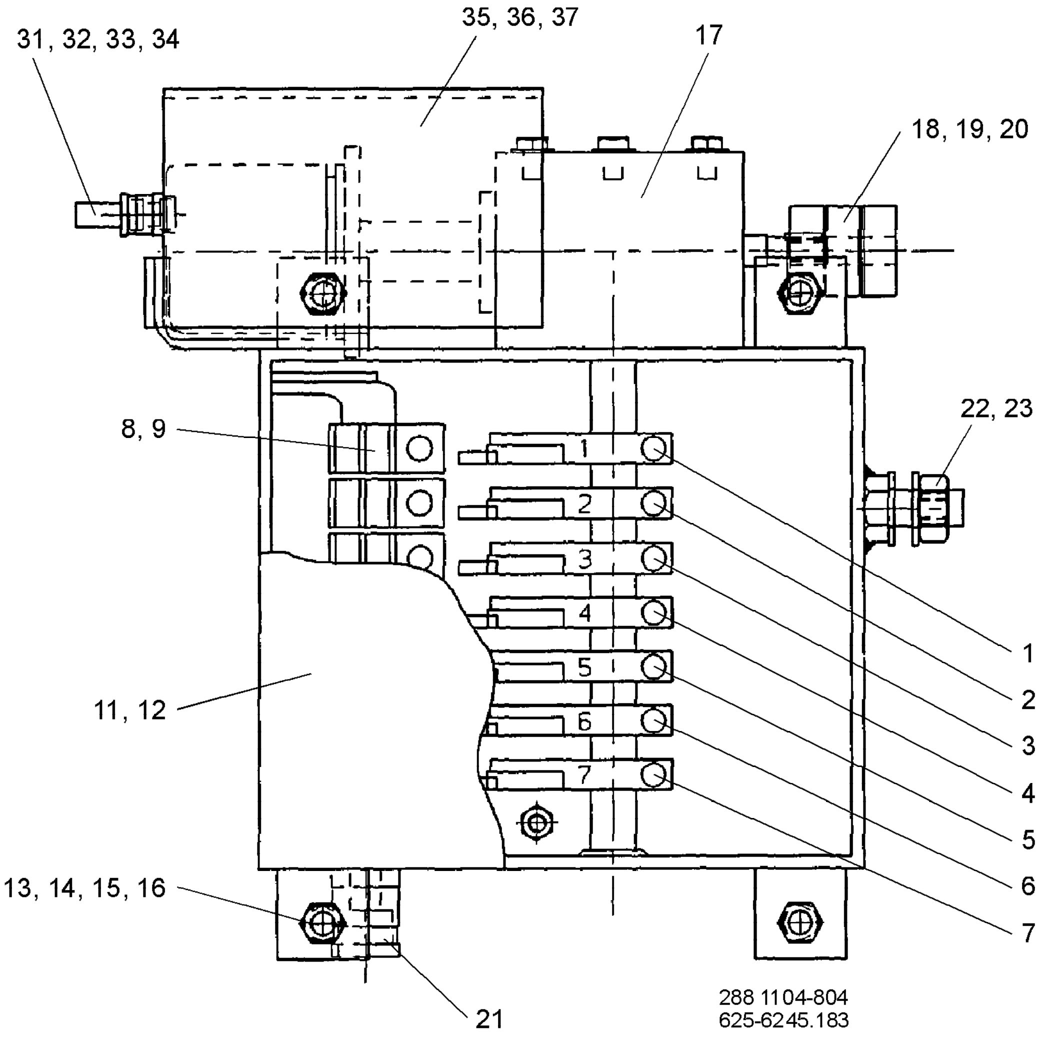

LIMIT SWITCHES BOX, LUFFING

Item	Qty	Article no	Description	Supplementary data
000	1	288 1104-804	LIMIT SWITCHES BOX, LUFFING	
001	1	875 15005-001	. CAM DISC WITH ARM, COMPLETE	10°
002	1	875 15005-007	. CAM DISC WITH ARM, COMPLETE	120°
003	1	875 15005-004	. CAM DISC WITH ARM, COMPLETE	80°
004	1	875 15005-008	. CAM DISC WITH ARM, COMPLETE	20°
005	1	875 15005-001	. CAM DISC WITH ARM, COMPLETE	10°
006	1	875 15005-011	. CAM DISC WITH ARM, COMPLETE	130°
007	1	875 15005-009	. CAM DISC WITH ARM, COMPLETE	100°
008	7	875 15001-001	. MICRO SWITCH	1 A
009	2	875 15001-002	. THREADED STUD WITH NUTS	M3. Cut to right length.
011	1	875 15003-003	. COVER, COMPLETE	Incl. screws and sealing.
012	1	875 15003-002	.. SEALING	L = 1500 mm
013	4	2121 2034-457	.. SCREW	M6S 8 x 35 -A4-80
014	4	2126 2034-118	.. NUT	M6M 8 -A4 -80
015	4	2151 2027-164	.. WASHER	BRB 8.4 x 16 -A4
016	4	2126 2432-118	.. CAP NUT	MHM 8 -6 FZB
017	1	875 15012-002	. WORM GEAR, COMPLETE	Incl. screws. Ratio 40:1.
018	1	875 15006-002	. SHAFT COUPLING	
019	1	875 15008-001	. KEY	3 x 3 x 15
020	2	875 15009-001	. SCREW	SK6SS 6 x 8 - A2
021	2	875 15002-001	. CABLE GLAND WITH NUT	
022	2	2126 2634-122	. NUT	M6M 12 -A4 -80
023	4	2151 2022-178	. WASHER	BRB 13 x 24 FZB
031	1	414 4787-801	. ANGLE INDICATOR	
032	3	414 4809-001	. CLAMP	
033	3	2121 2254-226	. SCREW	MCS 3 x 12 - 4.8 FZB
034	1	2241 2101-120	. SHAFT COUPLING	
035	1	875 15010-001	. PROTECTIVE CAP	
036	2	2121 2032-447	. SCREW	M6S 8 x 12 -8.8 FZB
037	2	2151 2022-164	. WASHER	BRB 8.4 x 16 FZB; DIN 125A

INCLINOMETER, C

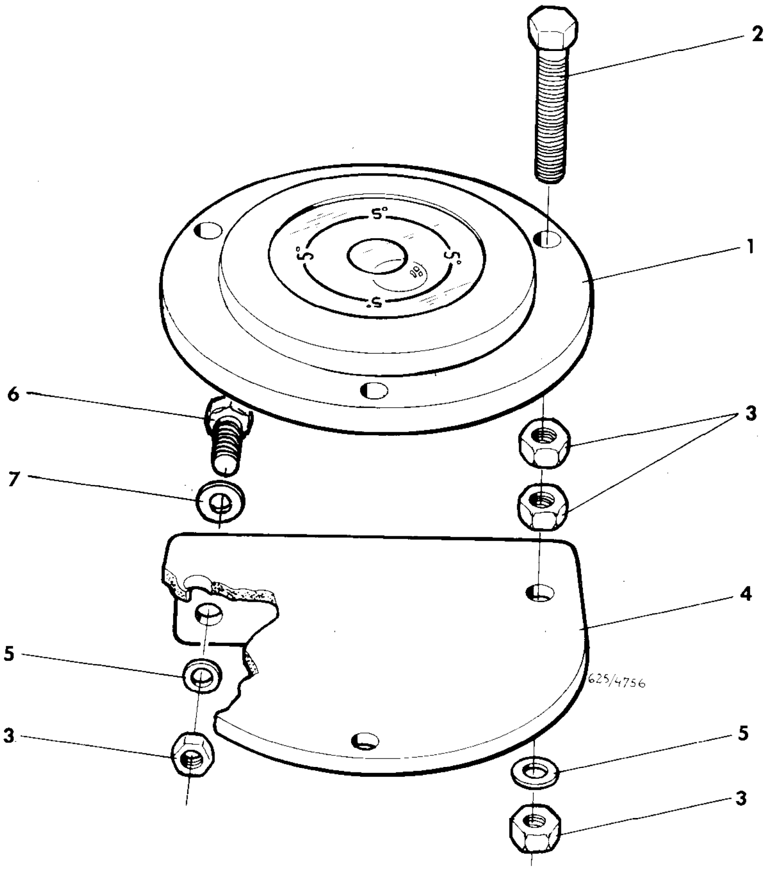

INCLINOMETER, C

Item	Qty	Article no	Description	Supplementary data
000	1	388 0176-801	INCLINOMETER, C	
001	1	488 7233-001	. INCLINOMETER	
002	3	2121 2254-378	. SCREW	MCS 6 x 40 -5.8 G
003	11	2126 2032-116	. NUT	M6M 6 -8 FZB
004	1	488 7511-001	. BRACKET	
005	5	2151 2022-153	. WASHER	BRB 6.4 x 12 G
006	2	2121 2034-370	. SCREW	M6S 6 x 20 -A2-70
007	2	2151 2025-153	. WASHER	BRB 6.4 x 12 SSt

TOL. SYSTEM ACC. TO ISO	General tol. for dimensions without tol. indications
Surface roughness R_a in µm	Machining ISO 2768 tol-class:
	Welding DIN 8570 tol-class:

Dragföljd/Tightening sequence

Kranens framsida/Front of the crane

Krantyp/Crane type
GL3628-2S

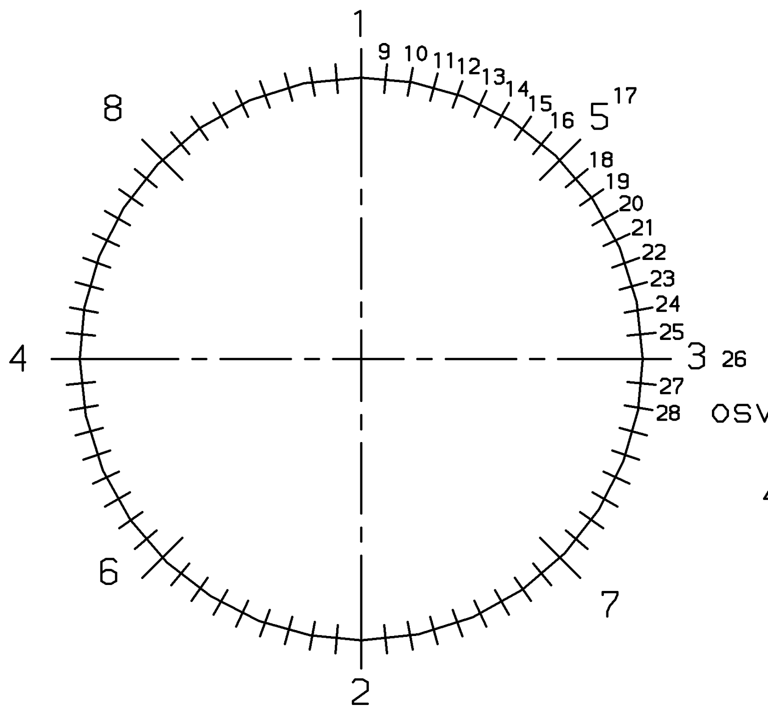

Scale 1:5

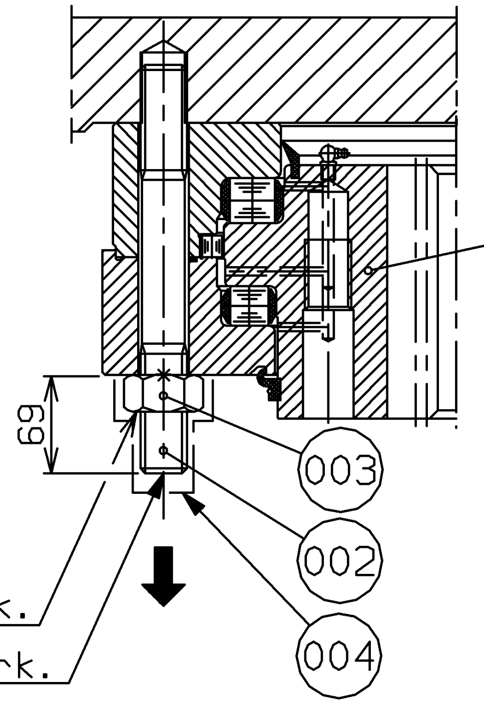

⚠A Muttermärkn./Nut mark.

Skruvmärkn./Screw mark.

Förspännings kraft 564 kN
med hydraulisk skruvsträckare
Prestressing force 564 kN
with hydraulic screw tensioner

MONTERINGSANVISNING/ASSEMBLY INSTRUCTION

1. Kontaktytorna rengjorda före montering
   Skruvmärkning i mutterända
   Contact surfaces cleaned before mounting
   Screw mark at nut end
2. Skruvarna dragna med hydraulisk skruv-
   sträckare,dragföljd enl.fig.
   The bolts loaded with a hydraulic ten-
   sioner,tightening sequence acc. to fig.
3. Dragningen upprepad på de först dragna
   bultarna tills alla bultarna har rätt
   spännkraft
   The tightening repeated at first tensioned
   bolts until all bolts have the correct pre-
   stressing force
4. Placering av härdsläpp (S) alt kulvändkrans
   med pluggat hål
   Placing of hardening stress (S) alt slewing rim
   with pluged hole
5. ⚠B General instruction: Acc. to MGC M2096-017E

Item no.	Qty/801	Article no.	Description (own language)	Dimensions	Description (English)
⚠B 004	xx	1176 2060-001	Hempinol 10220		Paint
003	90	2126 2039-131	M6M 33-10-spec		Hexagon nut
002	90	388 7196-002	Pinnskruv L=290		Stud
001	1	288 1508-801	Vändkranslager		Slewing bearing

RevInd	Revision	Appd	Year	Week
B	386211	BWG	00	50
A	383463	TLG	97	32

Design checked by	Accepted by qual dept	Part of: 188 0323	
Drawing checked by: BWG	Accepted for prod by	Specification	Scale: 1:20
Dept: 413	Drawn by: H Nylander	Year Week: 97 12	Description (English): Slewing bearing mount.	Weight kg: 2510
		Description (own language): Vändkranslager mont.	Prod.group: 628
		Drwg no.: 389 1628	Rev ind: B; Sheet: 1; No of sh: 1

MacGREGOR HÄGGLUNDS
MEMBER OF THE INCENTIVE GROUP

Reference: KR97012

Similar drawing no.: 388 9928

This document must not be copied without our written permission, and the contents thereof must not be imparted to a third party nor be used for any unauthorized purpose. Contravention will be prosecuted.

TOL. SYSTEM ACC. TO ISO	General tol. for dimensions without tol. indications
Surface roughness R_a in µm	Machining ISO 2768 tol-class: Welding DIN 8570 tol-class:

20 smörjnipplar CM10x1 DIN 71412 jämnt fördelade
20 grease nipples CM10x1 DIN 71412 equivalent grade

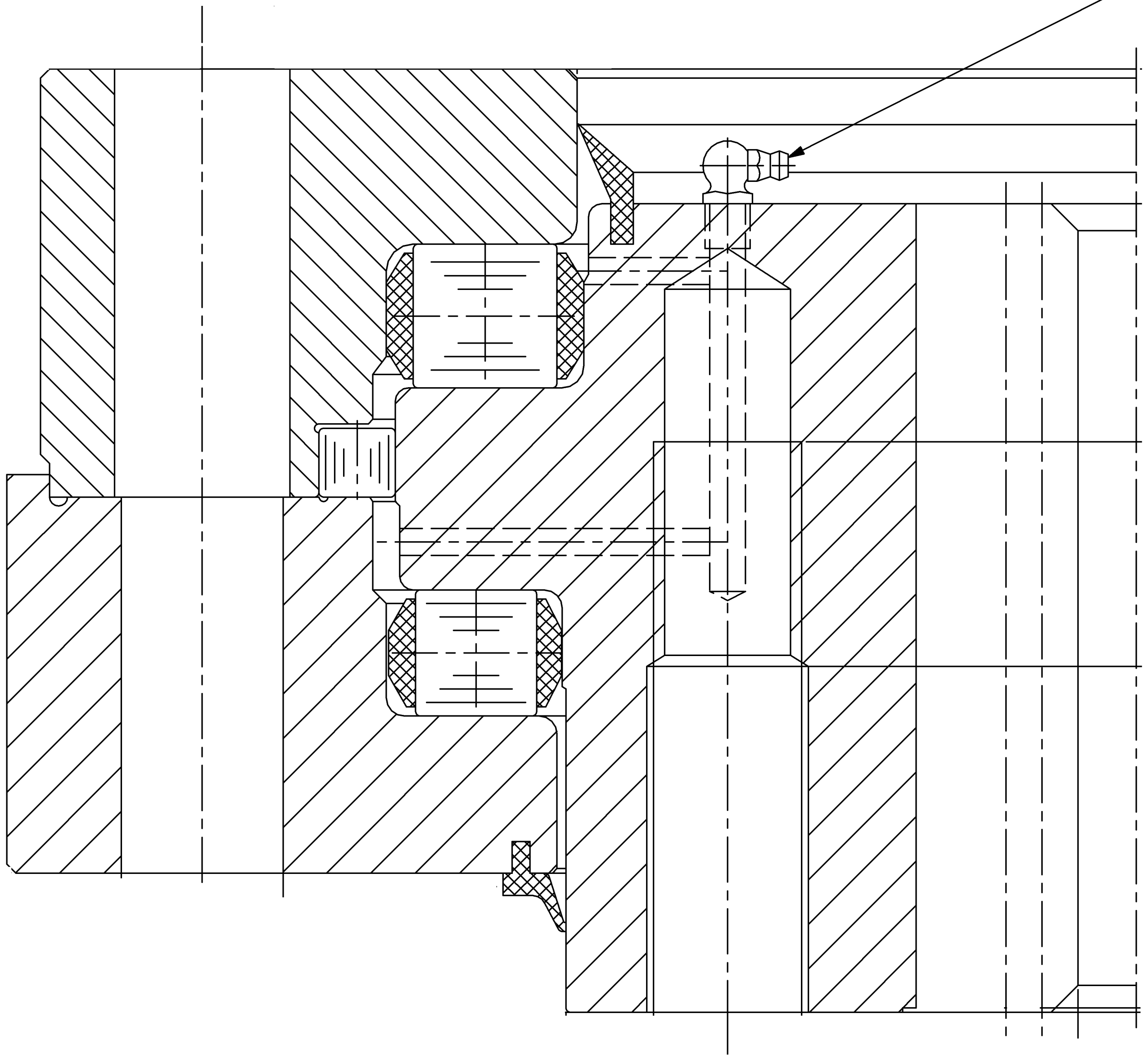

This document must not be copied without our written permission, and the contents thereof must not be imparted to a third party nor be used for any unauthorized purpose. Contravention will be prosecuted.

Reference KR97012

Similar drawing No. 288 1093

RevInd	Revision	Appd	Year	Week
C	386308	BWG	01	08
B	386030	BWG	00	37
A	383449	HNR	97	32

Item no.	Qty/801	Article no.	Description (own language)	Dimensions	Description (English)

Design checked by	Accepted by qual dept	Part of 389 1628	
Drawing checked by BWG	Accepted for prod by	Specification	Scale 1:1
Dept 413	Drawn by H Nylander, Year Week 97 16	Description (English) Slewing bearing	Weight kg 2220
MacGREGOR HÄGGLUNDS MEMBER OF THE INCENTIVE GROUP		Description (own language) Vändkranslager	Prod.group 628
		Drwg no. 288 1508	Rev ind C; Sheet 1; No of sh 1

Krantyp/Crane type
GL4028-2S

MONTERINGSANVISNING / ASSEMBLE INSTRUKTION

1. Kontaktytorna rengjorda före montering
   Skruvmärkning i mutterända
   Contact surfaces cleaned before mounting
   Screw mark at nut end

2. Skruvarna dragna med hydraulisk skruvsträckare, dragföljd enl. fig.
   The bolts loaded with a hydraulic tensioner, tightening sequense acc. to fig.

3. Dragningen upprepad på de först dragna bultarna tills alla bultarna har rätt spännkraft
   The tightening repeated at first tensioned bolts until all bolts have the correct prestressing forse

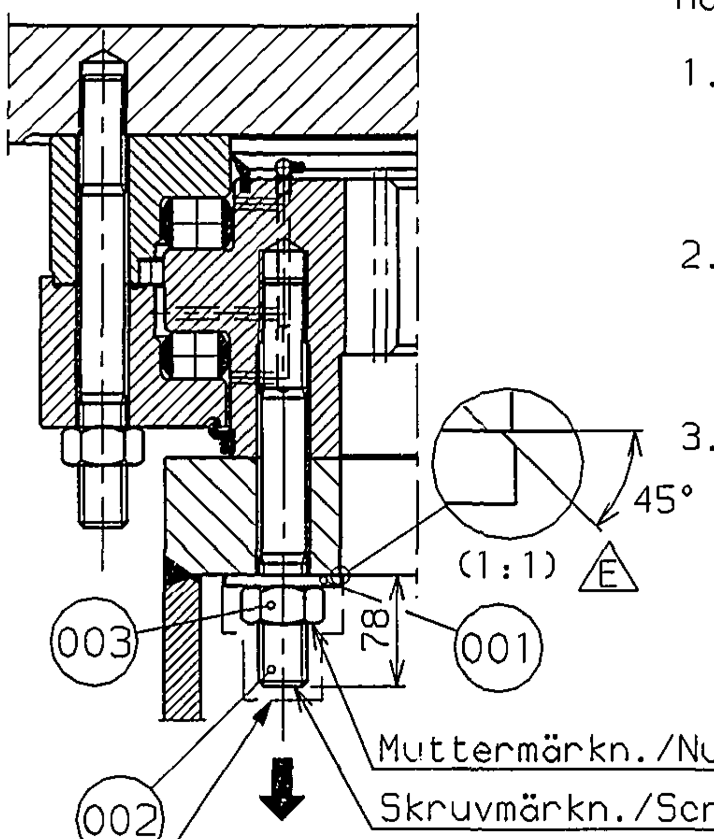

Dragföljd/Tightening sequence

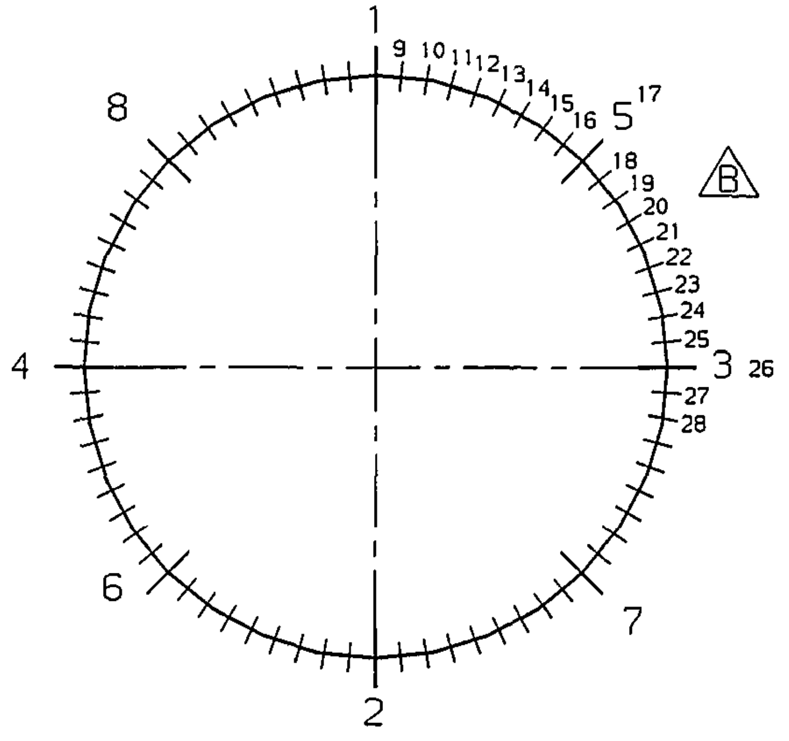

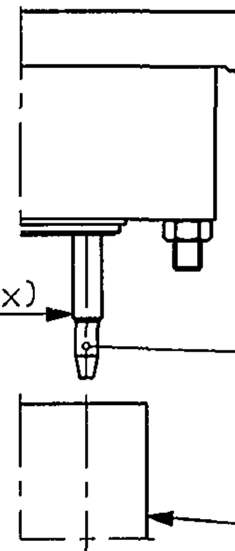

Förspännings kraft 564 kN
med hydraulisk skruvsträckare
Prestressing force 564 kN
with hydraulic screw tensioner

Rev	Item no.	Qty/801	Article no.	Description (own language)	Dimensions	Description (English)
B	006	x	387 4402-012	Styrpinne		Guide pin
	005	x	387 4402-010	Styrpinne		Guide pin
C	004	xx	1176 2123-001	Intertuf JBA 016		Paint
	003	90	2126 2039-131	M6M 33 -10 spec		Nut
B	002	90	388 7132-006	Pinnskruv	M33x280 -10.9 s	Stud
E	001	90	489 5960-002	Bricka		Washer

Revind	Revision	Appd	Year	Week
E	383693	HNR	97	44
D	383463	TLG	97	33
C	383194	ASN	97	13
B	382276	ASA	95	46
A	381822	ASN	95	11

Design checked by: | Accepted by qual dept: | Part of: Pack spec | Tol acc. to M 2002-020 Other not indicated tol. ± 2

Drawing checked by: SON | Accepted for prod by: DA | Specification: | Indicates R_a in µm. | Scale 1:5

Dept 413 | Drawn by S-O Nordell/ÅS | Year Week 95 02 | Description (English): Slewing rim yard | Weight kg 135

Description (own language): Vändkranslager varvsmont | Prod.group 628

MacGREGOR HÄGGLUNDS
MEMBER OF THE INCENTIVE GROUP

Drwg no. 388 7330 | Rev ind E | Sheet 1 | No of sh 1

Reference: KR 94027

Similar drawing no.: 388 7257

This document must not be copied without our written permission, and the contents thereof must not be imparted to a third party nor be used for any unauthorized purpose. Contravention will be prosecuted.

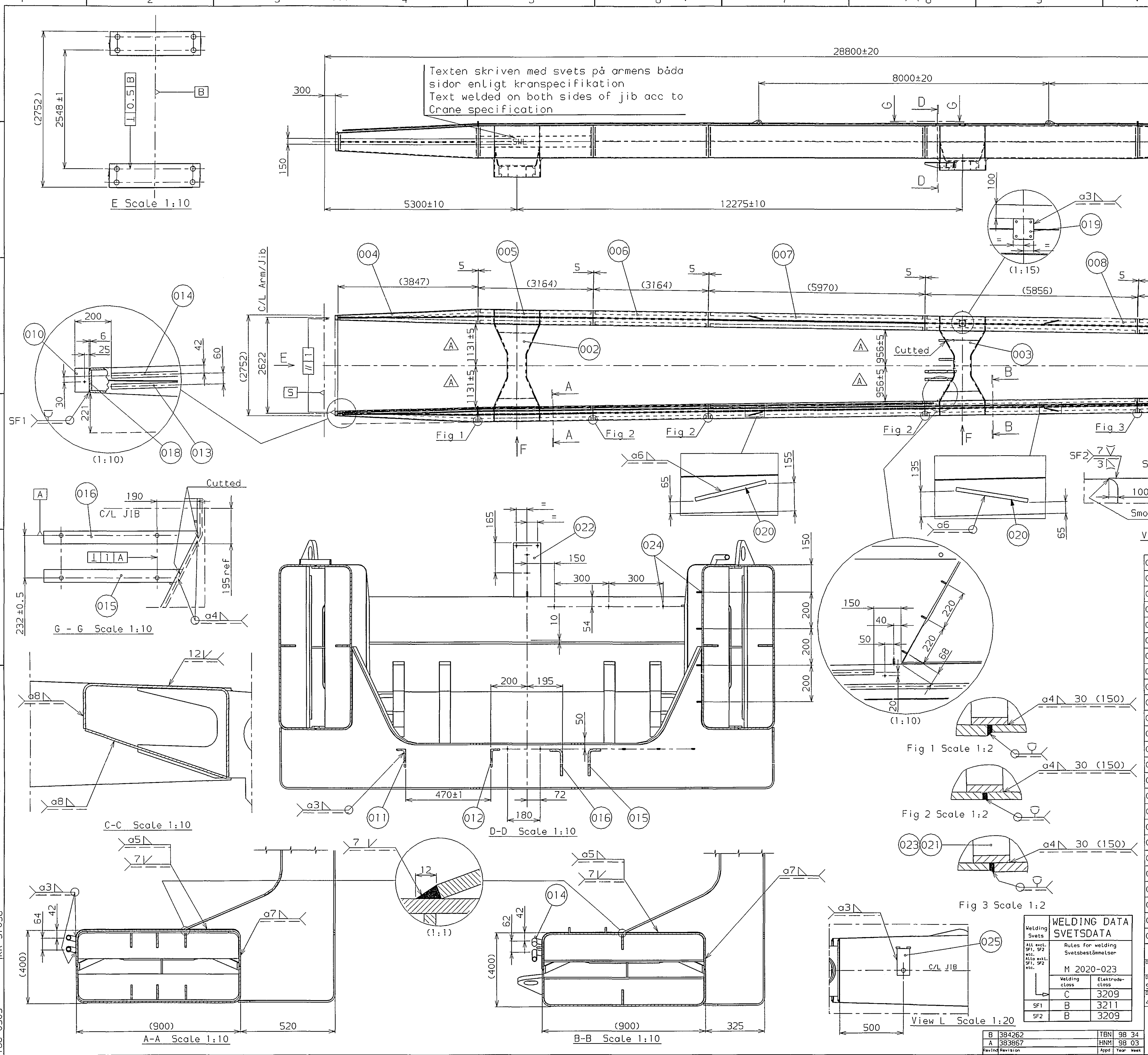

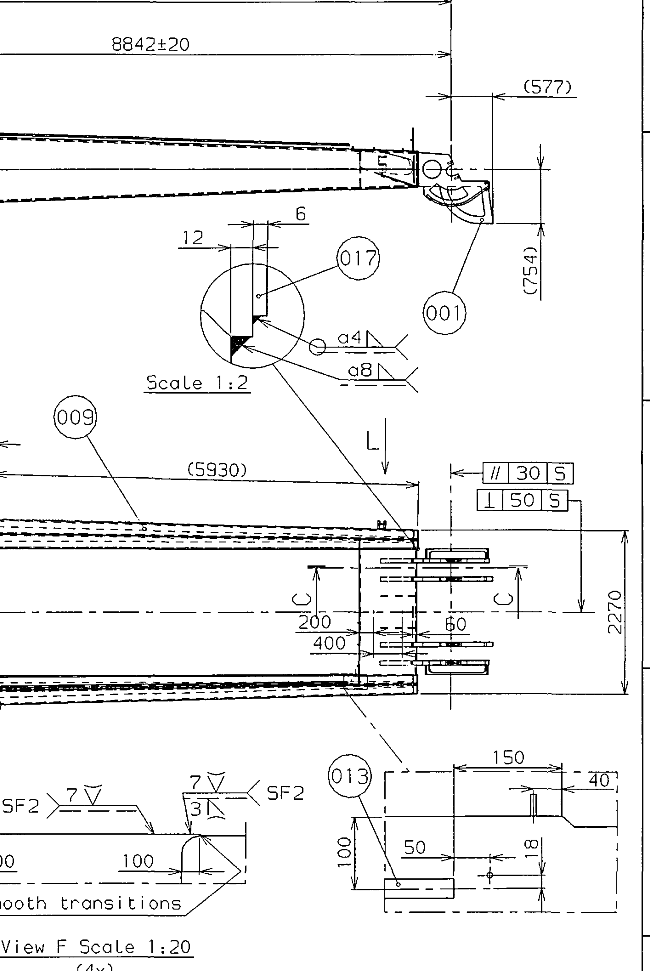

Item no.	Qty/801	Article no.	Description (own language)	Dimensions	Description (English)
025	1	387 8532-801	Fäste		Bracket
024	19	2129 2904-455	Svetsbult M8x30-A2-50		Weld bolt
023	1	1324 2251-321	Stål S235JRG2 8x40	L=16400	Bar
022	1	489 3312-001	Fäste		Bracket
021	40	489 2288-001	Skarvjärn		Splice bar
020	4	488 7612-001	Lyftöra		Ear
019	1	488 4633-001	Fäste		Bracket
018	2	388 3810-006	Distansplåt		Spacer plate
017	2	388 3810-004	Distansplåt		Spacer plate
016	1	388 8995-002	Fäste		Bracket
015	1	388 8995-001	Fäste		Bracket
014	1	387 6181-801	Kabelrör L=		Cable tube
013	1	387 6181-801	Kabelrör L=		Cable tube
012	1	387 1379-004	Strålkastarfäste		Headlight bracket
011	1	387 1379-003	Strålkastarfäste		Headlight bracket
010	2	388 5005-001	Fästplatta		Attaching plate
009	2	288 1639-801	Armprofil		Jib profile
008	2	288 1638-801	Armprofil		Jib profile
007	2	288 1637-801	Armprofil		Jib profile
006	2	288 1636-801	Armprofil		Jib profile
005	2	288 1635-801	Armprofil		Jib profile
004	2	288 1634-801	Armprofil		Jib profile
003	1	288 1885-801	Stag		Stay
002	1	288 1884-801	Stag		Stay
001	1	188 0787-801	Armtopp		Top of jib

Welding Svets	WELDING DATA SVETSDATA	
All excl. SF1, SF2 etc. Alla exkl. SF1, SF2 etc.	Rules for welding Svetsbestämmelser M 2020-023	
	Welding class	Elektrode-class
	C	3209
SF1	B	3211
SF2	B	3209

Rev Ind	Revision	Appd	Year	Week
B	384262	TBN	98	34
A	383867	HNM	98	03

Design checked by	Accepted by qual dept	Part of: Order spec	
Drawing checked by: SON	Accepted for prod by: DA	Specification	Scale: 1:50
Dept: 413 · Drawn by: H Nyström · Year Week: 97 36		Description (English): Jib welding	Weight kg: 12804
		Description (own language): Kranarm svets	Prod.group: 628
		Drwg no.: 188 0720	Rev ind: B · Sheet: 1 · No of sh: 1

MacGREGOR HÄGGLUNDS
MEMBER OF THE INCENTIVE GROUP

This document must not be copied without our written permission, and the contents thereof must not be imparted to a third party nor be used for any unauthorized purpose. Contravention will be prosecuted.

Reference KR 97038
Similar drawing No. 188 0505

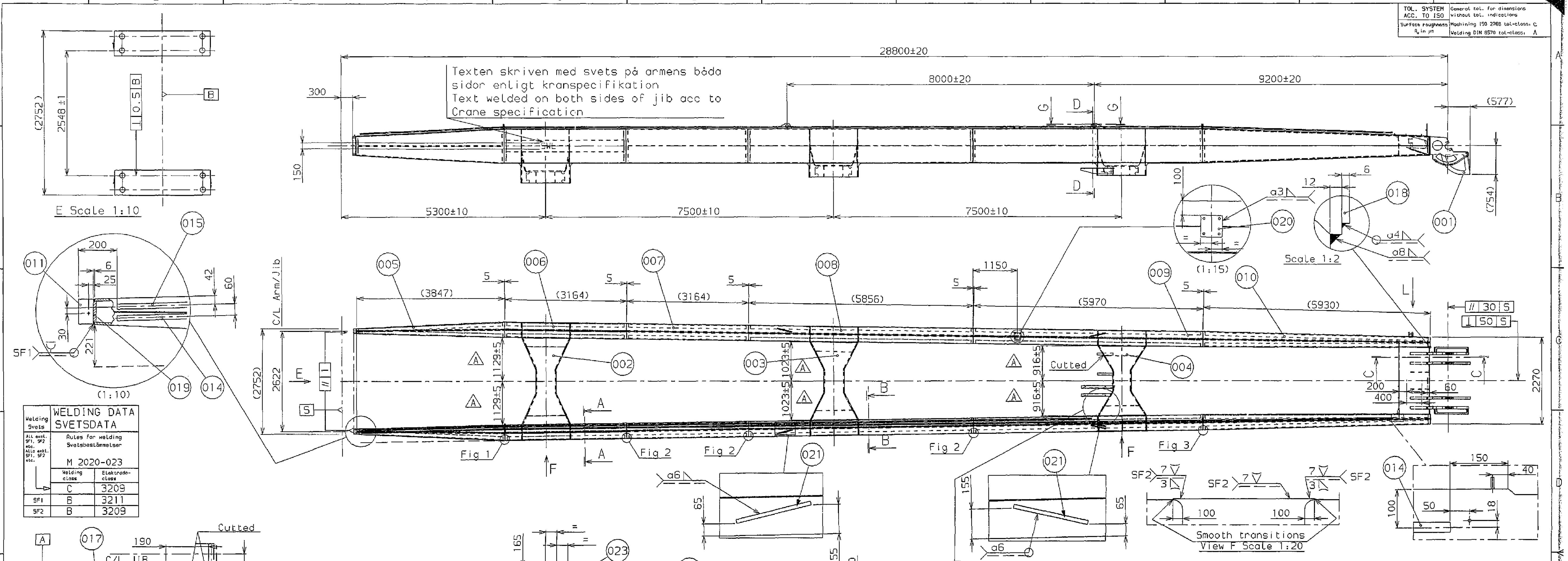

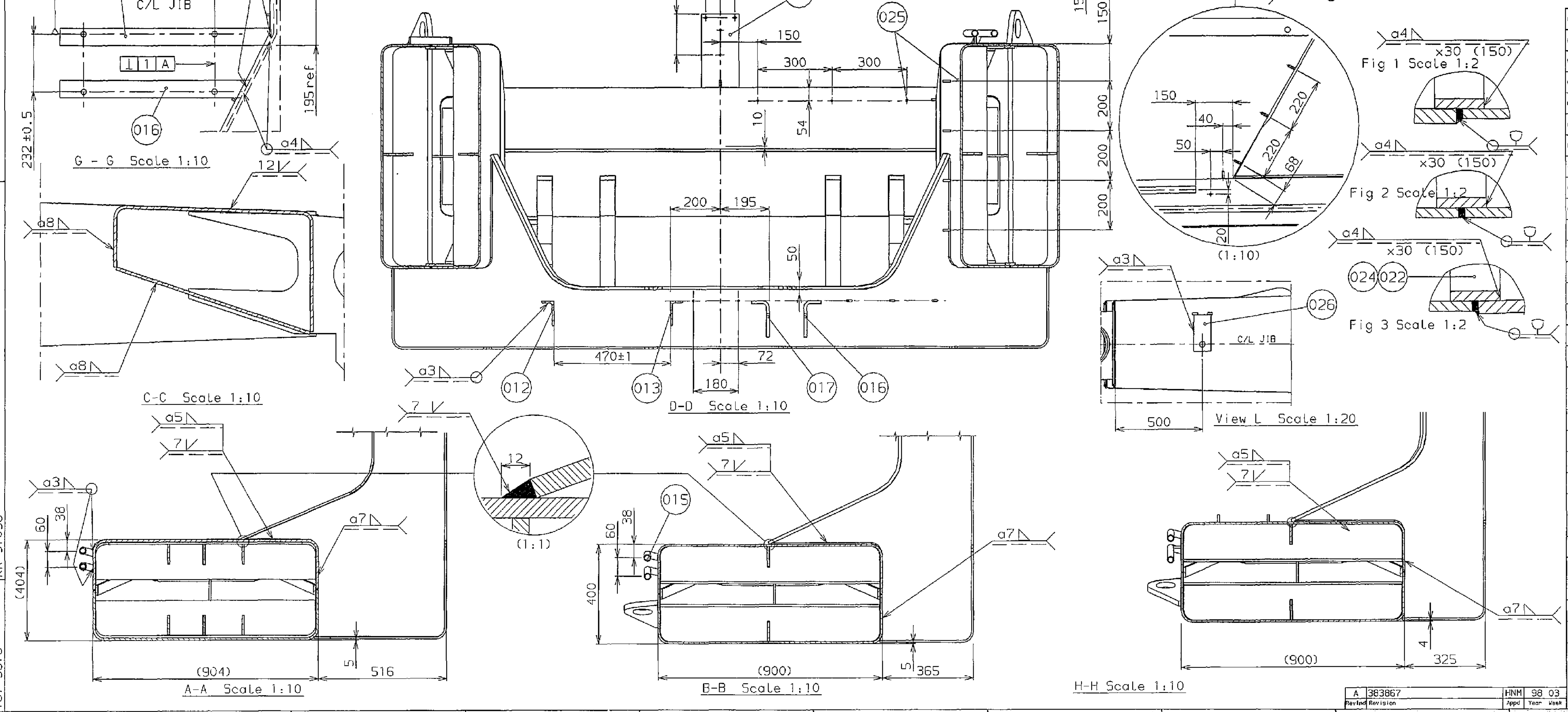

026	1	387 8532-801	Fäste		Bracket
025	19	2129 2904-455	Svetsbult M8x30-A2-50		Weld bolt
024	1	1324 2251-321	Stål S235JRG2 8x40	L=16400	Bar
023	1	489 3312-001	Fäste		Bracket
022	40	489 2288-001	Skarvjärn		Splice bar
021	4	488 7612-001	Lyftöra		Ear
020	1	488 4633-001	Fäste		Bracket
019	2	388 3810-006	Distansplåt		Spacer plate
018	2	388 3810-004	Distansplåt		Spacer plate
017	1	388 8995-002	Fäste		Bracket
016	1	388 8995-001	Fäste		Bracket
015	1	387 6181-801	Kabelrör L=19100		Cable tube
014	1	387 6181-801	Kabelrör L=27000		Cable tube
013	1	387 1379-004	Strålkastarfäste		Headlight bracket
012	1	387 1379-003	Strålkastarfäste		Headlight bracket
011	2	388 5005-001	Fästplatta		Attaching plate
010	2	288 1639-801	Armprofil		Jib profile
009	2	288 1927-801	Armprofil		Jib profile
008	2	288 1926-801	Armprofil		Jib profile
007	2	288 1636-801	Armprofil		Jib profile
006	2	288 1920-801	Armprofil		Jib profile
005	2	288 1634-801	Armprofil		Jib profile
004	1	288 1906-801	Stag		Stay
003	1	288 1905-801	Stag		Stay
002	1	288 1904-801	Stag		Stay
001	1	188 0787-801	Armtopp		Top of jib
Item no.	Qty/801	Article no.	Description (own language)	Dimensions	Description (English)

Design checked by	Accepted by qual dept	Part of: Order spec	
Drawing checked by: SON	Accepted for prod by: DA	Specification	Scale: 1:50
Dept: 413 · Drawn by: H Nyström · Year Week: 97 39		Description (English): Jib welding	Weight kg: 13860
		Description (own language): Kramarm svets	Prod.group: 628
MacGREGOR HÄGGLUNDS — MEMBER OF THE INCENTIVE GROUP		Drwg no.: 188 0750	Rev ind: A · Sheet: 1 · No of sh: 1

Rev Ind	Revision	Appd	Year	Week
A	383867	HNM	98	03

KR 97038

187 9679

TWO SPEED VALVE

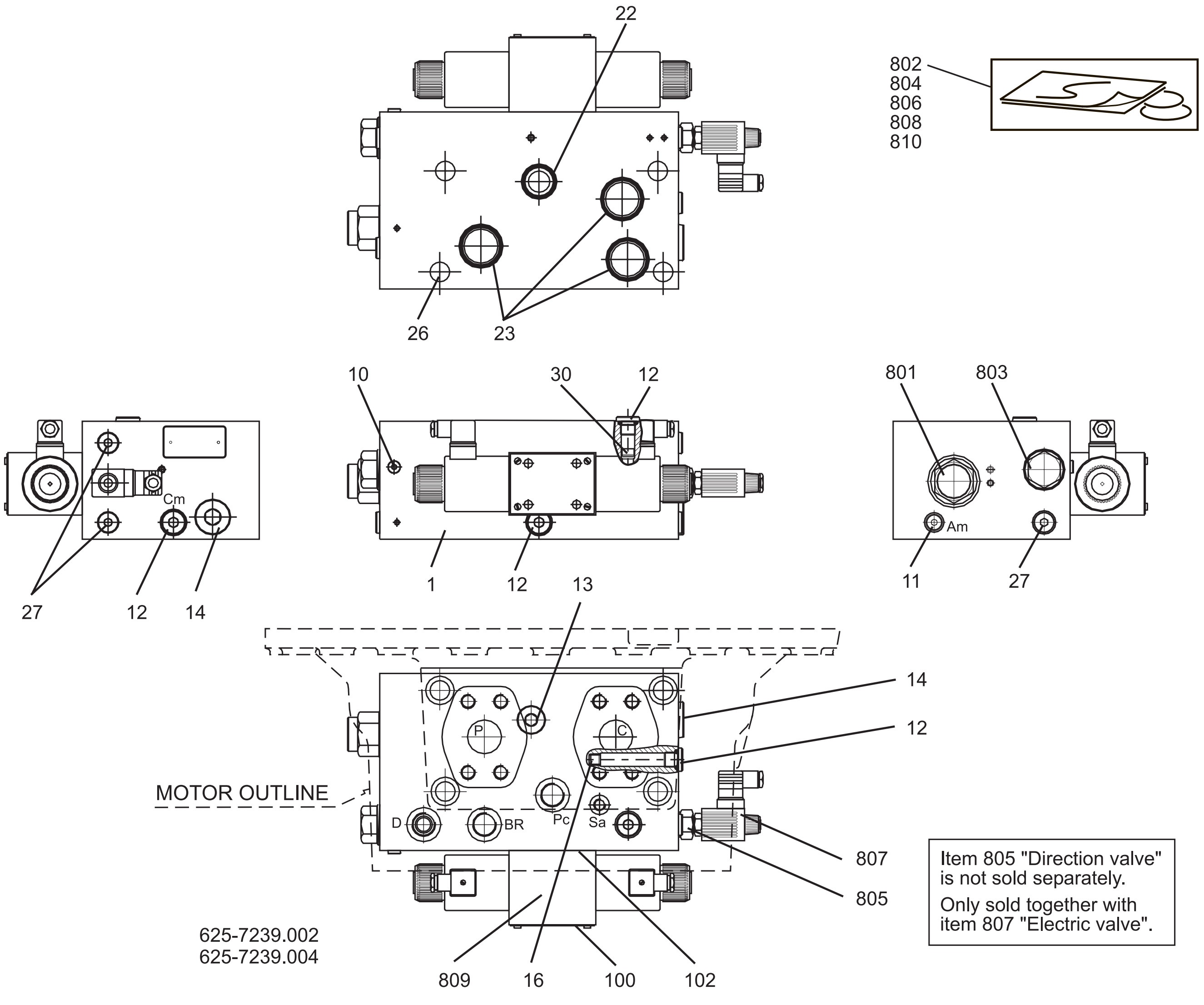

Item 805 "Direction valve" is not sold separately.
Only sold together with item 807 "Electric valve".

TWO SPEED VALVE

Item	Qty	Article no	Description	Supplementary data
000	1	178 2568-801	TWO SPEED VALVE	
001	1	178 2655-001	. HOUSING	
010	1	2522 2106-018	. PLUG WITH SEAL	
011	1	2522 2106-014	. PLUG WITH SEAL	
012	3	2522 2106-012	. PLUG WITH SEAL	
013	1	2522 2106-034	. PLUG WITH SEAL	
014	1	2522 2106-100	. PLUG WITH SEAL	
016	1	2522 2115-111	. PLUG	
022	1	2152 2101-940	. O-RING	
023	3	2152 2101-809	. O-RING	
026	4	2121 2541-691	. SCREW	
027	3	2522 2106-038	. PLUG WITH SEAL	
030	1	6524 2119-300	. CHECK VALVE	
100	4	2121 2541-378	. SCREW	MC6S 6 x 40 -12.9
102	4	2152 2115-803	. O-RING	
801	1	378 2193-801	. DIRECTION VALVE	
802	1	378 2193-901	.. GASKET SET	
803	1	478 3708-801	. CHECK VALVE	
804	1	478 3708-901	.. GASKET SET	
805	1	478 3746-801	. DIRECTION VALVE	Must be sold together with item 807
806	1	478 3746-901	.. GASKET SET	
807	1	478 3706-801	. ELECTRICAL VALVE	
808	1	478 3706-901	.. GASKET SET	
809	1	278 2134-801	. DIRECTION VALVE	
810	1	278 2134-901	.. GASKET SET	

FLUSH - UNLOADING UNIT

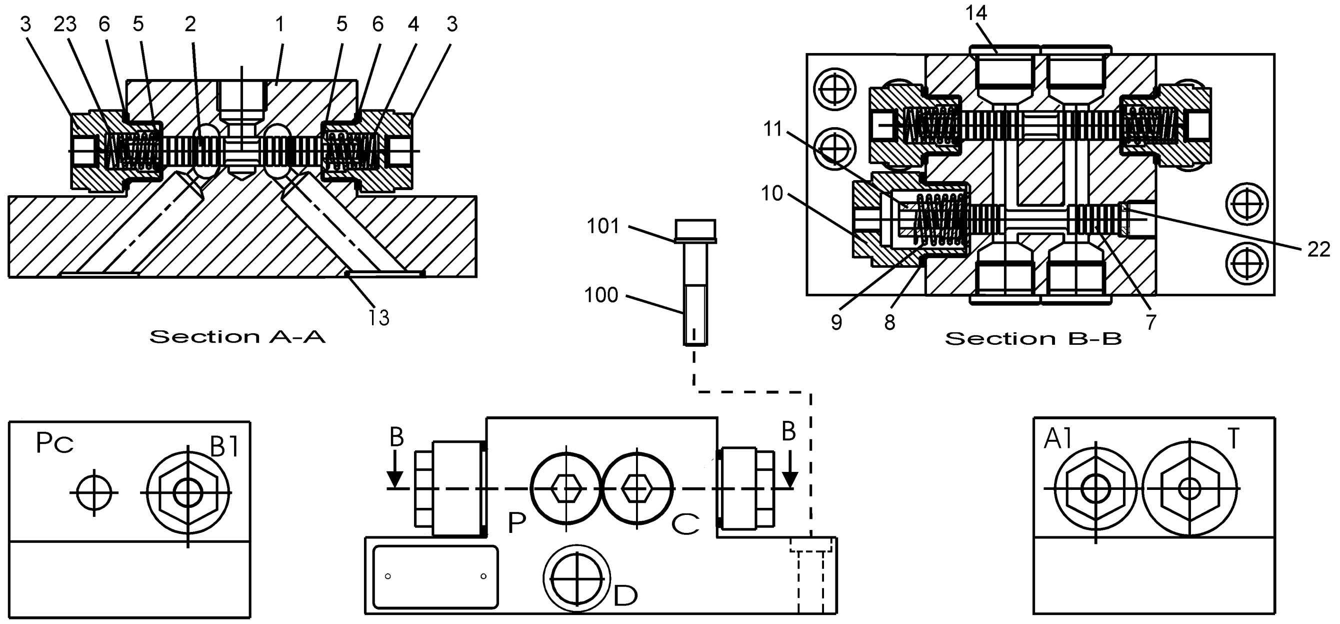

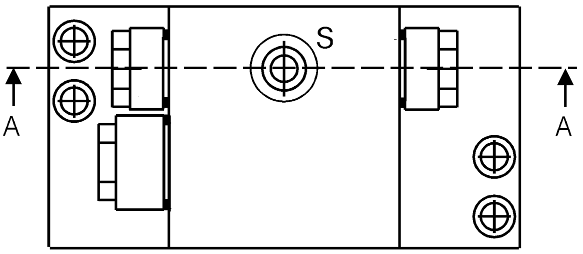

178 2569-801
625-7802.003

875 11000-006
625-7802.008

FLUSH - UNLOADING UNIT

Item	Qty	Article no	Description	Supplementary data
000	1	178 2569-801	FLUSH - UNLOADING UNIT	
001	1	278 2144-001	. HOUSING	
002	1	378 2213-001	. PISTON	
003	2	478 3697-001	. SPRING SLEEVE	
004	1	478 3700-001	. SPRING	
005	2	478 3696-001	. SPRING SEAT	
006	2	2152 2151-206	. SEAL RING	
007	1	378 2214-001	. PISTON	
008	1	2152 2151-215	. SEAL RING	TT 026 (R 1 1/4")
009	1	478 3712-001	. SPRING	
010	1	478 3699-001	. SPRING SLEEVE	
011	1	478 3698-001	. SPRING SEAT	
013	3	2152 2118-844	. O-RING	
014	4	2522 2105-100	. PLUG	
022	1	478 3730-801	. WASHER	
023	1	478 3753-001	. SPRING	
100	4	2121 2561-381	. SCREW	Not included in delivery of valve
101	4	487 2826-002	. WASHER	Not included in delivery of valve

VALVE UNIT

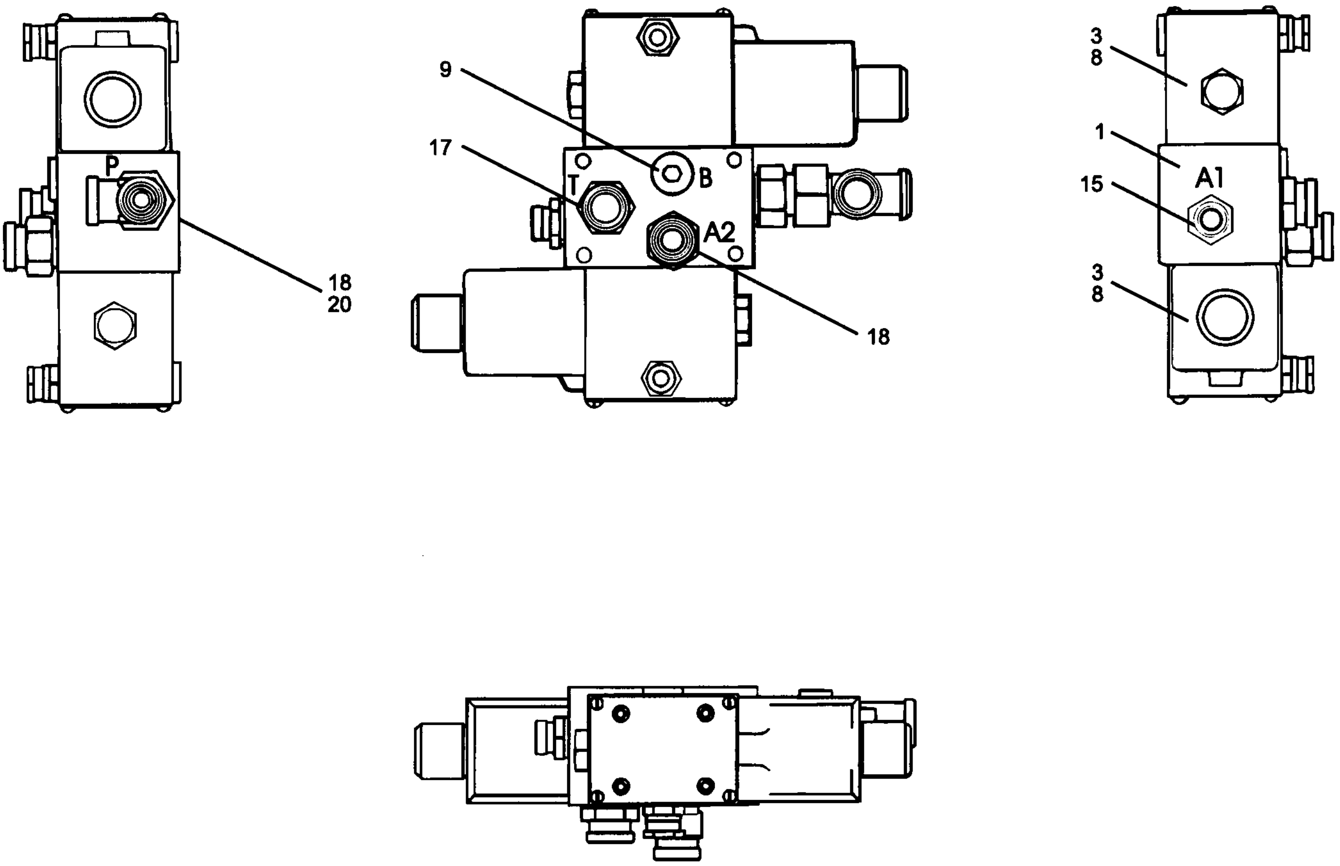

VALVE UNIT

Item	Qty	Article no	Description	Supplementary data
000	1	278 2101-801	VALVE UNIT	
001	1	178 2499-001	. VALVE BLOCK	
003	2	278 2142-801	. DIRECTIONAL VALVE	
008	8	2121 2541-378	. SCREW	MC6S 6 x 40 -12.9
009	1	2522 2105-012	. PLUG WITH SEAL	
015	1	2528 2512-615	. COUPLING	
017	1	2528 2512-622	. COUPLING	
018	1	2528 2512-623	. COUPLING	
020	1	2528 3633-222	. COUPLING	

UNLOADING UNIT

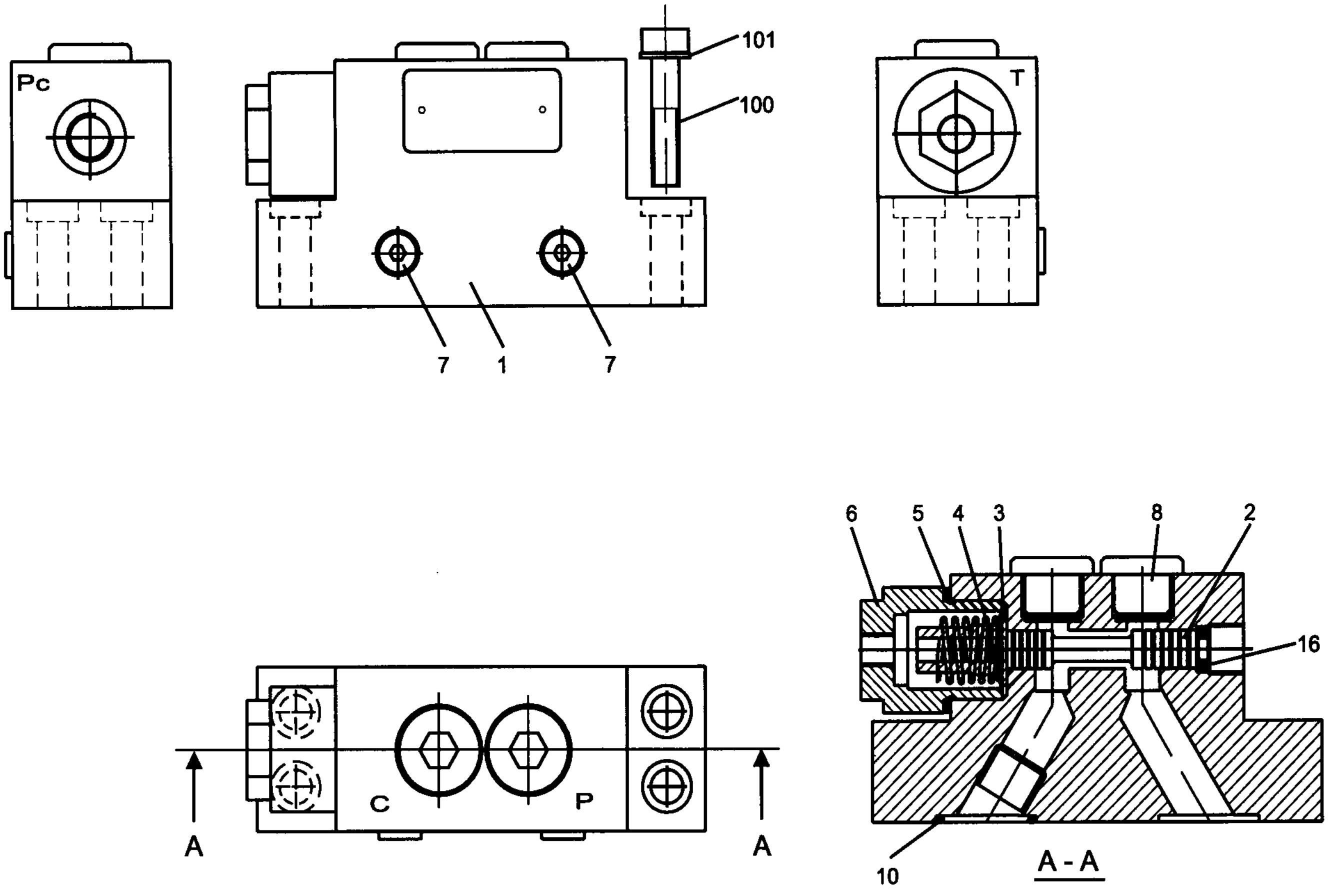

UNLOADING UNIT

Item	Qty	Article no	Description	Supplementary data
000	1	278 2088-801	UNLOADING UNIT	
001	1	278 2087-001	. HOUSING	
002	1	378 2214-001	. PISTON	
003	1	478 3698-001	. SPRING SEAT	
004	1	478 3712-001	. SPRING	
005	1	2152 2151-215	. SEAL RING	TT 026 (R 1 1/4")
006	1	478 3699-001	. SPRING SLEEVE	
007	2	2522 2105-014	. PLUG WITH SEAL	
008	2	2522 2105-034	. PLUG WITH SEAL	
010	2	2152 2115-832	. O-RING	
016	1	478 3730-001	. WASHER	
100	4	2121 2561-289	. SCREW	UC6S 1/2 UNC x 51 -12.9
101	4	487 2826-007	. WASHER	

DIRECTION VALVE

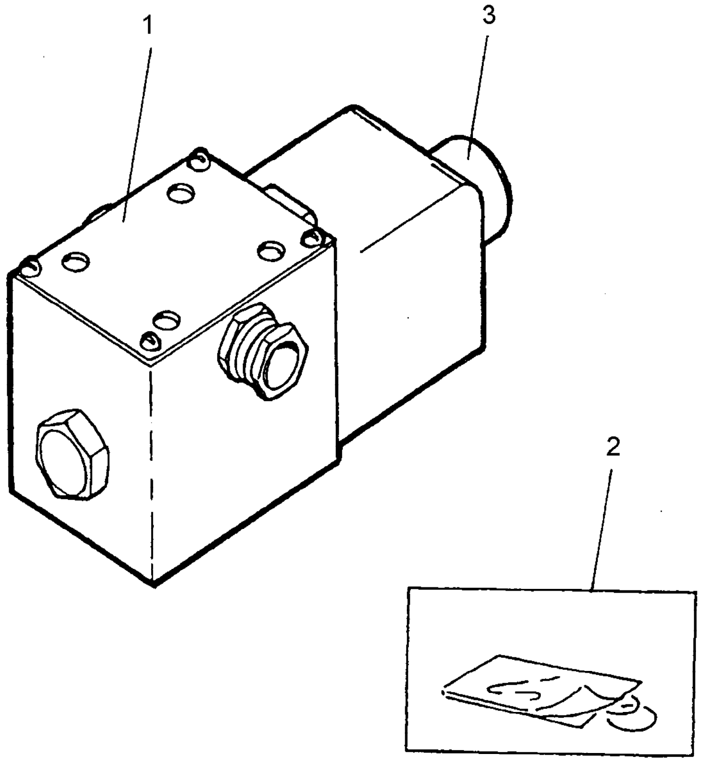

287 5950-801
625-7508

DIRECTION VALVE

Item	Qty	Article no	Description	Supplementary data
001	1	287 5950-801	DIRECTION VALVE	
002	1	489 3792-801	. GASKET SET	
003	1	489 3795-801	. COIL	

FLUSH AND UNLOADING UNIT

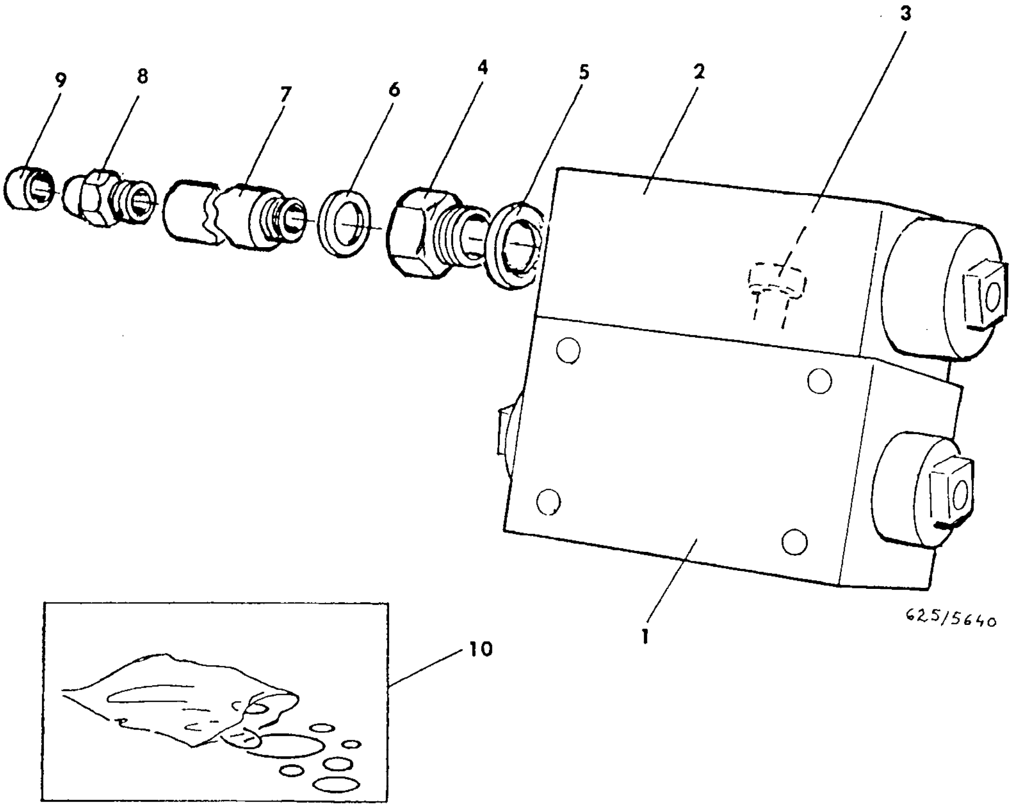

FLUSH AND UNLOADING UNIT

Item	Qty	Article no	Description	Supplementary data
000	1	388 3580-801	FLUSH AND UNLOADING UNIT	
001	1	388 3579-801	. FLUSHING VALVE	SEE FIG 625-7287
002	1	388 3576-801	. UNLOADING UNIT	SEE FIG 625-7291
003	4	2121 2541-497	. SCREW	MC63 10 x 35 -12.9
004	1	2521 2135-205	. COUPLING	
005	1	2152 2151-194	. SEAL RING	INCLUDED IN GASKET SET
006	1	2152 2151-181	. SEAL RING	INCLUDED IN GASKET SET
007	1	489 3059-801	. CHECK-RESTRICTION VALVE	
008	1	2528 2512-610	. COUPLING	
009	1	2522 4506-191	. PROTECTIVE PLUG	
010	1	489 3705-803	. GASKET SET	

FLUSHING VALVE

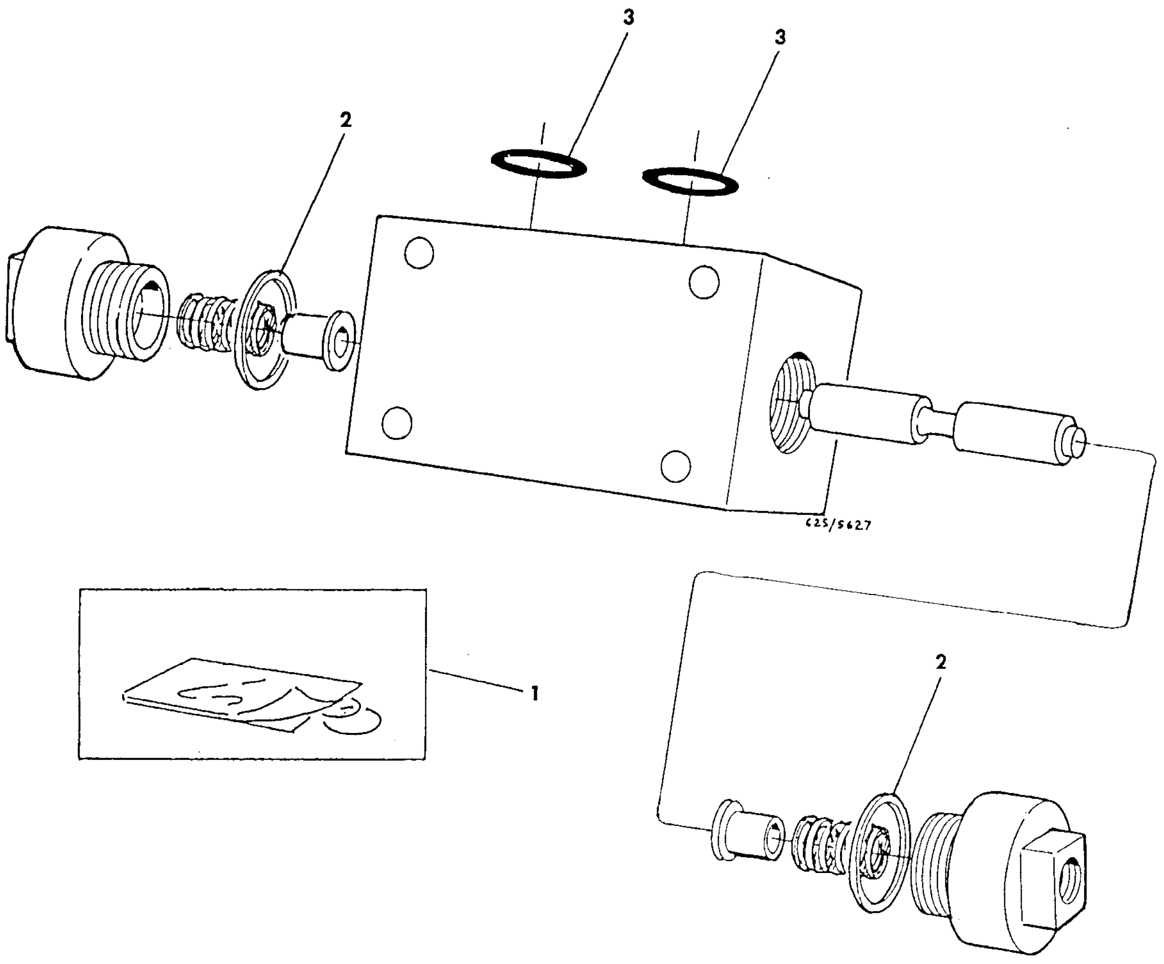

FLUSHING VALVE

Item	Qty	Article no	Description	Supplementary data
000	1	388 3579-801	FLUSHING VALVE	
001	1	489 3705-802	. GASKET SET	
002	2	2152 2151-206	.. SEAL RING	INCLUDED IN GASKET SET
003	2	2152 2118-406	.. O-RING	INCLUDED IN GASKET SET

UNLOADING UNIT

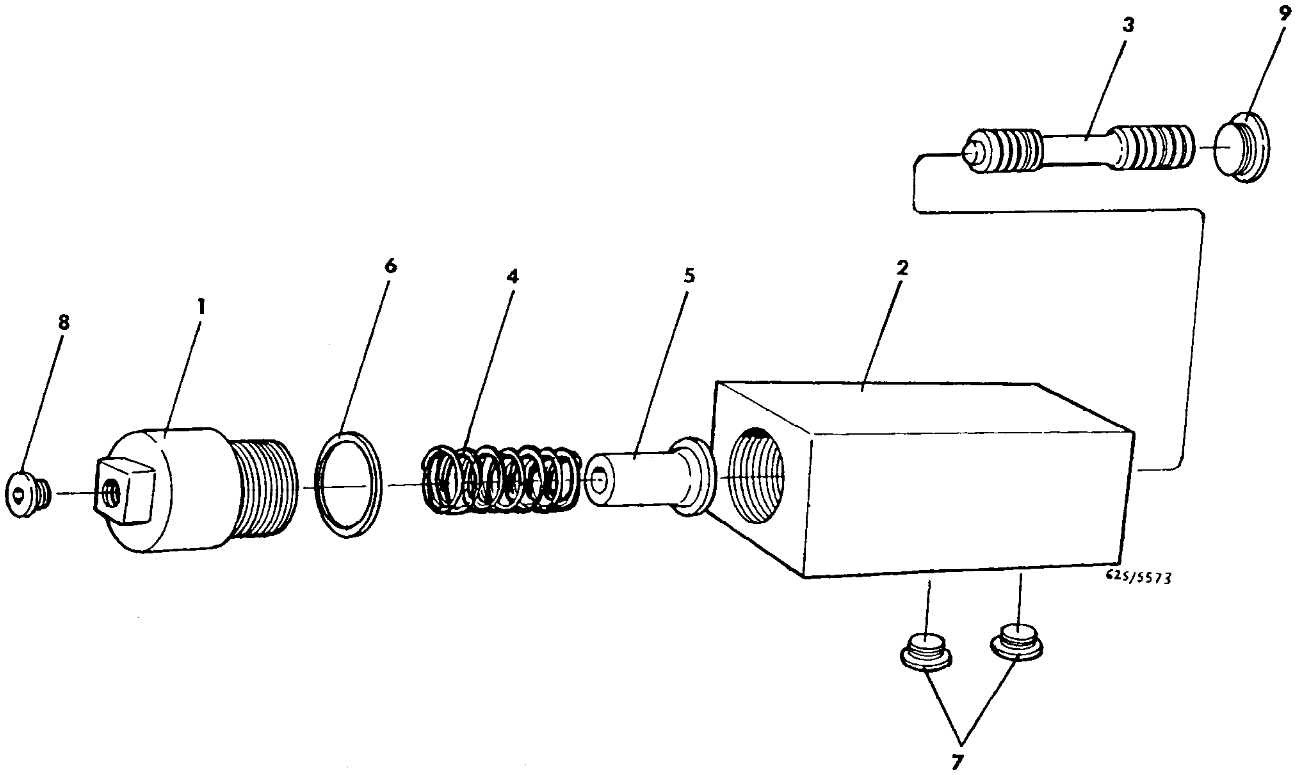

UNLOADING UNIT

Item	Qty	Article no	Description	Supplementary data
000		388 3576-801	UNLOADING UNIT	
001	1	388 3575-001	. SPRING SLEEVE	CAN NOT BE SUPPLIED SEPARATELY
002	1	287 8772-001	. VALVE HOUSING	CAN NOT BE SUPPLIED SEPARATELY
003	2	388 3574-001	. PISTON	CAN NOT BE SUPPLIED SEPARATELY
004		388 3576-005	. SPRING	CAN NOT BE SUPPLIED SEPARATELY
005	1	489 3061-001	. SPRING SEAT	CAN NOT BE SUPPLIED SEPARATLY
006	1	2152 2151-215	. SEAL RING	
007	1	2522 4502-111	. PLUG	
008	1	2522 4502-110	. PLUG	
009	1	2522 4502-113	. PLUG	

HYDRAULIC CYLINDER, C

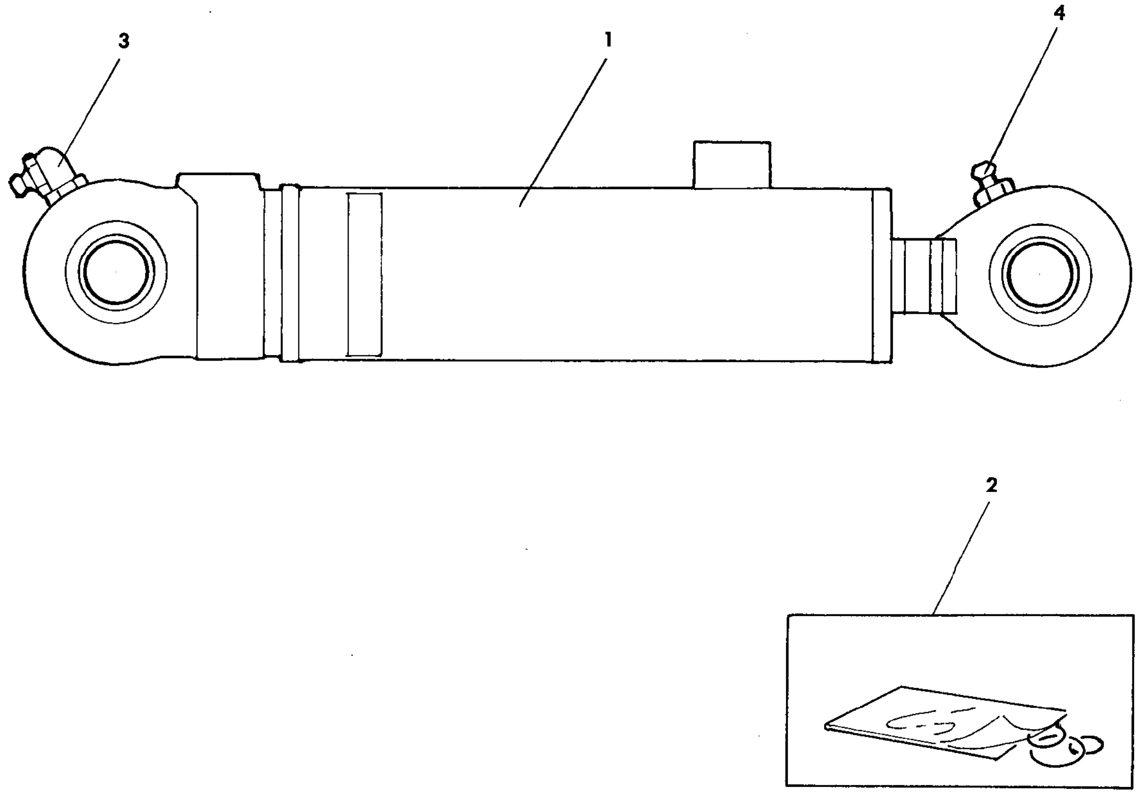

HYDRAULIC CYLINDER, C

Item	Qty	Article no	Description	Supplementary data
000	1	388 5046-801	HYDRAULIC CYLINDER, C	
001	1	388 5046-901	. HYDRAULIC CYLINDER	
002	1	489 3549-801	. GASKET SET	
003	1		GREASE NIPPLE	
004	1		GREASE NIPPLE	

FILTER UNIT, INLET

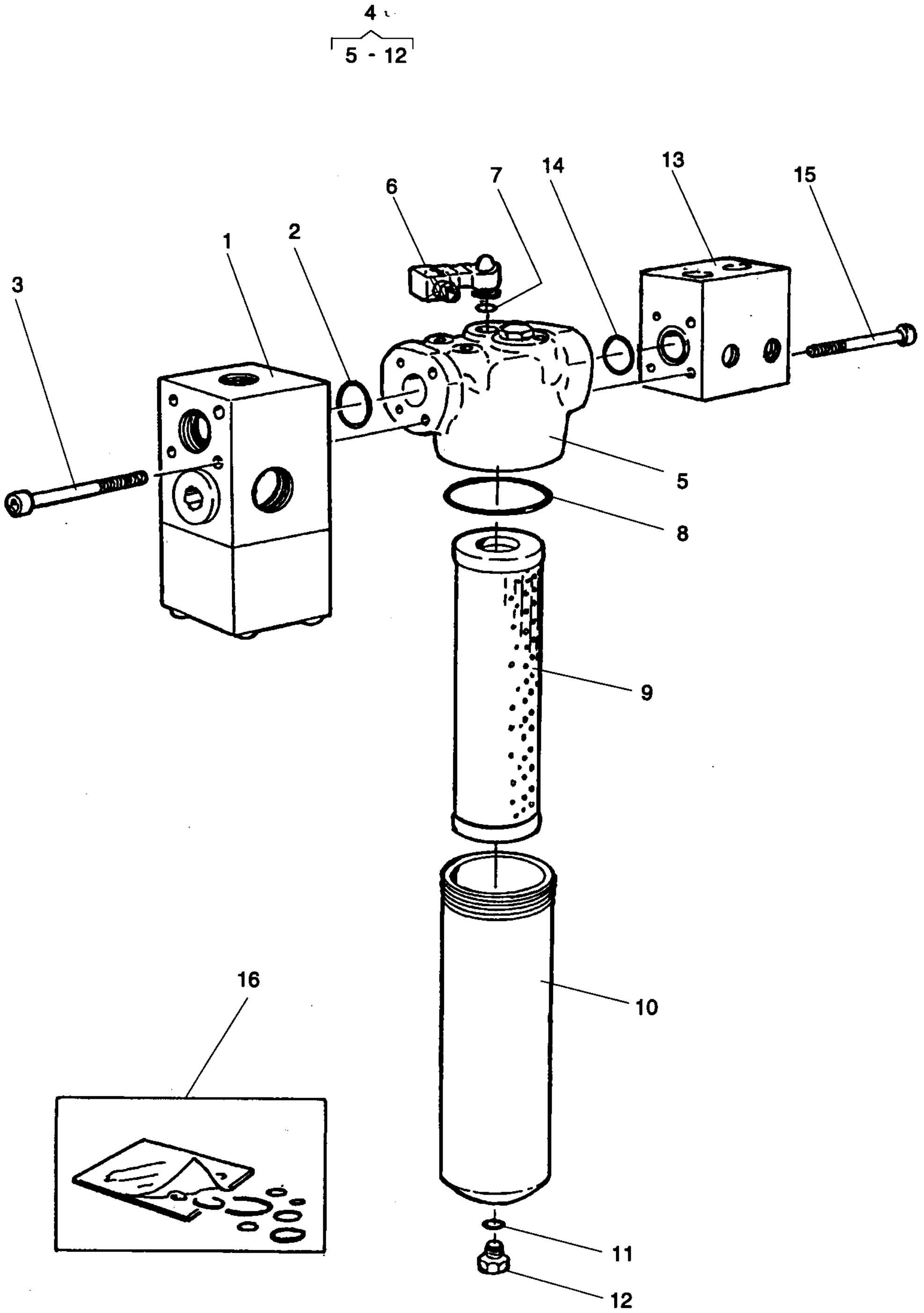

FILTER UNIT, INLET

Item	Qty	Article no	Description	Supplementary data
000	1	189 0418-801	FILTER UNIT, INLET	
001	1	489 3731-801	. VALVE UNIT	
002	1		.. O-RING	INCLUDED IN GASKET SET
003	8		.. SCREW	UC6S 1/2 x 114
004	1	489 3732-801	. FILTER UNIT	
005	1		.. FILTER HEAD	
006	1		.. EL. INDICATOR	
007	1		.. O-RING	INCLUDED IN GASKET SET
008	1		.. SEALING	INCLUDED IN GASKET SET
009	1	489 3104-001	.. FILTER ELEMENT	
010	1		.. FILTER CAP	
011	1		.. O-RING	INCLUDED IN GASKET SET
012	1		.. DRAIN PLUG	INCLUDED IN GASKET SET
013	1	390 0026-801	. BLOCK	
014	1		.. O-RING	INCLUDED IN GASKET SET
015	4		.. SCREW	UC6S 1/2 x 89
016	1	489 5647-801	. GASKET SET	

FILTER UNIT, OUTLET

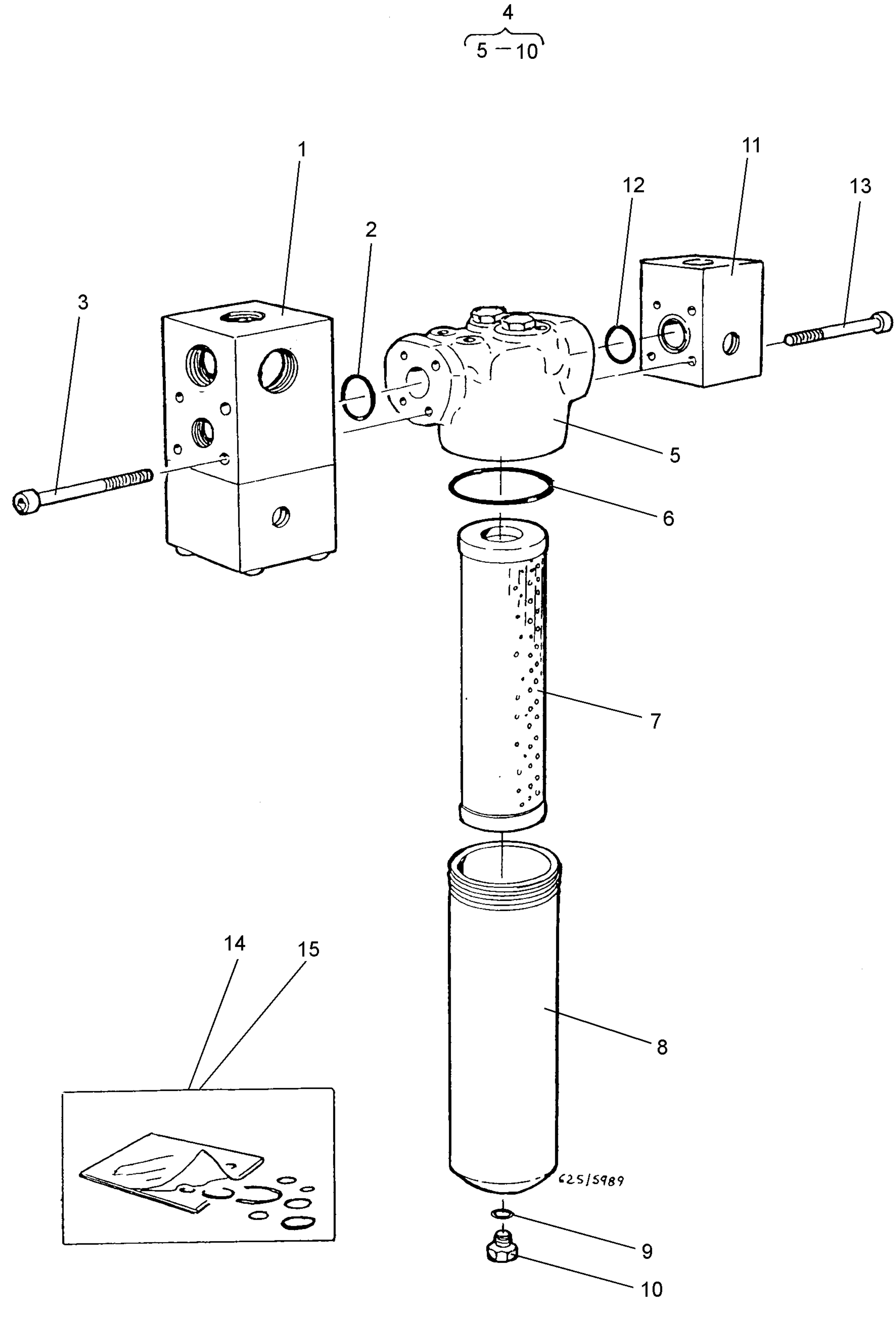

188 0118-801
625-7314.002

FILTER UNIT, OUTLET

Item	Qty	Article no	Description	Supplementary data
000	1	188 0118-801	FILTER UNIT, OUTLET	
001	1	489 3734-801	. VALVE UNIT	
002	1		.. O-RING	INCLUDED IN GASKET SET
003	8		.. SCREW	UC6S 1/2 x 114
004	1	489 3735-801	. FILTER UNIT	
005	1		.. FILTER HEAD	
006	1		.. SEALING	INCLUDED IN GASKET SET
007	1	489 3104-001	.. FILTER ELEMENT	
008	1		.. FILTER CAP	
009	1		.. O-RING	INCLUDED IN GASKET SET
010	1		.. DRAIN PLUG	INCLUDED IN GASKET SET
011	1	389 1479-801	. BLOCK	
012	1		.. O-RING	INCLUDED IN GASKET SET
013	4		.. SCREW	UC6S 1/2 x 89
014	1	489 5647-801	. GASKET SET	
015	1	489 5647-802	. GASKET SET	

ACCUMULATOR

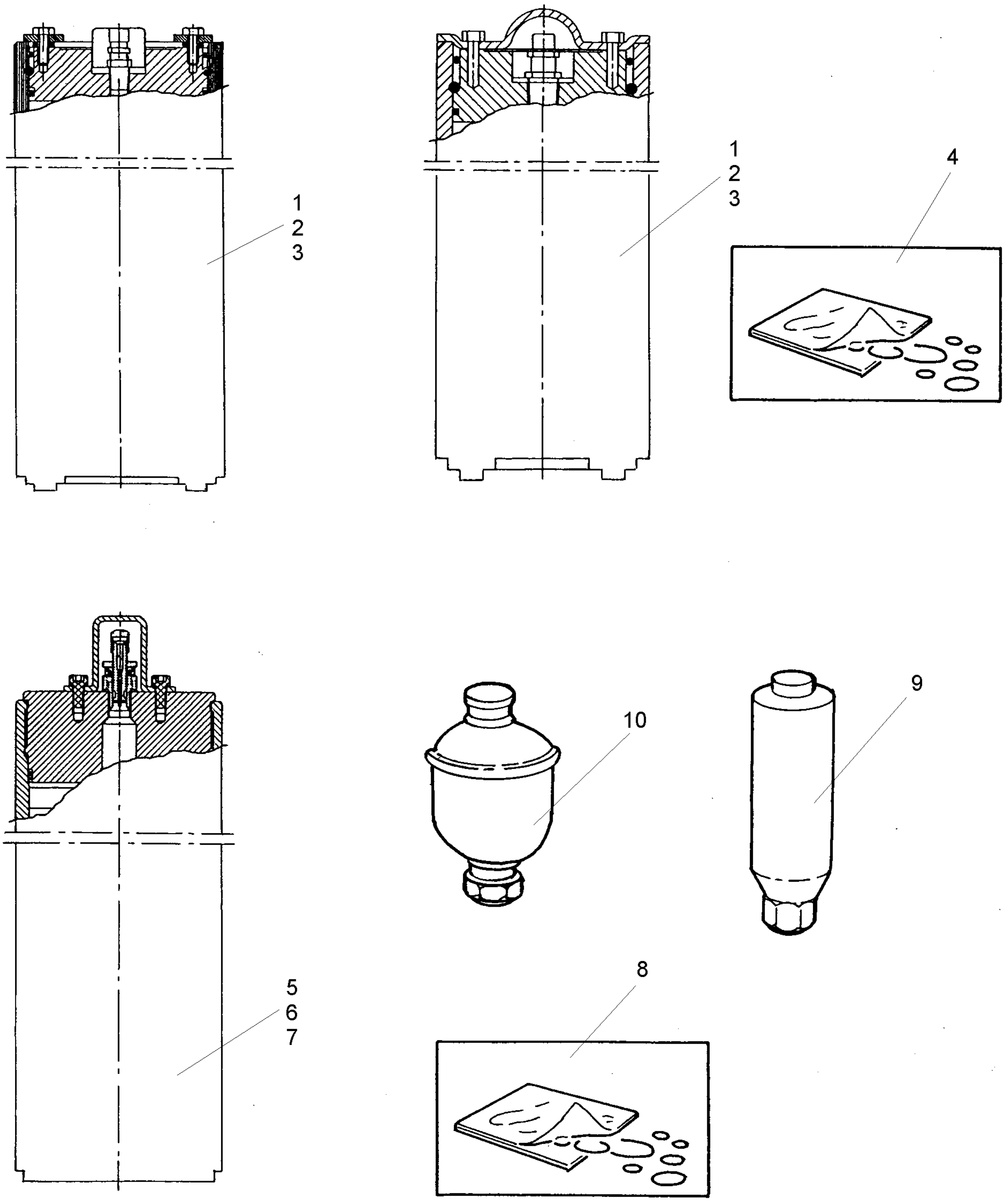

ACCUMULATOR

Item	Qty	Article no	Description	Supplementary data
001	1	388 0362-801	ACCUMULATOR	Feed press. circ; Charg. press. 1.5 MPa
002	1	388 0362-802	ACCUMULATOR	Feed press. circ; Charg. press. 2.8 MPa
003	1	388 0362-803	ACCUMULATOR	Feed press. circ; Charg. press. 2.0 MPa
004	X	488 7888	GASKET SET	Valid for item 1-3.
005	1	388 0362-801	ACCUMULATOR	Feed press. circ; Charg. press. 1.5 MPa
006	1	388 0362-802	ACCUMULATOR	Feed press. circ; Charg. press. 2.8 MPa
007	1	388 0362-803	ACCUMULATOR	Feed press. circ; Charg. press. 2.0 MPa
008	X	490 3956	GASKET SET	Valid for item 5-7.
009	1	488 4033-801	ACCUMULATOR	Stab. circuit; Charg. press. 5.0 MPa
010	1	489 2520-801	ACCUMULATOR	Stab. circuit; Charg. press. 5.0 MPa

When ordering hydraulic hoses, see marking on each hose

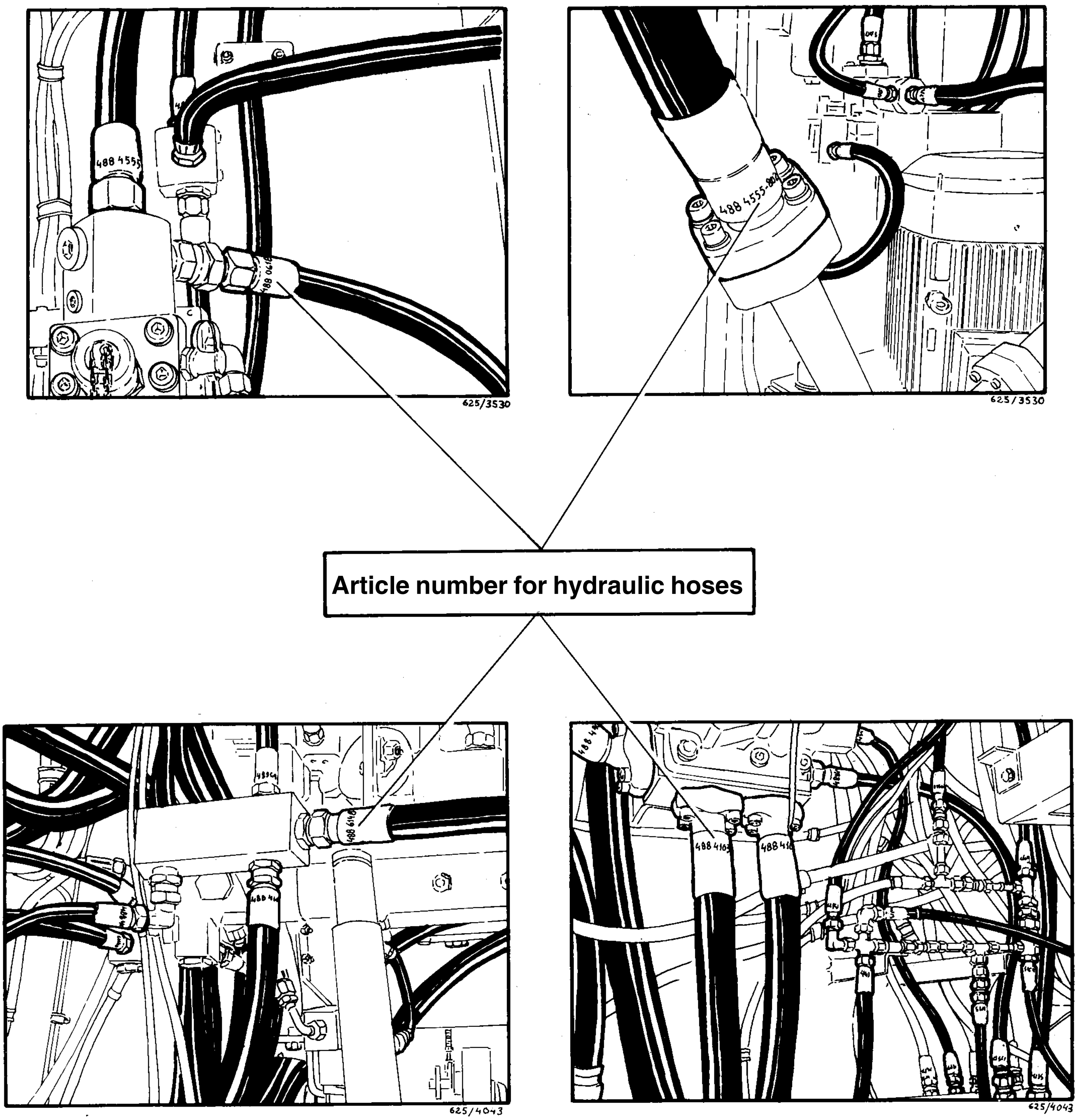

Quality demands hydraulic hoses, see page 2

MacGREGOR Cranes

Quality and Manufacturing Demands - Hydraulic Hoses

- **MacGREGOR Cranes are certified holders according to ISO 9001, and therefore the hydraulic hoses with couplings supplied by us must have type approval on all material.**
- **Standard rubber hose for our 1" high pressure hoses are made for a working pressure of 380 bar.**
- **The hoses are pressure-proof tested according to the DIN norms.**
- **100% of the high pressure hoses are proof tested and leakage tested at 450 bar during minimum 30 seconds and maximum 60 seconds. This pressure corresponds to 1.3 times the working pressure.**
- **100% of the hoses are marked with MacGREGOR Cranes part numbers, for quick and correct identification.**
- **100% of all high pressure hoses are, furthermore, marked with manufacturing date.**
- **100% of all hoses are carefully cleaned after testing. They are cleaned by means of cavitation with a mixture of water and an anti-corrosive agent.**
- **The cleanliness after washing is equal to, or better than, class 9 according to NAS norm 1638.**

Factors Influencing the Lifetime of Hydraulic HOSES

- **Pressure - temperature - bending radius - damage caused by outside factors.**
- **Sunlight and artificial light are also harmful to hydraulic hoses.**

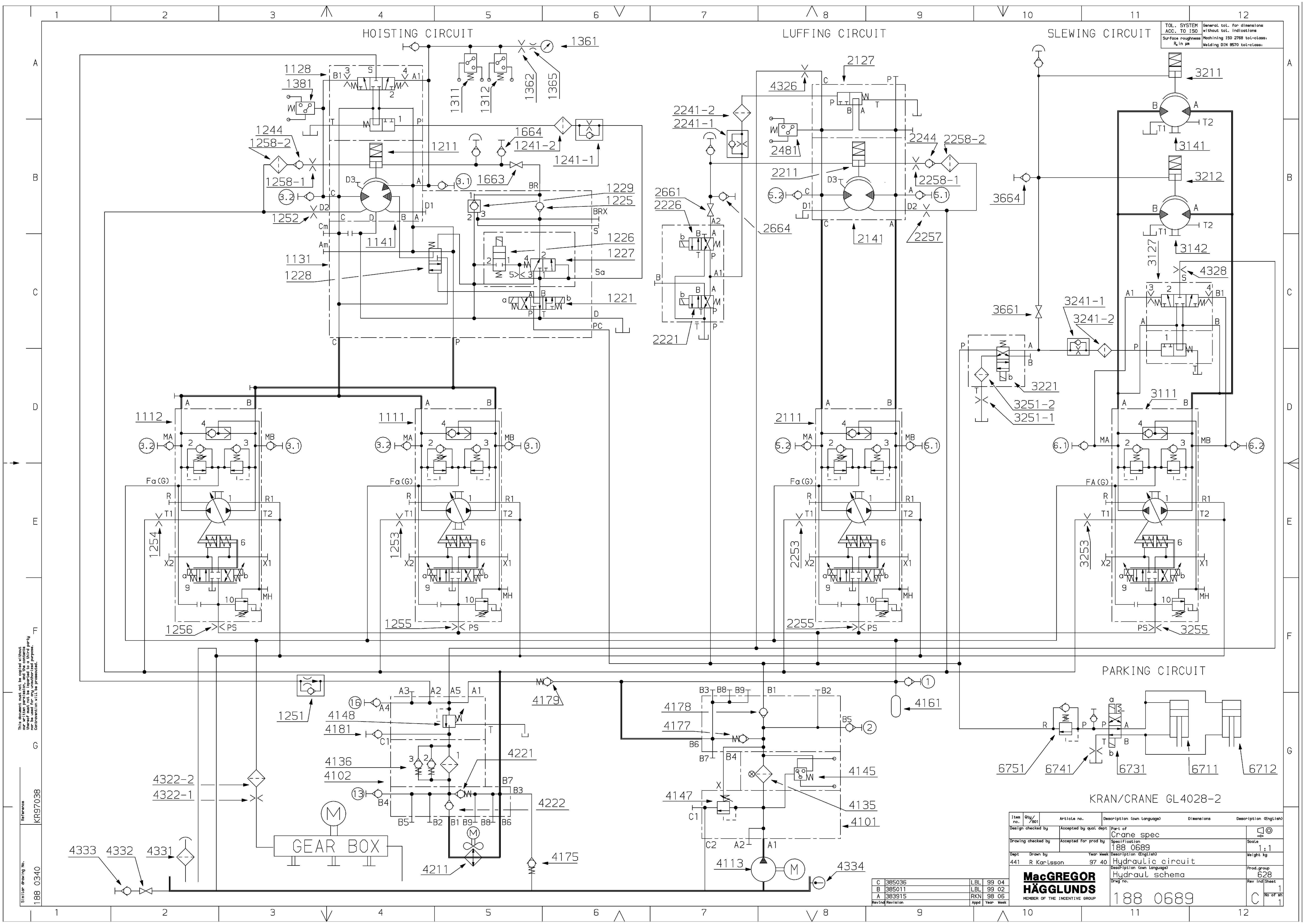
HOISTING CIRCUIT
LUFFING CIRCUIT
SLEWING CIRCUIT
PARKING CIRCUIT
GEAR BOX
KRAN/CRANE GL4028-2
TOL. SYSTEM ACC. TO ISO
General tol. for dimensions without tol. indications
Surface roughness Ra in µm
Machining ISO 2768 tol-class:
Welding DIN 8570 tol-class:
Crane spec
188 0689
Hydraulic circuit
Hydraul schema
R Karlsson
97 40
1:1
628
MacGREGOR HÄGGLUNDS
MEMBER OF THE INCENTIVE GROUP
188 0689
C 385036 LBL 99 04
B 385011 LBL 99 02
A 383915 RKN 98 06
KR97038
188 0340
This document must not be copied without our written permission, and the contents thereof must not be imparted to a third party nor be used for any unauthorized purpose. Contravention will be prosecuted.

MacGREGOR Hydraulic Component Specification Status

HYDRAULISKA KOMPONENTER<br>enligt kopplingsschemat			HYDRAULIC COMPONENTS<br>according to the circuit diagram	
Pos<br>Item	Benämning	Description	Artikel nr<br>Article No	Kompl uppgifter<br>Suppl data
	LASTKRETS	HOISTING CIRCUIT		
1111	Pump	Pump	287 9493-801	A4VG 125
1112	Pump	Pump	287 9493-801	A4VG 125
1128	Spol-avlastningsenhet	Flush-unloading unit	178 2569-801	
1131	Dubbelhast ventil	Two-speed valve	178 2568-801	
1141	Hydraulmotor	Hydraulic motor	178 2573-801	CA 420-400
1211	Broms	Brake	178 2581-702	MDA 42
1221	Riktningsventil	Direction valve	278 2134-801	
1225	Backventil	Check valve	6524 2119-300	
1226	Riktningsventil	Direction valve	478 3706-801	
1227	Riktningsventil	Direction valve	478 3746-801	
1228	Riktningsventil	Direction valve	378 2193-801	
1229	Pilotstyrd backventil	Check valve	478 3708-801	
1241-1	Back-strypventil	Check restriction valve	489 3059-801	
1241-2	Silbricka	Strainer	2590 4136-110	
1244	Backventil	Check valve	6524 2102-151	
1251	Volymströmsventil	Flow control valve	488 9540-801	
1252	Strypbricka	Restriction washer	487 6213-009	
1253	Strypbricka	Restriction washer	487 6213-014	
1254	Strypbricka	Restriction washer	487 6213-014	
1255	Strypskruv	Restriction screw	488 0044-004	
1256	Strypskruv	Restriction screw	488 0044-004	
1258-1	Strypbricka	Restriction washer	487 6213-016	
1258-2	Silbricka	Strainer	2590 4136-115	
1311	Pressostat	Pressure switch	388 3600-801	
1312	Pressostat	Pressure switch	388 3600-801	
1361	Manometer	Manometer	5693 4135-400	
1362	Strypskruv	Restriction screw	487 5073-001	
1365	Dämpningsdon	Damper	489 3764-801	
1381	Pressostat	Pressure switch	387 7053-802	0,85 MPa<br>Fallande/falling
1663	Avstängningskran	Stop cock	487 4436-801	Emergency brake
1664	Kopplingsnippel	Connection nipple	2529 2558-111	Emergency brake

This document remains our property. It must not be reproduced or made accessible to our competitors or others who could make undue use of it, without our written permission.

				Reference (Project No)<br>KR97038		
C	385036	RKN	9904	Circuit diagram	Crane type<br>GL 4028-2	
Rev ind	Revision	Appd	Year Week			
Description (English)<br>Hydraulic circuit				Drawn by<br>R Karlsson		Year Week<br>97 40
Description (own language)<br>Hydraul schema				Drawing No<br>1880689	Rev ind.<br>C	Sheet<br>1 (6)

HYDRAULISKA KOMPONENTER enligt kopplingsschemat			HYDRAULIC COMPONENTS according to the circuit diagram	
Pos Item	Benämning	Description	Artikel nr Article No	Kompl uppgifter Suppl data
	TOPPNINGSKRETS	LUFFING CIRCUIT		
2111	Pump	Pump	287 9493-801	A4VG 125
2127	Avlastningsenhet	Unloading unit	278 2088-801	
2141	Hydraulmotor	Hydraulic motor	178 1934-701	CA140
2211	Broms	Brake	178 2268-722	MDA 21
2221	Riktningsventil	Direction valve	278 2142-801	Into 278 2101-801
2226	Riktningsventil	Direction valve	278 2142-801	Into 278 2101-801
2241-1	Back-strypventil	Check-restriction valve	489 3059-801	
2241-2	Silbricka	Strainer	2590 4136-110	
2244	Backventil	Check valve	6524 2102-151	
2253	Strypbricka	Restriction washer	487 6213-014	
2255	Strypskruv	Restriction screw	488 0044-004	
2257	Strypbricka	Restriction washer	487 6213-009	
2258-1	Skrypbricka	Restriction washer	487 6213-016	
2258-2	Silbricka	Strainer	2590 4136-115	
2481	Pressostat	Pressure switch	387 7053-802	0,85 MPa Fallande/Falling
2661	Avstängningskran	Stop cock	487 4436-801	Emergency brake
2664	Kopplingsnippel	Connection nipple	2529 2558-111	Emergency brake
	VRIDNINGSKRETS	SLEWING CIRCUIT		
3111	Pump	Pump	287 9494-801	A4VG 125
3127	Spol-avlastningsventil	Flush-unloading unit	388 3580-801	
3141	Hydraulmotor	Hydraulic motor	388 3127-801	F12-60
3142	Hydraulmotor	Hydraulic motor	388 3127-801	F12-60
3211	Broms	Brake		See 288 0867-801
3212	Broms	Brake		See 288 0867-801
3221	Riktningsventil	Direction valve	287 5950-801	
3241-1	Back-strypventil	Check-restriction valve	489 3059-801	
3241-2	Silbricka	Strainer	2590 4136-110	Into 3241-1
3251-1	Strypbricka	Restriction washer	487 6213-027	
3251-2	Silbricka	Strainer	2590 4136-115	
3253	Strypbricka	Restriction washer	487 6213-014	
3255	Strypskruv	Restriction screw	488 0044-004	
3661	Avstängningskran	Stop cock	2541 4129-113	Emergency brake
3664	Kopplingsnippel	Connection nipple	2529 2558-111	Emergency brake

This document remains our property. It must not be reproduced or made accessible to our competitors or others who could make undue use of it, without our written permission.

				Reference (Project No) KR97038		
C	385036	RKN	9904	Circuit diagram	Crane type GL 4028-2	
Rev ind	Revision	Appd	Year Week			
Description (English) Hydraulic circuit				Drawn by R Karlsson		Year Week 97 40
Description (own language) Hydraul schema				Drawing No 1880689	Rev ind. C	Sheet 2 (6)

HYDRAULISKA KOMPONENTER enligt kopplingsschemat			HYDRAULIC COMPONENTS according to the circuit diagram	
Pos Item	Benämning	Description	Artikel nr Article No	Kompl uppgifter Suppl data
	MATAR-FILTRERINGSKRETS	FEEDING/FILTERING CIRCUIT		
4101	Filterenhet	Filter unit	189 0418-801	
4102	Filterenhet	Filter unit	188 0118-801	
4113	Skruvpump	Screw pump	388 1669-801	E4 038 N1
4135	Filter	Filter	489 3104-001	
4136-1	Filter	Filter	489 3104-001	
4136-2	Backventil	Check valve		Into 188 0118-801
4136-3	Backventil	Check valve		Into 188 0118-801
4145	Indikator (öppn 0,5 MPa)	Indicator (open 0,5 MPa)		Into 189 0418-801
4147	By-passventil (öppn 0,7 MPa)	By-pass valve (open 0,7 MPa)		Into 189 0418-801
4148	Matartrycksventil	Feed pressure valve		Into 188 0118-801
4161	Ackumulator	Accumulator	388 0362-801	
4175	Backventil	Check valve	6524 2104-423	
4177	Backventil	Check valve	389 1673-805	Into 189 0418-801
4178	Backventil	Check valve	389 1673-801	Into 189 0418-801
4179	Backventil	Check valve	6524 2106-357	
4181	Kopplingshus	Coupling house	2529 2458-116	
	Kopplingsnippel	Coupling nipple	2529 2558-116	
4211	Oljekylare	Oil cooler	287 6954-801	
4221	Backventil	Check valve	389 1673-802	
4222	Backventil	Check valve	389 1673-801	
4322-1	Strypskruv	Restriction screw	488 0044-001	
4322-2	Silbricka	Strainer	2590 4136-115	
4326	Strypbricka	Restriction washer	488 4856-004	
4328	Strypbricka	Restriction washer	488 8184-001	
4331	Luftfilter	Air breather	489 3166-801	
4332	Kilslidventil	Vedge valve	2541 2506-117	
4333	Koppl nippel	Connection nipple	2529 2558-116	
4334	Termometer	Thermometer	489 5906-801	

HYDRAULISKA KOMPONENTER	HYDRAULIC COMPONENTS

This document remains our property. It must not be reproduced or made accessible to our competitors or others who could make undue use of it, without our written permission.

				Reference (Project No) KR97038		
C	385036	RKN	9904	Circuit diagram	Crane type GL 4028-2	
Rev ind	Revision	Appd	Year Week			
Description (English) Hydraulic circuit				Drawn by R Karlsson		Year Week 97 40
Description (own language) Hydraul schema				Drawing No 1880689	Rev ind. C	Sheet 3 (6)

MacGREGOR **Hydraulic Component Specification** **Status**

enligt kopplingsschemat			according to the circuit diagram	
Pos<br>Item	Benämning	Description	Artikel nr<br>Article No	Kompl uppgifter<br>Suppl data
	Parkeringskrets	Parking circuit		
6711	Hydraulcylinder	Hydraulic cylinder	388 5046-801	
6712	Hydraulcylinder	Hydraulic cylinder	388 5046-801	
6731	Riktningsventil	Direction valve	388 5114-801	
6741	Strypbricka	Restriction washer	487 6213-028	
6751	Tryckbegränsningsvent.	Pressure relief valve	6523 2151-242	

This document remains our property. It must not be reproduced or made accessible to our competitors or others who could make undue use of it, without our written permission.

				Reference (Project No)		
				KR97038		
C	385036	RKN	9904	Circuit diagram	Crane type	
Rev ind	Revision	Appd	Year Week		GL 4028-2	
Description (English)				Drawn by		Year Week
Hydraulic circuit				R Karlsson		97 40
Description (own language)				Drawing No	Rev ind.	Sheet
Hydraul schema				1880689	C	4 (6)

MacGREGOR Hydraulic Component Specification Status

Mätpunkt<br>Gauge connection	Hydraulschema<br>Hydraulic circuit	Ventil<br>Valve	Tryck vid +40-50 C°<br>Pressure at +40-50 C°	Not<br>Note
1	Feed pressure inlet		2 MPa (20 bar)	(x)
2	Control pressure	4177	2,8 MPa (28 bar)	
3.1	Hoisting shock circuit pump	1111-3, 1112-3	42 MPa (420 bar)	(1)
3.1	Pressure cut off	1110-10,1112-10	40 MPa (400 bar)	(1)
3.1	Starting pressure	1311	29 MPa (290 bar)	(1)(3)
3.1	Running pressure	1312	27 Mpa (270 bar)	(1)(3)
3.2	Hoisting pressure	1111-2,1112-2	10 MPa (100 bar)	(1)
5.1	Luffing shock circuit pump	2111-3	42 MPa (420 bar)	(1)
5.1	Pressure cut off	2111-10	27,5 MPa (275 bar)	(1)
5.2	Luffing pressure	2111-2	10 MPa (100 bar)	(1)
6.1, 6.2	Slewing shock circuit	3111-2,3111-3	34 MPa (340 bar)	(1)
6.1, 6,2	Pressure cut off	3111-10	29 Mpa (290 bar)	(1)
13	Flushing pressure		>0,3 MPa (>3 bar)	
16	Feed pressure outlet	4148	≈1,3 MPa (≈13 bar)	

(x) Pressure setting at valve 4148
(1) Opening pressure
(2) Zero pressure with controllers in neutral position
(3) 40 ton (single speed) layer two

Plussning / Plussing		Tryck vid +40-50°C/Pressure at +40-50°C
Last / Hoisting	(B-port pump)	10 MPa (100 bar) $^{+2\ \text{MPa (20 bar)}}_{-0}$
Toppning / Luffing	(B-port pump)	10 MPa (100 bar) $^{+2\ \text{MPa (20 bar)}}_{-0}$

This document remains our property. It must not be reproduced or made accessible to our competitors or others who could make undue use of it, without our written permission.

				Reference (Project No) KR97038			
C	385036	RKN	9904	Circuit diagram		Crane type GL 4028-2	
Rev ind	Revision	Appd	Year Week				
Description (English) Hydraulic circuit				Drawn by R Karlsson			Year Week 97 40
Description (own language) Hydraul schema				Drawing No 1880689		Rev ind. C	Sheet 5 (6)

OPENING PRESSURE, CHECK VALVES			
Check valve	Opening pressure	Check valve	Opening pressure
1225	0,02 MPa (0,2 bar)	4136-3	0,35 MPa (3,5 bar)
1241-1	0,05 MPa (0,5 bar)	4175	0,3 MPa (3 bar)
2241-1	0,05 MPa (0,5 bar)	4177	0,5 MPa (5 bar)
1244	0,05 MPa (0,5 bar)	4178	0,02 MPa (0,2 bar)
2244	0,05 MPa (0,5 bar)	4179	0,7 MPa (7 bar)
3241-1	0,05 MPa (0,5 bar)	4221	0,7 MPa (7 bar)
4136-2	0,35 MPa (3,5 bar)	4222	0,02 MPa (0,2 bar)

Restrictions	Qty x dim	Restrictions	Qty x dim
1128-3	1 x 1,0	2257	1 x 4,0
1128-4	1 x 1,0	2258-1	1 x 1,0
1241-1	1 x 1,0	3127-3	1 x 1,0
1252	1 x 4,0	3127-4	1 x 1,0
1253	1 x 6,0	3241-1	1 x 1,0
1254	1 x 6,0	3251-1	1 x 2,0
1255	1 x 1,3	3253	1 x 6,0
1256	1 x 1,3	3255	1 x 1,3
1258-1	1 x 1,0	4322	1 x 1,0
1362	5 x 1,0	4326	1 x 3,8
2241-1	1 x 1,0	4328	1 x 3,8
2253	1 x 6,0	6741	1 x 2,0
2255	1 x 1,3		

This document remains our property. It must not be reproduced or made accessible to our competitors or others who could make undue use of it, without our written permission.

CONTROLLER PANEL

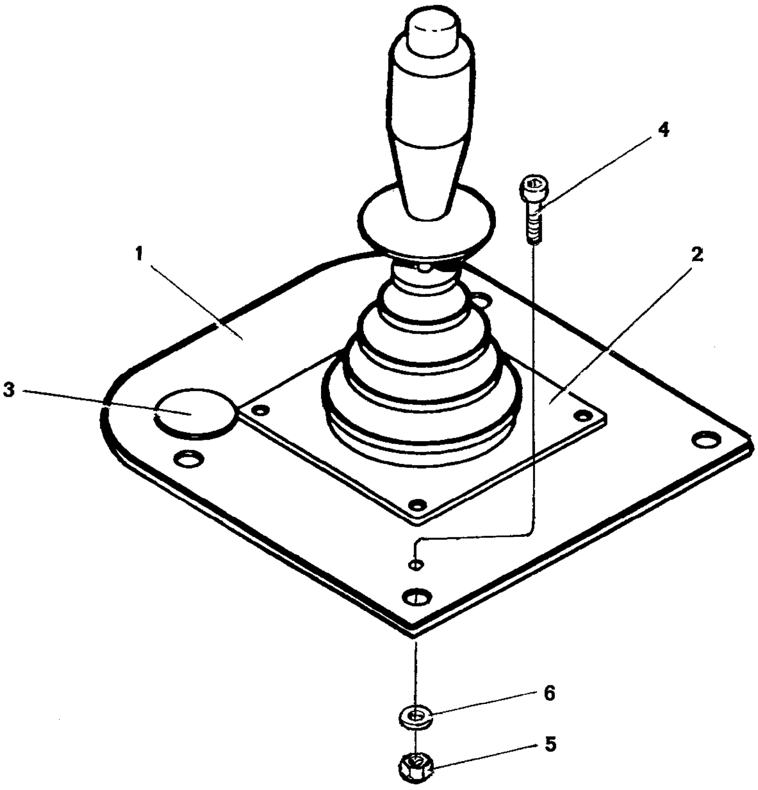

CONTROLLER PANEL

Item	Qty	Article no	Description	Supplementary data
000	1	414 6792-801	CONTROLLER PANEL	
001	1	414 7054-001	. COVER	
002	1	314 2006-802	. CONTROLLER	
003	1	2152 2067-018	. COVER PLATE	
004	1	2121 2550-331	. SCREW	MC6S 5 x 20 A4-80
005	2	2126 2634-114	. NUT	M6M 5 -A4 -80
006	1	2151 2027-146	. WASHER	RB 5.3 x 10 -A4

CONTROLLER, HOISTING

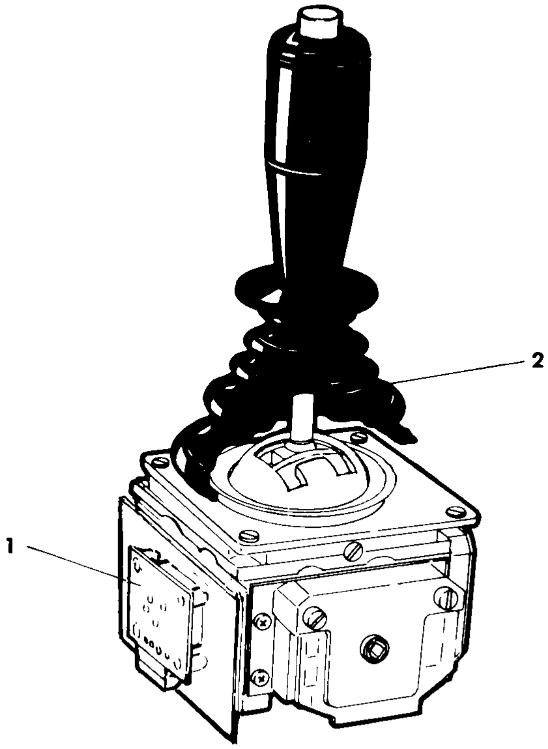

CONTROLLER, HOISTING

Item	Qty	Article no	Description	Supplementary data
000	1	314 2005-802	CONTROLLER, HOISTING	
001	1	414 5396-801	. POTENTIOMETER WITH CARD	
002	1	414 2081-801	BELLOWS WITH BRACE	SPARE PART NO: 662 0034-000

CONTROLLER, LUFFING/SLEWING

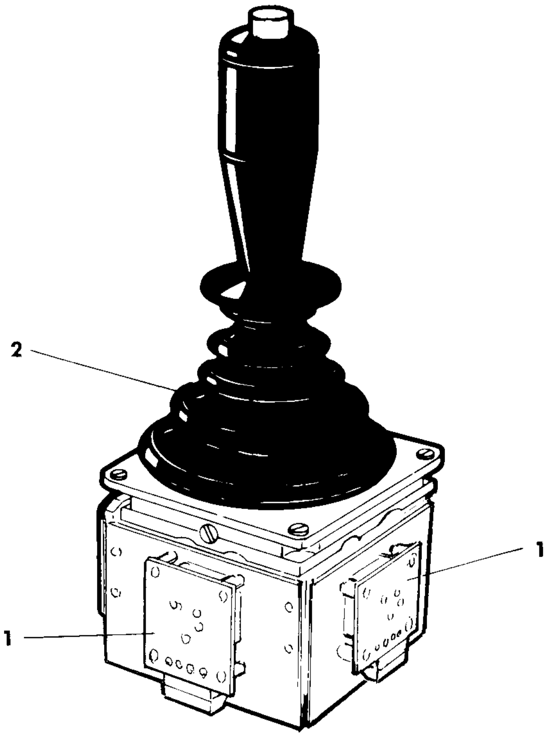

CONTROLLER, LUFFING/SLEWING

Item	Qty	Article no	Description	Supplementary data
000	1	314 2006-802	CONTROLLER, LUFFING/SLEWING	
001	2	414 5396-801	. POTENTIOMETER WITH CARD	
002	1	414 2081-801	BELLOWS WITH BRACE	SPARE PART NO: 662 0034-000

POWER SUPPLY CE, C

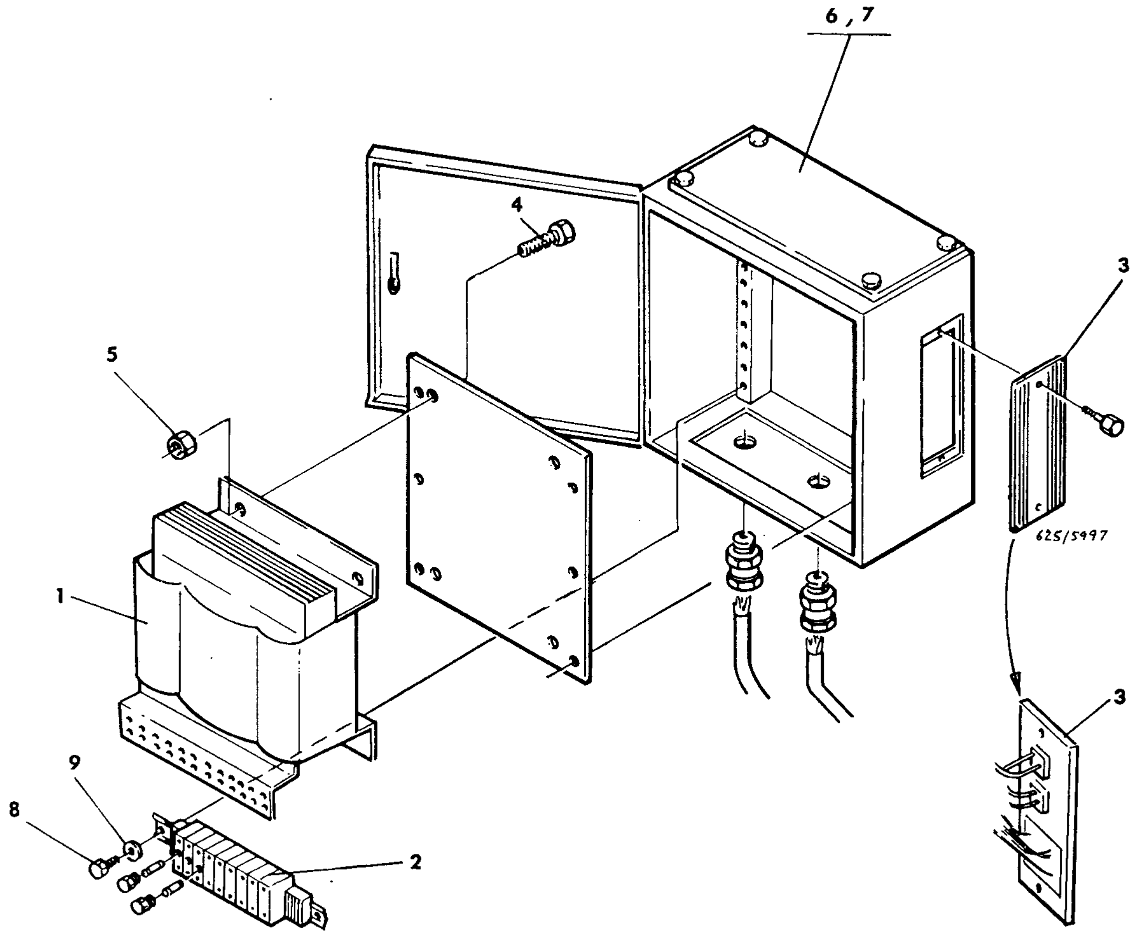

POWER SUPPLY CE, C

Item	Qty	Article no	Description	Supplementary data
000	1	314 3437-801	POWER SUPPLY CE, C	
001	1	314 3439-801	. TRANSFORMER	
002	1	314 3438-801	. FUSE PLINTH	
003	1	314 3440-801	. RECTIFIER PLATE	
004	14	2121 2032-451	. SCREW	M6S 8 x 20 -8.8 FZB; DIN 933
005	14	2126 2032-118	. NUT	M6M 8 -8 FZB
006	3	5239 2105-210	. GASKET	
007	1	5237 4116-622	. FLANGE	
008	2	2121 2032-368	. SCREW	M6S 6 x 16 -8.8 FZB
009	2	2126 2032-116	. NUT	M6M 6 -8 FZB

SLIPRING UNIT

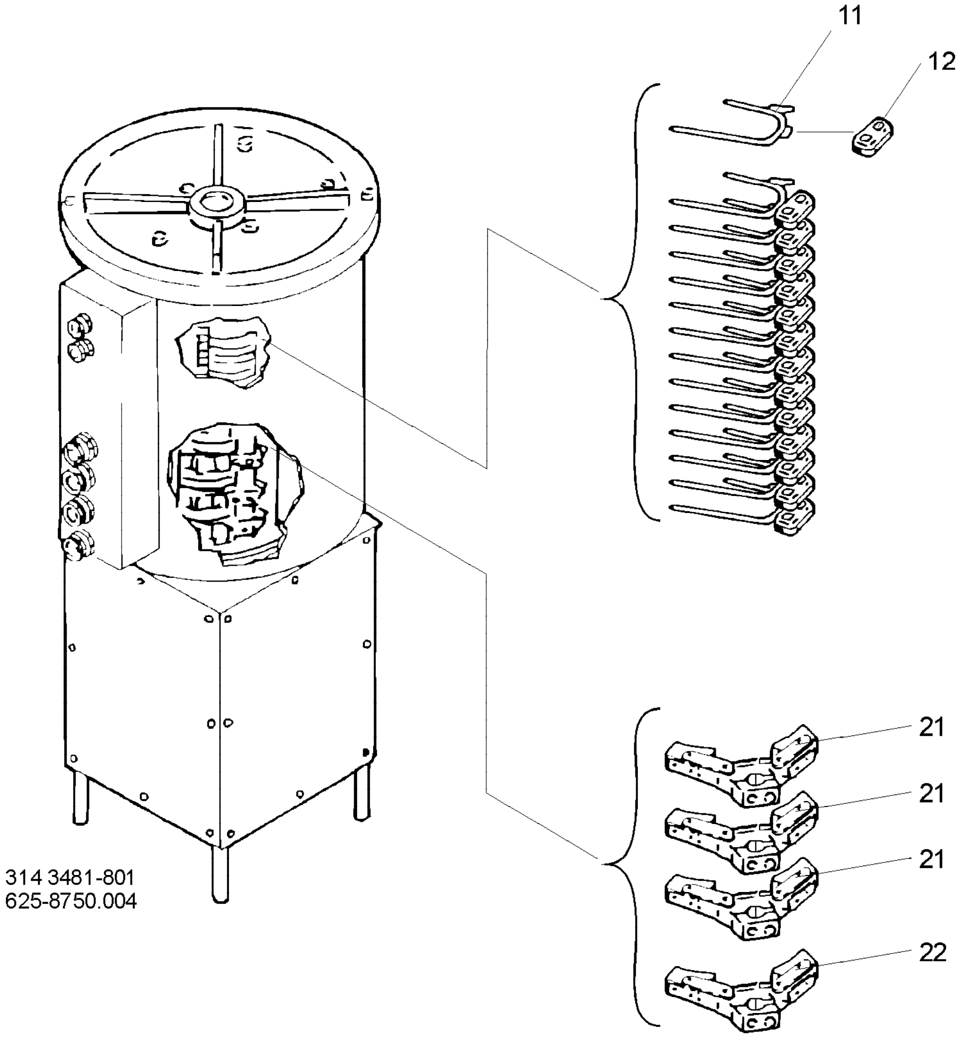

SLIPRING UNIT

Item	Qty	Article no	Description	Supplementary data
000	1	314 3481-801	SLIPRING UNIT	
011	10	875 17202-001	. SIGNAL BRUSH, COMPLETE	20 A
012	12	875 17203-001	. HOLDER	
021	3	875 17201-001	. POWER BRUSH, COMPLETE	400 A
022	1	875 17201-002	. POWER BRUSH, COMPLETE	400 A, earth

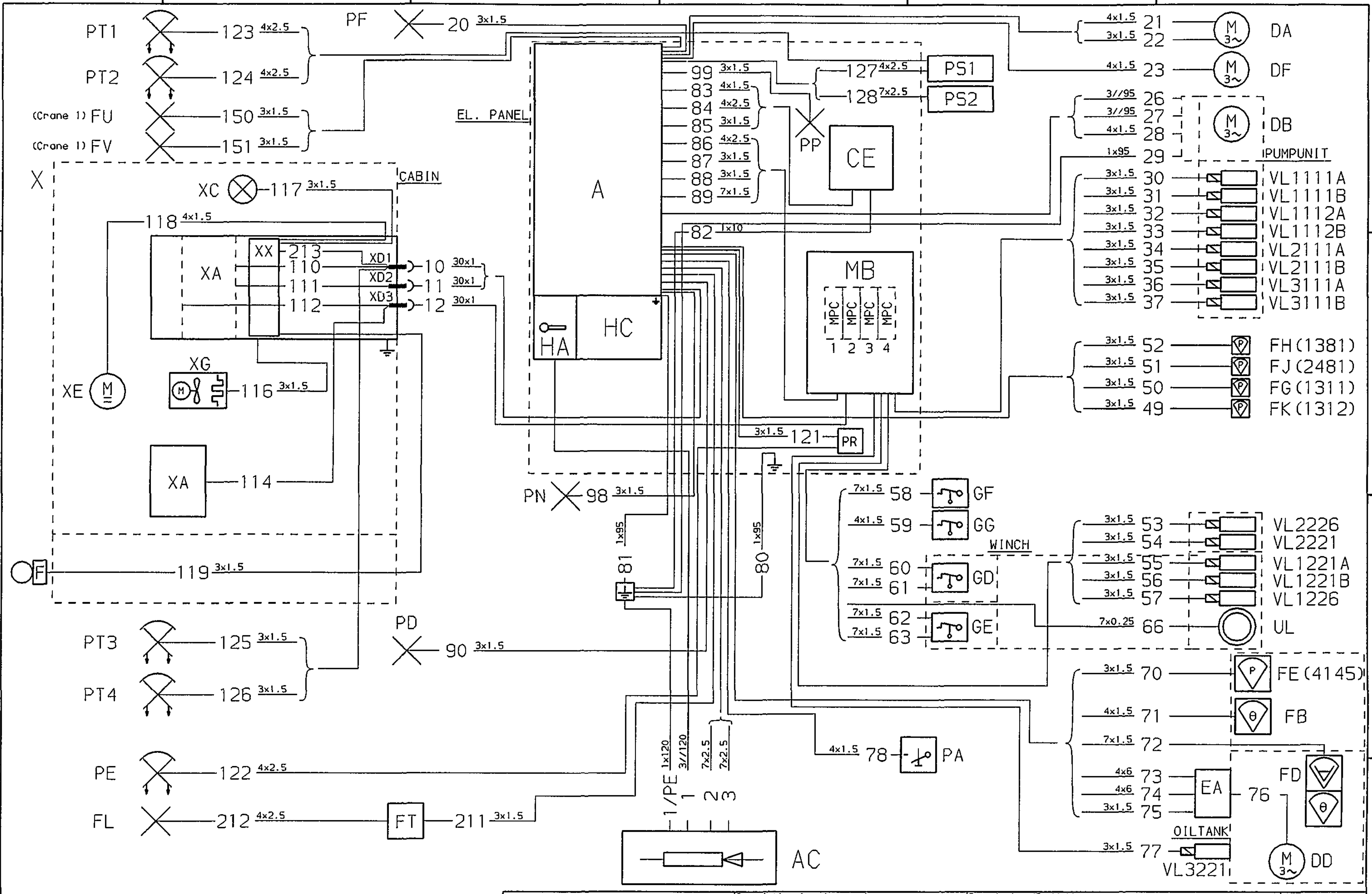

This document must not be copied without our written permission, and the contents thereof must not be imparted to a third party nor be used for any unauthorized purpose. Contravention will be prosecuted.

Reference	KR97038
Similar drawing no.	314 3836

MacGREGOR HÄGGLUNDS
MEMBER OF THE INCENTIVE GROUP

Description (own language): Block schema	Description (English): Block diagram	Prod.group: 628	
Design checked by: ASG	Drawing checked by: ASG	Drwg no.	Rev ind / Sheet: 1
Dept: 421	Drawn by: A Sundberg	Year Week: 97 42	No of sh: 1
		314 3904	

Rev ind	Revision	Appd	Year	Week

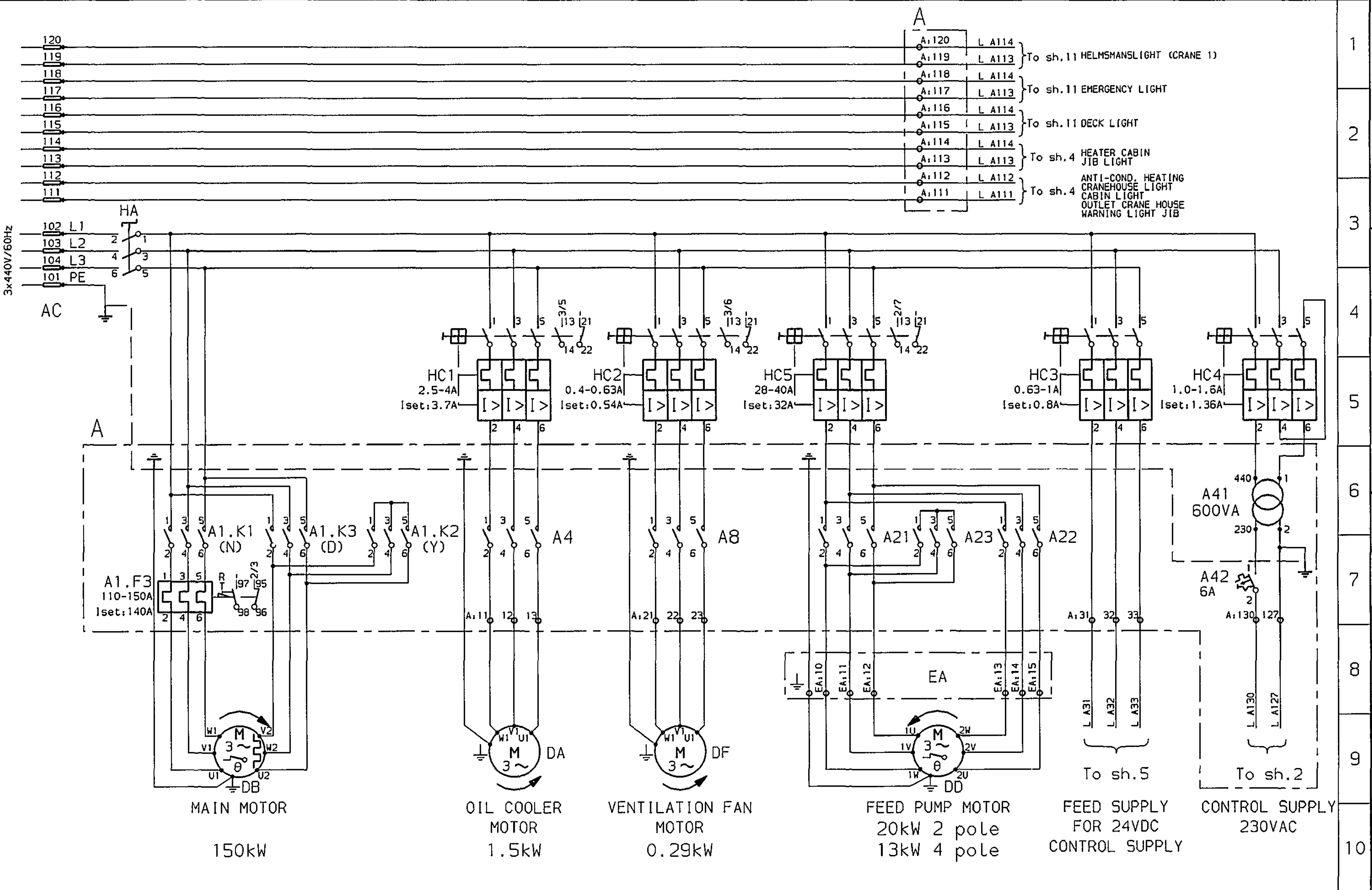

Comp.list : 414 6983

Rev Ind	Revision	Appd	Year	Week
A	383683	LBL	97	43

MacGREGOR HÄGGLUNDS
MEMBER OF THE INCENTIVE GROUP

Description (own language)	Krets schema
Description (English)	Circuit diagram
Design checked by	ASG
Drawing checked by	ASG
Dept	421
Drawn by	A Sundberg
Year Week	97 35
Drwg no.	314 3905
Prod.group	628
Rev ind	A
Sheet	1
No of sh	11

Reference KR97038

Similar drawing no. 314 3837

This document must not be copied without our written permission, and the contents thereof must not be imparted to a third party nor be used for any unauthorized purpose. Contravention will be prosecuted.

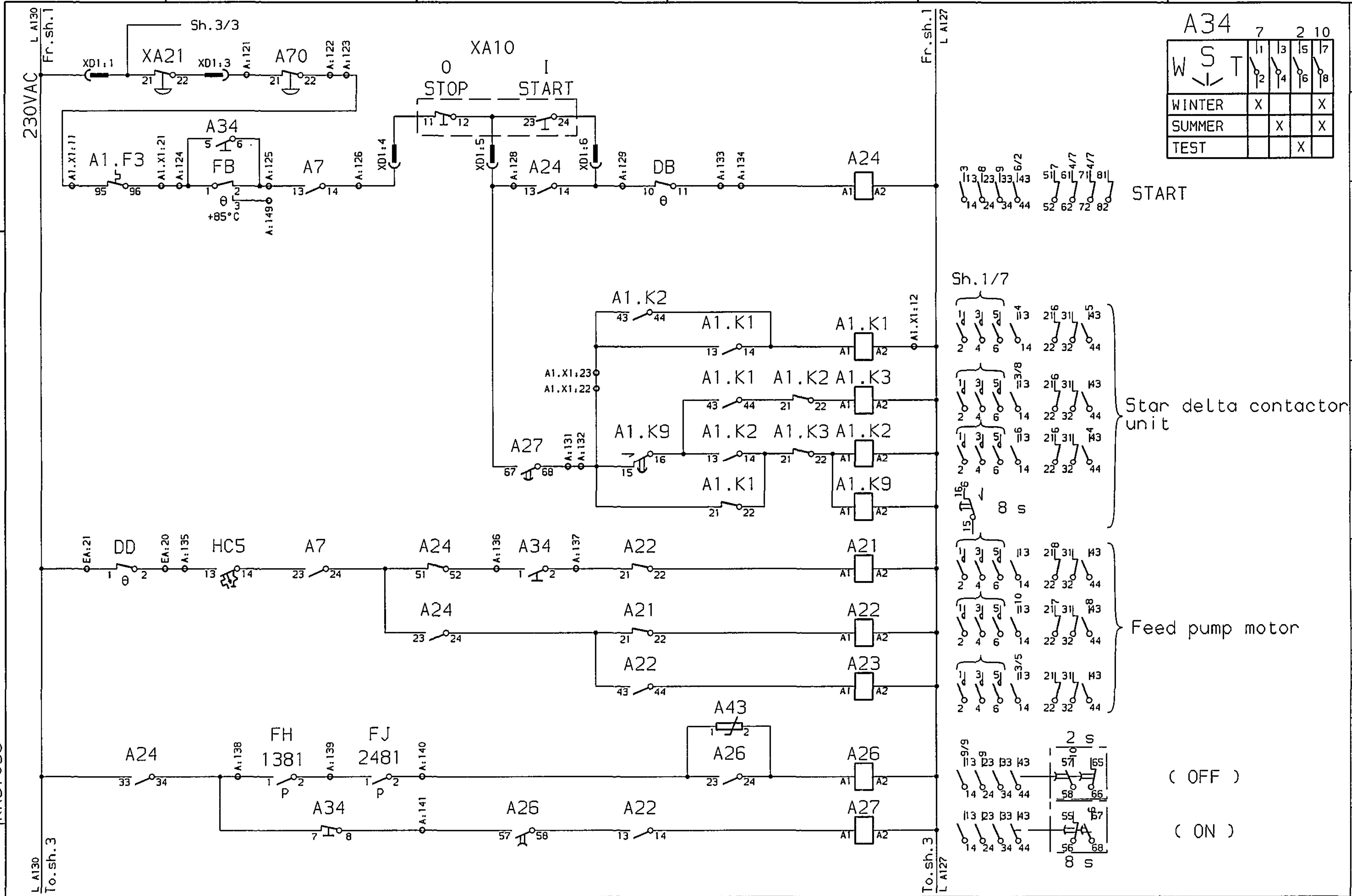

A34	7	3	2	10
W S T	1-2	3-4	5-6	7-8
WINTER	X			X
SUMMER		X		X
TEST			X	

This document must not be copied without our written permission, and the contents thereof must not be imparted to a third party nor be used for any unauthorized purpose. Contravention will be prosecuted.

Reference: KR97038

Similar drawing no. 314 3837

Rev ind	Revision	Appd	Year	Week
A	383683	LBL	97	43

MacGREGOR HÄGGLUNDS
MEMBER OF THE INCENTIVE GROUP

Description (own language)	Krets schema
Description (English)	Circuit diagram
Prod.group	628
Design checked by	ASG
Drawing checked by	ASG
Drwg no.	314 3905
Rev ind	A
Sheet	2
No of sh	11
Dept	421
Drawn by	A Sundberg
Year Week	97 36

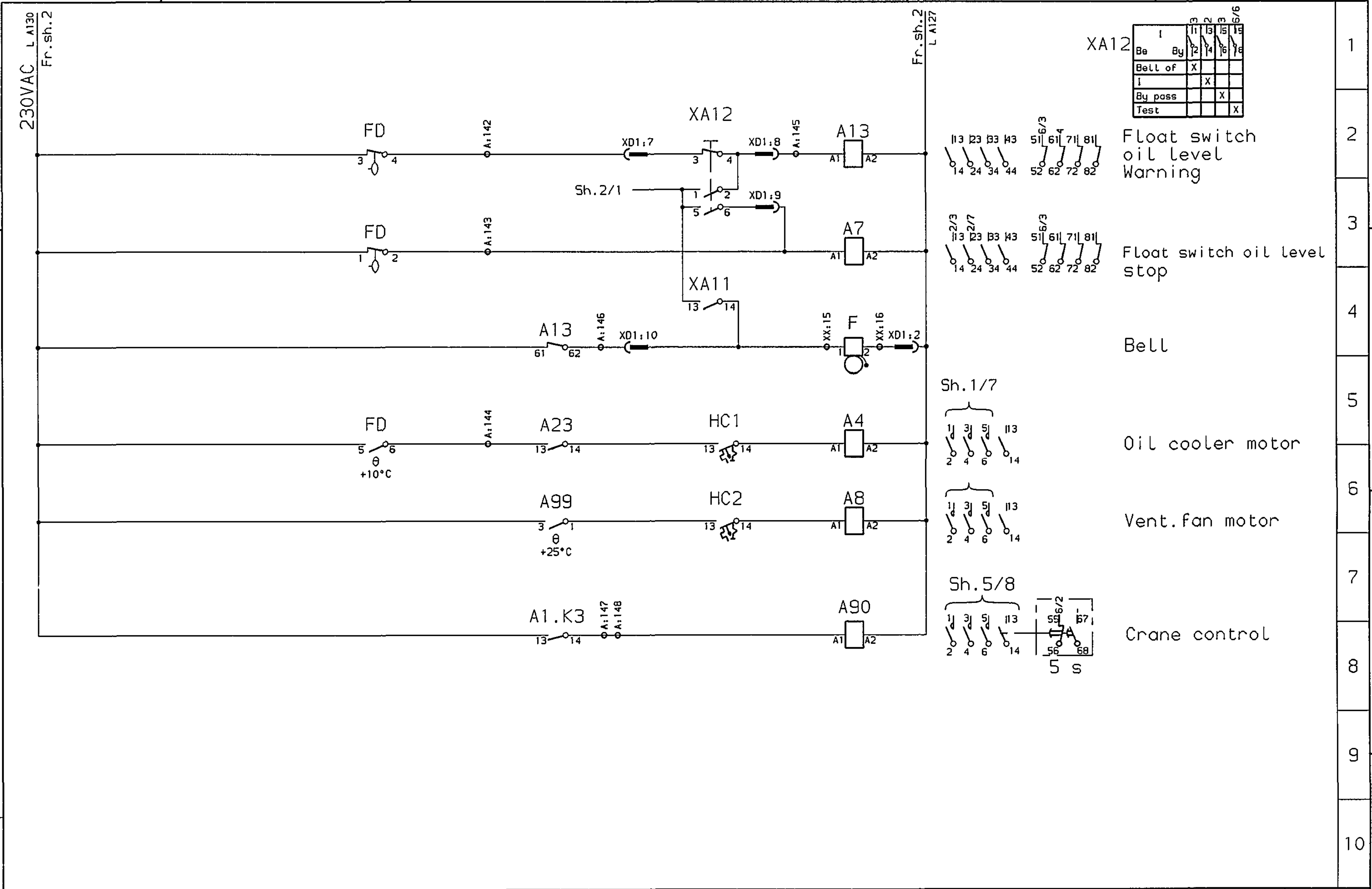

This document must not be copied without our written permission, and the contents thereof must not be imparted to a third party nor be used for any unauthorized purpose. Contravention will be prosecuted.

Reference: KR97038

Similar drawing no.: 314 3837

Rev ind	Revision	Appd	Year	Week
A	383683	LBL	97	43

MacGREGOR HÄGGLUNDS
MEMBER OF THE INCENTIVE GROUP

Description (own language)	Description (English)	Prod.group
Krets schema	Circuit diagram	628

Design checked by	Drawing checked by	Drwg no.	Rev ind	Sheet
ASG	ASG	314 3905	A	3

Dept	Drawn by	Year Week	No of sh
421	A Sundberg	97 36	11

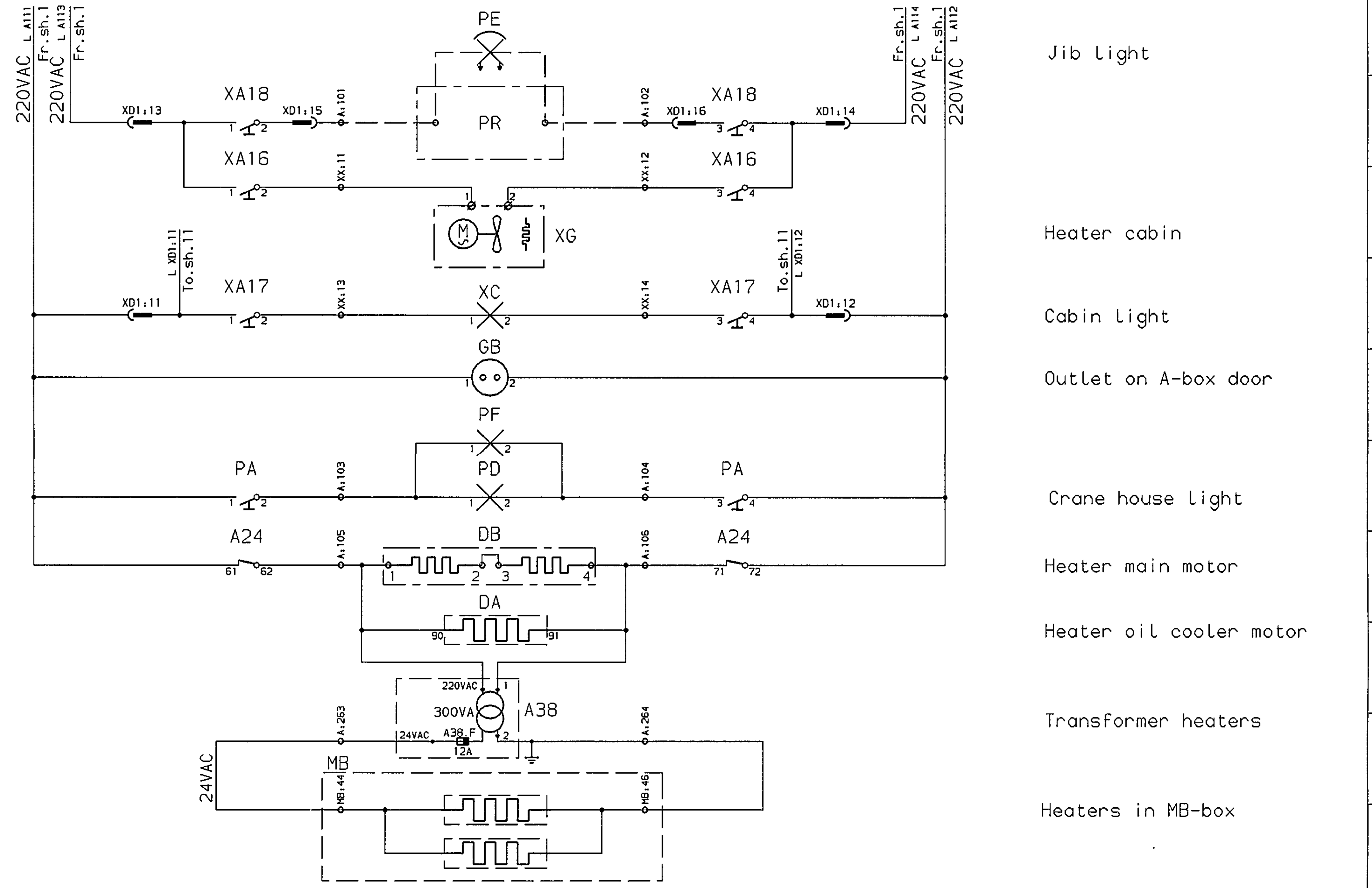

This document must not be copied without our written permission, and the contents thereof must not be imparted to a third party nor be used for any unauthorized purpose. Contravention will be prosecuted.

Reference: KR97038

Similar drawing no.: 314 3837

RevInd	Revision	Appd	Year	Week
A	383683	LBL	97	43

MacGREGOR HÄGGLUNDS
MEMBER OF THE INCENTIVE GROUP

Description (own language)	Krets schema
Description (English)	Circuit diagram
Prod.group	628
Design checked by	ASG
Drawing checked by	ASG
Dept	421
Drawn by	A Sundberg
Year Week	97 36
Drwg no.	314 3905
Rev ind	A
Sheet	4
No of sh	11

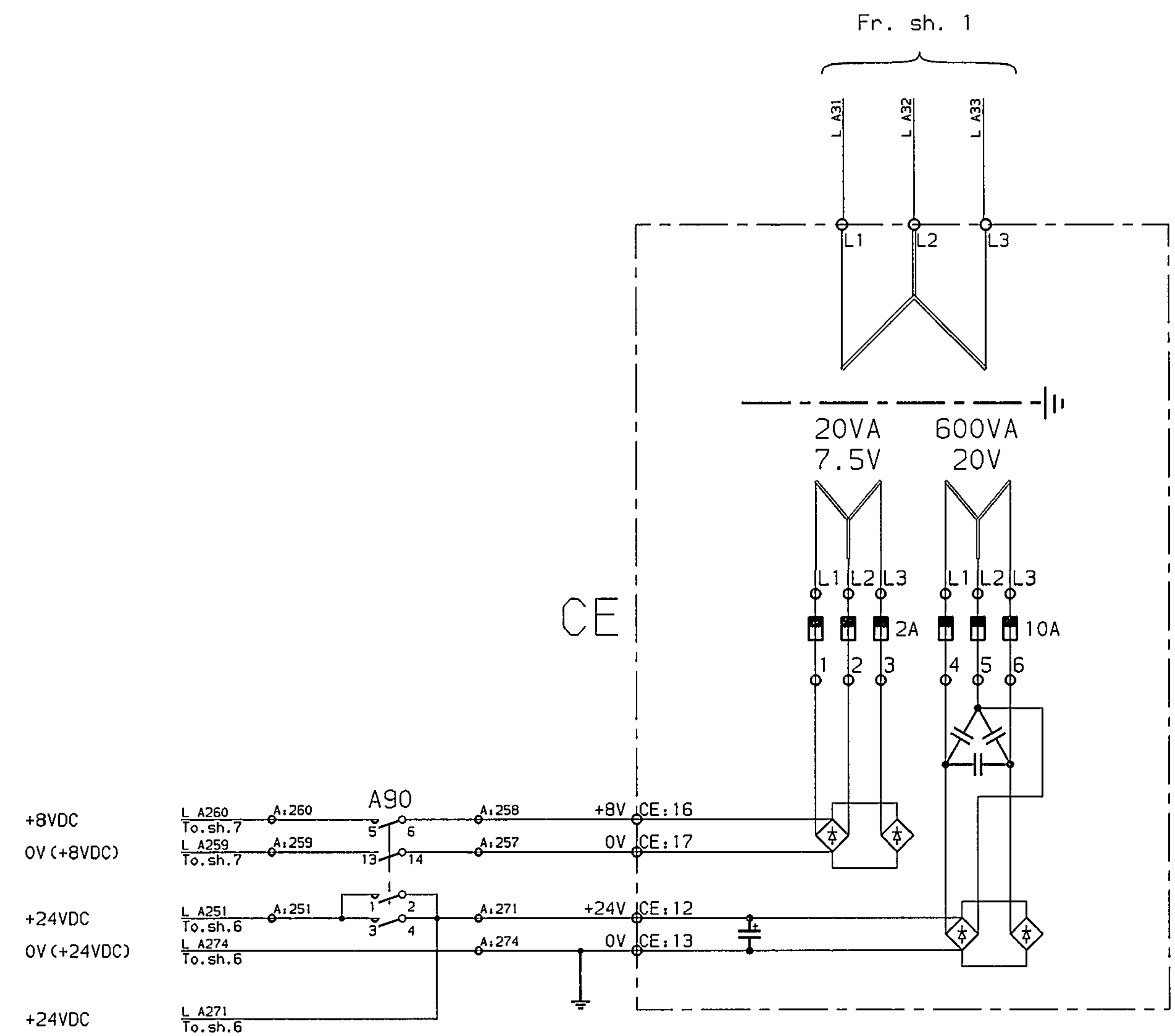

Reference: KR97038

Similar drawing no.: 314 3837

Revind	Revision	Appd	Year Week
A	383683	LBL	97 43

MacGREGOR HÄGGLUNDS
MEMBER OF THE INCENTIVE GROUP

Description (own language): Krets schema

Description (English): Circuit diagram

Prod.group: 628

Design checked by: ASG | Drawing checked by: ASG

Dept: 421 | Drawn by: A Sundberg | Year Week: 97 36

Drwg no.: 314 3905

Rev ind: A | Sheet: 5 | No of sh: 11

This document must not be copied without our written permission, and the contents thereof must not be imparted to a third party nor be used for any unauthorized purpose. Contravention will be prosecuted.

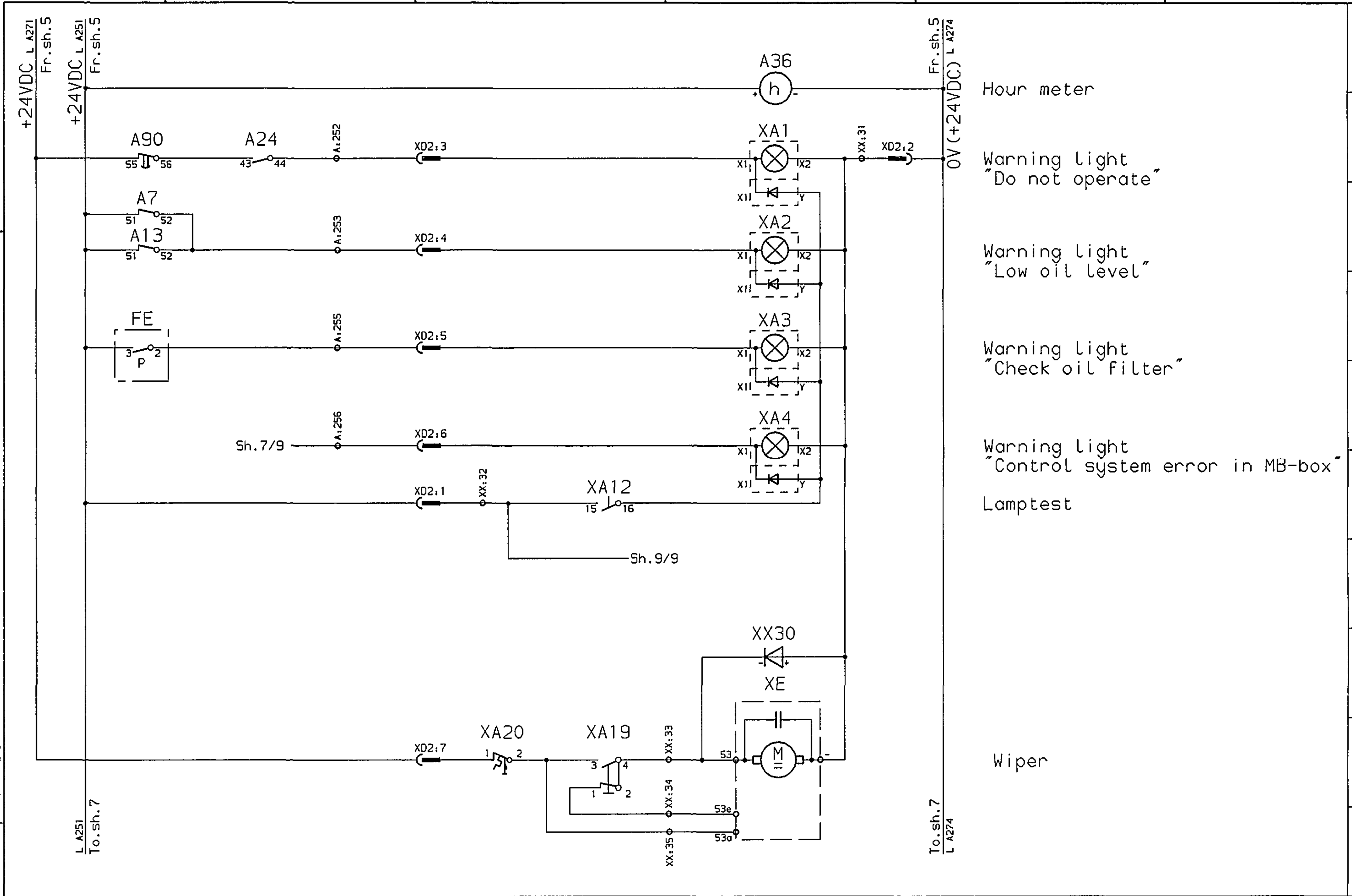

This document must not be copied without our written permission, and the contents thereof must not be imparted to a third party nor be used for any unauthorized purpose. Contravention will be prosecuted.

Reference: KR97038

Similar drawing no.: 314 3837

Rev ind	Revision	Appd	Year Week
A	383683	LBL	97 43

MacGREGOR HÄGGLUNDS
MEMBER OF THE INCENTIVE GROUP

Description (own language)	Description (English)	Prod.group
Krets schema	Circuit diagram	628

Design checked by	Drawing checked by	Drwg no.	Rev ind	Sheet
ASG	ASG	314 3905	A	6

Dept	Drawn by	Year Week	No of sh
421	A Sundberg	97 36	11

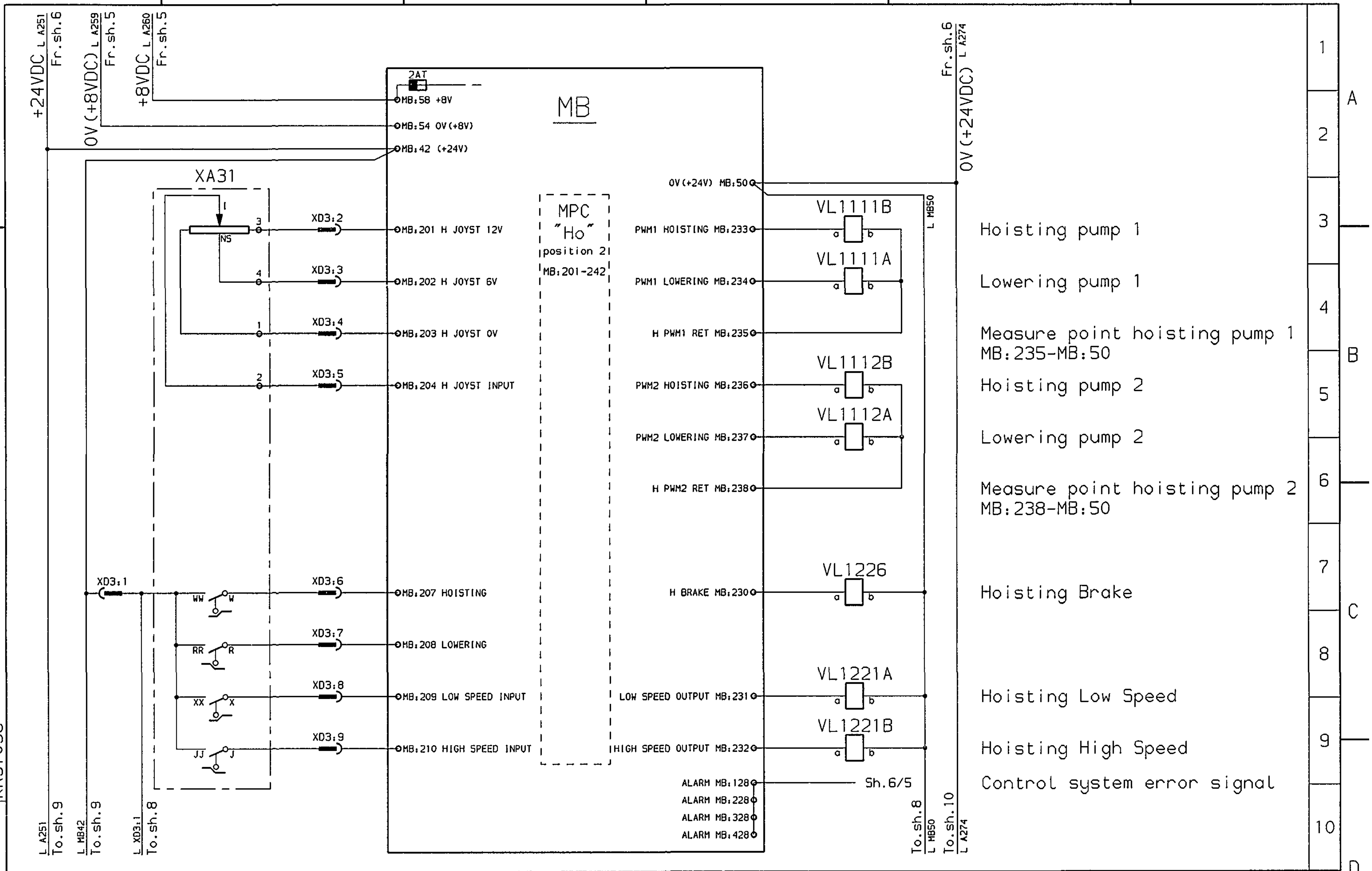

This document must not be copied without our written permission, and the contents thereof must not be imported to a third party nor be used for any unauthorized purpose. Contravention will be prosecuted.

Reference: KR97038

Similar drawing no.: 314 3837

Rev ind	Revision	Appd	Year	Week
A	383683	LBL	97	43

MacGREGOR HÄGGLUNDS
MEMBER OF THE INCENTIVE GROUP

Description (own language)	Description (English)	Prod.group
Krets schema	Circuit diagram	628

Design checked by	Drawing checked by	Drwg no.	Rev ind	Sheet
ASG	ASG	314 3905	A	7

Dept	Drawn by	Year	Week	No of sh
421	A Sundberg	97	36	11

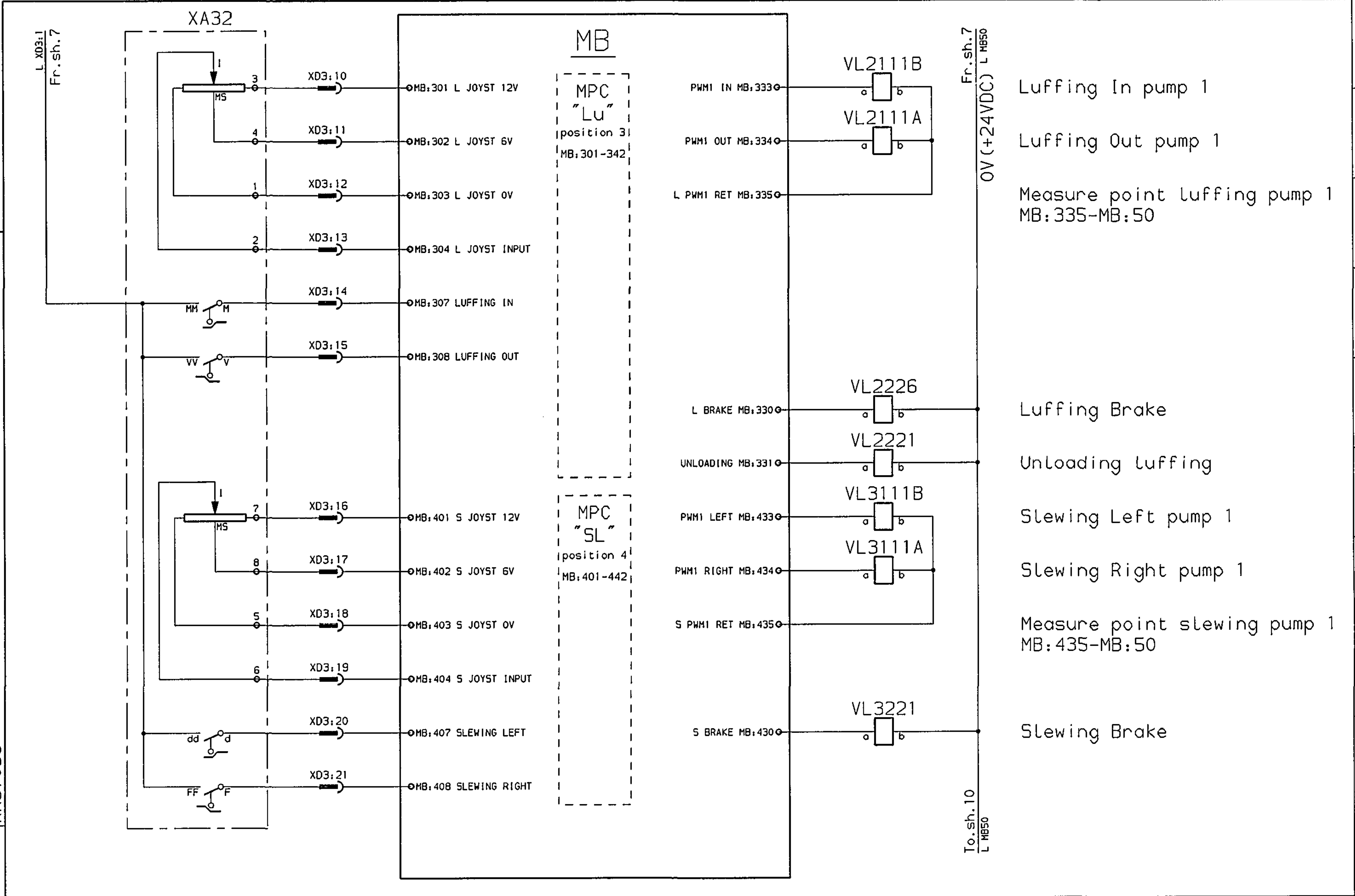

This document must not be copied without our written permission, and the contents thereof must not be imparted to a third party nor be used for any unauthorized purpose. Contravention will be prosecuted.

Reference: KR97038

Similar drawing no.: 314 3837

Rev ind	Revision	Appd	Year Week
A	383683	LBL	97 43

MacGREGOR HÄGGLUNDS
MEMBER OF THE INCENTIVE GROUP

Description (own language)	Krets schema
Description (English)	Circuit diagram
Prod.group	628
Design checked by	ASG
Drawing checked by	ASG
Dept	421
Drawn by	A Sundberg
Year Week	97 36
Drwg no.	314 3905
Rev ind	A
Sheet	8
No of sh	11

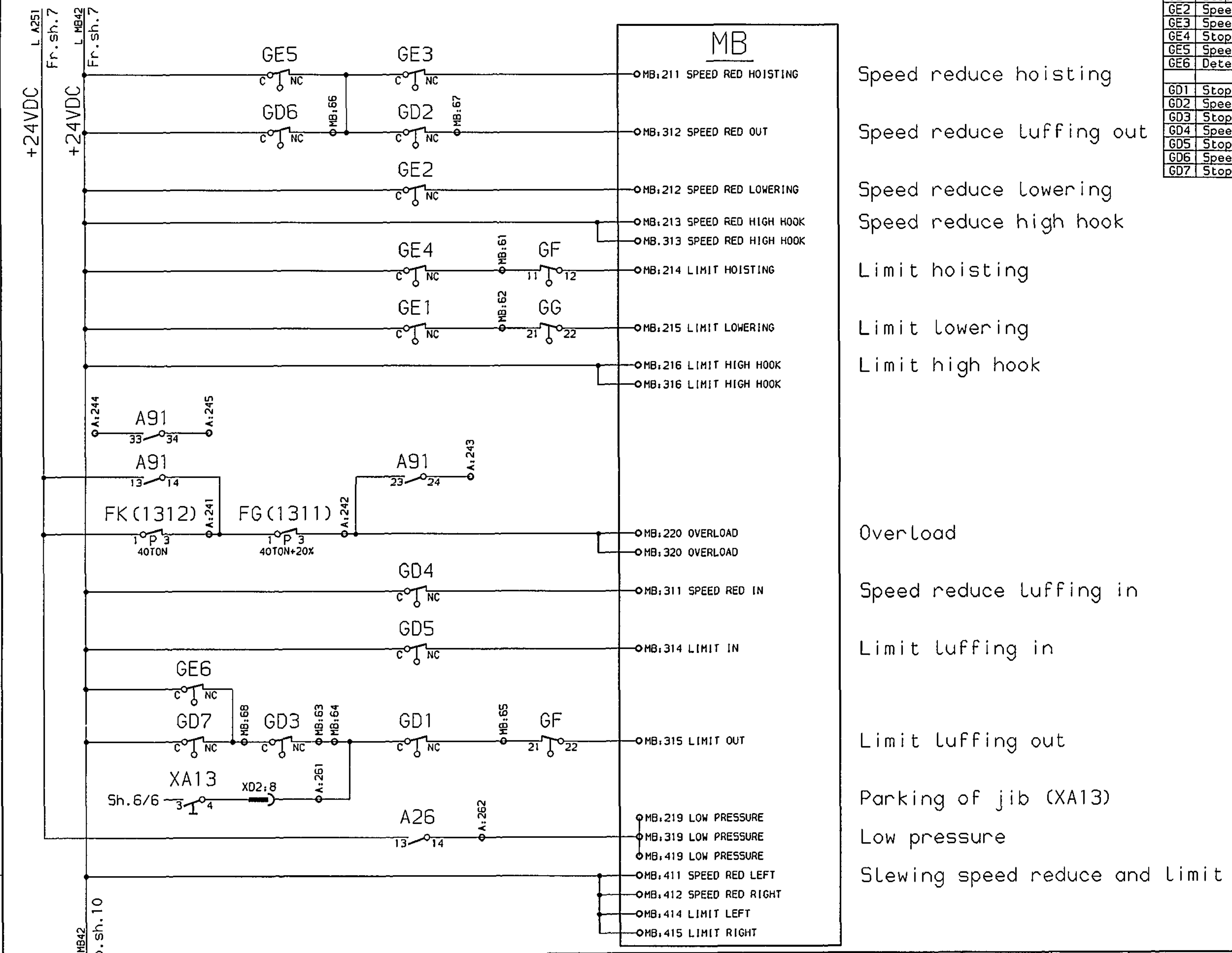

GE1	Stop empty drum
GE2	Speed red. empty drum
GE3	Speed red. full drum
GE4	Stop full drum
GE5	Speed red. high hook
GE6	Detector high hook
GD1	Stop parking outreach
GD2	Speed red. max. outreach
GD3	Stop max. outreach
GD4	Speed red. min. outreach
GD5	Stop min. outreach
GD6	Speed red. high hook outre.
GD7	Stop high hook outre.

This document must not be copied without our written permission, and the contents thereof must not be imparted to a third party nor be used for any unauthorized purpose. Contravention will be prosecuted.

Reference: KR97038

Similar drawing no.: 314 3837

Rev ind	Revision	Appd	Year	Week
A	383683	LBL	97	43

MacGREGOR HÄGGLUNDS
MEMBER OF THE INCENTIVE GROUP

Description (own language)	Description (English)	Prod.group
Krets schema	Circuit diagram	628

Design checked by: ASG — Drawing checked by: ASG
Dept: 421 — Drawn by: A Sundberg — Year Week: 97 36
Drwg no.: 314 3905 — Rev ind: A — Sheet: 9 — No of sh: 11

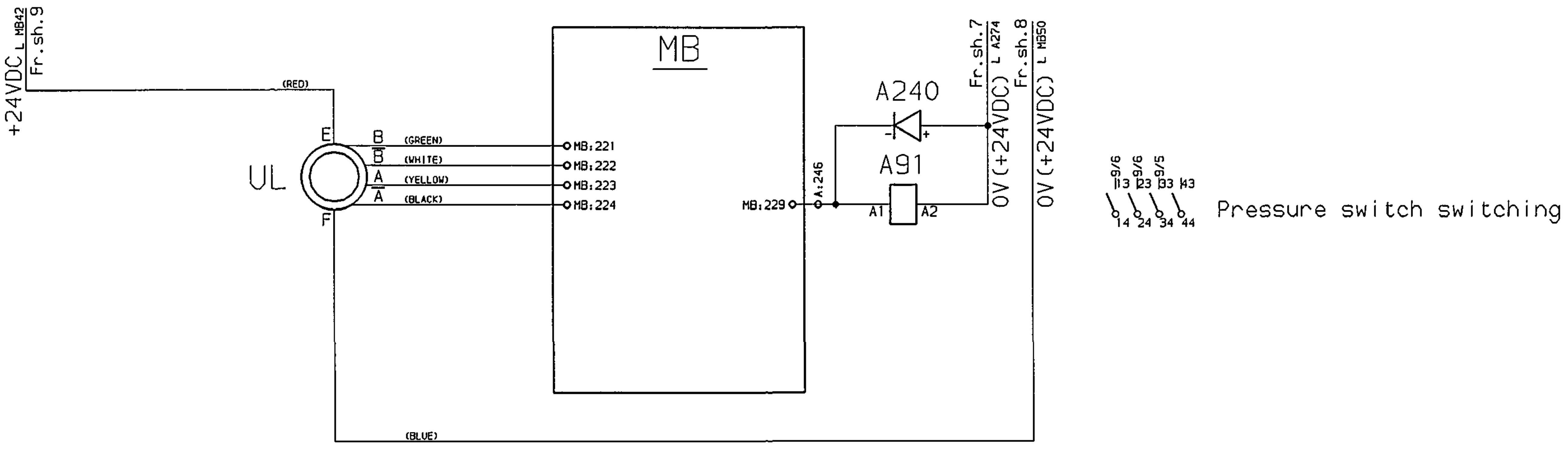

This document must not be copied without our written permission, and the contents thereof must not be imparted to a third party nor be used for any unauthorized purpose. Contravention will be prosecuted.

Reference: KR97038

Similar drawing no.: 314 3837

Rev Ind	Revision	Appd	Year	Week
A	383683	LBL	97	43

MacGREGOR HÄGGLUNDS
MEMBER OF THE INCENTIVE GROUP

Description (own language)	Description (English)	Prod.group
Krets schema	Circuit diagram	628

Design checked by	Drawing checked by	Drwg no.	Rev ind	Sheet	No of sh
ASG	ASG	314 3905	A	10	11

Dept	Drawn by	Year	Week
421	A Sundberg	97	36

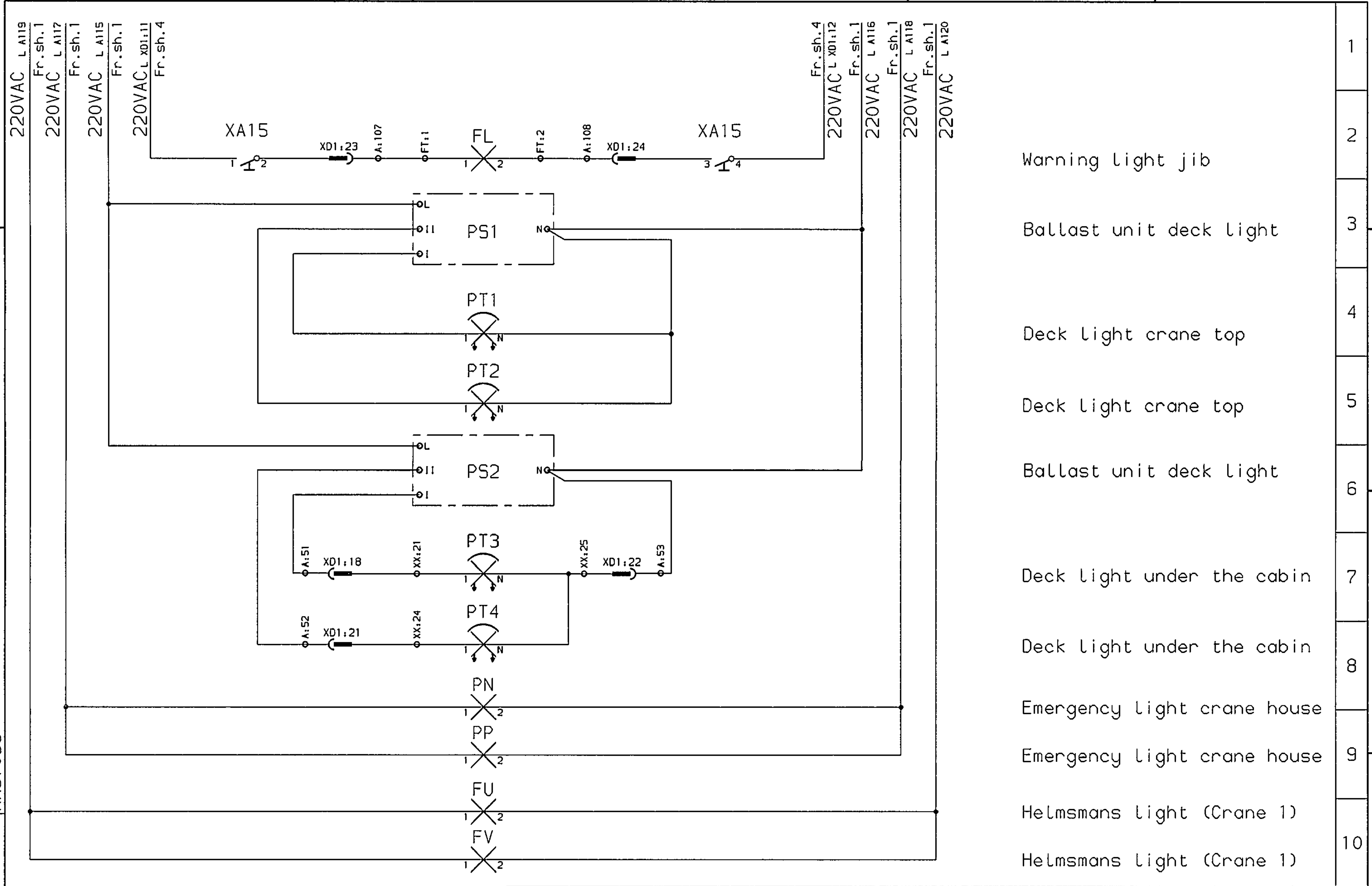

This document must not be copied without our written permission, and the contents thereof must not be imparted to a third party nor be used for any unauthorized purpose. Contravention will be prosecuted.

Reference: KR97038

Similar drawing no.: 314 3837

Rev ind	Revision	Appd	Year Week
A	383683	LBL	97 43

MacGREGOR HÄGGLUNDS
MEMBER OF THE INCENTIVE GROUP

Description (own language)	Krets schema
Description (English)	Circuit diagram
Prod.group	628
Design checked by	ASG
Drawing checked by	ASG
Dept	421
Drawn by	A Sundberg
Year Week	97 40
Drwg no.	314 3905
Rev ind	A
Sheet	11
No of sh	11

Item	Qty	Data	Description and function	Type No or cataloque No	Data	Article No Drawing No	Remarks
A			Cubicle switch board			214 1802-801	
A1.			Star/Delta starter	DEHS 210 ABB	110-150 A 220-240 V 50/60 Hz	5591 2148-150	
A1.K1			Contactor	EH 210 ABB	220-240 V 50/60 Hz	5354 4776-250	N
A1.K2			Contactor	EH100 ABB	220-240 V 50/60 Hz	5354 4776-136	Y
A1.K3		A1.K1	Contactor				D
A1.F3			Overcurrent relay	T200 DU150 ABB	110-150 A I_{set}: 140 A	5627 4215-204	
A1.K9			Time relay	C461.13 ABB	110-240 V,50/60 Hz, 1,5-30s T_{set}: 8,0 s	5632 4208-001	
A4			Contactor	B9-30-10ABB	220-240 V 50/60 Hz	5354 4272-090	
A7			Contactor	K40E ABB	220-240 V 50/60 Hz	5354 4131-400	
A7.1			Auxiliary contact	4xCA7-01 ABB		5354 4136-701	
A8		A4	Contactor				
A13		A7	Contactor				
A13.1		A7.1	Auxiliary contact				
A21			Contactor	B50-30-11 ABB	220-240 V 50/60 Hz	5354 4272-406	
A21.1		A7.1	Auxiliary contact				
A21.2			Auxiliary contact	CA7-10 ABB		5354 4136-710	
A22		A21	Contactor				
A22.1		A7.1	Auxiliary contact				
A22.2		A21.2	Auxiliary contact				
A23		A21	Contactor				
A23.1		A7.1	Auxiliary contact				
A23.2		A21.2	Auxiliary contact				

This document must not be copied without our written permission, and the contents thereof must not be imparted to a third party nor be used for any unauthorized purpose. Contravention will be prosectued.

Item	Qty	Data	Description and function	Type No or cataloque No	Data	Article No Drawing No	Remarks
A24<br>A24.1		A7<br>A7.1	Contactor<br>Auxiliary contact				
A26<br>A26.1		A7	Contactor<br>Time delayer	TP40I ABB	0,1-40s Tset: 2,0 s	5354 4134-008	
A27<br>A27.1		A7<br>A90.1	Contactor<br>Time delayer		T_{set}: 8,0 s		
A34<br>A36<br>A38<br>A41			Switch<br>Hour meter<br>Transformer<br>Transformer	PR12 BACO<br>BG4017-1248<br>Nordtrafo TT201<br>Nordtrafo	"Feed pump motor"<br>12-48 VDC<br>300 VA, 220VAC/24VAC<br>380/400/440-230V, 600 VA	414 4627-801<br>5692 4740-024<br>314 3452-801<br>314 3675-801	
A42<br>A43			Circuit breaker<br>Thermistor	S721-K6 ABB<br>Siemens	6A	5672 4248-006<br>414 1941-801	
A47<br>A48<br>A49		 A7<br>A47	Contactor<br>Contactor<br>Contactor	K22E ABB	220-240 V 50/60 Hz	5354 4131-220	
A70			Emergency stop	BACO		5372 2449-233	
A90<br>A90.1		A4	Contactor<br>Time delayer	TP40D ABB	0,1-40s T_{set}: 5,0 s	5354 4134-007	
A91<br>A99			Contactor<br>Thermostat	KC40E ABB<br>TC1W20R	24 VDC<br>+20°- +110°C Temp set:+25°	5354 4132-401<br>7635 4113-200	
AC			Slipring device	Kraus		314 3481-801	

This document must not be copied without our written permission, and the contents thereof must not be imparted to a third party nor be used for any unauthorized purpose. Contravention will be prosectued.

Item	Qty	Data	Description and function	Type No or cataloque No	Data	Article No<br>Drawing No	Remarks
CE			Power supply	Nya elbolaget Örnsköldsvik		314 3437-801	
CE1-3			Fuse	5x25 mm	2A A/M	5672 4214-120	
CE4-6			Fuse	5x25 mm	10A A/F	5672 4214-210	
DA			Fan motor	Brook Crompton	4pol 1,5kW 3,7A 440V/60 Hz<br>4pol 1,1kW 3,0A 380V/50Hz	287 6956-801	
DB			Main motor	M2CA 315 SMA4 ABB	150kW 243 A 440V/60 Hz<br>132kW 252 A 380V/50 Hz	388 6313-801	
DD			Feed pump motor	Ziehl-Abegg HYDTA 132.44-4/2	2pol 20kW 37,5A 440 V/60 Hz<br>4pol 13kW 29,5A 440 V/60 Hz<br>2pol 17kW 37A 380 V/50 Hz<br>4pol 11kW 29A 380 V/50 Hz	388 0369-801	
DF			Fan motor	Ziehl-Abegg	4pol 0,29kW 0,54A 440 V/60 Hz<br>4pol 0,21kW 0,41A 380 V/50 Hz	388 9464-801	
EA			*Junction box*	Weidmüller		314 3436-801	
EM			Junction box	MA 4982		5388 4121-010	
EN		EM	Junction box				
EP		PR	Junction box				
F			*Siren*	CEAD/Weidmüller	230 V 50/60 Hz	414 6769-801	
FB			Temp guard	TC1A 19R F001	20-95°C, set +85°C	7635 4112-119	
FD			Float switch	Bühler		287 8856-803	
FE (4145)			Pressure switch				
FG (1311)			Pressure switch				
FH (1381)			Pressure switch				
FJ (2481)			Pressure switch				

This document must not be copied without our written permission, and the contents thereof must not be imparted to a third party nor be used for any unauthorized purpose. Contravention will be prosecuted.

Item	Qty	Data	Description and function	Type No or cataloque No	Data	Article No Drawing No	Remarks
FK (1312)			Pressure switch				
FL			Armature	MA 279 red	B22, 220 V, 60 Hz	5915 4251-021	
FL.1			Lamp bulb		B22, 220 V, 60 Hz	5911 4528-066	
FT		PR	Junction box				
FU			Helmsmans light				Yard supply
FV		FU	Helmsmans light				Yard supply
GB			1-phase wall outlet	2-pol	10A/250 V	5381 4280-001	
GD			Limit switch, luffing				
GE			Limit switch, hoisting				
GF			Limit switch	ZCK-J Telemecanique		5661 4126-001	
GG			Limit switch	XCK-J 50511 Telemecanique		5661 4116-512	
GL1			Impuls unit	IFM II0096 IIA 2010-BBOA	220 VAC (breaking)	5661 4214-011	
GL2			Impuls unit	IFM II0011 IIA 2010-ABOA	220 VAC (closing)	5661 4214-010	
GL3		GL1	Impuls unit				
GL4		GL2	Impuls unit				
GL5		GL2	Impuls unit				
HA			Enclosed breaker	OETL 400 ABB	400 A	5381 4226-001	
HC.1			Motor switch	MS 325-4 ABB	2,5-4,0 A, I_{set}: 3,7A 440V/60Hz<br>3,0 A 380V/50Hz	5572 4100-400	
HC.1.1			Auxiliary contact	HK ABB		5218 3110-011	
HC.2			Motor switch	MS 325-0,63 ABB	0,4-0,63 A,I_{set}:0,54A 440V/60Hz<br>0,41A 380V/50Hz	5572 4100-063	
HC.2.1		HC.1.1	Auxiliary contact				
HC.3			Motor switch	MS 325-1 ABB	0,63-1,0 A, I_{set}: 0,8A 440V/60Hz<br>0,91A 380V/50Hz	5572 4100-100	

This document must not be copied without our written permission, and the contents thereof must not be imparted to a third party nor be used for any unauthorized purpose. Contravention will be prosectued.

Item	Qty	Data	Description and function	Type No or cataloque No	Data	Article No Drawing No	Remarks
HC.4			Motor switch	MS 325-1,6 ABB	1,0-1,6 A I_{set}: 1,36A 440V/60Hz<br>1,58A 380V/50 Hz	5572 4100-160	
HC.5			Motor switch	M63-TM40 ABB	28-40 A, A, I_{set}:32A 440V/60Hz<br>32A 380V/50Hz	5572 4101-040	
MB<br>MB.1<br>MB.2.1<br>MB.2.2		 <br><br>MB.2.1	Electronic box<br>Control system CC2000<br>Heater element<br>Heater element	Nya elbolaget Örnsköldsvik<br><br>Rittal SK 3116-SJ	 <br>24V/50 W	314 3500-801<br>314 3499-801<br>414 5351-801	
PA<br>PD<br>PD.1			Switch<br>Armature<br>Flourecent tube	BACO<br>ABB Asea Skandia	 2x18W, 220 V 50/60 Hz<br>18 W	5375 4231-024<br>5915 4315-001<br>5912 4102-020	
PE<br>PE.1			Headlight<br>Sodium lamp	Aqua signal	400 W, 220 V, E40<br>400 W, 220-230 V, E40	5921 2104-101<br>5912 4128-400	
PF<br>PF.1		PD<br>PD.1	Armature<br>Flourecent tube				
PN<br>PN.1		XC<br>XC.1	Armature<br>Lamp bulb				
PP<br>PP.1		XC<br>XC.1	Armature<br>Lamp bulb				
PR			Ballast unit	Aqua signal	1x400 W 220 V/60 Hz	5921 2104-901	
PT1<br>PT1.1			Headlight<br>Sodium lamp				Yard supply<br>Yard supply
PT2<br>PT2.1		PT.1<br>PT.1.1	Headlight<br>Sodium lamp				
PT3<br>PT3.1		PT.1<br>PT.1.1	Headlight<br>Sodium lamp				

This document must not be copied without our written permission, and the contents thereof must not be imparted to a third party nor be used for any unauthorized purpose. Contravention will be prosecuted.

Item	Qty	Data	Description and function	Type No or cataloque No	Data	Article No<br>Drawing No	Remarks
PT4<br>PT4.1		PT.1<br>PT.1.1	Headlight<br>Sodium lamp				
UL			Puls unit	Leine&Linde (Hägglunds Drives)		376 0052-801	
X<br>XA1<br>XA1.1<br>XA1.2			Driver desk<br>Indicator head<br>Bulb<br>Lamp socket	 V10 SA10 red BACO<br><br>233 EAT BACO	 "Do not operate "<br>24 V, 2 W, BA9s	114 1210-801<br>5298 2057-001<br>5911 4509-002<br>5372 2017-001	
XA2<br>XA2.1<br>XA2.2		XA1<br>XA1.1<br>XA1.2	Indicator head<br>Bulb<br>Lamp socket		"Low oil level"		
XA3<br>XA3.1<br>XA3.2		XA1<br>XA1.1<br>XA1.2	Indicator head<br>Bulb<br>Lamp socket		"Check oil filter"		
XA4<br>XA4.1<br>XA4.2		XA1<br>XA1.1<br>XA1.2	Indicator head<br>Bulb<br>Lamp socket		"Control system error in MB-box"		
XA10<br>XA10.1			Push button<br>Switch block	0/1 BACO<br>233 E11 BACO	(Stop/Start)	5372 2040-001<br>5372 2016-006	
XA11<br>XA11.1			Push button<br>Switch block	C23 AD03 black BACO<br>233 E20 BACO	"Bell"	5372 2039-003<br>5372 2016-005	
XA12<br>XA13<br>XA15			Switch<br>Switch<br>Switch	PR12 BACO<br>PR12 BACO<br>PR12 BACO	"Float switch"<br>"Parking of jib"<br>"Warning light jib"	414 4622-801<br>414 4628-801<br>414 5482-801	

This document must not be copied without our written permission, and the contents thereof must not be imparted to a third party nor be used for any unauthorized purpose. Contravention will be prosecuted.

Item	Qty	Data	Description and function	Type No or cataloque No	Data	Article No Drawing No	Remarks
XA16			Switch	PR12 BACO	"Heater cabin"	414 4625-801	
XA17			Switch	PR12 BACO	"Cabin light"	414 4621-801	
XA18			Switch	PR12 BACO	"Floodlight"	414 4620-801	
XA19			Switch	PR12 BACO	"Wiper"	414 4626-801	
XA20			Fuse	33-2040-S	"Reset wiper" 2A	5672 4245-002	
XA21		A70	Push button				
XA31			Controller	Siemens/Spohn & Burkhardt		314 2005-802	
XA32			Controller	Siemens/Spohn & Burkhardt		314 2006-802	
XB			Operating switch box			314 3840-801	
XB1		XA1	Indicator head		"Parking lock engaged"		
XB1.1		XA1.1	Bulb				
XB1.2		XA1.2	Lamp socket				
XB2		XA1	Indicator head		"Parking lock not engaged"		
XB2.1		XA1.1	Bulb				
XB2.2		XA1.2	Lamp socket				
XB3			Switch	PR 12 BACO	"Parking slewing lock"	414 5598-801	
XB4			Switch	PR 12 BACO	"Lock pins"	414 6914-801	
XB5			Indicator head		"Parking position slewing lock"	5298 2057-004	
XB5.1		XA1.1	Bulb				
XB5.2		XA1.2	Lamp socket				
XB6			Alarm (sound)	118 068 28		5931 4108-828	
XC			Armature	Asea Skandia	60 W	5915 4262-002	
XC.1			Bulb		220 V, 60 W E27	5911 4145-060	
XD1.1			Plug hood	Weidmüller		5217 4833-240	
XD1.2			Screw connection	Weidmüller	380 V AC/16 A	5217 4834-240	

This document must not be copied without our written permission, and the contents thereof must not be imparted to a third party nor be used for any unauthorized purpose. Contravention will be prosecuted.

Item	Qty	Data	Description and function	Type No or cataloque No	Data	Article No Drawing No	Remarks
XD1.3			Screw connection	Weidmüller	380 V AC/16 A	5217 4835-240	
XD1.4			Bulkhead housing	Weidmüller		5217 4836-240	
XD2.1		XD1.1	Plug hood				
XD2.2		XD1.2	Screw connection				
XD2.3		XD1.3	Screw connection				
XD2.4		XD1.4	Bulkhead housing				
XD3.1		XD1.1	Plug hood				
XD3.2		XD1.2	Screw connection				
XD3.3		XD1.3	Screw connection				
XD3.4		XD1.4	Bulkhead housing				
XD5.1		XD1.1	Plug hood				
XD5.2		XD1.2	Screw connection				
XD5.3		XD1.3	Screw connection				
XD5.4		XD1.4	Bulkhead housing				
XE			Wiper			387 6227-802	
XG			Cabin heater	Calix KK7	0-2000 W, 220 V	6678 2021-007	
XX			Terminal strip	Weidmüller	WDU 2,5	314 3632-801	
XX30			Diode plinth	Weidmüller	1A	2669 4190-025	

This document must not be copied without our written permission, and the contents thereof must not be imparted to a third party nor be used for any unauthorized purpose. Contravention will be prosecuted.

TOL. SYSTEM ACC. TO ISO	General tol. for dimensions without tol. indications
Surface roughness R_a in µm	Machining ISO 2768 tol-class:
	Welding DIN 8570 tol-class:

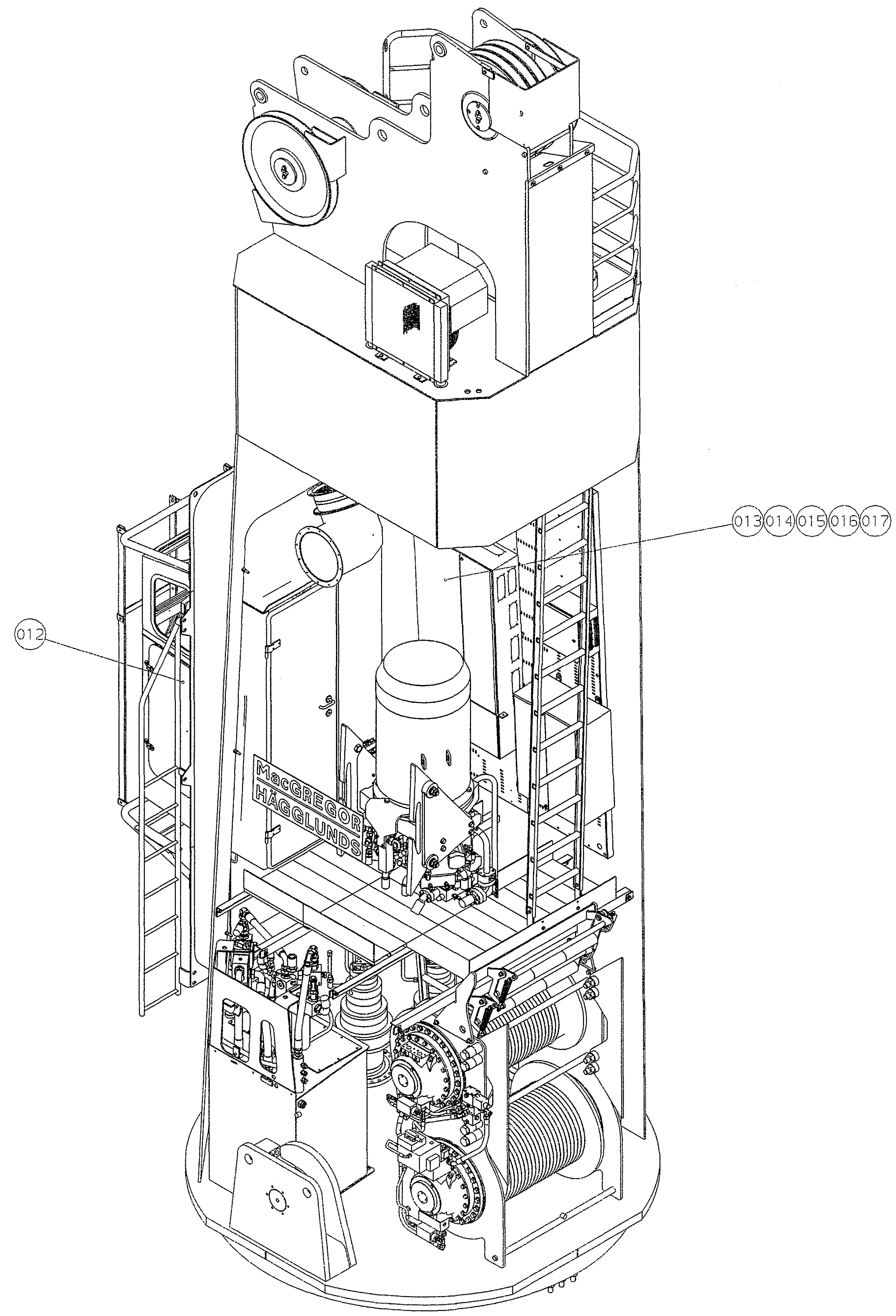

Item no.	Qty/801	Article no.	Description (own language)	Dimensions	Description (English)
017	1	114 1314-801	El inst. kranarmsbel.		El. inst. jib li
016	1	114 1225-801	El varn.skylt plac.		El. warning sign plac.
015	1	114 1224-801	El inst. kranhus		El. inst. craneh
014	1	214 1803-801	El panel		El. panel
013	1	114 1204-801	Elpanel kablering		El. panel cabling
012	X	114 1210-801	Förarpulpet		Driver desk
011					
010					
009	X	414 6770-000	Förb.tab. strålk.		Wiring tab. fl.light jib
008	X	414 6768-000	Förb. tabell hytt		Wiring table cabin
007	X	414 6714-000	Förb. tabell A-skåp		Wiring table A cubicle
006	X	414 6969-000	Förb.tabeli elpanel		Wiring table el. panel

Design checked by: ASG	Accepted by qual dept	Part of: Order spec	
Drawing checked by: ASG	Accepted for prod by: MN	Specification	Scale: 1:20
Dept: 421	Drawn by: A Sundberg/LB — Year Week: 97 22	Description (English): El. assembly GL-	Weight kg
MacGREGOR HÄGGLUNDS — MEMBER OF THE INCENTIVE GROUP		Description (own language): El smst. GL-kran	Prod.group: 628
		Drwg no.: 114 1324	Rev ind — Sheet: 1 — No of sh: 1

Revind	Revision	Appd	Year	Week

Similar drawing No.

Reference: KR96050

This document must not be copied without our written permission, and the contents thereof must not be imparted to a third party nor be used for any unauthorized purpose. Contravention will be prosecuted.

TOL. SYSTEM ACC. TO ISO	General tol. for dimensions without tol. indications
Surface roughness R_a in µm	Machining ISO 2768 tol-class:
	Welding DIN 8570 tol-class:

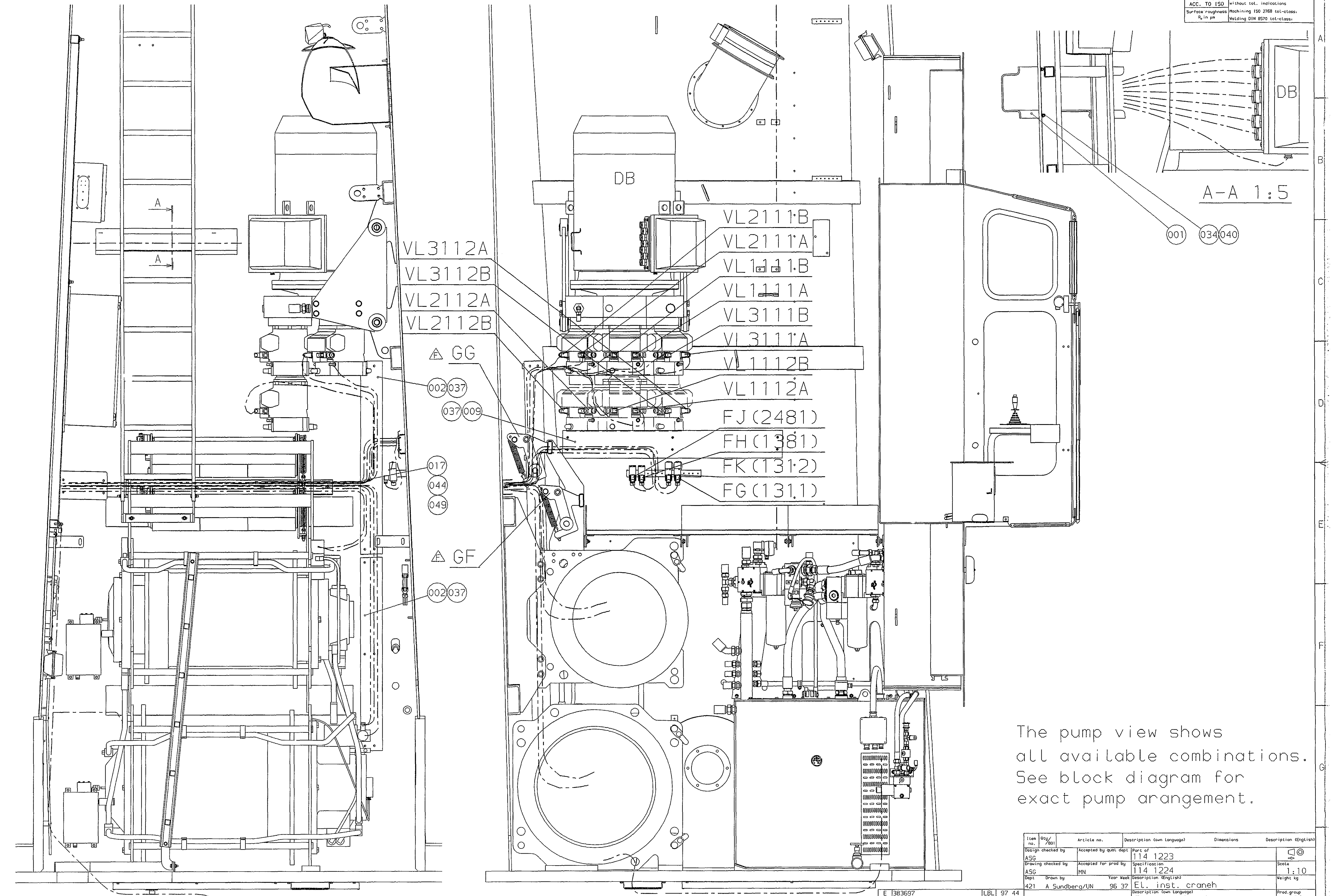

The pump view shows all available combinations. See block diagram for exact pump arangement.

Item no.	Qty/801	Article no.	Description (own language)	Dimensions	Description (English)

Design checked by	ASG
Accepted by qual dept	
Drawing checked by	ASG
Accepted for prod by	MN
Dept	421
Drawn by	A Sundberg/UN
Year Week	96 37
Part of	114 1223
Specification	114 1224
Scale	1:10
Description (English)	El. inst. craneh
Weight kg	
Description (own language)	El inst. kranhus
Prod.group	628
Drwg no.	114 1224
Rev ind	F
Sheet	1
No of sh	4

Revind	Revision	Appd	Year	Week
F	383863	LBL	98	03
E	383697	LBL	97	44
D	383631	LBL	97	41
C	383482	LBL	97	34
B	383202	LBL	97	12
A	382921	UNN	96	47

MacGREGOR HÄGGLUNDS
MEMBER OF THE INCENTIVE GROUP

Reference KP96000
Similar drawing No.

TOL. SYSTEM ACC. TO ISO	General tol. for dimensions without tol. indications
Surface roughness R_a in µm	Machining ISO 2768 tol-class:
	Welding DIN 8570 tol-class:

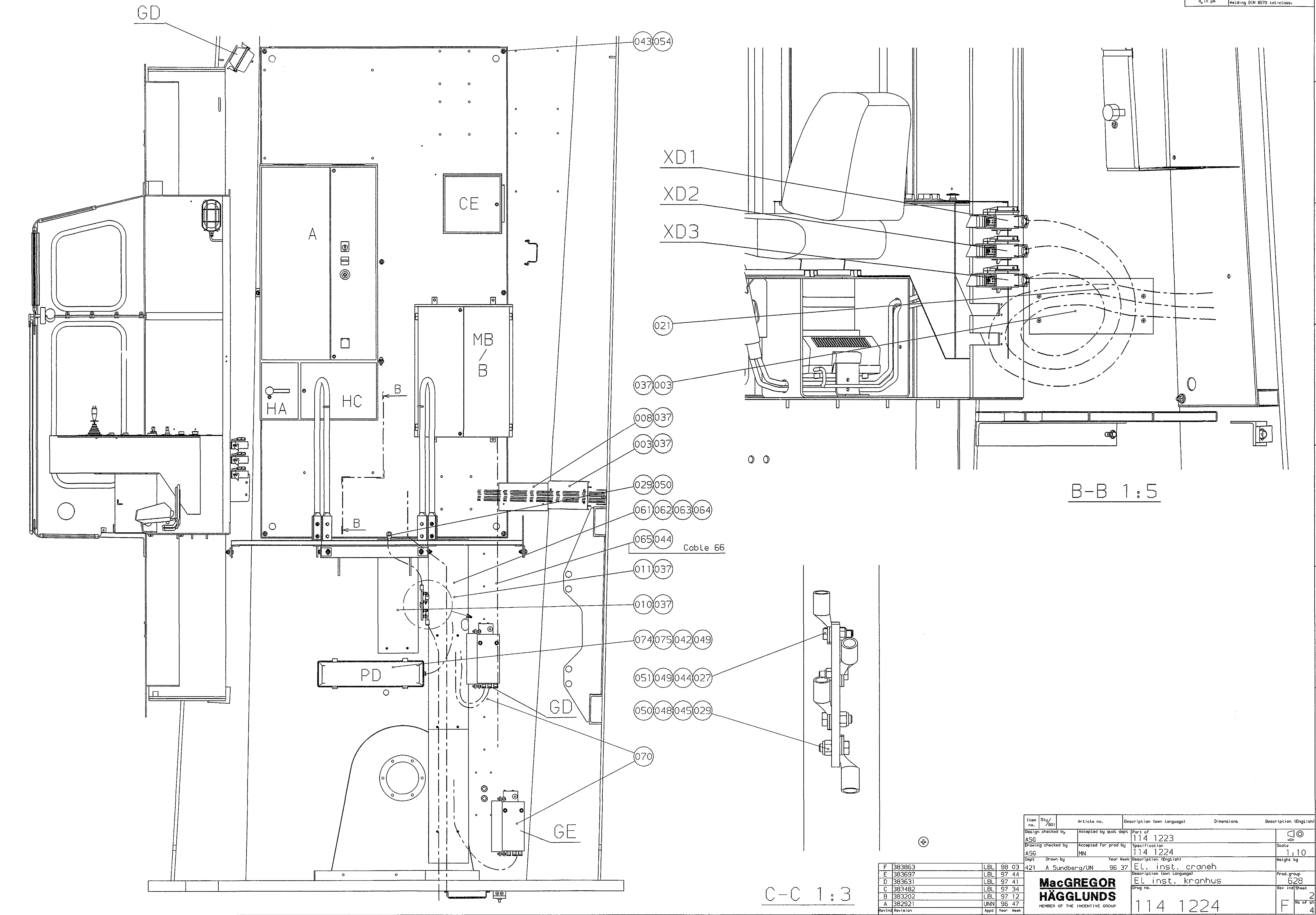

Item no.	Qty/801	Article no.	Description (own language)	Dimensions	Description (English)

Design checked by: ASG	Accepted by qual dept	Part of: 114 1223	
Drawing checked by: ASG	Accepted for prod by: MN	Specification: 114 1224	Scale: 1:10
Dept: 421	Drawn by: A Sundberg/UN, Year Week: 96 37	Description (English): El. inst. craneh	Weight kg
MacGREGOR HÄGGLUNDS MEMBER OF THE INCENTIVE GROUP		Description (own language): El inst. kranhus	Prod.group: 628
		Drwg no.: 114 1224	Rev ind: F; Sheet: 2; No of sh: 4

Rev ind	Revision	Appd	Year	Week
F	383863	LBL	98	03
E	383697	LBL	97	44
D	383631	LBL	97	41
C	383482	LBL	97	34
B	383202	LBL	97	12
A	382921	UNN	96	47

Reference KR96000

TOL. SYSTEM ACC. TO ISO	General tol. for dimensions without tol. indications
Surface roughness R_a in μm	Machining ISO 2768 tol-class:
	Welding DIN 8570 tol-class:

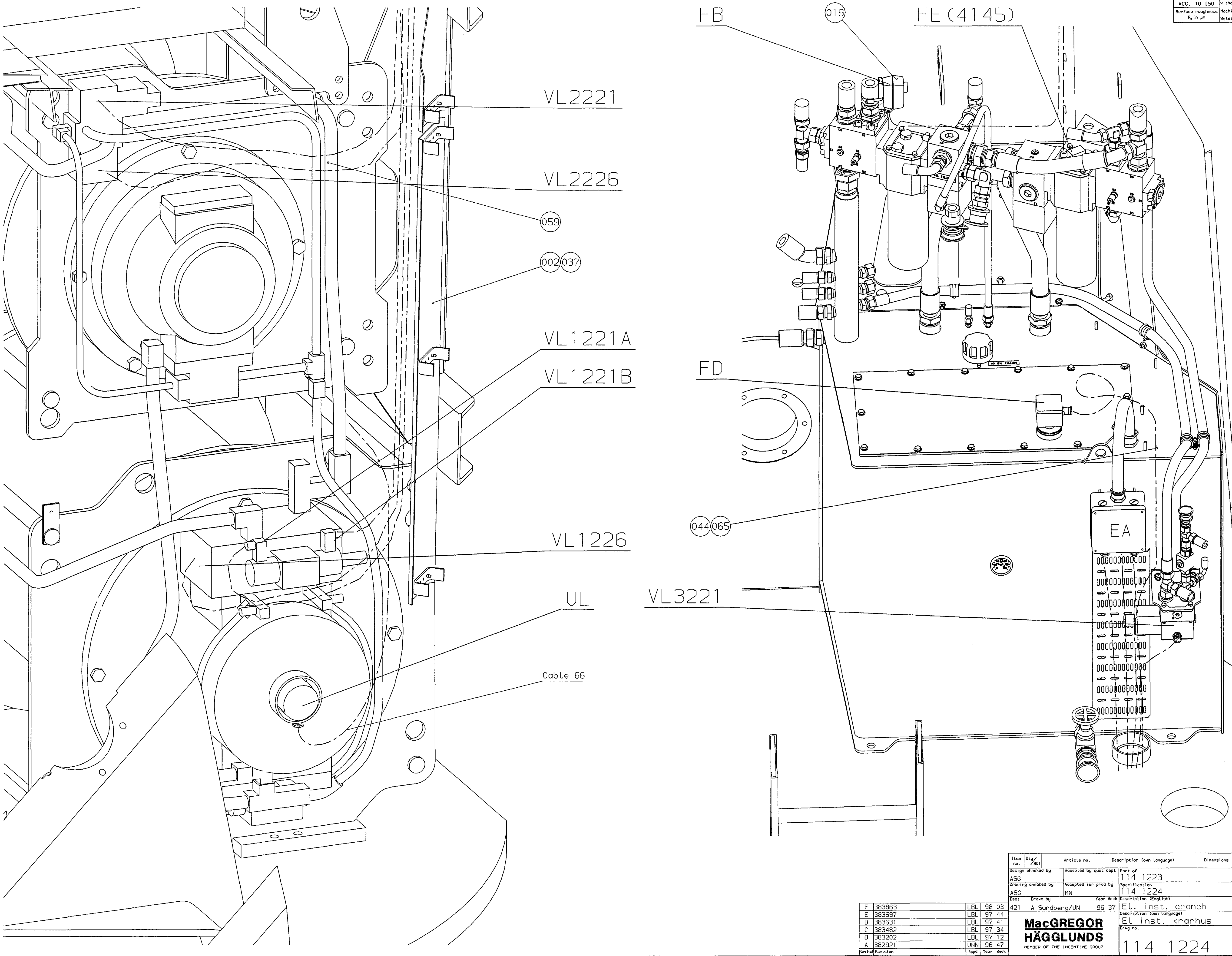

Item no.	Qty/801	Article no.	Description (own language)	Dimensions	Description (English)

Design checked by: ASG	Accepted by qual dept	Part of: 114 1223	
Drawing checked by: ASG	Accepted for prod by: MN	Specification: 114 1224	Scale: 1:10
Dept: 421	Drawn by: A Sundberg/UN, Year Week: 96 37	Description (English): El. inst. craneh	Weight kg
MacGREGOR HÄGGLUNDS<br>MEMBER OF THE INCENTIVE GROUP		Description (own language): El inst. kranhus	Prod.group: 628
		Drwg no.: 114 1224	Rev ind: F; Sheet: 3; No of sh: 4

Rev Ind	Revision	Appd	Year Week
F	383863	LBL	98 03
E	383697	LBL	97 44
D	383631	LBL	97 41
C	383482	LBL	97 34
B	383202	LBL	97 12
A	382921	UNN	96 47

KR96000

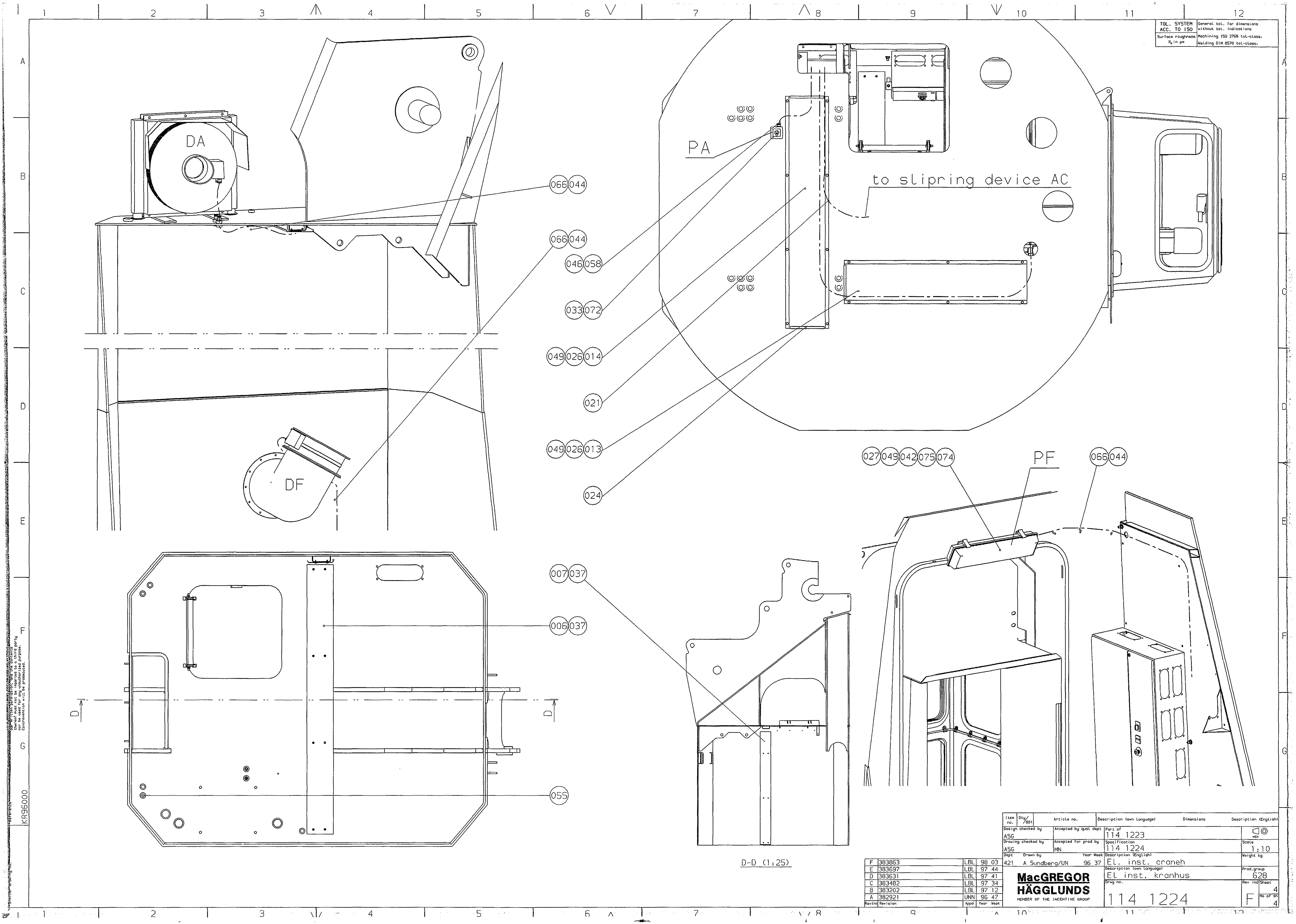

TOL. SYSTEM ACC. TO ISO	General tol. for dimensions without tol. indications
Surface roughness R_a in µm	Machining ISO 2768 tol-class:
	Welding DIN 8570 tol-class:

Item no.	Qty/801	Article no.	Description (own language)	Dimensions	Description (English)

Rev Ind	Revision	Appd	Year	Week
F	383863	LBL	98	03
E	383697	LBL	97	44
D	383631	LBL	97	41
C	383482	LBL	97	34
B	383202	LBL	97	12
A	382921	UNN	96	47

Design checked by: ASG	Accepted by qual dept:	Part of: 114 1223	
Drawing checked by: ASG	Accepted for prod by: MN	Specification: 114 1224	Scale: 1:10
Dept: 421	Drawn by: A Sundberg/UN; Year Week: 96 37	Description (English): El. inst. craneh	Weight kg:
MacGREGOR HÄGGLUNDS MEMBER OF THE INCENTIVE GROUP		Description (own language): El inst. kranhus	Prod.group: 628
		Drwg no.: 114 1224	Rev ind: F; Sheet: 4; No of sh: 4

This document must not be copied without our written permission, and the contents thereof must not be imparted to a third party nor be used for any unauthorized purpose. Contravention will be prosecuted.

Reference: KR96000

Drawing No.

This document must not be copied without our written permission, and the contents thereof must not be imparted to a third party nor be used for any unauthorized purpose. Contravention will be prosecuted.

Item no.	Article no.	Description (own language)	Dimensions	Description (English)	Qty/801	Note
001	114 1228-001	Kabelränna		Cable groove	1	
002	214 1746-118	Kabelränna		Cable groove	2	
003	214 1746-038	Kabelränna		Cable groove	2	
005						
006	214 1746-174	Kabelränna		Cable groove	1	
007	214 1746-198	Kabelränna		Cable groove	1	
008	214 1746-030	Kabelränna		Cable groove	1	
009	214 1746-134	Kabelränna		Cable groove	1	
010	214 1747-070	Kabelränna		Cable groove	1	
011	214 1747-198	Kabelränna		Cable groove	1	
012						
013	214 1754-118	Kabelränna		Cable groove	1	
014	214 1754-150	Kabelränna		Cable groove	1	
015						
016						
017	214 1824-001	Kabelränna		Cable groove	1	
018						
019	414 6799-801	Termostat		Temperature regulator	1	
020						

Rev Ind	Revision	Appd	Year Week
F	383863	LBL	98 03

Design checked by: ASG
Accepted by qual dept: .
Part of: 114 1223
Assembly drawing: 114 1224
Drawing checked by: ASG
Accepted for prod by: MN
Description (English): El. inst. craneh
Dept: 441
Drawn by: Y Jonsson/UN
Year Week: 96 37
Description (own language): El inst. kranhus

MacGREGOR HÄGGLUNDS
MEMBER OF THE INCENTIVE GROUP

Drwg no.: 114 1224
Rev Ind: F
Sheet: 1
No of sh: 4

This document must not be copied without our written permission, and the contents thereof must not be imported to a third party nor be used for any unauthorized purpose. Contravention will be prosecuted.

Item no.	Article no.	Description (own language)	Dimensions	Description (English)	Qty/801	Note
021	388 7631-001	Spännrem		Stretching belt	7	
022						
023						
024	1866 2114-001	Plastprofil U 3x11.5	L=860	Plastic profile	1	
025						
026	2121 2032-451	M6S 8x20-8.8 fzb		Screw	15	
027	2121 2032-455	M6S 8x30 -8.8 fzb		Screw	5	
028						
029	2121 2032-495	M6S 10x30 -8.8 fzb		Screw	3	
030						
031						
032						
033	2121 2550-291	MC6S 4x12 A4-80		Allen screw	2	
034	2121 2550-370	MC6S 6x20 A4-80		Allen screw	4	
035						
036						
037	2124 2501-368	MRX-DUO TAPTITE M6x16		Screw	66	
038						
039						

Rev Ind	Revision	Appd	Year Week
F	383863	LBL	98 03

Design checked by	Accepted by qual dept	Part of	Assembly drawing
ASG	.	114 1223	114 1224

Drawing checked by	Accepted for prod by	Description (English)
ASG	MN	El. inst. craneh

Dept	Drawn by	Year Week	Description (own language)
441	Y Jonsson/UN	96 37	El inst. kranhus

MacGREGOR HÄGGLUNDS
MEMBER OF THE INCENTIVE GROUP

Drwg no.	Rev Ind	Sheet	No of sh
114 1224	F	2	4

This document must not be copied without
our written permission, and the contents
thereof must not be imparted to a third party
nor be used for any unauthorized purpose.
Contravention will be prosecuted.

Item no.	Article no.	Description (own language)	Dimensions	Description (English)	Qty/801	Note
040	2126 2634-116	Låsm-M6M 6-A4-80		Locking nut	4	
041						
042	2126 2636-118	Låsm-M6M 8-8 fzb		Locking nut	4	
043	2126 2636-120	Låsm-M6M 10-8 fzb		Locking nut	7	
044	2126 2032-118	M6M 8 -8 FZB		NUT	52	
045	2126 2032-120	M6M 10 -8 fzb		Nut	2	
046	2126 2911-186	Kontramutter PR18,6		Jam nut	1	
047						
048	2151 2022-173	BRB 10.5x22 fzb		Washer	2	
049	2151 2022-164	BRB 8,4x16 fzb		Washer	22	
050	2154 2033-010	Spännbricka BRIF 10.5 fz		Washer	3	
051	2154 2033-009	Spännbricka BRIF 8,4 fz		Washer	2	
052						
053						
054	2151 2058-173	TBRSB 10.5x28 FZB		WASHER	7	
055	2152 2121-225	Avslutningspropp Pr 22,5		Concluding plug	4	
056						
057						
058	2166 2051-812	Kabelförskr. Pr 18,6		Cable gland	1	

Rev Ind	Revision	Appd	Year Week
F	383863	LBL	98 03

Design checked by: ASG	Accepted by qual dept: .	Part of: 114 1223	Assembly drawing: 114 1224
Drawing checked by: ASG	Accepted for prod by: MN	Description (English): EL. inst. craneh	
Dept: 441	Drawn by: Y Jonsson/UN	Year Week: 96 37	Description (own language): EL inst. kranhus

MacGREGOR HÄGGLUNDS
MEMBER OF THE INCENTIVE GROUP

Drwg no.	Rev Ind	Sheet	No of sh
114 1224	F	3	4

This document must not be copied without our written permission, and the contents thereof must not be imparted to a third party nor be used for any unauthorized purpose. Contravention will be prosecuted.

Item no.	Article no.	Description (own language)	Dimensions	Description (English)	Qty/801	Note
059	2166 2228-172	Spännband SST-2S L=172		Strap	30	
060						
061	2166 2261-225	Spännband AE112S rf		Strap	80	
062	2166 2261-300	Spännband AE113S rf		Strap	20	
063	2166 2261-313	Spännband AE313S rf		Strap	20	
064	2166 2261-450	Spännband AE114S rf		Strap	5	
065	2166 4127-084	Klammer RSGU 8/20		Clamp	8	
066	2166 4127-114	Klammer RSGU 11/20		Clamp	27	
067	2166 4127-154	Klammer RSGU 15/20		Clamp	1	
068						
069						
070	2641 2034-002	Kabelsko 0,4-1,6 M4		Cable terminal	30	
071						
072	5375 4231-024	Kapslad strömbrytare		Switch	1	
073						
074	5912 4102-020	Lysrör 18W		Flouresent tube	4	
075	5915 4315-001	Lysrörsarmatur 2x18W 220V		Armature	2	
076						

Rev Ind	Revision	Appd	Year Week
F	383863	LBL	98 03

Design checked by: ASG	Accepted by qual dept: .	Part of: 114 1223	Assembly drawing: 114 1224
Drawing checked by: ASG	Accepted for prod by: MN	Description (English): El. inst. craneh	
Dept: 441	Drawn by: Y Jonsson/UN; Year Week: 96 37	Description (own language): El inst. kranhus	

MacGREGOR HÄGGLUNDS
MEMBER OF THE INCENTIVE GROUP

Drwg no.	Rev Ind	Sheet	No of sh
114 1224	F	4	4

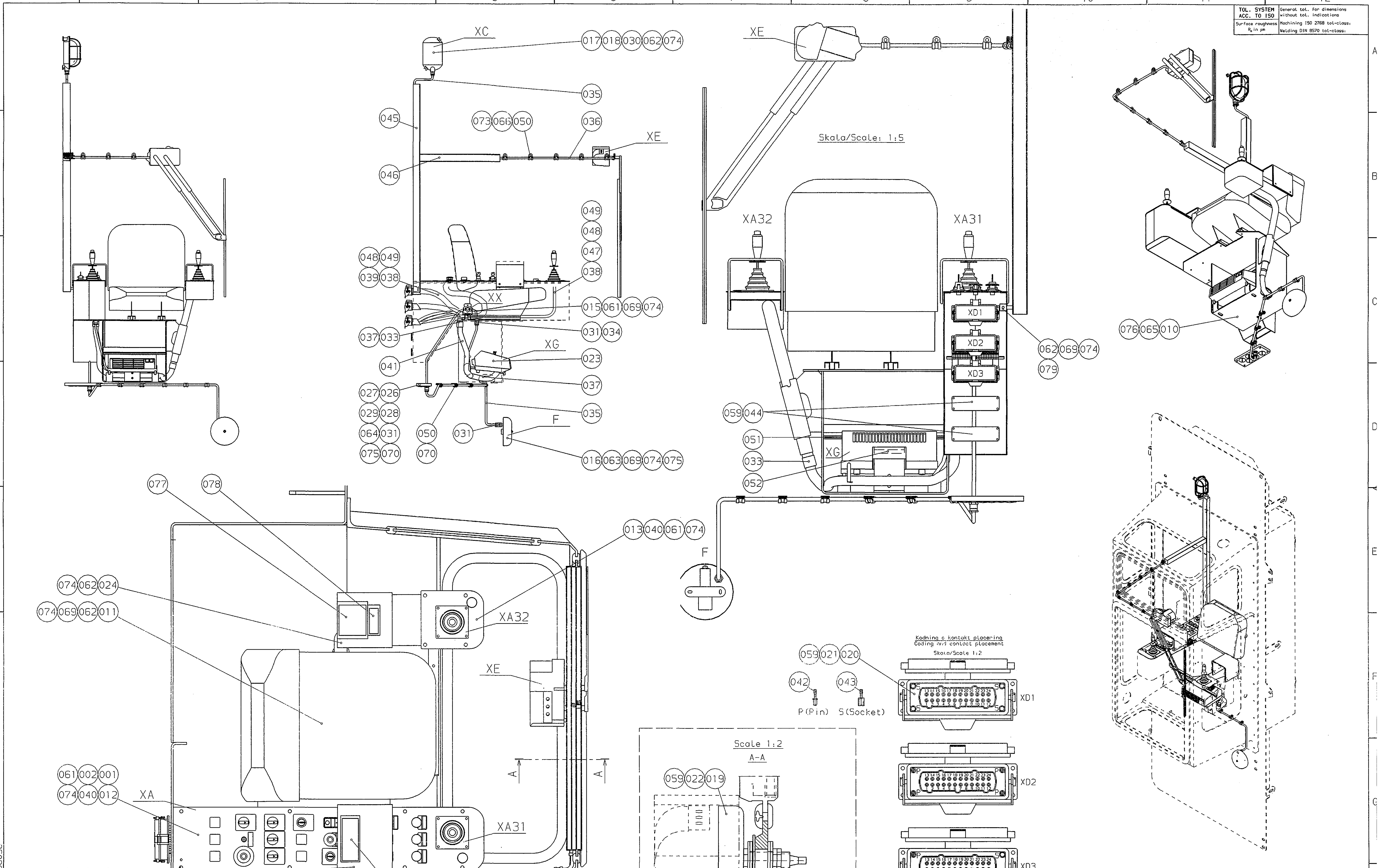

TOL. SYSTEM ACC. TO ISO	General tol. for dimensions without tol. indications
Surface roughness R_a in µm	Machining ISO 2768 tol-class:
	Welding DIN 8570 tol-class:

Item no.	Qty/801	Article no.	Description (own language)	Dimensions	Description (English)

Design checked by: ASG	Accepted by qual dept	Part of	
Drawing checked by: ASG	Accepted for prod by: ÅS	Specification: 114 1236	Scale: 1:10
Dept: 421	Drawn by: A Sundberg — Year Week: 96 40	Description (English): Driver desk	Weight kg
MacGREGOR HÄGGLUNDS — MEMBER OF THE INCENTIVE GROUP		Description (own language): Förarpulpet	Prod.group: 628
		Drwg no.: 114 1210	Rev ind: C — Sheet: 1 — No of sh: 1

RevInd	Revision	Appd	Year	Week
C	383320	LBL	97	21
B	383264	LBL	97	18
A	383166	LBL	97	12

This document must not be copied without our written permission, and the contents thereof must not be imparted to a third party nor be used for any unauthorized purpose. Contravention will be prosecuted.

Item no.	Article no.	Description (own language)	Dimensions	Description (English)	Qty/801	Note
001	414 6768-000	Förb. tabell hytt		Wiring table cabin	X	
010	288 1051-801	Stol montering		Chair assembly	1	
011	488 6922-001	Förarsäte		Drivers seat	1	
012	214 1734-801	Manöverpanel		Control panel	1	
013	414 6792-801	Kontroller panel		Controller panel	1	
015	314 3632-801	Plint rad		Terminal strip	1	
016	414 6769-801	Ringklocka		Bell	1	
017	5915 4262-002	Takarmatur 60 W		Ceiling light	1	
018	5911 4145-060	Lampa matt		Lampbulb	1	
019	387 6227-802	Vindrutetorkare		Windscreen cleaner	1	
020	5217 4835-240	Kontaktdon plint		Screw connection	3	
021	5217 4836-240	Kontaktdonskåpa		Bulkhead housing	3	
022	388 9799-001	Kåpa		Case	1	
023	6678 2021-007	Kupevärmare		Heater	1	
024	287 4867-002	Armstöd		Arm rest	1	
025	288 1058-001	Armstöd		Arm rest	1	
026	5237 4116-637	Rörfläns		Flange	1	
027	5239 2105-210	Flänspackning		Flange gasket	1	
028	2152 2121-370	Avslutningspropp		Concluding plug	5	

Rev Ind	Revision	Appd	Year Week
C	383320	LBL	97 21

Design checked by: ASG	Accepted by qual dept: .	Part of: 187 9937	Assembly drawing: 114 1210
Drawing checked by: ASG	Accepted for prod by: ÅS	Description (English): Driver desk	
Dept: 421	Drawn by: A Sundberg, Year Week: 96 35	Description (own language): Förarpulpet	

MacGREGOR HÄGGLUNDS
MEMBER OF THE INCENTIVE GROUP

Drwg no.	Rev Ind	Sheet	No of sh
114 1210	C	1	4

This document must not be copied without our written permission, and the contents thereof must not be imparted to a third party nor be used for any unauthorized purpose. Contravention will be prosecuted.

Item no.	Article no.	Description (own language)	Dimensions	Description (English)	Qty/801	Note
029	2686 4425-371	Reduc-muff Pr 37,0-22,5		Reducing nipple	1	
030	2166 2051-812	Kabelförskr. Pr 18,6		Cable gland	1	
031	2166 2051-821	Kabelförskr. Pr 22,5		Cable gland	3	
033	2521 2531-283	Muff		Sleeve	2	
034	2126 2911-225	Kontramutter PR22,5		Jam nut	1	
035	1684 2136-123	Elkabel RC 3x1,5	L=5m	Cable	1	
036	1684 2136-124	Elkabel RC 4x1,5	L=4m	Cable	1	
037	2126 2911-283	Kontramutter PR28,3		Jam nut	1	
038	1685 2104-120	Elkabel RK 1,5 svart	L=150m	Cable	1	
039	1685 2104-123	Kabel RK 1.5 grön/gul	L=2m	Cable	1	
040	1685 2104-133	Elkabel RK 2,5 grön/gul	L=3m	Cable	1	
041	1368 2229-290	Spiralslang 29,4	L=1m	Hose	1	
042	414 6785-001	Kodnings pinne		Coding pin	6	
043	414 6790-001	Kodnings hylsa		Coding socket	6	
044	5237 4107-081	Täckfläns ABD-8-GR		Covering flange	2	
045	2166 2308-404	Kabelkanal LKO 40/40	L=1200	Cable pit	1	
046	2166 2308-404	Kabelkanal LKO 40/40	L=475	Cable pit	1	
047	1865 2321-980	Isolerslang	L=1.5m	Tube	1	
048	2166 2058-004	Bandfäste		Mounting base	15	

Rev Ind	Revision	Appd	Year Week
C	383320	LBL	97 21

Design checked by	Accepted by qual dept	Part of	Assembly drawing
ASG	.	187 9937	114 1210

Drawing checked by	Accepted for prod by	Description (English)
ASG	ÅS	Driver desk

Dept	Drawn by	Year Week	Description (own language)
421	A Sundberg	96 35	Förarpulpet

MacGREGOR HÄGGLUNDS
MEMBER OF THE INCENTIVE GROUP

Drwg no.	Rev Ind	Sheet	No of sh
114 1210	C	2	4

This document must not be copied without our written permission, and the contents thereof must not be imparted to a third party nor be used for any unauthorized purpose. Contravention will be prosecuted.

Item no.	Article no.	Description (own language)	Dimensions	Description (English)	Qty/801	Note
049	2166 2228-172	Spännband SST-2S L=172		Strap	30	
050	2166 4127-104	Klammer RSGU 10/20		Clamp	14	
051	1856 2141-006	Gummiprofil 25x17x6	L=290	Rubber profile	1	
052	1856 2141-006	Gummiprofil 25x17x6	L=90	Rubber profile	1	
053	488 3098-001	Bricka		Washer	2	
054	488 3098-002	Bricka		Washer	2	
055	488 3099-001	Bricka		Washer	2	
056	488 3099-002	Bricka		Washer	2	
057	6179 2151-617	Mutter M20x1 msg		Nut	2	
058	6179 2151-624	Mutter M16x1 900035		Nut	2	
059	2121 2550-291	MC6S 4x12 A4-80		Allen screw	22	
061	2121 2550-366	MC6S 6x12 A4-80		Allen screw	16	
062	2121 2550-370	MC6S 6x20 A4-80		Allen screw	15	
063	2121 2550-374	MC6S 6x30 A4-80		Allen screw	2	
064	2121 2034-455	M6S 8x30 A4-80		Hexagon screw	4	
065	2121 2034-536	M6S 12x30 A4-80		Hexagon screw	3	
066	2124 2127-283	RXS Z ST 3.5x16 A4		Screw	8	
069	2126 2634-116	Låsm-M6M 6-A4-80		Locking nut	11	
070	2126 2634-118	Låsm-M6M 8-A4-80		Locking nut	10	

Rev Ind	Revision	Appd	Year Week
C	383320	LBL	97 21

Design checked by	Accepted by qual dept	Part of	Assembly drawing
ASG	.	187 9937	114 1210
Drawing checked by	Accepted for prod by	Description (English)	
ASG	ÅS	Driver desk	
Dept 421	Drawn by A Sundberg	Year Week 96 35	Description (own language) Förarpulpet

MacGREGOR HÄGGLUNDS
MEMBER OF THE INCENTIVE GROUP

Drwg no.	Rev Ind	Sheet	No of sh
114 1210	C	3	4

This document must not be copied without our written permission, and the contents thereof must not be imparted to a third party nor be used for any unauthorized purpose. Contravention will be prosecuted.

Item no.	Article no.	Description (own language)	Dimensions	Description (English)	Qty/801	Note
073	2151 2027-146	RB 5.3x10-A4		Washer	8	
074	2151 2027-156	BRB 6,7x14 -A4		Washer	34	
075	2151 2027-164	RB 8.4x16-A4		Washer	10	
076	2151 2027-178	BRB 13x24 A4		Washer	3	
077	488 7461-001	Skylt		Sign	1	
078	488 6906-001	Skylt		Sign	1	
079	2641 2034-008	Kabelsko 1.5-2.5 M6		Cable terminal	5	
090	489 3454-001	Skylt		Sign	1	

Rev Ind	Revision	Appd	Year Week
C	383320	LBL	97 21

Design checked by	Accepted by qual dept	Part of	Assembly drawing
ASG	.	187 9937	114 1210

Drawing checked by	Accepted for prod by	Description (English)
ASG	ÅS	Driver desk

Dept	Drawn by	Year Week	Description (own language)
421	A Sundberg	96 35	Förarpulpet

MacGREGOR HÄGGLUNDS
MEMBER OF THE INCENTIVE GROUP

Drwg no.	Rev Ind	Sheet	No of sh
114 1210	C	4	4

CONTROL PANEL

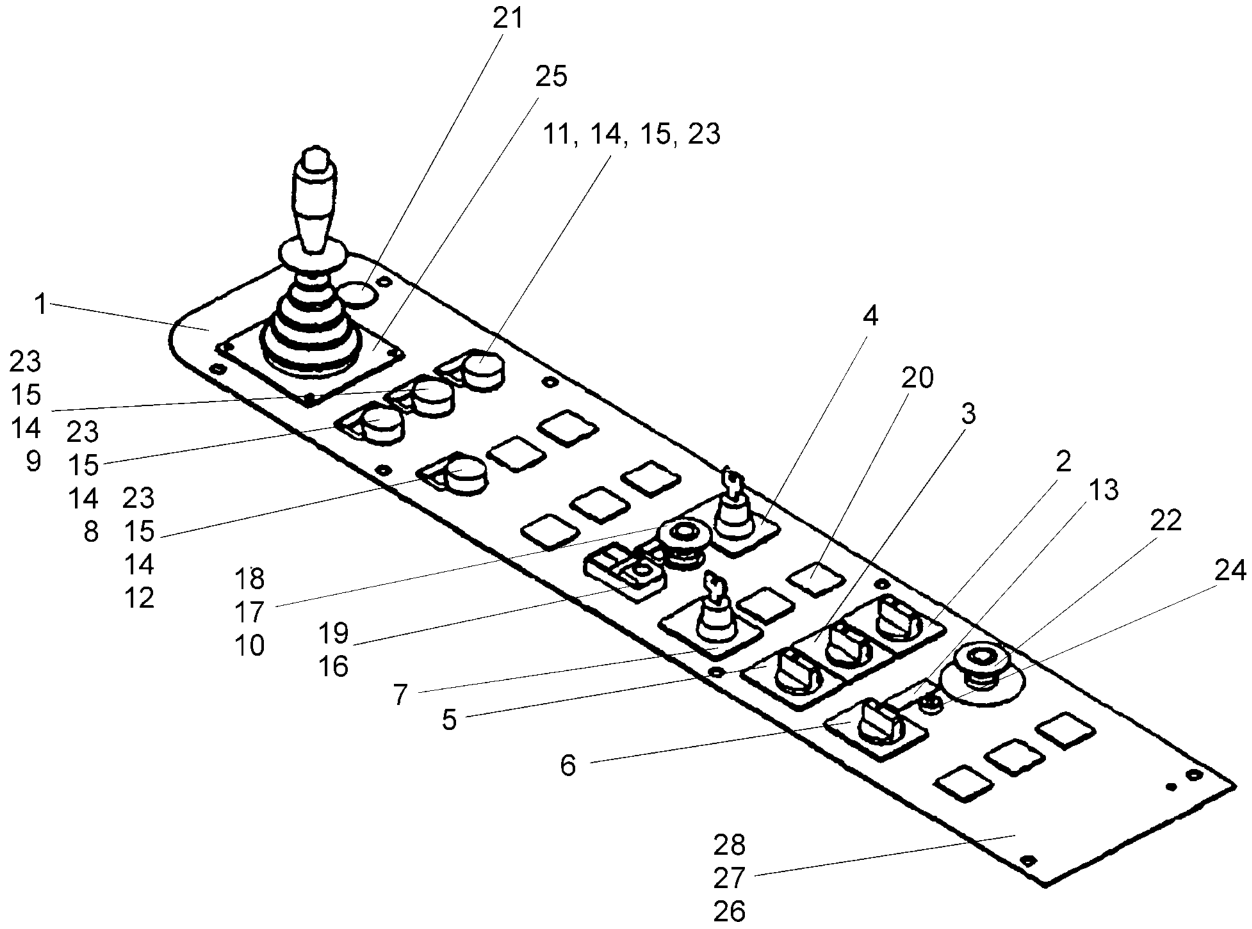

CONTROL PANEL

Item	Qty	Article no	Description	Supplementary data
000	1	214 1734-801	CONTROL PANEL	
001	1	314 3624-001	. COVER	
002	1	414 4620-801	. SWITCH	
003	1	414 4621-801	. SWITCH	
004	1	414 4622-801	. SWITCH	
005	1	414 4625-801	. SWITCH	
006	1	414 4626-801	. SWITCH	
007	1	414 4628-801	. SWITCH	
008	1	414 6954-057	. SIGN WITH FRAME	
009	1	414 6954-002	. SIGN WITH FRAME	
010	1	414 6954-046	. SIGN WITH FRAME	
011	1	414 6954-013	. SIGN WITH FRAME	
012	1	414 6954-055	. SIGN WITH FRAME	
013	1	414 1500-051	. SIGN	
014	4	5298 2057-001	. INDICATOR HEAD	
015	4	5372 2017-001	. LAMP HOLDER	
016	1	5372 2040-001	. PUSH BUTTON	
017	1	5372 2039-003	. PUSH BUTTON	
018	1	5372 2016-005	. CONTACT BLOCK	
019	1	5372 2016-006	. CONTACT BLOCK	
020	10	2152 2069-225	. COVER PLATE	
021	1	2152 2067-018	. COVER PLATE	
022	1	5372 2449-233	. EMERGENCY STOP	
023	4	5911 4509-002	. BULB	
024	1	5672 4245-002	. CIRCUIT BREAKER	
025	1	314 2005-802	. CONTROLLER, HOISTING	SEE FIG. 625-8591
026	1	2121 2550-331	. SCREW	MC6S 5 x 20 A4-80
027	2	2126 2634-114	. NUT	M6M 5 -A4 -80
028	1	2151 2027-146	. WASHER	RB 5.3 x 10 -A4

ELECTRIC PANEL

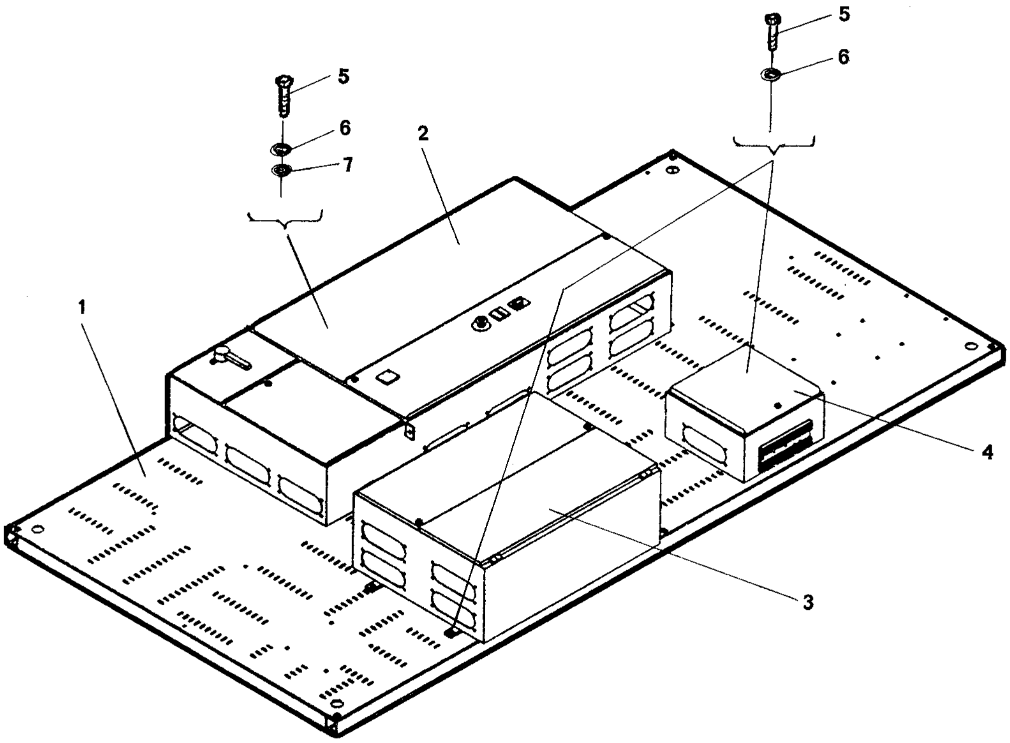

ELECTRIC PANEL

Item	Qty	Article no	Description	Supplementary data
000	1	214 1803-801	ELECTRIC PANEL	
001	1	214 1778-801	. PANEL PLATE	
002	1	214 1802-801	. CUBICLE A	
003	1	314 3500-801	. ELECTRONIC BOX MB	
004	1	314 3437-801	. POWER SUPPLY CE, C	
005	14	2121 2032-495	. SCREW	M6S 10 x 30 -8.8 G
006	14	2151 2022-173	. WASHER	BRB 10.5 x 22 G
007	6	2151 2058-173	. WASHER	TBRSB 10.5 x 28 G

TOL. SYSTEM ACC. TO ISO	General tol. for dimensions without tol. indications
Surface roughness R_a in µm	Machining ISO 2768 tol-class: Welding DIN 8570 tol-class:

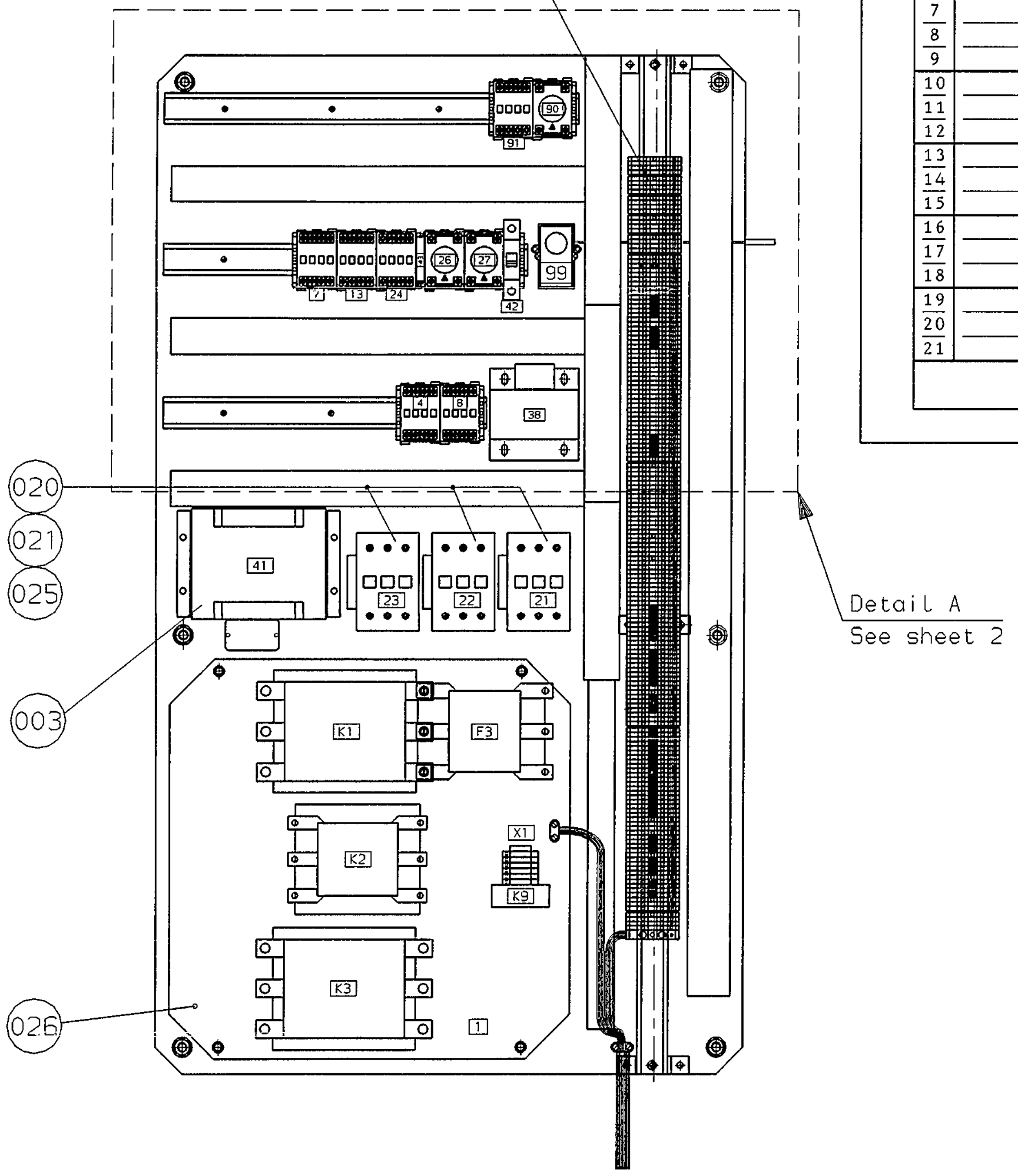

No	HC	I_{set} (A)
1	OIL COOLER MOTOR DA	3.0A
2	VENTILATION FAN DF	0.41A
3	TRANSFORMER CE	0.91A
4	TRANSFORMER A41	1.58A
5	FEED PUMP MOTOR DD	32A
6		
7		
8		
9		
10		
11		
12		
13		
14		
15		
16		
17		
18		
19		
20		
21		

MacGREGOR HÄGGLUNDS

380V/50Hz

No	HC	I_{set} (A)
1	OIL COOLER MOTOR DA	3.7A
2	VENTILATION FAN DF	0.54A
3	TRANSFORMER CE	0.8A
4	TRANSFORMER A41	1.36A
5	FEED PUMP MOTOR DD	32A
6		
7		
8		
9		
10		
11		
12		
13		
14		
15		
16		
17		
18		
19		
20		
21		

MacGREGOR HÄGGLUNDS

440V/60Hz

Skala / Scale 1:10

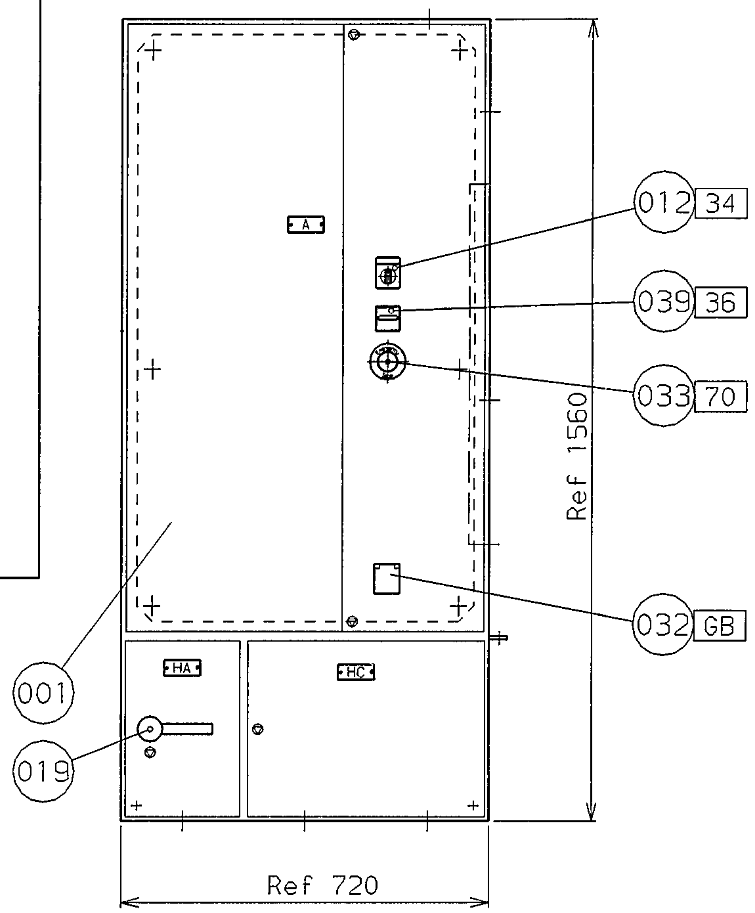

Kopparskenan kapas av
Copperbar cuts of

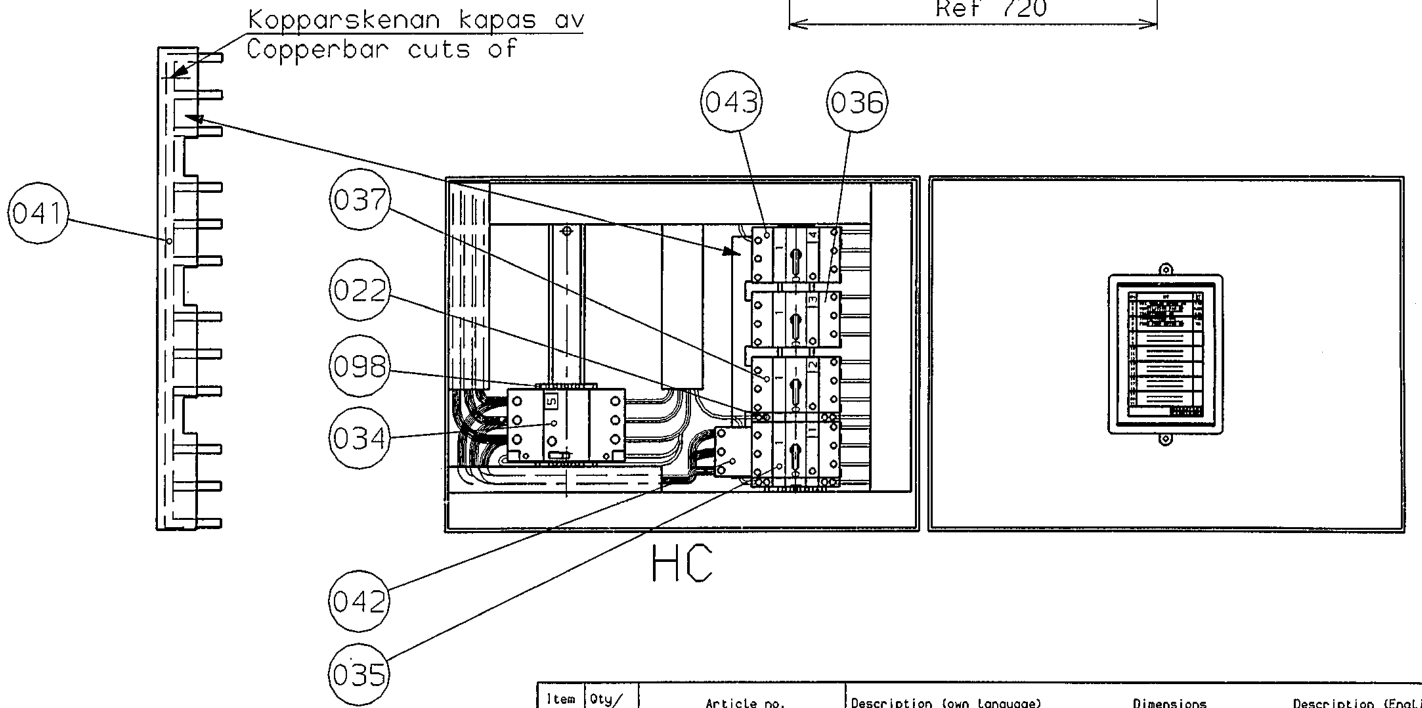

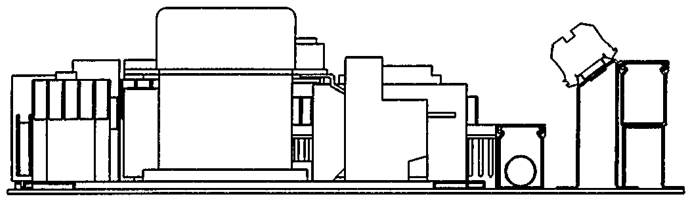

This document must not be copied without our written permission, and the contents thereof must not be imparted to a third party nor be used for any unauthorized purpose. Contravention will be prosecuted.

Reference KR96115

Similar drawing No. 214 1710

Item no.	Qty/801	Article no.	Description (own language)	Dimensions	Description (English)

Design checked by: ASG	Accepted by qual dept	Part of	
Drawing checked by: ASG	Accepted for prod by: MN	Specification: 214 1802	Scale: 1:5
Dept: 421	Drawn by: A Sundberg/LB — Year Week: 97 13	Description (English): A cubicle switch board	Weight kg
		Description (own language): Apparatskåp A	Prod.group: 628
MacGREGOR HÄGGLUNDS — MEMBER OF THE INCENTIVE GROUP		Drwg no.: 214 1802	Rev ind / Sheet: 1 / No of sh: 2

Revind	Revision	Appd	Year	Week

TOL. SYSTEM ACC. TO ISO	General tol. for dimensions without tol. indications
Surface roughness R_a in µm	Machining ISO 2768 tol-class:
	Welding DIN 8570 tol-class:

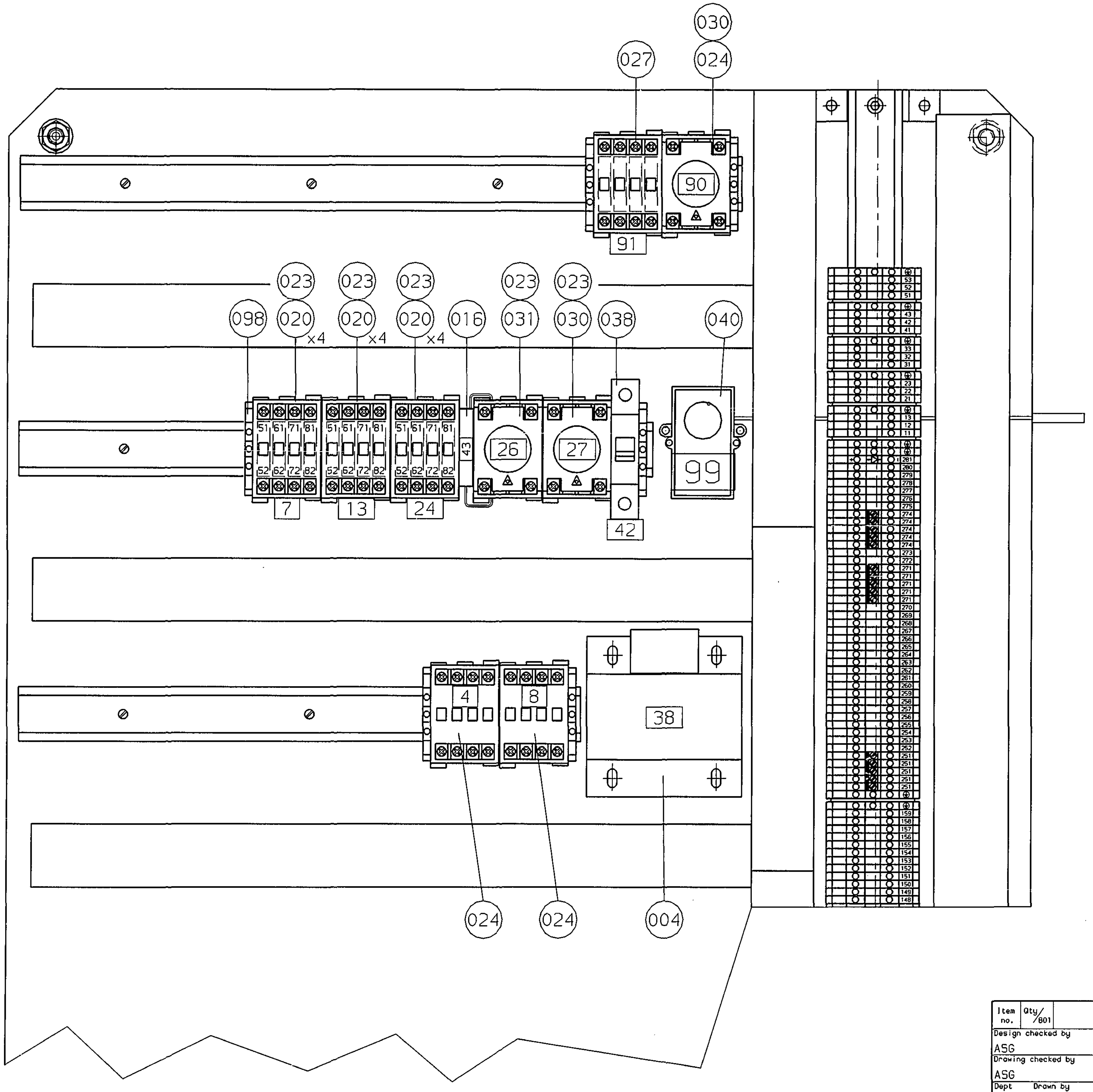

Detail A

This document must not be copied without our written permission, and the contents thereof must not be imparted to a third party nor be used for any unauthorized purpose. Contravention will be prosecuted.

Reference KR96115

Similar drawing No.

Item no.	Qty/801	Article no.	Description (own language)	Dimensions	Description (English)

Design checked by: ASG	Accepted by qual dept	Part of
Drawing checked by: ASG	Accepted for prod by: MN	Specification: 214 1802
Dept: 421	Drawn by: A Sunberg/LBL	Year Week: 97 :3
Description (English): A cubicle switch board		Scale: 1:2
Description (own language): Apparatskåp A		Weight kg
		Prod.group: 628
Drwg no.: 214 1802	Rev ind	Sheet: 2
		No of sh: 2

MacGREGOR HÄGGLUNDS
MEMBER OF THE INCENTIVE GROUP

Rev ind	Revision	Appd	Year	Week

This document must not be copied without our written permission, and the contents thereof must not be imparted to a third party nor be used for any unauthorized purpose. Contravention will be prosecuted.

Item no.	Article no.	Description (own language)	Dimensions	Description (English)	Qty/801	Note
000	414 6714-000	Förb. tabell A-skåp		Wiring table A cubicle	X	
001	214 1799-801	Apparatskåp A grund		Cubicle A basic	1	
003	314 3675-801	Transformator 1-fas		Transformer 1 phase	1	
004	314 3452-801	Transformator 1-fas		Transformer 1 phase	1	
005	314 3434-801	Plint		Plinth	1	
012	414 4627-801	Omkopplare		Switch	1	
016	414 1941-801	Termistor		Thermistor	1	
019	5381 4226-001	Lastbrytare		Main switch	X	
020	5354 4136-701	Hjälpkontakt		Auxilliary contact	15	
021	5354 4136-710	Hjälpkontakt		Auxilliary contact	3	
022	5218 3110-011	Hjälpkontakt		Auxilliary contact	2	
023	5354 4131-400	Reläkonta.		Relay contactor	5	
024	5354 4272-090	Kontaktor		Contactor	3	
025	5354 4272-406	Kontaktor		Contactor	3	
026	5591 2148-150	Y/D		Star Delta Start	1	
027	5354 4132-401	Reläkontaktor		Relay contaktor	1	
030	5354 4134-007	Tiddon		Time delayer	2	
031	5354 4134-008	Tiddon		Time delayer	1	
032	5381 4280-001	El-uttag		Socket	1	

Design checked by	Accepted by qual dept	Part of	Assembly drawing
ASG	.		214 1802

Drawing checked by	Accepted for prod by	Description (English)
ASG	MN	A cubicle switch board

Dept	Drawn by	Year Week	Description (own language)
421	A Sundberg/LB	97 13	Apparatskåp A

Rev Ind	Revision	Appd	Year Week

MacGREGOR HÄGGLUNDS
MEMBER OF THE INCENTIVE GROUP

Drwg no.	Rev Ind	Sheet	No of sh
214 1802		1	2

This document must not be copied without our written permission, and the contents thereof must not be imparted to a third party nor be used for any unauthorized purpose. Contravention will be prosecuted.

Item no.	Article no.	Description (own language)	Dimensions	Description (English)	Qty/801	Note
033	5372 2449-233	Nödstopp		Emergency stop	1	
034	5572 4101-040	Motorsk.brytare		Motor switch	1	
035	5572 4100-400	Motorsk-brytare		Motor switch	1	
036	5572 4100-100	Motorsk-brytare		Motor switch	1	
037	5572 4100-063	Motors-brytare		Motor switch	1	
038	5672 4248-006	Automatsäkring		Automatic circuit breaker	1	
039	5692 4740-024	Timräknare		Hour meter	1	
040	7635 4113-200	Termostat		Thermostat	1	
041	2611 3110-001	Fasskena 4 parallella med hj.ko.		Collector rail	1	
042	2663 4660-002	Anslutningsplint		Conn. plinth	1	
043	5572 4100-160	Motorsk-brytare		Motor switch	1	
098	2669 4160-015	Ändstöd		End holder	9	

Design checked by	Accepted by qual dept	Part of	Assembly drawing
ASG	.		214 1802
Drawing checked by	Accepted for prod by	Description (English)	
ASG	MN	A cubicle switch board	

Dept	Drawn by	Year	Week	Description (own language)
421	A Sundberg/LB	97	13	Apparatskåp A

Rev Ind	Revision	Appd	Year	Week

MacGREGOR HÄGGLUNDS
MEMBER OF THE INCENTIVE GROUP

Drwg no.	Rev Ind	Sheet	No of sh
214 1802		2	2

ELECTRONIC BOX MB

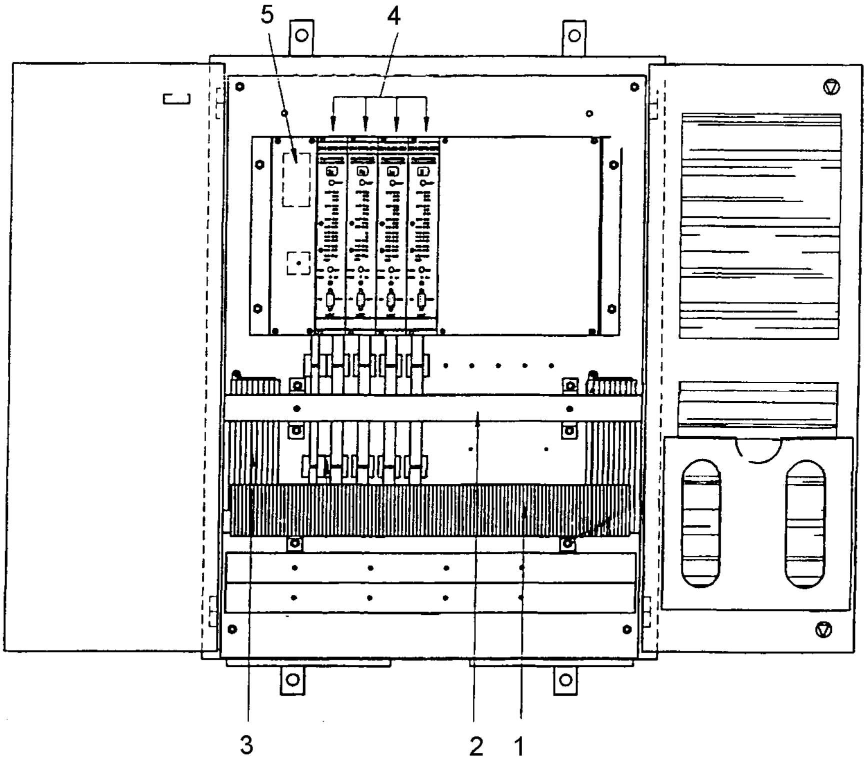

314 3500-801
625-8607.001

ELECTRONIC BOX MB

Item	Qty	Article no	Description	Supplementary data
000	1	314 3500-801	ELECTRONIC BOX MB	
001	1	314 3609-801	. PLINTH, LOWER	
002	1	314 3687-801	. PLINTH, UPPER	
003	2	414 5351-801	. HEATER	
004	4	314 3603-902	. MPC-CARD	
005	1	314 3605-901	. MEMORY CARD	

EL. INST. JIB LIGHT

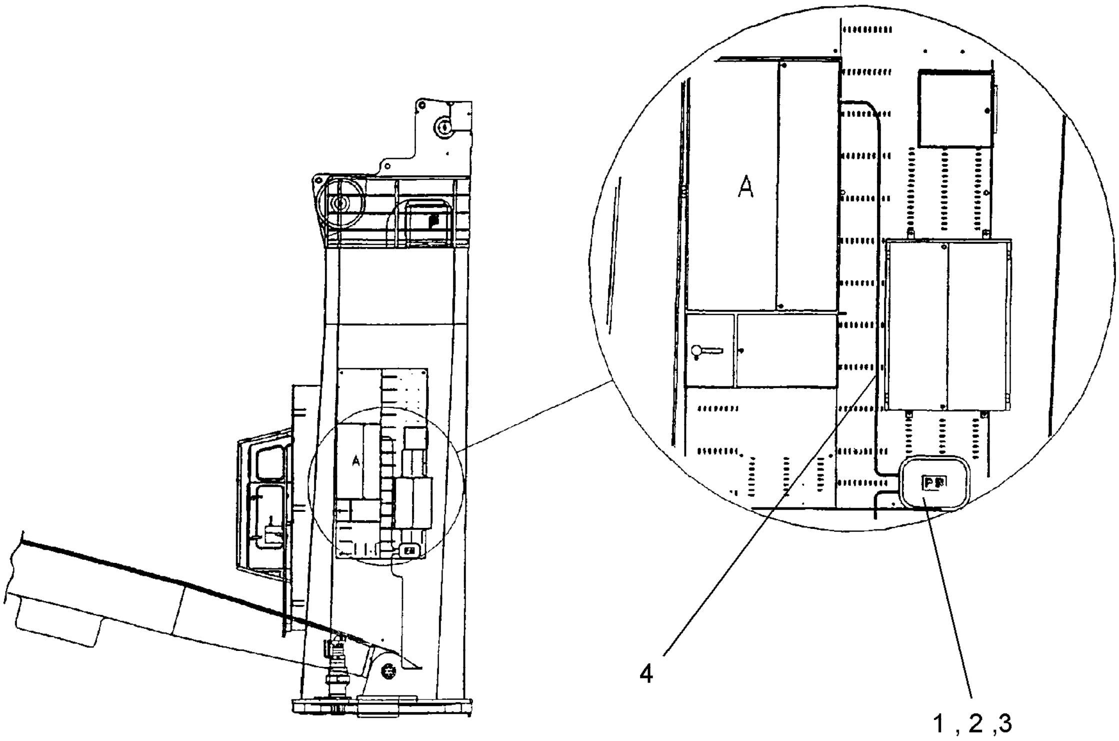

EL. INST. JIB LIGHT

Item	Qty	Article no	Description	Supplementary data
000	1	114 1314-801	EL. INST. JIB LIGHT	
001	1	5921 2104-901	. BALLAST UNIT	
002	4	2121 2032-495	. SCREW	M6S 10 x 30 -8.8 FZB
003	4	2151 2022-173	. WASHER	BRB 10.5 x 22 FZB
004	1	1684 2136-123	. CABLE	L = 4 m

EL. INST. JIB LIGHT

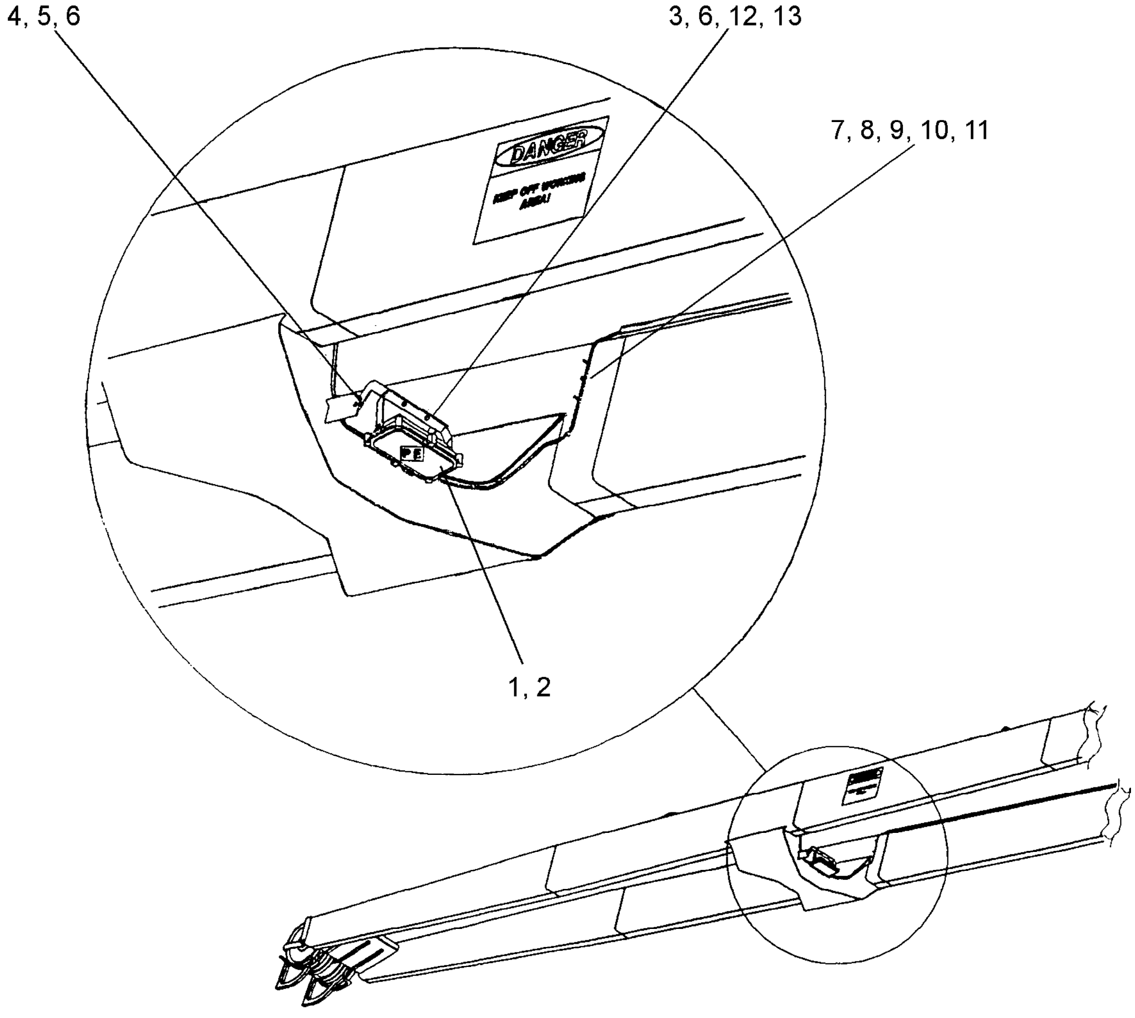

114 1315-801
625-8567.001

EL. INST. JIB LIGHT

Item	Qty	Article no	Description	Supplementary data
000	1	114 1315-801	EL. INST. JIB LIGHT	
001	1	5921 2104-101	. FLOOD LIGHT	
002	1	5912 4128-400	. SODIUM LAMP	
003	1	387 8717-002	. BRACKET	
004	2	487 2011-003	. SCREW	
005	2	487 2012-001	. WASHER	
006	4	2126 2634-122	. NUT	M6M 12 -A4 -80
007	1	1686 2136-134	. CABLE	L = 40 m
008	18	2166 4127-154	. CLAMP	
009	18	2126 2032-118	. NUT	M6M 8 -8 FZB
010	18	2126 2634-118	. NUT	M6M 8 -A4-80
011	18	2151 2027-164	. WASHER	RB 8.4 x 16 -A4
012	4	2121 2034-540	. SCREW	M6S 12 x 40 A4-80
013	4	2151 2025-178	. WASHER	BRB 13 x 24 RF

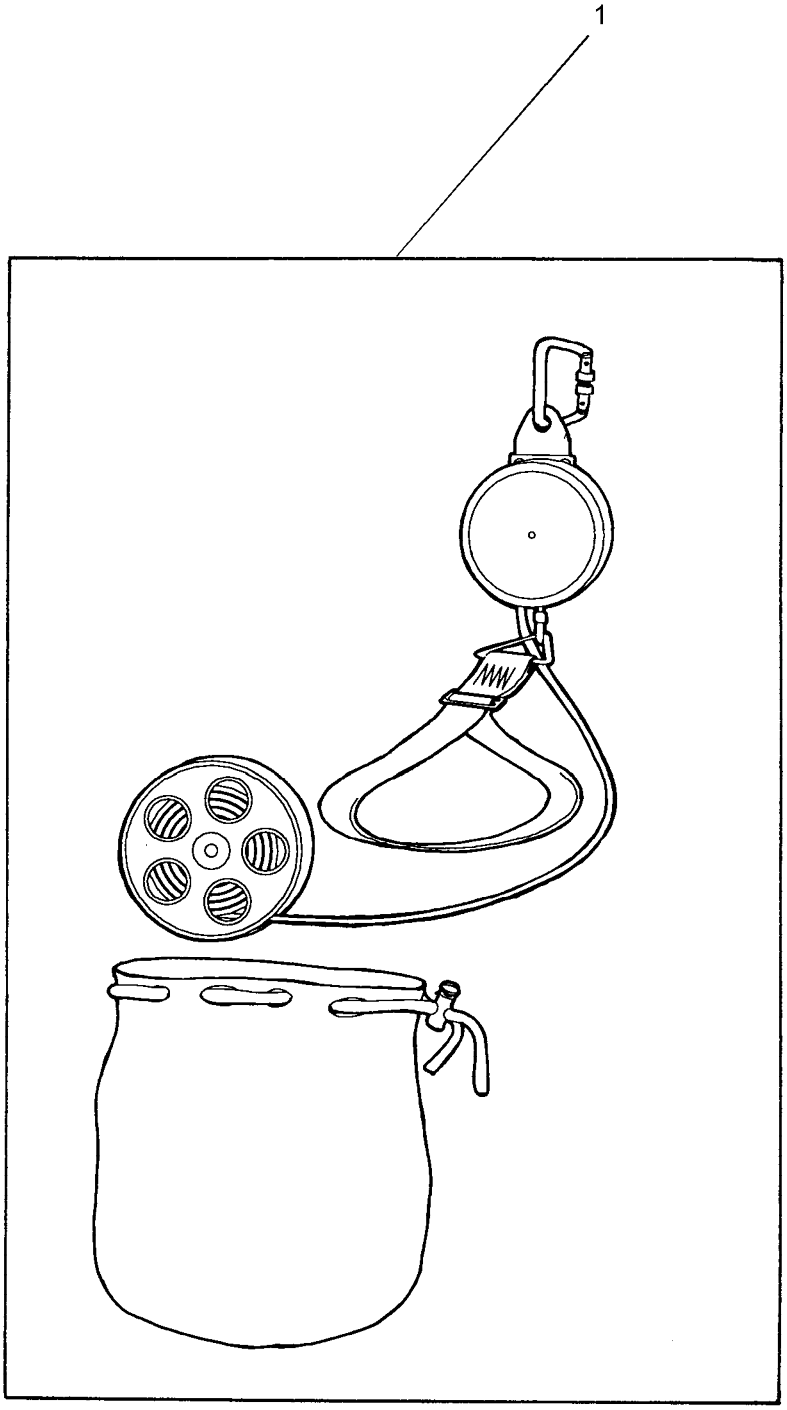
1

RESCUE EQUIPMENT

Item	Qty	Article no	Description	Supplementary data
001	1	388 9561-801	RESCUE EQUIPMENT	

ANTI-DAZZLING SCREEN MOUNT

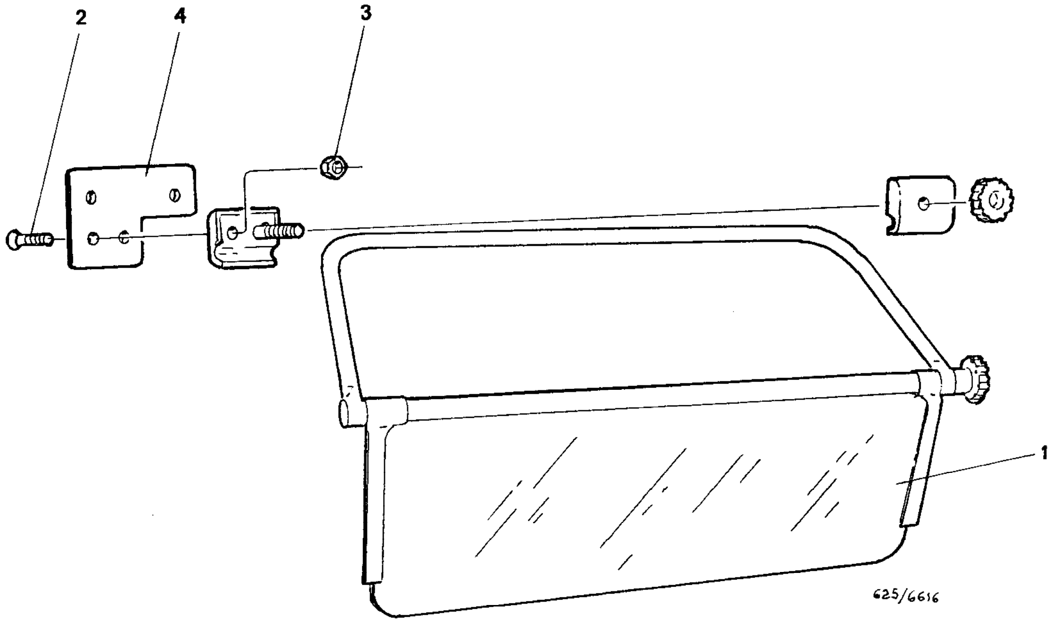

ANTI-DAZZLING SCREEN MOUNT

Item	Qty	Article no	Description	Supplementary data
000	1	287 7010-801	ANTI-DAZZLING SCREEN MOUNT	
001	1	6172 2111-001	. SUN VISOR	
002	2	2121 2634-370	. SCREW	MFS 6 x 20 -5.8 G
003	2	2126 2032-116	. NUT	M6M 6 -8 G
004	1	489 5610-001	. MOUNTING PLATE	

FIRE EXTINGUISHER, MOUNT.

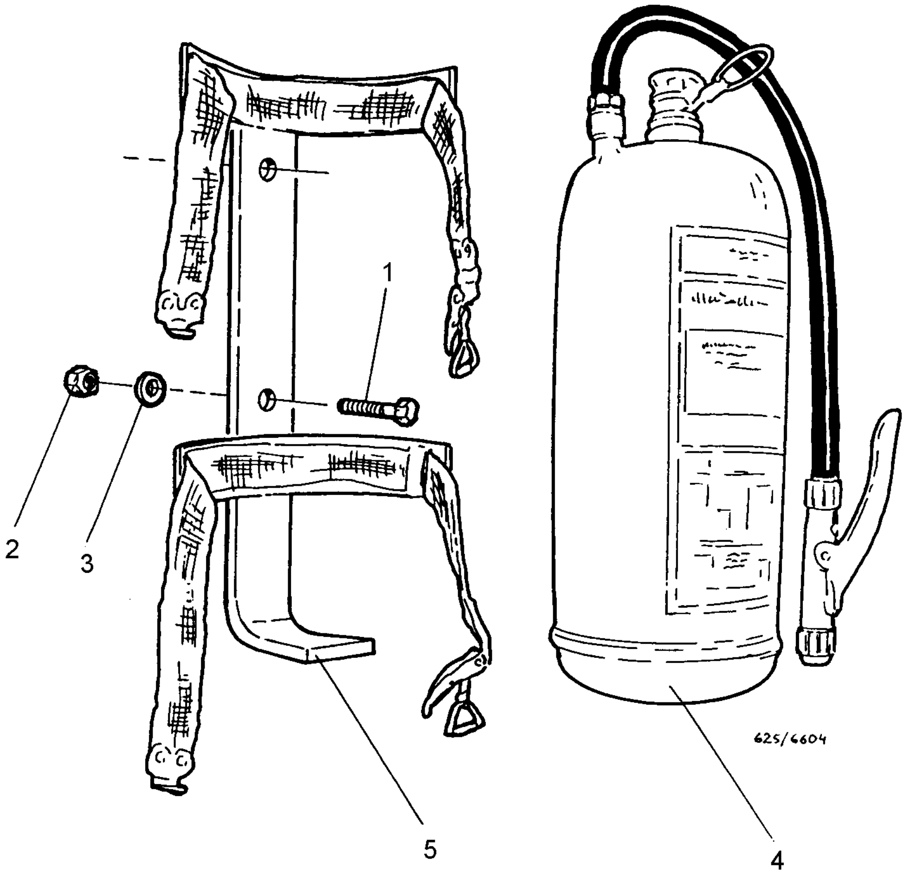

488 8556-801
624-2520

FIRE EXTINGUISHER, MOUNT.

Item	Qty	Article no	Description	Supplementary data
000	1	488 8556-801	FIRE EXTINGUISHER, MOUNT.	
001	2	2121 2532-451	. SCREW	MC6S 8 x 20 -8.8 FZB; DIN 912
002	2	2126 2636-118	. NUT	NYLOC -M6M 8 -8 FZB
003	2	2151 2022-164	. WASHER	BRB 8.4 x 16 FZB; DIN 125A
004	1	6956 2037-060	. FIRE EXTINGUISHER POWDER	
005	1		.. MOUNTING BRACKET	

TOL. SYSTEM ACC. TO ISO	General tol. for dimensions without tol. indications
Surface roughness R_a in µm	Machining ISO 2768 tol-class:
	Welding DIN 8570 tol-class:

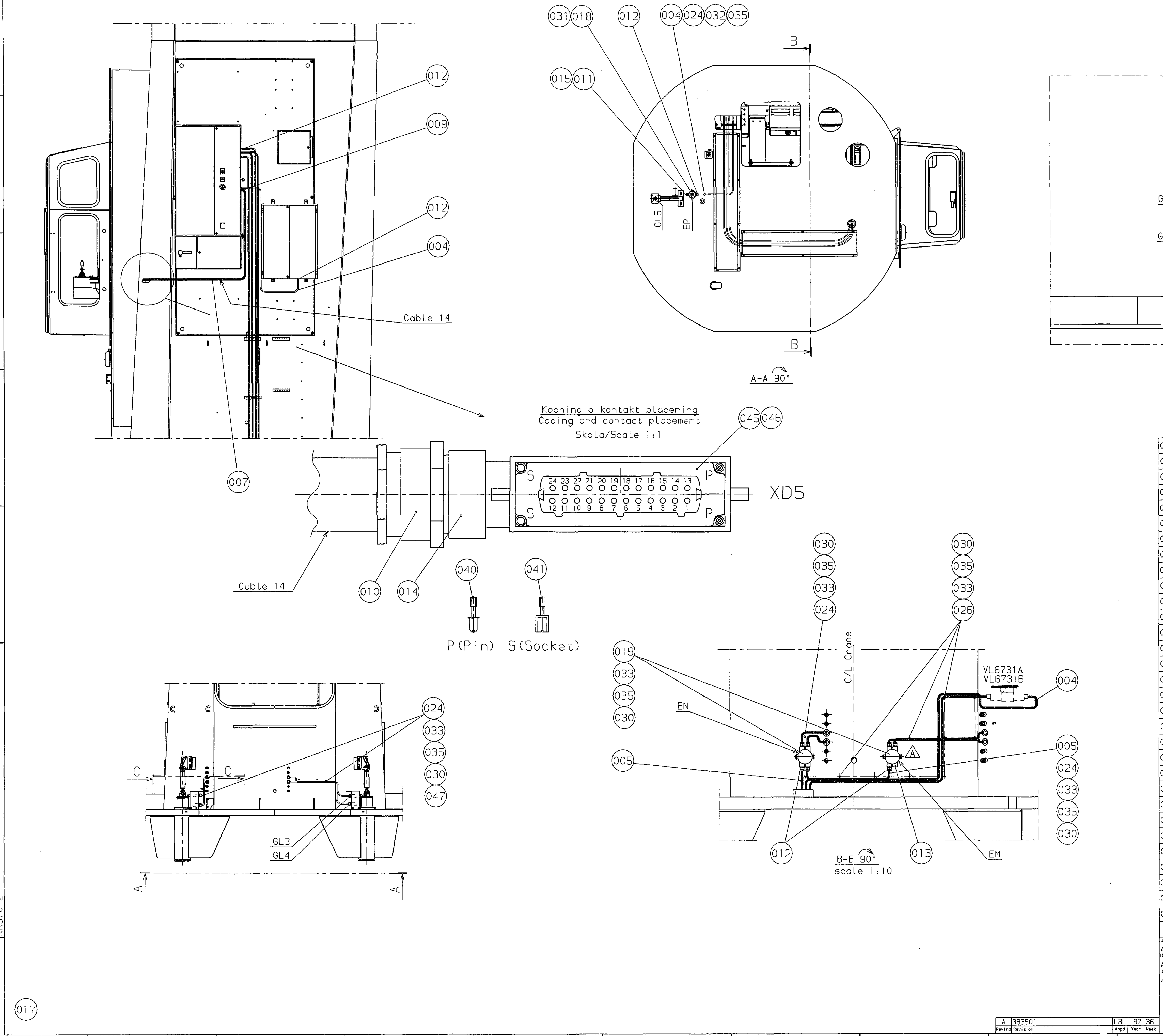

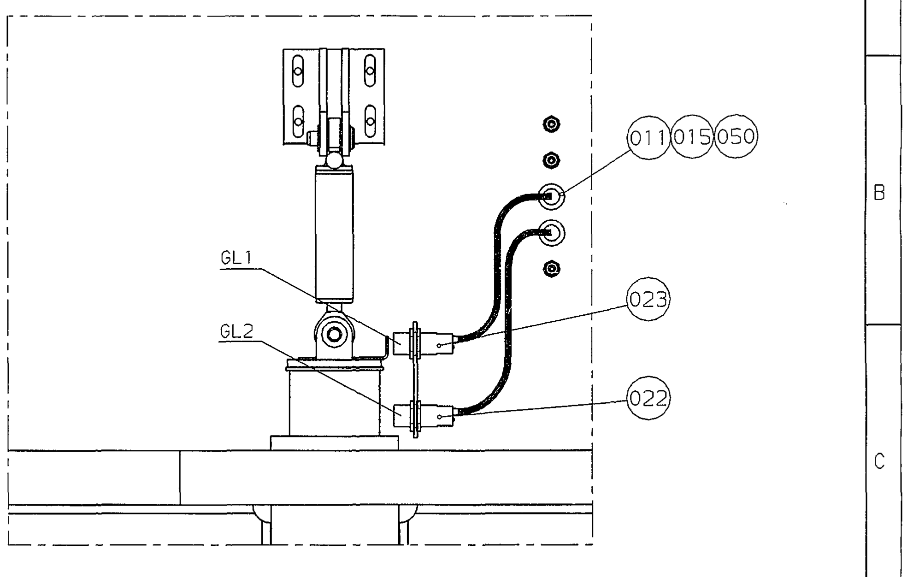

C-C scale 1:5

Item no.	Qty/801	Article no.	Description (own language)	Dimensions	Description (English)
050	x	004 8019-001	Tätningsband		Sealing compound
047	1	1865 2321-910	PVC-slang	L=6m	Insulating PVC sle.
046	1	5217 4834-240	Kontaktdon plint		Screw connection
045	1	5217 4833-240	Kabelkåpa		Plug hood
041	2	414 6790-001	Kodnings hylsa		Coding socket
040	2	414 6785-001	Kodnings pinne		Coding pin
035	26	2151 2022-164	BRB 8,4x16 fzb		Washer
033	26	2126 2032-118	M6M 8 -8 FZB		NUT
032	1	2121 2032-451	M6S 8x20 -8.8 Fzb		SCREW
031	2	2121 2032-370	M6S 6x20 -8.8 FZB		SCREW
030	26	2126 2636-118	Låsm-M6M 8-8 fzb		Locking nut
026	10	2166 4127-194	Klammer RSGU 19/20		Clamp
024	12	2166 4127-114	Klammer RSGU 11/20		Clamp
023	2	5661 4214-011	Givare induktiv		Impuls unit
022	2	5661 4214-010	Givare induktiv		Impuls unit
019	2	5388 4121-010	Kopplingsdosa		Junction box
018	1	5388 4121-002	Kopplingsdosa		Connection box
017	20	2949 4521-999	Märkhylsa PK 2/4 gul		Marking cage
015	9	2686 4425-225	Reduc-muff Pr 22,5-18,6		Reducing nipple
014	1	2686 4426-370	Först-muff Pr 37,0-47,0		Enlarging nipple
013	1	2152 2123-225	Avslutningspropp Pr 22,5		Concluding Plug
012	10	2166 2051-821	Kabelförskr. Pr 22,5		Cable gland
011	9	2166 2051-811	Kabelförskr. Pr 18,6		Cable gland
010	1	2166 4115-047	Kabelförskruvning Pr47		Cable gland
009	1	2166 2051-852	Kabelförskr. Pr 47,0		Cable gland
007	1	1684 2145-030	Elkabel H07RND3-F 30G1 AT L=6m		Cable
005	1	1684 2136-124	Elkabel RU 4x1,5	L=16m	Cable
004	1	1684 2136-123	Elkabel RU 3x1,5	L=39m	Cable
003	x	414 6982-000	Förb. tab. park.låsn		Winging table park.lock
002	x	314 3788-000	Kretssch. park.lås		Circuit diagram park. lock
001	x	314 3787-000	Blocksch. park.lås		Block diagram park. lock

Design checked by: ASG	Accepted by qual dept	Part of	
Drawing checked by: ASG	Accepted for prod by: MN	Specification	Scale 1:20
Dept 421, Drawn by A Sundberg/LB, Year Week 97 15		Description (English): El inst. crane p	Weight kg
		Description (own language): El inst. kran park. låsni	Prod.group
MacGREGOR HÄGGLUNDS — MEMBER OF THE INCENTIVE GROUP		Drwg no. 114 1286	Rev ind A; Sheet 1; No of sh 1

Rev ind	Revision	Appd	Year Week
A	383501	LBL	97 36

Reference: KR97012

Similar drawing No.

This document must not be copied without our written permission, and the contents thereof must not be imparted to a third party nor be used for any unauthorized purpose. Contravention will be prosecuted.

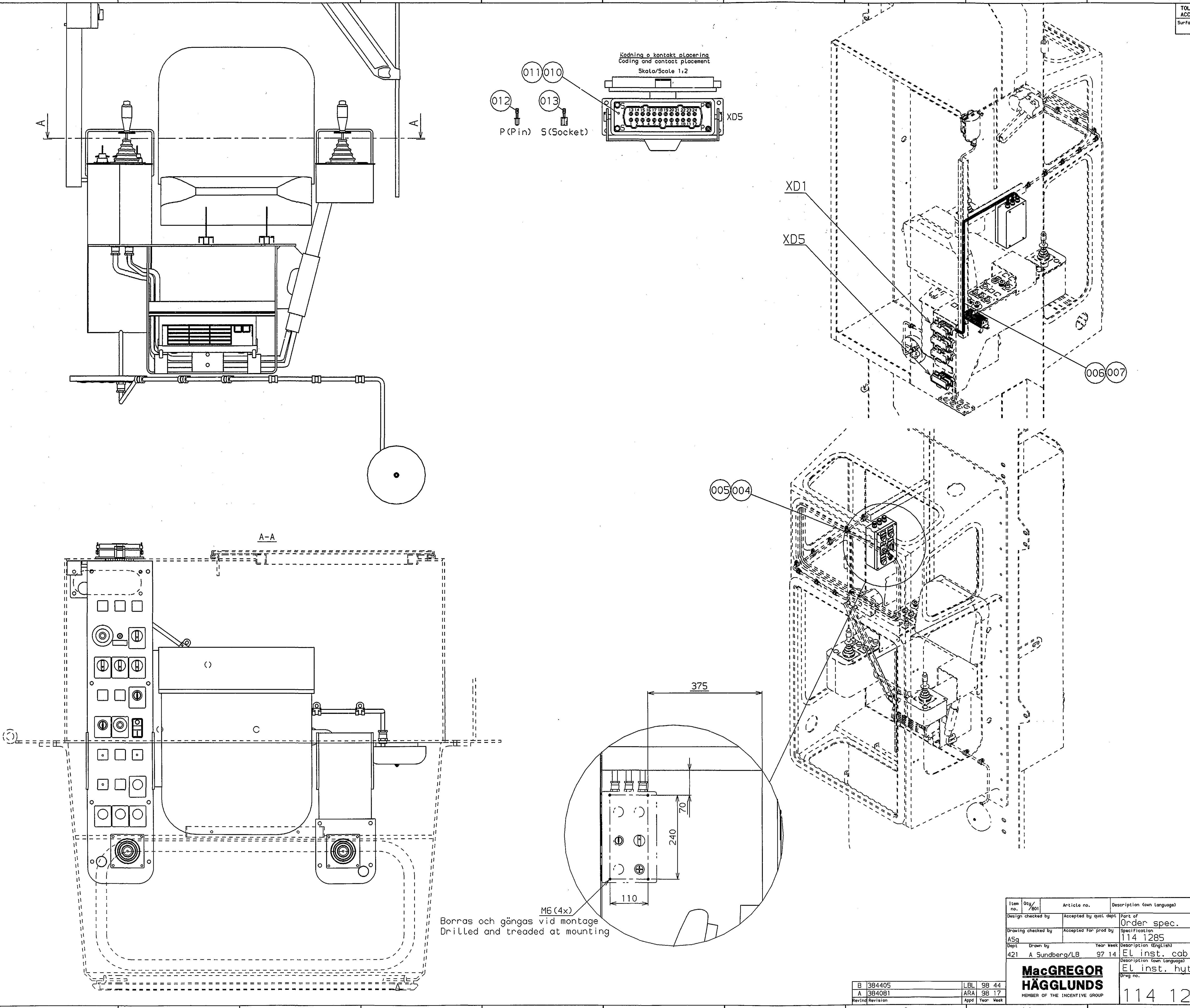

TOL. SYSTEM ACC. TO ISO	General tol. for dimensions without tol. indications
Surface roughness R_a in µm	Machining ISO 2768 tol-class:
	Welding DIN 8570 tol-class:

Item no.	Qty/801	Article no.	Description (own language)	Dimensions	Description (English)

Design checked by	Accepted by qual dept	Part of: Order spec.	
Drawing checked by: ASg	Accepted for prod by	Specification: 114 1285	Scale: 1:5
Dept: 421	Drawn by: A Sundberg/LB, Year Week: 97 14	Description (English): El inst. cabin p	Weight kg
MacGREGOR HÄGGLUNDS MEMBER OF THE INCENTIVE GROUP		Description (own language): El inst. hytt park. låsni	Prod.group: 628
		Drwg no.: 114 1285	Rev ind: B; Sheet: 1; No of sh: 1

Rev Ind	Revision	Appd	Year	Week
B	384405	LBL	98	44
A	384081	ARA	98	17

This document must not be copied without our written permission, and the contents thereof must not be imparted to a third party nor be used for any unauthorized purpose. Contravention will be prosecuted.

Reference: KR97012

Similar drawing No.: 114 1219

This document must not be copied without our written permission, and the contents thereof must not be imparted to a third party nor be used for any unauthorized purpose. Contravention will be prosecuted.

Item No.	Article No.	Description (own language)	Dimensions	Description (English)	Qty/801	Note
003	414 7241-000	Förb.tabell park.låsning		Wiring table	x	
004	314 3840-801	Manöverlåda		Operating switch box	1	
005	1685 2104-120	Elkabel RK 1,5 svart	L=2m	Cable	1	
006	1684 2136-127	Elkabel RU 7x1,5	L=5m	Cable	1	
007	1684 2136-123	Elkabel RU 3x1,5	L=2.5m	Cable	1	
010	5217 4835-240	Kontaktdon plint		Screw connection	1	
011	5217 4836-240	Kontaktdonskåpa		Bulkhead housing	1	
012	414 6785-001	Kodnings pinne		Coding pin	2	
013	414 6790-001	Kodnings hylsa		Coding socket	2	
015	2166 2228-172	Spännband SST-25 L=172		Strap	10	

Rev Ind	Revision	Appd	Year Week
B	384405	LBL	98 44

Spec checked by: ASg

Drawn by: B Andersson/LB — Year Week: 97 14

MacGREGOR HÄGGLUNDS

Assembly drawing: 114 1285

Description (English): El inst. cabin p

Description (own language): El inst. hytt park. låsni

Spec No.: 114 1285

Rev Ind: B

Sheet: 1

No of sh: 1

TOL. SYSTEM ACC. TO ISO	General tol. for dimensions without tol. indications
Surface roughness R_a in µm	Machining ISO 2768 tol-class:
	Welding DIN 8570 tol-class:

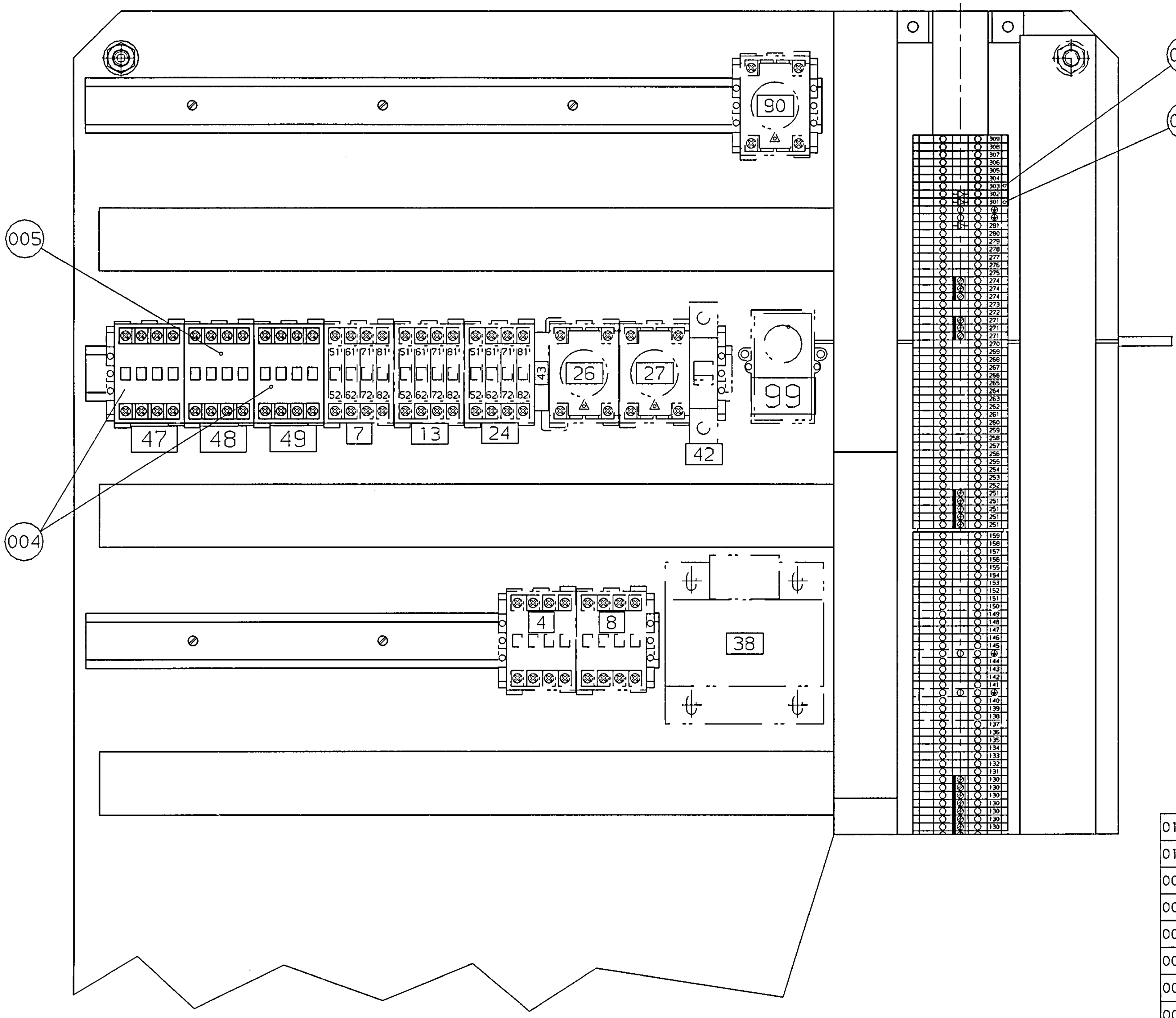

Item no.	Qty/801	Article no.	Description (own language)	Dimensions	Description (English)
011	9	2940 4108-999	Märkning WS 10/5		Marking
010	1	1685 2104-120	Elkabel RK 1,5 svart	L=10m	Cable
007	2	2669 4190-025	Diodplint		Diode terminal
006	7	2663 4382-025	Kopplingsplint		Terminal block
005	1	5354 4131-400	Reläkonta.		Relay contactor
004	2	5354 4131-220	Reläkonta.		Relay contactor
003	x	414 7242-000	Förb.tab. p.låsning		Wiring table park.lock
002	x	314 3788-000	Kretssch. p.låsn.		Circuit diagram p. lock
001	x	314 3787-000	Blockschema p.låsn.		Block diagram p. lock

Design checked by	Accepted by qual dept	Part of	
ASg			
Drawing checked by	Accepted for prod by	Specification	Scale
ASg	MN		1:2
Dept / Drawn by / Year Week		Description (English)	Weight kg
421 A Sundberg/LB 97 15		El. inst. A-cub.	
		Description (own language)	Prod.group
**MacGREGOR HÄGGLUNDS** MEMBER OF THE INCENTIVE GROUP		El inst. A-skåp park.låsn	
		Drwg no. 214 1797	Rev ind A / Sheet 1 / No of sh 1

Rev ind	Revision	Appd	Year	Week
A	384081	ARA	98	17

Reference KR97012

Similar drawing No.

This document must not be copied without our written permission, and the contents thereof must not be imparted to a third party nor be used for any unauthorized purpose. Contravention will be prosecuted.

TOL. SYSTEM ACC. TO ISO	General tol. for dimensions without tol. indications
Surface roughness R_a in µm	Machining ISO 2768 tol-class:
	Welding DIN 8570 tol-class:

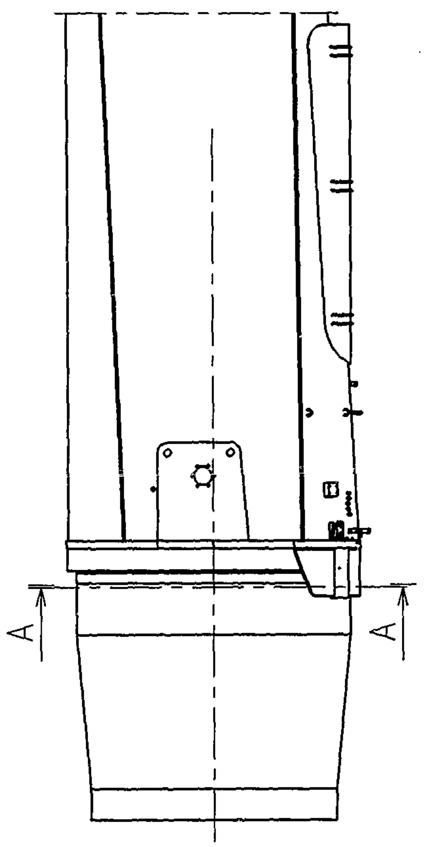

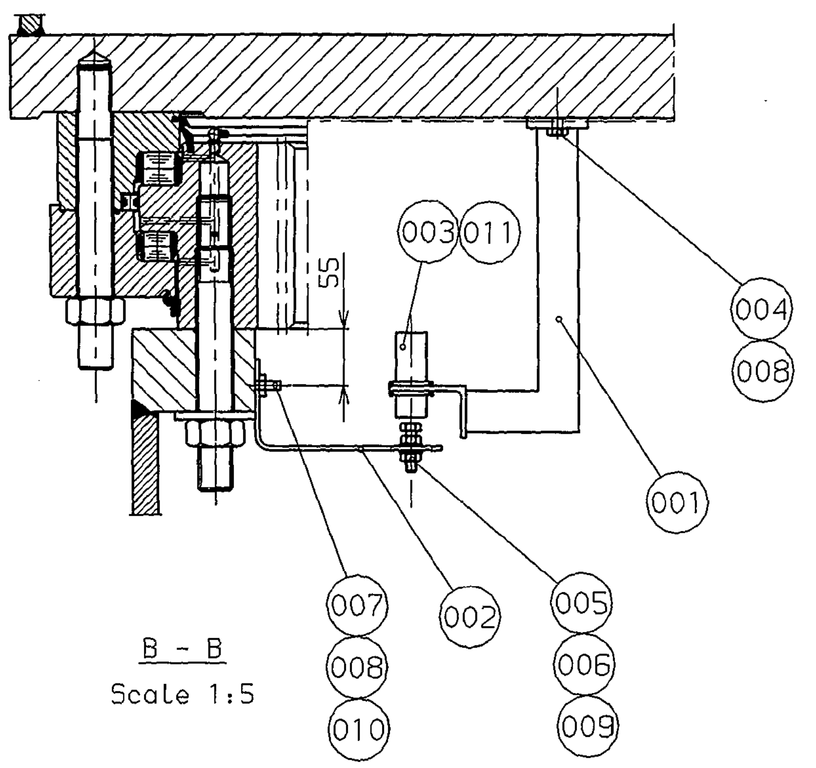

B - B
Scale 1:5

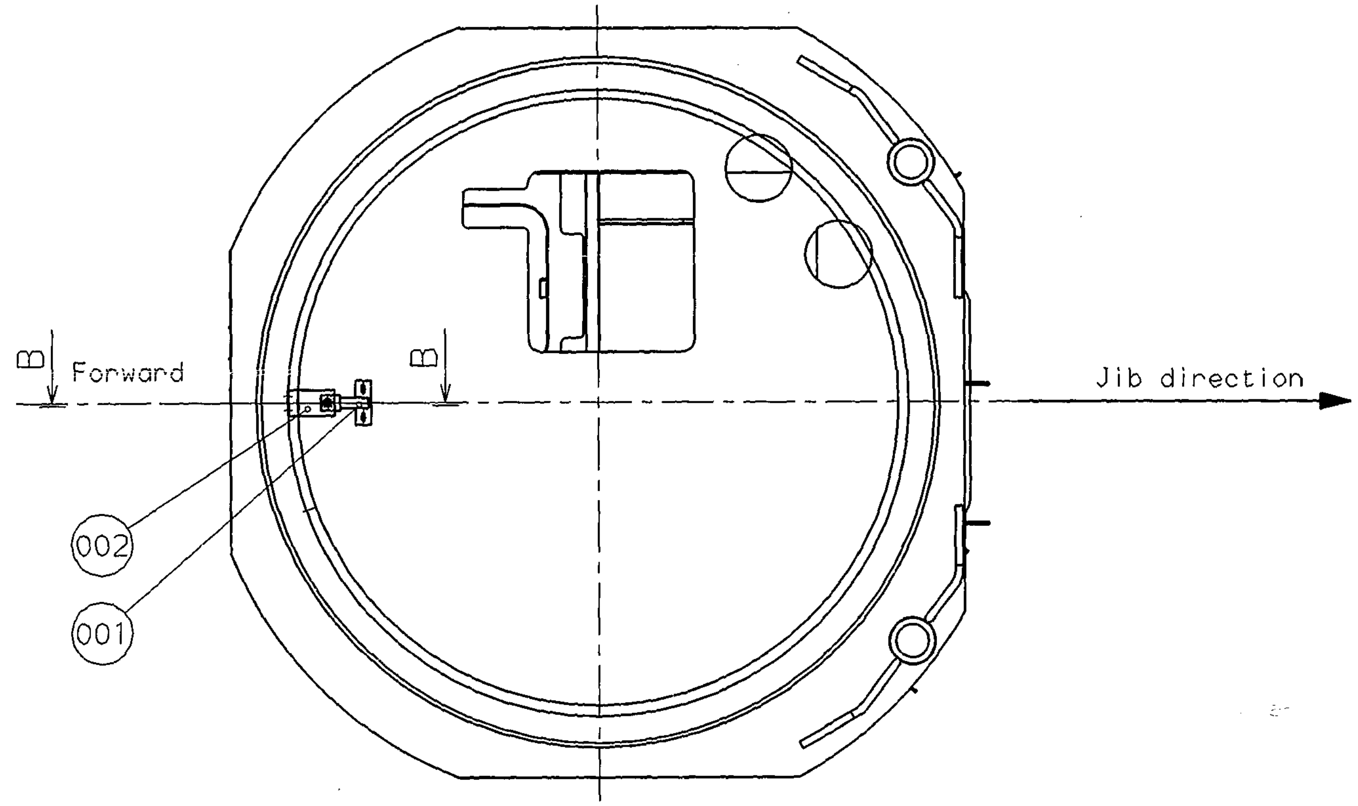

A - A
Scale 1:20

Item no.	Qty/BOI	Article no.	Description (own language)	Dimensions	Description (English)
011	6	2166 2228-280	Spännband SST		Strap
010	2	2129 2901-453	SVETSBULT M8x25		WELDING BOLT
009	2	2151 2022-173	BRB 10,5x22 fzb		Washer
008	4	2151 2022-164	BRB 8,4x16 fzb		Washer
007	2	2126 2636-118	Låsm-M6M 8-8 fzb		Locking nut
006	2	2126 2032-120	M6M 10 -8 fzb		Nut
005	1	2121 2032-499	M6S 10x40 -8.8 FZB		SCREW
004	2	2121 2032-453	M6S 8x25 -8.8 fzb		Screw
003	1	5661 4214-010	Givare induktiv		Impuls unit
002	1	389 1366-001	Fäste		Bracket
001	1	389 2322-801	Fäste		Bracket

Design checked by	Accepted by qual dept	Part of: Order spec	
Drawing checked by: SON	Accepted for prod by: DA	Specification	Scale: 1:50
Dept: 413	Drawn by: A Sedin, Year Week: 97 29	Description (English): Mounting impuls unit	Weight kg: 3.5
		Description (own language): Montering givare	Prod.group: 628
MacGREGOR HÄGGLUNDS, MEMBER OF THE INCENTIVE GROUP		Drwg no.: 288 1845	Rev ind; Sheet: 1; No of sh: 1

Revind	Revision	Appd	Year	Week

288 1390
A0 KR970178

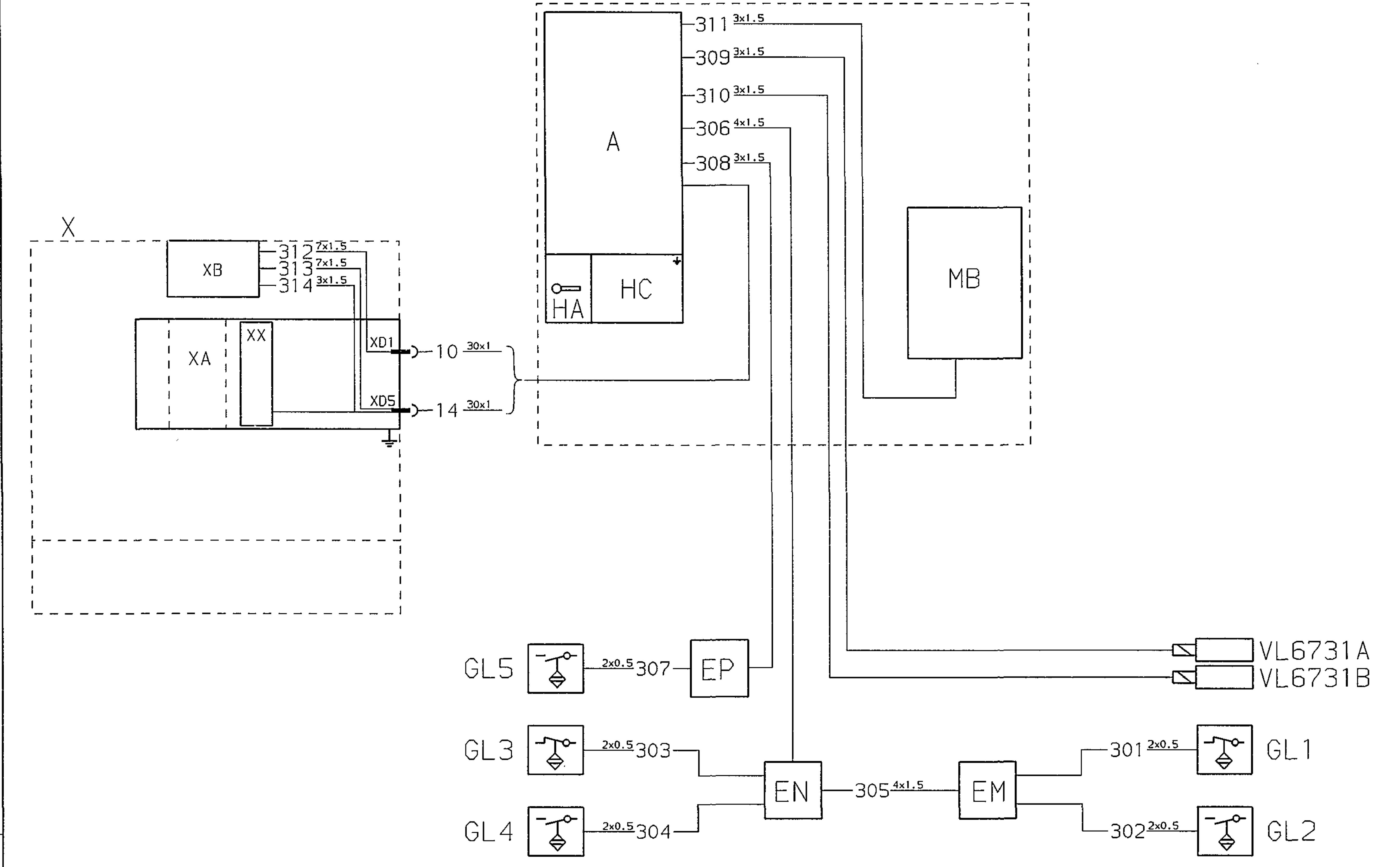

This document must not be copied without our written permission, and the contents thereof must not be imparted to a third party nor be used for any unauthorized purpose. Contravention will be prosecuted.

Reference: KR97012

Similar drawing no.: 314 3765

Rev Ind	Revision	Appd	Year	Week

MacGREGOR HÄGGLUNDS
MEMBER OF THE INCENTIVE GROUP

Description (own language)	Description (English)	Prod.group
Blocksch. park.låsn	Block diagram park. lock	628

Design checked by	Drawing checked by	Drwg no.	Rev ind	Sheet
ASG	ASG	314 3787		1

Dept	Drawn by	Year Week	No of sh
421	A Sundberg	97 20	1

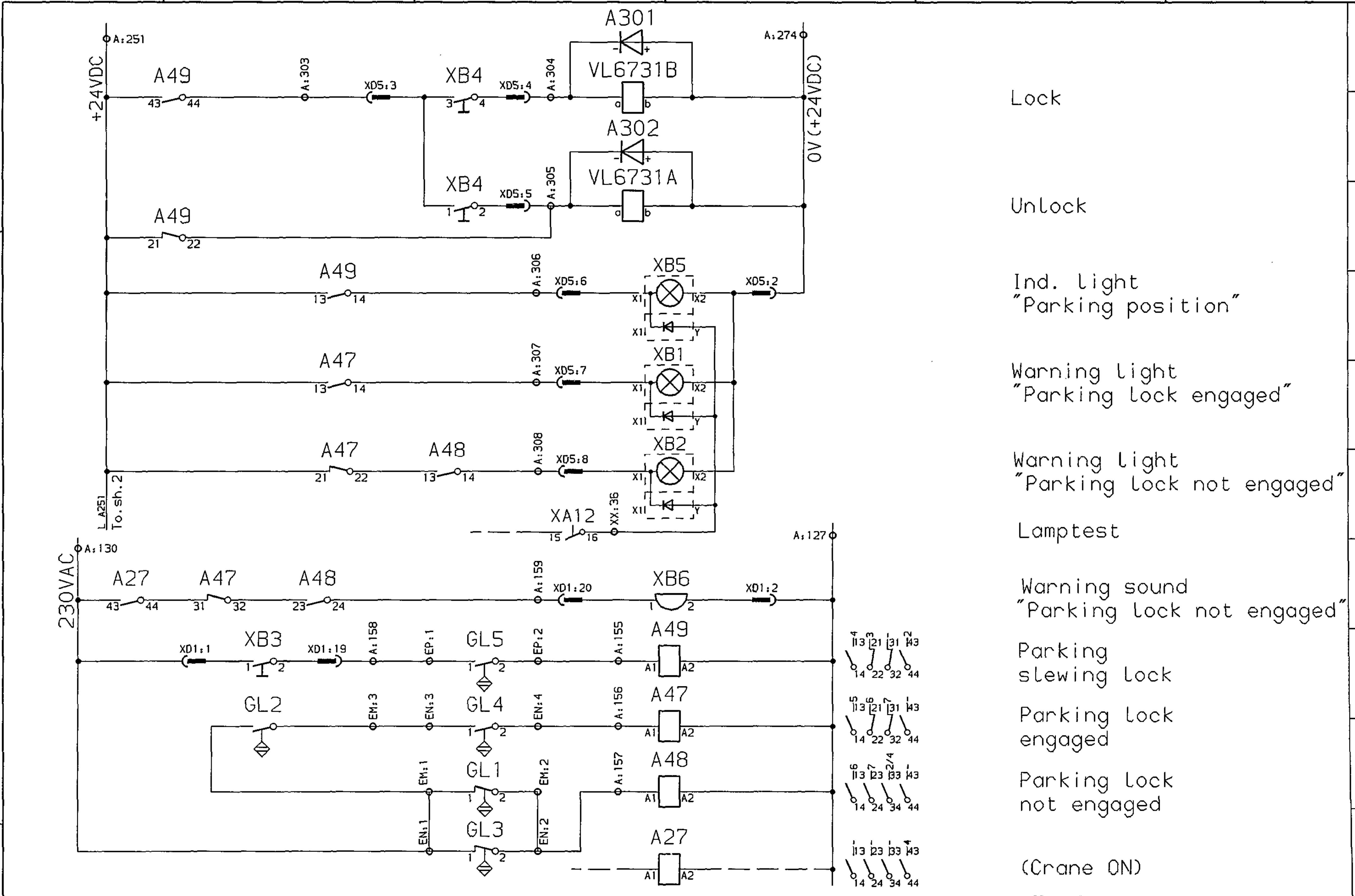

This document must not be copied without our written permission, and the contents thereof must not be imparted to a third party nor be used for any unauthorized purpose. Contravention will be prosecuted.

Reference: KR97012

Similar drawing no.: 314 3764

Rev Ind	Revision	Appd	Year	Week
A	383527	ARA	97	36

MacGREGOR HÄGGLUNDS
MEMBER OF THE INCENTIVE GROUP

Description (own language)	Kretssch. park.låsn.
Description (English)	Circuit diagram park. lock
Prod.group	628
Design checked by	ASG
Drawing checked by	ASG
Dept	421
Drawn by	A Sundberg
Year Week	97 20
Drwg no.	314 3788
Rev ind	A
Sheet	1
No of sh	2

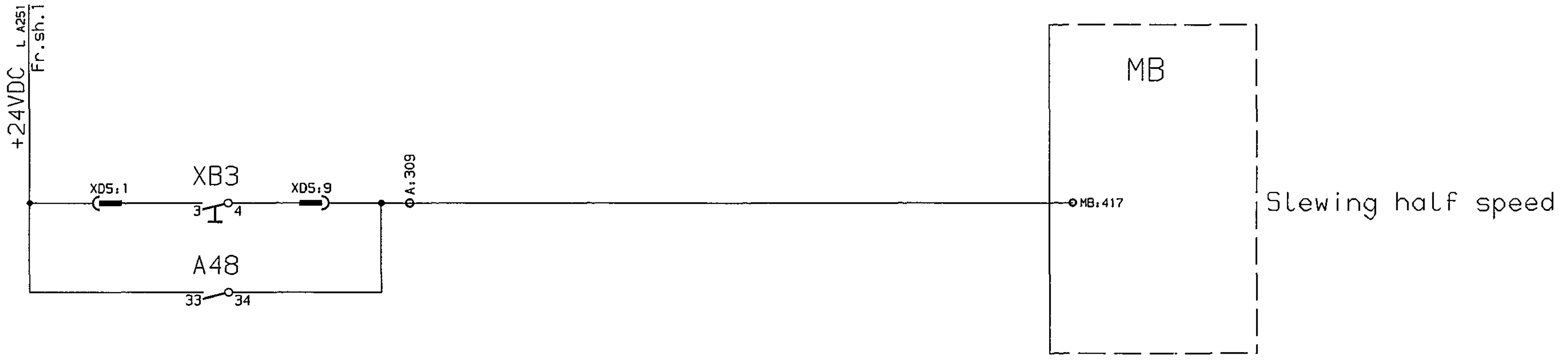

This document must not be copied without our written permission, and the contents thereof must not be imparted to a third party nor be used for any unauthorized purpose. Contravention will be prosecuted.

Reference: KR97012

Similar drawing no.: 314 3764

Revind	Revision	Appd	Year	Week
A	383527	ARA	97	36

MacGREGOR HÄGGLUNDS
MEMBER OF THE INCENTIVE GROUP

Field	Value
Description (own language)	Kretssch. park.låsn
Description (English)	Circuit diagram park. lock
Prod.group	628
Design checked by	ASG
Drawing checked by	ASG
Dept	421
Drawn by	A Sundberg
Year Week	97 20
Drwg no.	314 3788
Rev ind	A
Sheet	2
No of sh	2

TOL. SYSTEM ACC. TO ISO	General tol. for dimensions without tol. indications
Surface roughness R_a in µm	Machining ISO 2768 tol-class:
	Welding DIN 8570 tol-class:

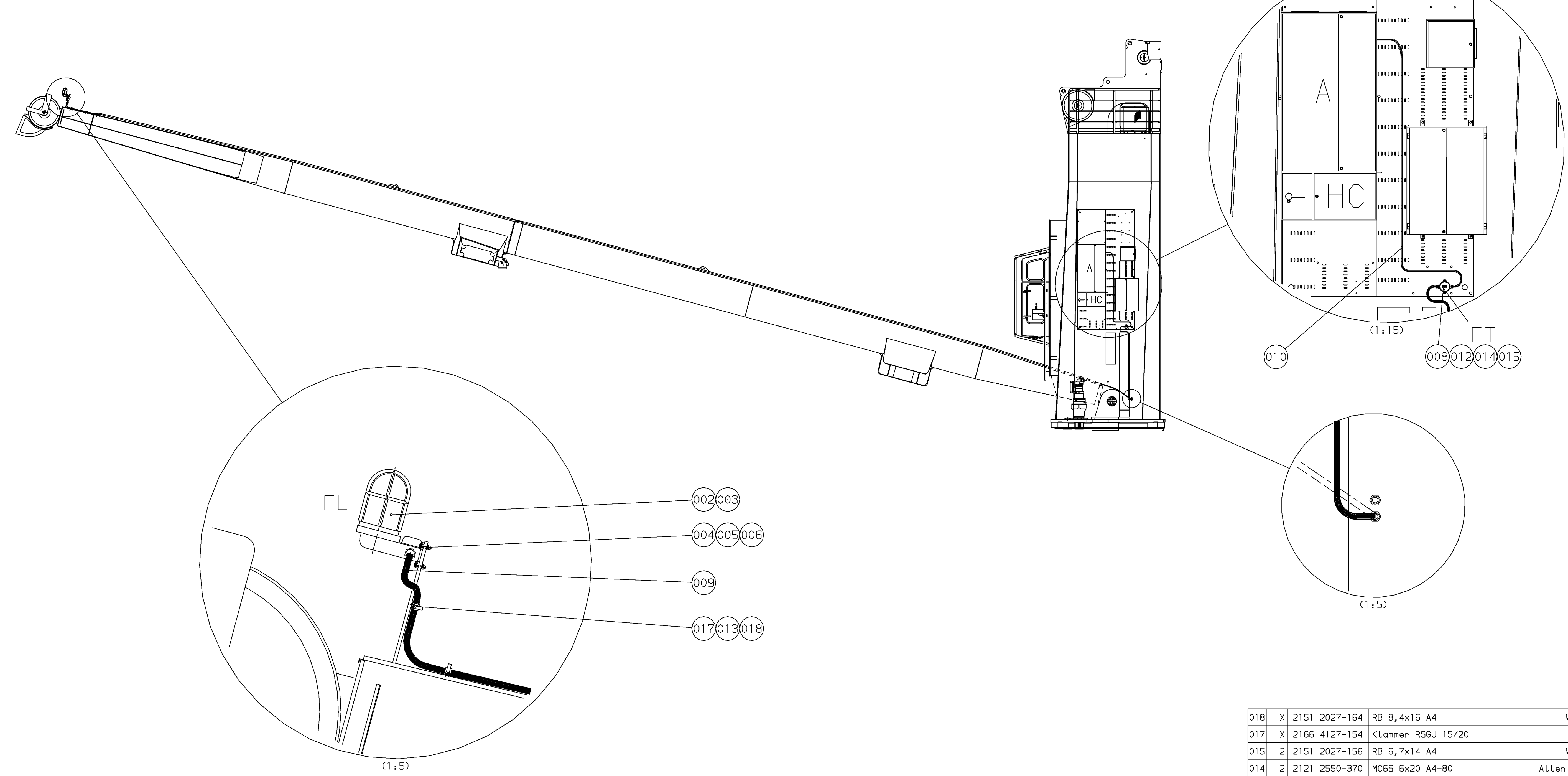

Item no.	Qty/801	Article no.	Description (own language)	Dimensions	Description (English)
018	X	2151 2027-164	RB 8,4x16 A4		Washer
017	X	2166 4127-154	Klammer RSGU 15/20		Clamp
015	2	2151 2027-156	RB 6,7x14 A4		Washer
014	2	2121 2550-370	MC6S 6x20 A4-80		Allen screw
013	X	2126 2634-118	Låsm-M6M 8-A4-80		Locking nut
012	1	2166 2051-823	Kabelförskr. Pr 22,5		Cable gland
010	1	1684 2136-123	Elkabel RU 3x1,5	L=4m	Cable
009	X	1686 2136-134	Elkabel A07RN-F 4x2,5	L=40m	Cable
008	1	5388 4121-002	Kopplingsdosa		Connection box
006	X	2151 2027-156	RB 6,7x14 A4		Washer
005	X	2126 2634-116	Låsm-M6M 6-A4-80		Locking nut
004	X	2121 2550-374	MC6S 6x30 A4-80		Allen screw
003	X	5911 4528-066	Glödlampa 60W B22		Bulb
002	X	5915 4251-021	Skottarmatur MA279 röd		Armature
001	X	414 6823-000	Förb. tabell varningsljus		Wiring table

Rev ind	Revision	Appd	Year Week
B	385026	LBL	99 03
A	383510	LSK	97 36

Design checked by	Accepted by qual dept	Part of	
Drawing checked by ASg	Accepted for prod by	Specification	Scale 1:50
Dept 421	Drawn by A Sundberg	Year Week 96 39	Description (English) El. inst. warninglight jib	Weight kg
MacGREGOR HÄGGLUNDS MEMBER OF THE INCENTIVE GROUP		Description (own language) El inst. varningsljus arm	Prod.group 628
		Drwg no. 114 1234	Rev ind B · Sheet 1 · No of sh 1

This document must not be copied without our written permission, and the contents thereof must not be imparted to a third party nor be used for any unauthorized purpose. Contravention will be prosecuted.

Reference K096058

Similar drawing No. 114 0811-801

VARVSMONTERING/SHIP YARD MOUNTING

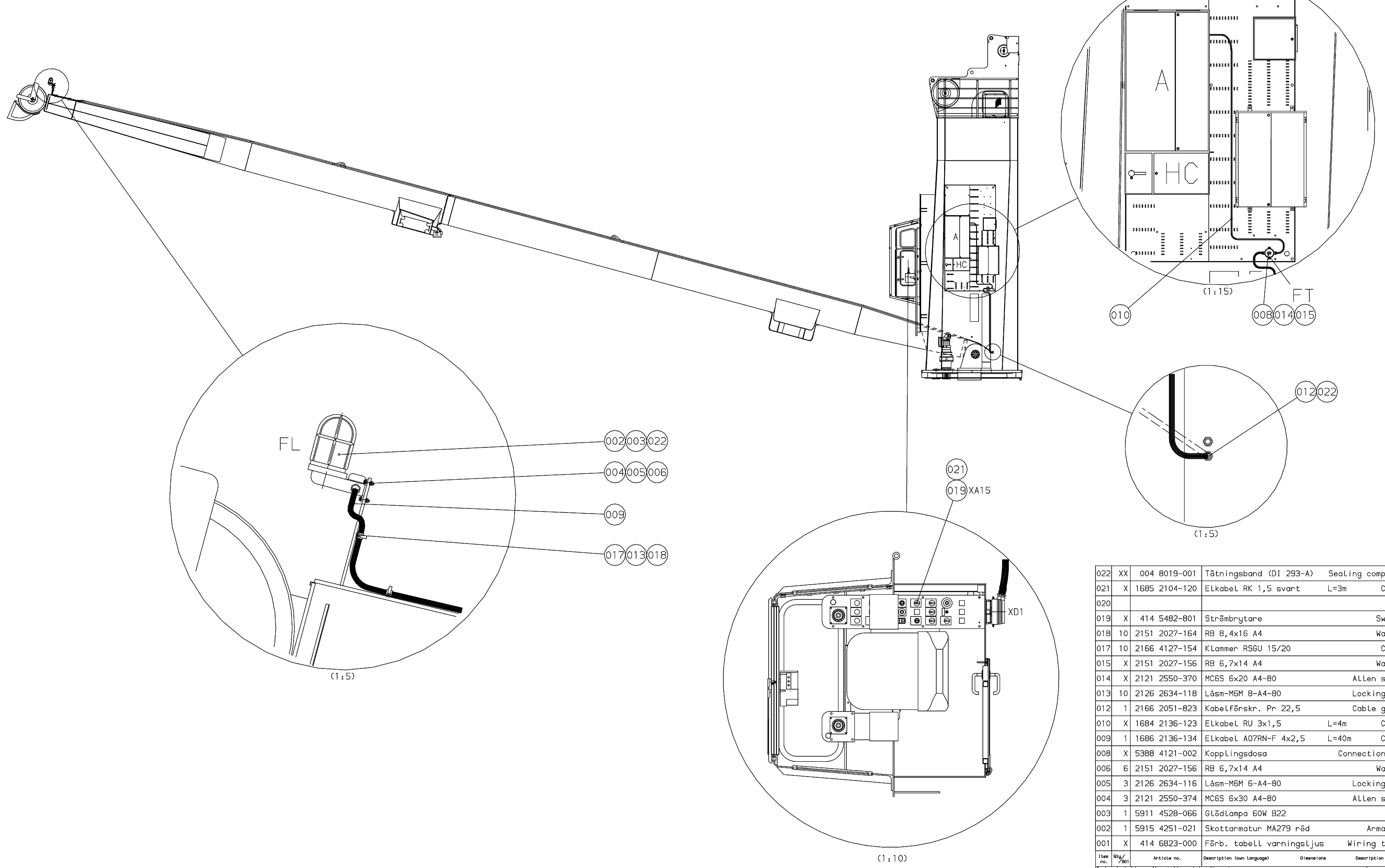

TOL. SYSTEM ACC. TO ISO	General tol. for dimensions without tol. indications
Surface roughness R_a in µm	Machining ISO 2768 tol-class:
	Welding DIN 8570 tol-class:

Item no.	Qty/801	Article no.	Description (own language)	Dimensions	Description (English)
022	XX	004 8019-001	Tätningsband (DI 293-A)		Sealing compound
021	X	1685 2104-120	Elkabel RK 1,5 svart	L=3m	Cable
020					
019	X	414 5482-801	Strömbrytare		Switch
018	10	2151 2027-164	RB 8,4x16 A4		Washer
017	10	2166 4127-154	Klammer RSGU 15/20		Clamp
015	X	2151 2027-156	RB 6,7x14 A4		Washer
014	X	2121 2550-370	MC6S 6x20 A4-80		Allen screw
013	10	2126 2634-118	Låsm-M6M 8-A4-80		Locking nut
012	1	2166 2051-823	Kabelförskr. Pr 22,5		Cable gland
010	X	1684 2136-123	Elkabel RU 3x1,5	L=4m	Cable
009	1	1686 2136-134	Elkabel A07RN-F 4x2,5	L=40m	Cable
008	X	5388 4121-002	Kopplingsdosa		Connection box
006	6	2151 2027-156	RB 6,7x14 A4		Washer
005	3	2126 2634-116	Låsm-M6M 6-A4-80		Locking nut
004	3	2121 2550-374	MC6S 6x30 A4-80		Allen screw
003	1	5911 4528-066	Glödlampa 60W B22		Bulb
002	1	5915 4251-021	Skottarmatur MA279 röd		Armature
001	X	414 6823-000	Förb. tabell varningsljus		Wiring table

Design checked by	Accepted by qual dept	Part of	
Drawing checked by: ASg	Accepted for prod by	Specification	Scale: 1:50
Dept: 421 / Drawn by: A Sundberg / Year Week: 96 39		Description (English): El. inst. warninglight jib	Weight kg
MacGREGOR HÄGGLUNDS MEMBER OF THE INCENTIVE GROUP		Description (own language): El inst. varningsljus arm	Prod.group: 628
		Drwg no.: 114 1235	Rev ind: A / Sheet: 1 / No of sh: 1

Rev ind	Revision	Appd	Year	Week
A	385026	LBL	99	03

This document must not be copied without our written permission, and the contents thereof must not be imparted to a third party nor be used for any unauthorized purpose. Contravention will be prosecuted.

Reference: K096058

Similar drawing no.: 114 0811-802

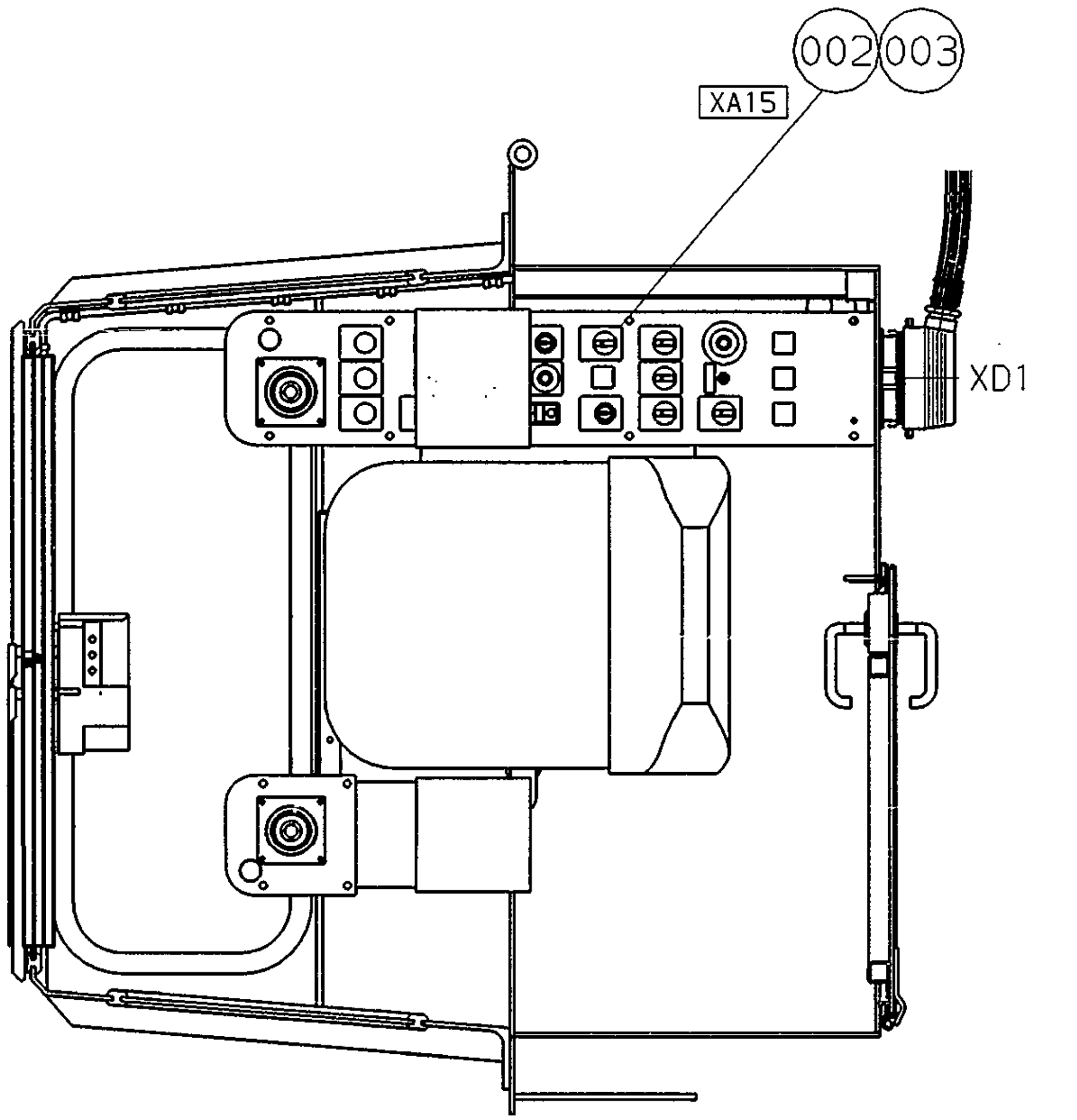

TOL. SYSTEM ACC. TO ISO	General tol. for dimensions without tol. indications
Surface roughness R_a in µm	Machining ISO 2768 tol-class:
	Welding DIN 8570 tol-class:

This document must not be copied without our written permission, and the contents thereof must not be imported to a third party nor be used for any unauthorized purpose. Contravention will be prosecuted.

Item no.	Qty/801	Article no.	Description (own language)	Dimensions	Description (English)
003	1	1685 2104-120	Elkabel RK 1,5 svart	L=3m	Cable
002	1	414 5482-801	Strömbrytare		Switch
001		414 6823-000	Förb.tab varningsl.		Wiring tab.

Reference	KR97012
Similar drawing no.	314 3756

Design checked by	Accepted by qual dept	Part of	
ASG		Order spec !	
Drawing checked by	Accepted for prod by	Specification	Scale
ASG	MN		1:10
Dept / Drawn by / Year Week		Description (English)	Weight kg
421 L-E Söderbäck 97 36		El.inst cab.warningl.jib	
		Description (own language)	Prod.group
		El.inst hytt varningsl.	628
		Drwg no.	Rev ind / Sheet
		314 3908	1 / No of sh 1

MacGREGOR HÄGGLUNDS
MEMBER OF THE INCENTIVE GROUP

RevInd	Revision	Appd	Year	Week

TOL. SYSTEM ACC. TO ISO	General tol. for dimensions without tol. indications
Surface roughness R_a in µm	Machining ISO 2768 tol-class:
	Welding DIN 8570 tol-class:

A-A Skala / Scale 1:3

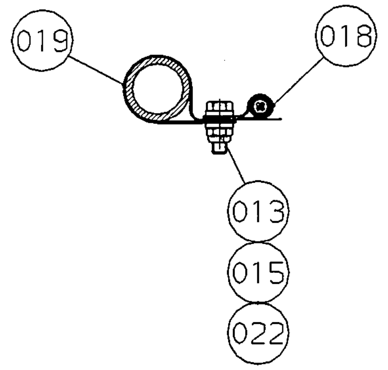

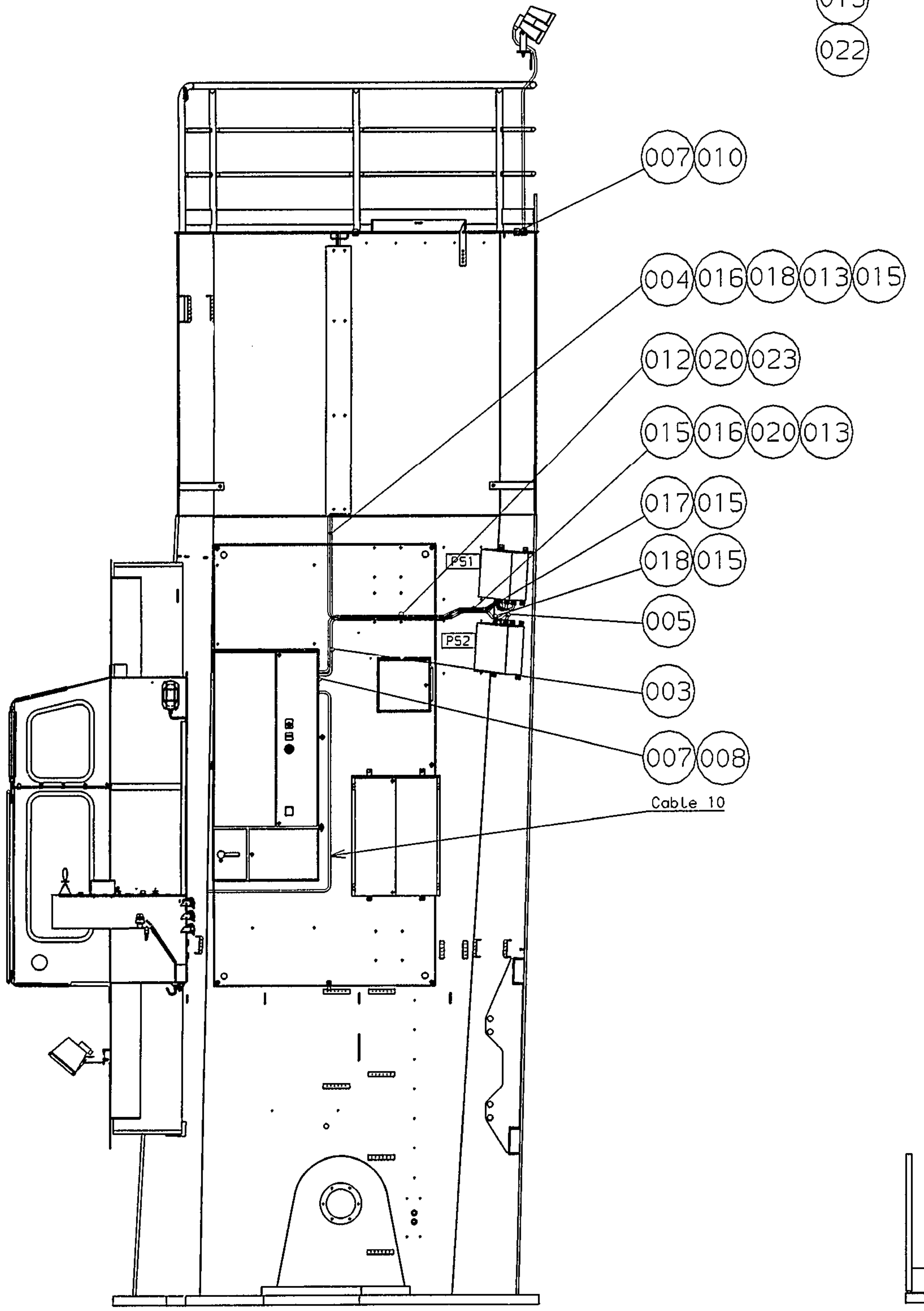

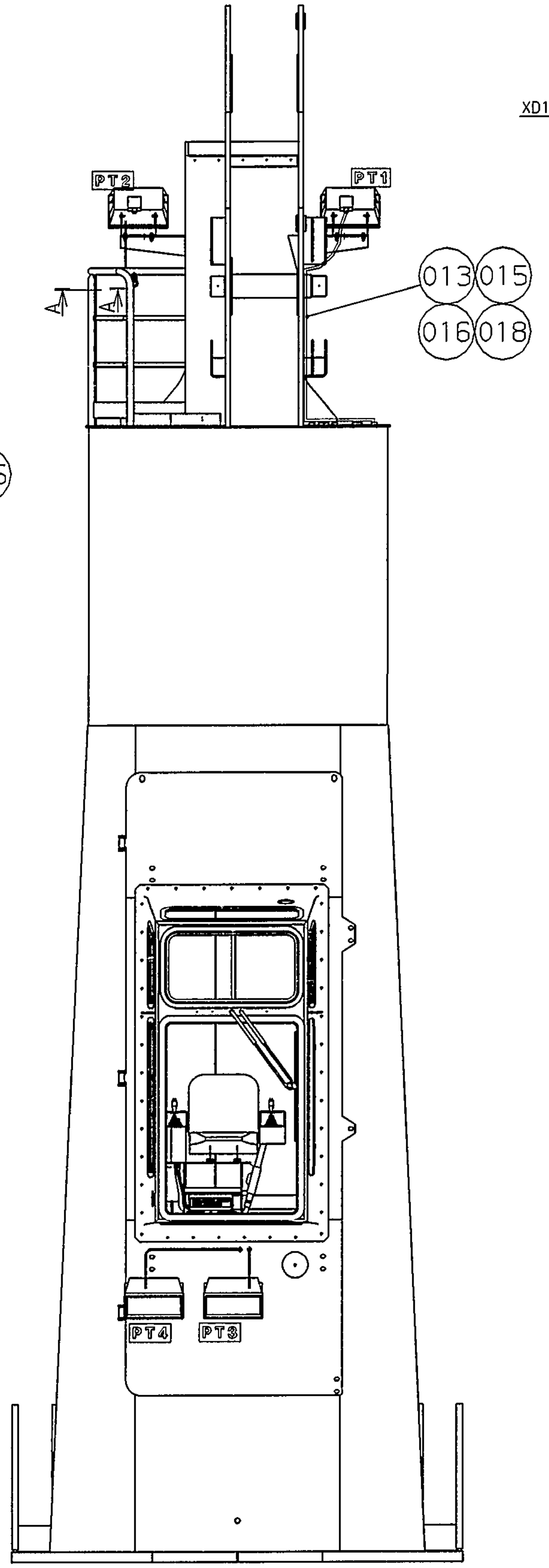

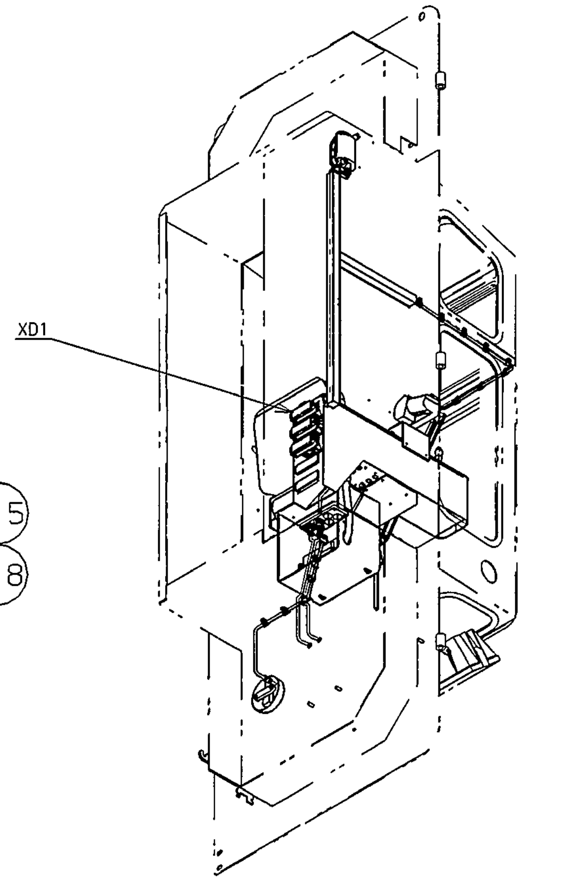

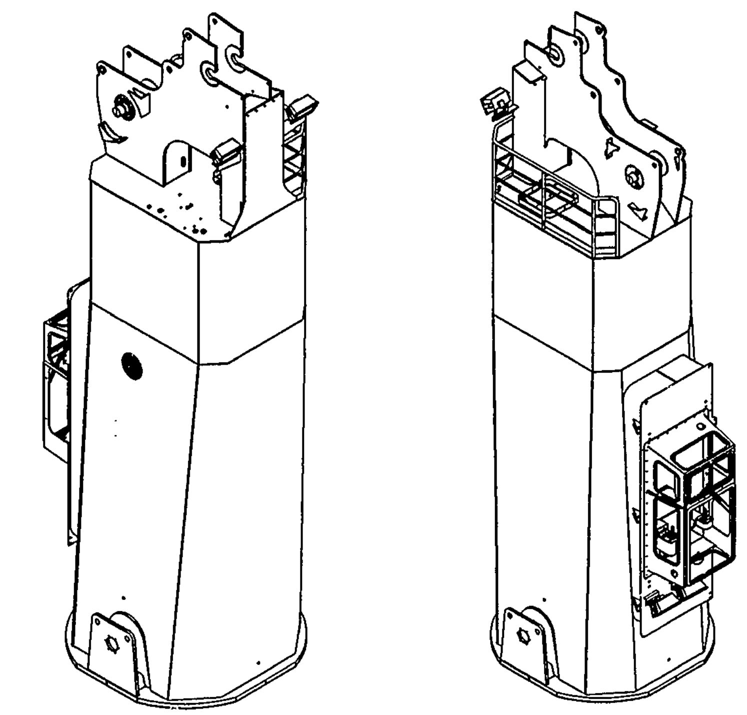

A	383772	LBL	97 49
Rev ind	Revision	Appd	Year Week

Item no.	Qty/801	Article no.	Description (own language)	Dimensions	Description (English)

Design checked by ASG	Accepted by qual dept	Part of	
Drawing checked by ASG	Accepted for prod by MN	Specification 214 1801	Scale 1:30
Dept 421 / Drawn by A Sundberg/LB / Year Week 97 23		Description (English) EL. inst. crane deck light	Weight kg
		Description (own language) El inst. kran däcksbel.	Prod.group
MacGREGOR HÄGGLUNDS MEMBER OF THE INCENTIVE GROUP		Drwg no. 214 1801	Rev ind A / Sheet 1 / No of sh 1

BILDKORT

This document must not be copied without our written permission, and the contents thereof must not be imparted to a third party nor be used for any unauthorized purpose. Contravention will be prosecuted.

Reference KR96050

Similar drawing No. 114 1289

This document must not be copied without our written permission, and the contents thereof must not be imparted to a third party nor be used for any unauthorized purpose. Contravention will be prosecuted.

Item no.	Article no.	Description (own language)	Dimensions	Description (English)	Qty/801	Note
001	414 7003-000	Förb.tab däcksbelysning		Wiring tab. decklight	x	
003	1684 2136-134	Elkabel RU 4x2,5	L=4m	Cable	1	
004	1686 2136-134	Elkabel A07RN-F 4x2,5	L=27m	Cable	1	
005	1684 2136-137	Elkabel RU 7x2,5	L=4m	Cable	1	
007	2166 2051-823	Kabelförskr. Pr 22,5		Cable gland	3	
008	2166 2051-822	Kabelförskr. Pr 22,5		Cable gland	1	
010	004 8019-001	Tätningsband (DI 293-A)		Sealing compound	x	
012	2151 2022-173	BRB 10.5x22 fzb		Washer	2	
013	2151 2022-164	BRB 8,4x16 fzb		Washer	27	
015	2126 2636-118	Låsm-M6M 8-8 fzb		Locking nut	29	
016	2126 2032-118	M6M 8 -8 FZB		NUT	20	
017	2166 4127-284	Klammer RSGU 28/20		Clamp	1	
018	2166 4127-154	Klammer RSGU 15/20		Clamp	26	
019	2166 4127-404	Klammer RSGU 40/20		Clamp	7	
020	2166 4127-374	Klammer RSGU 37/20		Clamp	4	
022	2121 2032-453	M6S 8x25 -8.8 fzb		Screw	7	
023	2121 2032-491	M6S 10x20 -8.8 fzb		Screw	2	

Rev Ind	Revision	Appd	Year Week
A	383772	LBL	97 49

Design checked by: ASG	Accepted by qual dept: .	Part of	Assembly drawing: 214 1801
Drawing checked by: ASG	Accepted for prod by: MN	Description (English): EL. inst. crane deck ligh	
Dept: 421	Drawn by: B Andersson/LB	Year Week: 97 23	Description (own language): El inst. kran däcksbel.
MacGREGOR HÄGGLUNDS, MEMBER OF THE INCENTIVE GROUP	Drwg no.: 214 1801	Rev Ind: A	Sheet: 1, No of sh: 1

TOL. SYSTEM ACC. TO ISO	General tol. for dimensions without tol. indications
Surface roughness R_a in µm	Machining ISO 2768 tol-class: Welding DIN 8570 tol-class:

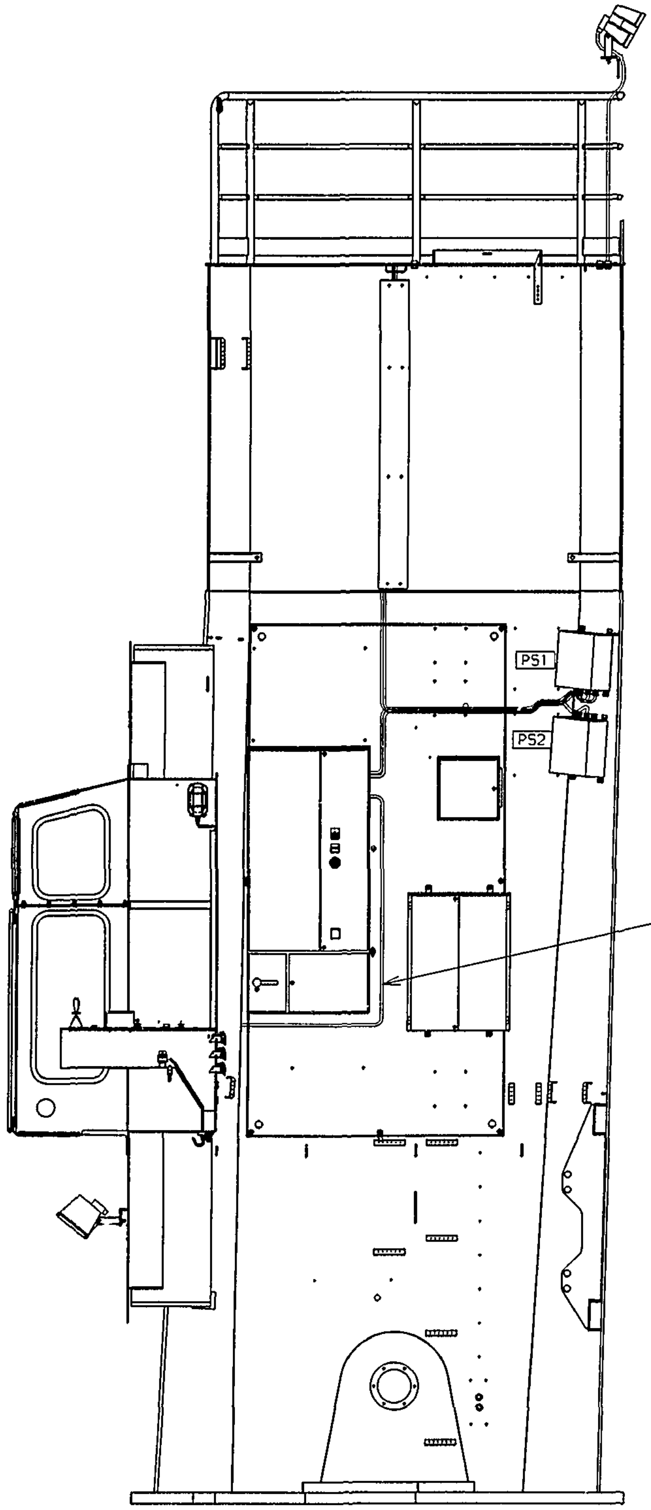

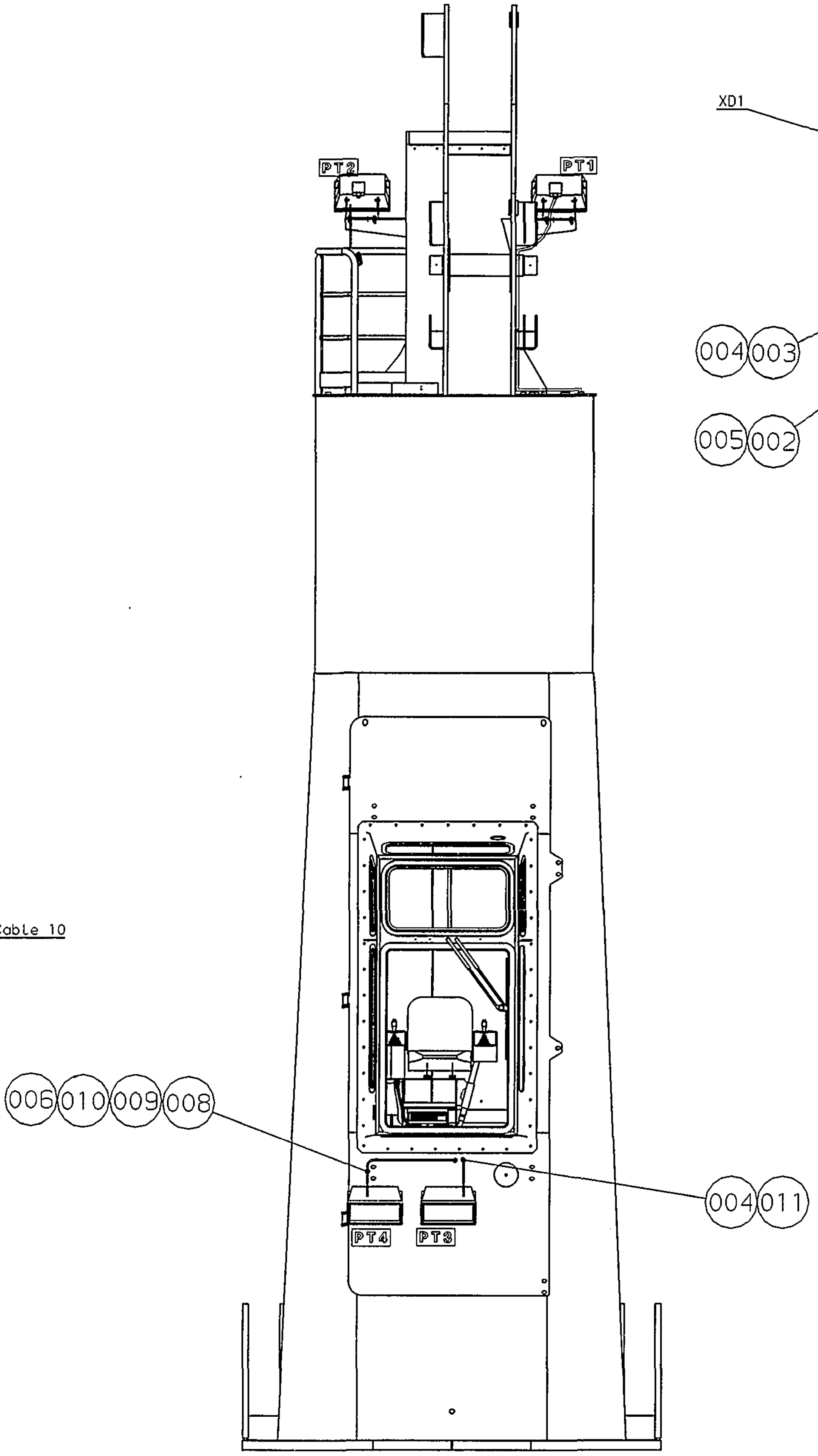

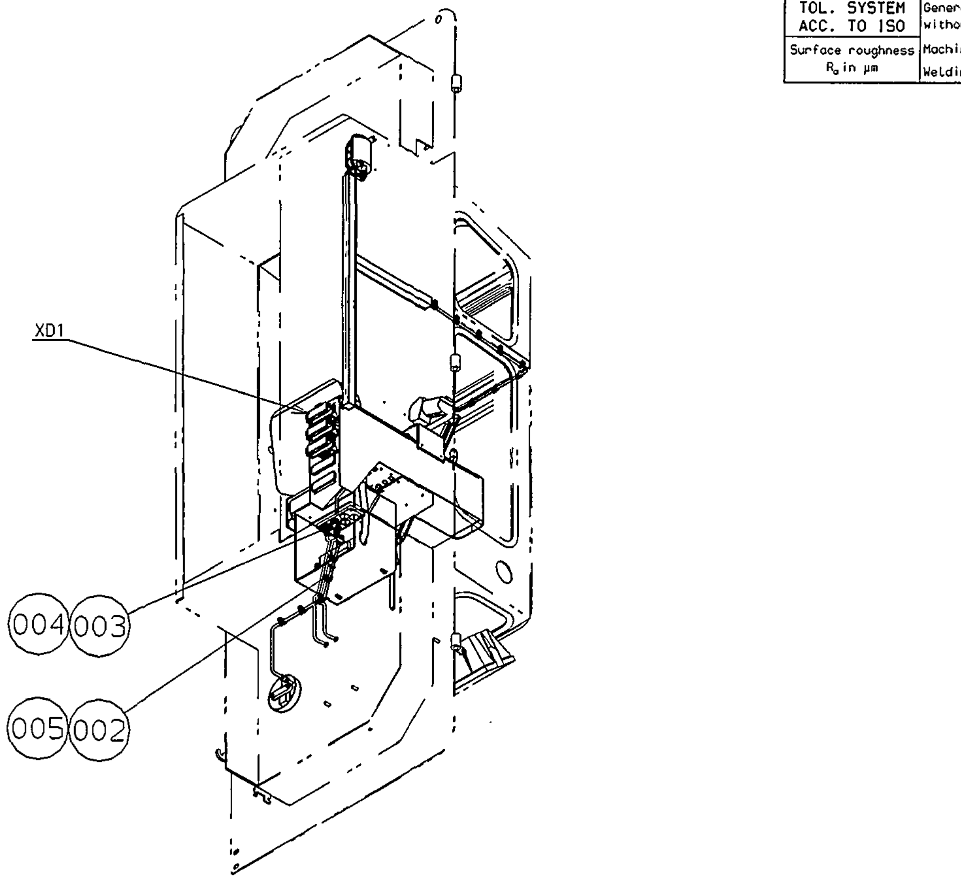

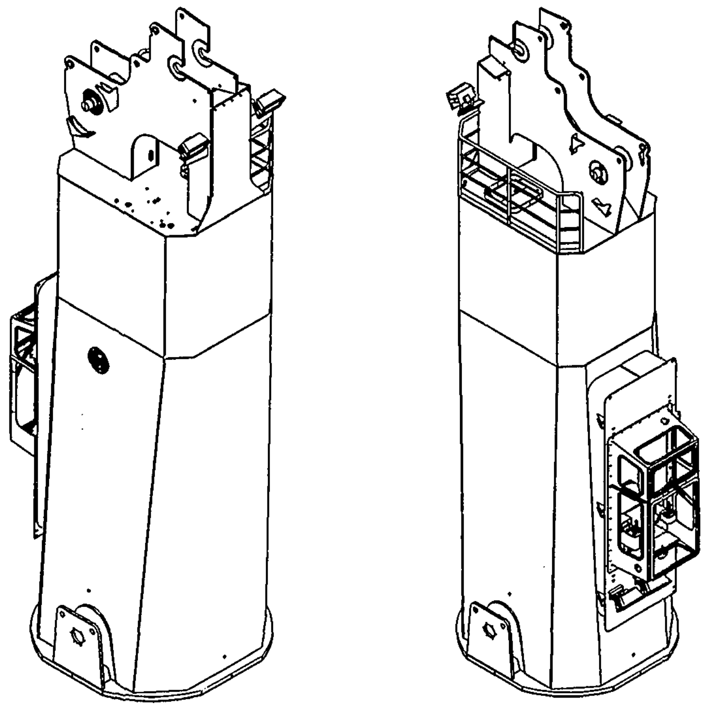

BILDKORT

This document must not be copied without our written permission, and the contents thereof must not be imparted to a third party nor be used for any unauthorized purpose. Contravention will be prosecuted.

Reference KR96050

Similar drawing No. 114 1327

Item no.	Qty/B01	Article no.	Description (own language)	Dimensions	Description (English)

Design checked by: ASG	Accepted by qual dept	Part of	
Drawing checked by: ASG	Accepted for prod by: MN	Specification: 214 1800	Scale: 1:30
Dept: 421	Drawn by: A Sundberg/LB, Year Week: 97 23	Description (English): El. inst. cabin deck light	Weight kg
		Description (own language): El inst. hytt däcksbel.	Prod.group
MacGREGOR HÄGGLUNDS MEMBER OF THE INCENTIVE GROUP		Drwg no.: 214 1800	Rev ind: A; Sheet: 1; No of sh: 1

Revind	Revision	Appd	Year Week
A	383772	LBL	97 49

This document must not be copied without our written permission, and the contents thereof must not be imparted to a third party nor be used for any unauthorized purpose. Contravention will be prosecuted.

Item no.	Article no.	Description (own language)	Dimensions	Description (English)	Qty/801	Note
001	414 7003-000	Förb.tab däcksbelysning		Wiring tab. decklight	x	
002	1684 2136-123	Elkabel RU 3x1,5	L=6m	Cable	1	
003	2686 4425-371	Reduc-muff Pr 37,0-22,5		Reducing nipple	2	
004	2166 2051-823	Kabelförskr. Pr 22,5		Cable gland	4	
005	2166 4127-224	Klammer RSGU 22/20		Clamp	4	
006	2166 4127-104	Klammer RSGU 10/20		Clamp	6	
008	2151 2022-164	BRB 8,4x16 fzb		Washer	6	
009	2126 2636-118	Låsm-M6M 8-8 fzb		Locking nut	6	
010	2126 2032-118	M6M 8 -8 FZB		NUT	6	
011	004 8019-001	Tätningsband (DI 293-A)		Sealing compound	x	

Rev Ind	Revision	Appd	Year Week
A	383772	LBL	97 49

Design checked by: ASG	Accepted by qual dept: .	Part of	Assembly drawing: 214 1800
Drawing checked by: ASG	Accepted for prod by: MN	Description (English): El. inst. cabin deck ligh	
Dept: 421	Drawn by: B Andersson/LB	Year Week: 97 23	Description (own language): El inst. hytt däcksbel.

MacGREGOR HÄGGLUNDS
MEMBER OF THE INCENTIVE GROUP

Drwg no.	Rev Ind	Sheet	No of sh
214 1800	A	1	1

TOL. SYSTEM ACC. TO ISO	General tol. for dimensions without tol. indications
Surface roughness R_a in µm	Machining ISO 2768 tol-class:<br>Welding DIN 8570 tol-class:

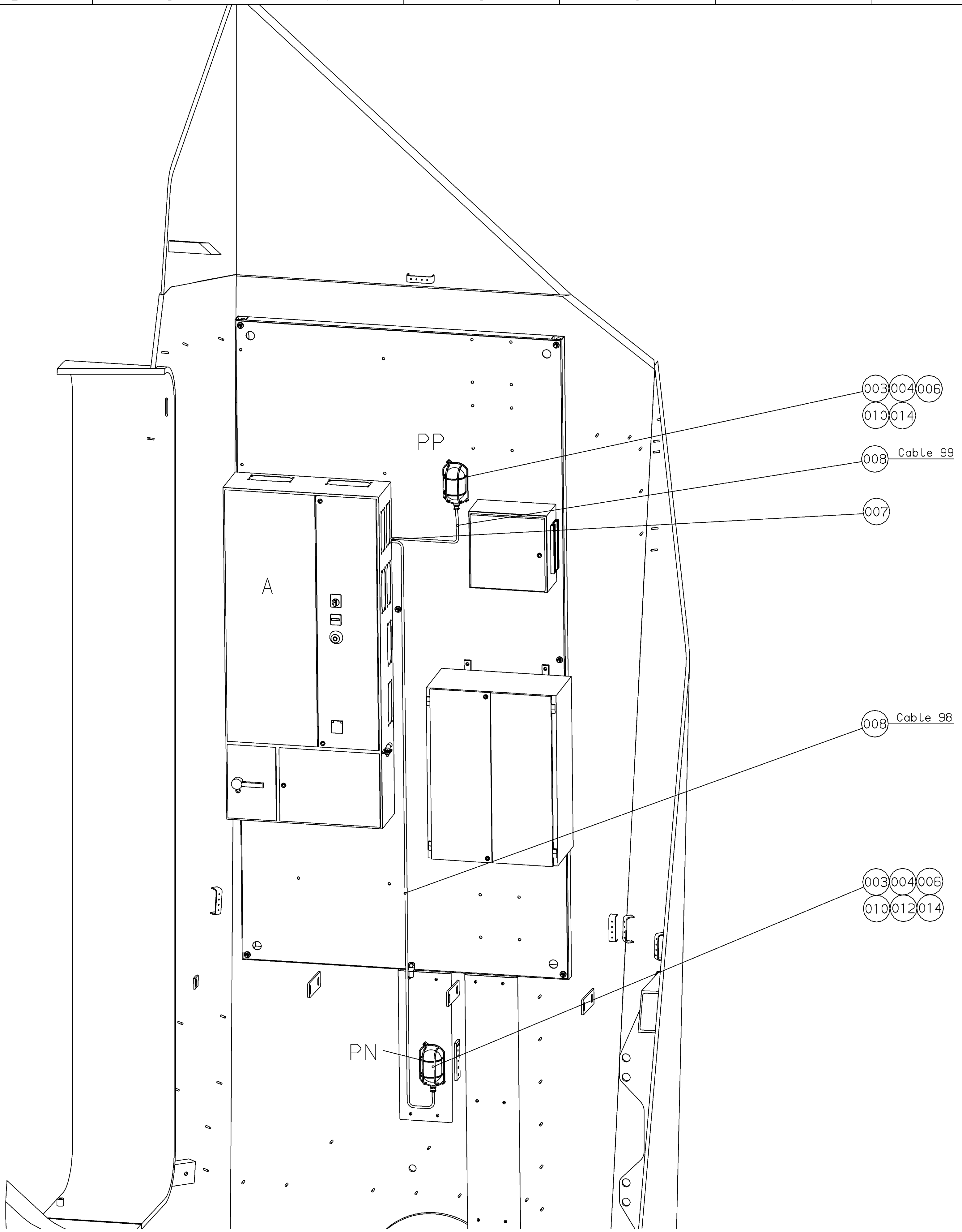

Scale 1:10

Item no.	Qty/801	Article no.	Description (own language)	Dimensions	Description (English)
014	6	2151 2025-153	BRB 6.4x12 RF		Washer
012	2	2126 2636-116	Nyloc-M6M 6 -8 fzb		Locking nut
010	4	2121 2550-370	MC6S 6x20 A4-80		Allen screw
008	1	1684 2136-123	Elkabel RU 3x1,5	L=7m	Cable
007	2	2166 2051-821	Kabelförskr. Pr 22,5		Cable gland
006	2	2166 2051-813	Kabelförskr. Pr 18,6		Cable gland
004	2	5911 4145-060	Lampa matt 220V,60W,E27		Lampbulb
003	2	5915 4262-002	Takarmatur 60 W		Ceiling light
001	x	414 7031-000	Förb.tab nödbel.		Wiring table emerg. light

RevInd	Revision	Appd	Year Week
A	385091	LBL	99 07

Design checked by:
Accepted by qual dept:
Part of:
Drawing checked by:
Accepted for prod by:
Specification:
Scale: 1:20
Dept: 421 Drawn by: A Sundberg/LB Year Week: 97 23
Description (English): El. inst. emerge
Weight kg:
Description (own language): El inst. nödljus
Prod.group: 628
Drwg no.: 114 1325
Rev ind: A
Sheet: 1
No of sh: 1

MacGREGOR HÄGGLUNDS
MEMBER OF THE INCENTIVE GROUP

Similar drawing No.
Reference: KR96050

This document must not be copied without our written permission, and the contents thereof must not be imparted to a third party nor be used for any unauthorized purpose. Contravention will be prosecuted.

TOL. SYSTEM ACC. TO ISO	General tol. for dimensions without tol. indications
Surface roughness R_a in µm	Machining ISO 2768 tol-class:
	Welding DIN 8570 tol-class:

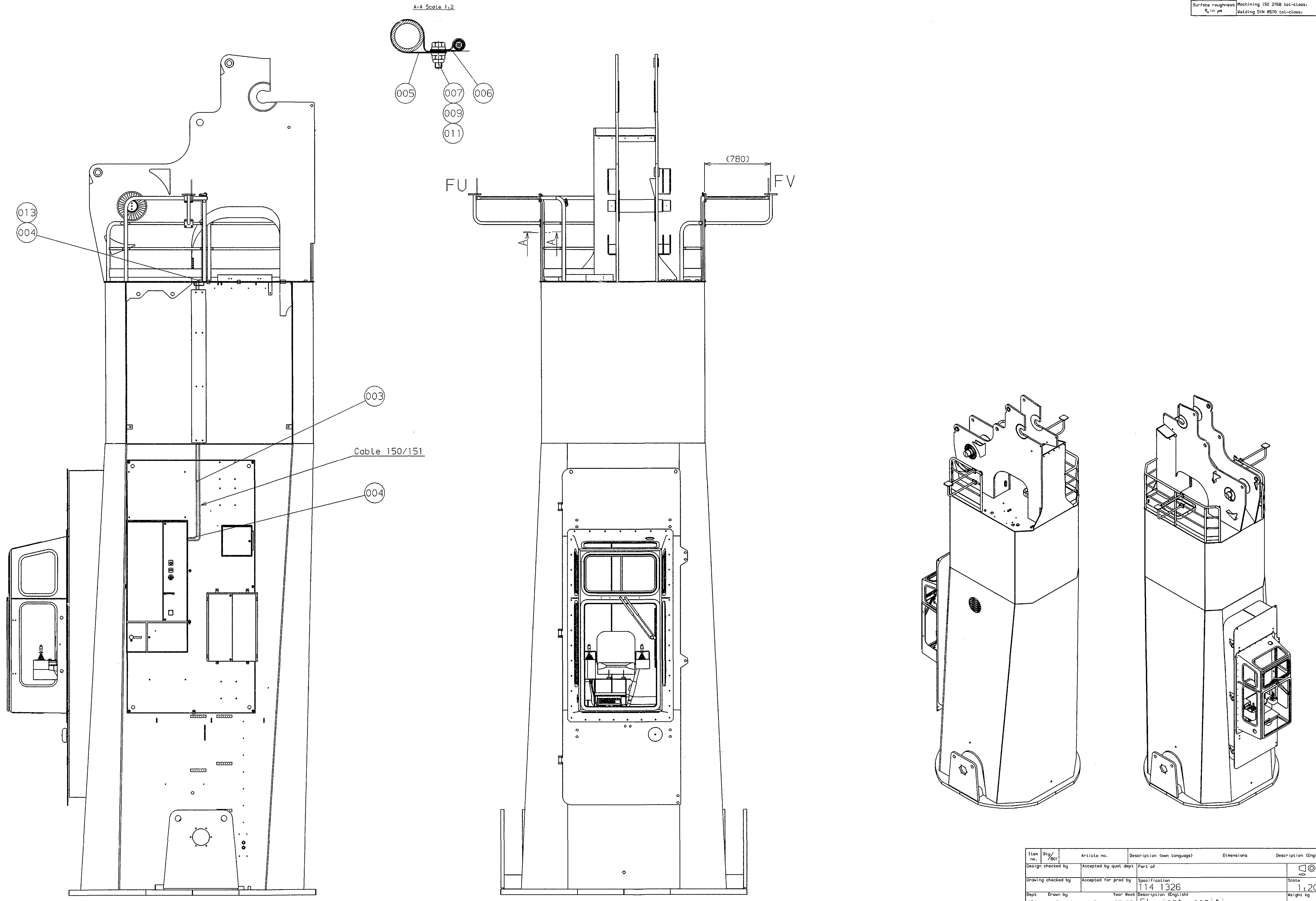

This document must not be copied without our written permission, and the contents thereof must not be imparted to a third party nor be used for any unauthorized purpose. Contravention will be prosecuted.

Reference KR96050

Similar drawing No. 114 1023

Item no.	Qty/801	Article no.	Description (own language)	Dimensions	Description (English)

Design checked by	Accepted by qual dept	Part of	
Drawing checked by	Accepted for prod by	Specification 114 1326	Scale 1:20
Dept 421	Drawn by A Sundberg/LB, Year 97 Week 27	Description (English) El. inst. positi	Weight kg
**MacGREGOR HÄGGLUNDS** MEMBER OF THE INCENTIVE GROUP		Description (own language) El inst. positionsljus	Prod.group 628
		Drwg no. 114 1326	Rev ind; Sheet 1; No of sh 1

Rev ind	Revision	Appd	Year	Week
